# Microarray-Based Genotyping and Clinical Outcomes of *Staphylococcus aureus* Bloodstream Infection: An Exploratory Study

**DOI:** 10.1371/journal.pone.0071259

**Published:** 2013-08-14

**Authors:** Siegbert Rieg, Daniel Jonas, Achim J. Kaasch, Christine Porzelius, Gabriele Peyerl-Hoffmann, Christian Theilacker, Marc-Fabian Küpper, Christian Schneider, Harald Seifert, Winfried V. Kern

**Affiliations:** 1 Department of Medicine, Center for Infectious Diseases and Travel Medicine and IFB-Center for Chronic Immunodeficiency, University Medical Center Freiburg, Freiburg, Germany; 2 Department of Environmental Health Sciences, University Medical Center Freiburg, Freiburg, Germany; 3 Institute for Medical Microbiology, Immunology and Hygiene, University of Cologne, Cologne, Germany; 4 Institute of Medical Biometry and Medical Informatics, University Medical Center Freiburg, Freiburg, Germany; 5 Institute for Medical Informatics, Statistics and Epidemiology, University of Leipzig, Leipzig, Germany; 6 Institute of Medical Microbiology and Hygiene, University Medical Center Freiburg, Freiburg, Germany; Rockefeller University, United States of America

## Abstract

The clinical course of *Staphylococcus aureus* bacteremia varies extensively. We sought to determine the relationship between genetic characteristics of the infecting pathogen and clinical outcomes in an exploratory study. In two study centers, 317 blood culture isolates were analyzed by DNA microarray and *spa* genotyping. By uni- and multivariate regression analyses associations of genotype data with 30-day all-cause mortality, severe sepsis/septic shock, disseminated disease, endocarditis, and osteoarticular infection were investigated. Univariate analysis showed significant association between *S. aureus* genes/gene-clusters or clonal complexes and clinical endpoints. For example CC15 was associated with 30-day mortality and CC22 with osteoarticular infection. In multivariate analysis methicillin resistance (*mecA,* OR 4.8 [1.43–16.06]) and the beta-lactamase-gene (*bla*, OR 3.12 [1.17–8.30]) remained independently associated with 30-day mortality. The presence of genes for enterotoxins (*sed/sej/ser*) was associated with endocarditis (OR 5.11 [1.14–18.62]). Host factors such as McCabe classification (OR 4.52 [2.09–9.79] for mortality), age (OR 1.06 [1.03–1.10] per year), and community-acquisition (OR 3.40 [1.31–8.81]) had a major influence on disease severity, dissemination and mortality. Individual genotypes and clonal complexes of *S. aureus* can only partially explain clinical features and outcomes of *S. aureus* bacteremia. Genotype-phenotype association studies need to include adjustments for host factors like age, comorbidity and community-acquisition.

## Introduction

*Staphylococcus aureus* remains a major human pathogen that is able to cause a wide spectrum of clinical manifestations ranging from asymptomatic carriage to localized or disseminated infections, such as skin and soft tissue infections (SSTI), bone and joint infections, and infective endocarditis [1].

*S. aureus* is equipped with a broad range of virulence factors including exoproteins and proteases, superantigens and toxins, microbial surface components recognizing adhesive matrix molecules (MSCRAMMs) and adhesins. Moreover, *S. aureus* is capable to circumvent innate and adaptive host defences by multiple immune evasion strategies and to withstand antibiotics due to acquisition of diverse resistance mechanisms [2]. The question whether particular virulence genes, resistance determinants, or specific clonal lineages of *S. aureus* predispose to certain clinical manifestations and are associated with poor outcome has raised substantial interest [3].

A range of studies identified genotypic *S. aureus* factors likely to contribute to invasive disease by comparing *S. aureus* isolates recovered from the anterior nares of healthy carriers with isolates obtained from patients with different clinical disease manifestations [4]–[10]. In contrast, studies comparing the genetic determinants of isolates from patients with different clinical disease manifestations and/or poor versus good outcomes have been rare and hampered by small isolate numbers [11], non-inclusion of MSSA [12], paucity of clinical data [13] and few genes investigated [14]. Using microarray techniques, the presence of several hundred genes within an isolate can now be readily determined, thus enabling a comprehensive study of putative *S. aureus* virulence and resistance determinants [15].

The aim of the current study was to explore whether the genetic makeup of *S. aureus* can be linked with mortality, infection severity, metastatic disease, and tropism for endovascular and osteoarticular sites of infection. For this purpose we used clinical data and isolates from a large prospective cohort study of SAB at two German university hospitals.

## Patients and Methods

### Patients

The study was a non-interventional observational study conducted at the University Medical Centers Freiburg (1.500-bed tertiary care center) and Cologne (1.300 bed tertiary care center) within the framework of an ongoing quality assurance programme at both institutions. Blood cultures growing *S. aureus* were reported daily by the microbiology laboratory, and adult patients were assessed by infectious disease physicians onsite who recommended intensified diagnostic studies and optimized treatment if needed. The patients were usually followed until discharge from the hospital. For each case relevant clinical and microbiological data were entered into a database that included age, sex, underlying medical conditions, clinical signs and symptoms at SAB onset, diagnostic and therapeutic procedures, antimicrobial therapy, complications and outcomes. Outcomes after discharge were routinely assessed by active case finding, by contact with the primary care physician, or by assessing the patient in the infectious diseases outpatient clinics.

For the present analysis we retrospectively chose every second case seen between January 2006 and May 2010 for which clinical data was complete and the bacterial isolate was available for further study. The subgroup of SAB cases included in the study was representative of the complete cohort with respect to underlying diseases, proportions of MRSA isolates, endocarditis, disseminated disease as well as 30-day all-cause mortality (File S1). The study and data collection were approved by the Institutional Review Boards of the University Medical Centers Freiburg and Cologne. Written informed consent was obtained from the patients at the University Medical Center Cologne. The Institutional Review Board of the University Medical Center Freiburg considered the investigation as evaluation of service within a quality assurance programme and waived the need for written informed consent.

### Clinical definitions

Mode of acquisition (community-acquired, community-onset healthcare-associated or hospital-acquired SAB) was defined as previously described [16]. Severity of illness was assessed by the McCabe-Jackson classification. Intravascular catheter/device-related SAB was considered when clinical signs of catheter/device-infection and/or positive culture results of catheter material were present without evidence of an alternative source of SAB. Infective endocarditis was diagnosed according to the modified Duke criteria. Metastatic lesions were actively sought by clinical examination and imaging studies. Severe sepsis and septic shock were defined according to definitions of the ACCP/SCCM consensus conference and was considered an SAB-related outcome when present within 48 h before and 72 h after the first positive blood culture was drawn. Osteoarticular infection (defined as SAB with vertebral/non-vertebral osteomyelitis or septic arthritis) was considered as documented focus if there were suggestive clinical and pathological findings, evidence from imaging studies and if *S. aureus* was grown from a joint aspirate or osteoarticular biopsy sample. Disseminated disease was defined as SAB with at least one other hematogenous metastatic manifestation distant from the primary focus. 30-day all-cause mortality was included as outcome.

### *spa* typing

*spa* typing was performed according to standard protocols. Cycle sequencing products were separated on an ABI310 Genetic Analyser. Sequence data were analyzed and assigned to *spa*-types by use of the Ridom Staph Type (version 2.0.3) software (Ridom Bioinformatics Münster, Germany).

### Microarray Typing

Microarray-based DNA genotyping (StaphType, Alere, Jena, Germany) was performed as previously described [17]. The array covers 334 target sequences corresponding to 185 distinct genes and their allelic variants and detects a wide range of genetic determinants including virulence and resistance genes, genes encoding exotoxins, superantigens, and MSCRAMMs as well as SCCmec, capsule and agr group typing makers [18]. Data interpretation, threshold definition and categorization was performed as described [Monecke FEMS Immunol Med Microbiol [15]. Results categorized as ambiguous were counted as negative. On the basis of hybridisation patterns and by comparing to a database of reference strains isolates were assigned to clonal complexes (CC) using MLST-based nomenclature [15].

### Statistical Methods

All statistical analyses were performed using R version 2.13.1 [R foundation for Statistical Computing, Vienna, Austria, http://www.R-project.org/]. In univariate analyses, Fisheŕs exact test was used for binary variables (including demographic and clinical data, individual genotypes, clonal complex assignments), and a χ^2^-test was used for *agr* types.

In multivariate analyses, a two-step procedure was applied for each endpoint. In the first step, logistic regression models were fitted for each covariate separately including *agr* type, the most prevalent clonal complexes, and the individual multiarray-defined genetic resistance determinants and pathogenicity factors excluding low (<5%) and high (>95%) prevalent genes. For this, *agr* type and clonal complex were taken as binary covariate each. Furthermore, age, study center, sex, methicillin resistance, McCabe-Jackson classification (non-fatal vs. ultimately/rapidly fatal) and mode of acquisition (dummy coded) were included as mandatory covariates. In the second step, covariates with p values≤0.05 were included in a multivariate logistic regression model including the same mandatory covariates as indicated above, allowing to assess the additional value of the covariates of interest beyond readily available clinical variables. For covariates highly correlated with each other (such as individual genes linked in gene clusters), representatives to be included in the analysis were selected by hand.

P values<0.05 were considered to be statistically significant. As this investigation had an explorative character, no adjustment for multiple testing was done.

## Results

### Patient Characteristics

A total of 317 isolates from SAB cohort patients were included in the study. The patient characteristics are summarized in **table 1**. The median age was 65 years, 69% were male, and 63% had an underlying disease classified as non-fatal. Almost half of the SAB episodes were hospital-acquired, 24% were community-onset healthcare-associated, and 27% were community-acquired. In 45% the primary focus was an intravascular catheter/device. An osteoarticular focus was identified (vertebral/non-vertebral osteomyelitis or septic arthritis) in 16%. Other primary foci such as respiratory tract or cardiovascular infection or skin and soft tissue infection were rare (<10% each). In 11% a primary focus could not be identified. The patients of the two centers had similar age, gender ratio, McCabe classification, SAB primary focus distribution, and outcomes. Endstage renal disease was more prevalent in one center (4% vs. 16%, p<0.01).

**Table 1:** Patient characteristics and demographic data of 317 patients with SAB.

Parameter	total
	n = 317
**Male**	220 (69.4%)
**Age (median)**	65 yrs
**Underlying condition**	
Malignancy (hematologic and solid tumor)	92 (29.0%)
Diabetes mellitus	73 (23.0%)
Endstage renal disease	34 (10.7%)
Intravenous drug abuse	13 (4.1%)
HIV-infection	5 (1.6%)
**Severity of illness**	
McCabe non-fatal	200 (63.1%)
McCabe ultimately fatal	106 (33.4%)
McCabe rapidly fatal	11 (3.5%)
**Mode of acquisition**	
Community-acquired	84 (26.5%)
Community-onset healthcare-associated	77 (24.3%)
Hospital-acquired	156 (49.2%)
**MRSA**	31 (9.8%)
**Source of bacteremia/primary focus**	
Unknown	35 (11.0%)
Intravascular catheter/device-related	141 (44.5%)
Osteoarticular infection (osteomyelitis, vertebral osteomyelitis, septicarthritis)	50 (15.8%)
Others	91 (28.7%)
**Infective endocarditis**	35 (11.0%)
**Severe sepsis or septic shock**	102 (32.2%)
**Disseminated disease**	79 (24.9%)
**All-cause mortality at day 30** §	58 (18.4%)
**Late recurrence**	21 (6.6%)

§ Three patients were lost to follow-up.

### Microarray and Spa Typing Results

Forty-nine of the genes detected in the DNA microarray were highly prevalent (≥95% of isolates, 55 genes in ≥90% of isolates) whereas 59 genes were detected in ≤5% of isolates (80 genes in ≤10% of isolates). Thirty-two isolates (10%) were methicillin-resistant (*mecA* positive), 29 of them originated from community-onset healthcare-associated or hospital-acquired SAB. 163 isolates (51%) belonged to *agr* group I, 94 isolates (30%) to *agr* group II, 49 isolates (15%) were *agr* group III and 7 isolates (2%) were *agr* group IV. Four isolates could not be classified. The prevalence of other resistance and major virulence genes is summarized in **table 2**. The complete hybridisation profiles for the individual strains are provided in File S2.

**Table 2:** Prevalence of major virulence factors and resistance determinants in 317 SAB isolates.

Virulence factors	Gene/[Microarray label]	Isolates positive [%]
*egc* gene cluster	*seg, sei, sem, sen, seo, seu*	57.1%
Enterotoxin A	*sea*	35.1%
Enterotoxin B	*seb*	9.1%
Enterotoxin C	*sec*	17.4%
Enterotoxin D/J/R	*sed, sej, ser*	9.1%
Hemolysin beta	*hlb*	62.8%
Leukocidin D/E	*lukD, lukE*	59.0%, 58.0%
Staphylococcal superantigen-like protein-3,-6,-8,-11	[ssl3, ssl6, ssl8, ssl11]	71.0%, 40.4%, 63.7%, 51.7%
Staphylokinase	*sak*	83.0%
Toxic shock syndrome toxin-1	*tst1*	11.0%
**Capsule types, MSCRAMMs and biofilm genes**		
Capsule type 8	[capH8/I8/J8/K8]	59.9%
Capsule type 5	[capH5/I5/J5/K5]	39.7%
Cell wall-associated fibronectin-binding protein	*ebh*	92.7%
Collagen-binding adhesin	*cna*	42.6%
Fibronectin-binding protein B	*fnbB*	81.7%
*S. aureus* surface protein G	*sasG*	49.8%
**Resistance determinants**		
Aminoglycoside adenyltransferase (tobramycin resistance)	*aadD*	6.6%
Beta-lactamase	*blaR, blaZ, (blaI)*	70.7% (71.0%)
Macrolide, lincosamide, streptogramin resistance	*ermC*	6.0%
Metallothiol transferase (fosfomycin resistance)	*fosB*	59.6%
Penicillin binding-protein 2 (methicillin resistance)	*mecA*	10.1%
Tetracycline efflux protein	[tetEfflux]	92.7%
**Others**		
Bone sialoprotein-binding protein	*bbp*	88.3%
Chemotaxis-inhibiting protein (CHIPS)	*chp*	63.4%
Hemolysin gamma A component	*hlgA*	93.1%
Lysylphosphatidylglycerol synthetase	*mprF*	88.3%
Staphylococcal complement inhibitor	*scn*	93.4
Staphylococcal exotoxin-like protein	*setB1*	77.3%
Serine protease A/B/E	*splA, splB, splE*	59.9%, 59.3%, 51.7%

Genes encoding exfoliative toxin serotype A and B (*etA*, *etB*), exfoliative toxin D (*etD*), PVL (*lukF-PV, lukS-PV*) were detected in <5% of isolates. Fusidic acid resistance (*far1*), surface protein involved in biofilm formation (*bap*) genes and capsule type 1 genes (capH1/I1/J1/K1) were not detected.

Genes encoding staphylococcal superantigen-like protein-1,-2,-4,-5,-7,-9,-10 (*ssl*-genes), clumping factor A/B (*clfA/B*), hemolysin alpha (*hla*), aureolysin (*aur*), intercellular adhesion protein A/C (*icaA*, *icaC*) and biofilm PIA synthesis protein D (*icaD*), fibronectin-binding protein A (*fnbA*), fibrinogen binding protein (*fib*) were detected in >95% of isolates. The hemolysin delta gene (*hld*) was found in 100% of isolates.

Based on the microarray genotyping 307 isolates were assigned to 21 different clonal complexes. Seven clonal complexes comprised 74% of all isolates. The most prevalent clonal complex was CC5 (50 isolates, 16%), followed by CC45 (46 isolates, 15%), CC30 (39 isolates, 12%), CC15 (28 isolates, 9%), CC7 (27 isolates, 9%), CC8 (24 isolates, 8%) and CC22 (22 isolates, 7%) (table 2).

*spa* typing revealed 157 different *spa* types among 316 typeable isolates and showed a substantial biodiversity within each of the clonal lineages and no overlap between microarray-derived clonal lineages (File S3). Only seven *spa* types were detected 10 times or more often, and 121 isolates represented single *spa* types.

### Association between Microarray Genotypes and Mortality and Disease Severity

Univariate analyses were performed to evaluate a potential association of the seven most prevalent clonal lineages or *agr* types with different clinical outcomes. CC 15 was found to be associated with a higher mortality at day 30 (36% vs. 17%, p = 0.017), a tendency for more frequent septic shock or severe sepsis/septic shock (not significant), and a lower prevalence of disseminated disease (7% vs. 27%, p = 0.022) compared to non-CC15 isolates. Next, a univariate analysis of possible associations of 185 prevalent virulence and resistance genes was performed. An association with day-30 mortality (p<0.05) was found for *mecA*, *bla*, *ermA*, *ermC*, *aadD*, *sed/sej/ser*, and *ssl11*. Genes identified to be associated with severe sepsis or septic shock in univariate analysis were *aadD*, *fosB*, *sed/sej/ser*. The genes *lukE*, *sak*, *chp*, *fib* were found to be associated with disseminated disease.

### Association between Microarray Genotypes and Infection Site (Endovascular or Osteoarticular)

Univariate analyses revealed an association between CC22 versus non-CC22 isolates and osteoarticular infection (36% vs. 14%, p = 0.015) but no other significant associations between clonal complex or *agr* type and infection site as investigated here. Individual genes associated with osteoarticular infections were *egc* gene cluster, *lukD*, *splA/B*, *ssl*3, *ssl*8, *cna*, *ebh*. There was no significant association in univariate analysis between individual genes and gene clusters with endocarditis. Complete data of univariate analyses are provided in File S4.

### Association of Genotypes versus Host Factors with Clinical Outcomes

The following determinants were included in multivariate regression analyses: *S. aureus* clonal complex (7 most prevalent clonal complexes), *agr* type, microarray-defined virulence and resistance genes or gene clusters, mode of acquisition of SAB, study center, and age, sex and McCabe classification as host factors (**table 3**).

**Table 3:** Multivariate logistic regression analyses for endpoints 30-day mortality, severe sepsis or septic shock and disseminated disease.

	30-day all-cause mortality (58 vs. 256 patients)§		Severe sepsis or septic shock (102 vs. 215 patients)		Disseminated disease (79 vs. 238 patients)	
Parameter/Risk factor	OR (95% CI)	p value	OR (95% CI)	p value	OR (95% CI)	p value
Intercept	–	<0.01	–	<0.01	–	<0.01
**Age [per year]**&	1.06 (1.03–1.10)	<0.01	1.02 (1.00–1.04)	0.02	1.0 (0.98–1.02)	0.99
**Study center B**&	1.81 (0.87–3.77)	0.11	0.91 (0.53–1.54)	0.72	0.47 (0.26–0.87)	0.02
**Male Sex**&	1.38 (0.64–2.96)	0.42	1.66 (0.93–2.94)	0.08	0.95 (0.52–1.76)	0.88
**McCabe ultimately or rapidly fatal**&	4.52 (2.09–9.79)	<0.01	1.38 (0.79–2.41)	0.26	0.90 (0.48–1.71)	0.75
**Mode of acquisition**&						
Community-acquired SAB	3.40 (1.31–8.81)	0.01	4.82 (2.50–9.50)	<0.01	5.21 (2.55–10.63)	<0.01
Community-onset healthcare-associated SAB	3.68 (1.58–8.54)	<0.01	2.88 (1.52–5.48)	<0.01	2.70 (1.31–5.57)	0.01
**Methicillin resistance (** ***mecA*** **) ^&^** ‡	4.80 (1.43–16.06)	0.01	1.21 (0.46–3.16)	0.70	0.83 (0.26–2.62)	0.75
**Beta-lactamase (** ***blaZ/R*** **)** #	3.12 (1.17–8.30)	0.02				
**Macrolide, lincosamide, streptogramin resistance (** ***ermC*** **)**	4.64 (1.32–16.35)	0.02				
**Fosfomycin resistance (** ***fosB*** **)**			1.71 (0.98–2.99)	0.06		
**Enterotoxin D/J/R (** ***sed/sej/ser*** **)** § †			2.27 (0.84–6.15)	0.11	1.87 (0.64–5.47)	0.25
**Enterotoxin A (** ***sea*** **)**					1.01 (0.51–2.01)	0.97
**Leukocidin E (** ***lukE*** **)**					1.57 (0.66–3.73)	0.31
**Staphylokinase (** ***sak*** **)**					1.55 (0.44–5.48)	0.49
**Chemotaxis-inhibiting protein (** ***chp*** **)**					0.85 (0.40–1.83)	0.68
**Hemolysin beta (** ***hlb*** **)**					1.37 (0.53–3.56)	0.52
**CC15** ‡	2.66 (0.96–7.40)	0.06			0.35 (0.04–2.84)	0.33
**CC5**	0.38 (0.11–1.36)	0.14				

§ Three patients were lost to follow-up.

&Mandatory variable.

# Due to an extremely high correlation of *blaI* and *blaR* (contingency coefficient 1.0 of *blaR* with *blaZ*), only *blaZ* was included in the multivariate logistic regression model (endpoint 30-day mortality).

§ Due to a high correlation of *sed/sej/ser* and *aadD* (contingency coefficient 0.71), only *sed/sej/ser* was included in the multivariate logistic regression model (endpoint severe sepsis/septic shock). If *aadD* is included instead, results are OR 4.23 (95% CI 0.91–19.61, p = 0.07) for *aadD* and OR 1.80 (95% CI 1.04–3.10, p = 0.03) for *fosB*.

† Due to a contingency coefficient of 1.0 of *sed*, *sej*, *ser*, only *sed* was included in the multivariable logistic regression model (endpoint disseminated disease).

‡ If 22 SAB cases with initiation of adequate antibiotic therapy ≥3 days after onset of SAB were excluded from the analysis CC15 exhibited a marginally significant effect with OR 2.81 (95% CI 1.0–7.91, P = 0.05) whereas mecA lost its significant impact (OR 3.35, 95% CI 0.79–14.21, p = 0.10). For the other endpoints no significant changes were observed.

#### 30-day all-cause mortality

age (OR 1.06, 95% CI 1.03–1.1), McCabe classification (OR 4.5, 95% CI 2.1–9.8) and mode of acquisition (OR 3.4, 95% CI 1.3–8.8, for community-acquired SAB and OR 3.7, 95% CI 1.6–8.5, for community-onset healthcare-associated SAB) were factors found to significantly contribute to day-30 mortality, in addition to the pathogen factors *mecA* (OR 4.8, 95% CI 1.4–16.1), *bla* encoding for beta-lactamase (OR 3.1, 95% CI 1.2–8.3) and *ermC* encoding macrolide, lincosamide and streptogramin resistance (OR 4.6, 95% CI 1.3–16.4). A trend towards a higher day-30 mortality was noted for CC15 isolates (OR 2.7, 95% CI 0.96–7.4), whereas CC5 tended to be associated with lower day-30 mortality (OR 0.4, 95% CI 0.1–1.4).

#### Disease severity

The development of severe sepsis/septic shock was associated with age (OR 1.02, 95% CI 1.0–1.04) and mode of acquisition (OR 5.0, 95% CI 2.6–9.6, for community-acquired SAB, and OR 3.0, 95% CI 1.6–5.7, for community-onset healthcare-associated SAB).

#### Disseminated disease

Community acquisition (OR 5.2, 95% CI 2.6–10.6) and community-onset healthcare-associated SAB (OR 2.7, 95% CI 1.3–5.6), and study center (OR 0.5, 95% CI 0.3–0.9) but none of the pathogen factors were found to be associated with disseminated disease.

#### Infective endocarditis

Mode of acquisition (OR 7.1, 95% CI 2.5–19.7, for community-acquired SAB and OR 3.6, 95% CI 1.2–10.7, for community-onset healthcare-associated SAB) was also associated with endocarditis (**table 4**). Independent pathogen factors identified here were the genes *sed, sej, ser* (located on the same mobile genetic element) coding for enterotoxin D, J and R (OR 5.1, 95% CI 1.4–18.6) which had not been identified by univariate analysis.

**Table 4:** Multivariate logistic regression analysis for endpoint infective endocarditis.

	Infective endocarditis (35 vs. 282 patients)	
Parameter/Risk factor	OR (95% CI)	p value
Intercept	–	p<0.01
**Age [per year]** &	0.99 (0.97–1.01)	0.55
**Study center B** &	0.77 (0.36–1.67)	0.51
**Male Sex** &	1.20 (0.53–2.72)	0.66
**McCabe ultimately or rapidly fatal &**	0.77 (0.32–1.88)	0.57
**Mode of acquisition** &		
Community-acquired SAB	7.08 (2.54–19.74)	<0.01
Community-onset healthcare-associated SAB	3.62 (1.22–10.68)	0.02
**Methicillin resistance (** ***mecA*** **)** &	0.45 (0.09–2.33)	0.32
**Enterotoxin D/J/R (** ***sed/sej/ser*** **)** #	5.11 (1.4–18.62)	0.01

OR Odds ratio. CI Confidence interval. & Mandatory variable.

# Due to a contingency coefficient of 1.0 of *sed*, *sej*, *ser*, only *sed* was included in the multivariable logistic regression model.

#### Osteoarticular infection

Osteoarticular infection was associated with 24 covariates at a p-value<0.05. Due to this large number and high intercorrelations, a multivariate regression model could not be built. In the basic model with methicillin resistance, age, study center, sex, comorbidity and mode of acquisition, only the latter showed a significant effect (community-acquired SAB, OR 16.4, 95% CI 5.8–46.2; and community-onset healthcare-associated SAB, OR 7.9, 95% CI 2.7–22.7). **Table 5** lists all covariates with a p value<0.05 when included separately in this basic model (step 1 of the above described procedure). CC22 (OR 4.6, 95% CI 1.5–14.1), presence of enterotoxins G/I/M/N/O/U (*egc* gene cluster, OR 2.4, 95% CI 1.2–5.0) and collagen-binding adhesin (*cna*, OR 2.6, 95% CI 1.3–5.2) were factors associated with osteoarticular infections, whereas a range of other factors was negatively associated with osteoarticular infections, notably *splA* and *splB* encoding serine proteases A and B, *ssl3* and *ssl8* encoding superantigen-like proteins, and cell wall-associated fibronectin-binding protein (*ebh*) among others (**table 5**).

**Table 5:** Logistic regression analysis for endpoint osteoarticular infection adjusted for age, study center, sex, comorbidity, mode of acquisition and methicillin resistance.

	Osteoarticular infection (50 vs. 267 patients)	
Parameter/Risk factor	OR (95% CI)	adjusted p value
**Cell wall-associated fibronectin-binding protein (** ***ebh*** **)**	0.26 (0.09–0.75)	0.01
**Collagen-binding adhesin (** ***cna*** **)**	2.59 (1.30–5.16)	0.01
**Enterotoxin G/I/M/N/O/U (egc gene cluster)**	2.43 (1.18–4.99)	0.02
**Leukocidin D (** ***lukD*** **)**	0.37 (0.19–0.73)	<0.01
**Leukocidin E (** ***lukE*** **)**	0.45 (0.23–0.89)	0.02
**Lysylphosphatidylglycerol synthetase (** ***mprF*** **)**	0.40 (0.16–0.99)	0.05
**Putative hemolysin membrane protein (** ***hlIII*** **)**	0.26 (0.09–0.75)	0.01
**Serine protease A (** ***splA*** **)**	0.40 (0.20–0.79)	0.01
**Serine protease B (** ***splB*** **)**	0.41 (0.21–0.80)	0.01
**Staphylococcal exotoxin-like protein (** ***setB1*** **)**	0.37 (0.18–0.76)	0.01
**Staphylococcal exotoxin-like protein (** ***setC*** **)**	0.45 (0.21–0.94)	0.03
**Staphylococcal superantigen-like protein 3 (** ***ssl3*** **)**	0.38 (0.19–0.76)	0.01
**Staphylococcal superantigen-like protein 8 (** ***ssl8*** **)**	0.42 (0.21–0.82)	0.01
**Tetracycline efflux protein**	0.26 (0.09–0.75)	0.01
**Type 1 site specific deoxyribonuclease subunit (** ***hsd2*** **)**	4.27 (1.56–11.68)	<0.01
**CC22**	4.61 (1.51–14.10)	0.01

## Discussion

Studies investigating associations between genetic traits of *S. aureus* and clinical manifestations and outcomes are challenging and have yielded conflicting results for several reasons. First, *S. aureus* isolates within a clonal lineage possess a consensus repertoire of virulence genes with limited variation due to mobile genetic elements. Thus, studies on the association of virulence genes with invasiveness or other endpoints can be biased by an uneven distribution of clonal complexes between the two groups investigated (hitchhiker effect) [6], [7], [13]. Second, grouping of invasive isolates from a variety of infections such as skin infection, pneumonia, joint infection and bacteremia may have hampered some studies [4]–[7], [9], [10], [19]. Finally, host factors such as underlying disease [20] and age [21], have a major impact on clinical manifestation and outcome of *S. aureus* disease and need to be considered as possible confounders. The present study aimed to account for these issues and found few associations between clonal complex or specific pathogen genes and outcome.

Similar to other studies from other areas (UK, Netherlands, USA) we found isolates of CC5, CC45, CC30, CC15 and CC8 to be among the most prevalent to cause invasive disease [4], [22], [23]. Several groups have linked CC30 isolates to more severe disease, i.e. hematogenous complications [22], infective endocarditis [24], persistent bacteremia [12], or invasive disease per se [4], [10]. In contrast to these studies we did not observe a greater pathogenic potential of CC30 isolates.

In addition, there was no evidence in our cohort of an association of CC5 isolates with disseminated disease as previously described in an investigation that compared carriage and uncomplicated infections [22]. Interestingly, in our study CC5 was associated with lower 30-day all-cause mortality while for CC15, there was a tendency for higher mortality, independent of antimicrobial resistance determinants.

The most significant finding regarding clonal lineages was the association between CC22 and osteoarticular infection. However, it proved difficult to confirm this to be an independent association since a meaningful multivariate analysis could not be performed due to too many intercorrelating factors. An association between CC22 and osteoarticular infection has not been described previously [8], [25]. Also, *cna* as a potential factor for osteoarticular infection has not been identified in earlier studies.

Several of the genes found associated with selected clinical endpoints in this study (e.g. *cna*, *egc* gene cluster, *sea*) have been described by others to be associated with invasive, disseminated or severe disease [9], [13], [26]. Some of them remained significant in the current study if tested in a multivariate analysis that included further genetic determinants and patient factors. Antimicrobial resistance determinants rather than virulence factors were linked to poor survival which in the case of *mecA* corroborates previous findings with phenotypic methicillin resistance [16], [26] but which has not been described so far for *bla* (prevalence 71%) and *ermC* (prevalence 6%). The significance of these two associations remains unclear. In line with findings of a French study we could not determine any of the *S. aureus* superantigens to be associated with development of severe sepsis or septic shock [19].

In a recent multinational cohort study that compared infective endocarditis (IE) with SSTI isolates, IE isolates were more likely to be positive for adhesin genes *sdrC*, *cna*, *map/eap* and for genes *tst*, *sea*, *sed*, *see* and *sei* that code for toxins [24]. Using a different study design by comparing SAB isolates with vs. without IE we also found the presence of the enterotoxin gene *sed* (accompanied by *sej* and *ser*) to be independently associated with IE. As a plausible pathophysiological hypothesis is not imminent so far, a linkage of *sed* to yet unrecognized virulence genes deserves further consideration [24].

The relative paucity of individual pathogen factors that were associated with the investigated endpoints is contrasted by the fact that several host factors like age, comorbidity and mode of acquisition substantially impacted on SAB outcome and manifestation. The identification of mode of acquisition (particularly community-acquisition) as strong independent factor for each of the five endpoints is consistent with results of several previous studies [27], [28]. The observed influence may partly be a consequence of a longer “incubation period” with the longer presence of *S. aureus* in the bloodstream leading to different infective foci, such as endocarditis. Apart from host and pathogen factors, clinical management was shown to impact SAB outcome [16]. In our study, time from initial symptoms to initiation of adequate antimicrobial therapy could not be included as an additional variable in the multivariate model, as it was highly correlated with community-acquisition of SAB. Excluding the 22 cases with initiation of adequate antibiotic therapy of ≥3 days after SAB onset revealed no changes of factors contributing to the endpoints endocarditis, severe sepsis/septic shock, disseminated disease and osteoarticular infections and only very minor changes to the endpoint 30-day all-cause mortality.

Several conclusions may be drawn from our investigation. Studies on associations of genetic determinants with *S. aureus* manifestations and outcome need to be adjusted for host characteristics. In fact, our study revealed that most of the genetic determinants identified by univariate analysis were no longer significant after adjustment for host characteristics. Notably, age and mode of acquisition exhibited the strongest impact on clinical outcomes. In view of the present and previous reports [4], [7], [14] it appears that single virulence factors as currently determined by DNA microarray tests cannot sufficiently explain the invasive and pathogenic potential of *S. aureus* nor the clinical phenotype of *S. aureus* infections. Unknown genes [5], gene expression, and gene regulation may be more relevant for SAB outcome [3], [29].

There are limitations of the present study. As our investigation had an exploratory character, our results have to be validated in other cohorts. Only genes present on the microarray were investigated, yet other genes may be of major importance for the outcome of SAB. The study was conducted at only two tertiary care centers in a single country. Although the distribution of clonal complexes was similar to that described in a recent multinational European study of invasive *S. aureus* infections [30], our results can only be generalized after confirmation in other large SAB cohorts in geographically distant regions. Though one of the largest studies in this field, a larger sample might be needed to be able to demonstrate relatively modest effects (odds ratio of <3) of bacterial genotypes, therefore collaborative cohorts or multinational consortia may be of importance to address these issues in future.

In summary, we found only a limited number of microbial genetic determinants to be independently associated with the investigated clinical endpoints of SAB. Conversely, host factors such as age, comorbidity and community-acquisition of SAB revealed a substantial independent impact on the course and outcome of SAB and need to be accounted for as effect modifiers in genotypic association studies. Clinical awareness for host factors that predict a poor SAB outcome is warranted. Finally, the study indicates the need to intensify research on immunological host factors underlying intermittent and persistent *S. aureus* colonization and the susceptibility to severe, invasive and systemic *S. aureus* infections.

## Supporting Information

File S1 Patient characteristics and demographic data of all SAB patients within the INvasive STapylococcus aureus INfections CohorT (INSTINCT) study and the subgroup of SAB cases included within this study.
(PDF)

File S2 Complete hybridisation results for *S*. *aureus* strains examined in this study. Data are ordered by the assigned clonal complex and the strain designation.
(PDF)

File S3 Distribution of clonal complexes and *spa* types in 317 patients with SAB.
(PDF)

File S4 Complete data of univariate analyses of pathogen factors (clonal complex, *agr* type and microarray-derived virulence and resistance genes) associated with investigated clinical endpoints.
(PDF)
