# Supplementary material for: Microarray-Based Genotyping and Clinical Outcomes of Staphylococcus aureus Bloodstream Infection: An Exploratory Study
Source: PLoS One. 2013 Aug 14;8(8):e71259. doi: 10.1371/journal.pone.0071259 (PMC3743874; doi:10.1371/journal.pone.0071259)
Supplement: File S1 — Patient characteristics and demographic data of all SAB patients within the INvasive STapylococcus aureus INfections CohorT (INSTINCT) study and the subgroup of SAB cases included within this study. (PDF) [file pone.0071259.s001.pdf]

Supplemental file S1

Patient characteristics and demographic data of all SAB patients within the INvasive STapylococcus aureus INfections CohorT (INSTINCT) and the subgroup of SAB cases included in this study.

Parameter	All SAB cases within INSTINCT n=779	SAB cases incuded n=317
**Male**	519 (66.6%)	220 (69.4%)
**Age (median)**	66 yrs	65 yrs
**Underlying condition**		
Malignancy (hematologic and solid tumor)	244 (31.3%)	92 (29.0%)
Diabetes mellitus	177 (22.7%)	73 (23.0%)
Endstage renal disease	81 (10.4%)	34 (10.7%)
Intravenous drug abuse	27 (3.5%)	13 (4.1%)
HIV-infection	10 (1.3%)	5 (1.6%)
**Severity of illness**		
McCabe non-fatal	451 (57.9%)	200 (63.1%)
McCabe ultimately fatal	288 (37.0%)	106 (33.4%)
McCabe rapidly fatal	40 (5.1%)	11 (3.5%)
**Mode of acquisition**		
Community-acquired	125 (16.0%)	84 (26.5%)
Community-onset healthcare-associated	246 (31.6%)	77 (24.3%)
Hospital-acquired	408 (52.4%)	156 (49.2%)
**MRSA**	81 (10.4%)	31 (9.8%)
**Source of bacteremia/primary focus**		
Unknown	165 (21.2)	35 (11.0%)
Intravascular catheter/device-related	254 (32.6%)	141 (44.5%)
Osteoarticular infection (osteomyelitis, vertebral osteomyelitis, septic arthritis)	98 (12.6%)	50 (15.8%)
Others	262 (33.6%)	91 (28.7%)
**Infective endocarditis**	81 (10.4%)	35 (11.0%)
**Severe sepsis or septic shock**	124+104 (29.3%)	102 (32.2%)
**Disseminated disease**	181 (23.2%)	79 (24.9%)
**All-cause mortality at day 30 §**	142 (18.2%)	58 (18.4%)
**Late recurrence**	52 (6.7%)	21 (6.6%)

Total numbers of SAB cases within the study period were 805, of which 779 (97%) were evaluated within the INSTINCT study (419/422 in study center A, 360/383 in study center B). 293 isolates were available in the study center A, of which every second isolate (147 isolates) was included in the study. 340 isolates were available in study center B, of which 170 isolates were included in the study.

§ Three patients were lost to follow-up.
