# Supplementary material for: Microarray-Based Genotyping and Clinical Outcomes of Staphylococcus aureus Bloodstream Infection: An Exploratory Study
Source: PLoS One. 2013 Aug 14;8(8):e71259. doi: 10.1371/journal.pone.0071259 (PMC3743874; doi:10.1371/journal.pone.0071259)
Supplement: File S2 — Complete hybridisation results for S. aureus strains examined in this study. Data are ordered by the assigned clonal complex and the strain designation. (PDF) [file pone.0071259.s002.pdf]

				SPECIES MARKERS							REGULATORY GENES						
Strain	Clonal complex	*agr* group	spa-type	Ribos STAU	gapA	katA	CoA	proteinA	sbi	nuc	agrI (total)	agrII (total)	agrIII (total)	agrIV (total)	sarA	saeS	vraS
MSSA1805	CC1	III	t273	POS	POS	POS	POS	POS	POS	POS	NEG	NEG	POS	NEG	POS	POS	POS
MSSA1823	CC1	III	t321	POS	POS	POS	POS	POS	POS	POS	NEG	NEG	POS	NEG	POS	POS	POS
MSSA1837	CC1	III	t127	POS	POS	POS	POS	POS	POS	POS	NEG	NEG	POS	NEG	POS	POS	POS
MSSA2044	CC1	III	t1784	POS	POS	POS	POS	POS	POS	POS	NEG	NEG	POS	NEG	POS	POS	POS
MSSA2046	CC1	III	t127	POS	POS	POS	POS	POS	POS	POS	NEG	NEG	POS	NEG	POS	POS	POS
MSSA2059	CC1	III	t127	POS	POS	POS	POS	POS	POS	POS	NEG	NEG	POS	NEG	POS	POS	POS
MSSA2076	CC1	III	t127	POS	POS	POS	POS	POS	POS	POS	NEG	NEG	POS	NEG	POS	POS	POS
MSSA2200	CC1	III	t127	POS	POS	POS	POS	POS	POS	POS	NEG	NEG	POS	NEG	POS	POS	POS
MRSA3797	CC5	II	t003	POS	POS	POS	POS	POS	POS	POS	NEG	POS	NEG	NEG	POS	POS	POS
MRSA3798	CC5	II	t003	POS	POS	POS	POS	POS	POS	POS	NEG	POS	NEG	NEG	POS	POS	POS
MRSA3799	CC5	II	t003	POS	POS	POS	POS	POS	POS	POS	NEG	POS	NEG	NEG	POS	POS	POS
MRSA3800	CC5	II	t003	POS	POS	POS	POS	POS	POS	POS	NEG	POS	NEG	NEG	POS	POS	POS
MRSA3801	CC5	II	t003	POS	POS	POS	POS	POS	POS	POS	NEG	POS	NEG	NEG	POS	POS	POS
MRSA3804	CC5	II	t003	POS	POS	POS	POS	POS	POS	POS	NEG	POS	NEG	NEG	POS	POS	POS
MRSA3806	CC5	II	t003	POS	POS	POS	POS	POS	POS	POS	NEG	POS	NEG	NEG	POS	POS	POS
MRSA3961	CC5	II	t003	POS	POS	POS	POS	POS	POS	POS	NEG	POS	NEG	NEG	POS	POS	POS
MRSA3962	CC5	II	t002	POS	POS	POS	POS	POS	POS	POS	NEG	POS	NEG	NEG	POS	POS	POS
MRSA3963	CC5	II	t002	POS	POS	POS	POS	POS	POS	POS	NEG	POS	NEG	NEG	POS	POS	POS
MRSA3966	CC5	II	t002	POS	POS	POS	POS	POS	POS	POS	NEG	POS	NEG	NEG	POS	POS	POS
MRSA3978	CC5	II	t003	POS	POS	POS	POS	POS	POS	POS	NEG	POS	NEG	NEG	POS	POS	POS
MRSA3979	CC5	II	t003	POS	POS	POS	POS	POS	POS	POS	NEG	POS	NEG	NEG	POS	POS	POS
MRSA3980	CC5	II	t003	POS	POS	POS	POS	POS	POS	POS	NEG	POS	NEG	NEG	POS	POS	POS
MRSA4179	CC5	II	t045	POS	POS	POS	POS	POS	POS	POS	NEG	POS	NEG	NEG	POS	POS	POS
MRSA4216	CC5	II	t003	POS	POS	POS	POS	POS	POS	POS	NEG	POS	NEG	NEG	POS	POS	POS
MRSA4217	CC5	II	t003	POS	POS	POS	POS	POS	POS	POS	NEG	POS	NEG	NEG	POS	POS	POS
MRSA4220	CC5	II	t003	POS	POS	POS	POS	POS	POS	POS	NEG	POS	NEG	NEG	POS	POS	POS
MRSA4221	CC5	II	t003	POS	POS	POS	POS	POS	POS	POS	NEG	POS	NEG	NEG	POS	POS	POS
MRSA4222	CC5	II	t003	POS	POS	POS	POS	POS	POS	POS	NEG	POS	NEG	NEG	POS	POS	POS
MSSA1751	CC5	II	t2249	POS	POS	POS	POS	POS	POS	POS	NEG	POS	NEG	NEG	POS	POS	POS
MSSA1760	CC5	II	t002	POS	POS	POS	POS	POS	POS	POS	NEG	POS	NEG	NEG	POS	POS	POS
MSSA1802	CC5	II	t002	POS	POS	POS	POS	POS	POS	POS	NEG	POS	NEG	NEG	POS	POS	POS
MSSA1809	CC5	II	t010	POS	POS	POS	POS	POS	POS	POS	NEG	POS	NEG	NEG	POS	POS	POS
MSSA1815	CC5	II	t6396	POS	POS	POS	POS	POS	POS	POS	NEG	POS	NEG	NEG	POS	POS	POS
MSSA1829	CC5	II	t586	POS	POS	POS	POS	POS	POS	POS	NEG	POS	NEG	NEG	POS	POS	POS
MSSA1908	CC5	II	t062	POS	POS	POS	POS	POS	POS	POS	NEG	POS	NEG	NEG	POS	POS	POS
MSSA1960	CC5	II	t003	POS	POS	POS	POS	POS	POS	POS	NEG	POS	NEG	NEG	POS	POS	POS
MSSA1965	CC5	II	t002	POS	POS	POS	POS	POS	POS	POS	NEG	POS	NEG	NEG	POS	POS	POS
MSSA1977	CC5	II	t002	POS	POS	POS	POS	POS	POS	POS	NEG	POS	NEG	NEG	POS	POS	POS
MSSA1978	CC5	II	t3012	POS	POS	POS	POS	POS	POS	POS	NEG	POS	NEG	NEG	POS	POS	POS
MSSA1999	CC5	II	t688	POS	POS	POS	POS	POS	POS	POS	NEG	POS	NEG	NEG	POS	POS	POS
MSSA2003	CC5	II	t002	POS	POS	POS	POS	POS	POS	POS	NEG	POS	NEG	NEG	POS	POS	POS
MSSA2006	CC5	II	t045	POS	POS	POS	POS	POS	POS	POS	NEG	POS	NEG	NEG	POS	POS	POS
MSSA2008	CC5	II	t7893	POS	POS	POS	POS	POS	POS	POS	NEG	POS	NEG	NEG	POS	POS	POS
MSSA2011	CC5	II	t003	POS	POS	POS	POS	POS	POS	POS	NEG	POS	NEG	NEG	POS	POS	POS
MSSA2017	CC5	II	t548	POS	POS	POS	POS	POS	POS	POS	NEG	POS	NEG	NEG	POS	POS	POS
MSSA2022	CC5	II	t627	POS	POS	POS	POS	POS	POS	POS	NEG	POS	NEG	NEG	POS	POS	POS
MSSA2055	CC5	II	t002	POS	POS	POS	POS	POS	POS	POS	NEG	POS	NEG	NEG	POS	POS	POS
MSSA2060	CC5	II	t003	POS	POS	POS	POS	POS	POS	POS	NEG	POS	NEG	NEG	POS	POS	POS
MSSA2080	CC5	II	t002	POS	POS	POS	POS	POS	POS	POS	NEG	POS	NEG	NEG	POS	POS	POS
MSSA2082	CC5	II	t002	POS	POS	POS	POS	POS	POS	POS	NEG	POS	NEG	NEG	POS	POS	POS
MSSA2084	CC5	II	t311	POS	POS	POS	POS	POS	POS	POS	NEG	POS	NEG	NEG	POS	POS	POS
MSSA2087	CC5	II	t3524	POS	POS	POS	POS	POS	POS	POS	NEG	POS	NEG	NEG	POS	POS	POS
MSSA2094	CC5	II	t003	POS	POS	POS	POS	POS	POS	POS	NEG	POS	NEG	NEG	POS	POS	POS
MSSA2097	CC5	II	t002	POS	POS	POS	POS	POS	POS	POS	NEG	POS	NEG	NEG	POS	POS	POS
MSSA2110	CC5	II	t002	POS	POS	POS	POS	POS	POS	POS	NEG	POS	NEG	NEG	POS	POS	POS
MSSA2113	CC5	II	t062	POS	POS	POS	POS	POS	POS	POS	NEG	POS	NEG	NEG	POS	POS	POS
MSSA2132	CC5	II	t2066	POS	POS	POS	POS	POS	POS	POS	NEG	POS	NEG	NEG	POS	POS	POS
MSSA2134	CC5	II	t509	POS	POS	POS	POS	POS	POS	POS	NEG	POS	NEG	NEG	POS	POS	POS
MSSA1746	CC7	I	t091	POS	POS	POS	POS	POS	POS	POS	POS	NEG	NEG	NEG	POS	POS	POS
MSSA1757	CC7	I	t091	POS	POS	POS	POS	POS	POS	POS	POS	NEG	NEG	AMB	POS	POS	POS
MSSA1772	CC7	I	t091	POS	POS	POS	POS	POS	POS	POS	POS	NEG	NEG	NEG	POS	POS	POS
MSSA1795	CC7	I	t091	POS	POS	POS	POS	POS	POS	POS	POS	NEG	NEG	AMB	POS	POS	POS
MSSA1813	CC7	I	t091	POS	POS	POS	POS	POS	POS	POS	POS	NEG	NEG	NEG	POS	POS	POS
MSSA1826	CC7	I	t091	POS	POS	POS	POS	POS	POS	POS	POS	NEG	NEG	NEG	POS	POS	POS
MSSA1830	CC7	I	t091	POS	POS	POS	POS	POS	POS	POS	POS	NEG	NEG	NEG	POS	POS	POS
MSSA1838	CC7	I	t091	POS	POS	POS	POS	POS	POS	POS	POS	NEG	NEG	NEG	POS	POS	POS
MSSA1944	CC7	I	t091	POS	POS	POS	POS	POS	POS	POS	POS	NEG	NEG	NEG	POS	POS	POS
MSSA1947	CC7	I	t091	POS	POS	POS	POS	POS	POS	POS	POS	NEG	NEG	NEG	POS	POS	POS
MSSA1964	CC7	I	t091	POS	POS	POS	POS	POS	POS	POS	POS	NEG	NEG	NEG	POS	POS	POS
MSSA1969	CC7	I	t091	POS	POS	POS	POS	POS	POS	POS	POS	NEG	NEG	NEG	POS	POS	POS
MSSA2001	CC7	I	t1943	POS	POS	POS	POS	POS	POS	POS	POS	NEG	NEG	NEG	POS	POS	POS
MSSA2004	CC7	I	t1119	POS	POS	POS	POS	POS	POS	POS	POS	NEG	NEG	NEG	POS	POS	POS
MSSA2024	CC7	I	t091	POS	POS	POS	POS	POS	POS	POS	POS	NEG	NEG	NEG	POS	POS	POS
MSSA2025	CC7	I	t091	POS	POS	POS	POS	POS	POS	POS	POS	NEG	NEG	NEG	POS	POS	POS
MSSA2026	CC7	I	t091	POS	POS	POS	POS	POS	POS	POS	POS	NEG	NEG	NEG	POS	POS	POS
MSSA2032	CC7	I	t091	POS	POS	POS	POS	POS	POS	POS	POS	NEG	NEG	NEG	POS	POS	POS
MSSA2043	CC7	I	t1943	POS	POS	POS	POS	POS	POS	POS	POS	NEG	NEG	NEG	POS	POS	POS
MSSA2061	CC7	I	t091	POS	POS	POS	POS	POS	POS	POS	POS	NEG	NEG	NEG	POS	POS	POS
MSSA2085	CC7	I	t091	POS	POS	POS	POS	POS	POS	POS	POS	NEG	NEG	NEG	POS	POS	POS
MSSA2098	CC7	I	t091	POS	POS	POS	POS	POS	POS	POS	POS	NEG	NEG	AMB	POS	POS	POS
MSSA2100	CC7	I	t091	POS	POS	POS	POS	POS	POS	POS	POS	NEG	NEG	NEG	POS	POS	POS
MSSA2111	CC7	I	t867	POS	POS	POS	POS	POS	POS	POS	POS	NEG	NEG	AMB	POS	POS	POS
MSSA2178	CC7	I	t091	POS	POS	POS	POS	POS	POS	POS	POS	NEG	NEG	NEG	POS	POS	POS
MSSA2202	CC7	I	t091	POS	POS	POS	POS	POS	POS	POS	POS	NEG	NEG	NEG	POS	POS	POS
MSSA2205	CC7	I	t091	POS	POS	POS	POS	POS	POS	POS	POS	NEG	NEG	NEG	POS	POS	POS
MRSA3796	CC8	I	t068	POS	POS	POS	POS	POS	POS	POS	POS	NEG	NEG	AMB	POS	POS	POS
MRSA3805	CC8	I	t030	POS	POS	POS	POS	POS	POS	POS	POS	NEG	NEG	AMB	POS	POS	POS
MRSA3965	CC8	I	t068	POS	POS	POS	POS	POS	POS	POS	POS	NEG	NEG	NEG	POS	POS	POS
MSSA1761	CC8	I	t304	POS	POS	POS	POS	POS	POS	POS	POS	NEG	NEG	AMB	POS	POS	POS
MSSA1764	CC8	I	t008	POS	POS	POS	POS	POS	POS	POS	POS	NEG	NEG	NEG	POS	POS	POS
MSSA1765	CC8	I	t024	POS	POS	POS	POS	POS	POS	POS	POS	NEG	NEG	NEG	POS	POS	POS
MSSA1784	CC8	I	t118	POS	POS	POS	POS	POS	POS	POS	POS	NEG	NEG	AMB	POS	POS	POS
MSSA1806	CC8	I	t6948	POS	POS	POS	POS	POS	POS	POS	POS	NEG	NEG	NEG	POS	POS	POS
MSSA1807	CC8	I	t967	POS	POS	POS	POS	POS	POS	POS	POS	NEG	NEG	NEG	POS	POS	POS
MSSA1888	CC8	I	t6395	POS	POS	POS	POS	POS	POS	POS	POS	NEG	NEG	NEG	POS	POS	POS
MSSA1894	CC8	I	t008	POS	POS	POS	POS	POS	POS	POS	POS	NEG	NEG	NEG	POS	POS	POS
MSSA1922	CC8	I	t008	POS	POS	POS	POS	POS	POS	POS	POS	NEG	NEG	NEG	POS	POS	POS
MSSA1950	CC8	I	t008	POS	POS	POS	POS	POS	POS	POS	POS	NEG	NEG	NEG	POS	POS	POS
MSSA1954	CC8	I	t2293	POS	POS	POS	POS	POS	POS	POS	POS	NEG	NEG	NEG	POS	POS	POS
MSSA1966	CC8	I	t6395	POS	POS	POS	POS	POS	POS	POS	POS	NEG	NEG	NEG	POS	POS	POS
MSSA1972	CC8	I	t008	POS	POS	POS	POS	POS	POS	POS	POS	NEG	NEG	NEG	POS	POS	POS
MSSA2010	CC8	I	t008	POS	POS	POS	POS	POS	POS	POS	POS	NEG	NEG	NEG	POS	POS	POS
MSSA2020	CC8	I	t008	POS	POS	POS	POS	POS	POS	POS	POS	NEG	NEG	NEG	POS	POS	POS
MSSA2028	CC8	I	t024	POS	POS	POS	POS	POS	POS	POS	POS	NEG	NEG	AMB	POS	POS	POS
MSSA2088	CC8	I	t008	POS	POS	POS	POS	POS	POS	POS	POS	NEG	NEG	AMB	POS	POS	POS
MSSA2187	CC8	I	t190	POS	POS	POS	POS	POS	POS	POS	POS	NEG	NEG	AMB	POS	POS	POS
MSSA2204	CC8	I	t008	POS	POS	POS	POS	POS	POS	POS	POS	NEG	NEG	AMB	POS	POS	POS
MSSA2210	CC8	I	t008	POS	POS	POS	POS	POS	POS	POS	POS	NEG	NEG	NEG	POS	POS	POS
MSSA2223	CC8	I	t008	POS	POS	POS	POS	POS	POS	POS	POS	NEG	NEG	NEG	POS	POS	POS
MSSA1763	CC9	II	t209	POS	POS	POS	POS	POS	POS	POS	NEG	POS	NEG	NEG	POS	POS	POS
MSSA1777	CC9	II	t100	POS	POS	POS	POS	POS	POS	POS	NEG	POS	NEG	NEG	POS	POS	POS
MSSA2009	CC9	II	t209	POS	POS	POS	POS	POS	POS	POS	NEG	POS	NEG	NEG	POS	POS	POS
MSSA2047	CC9	II	t100	POS	POS	POS	POS	POS	POS	POS	NEG	POS	NEG	NEG	POS	POS	POS
MSSA2079	CC9	II	t100	POS	POS	POS	POS	POS	POS	POS	NEG	POS	NEG	NEG	POS	POS	POS
MSSA2101	CC9	II	t100	POS	POS	POS	POS	POS	POS	POS	NEG	POS	NEG	NEG	POS	POS	POS
MSSA1821	CC12	II	t160	POS	POS	POS	POS	POS	POS	POS	NEG	POS	NEG	NEG	POS	POS	POS
MSSA1898	CC12	II	t3697	POS	POS	POS	POS	POS	POS	POS	NEG	POS	NEG	NEG	POS	POS	POS
MSSA1973	CC12	II	t160	POS	POS	POS	POS	POS	POS	POS	NEG	POS	NEG	NEG	POS	POS	POS
MSSA2071	CC12	II	t160	POS	POS	POS	POS	POS	POS	POS	NEG	POS	NEG	NEG	POS	POS	POS
MSSA2083	CC12	II	t3938	POS	POS	POS	POS	POS	POS	POS	NEG	POS	NEG	NEG	POS	POS	POS
MSSA2106	CC12	II	t160	POS	POS	POS	POS	POS	POS	POS	NEG	POS	NEG	NEG	POS	POS	POS
MSSA2112	CC12	II	t156	POS	POS	POS	POS	POS	POS	POS	NEG	POS	NEG	NEG	POS	POS	POS
MSSA2115	CC12	II	t156	POS	POS	POS	POS	POS	POS	POS	NEG	POS	NEG	NEG	POS	POS	POS
MSSA2195	CC12	II	t160	POS	POS	POS	POS	POS	POS	POS	NEG	POS	NEG	NEG	POS	POS	POS
MSSA2207	CC12	II	t160	POS	POS	POS	POS	POS	POS	POS	NEG	POS	NEG	NEG	POS	POS	POS
MSSA1758	CC15	II	t3097	POS	POS	POS	POS	POS	POS	POS	NEG	POS	NEG	NEG	POS	POS	POS
MSSA1762	CC15	II	t346	POS	POS	POS	POS	POS	POS	POS	NEG	POS	NEG	NEG	POS	POS	POS
MSSA1769	CC15	II	t084	POS	POS	POS	POS	POS	POS	POS	NEG	POS	NEG	NEG	POS	POS	POS
MSSA1771	CC15	II	t6286	POS	POS	POS	POS	POS	POS	POS	NEG	POS	NEG	NEG	POS	POS	POS
MSSA1789	CC15	II	t279	POS	POS	POS	POS	POS	POS	POS	NEG	POS	NEG	NEG	POS	POS	POS
MSSA1800	CC15	II	t085	POS	POS	POS	POS	POS	POS	POS	NEG	POS	NEG	NEG	POS	POS	POS
MSSA1833	CC15	II	t094	POS	POS	POS	POS	POS	POS	POS	NEG	POS	NEG	NEG	POS	POS	POS
MSSA1890	CC15	II	t084	POS	POS	POS	POS	POS	POS	POS	NEG	POS	NEG	NEG	POS	POS	POS
MSSA1903	CC15	II	t254	POS	POS	POS	POS	POS	POS	POS	NEG	POS	NEG	NEG	POS	POS	POS
MSSA1940	CC15	II	t144	POS	POS	POS	POS	POS	POS	POS	NEG	POS	NEG	NEG	POS	POS	POS
MSSA1962	CC15	II	t360	POS	POS	POS	POS	POS	POS	POS	NEG	POS	NEG	NEG	POS	POS	POS
MSSA1963	CC15	II	t360	POS	POS	POS	POS	POS	POS	POS	NEG	POS	NEG	NEG	POS	POS	POS
MSSA2016	CC15	II	t084	POS	POS	POS	POS	POS	POS	POS	NEG	POS	NEG	NEG	POS	POS	POS
MSSA2018	CC15	II	t346	POS	POS	POS	POS	POS	POS	POS	NEG	POS	NEG	NEG	POS	POS	POS
MSSA2019	CC15	II	t084	POS	POS	POS	POS	POS	POS	POS	NEG	POS	NEG	NEG	POS	POS	POS
MSSA2029	CC15	II	t491	POS	POS	POS	POS	POS	POS	POS	NEG	POS	NEG	NEG	POS	POS	POS
MSSA2045	CC15	II	t084	POS	POS	POS	POS	POS	POS	POS	NEG	POS	NEG	NEG	POS	POS	POS
MSSA2048	CC15	II	t1492	POS	POS	POS	POS	POS	POS	POS	NEG	POS	NEG	NEG	POS	POS	POS
MSSA2049	CC15	II	t084	POS	POS	POS	POS	POS	POS	POS	NEG	POS	NEG	NEG	POS	POS	POS
MSSA2050	CC15	II	t3698	POS	POS	POS	POS	POS	POS	POS	NEG	POS	NEG	NEG	POS	POS	POS
MSSA2099	CC15	II	t084	POS	POS	POS	AMB	POS	POS	POS	NEG	POS	NEG	NEG	POS	POS	POS
MSSA2109	CC15	II	t1509	POS	POS	POS	POS	POS	POS	POS	NEG	POS	NEG	NEG	POS	POS	POS
MSSA2130	CC15	II	t254	POS	POS	POS	POS	POS	POS	POS	NEG	POS	NEG	NEG	POS	POS	POS
MSSA2133	CC15	II	t084	POS	POS	POS	POS	POS	POS	POS	NEG	POS	NEG	NEG	POS	POS	POS
MSSA2139	CC15	II	t084	POS	POS	POS	POS	POS	POS	POS	NEG	POS	NEG	NEG	POS	POS	POS
MSSA2141	CC15	II	t346	POS	POS	POS	POS	POS	POS	POS	NEG	POS	NEG	NEG	POS	POS	POS
MSSA2208	CC15	II	t084	POS	POS	POS	POS	POS	POS	POS	NEG	POS	NEG	NEG	POS	POS	POS
MSSA2209	CC15	II	t084	POS	POS	POS	POS	POS	POS	POS	NEG	POS	NEG	NEG	POS	POS	POS
MSSA1893	CC20	I	t1544	POS	POS	POS	POS	POS	POS	POS	POS	NEG	NEG	NEG	POS	POS	POS
MSSA2105	CC20	I	t164	POS	POS	POS	POS	POS	POS	POS	POS	NEG	NEG	NEG	POS	POS	POS
MRSA3836	CC22	I	t032	POS	POS	POS	POS	POS	POS	POS	POS	NEG	NEG	NEG	POS	POS	POS
MRSA3840	CC22	I	t032	POS	POS	POS	POS	POS	POS	POS	POS	NEG	NEG	NEG	POS	POS	POS
MRSA4188	CC22	I	t032	POS	POS	POS	POS	POS	POS	POS	POS	NEG	NEG	AMB	POS	POS	POS
MSSA1814	CC22	I	t1556	POS	POS	POS	POS	POS	POS	POS	POS	NEG	NEG	NEG	POS	POS	POS
MSSA1816	CC22	I	t608	POS	POS	POS	POS	POS	POS	POS	POS	NEG	NEG	NEG	POS	POS	POS
MSSA1825	CC22	I	t005	POS	POS	POS	POS	POS	POS	POS	POS	NEG	NEG	NEG	POS	POS	POS
MSSA1834	CC22	I	t608	POS	POS	POS	POS	POS	POS	POS	POS	NEG	NEG	NEG	POS	POS	POS
MSSA1938	CC22	I	t005	POS	POS	POS	POS	POS	POS	POS	POS	NEG	NEG	NEG	POS	POS	POS
MSSA1948	CC22	I	t2790	POS	POS	POS	POS	POS	POS	POS	POS	NEG	NEG	NEG	POS	POS	POS
MSSA1951	CC22	I	t005	POS	POS	POS	POS	POS	POS	POS	POS	NEG	NEG	NEG	POS	POS	POS
MSSA2005	CC22	I	t005	POS	POS	POS	POS	POS	POS	POS	POS	NEG	NEG	NEG	POS	POS	POS
MSSA2051	CC22	I	t608	POS	POS	POS	POS	POS	POS	POS	POS	NEG	NEG	AMB	POS	POS	POS
MSSA2056	CC22	I	t476	POS	POS	POS	POS	POS	POS	POS	POS	NEG	NEG	NEG	POS	POS	POS
MSSA2066	CC22	I	t005	POS	POS	POS	POS	POS	POS	POS	POS	NEG	NEG	AMB	POS	POS	POS
MSSA2067	CC22	I	t005	POS	POS	POS	POS	POS	POS	POS	POS	NEG	NEG	NEG	POS	POS	POS
MSSA2086	CC22	I	t7573	POS	POS	POS	POS	POS	POS	POS	POS	NEG	NEG	NEG	POS	POS	POS
MSSA2089	CC22	I	t005	POS	POS	POS	POS	POS	POS	POS	POS	NEG	NEG	NEG	POS	POS	POS
MSSA2103	CC22	I	t005	POS	POS	POS	POS	POS	POS	POS	POS	NEG	NEG	NEG	POS	POS	POS
MSSA2173	CC22	I	t608	POS	POS	POS	POS	POS	POS	POS	POS	NEG	NEG	NEG	POS	POS	POS
MSSA2183	CC22	I	t2518	POS	POS	POS	POS	POS	POS	POS	POS	NEG	NEG	NEG	POS	POS	POS
MSSA2196	CC22	I	t608	POS	POS	POS	POS	POS	POS	POS	POS	NEG	NEG	NEG	POS	POS	POS
MSSA2219	CC22	I	t032	POS	POS	POS	POS	POS	POS	POS	POS	NEG	NEG	AMB	POS	POS	POS
MSSA1753	CC25	I	t081	POS	POS	POS	POS	POS	POS	POS	POS	NEG	NEG	NEG	POS	POS	POS
MSSA1773	CC25	I	t1350	POS	POS	POS	POS	POS	POS	POS	POS	NEG	NEG	NEG	POS	POS	POS
MSSA1811	CC25	I	t1315	POS	POS	POS	POS	POS	POS	POS	POS	NEG	NEG	NEG	POS	POS	POS
MSSA1897	CC25	I	t401	POS	POS	POS	POS	POS	POS	POS	POS	NEG	NEG	AMB	POS	POS	POS
MSSA1931	CC25	I	t6658	POS	POS	POS	POS	POS	POS	POS	POS	NEG	NEG	NEG	POS	POS	POS
MSSA2114	CC25	I	t7953	POS	POS	POS	POS	POS	POS	POS	POS	NEG	NEG	AMB	POS	POS	POS
MSSA1745	CC30	III	t093	POS	POS	POS	POS	POS	POS	POS	NEG	NEG	POS	NEG	POS	POS	POS
MSSA1747	CC30	III	t012	POS	POS	POS	POS	POS	POS	POS	NEG	NEG	POS	NEG	POS	POS	POS
MSSA1749	CC30	III	t012	POS	POS	POS	POS	POS	POS	POS	NEG	NEG	POS	NEG	POS	POS	POS
MSSA1768	CC30	III	t974	POS	POS	POS	POS	POS	POS	POS	NEG	NEG	POS	NEG	POS	POS	POS
MSSA1774	CC30	III	t1552	POS	POS	POS	POS	POS	POS	POS	NEG	NEG	POS	NEG	POS	POS	POS
MSSA1775	CC30	III	t021	POS	POS	POS	POS	POS	POS	POS	NEG	NEG	POS	NEG	POS	POS	POS
MSSA1776	CC30	III	t122	POS	POS	POS	POS	POS	POS	POS	NEG	NEG	POS	NEG	POS	POS	POS
MSSA1779	CC30	III	t1988	POS	POS	POS	POS	POS	POS	POS	NEG	NEG	POS	NEG	POS	POS	POS
MSSA1780	CC30	III	t012	POS	POS	POS	POS	POS	POS	POS	NEG	NEG	POS	NEG	POS	POS	POS

				SPECIES MARKERS							REGULATORY GENES						
**Strain**	**Clonal complex**	***agr* group**	**spa-type**	**Ribos STAU**	**gapA**	**katA**	**CoA**	**proteinA**	**sbi**	**nuc**	**agrI (total)**	**agrII (total)**	**agrIII (total)**	**agrIV (total)**	**sarA**	**saeS**	**vraS**
MSSA1781	CC30	III	t840	POS	POS	POS	POS	POS	POS	POS	NEG	NEG	POS	NEG	POS	POS	POS
MSSA1786	CC30	III	t1515	POS	POS	POS	POS	POS	POS	POS	NEG	NEG	POS	NEG	POS	POS	POS
MSSA1793	CC30	III	t6285	POS	POS	POS	POS	POS	POS	POS	NEG	NEG	POS	NEG	POS	POS	POS
MSSA1803	CC30	III	t012	POS	POS	POS	POS	POS	POS	POS	NEG	NEG	POS	NEG	POS	POS	POS
MSSA1836	CC30	III	t6011	POS	POS	POS	POS	POS	POS	POS	NEG	NEG	POS	NEG	POS	POS	POS
MSSA1839	CC30	III	t238	POS	POS	POS	POS	POS	POS	POS	NEG	NEG	POS	NEG	POS	POS	POS
MSSA1900	CC30	III	t012	POS	POS	POS	POS	POS	POS	POS	NEG	NEG	POS	NEG	POS	POS	POS
MSSA1910	CC30	III	t153	POS	POS	POS	POS	POS	POS	POS	NEG	NEG	POS	NEG	POS	POS	POS
MSSA1918	CC30	III	t089	POS	POS	POS	POS	POS	POS	POS	NEG	NEG	POS	NEG	POS	POS	POS
MSSA1921	CC30	III	t1397	POS	POS	POS	POS	POS	POS	POS	NEG	NEG	POS	NEG	POS	POS	POS
MSSA1923	CC30	III	t300	POS	POS	POS	POS	POS	POS	POS	NEG	NEG	POS	NEG	POS	POS	POS
MSSA1981	CC30	III	t1067	POS	POS	POS	POS	POS	POS	POS	NEG	NEG	POS	NEG	POS	POS	POS
MSSA1998	CC30	III	t318	POS	POS	POS	POS	POS	POS	POS	NEG	NEG	POS	NEG	POS	POS	POS
MSSA2000	CC30	III	t153	POS	POS	POS	POS	POS	POS	POS	NEG	NEG	POS	NEG	POS	POS	POS
MSSA2007	CC30	III	t012	POS	POS	POS	POS	POS	POS	POS	NEG	NEG	POS	NEG	POS	POS	POS
MSSA2012	CC30	III	t021	POS	POS	POS	POS	POS	POS	POS	NEG	NEG	POS	NEG	POS	POS	POS
MSSA2015	CC30	III	t012	POS	POS	POS	POS	POS	POS	POS	NEG	NEG	POS	NEG	POS	POS	POS
MSSA2021	CC30	III	t012	POS	POS	POS	POS	POS	POS	POS	NEG	NEG	POS	NEG	POS	POS	POS
MSSA2027	CC30	III	t021	POS	POS	POS	POS	POS	POS	POS	NEG	NEG	POS	NEG	POS	POS	POS
MSSA2063	CC30	III	t012	POS	POS	POS	POS	POS	POS	POS	NEG	NEG	POS	NEG	POS	POS	POS
MSSA2064	CC30	III	t1749	POS	POS	POS	POS	POS	POS	POS	NEG	NEG	POS	NEG	POS	POS	POS
MSSA2065	CC30	III	t7408	POS	POS	POS	POS	POS	POS	POS	NEG	NEG	POS	NEG	POS	POS	POS
MSSA2075	CC30	III	t012	POS	POS	POS	POS	POS	POS	POS	NEG	NEG	POS	NEG	POS	POS	POS
MSSA2093	CC30	III	t7507	POS	POS	POS	POS	POS	POS	POS	NEG	NEG	POS	NEG	POS	POS	POS
MSSA2102	CC30	III	t012	POS	POS	POS	POS	POS	POS	POS	NEG	NEG	POS	NEG	POS	POS	POS
MSSA2138	CC30	III	t342	POS	POS	POS	POS	POS	POS	POS	NEG	NEG	POS	NEG	POS	POS	POS
MSSA2168	CC30	III	t012	POS	POS	POS	POS	POS	POS	POS	NEG	NEG	POS	NEG	POS	POS	POS
MSSA2181	CC30	III	t019	POS	POS	POS	POS	POS	POS	POS	NEG	NEG	POS	NEG	POS	POS	POS
MSSA2189	CC30	III	t166	POS	POS	POS	POS	POS	POS	POS	NEG	NEG	POS	NEG	POS	POS	POS
MSSA2214	CC30	III	t012	POS	POS	POS	POS	POS	POS	POS	NEG	NEG	POS	NEG	POS	POS	POS
MRSA3803	CC45	I	t038	POS	POS	POS	POS	POS	POS	POS	POS	NEG	NEG	NEG	POS	POS	POS
MSSA1750	CC45	I	t026	POS	POS	POS	POS	POS	POS	POS	POS	NEG	NEG	NEG	POS	POS	POS
MSSA1756	CC45	I	t050	POS	POS	POS	POS	POS	POS	POS	POS	NEG	NEG	NEG	POS	POS	POS
MSSA1759	CC45	I	t7608	POS	POS	POS	POS	POS	POS	POS	POS	NEG	NEG	NEG	POS	POS	POS
MSSA1767	CC45	I	t015	POS	POS	POS	POS	POS	POS	POS	POS	NEG	NEG	NEG	POS	POS	POS
MSSA1787	CC45	I	t3783	POS	POS	POS	POS	POS	POS	POS	POS	NEG	NEG	NEG	POS	POS	POS
MSSA1790	CC45	I	t015	POS	POS	POS	POS	POS	POS	POS	POS	NEG	NEG	NEG	POS	POS	POS
MSSA1794	CC45	I	t050	POS	POS	POS	POS	POS	POS	POS	POS	NEG	NEG	NEG	POS	POS	POS
MSSA1808	CC45	I	t1040	POS	POS	POS	POS	POS	POS	POS	POS	NEG	NEG	NEG	POS	POS	POS
MSSA1812	CC45	I	t2171	POS	POS	POS	POS	POS	POS	POS	POS	NEG	NEG	NEG	POS	POS	POS
MSSA1819	CC45	I	t015	POS	POS	POS	POS	POS	POS	POS	POS	NEG	NEG	NEG	POS	POS	POS
MSSA1824	CC45	I	t331	POS	POS	POS	POS	POS	POS	POS	POS	NEG	NEG	NEG	POS	POS	POS
MSSA1827	CC45	I	t015	POS	POS	POS	POS	POS	POS	POS	POS	NEG	NEG	NEG	POS	POS	POS
MSSA1889	CC45	I	t015	POS	POS	POS	POS	POS	POS	POS	POS	NEG	NEG	NEG	POS	POS	POS
MSSA1895	CC45	I	t6394	POS	POS	POS	POS	POS	POS	POS	POS	NEG	NEG	NEG	POS	POS	POS
MSSA1904	CC45	I	t230	POS	POS	POS	POS	POS	POS	POS	POS	NEG	NEG	NEG	POS	POS	POS
MSSA1905	CC45	I	t630	POS	POS	POS	POS	POS	POS	POS	POS	NEG	NEG	NEG	POS	POS	POS
MSSA1907	CC45	I	t015	POS	POS	POS	POS	POS	POS	POS	POS	NEG	NEG	NEG	POS	POS	POS
MSSA1919	CC45	I	t031	POS	POS	POS	POS	POS	POS	POS	POS	NEG	NEG	NEG	POS	POS	POS
MSSA1920	CC45	I	t3176	POS	POS	POS	POS	POS	POS	POS	POS	NEG	NEG	NEG	POS	POS	POS
MSSA1929	CC45	I	t247	POS	POS	POS	POS	POS	POS	POS	POS	NEG	NEG	NEG	POS	POS	POS
MSSA1930	CC45	I	t015	POS	POS	POS	POS	POS	POS	POS	POS	NEG	NEG	NEG	POS	POS	POS
MSSA1934	CC45	I	t015	POS	POS	POS	POS	POS	POS	POS	POS	NEG	NEG	NEG	POS	POS	POS
MSSA1946	CC45	I	t065	POS	POS	POS	POS	POS	POS	POS	POS	NEG	NEG	NEG	POS	POS	POS
MSSA1971	CC45	I	t015	POS	POS	POS	POS	POS	POS	POS	POS	NEG	NEG	NEG	POS	POS	POS
MSSA1980	CC45	I	t6182	POS	POS	POS	POS	POS	POS	POS	POS	NEG	NEG	NEG	POS	POS	POS
MSSA1982	CC45	I	t550	POS	POS	POS	POS	POS	POS	POS	POS	NEG	NEG	NEG	POS	POS	POS
MSSA1996	CC45	I	t015	POS	POS	POS	POS	POS	POS	POS	POS	NEG	NEG	NEG	POS	POS	POS
MSSA2002	CC45	I	t040	POS	POS	POS	POS	POS	POS	POS	POS	NEG	NEG	NEG	POS	POS	POS
MSSA2014	CC45	I	t620	POS	POS	POS	POS	POS	POS	POS	POS	NEG	NEG	NEG	POS	POS	POS
MSSA2053	CC45	I	t133	POS	POS	POS	POS	POS	POS	POS	POS	NEG	NEG	NEG	POS	POS	POS
MSSA2054	CC45	I	t445	POS	POS	POS	POS	POS	POS	POS	POS	NEG	NEG	NEG	POS	POS	POS
MSSA2058	CC45	I	t331	POS	POS	POS	POS	POS	POS	POS	AMB	NEG	NEG	NEG	POS	POS	POS
MSSA2068	CC45	I	t015	POS	POS	POS	POS	POS	POS	POS	POS	NEG	NEG	NEG	POS	POS	POS
MSSA2070	CC45	I	t015	POS	POS	POS	POS	POS	POS	POS	POS	NEG	NEG	NEG	POS	POS	POS
MSSA2072	CC45	I	t1402	POS	POS	POS	POS	POS	POS	POS	POS	NEG	NEG	NEG	POS	POS	POS
MSSA2073	CC45	I	t004	POS	POS	POS	POS	AMB	AMB	POS	AMB	NEG	NEG	NEG	POS	POS	POS
MSSA2074	CC45	I	t1238	POS	POS	POS	POS	POS	POS	POS	POS	NEG	NEG	NEG	POS	POS	POS
MSSA2107	CC45	I	t015	POS	POS	POS	POS	POS	POS	POS	POS	NEG	NEG	NEG	POS	POS	POS
MSSA2108	CC45	I	t371	POS	POS	POS	POS	POS	POS	POS	POS	NEG	NEG	NEG	POS	POS	POS
MSSA2129	CC45	I	t630	POS	POS	POS	POS	POS	POS	POS	POS	NEG	NEG	NEG	POS	POS	POS
MSSA2135	CC45	I	t015	POS	POS	POS	POS	POS	POS	POS	POS	NEG	NEG	NEG	POS	POS	POS
MSSA2137	CC45	I	t2066	POS	POS	POS	POS	POS	POS	POS	AMB	NEG	NEG	POS	POS	POS	POS
MSSA2140	CC45	I	t230	POS	POS	POS	POS	POS	POS	POS	POS	NEG	NEG	NEG	POS	POS	POS
MSSA2182	CC45	I	t7952	POS	POS	POS	POS	POS	POS	POS	POS	NEG	NEG	NEG	POS	POS	POS
MSSA2188	CC45	I	t116	POS	POS	POS	POS	POS	POS	POS	POS	NEG	NEG	NEG	POS	POS	POS
MSSA1899	CC59	I	t7954	POS	POS	POS	POS	POS	POS	POS	POS	NEG	NEG	AMB	POS	POS	POS
MSSA1915	CC59	I	t216	POS	POS	POS	POS	POS	POS	POS	POS	NEG	NEG	NEG	POS	POS	POS
MSSA1949	CC59	I	t216	POS	POS	POS	POS	POS	POS	POS	POS	NEG	NEG	NEG	POS	POS	POS
MSSA2042	CC59	I	t216	POS	POS	POS	POS	POS	POS	POS	POS	NEG	NEG	NEG	POS	POS	POS
MSSA2077	CC59	I	t7694	POS	POS	POS	POS	POS	POS	POS	POS	NEG	NEG	NEG	POS	POS	POS
MSSA2095	CC59	I	t216	POS	POS	POS	POS	POS	POS	POS	POS	NEG	NEG	AMB	POS	POS	POS
MSSA1997	CC78	III	t3183	POS	POS	POS	POS	POS	POS	POS	NEG	NEG	POS	NEG	POS	POS	POS
MSSA1810	CC80	III	t878	POS	POS	POS	POS	POS	POS	POS	NEG	NEG	POS	NEG	POS	POS	POS
MSSA1832	CC97	I	t224	POS	POS	POS	POS	POS	POS	POS	POS	NEG	NEG	NEG	POS	POS	POS
MSSA1891	CC97	I	t1236	POS	POS	POS	POS	POS	POS	POS	POS	NEG	NEG	AMB	POS	POS	POS
MSSA1892	CC97	I	t3051	POS	POS	POS	POS	POS	POS	POS	POS	NEG	NEG	NEG	POS	POS	POS
MSSA1932	CC97	I	t3051	POS	POS	POS	POS	POS	POS	POS	POS	NEG	NEG	NEG	POS	POS	POS
MSSA1942	CC97	I	t359	POS	POS	POS	POS	POS	POS	POS	POS	NEG	NEG	NEG	POS	POS	POS
MSSA1957	CC97	I	t359	POS	POS	POS	POS	POS	POS	POS	POS	NEG	NEG	NEG	POS	POS	POS
MSSA1748	CC101	I	t2497	POS	POS	POS	POS	POS	POS	POS	POS	NEG	NEG	AMB	POS	POS	POS
MSSA1754	CC101	I	t056	POS	POS	POS	POS	POS	POS	POS	POS	NEG	NEG	NEG	POS	POS	POS
MSSA1770	CC101	I	t056	POS	POS	POS	POS	POS	POS	POS	POS	NEG	NEG	NEG	POS	POS	POS
MSSA1788	CC101	I	t056	POS	POS	POS	POS	POS	POS	POS	POS	NEG	NEG	NEG	POS	POS	POS
MSSA1804	CC101	I	t1312	POS	POS	POS	POS	POS	POS	POS	POS	NEG	NEG	NEG	POS	POS	POS
MSSA1818	CC101	I	t3923	POS	POS	POS	POS	POS	POS	POS	POS	NEG	NEG	NEG	POS	POS	POS
MSSA1914	CC101	I	t056	POS	POS	POS	POS	POS	POS	POS	POS	NEG	NEG	AMB	POS	POS	POS
MSSA1974	CC101	I	t056	POS	POS	POS	POS	POS	POS	POS	POS	NEG	NEG	NEG	POS	POS	POS
MSSA2023	CC101	I	t1168	POS	POS	POS	POS	POS	POS	POS	POS	NEG	NEG	NEG	POS	POS	POS
MSSA2030	CC101	I	t056	POS	POS	POS	POS	POS	POS	POS	POS	NEG	NEG	NEG	POS	POS	POS
MSSA2096	CC101	I	t056	POS	POS	POS	AMB	POS	POS	POS	POS	NEG	NEG	NEG	POS	POS	POS
MSSA2142	CC101	I	t056	POS	POS	POS	POS	POS	POS	POS	POS	NEG	NEG	NEG	POS	POS	POS
MSSA2203	CC101	I	t056	POS	POS	POS	POS	POS	POS	POS	POS	NEG	NEG	NEG	POS	POS	POS
MSSA1901	CC121	IV	t6393	POS	POS	POS	POS	POS	POS	POS	NEG	NEG	NEG	POS	POS	POS	POS
MSSA2013	CC121	IV	t645	POS	POS	POS	POS	POS	POS	POS	NEG	NEG	NEG	POS	POS	POS	POS
MSSA2031	CC121	IV	t6949	POS	POS	POS	POS	POS	POS	POS	AMB	NEG	NEG	POS	POS	POS	POS
MSSA2052	CC121	IV	t162	POS	POS	POS	POS	POS	POS	POS	NEG	NEG	NEG	POS	POS	POS	POS
MSSA2057	CC121	IV	t159	POS	POS	POS	POS	POS	POS	POS	NEG	NEG	NEG	POS	POS	POS	POS
MSSA2081	CC121	IV	t246	POS	POS	POS	POS	POS	POS	POS	AMB	NEG	NEG	POS	POS	POS	POS
MSSA1955	CC182	I	t5739	POS	POS	POS	POS	POS	POS	POS	POS	NEG	NEG	NEG	POS	POS	POS
MSSA1941	CC395	I	t1362	POS	POS	POS	POS	POS	POS	NEG	POS	NEG	NEG	NEG	POS	POS	POS
MRSA3838	CC398	I	t011	POS	POS	POS	POS	POS	POS	POS	POS	NEG	NEG	NEG	POS	POS	POS
MRSA3839	CC398	I	t1451	POS	POS	POS	POS	POS	POS	POS	POS	NEG	NEG	NEG	POS	POS	POS
MSSA1801	CC398	I	t779	POS	POS	POS	NEG	POS	POS	POS	AMB	NEG	NEG	NEG	POS	POS	POS
MSSA2131	CC398	I	t254	POS	POS	POS	POS	POS	POS	POS	POS	NEG	NEG	NEG	POS	POS	POS
MSSA1792	None	I	t148	POS	POS	POS	POS	POS	POS	POS	POS	NEG	NEG	NEG	POS	POS	POS
MSSA1796	None	None	t208	POS	POS	POS	POS	POS	POS	POS	NEG	POS	NEG	NEG	POS	POS	POS
MSSA1817	None	IV	t6676	POS	POS	POS	POS	POS	POS	POS	AMB	NEG	NEG	POS	POS	POS	POS
MSSA1911	None	None	nontypeable	POS	POS	NEG	NEG	NEG	NEG	POS	NEG	NEG	NEG	NEG	POS	POS	POS
MSSA1927	None	I	t701	POS	POS	POS	POS	POS	POS	POS	POS	NEG	NEG	NEG	POS	POS	POS
MSSA1928	None	I	t701	POS	POS	POS	POS	POS	POS	POS	POS	NEG	NEG	NEG	POS	POS	POS
MSSA1953	None	I	t701	POS	POS	POS	POS	POS	POS	POS	POS	NEG	NEG	NEG	POS	POS	POS
MSSA2062	None	I	t1631	POS	POS	POS	POS	POS	POS	POS	POS	NEG	NEG	NEG	POS	POS	POS
MSSA2078	None	None	t647	POS	POS	POS	POS	POS	POS	POS	NEG	POS	NEG	NEG	POS	POS	POS
MSSA2136	None	None	t1931	POS	POS	POS	POS	POS	POS	POS	POS	NEG	NEG	AMB	POS	POS	POS

	METHICILLIN RESISTANCE AND SCCmec TYPING														
Strain	mecA	mecR1 / delta mecR1	ugpQ	ccrA-1	ccrB-1 (probe 1)	ccrB-1 (probe 2)	plsSCC (COL)	Q9XB68-dcs	ccrA-2	ccrB-2	kdpA-SCC	kdpB-SCC	kdpC-SCC	kdpD-SCC	kdpE-SCC
MSSA1805	NEG	NEG	NEG	NEG	NEG	NEG	NEG	NEG	NEG	NEG	NEG	NEG	NEG	NEG	NEG
MSSA1823	NEG	NEG	NEG	NEG	NEG	NEG	NEG	NEG	NEG	NEG	NEG	NEG	NEG	NEG	NEG
MSSA1837	NEG	NEG	NEG	NEG	NEG	NEG	NEG	NEG	NEG	NEG	NEG	NEG	NEG	NEG	NEG
MSSA2044	NEG	NEG	NEG	NEG	NEG	NEG	NEG	NEG	NEG	NEG	NEG	NEG	NEG	NEG	NEG
MSSA2046	NEG	NEG	NEG	NEG	NEG	NEG	NEG	NEG	NEG	NEG	NEG	NEG	NEG	NEG	NEG
MSSA2059	NEG	NEG	NEG	NEG	NEG	NEG	NEG	NEG	NEG	NEG	NEG	NEG	NEG	NEG	NEG
MSSA2076	NEG	NEG	NEG	NEG	NEG	NEG	NEG	NEG	NEG	NEG	NEG	NEG	NEG	NEG	NEG
MSSA2200	NEG	NEG	NEG	NEG	NEG	NEG	NEG	NEG	NEG	NEG	NEG	NEG	NEG	NEG	NEG
MRSA3797	POS	POS	POS	NEG	NEG	NEG	NEG	POS	POS	POS	POS	POS	POS	POS	POS
MRSA3798	POS	POS	POS	NEG	AMB	NEG	NEG	POS	POS	POS	POS	POS	POS	POS	POS
MRSA3799	POS	POS	POS	NEG	NEG	NEG	NEG	POS	POS	POS	POS	POS	POS	POS	POS
MRSA3800	POS	POS	POS	NEG	NEG	NEG	NEG	POS	POS	POS	POS	POS	POS	POS	POS
MRSA3801	POS	POS	POS	NEG	AMB	NEG	NEG	POS	POS	POS	POS	POS	POS	POS	POS
MRSA3804	POS	POS	POS	NEG	AMB	NEG	NEG	POS	POS	POS	POS	POS	POS	POS	POS
MRSA3806	POS	POS	POS	NEG	AMB	NEG	NEG	POS	POS	POS	POS	POS	POS	POS	POS
MRSA3961	POS	POS	POS	NEG	NEG	NEG	NEG	POS	POS	POS	POS	POS	POS	POS	POS
MRSA3962	POS	POS	POS	NEG	NEG	NEG	NEG	POS	POS	POS	NEG	NEG	NEG	NEG	NEG
MRSA3963	POS	POS	POS	NEG	NEG	NEG	NEG	POS	POS	POS	POS	POS	POS	POS	POS
MRSA3966	POS	POS	POS	NEG	NEG	NEG	NEG	POS	POS	POS	POS	POS	POS	POS	POS
MRSA3978	POS	POS	POS	NEG	NEG	NEG	NEG	POS	POS	POS	POS	POS	POS	POS	POS
MRSA3979	POS	POS	POS	NEG	NEG	NEG	NEG	AMB	POS	POS	POS	POS	POS	POS	POS
MRSA3980	POS	POS	POS	NEG	NEG	NEG	NEG	POS	POS	POS	POS	POS	POS	POS	POS
MRSA4179	POS	POS	POS	NEG	NEG	NEG	NEG	POS	POS	POS	POS	POS	POS	POS	POS
MRSA4216	POS	POS	POS	NEG	NEG	NEG	NEG	POS	POS	POS	POS	POS	POS	POS	POS
MRSA4217	POS	POS	POS	NEG	NEG	NEG	NEG	POS	POS	POS	POS	POS	POS	POS	POS
MRSA4220	POS	POS	POS	NEG	NEG	NEG	NEG	POS	POS	POS	POS	POS	POS	POS	POS
MRSA4221	POS	POS	POS	NEG	NEG	NEG	NEG	POS	POS	POS	POS	POS	POS	POS	POS
MRSA4222	POS	POS	POS	NEG	NEG	NEG	NEG	POS	POS	POS	POS	POS	POS	POS	POS
MSSA1751	NEG	NEG	NEG	NEG	NEG	NEG	NEG	NEG	NEG	NEG	NEG	NEG	NEG	NEG	NEG
MSSA1760	NEG	NEG	NEG	NEG	AMB	NEG	NEG	NEG	NEG	NEG	NEG	NEG	NEG	NEG	NEG
MSSA1802	NEG	NEG	NEG	NEG	NEG	NEG	NEG	NEG	NEG	NEG	NEG	NEG	NEG	NEG	NEG
MSSA1809	NEG	NEG	NEG	NEG	NEG	NEG	NEG	NEG	NEG	NEG	NEG	NEG	NEG	NEG	NEG
MSSA1815	NEG	NEG	NEG	NEG	NEG	NEG	NEG	NEG	NEG	NEG	NEG	NEG	NEG	NEG	NEG
MSSA1829	NEG	NEG	NEG	NEG	NEG	NEG	NEG	NEG	NEG	NEG	NEG	NEG	NEG	NEG	NEG
MSSA1908	NEG	NEG	NEG	NEG	NEG	NEG	NEG	NEG	NEG	NEG	NEG	NEG	NEG	NEG	NEG
MSSA1960	NEG	NEG	NEG	NEG	NEG	NEG	NEG	NEG	NEG	NEG	NEG	NEG	NEG	NEG	NEG
MSSA1965	NEG	NEG	NEG	NEG	NEG	NEG	NEG	NEG	NEG	NEG	NEG	NEG	NEG	NEG	NEG
MSSA1977	NEG	NEG	NEG	NEG	NEG	NEG	NEG	NEG	NEG	NEG	NEG	NEG	NEG	NEG	NEG
MSSA1978	NEG	NEG	NEG	NEG	NEG	NEG	NEG	NEG	NEG	NEG	NEG	NEG	NEG	NEG	NEG
MSSA1999	NEG	NEG	NEG	NEG	NEG	NEG	NEG	NEG	NEG	NEG	NEG	NEG	NEG	NEG	NEG
MSSA2003	NEG	NEG	NEG	NEG	NEG	NEG	NEG	NEG	NEG	NEG	NEG	NEG	NEG	NEG	NEG
MSSA2006	NEG	NEG	NEG	NEG	NEG	NEG	NEG	NEG	NEG	NEG	NEG	NEG	NEG	NEG	NEG
MSSA2008	NEG	NEG	NEG	NEG	NEG	NEG	NEG	NEG	NEG	NEG	NEG	NEG	NEG	NEG	NEG
MSSA2011	POS	POS	POS	NEG	NEG	NEG	NEG	POS	POS	POS	POS	POS	POS	POS	POS
MSSA2017	NEG	NEG	NEG	NEG	NEG	NEG	NEG	NEG	NEG	NEG	NEG	NEG	NEG	NEG	NEG
MSSA2022	POS	POS	POS	NEG	NEG	NEG	NEG	POS	POS	POS	POS	POS	POS	POS	POS
MSSA2055	NEG	NEG	NEG	NEG	NEG	NEG	NEG	NEG	NEG	NEG	NEG	NEG	NEG	NEG	NEG
MSSA2060	NEG	NEG	NEG	NEG	NEG	NEG	NEG	NEG	NEG	NEG	NEG	NEG	NEG	NEG	NEG
MSSA2080	NEG	NEG	NEG	NEG	NEG	NEG	NEG	NEG	NEG	NEG	NEG	NEG	NEG	NEG	NEG
MSSA2082	NEG	NEG	NEG	NEG	NEG	NEG	NEG	NEG	NEG	NEG	NEG	NEG	NEG	NEG	NEG
MSSA2084	NEG	NEG	NEG	NEG	NEG	NEG	NEG	NEG	NEG	NEG	NEG	NEG	NEG	NEG	NEG
MSSA2087	NEG	NEG	NEG	NEG	NEG	NEG	NEG	NEG	NEG	NEG	NEG	NEG	NEG	NEG	NEG
MSSA2094	NEG	NEG	NEG	NEG	NEG	NEG	NEG	NEG	NEG	NEG	NEG	NEG	NEG	NEG	NEG
MSSA2097	NEG	NEG	NEG	NEG	NEG	NEG	NEG	NEG	NEG	NEG	NEG	NEG	NEG	NEG	NEG
MSSA2110	NEG	NEG	NEG	NEG	NEG	NEG	NEG	NEG	NEG	NEG	NEG	NEG	NEG	NEG	NEG
MSSA2113	NEG	NEG	NEG	NEG	NEG	NEG	NEG	NEG	NEG	NEG	NEG	NEG	NEG	NEG	NEG
MSSA2132	NEG	NEG	NEG	NEG	NEG	NEG	NEG	NEG	NEG	NEG	NEG	NEG	NEG	NEG	NEG
MSSA2134	NEG	NEG	NEG	NEG	NEG	NEG	NEG	NEG	NEG	NEG	NEG	NEG	NEG	NEG	NEG
MSSA1746	NEG	NEG	NEG	NEG	NEG	NEG	NEG	NEG	NEG	NEG	NEG	NEG	NEG	NEG	NEG
MSSA1757	NEG	NEG	NEG	NEG	AMB	NEG	NEG	NEG	NEG	NEG	NEG	NEG	NEG	NEG	NEG
MSSA1772	NEG	NEG	NEG	NEG	NEG	NEG	NEG	NEG	NEG	NEG	NEG	NEG	NEG	NEG	NEG
MSSA1795	NEG	NEG	NEG	NEG	AMB	NEG	NEG	NEG	NEG	NEG	NEG	NEG	NEG	NEG	NEG
MSSA1813	NEG	NEG	NEG	NEG	NEG	NEG	NEG	NEG	NEG	NEG	NEG	NEG	NEG	NEG	NEG
MSSA1826	NEG	NEG	NEG	NEG	NEG	NEG	NEG	NEG	NEG	NEG	NEG	NEG	NEG	NEG	NEG
MSSA1830	NEG	NEG	NEG	NEG	NEG	NEG	NEG	NEG	NEG	NEG	NEG	NEG	NEG	NEG	NEG
MSSA1838	NEG	NEG	NEG	NEG	NEG	NEG	NEG	NEG	NEG	NEG	NEG	NEG	NEG	NEG	NEG
MSSA1944	NEG	NEG	NEG	NEG	NEG	NEG	NEG	NEG	NEG	NEG	NEG	NEG	NEG	NEG	NEG
MSSA1947	NEG	NEG	NEG	NEG	NEG	NEG	NEG	NEG	NEG	NEG	NEG	NEG	NEG	NEG	NEG
MSSA1964	NEG	NEG	NEG	NEG	NEG	NEG	NEG	NEG	NEG	NEG	NEG	NEG	NEG	NEG	NEG
MSSA1969	NEG	NEG	NEG	NEG	NEG	NEG	NEG	NEG	NEG	NEG	NEG	NEG	NEG	NEG	NEG
MSSA2001	NEG	NEG	NEG	NEG	AMB	NEG	NEG	NEG	NEG	NEG	NEG	NEG	NEG	NEG	NEG
MSSA2004	NEG	NEG	NEG	NEG	NEG	NEG	NEG	NEG	NEG	NEG	NEG	NEG	NEG	NEG	NEG
MSSA2024	NEG	NEG	NEG	NEG	NEG	NEG	NEG	NEG	NEG	NEG	NEG	NEG	NEG	NEG	NEG
MSSA2025	NEG	NEG	NEG	NEG	NEG	NEG	NEG	NEG	NEG	NEG	NEG	NEG	NEG	NEG	NEG
MSSA2026	NEG	NEG	NEG	NEG	NEG	NEG	NEG	NEG	NEG	NEG	NEG	NEG	NEG	NEG	NEG
MSSA2032	NEG	NEG	NEG	NEG	NEG	NEG	NEG	NEG	NEG	NEG	NEG	NEG	NEG	NEG	NEG
MSSA2043	NEG	NEG	NEG	NEG	NEG	NEG	NEG	NEG	NEG	NEG	NEG	NEG	NEG	NEG	NEG
MSSA2061	NEG	NEG	NEG	NEG	NEG	NEG	NEG	NEG	NEG	NEG	NEG	NEG	NEG	NEG	NEG
MSSA2085	NEG	NEG	NEG	NEG	NEG	NEG	NEG	NEG	NEG	NEG	NEG	NEG	NEG	NEG	NEG
MSSA2098	NEG	NEG	NEG	NEG	NEG	NEG	NEG	NEG	NEG	NEG	NEG	NEG	NEG	NEG	NEG
MSSA2100	NEG	NEG	NEG	NEG	NEG	NEG	NEG	NEG	NEG	NEG	NEG	NEG	NEG	NEG	NEG
MSSA2111	NEG	NEG	NEG	NEG	AMB	NEG	NEG	NEG	NEG	NEG	NEG	NEG	NEG	NEG	NEG
MSSA2178	NEG	NEG	NEG	NEG	AMB	NEG	NEG	NEG	NEG	NEG	NEG	NEG	NEG	NEG	NEG
MSSA2202	NEG	NEG	NEG	NEG	NEG	NEG	NEG	NEG	NEG	NEG	NEG	NEG	NEG	NEG	NEG
MSSA2205	NEG	NEG	NEG	NEG	AMB	NEG	NEG	NEG	NEG	NEG	NEG	NEG	NEG	NEG	NEG
MRSA3796	POS	POS	POS	NEG	NEG	NEG	NEG	POS	POS	POS	NEG	NEG	NEG	NEG	NEG
MRSA3805	POS	POS	POS	NEG	AMB	AMB	NEG	POS	NEG	NEG	NEG	NEG	NEG	NEG	NEG
MRSA3965	POS	POS	POS	POS	POS	POS	POS	POS	NEG	NEG	NEG	NEG	NEG	NEG	NEG
MSSA1761	NEG	NEG	NEG	POS	AMB	AMB	NEG	NEG	NEG	NEG	NEG	NEG	NEG	NEG	NEG
MSSA1764	NEG	NEG	NEG	NEG	NEG	NEG	NEG	NEG	NEG	NEG	NEG	NEG	NEG	NEG	NEG
MSSA1765	NEG	NEG	NEG	NEG	NEG	NEG	NEG	NEG	NEG	NEG	NEG	NEG	NEG	NEG	NEG
MSSA1784	NEG	NEG	NEG	NEG	AMB	NEG	NEG	NEG	NEG	NEG	NEG	NEG	NEG	NEG	NEG
MSSA1806	NEG	NEG	NEG	NEG	NEG	NEG	NEG	NEG	NEG	NEG	NEG	NEG	NEG	NEG	NEG
MSSA1807	NEG	NEG	NEG	NEG	NEG	NEG	NEG	NEG	NEG	NEG	NEG	NEG	NEG	NEG	NEG
MSSA1888	NEG	NEG	NEG	NEG	NEG	NEG	NEG	NEG	NEG	NEG	NEG	NEG	NEG	NEG	NEG
MSSA1894	NEG	NEG	NEG	NEG	NEG	NEG	NEG	NEG	NEG	NEG	NEG	NEG	NEG	NEG	NEG
MSSA1922	NEG	NEG	NEG	NEG	NEG	NEG	NEG	NEG	NEG	NEG	NEG	NEG	NEG	NEG	NEG
MSSA1950	NEG	NEG	NEG	NEG	NEG	NEG	NEG	NEG	NEG	NEG	NEG	NEG	NEG	NEG	NEG
MSSA1954	NEG	NEG	NEG	NEG	NEG	NEG	NEG	NEG	NEG	NEG	NEG	NEG	NEG	NEG	NEG
MSSA1966	NEG	NEG	NEG	NEG	NEG	NEG	NEG	NEG	NEG	NEG	NEG	NEG	NEG	NEG	NEG
MSSA1972	NEG	NEG	NEG	NEG	NEG	NEG	NEG	NEG	NEG	NEG	NEG	NEG	NEG	NEG	NEG
MSSA2010	NEG	NEG	NEG	NEG	NEG	NEG	NEG	NEG	NEG	NEG	NEG	NEG	NEG	NEG	NEG
MSSA2020	NEG	NEG	NEG	NEG	NEG	NEG	NEG	NEG	NEG	NEG	NEG	NEG	NEG	NEG	NEG
MSSA2028	NEG	NEG	NEG	NEG	NEG	NEG	NEG	NEG	NEG	NEG	NEG	NEG	NEG	NEG	NEG
MSSA2088	NEG	NEG	NEG	NEG	NEG	NEG	NEG	NEG	NEG	NEG	NEG	NEG	NEG	NEG	NEG
MSSA2187	NEG	NEG	NEG	POS	NEG	NEG	NEG	NEG	NEG	NEG	NEG	NEG	NEG	NEG	NEG
MSSA2204	NEG	NEG	NEG	NEG	AMB	NEG	NEG	NEG	NEG	NEG	NEG	NEG	NEG	NEG	NEG
MSSA2210	NEG	NEG	NEG	NEG	NEG	NEG	NEG	NEG	NEG	NEG	NEG	NEG	NEG	NEG	NEG
MSSA2223	NEG	NEG	NEG	NEG	NEG	NEG	NEG	NEG	NEG	NEG	NEG	NEG	NEG	NEG	NEG
MSSA1763	NEG	NEG	NEG	NEG	NEG	NEG	NEG	NEG	NEG	NEG	NEG	NEG	NEG	NEG	NEG
MSSA1777	NEG	NEG	NEG	NEG	NEG	NEG	NEG	NEG	NEG	NEG	NEG	NEG	NEG	NEG	NEG
MSSA2009	NEG	NEG	NEG	NEG	NEG	NEG	NEG	NEG	NEG	NEG	NEG	NEG	NEG	NEG	NEG
MSSA2047	NEG	NEG	NEG	NEG	NEG	NEG	NEG	NEG	NEG	NEG	NEG	NEG	NEG	NEG	NEG
MSSA2079	NEG	NEG	NEG	NEG	NEG	NEG	NEG	NEG	NEG	NEG	NEG	NEG	NEG	NEG	NEG
MSSA2101	NEG	NEG	NEG	NEG	NEG	NEG	NEG	NEG	NEG	NEG	NEG	NEG	NEG	NEG	NEG
MSSA1821	NEG	NEG	NEG	NEG	NEG	NEG	NEG	NEG	NEG	NEG	NEG	NEG	NEG	NEG	NEG
MSSA1898	NEG	NEG	NEG	NEG	NEG	NEG	NEG	NEG	NEG	NEG	NEG	NEG	NEG	NEG	NEG
MSSA1973	NEG	NEG	NEG	NEG	NEG	NEG	NEG	NEG	NEG	NEG	NEG	NEG	NEG	NEG	NEG
MSSA2071	NEG	NEG	NEG	NEG	NEG	NEG	NEG	NEG	NEG	NEG	NEG	NEG	NEG	NEG	NEG
MSSA2083	NEG	NEG	NEG	NEG	NEG	NEG	NEG	NEG	NEG	NEG	NEG	NEG	NEG	NEG	NEG
MSSA2106	NEG	NEG	NEG	NEG	NEG	NEG	NEG	NEG	NEG	NEG	NEG	NEG	NEG	NEG	NEG
MSSA2112	NEG	NEG	NEG	NEG	NEG	NEG	NEG	NEG	NEG	NEG	NEG	NEG	NEG	NEG	NEG
MSSA2115	NEG	NEG	NEG	NEG	AMB	NEG	NEG	NEG	NEG	NEG	NEG	NEG	NEG	NEG	NEG
MSSA2195	NEG	NEG	NEG	NEG	NEG	NEG	NEG	NEG	NEG	NEG	NEG	NEG	NEG	NEG	NEG
MSSA2207	NEG	NEG	NEG	NEG	NEG	NEG	NEG	NEG	NEG	NEG	NEG	NEG	NEG	NEG	NEG
MSSA1758	NEG	NEG	NEG	NEG	NEG	NEG	NEG	NEG	NEG	NEG	NEG	NEG	NEG	NEG	NEG
MSSA1762	NEG	NEG	NEG	NEG	AMB	NEG	NEG	NEG	NEG	NEG	NEG	NEG	NEG	NEG	NEG
MSSA1769	NEG	NEG	NEG	NEG	NEG	NEG	NEG	NEG	NEG	NEG	NEG	NEG	NEG	NEG	NEG
MSSA1771	NEG	NEG	NEG	NEG	NEG	NEG	NEG	NEG	NEG	NEG	NEG	NEG	NEG	NEG	NEG
MSSA1789	NEG	NEG	NEG	NEG	NEG	NEG	NEG	NEG	NEG	NEG	NEG	NEG	NEG	NEG	NEG
MSSA1800	NEG	NEG	NEG	NEG	NEG	NEG	NEG	NEG	NEG	NEG	NEG	NEG	NEG	NEG	NEG
MSSA1833	NEG	NEG	NEG	NEG	NEG	NEG	NEG	NEG	NEG	NEG	NEG	NEG	NEG	NEG	NEG
MSSA1890	NEG	NEG	NEG	NEG	NEG	NEG	NEG	NEG	NEG	NEG	NEG	NEG	NEG	NEG	NEG
MSSA1903	NEG	NEG	NEG	NEG	NEG	NEG	NEG	NEG	NEG	NEG	NEG	NEG	NEG	NEG	NEG
MSSA1940	NEG	NEG	NEG	NEG	NEG	NEG	NEG	NEG	NEG	NEG	NEG	NEG	NEG	NEG	NEG
MSSA1962	NEG	NEG	NEG	NEG	NEG	NEG	NEG	NEG	NEG	NEG	NEG	NEG	NEG	NEG	NEG
MSSA1963	NEG	NEG	NEG	NEG	NEG	NEG	NEG	NEG	NEG	NEG	NEG	NEG	NEG	NEG	NEG
MSSA2016	NEG	NEG	NEG	NEG	NEG	NEG	NEG	NEG	NEG	NEG	NEG	NEG	NEG	NEG	NEG
MSSA2018	NEG	NEG	NEG	NEG	NEG	AMB	NEG	NEG	NEG	NEG	NEG	NEG	NEG	NEG	NEG
MSSA2019	NEG	NEG	NEG	NEG	NEG	NEG	NEG	NEG	NEG	NEG	NEG	NEG	NEG	NEG	NEG
MSSA2029	NEG	NEG	NEG	NEG	NEG	NEG	NEG	NEG	NEG	NEG	NEG	NEG	NEG	NEG	NEG
MSSA2045	NEG	NEG	NEG	NEG	NEG	NEG	NEG	NEG	NEG	NEG	NEG	NEG	NEG	NEG	NEG
MSSA2048	NEG	NEG	NEG	NEG	NEG	NEG	NEG	NEG	NEG	NEG	NEG	NEG	NEG	NEG	NEG
MSSA2049	NEG	NEG	NEG	NEG	NEG	NEG	NEG	NEG	NEG	NEG	NEG	NEG	NEG	NEG	NEG
MSSA2050	NEG	NEG	NEG	NEG	AMB	NEG	NEG	NEG	NEG	NEG	NEG	NEG	NEG	NEG	NEG
MSSA2099	NEG	NEG	NEG	NEG	NEG	NEG	NEG	NEG	NEG	NEG	NEG	NEG	NEG	NEG	NEG
MSSA2109	NEG	NEG	NEG	NEG	NEG	NEG	NEG	NEG	NEG	NEG	NEG	NEG	NEG	NEG	NEG
MSSA2130	NEG	NEG	NEG	NEG	NEG	NEG	NEG	NEG	NEG	NEG	NEG	NEG	NEG	NEG	NEG
MSSA2133	NEG	NEG	NEG	NEG	NEG	NEG	NEG	NEG	NEG	NEG	NEG	NEG	NEG	NEG	NEG
MSSA2139	NEG	NEG	NEG	NEG	NEG	NEG	NEG	NEG	NEG	NEG	NEG	NEG	NEG	NEG	NEG
MSSA2141	NEG	NEG	NEG	NEG	NEG	NEG	NEG	NEG	NEG	NEG	NEG	NEG	NEG	NEG	NEG
MSSA2208	NEG	NEG	NEG	POS	NEG	NEG	NEG	NEG	NEG	NEG	NEG	NEG	NEG	NEG	NEG
MSSA2209	NEG	NEG	NEG	NEG	NEG	NEG	NEG	NEG	NEG	NEG	NEG	NEG	NEG	NEG	NEG
MSSA1893	NEG	NEG	NEG	NEG	NEG	NEG	NEG	NEG	NEG	NEG	NEG	NEG	NEG	NEG	NEG
MSSA2105	NEG	NEG	NEG	NEG	NEG	NEG	NEG	NEG	NEG	NEG	NEG	NEG	NEG	NEG	NEG
MRSA3836	POS	POS	POS	NEG	NEG	NEG	NEG	POS	POS	POS	NEG	NEG	NEG	NEG	NEG
MRSA3840	POS	POS	POS	NEG	NEG	NEG	NEG	POS	POS	POS	NEG	NEG	NEG	NEG	NEG
MRSA4188	POS	POS	POS	NEG	NEG	NEG	NEG	POS	POS	POS	NEG	NEG	NEG	NEG	NEG
MSSA1814	NEG	NEG	NEG	NEG	NEG	NEG	NEG	NEG	NEG	NEG	NEG	NEG	NEG	NEG	NEG
MSSA1816	NEG	NEG	NEG	NEG	NEG	NEG	NEG	NEG	NEG	NEG	NEG	NEG	NEG	NEG	NEG
MSSA1825	NEG	NEG	NEG	NEG	NEG	NEG	NEG	NEG	NEG	NEG	NEG	NEG	NEG	NEG	NEG
MSSA1834	NEG	NEG	NEG	NEG	NEG	NEG	NEG	NEG	NEG	NEG	NEG	NEG	NEG	NEG	NEG
MSSA1938	NEG	NEG	NEG	NEG	NEG	NEG	NEG	NEG	NEG	NEG	NEG	NEG	NEG	NEG	NEG
MSSA1948	NEG	NEG	NEG	NEG	NEG	NEG	NEG	NEG	NEG	NEG	NEG	NEG	NEG	NEG	NEG
MSSA1951	NEG	NEG	NEG	NEG	NEG	NEG	NEG	NEG	NEG	NEG	NEG	NEG	NEG	NEG	NEG
MSSA2005	NEG	NEG	NEG	NEG	NEG	NEG	NEG	NEG	NEG	NEG	NEG	NEG	NEG	NEG	NEG
MSSA2051	NEG	NEG	NEG	NEG	NEG	NEG	NEG	NEG	NEG	NEG	NEG	NEG	NEG	NEG	NEG
MSSA2056	NEG	NEG	NEG	NEG	NEG	NEG	NEG	NEG	NEG	NEG	NEG	NEG	NEG	NEG	NEG
MSSA2066	NEG	NEG	NEG	NEG	NEG	NEG	NEG	NEG	NEG	NEG	NEG	NEG	NEG	NEG	NEG
MSSA2067	NEG	NEG	NEG	NEG	NEG	NEG	NEG	NEG	NEG	NEG	NEG	NEG	NEG	NEG	NEG
MSSA2086	NEG	NEG	NEG	NEG	NEG	NEG	NEG	NEG	NEG	NEG	NEG	NEG	NEG	NEG	NEG
MSSA2089	NEG	NEG	NEG	NEG	NEG	NEG	NEG	NEG	NEG	NEG	NEG	NEG	NEG	NEG	NEG
MSSA2103	NEG	NEG	NEG	NEG	NEG	NEG	NEG	NEG	NEG	NEG	NEG	NEG	NEG	NEG	NEG
MSSA2173	NEG	NEG	NEG	NEG	NEG	NEG	NEG	NEG	NEG	NEG	NEG	NEG	NEG	NEG	NEG
MSSA2183	NEG	NEG	NEG	NEG	NEG	NEG	NEG	NEG	NEG	NEG	NEG	NEG	NEG	NEG	NEG
MSSA2196	NEG	NEG	NEG	NEG	NEG	NEG	NEG	NEG	NEG	NEG	NEG	NEG	NEG	NEG	NEG
MSSA2219	POS	POS	POS	NEG	NEG	NEG	NEG	POS	POS	POS	NEG	NEG	NEG	NEG	NEG
MSSA1753	NEG	NEG	NEG	NEG	NEG	NEG	NEG	NEG	NEG	NEG	NEG	NEG	NEG	NEG	NEG
MSSA1773	NEG	NEG	NEG	NEG	NEG	NEG	NEG	NEG	NEG	NEG	NEG	NEG	NEG	NEG	NEG
MSSA1811	NEG	NEG	NEG	NEG	NEG	NEG	NEG	NEG	NEG	NEG	NEG	NEG	NEG	NEG	NEG
MSSA1897	NEG	NEG	NEG	NEG	NEG	NEG	NEG	NEG	NEG	NEG	NEG	NEG	NEG	NEG	NEG
MSSA1931	NEG	NEG	NEG	NEG	NEG	NEG	NEG	NEG	NEG	NEG	NEG	NEG	NEG	NEG	NEG
MSSA2114	NEG	NEG	NEG	NEG	NEG	NEG	NEG	NEG	NEG	NEG	NEG	NEG	NEG	NEG	NEG
MSSA1745	NEG	NEG	NEG	NEG	NEG	NEG	NEG	NEG	NEG	NEG	NEG	NEG	NEG	NEG	NEG
MSSA1747	NEG	NEG	NEG	NEG	NEG	NEG	NEG	NEG	NEG	NEG	NEG	NEG	NEG	NEG	NEG
MSSA1749	NEG	NEG	NEG	NEG	NEG	NEG	NEG	NEG	NEG	NEG	NEG	NEG	NEG	NEG	NEG
MSSA1768	NEG	NEG	NEG	NEG	NEG	NEG	NEG	NEG	NEG	NEG	NEG	NEG	NEG	NEG	NEG
MSSA1774	NEG	NEG	NEG	NEG	NEG	NEG	NEG	NEG	NEG	NEG	NEG	NEG	NEG	NEG	NEG
MSSA1775	NEG	NEG	NEG	NEG	NEG	NEG	NEG	NEG	NEG	NEG	NEG	NEG	NEG	NEG	NEG
MSSA1776	NEG	NEG	NEG	NEG	AMB	NEG	NEG	NEG	NEG	NEG	NEG	NEG	NEG	NEG	NEG
MSSA1779	NEG	NEG	NEG	NEG	NEG	NEG	NEG	NEG	NEG	NEG	NEG	NEG	NEG	NEG	NEG
MSSA1780	NEG	NEG	NEG	NEG	NEG	NEG	NEG	NEG	NEG	NEG	NEG	NEG	NEG	NEG	NEG

	METHICILLIN RESISTANCE AND SCCmec TYPING														
Strain	mecA	mecR1 / delta mecR1	ugpQ	ccrA-1	ccrB-1 (probe 1)	ccrB-1 (probe 2)	plsSCC (COL)	Q9XB68-dcs	ccrA-2	ccrB-2	kdpA-SCC	kdpB-SCC	kdpC-SCC	kdpD-SCC	kdpE-SCC
~~MSSA1781~~	NEG	NEG	NEG	NEG	NEG	NEG	NEG	NEG	NEG	NEG	NEG	NEG	NEG	NEG	NEG
MSSA1786	NEG	NEG	NEG	NEG	NEG	NEG	NEG	NEG	NEG	NEG	NEG	NEG	NEG	NEG	NEG
MSSA1793	NEG	NEG	NEG	NEG	NEG	NEG	NEG	NEG	NEG	NEG	NEG	NEG	NEG	NEG	NEG
MSSA1803	NEG	NEG	NEG	NEG	NEG	NEG	NEG	NEG	NEG	NEG	NEG	NEG	NEG	NEG	NEG
MSSA1836	NEG	NEG	NEG	NEG	NEG	NEG	NEG	NEG	NEG	NEG	NEG	NEG	NEG	NEG	NEG
MSSA1839	NEG	NEG	NEG	NEG	NEG	NEG	NEG	NEG	NEG	NEG	NEG	NEG	NEG	NEG	NEG
MSSA1900	NEG	NEG	NEG	NEG	NEG	NEG	NEG	NEG	NEG	NEG	NEG	NEG	NEG	NEG	NEG
MSSA1910	NEG	NEG	NEG	NEG	NEG	NEG	NEG	NEG	NEG	NEG	NEG	NEG	NEG	NEG	NEG
MSSA1918	NEG	NEG	NEG	NEG	NEG	NEG	NEG	NEG	NEG	NEG	NEG	NEG	NEG	NEG	NEG
MSSA1921	NEG	NEG	NEG	NEG	NEG	NEG	NEG	NEG	NEG	NEG	NEG	NEG	NEG	NEG	NEG
MSSA1923	NEG	NEG	NEG	NEG	NEG	NEG	NEG	NEG	NEG	NEG	NEG	NEG	NEG	NEG	NEG
MSSA1981	NEG	NEG	NEG	NEG	NEG	NEG	NEG	NEG	NEG	NEG	NEG	NEG	NEG	NEG	NEG
MSSA1998	NEG	NEG	NEG	NEG	NEG	NEG	NEG	NEG	NEG	NEG	NEG	NEG	NEG	NEG	NEG
MSSA2000	NEG	NEG	NEG	NEG	NEG	NEG	NEG	NEG	NEG	NEG	NEG	NEG	NEG	NEG	NEG
MSSA2007	NEG	NEG	NEG	NEG	NEG	NEG	NEG	NEG	NEG	NEG	NEG	NEG	NEG	NEG	NEG
MSSA2012	NEG	NEG	NEG	NEG	NEG	NEG	NEG	NEG	NEG	NEG	NEG	NEG	NEG	NEG	NEG
MSSA2015	NEG	NEG	NEG	NEG	NEG	NEG	NEG	NEG	NEG	NEG	NEG	NEG	NEG	NEG	NEG
MSSA2021	NEG	NEG	NEG	NEG	NEG	NEG	NEG	NEG	NEG	NEG	NEG	NEG	NEG	NEG	NEG
MSSA2027	NEG	NEG	NEG	NEG	NEG	NEG	NEG	NEG	NEG	NEG	NEG	NEG	NEG	NEG	NEG
MSSA2063	NEG	NEG	NEG	NEG	NEG	NEG	NEG	NEG	NEG	NEG	NEG	NEG	NEG	NEG	NEG
MSSA2064	NEG	NEG	NEG	NEG	NEG	NEG	NEG	NEG	NEG	NEG	NEG	NEG	NEG	NEG	NEG
MSSA2065	NEG	NEG	NEG	NEG	NEG	NEG	NEG	NEG	NEG	NEG	NEG	NEG	NEG	NEG	NEG
MSSA2075	NEG	NEG	NEG	NEG	NEG	NEG	NEG	NEG	NEG	NEG	NEG	NEG	NEG	NEG	NEG
MSSA2093	NEG	NEG	NEG	NEG	NEG	NEG	NEG	NEG	NEG	NEG	NEG	NEG	NEG	NEG	NEG
MSSA2102	NEG	NEG	NEG	NEG	NEG	NEG	NEG	NEG	NEG	NEG	NEG	NEG	NEG	NEG	NEG
MSSA2138	NEG	NEG	NEG	NEG	NEG	NEG	NEG	NEG	NEG	NEG	NEG	NEG	NEG	NEG	NEG
MSSA2168	NEG	NEG	NEG	NEG	NEG	NEG	NEG	NEG	NEG	NEG	NEG	NEG	NEG	NEG	NEG
MSSA2181	NEG	NEG	NEG	NEG	NEG	NEG	NEG	NEG	NEG	NEG	NEG	NEG	NEG	NEG	NEG
MSSA2189	NEG	NEG	NEG	NEG	NEG	NEG	NEG	NEG	NEG	NEG	NEG	NEG	NEG	NEG	NEG
MSSA2214	NEG	NEG	NEG	NEG	NEG	NEG	NEG	NEG	NEG	NEG	NEG	NEG	NEG	NEG	NEG
MRSA3803	POS	POS	POS	NEG	NEG	NEG	NEG	POS	POS	POS	NEG	NEG	NEG	NEG	NEG
MSSA1750	NEG	NEG	NEG	NEG	POS	NEG	NEG	NEG	NEG	NEG	NEG	NEG	NEG	NEG	NEG
MSSA1756	NEG	NEG	NEG	NEG	POS	NEG	NEG	NEG	NEG	NEG	NEG	NEG	NEG	NEG	NEG
MSSA1759	NEG	NEG	NEG	NEG	AMB	NEG	NEG	NEG	NEG	NEG	NEG	NEG	NEG	NEG	NEG
MSSA1767	NEG	NEG	NEG	NEG	NEG	NEG	NEG	NEG	NEG	NEG	NEG	NEG	NEG	NEG	NEG
MSSA1787	NEG	NEG	NEG	NEG	NEG	NEG	NEG	NEG	NEG	NEG	NEG	NEG	NEG	NEG	NEG
MSSA1790	NEG	NEG	NEG	NEG	AMB	NEG	NEG	NEG	NEG	NEG	NEG	NEG	NEG	NEG	NEG
MSSA1794	NEG	NEG	NEG	NEG	NEG	NEG	NEG	NEG	NEG	NEG	NEG	NEG	NEG	NEG	NEG
MSSA1808	NEG	NEG	NEG	NEG	NEG	NEG	NEG	NEG	NEG	NEG	NEG	NEG	NEG	NEG	NEG
MSSA1812	NEG	NEG	NEG	NEG	NEG	NEG	NEG	NEG	NEG	NEG	NEG	NEG	NEG	NEG	NEG
MSSA1819	NEG	NEG	NEG	NEG	NEG	NEG	NEG	NEG	NEG	NEG	NEG	NEG	NEG	NEG	NEG
MSSA1824	NEG	NEG	NEG	NEG	NEG	NEG	NEG	NEG	NEG	NEG	NEG	NEG	NEG	NEG	NEG
MSSA1827	NEG	NEG	NEG	NEG	NEG	NEG	NEG	NEG	NEG	NEG	NEG	NEG	NEG	NEG	NEG
MSSA1889	NEG	NEG	NEG	NEG	NEG	NEG	NEG	NEG	NEG	NEG	NEG	NEG	NEG	NEG	NEG
MSSA1895	NEG	NEG	NEG	NEG	NEG	NEG	NEG	NEG	NEG	NEG	NEG	NEG	NEG	NEG	NEG
MSSA1904	NEG	NEG	NEG	NEG	NEG	NEG	NEG	NEG	NEG	NEG	NEG	NEG	NEG	NEG	NEG
MSSA1905	NEG	NEG	NEG	NEG	NEG	NEG	NEG	NEG	NEG	NEG	NEG	NEG	NEG	NEG	NEG
MSSA1907	NEG	NEG	NEG	NEG	NEG	NEG	NEG	NEG	NEG	NEG	NEG	NEG	NEG	NEG	NEG
MSSA1919	NEG	NEG	NEG	NEG	NEG	NEG	NEG	NEG	NEG	NEG	NEG	NEG	NEG	NEG	NEG
MSSA1920	NEG	NEG	NEG	NEG	NEG	NEG	NEG	NEG	NEG	NEG	NEG	NEG	NEG	NEG	NEG
MSSA1929	NEG	NEG	NEG	NEG	NEG	NEG	NEG	NEG	NEG	NEG	NEG	NEG	NEG	NEG	NEG
MSSA1930	NEG	NEG	NEG	NEG	NEG	NEG	NEG	NEG	NEG	NEG	NEG	NEG	NEG	NEG	NEG
MSSA1934	NEG	NEG	NEG	NEG	NEG	NEG	NEG	NEG	NEG	NEG	NEG	NEG	NEG	NEG	NEG
MSSA1946	NEG	NEG	NEG	NEG	NEG	NEG	NEG	NEG	NEG	NEG	NEG	NEG	NEG	NEG	NEG
MSSA1971	NEG	NEG	NEG	NEG	NEG	NEG	NEG	NEG	NEG	NEG	NEG	NEG	NEG	NEG	NEG
MSSA1980	NEG	NEG	NEG	NEG	NEG	NEG	NEG	NEG	NEG	NEG	NEG	NEG	NEG	NEG	NEG
MSSA1982	NEG	NEG	NEG	NEG	NEG	NEG	NEG	NEG	NEG	NEG	NEG	NEG	NEG	NEG	NEG
MSSA1996	NEG	NEG	NEG	NEG	NEG	NEG	NEG	NEG	NEG	NEG	NEG	NEG	NEG	NEG	NEG
MSSA2002	NEG	NEG	NEG	NEG	NEG	NEG	NEG	NEG	NEG	NEG	NEG	NEG	NEG	NEG	NEG
MSSA2014	NEG	NEG	NEG	NEG	NEG	NEG	NEG	NEG	NEG	NEG	NEG	NEG	NEG	NEG	NEG
MSSA2053	NEG	NEG	NEG	NEG	NEG	NEG	NEG	NEG	NEG	NEG	NEG	NEG	NEG	NEG	NEG
MSSA2054	NEG	NEG	NEG	NEG	NEG	NEG	NEG	NEG	NEG	NEG	NEG	NEG	NEG	NEG	NEG
MSSA2058	NEG	NEG	NEG	NEG	NEG	NEG	NEG	NEG	NEG	NEG	NEG	NEG	NEG	NEG	NEG
MSSA2068	NEG	NEG	NEG	NEG	AMB	NEG	NEG	NEG	NEG	NEG	NEG	NEG	NEG	NEG	NEG
MSSA2070	NEG	NEG	NEG	NEG	NEG	NEG	NEG	NEG	NEG	NEG	NEG	NEG	NEG	NEG	NEG
MSSA2072	NEG	NEG	NEG	NEG	NEG	NEG	NEG	NEG	NEG	NEG	NEG	NEG	NEG	NEG	NEG
MSSA2073	NEG	NEG	NEG	NEG	NEG	NEG	NEG	NEG	NEG	NEG	NEG	NEG	NEG	NEG	NEG
MSSA2074	NEG	NEG	NEG	NEG	NEG	NEG	NEG	NEG	NEG	NEG	NEG	NEG	NEG	NEG	NEG
MSSA2107	NEG	NEG	NEG	NEG	AMB	NEG	NEG	NEG	NEG	NEG	NEG	NEG	NEG	NEG	NEG
MSSA2108	NEG	NEG	NEG	NEG	NEG	NEG	NEG	NEG	NEG	NEG	NEG	NEG	NEG	NEG	NEG
MSSA2129	NEG	NEG	NEG	NEG	NEG	NEG	NEG	NEG	NEG	NEG	NEG	NEG	NEG	NEG	NEG
MSSA2135	NEG	NEG	NEG	NEG	NEG	NEG	NEG	NEG	NEG	NEG	NEG	NEG	NEG	NEG	NEG
MSSA2137	NEG	NEG	NEG	NEG	NEG	NEG	NEG	NEG	NEG	NEG	NEG	NEG	NEG	NEG	NEG
MSSA2140	NEG	NEG	NEG	NEG	NEG	NEG	NEG	NEG	NEG	NEG	NEG	NEG	NEG	NEG	NEG
MSSA2182	NEG	NEG	NEG	NEG	NEG	NEG	NEG	NEG	NEG	NEG	NEG	NEG	NEG	NEG	NEG
MSSA2188	NEG	NEG	NEG	NEG	NEG	NEG	NEG	NEG	NEG	NEG	NEG	NEG	NEG	NEG	NEG
MSSA1899	NEG	NEG	NEG	NEG	NEG	NEG	NEG	NEG	NEG	NEG	NEG	NEG	NEG	NEG	NEG
MSSA1915	NEG	NEG	NEG	NEG	NEG	NEG	NEG	NEG	NEG	NEG	NEG	NEG	NEG	NEG	NEG
MSSA1949	NEG	NEG	NEG	NEG	NEG	NEG	NEG	NEG	NEG	NEG	NEG	NEG	NEG	NEG	NEG
MSSA2042	NEG	NEG	NEG	NEG	NEG	NEG	NEG	NEG	NEG	NEG	NEG	NEG	NEG	NEG	NEG
MSSA2077	NEG	NEG	NEG	NEG	NEG	NEG	NEG	NEG	NEG	NEG	NEG	NEG	NEG	NEG	NEG
MSSA2095	NEG	NEG	NEG	NEG	AMB	NEG	NEG	NEG	NEG	NEG	NEG	NEG	NEG	NEG	NEG
MSSA1997	NEG	NEG	NEG	NEG	NEG	NEG	NEG	NEG	NEG	NEG	NEG	NEG	NEG	NEG	NEG
MSSA1810	NEG	NEG	NEG	NEG	NEG	NEG	NEG	NEG	NEG	NEG	NEG	NEG	NEG	NEG	NEG
MSSA1832	NEG	NEG	NEG	NEG	NEG	NEG	NEG	NEG	NEG	NEG	NEG	NEG	NEG	NEG	NEG
MSSA1891	NEG	NEG	NEG	NEG	NEG	NEG	NEG	NEG	NEG	NEG	NEG	NEG	NEG	NEG	NEG
MSSA1892	NEG	NEG	NEG	NEG	AMB	NEG	NEG	NEG	NEG	NEG	NEG	NEG	NEG	NEG	NEG
MSSA1932	NEG	NEG	NEG	NEG	NEG	NEG	NEG	NEG	NEG	NEG	NEG	NEG	NEG	NEG	NEG
MSSA1942	NEG	NEG	NEG	NEG	NEG	NEG	NEG	NEG	NEG	NEG	NEG	NEG	NEG	NEG	NEG
MSSA1957	NEG	NEG	NEG	NEG	NEG	NEG	NEG	NEG	NEG	NEG	NEG	NEG	NEG	NEG	NEG
MSSA1748	NEG	NEG	NEG	NEG	NEG	NEG	NEG	NEG	NEG	NEG	NEG	NEG	NEG	NEG	NEG
MSSA1754	NEG	NEG	NEG	NEG	NEG	NEG	NEG	NEG	NEG	NEG	NEG	NEG	NEG	NEG	NEG
MSSA1770	NEG	NEG	NEG	NEG	NEG	NEG	NEG	NEG	NEG	NEG	NEG	NEG	NEG	NEG	NEG
MSSA1788	NEG	NEG	NEG	NEG	NEG	NEG	NEG	NEG	NEG	NEG	NEG	NEG	NEG	NEG	NEG
MSSA1804	NEG	NEG	NEG	NEG	NEG	NEG	NEG	NEG	NEG	NEG	NEG	NEG	NEG	NEG	NEG
MSSA1818	NEG	NEG	NEG	NEG	NEG	NEG	NEG	NEG	NEG	NEG	NEG	NEG	NEG	NEG	NEG
MSSA1914	NEG	NEG	NEG	NEG	AMB	NEG	NEG	NEG	NEG	NEG	NEG	NEG	NEG	NEG	NEG
MSSA1974	NEG	NEG	NEG	NEG	NEG	NEG	NEG	NEG	NEG	NEG	NEG	NEG	NEG	NEG	NEG
MSSA2023	NEG	NEG	NEG	NEG	AMB	NEG	NEG	NEG	NEG	NEG	NEG	NEG	NEG	NEG	NEG
MSSA2030	NEG	NEG	NEG	NEG	NEG	NEG	NEG	NEG	NEG	NEG	NEG	NEG	NEG	NEG	NEG
MSSA2096	NEG	NEG	NEG	NEG	NEG	NEG	NEG	NEG	NEG	NEG	NEG	NEG	NEG	NEG	NEG
MSSA2142	NEG	NEG	NEG	NEG	AMB	NEG	NEG	NEG	NEG	NEG	NEG	NEG	NEG	NEG	NEG
MSSA2203	NEG	NEG	NEG	NEG	NEG	NEG	NEG	NEG	NEG	NEG	NEG	NEG	NEG	NEG	NEG
MSSA1901	NEG	NEG	NEG	NEG	NEG	NEG	NEG	NEG	NEG	NEG	NEG	NEG	NEG	NEG	NEG
MSSA2013	NEG	NEG	NEG	NEG	NEG	NEG	NEG	NEG	NEG	NEG	NEG	NEG	NEG	NEG	NEG
MSSA2031	NEG	NEG	NEG	NEG	NEG	NEG	NEG	NEG	NEG	NEG	NEG	NEG	NEG	NEG	NEG
MSSA2052	NEG	NEG	NEG	NEG	NEG	NEG	NEG	NEG	NEG	NEG	NEG	NEG	NEG	NEG	NEG
MSSA2057	NEG	NEG	NEG	NEG	NEG	NEG	NEG	NEG	NEG	NEG	NEG	NEG	NEG	NEG	NEG
MSSA2081	NEG	NEG	NEG	NEG	NEG	NEG	NEG	NEG	NEG	NEG	NEG	NEG	NEG	NEG	NEG
MSSA1955	NEG	NEG	NEG	NEG	NEG	NEG	NEG	NEG	NEG	NEG	NEG	NEG	NEG	NEG	NEG
MSSA1941	NEG	NEG	NEG	NEG	NEG	NEG	NEG	NEG	NEG	NEG	NEG	NEG	NEG	NEG	NEG
MRSA3838	POS	NEG	POS	NEG	NEG	NEG	NEG	NEG	NEG	NEG	NEG	NEG	NEG	NEG	NEG
MRSA3839	POS	NEG	POS	NEG	NEG	NEG	NEG	NEG	NEG	NEG	NEG	NEG	NEG	NEG	NEG
MSSA1801	NEG	NEG	NEG	NEG	NEG	NEG	NEG	NEG	NEG	NEG	NEG	NEG	NEG	NEG	NEG
MSSA2131	NEG	NEG	NEG	NEG	NEG	NEG	NEG	NEG	NEG	NEG	NEG	NEG	NEG	NEG	NEG
MSSA1792	NEG	NEG	NEG	NEG	NEG	NEG	NEG	NEG	NEG	NEG	NEG	NEG	NEG	NEG	NEG
MSSA1796	NEG	NEG	NEG	NEG	NEG	NEG	NEG	NEG	NEG	NEG	NEG	NEG	NEG	NEG	NEG
MSSA1817	NEG	NEG	NEG	NEG	NEG	NEG	NEG	NEG	NEG	NEG	NEG	NEG	NEG	NEG	NEG
MSSA1911	NEG	NEG	NEG	NEG	NEG	NEG	NEG	NEG	NEG	NEG	NEG	NEG	NEG	NEG	NEG
MSSA1927	NEG	NEG	NEG	NEG	NEG	NEG	NEG	NEG	NEG	NEG	NEG	NEG	NEG	NEG	NEG
MSSA1928	NEG	NEG	NEG	NEG	NEG	NEG	NEG	NEG	NEG	NEG	NEG	NEG	NEG	NEG	NEG
MSSA1953	NEG	NEG	NEG	NEG	NEG	NEG	NEG	NEG	NEG	NEG	NEG	NEG	NEG	NEG	NEG
MSSA2062	NEG	NEG	NEG	NEG	NEG	NEG	NEG	NEG	NEG	NEG	NEG	NEG	NEG	NEG	NEG
MSSA2078	NEG	NEG	NEG	NEG	NEG	NEG	NEG	NEG	NEG	NEG	NEG	NEG	NEG	NEG	NEG
MSSA2136	NEG	NEG	NEG	NEG	AMB	NEG	NEG	NEG	NEG	NEG	NEG	NEG	NEG	NEG	NEG

	METHICILLIN RESISTANCE AND SCCmec TYPING, continued												RESISTANCE: PENICILLINASE				
Strain	mecI	mecR	xylR	ccrA-3	ccrB-3	merA	merB	ccrAA (MRSAZH47 probe 1)	ccrAA (MRSAZH47 probe 2)	ccrC (85-2082)	ccrA-4	ccrB-4	blaZ (probe 1)	blaZ (probe 2)	blaI	blaR (probe 1)	blaR (probe 2)
MSSA1805	NEG	NEG	NEG	NEG	NEG	NEG	NEG	NEG	NEG	NEG	NEG	NEG	POS	POS	POS	POS	POS
MSSA1823	NEG	NEG	NEG	NEG	NEG	NEG	NEG	NEG	NEG	NEG	NEG	NEG	POS	POS	POS	POS	POS
MSSA1837	NEG	NEG	NEG	NEG	NEG	NEG	NEG	NEG	NEG	NEG	NEG	NEG	POS	POS	POS	POS	POS
MSSA2044	NEG	NEG	NEG	NEG	NEG	NEG	NEG	NEG	NEG	NEG	NEG	NEG	POS	POS	POS	POS	POS
MSSA2046	NEG	NEG	NEG	NEG	NEG	NEG	NEG	NEG	NEG	NEG	NEG	NEG	POS	POS	POS	POS	POS
MSSA2059	NEG	NEG	NEG	NEG	NEG	NEG	NEG	NEG	NEG	NEG	NEG	NEG	POS	POS	POS	POS	POS
MSSA2076	NEG	NEG	NEG	NEG	NEG	NEG	NEG	NEG	NEG	NEG	NEG	NEG	POS	POS	POS	POS	POS
MSSA2200	NEG	NEG	NEG	NEG	NEG	NEG	NEG	NEG	NEG	NEG	NEG	NEG	POS	POS	POS	POS	POS
MRSA3797	POS	POS	POS	NEG	NEG	NEG	NEG	NEG	NEG	NEG	NEG	NEG	POS	POS	POS	POS	POS
MRSA3798	POS	POS	POS	NEG	NEG	NEG	NEG	NEG	NEG	NEG	NEG	NEG	POS	POS	POS	POS	POS
MRSA3799	POS	POS	POS	NEG	NEG	NEG	NEG	NEG	NEG	NEG	NEG	NEG	NEG	NEG	NEG	NEG	NEG
MRSA3800	POS	POS	POS	NEG	NEG	NEG	NEG	NEG	NEG	NEG	NEG	NEG	POS	POS	POS	POS	POS
MRSA3801	POS	POS	POS	NEG	NEG	NEG	NEG	NEG	NEG	NEG	NEG	NEG	POS	POS	POS	POS	POS
MRSA3804	POS	POS	POS	NEG	NEG	NEG	NEG	NEG	NEG	NEG	NEG	NEG	POS	POS	POS	POS	POS
MRSA3806	POS	POS	POS	NEG	NEG	NEG	NEG	NEG	NEG	NEG	NEG	NEG	POS	POS	POS	POS	POS
MRSA3961	POS	POS	POS	NEG	NEG	NEG	NEG	NEG	NEG	NEG	NEG	NEG	POS	POS	POS	POS	POS
MRSA3962	NEG	NEG	NEG	NEG	NEG	NEG	NEG	NEG	NEG	NEG	NEG	NEG	POS	POS	POS	POS	POS
MRSA3963	POS	POS	POS	NEG	NEG	NEG	NEG	NEG	NEG	NEG	NEG	NEG	NEG	NEG	NEG	NEG	NEG
MRSA3966	POS	POS	POS	NEG	NEG	NEG	NEG	NEG	NEG	NEG	NEG	NEG	NEG	NEG	NEG	NEG	NEG
MRSA3978	POS	POS	POS	NEG	NEG	NEG	NEG	NEG	NEG	NEG	NEG	NEG	POS	POS	POS	POS	POS
MRSA3979	POS	POS	POS	NEG	NEG	NEG	NEG	NEG	NEG	NEG	NEG	NEG	POS	POS	POS	POS	POS
MRSA3980	POS	POS	POS	NEG	NEG	NEG	NEG	NEG	NEG	NEG	NEG	NEG	POS	POS	POS	POS	POS
MRSA4179	POS	POS	POS	NEG	NEG	NEG	NEG	NEG	NEG	NEG	NEG	NEG	NEG	NEG	NEG	NEG	NEG
MRSA4216	POS	POS	POS	NEG	NEG	NEG	NEG	NEG	NEG	NEG	NEG	NEG	POS	POS	POS	POS	POS
MRSA4217	POS	POS	POS	NEG	NEG	NEG	NEG	NEG	NEG	NEG	NEG	NEG	POS	POS	POS	POS	POS
MRSA4220	POS	POS	POS	NEG	NEG	NEG	NEG	NEG	NEG	NEG	NEG	NEG	NEG	NEG	NEG	NEG	NEG
MRSA4221	POS	POS	POS	NEG	NEG	NEG	NEG	NEG	NEG	NEG	NEG	NEG	POS	POS	POS	POS	POS
MRSA4222	POS	POS	POS	NEG	NEG	NEG	NEG	NEG	NEG	NEG	NEG	NEG	POS	POS	POS	POS	POS
MSSA1751	NEG	NEG	AMB	NEG	NEG	NEG	NEG	NEG	NEG	NEG	NEG	NEG	POS	POS	POS	POS	POS
MSSA1760	NEG	NEG	NEG	NEG	NEG	NEG	NEG	NEG	NEG	NEG	NEG	NEG	POS	POS	POS	POS	POS
MSSA1802	NEG	NEG	NEG	NEG	NEG	NEG	NEG	NEG	NEG	NEG	NEG	NEG	POS	POS	POS	POS	POS
MSSA1809	NEG	NEG	NEG	NEG	NEG	NEG	NEG	NEG	NEG	NEG	NEG	NEG	POS	POS	POS	POS	POS
MSSA1815	NEG	NEG	NEG	NEG	NEG	NEG	NEG	NEG	NEG	NEG	NEG	NEG	NEG	NEG	NEG	NEG	NEG
MSSA1829	NEG	NEG	NEG	NEG	NEG	NEG	NEG	NEG	NEG	NEG	NEG	NEG	NEG	NEG	NEG	NEG	NEG
MSSA1908	NEG	NEG	NEG	NEG	NEG	NEG	NEG	NEG	NEG	NEG	NEG	NEG	POS	POS	POS	POS	POS
MSSA1960	NEG	NEG	NEG	NEG	NEG	NEG	NEG	NEG	NEG	NEG	NEG	NEG	NEG	NEG	NEG	NEG	NEG
MSSA1965	NEG	NEG	NEG	NEG	NEG	NEG	NEG	NEG	NEG	NEG	NEG	NEG	NEG	NEG	NEG	NEG	NEG
MSSA1977	NEG	NEG	NEG	NEG	NEG	NEG	NEG	NEG	NEG	NEG	NEG	NEG	POS	POS	POS	POS	POS
MSSA1978	NEG	NEG	NEG	NEG	NEG	NEG	NEG	NEG	NEG	NEG	NEG	NEG	NEG	NEG	NEG	NEG	NEG
MSSA1999	NEG	NEG	NEG	NEG	NEG	NEG	NEG	NEG	NEG	NEG	NEG	NEG	NEG	NEG	NEG	NEG	NEG
MSSA2003	NEG	NEG	NEG	NEG	NEG	NEG	NEG	NEG	NEG	NEG	NEG	NEG	NEG	NEG	NEG	NEG	NEG
MSSA2006	NEG	NEG	NEG	NEG	NEG	NEG	NEG	NEG	NEG	NEG	NEG	NEG	NEG	NEG	NEG	NEG	NEG
MSSA2008	NEG	NEG	NEG	NEG	NEG	NEG	NEG	NEG	NEG	NEG	NEG	NEG	NEG	NEG	NEG	NEG	NEG
MSSA2011	POS	POS	POS	NEG	NEG	NEG	NEG	NEG	NEG	NEG	NEG	NEG	POS	POS	POS	POS	POS
MSSA2017	NEG	NEG	NEG	NEG	NEG	NEG	NEG	NEG	NEG	NEG	NEG	NEG	POS	POS	POS	POS	POS
MSSA2022	POS	POS	POS	NEG	NEG	NEG	NEG	NEG	NEG	NEG	NEG	NEG	POS	POS	POS	POS	POS
MSSA2055	NEG	NEG	NEG	NEG	NEG	NEG	NEG	NEG	NEG	NEG	NEG	NEG	NEG	NEG	NEG	NEG	AMB
MSSA2060	NEG	NEG	NEG	NEG	NEG	NEG	NEG	NEG	NEG	NEG	NEG	NEG	NEG	NEG	NEG	NEG	NEG
MSSA2080	NEG	NEG	NEG	NEG	NEG	NEG	NEG	NEG	NEG	NEG	NEG	NEG	NEG	NEG	NEG	NEG	NEG
MSSA2082	NEG	NEG	NEG	NEG	NEG	NEG	NEG	NEG	NEG	NEG	NEG	NEG	POS	POS	POS	POS	POS
MSSA2084	NEG	NEG	NEG	NEG	NEG	NEG	NEG	NEG	NEG	NEG	NEG	NEG	NEG	NEG	NEG	NEG	NEG
MSSA2087	NEG	NEG	NEG	NEG	NEG	NEG	NEG	NEG	NEG	NEG	NEG	NEG	POS	POS	POS	POS	POS
MSSA2094	NEG	NEG	NEG	NEG	NEG	NEG	NEG	NEG	NEG	NEG	NEG	NEG	NEG	NEG	NEG	NEG	NEG
MSSA2097	NEG	NEG	NEG	NEG	NEG	NEG	NEG	NEG	NEG	NEG	NEG	NEG	NEG	NEG	NEG	NEG	NEG
MSSA2110	NEG	NEG	NEG	NEG	NEG	NEG	NEG	NEG	NEG	NEG	NEG	NEG	NEG	NEG	NEG	NEG	NEG
MSSA2113	NEG	NEG	NEG	NEG	NEG	NEG	NEG	NEG	NEG	NEG	NEG	NEG	POS	POS	POS	POS	POS
MSSA2132	NEG	NEG	NEG	NEG	NEG	NEG	NEG	NEG	NEG	NEG	NEG	NEG	NEG	NEG	NEG	NEG	NEG
MSSA2134	NEG	NEG	NEG	NEG	NEG	NEG	NEG	NEG	NEG	NEG	NEG	NEG	POS	POS	POS	POS	AMB
MSSA1746	NEG	NEG	NEG	NEG	NEG	NEG	NEG	NEG	NEG	NEG	NEG	NEG	POS	POS	POS	POS	POS
MSSA1757	NEG	NEG	NEG	NEG	NEG	NEG	NEG	NEG	NEG	NEG	NEG	NEG	POS	POS	POS	POS	POS
MSSA1772	NEG	NEG	NEG	NEG	NEG	NEG	NEG	NEG	NEG	NEG	NEG	NEG	POS	POS	POS	POS	POS
MSSA1795	NEG	NEG	NEG	NEG	NEG	NEG	NEG	NEG	NEG	NEG	NEG	NEG	POS	POS	POS	POS	POS
MSSA1813	NEG	NEG	NEG	NEG	NEG	NEG	NEG	NEG	NEG	NEG	NEG	NEG	POS	POS	POS	POS	POS
MSSA1826	NEG	NEG	NEG	NEG	NEG	NEG	NEG	NEG	NEG	NEG	NEG	NEG	NEG	NEG	NEG	NEG	NEG
MSSA1830	NEG	NEG	NEG	NEG	NEG	NEG	NEG	NEG	NEG	NEG	NEG	NEG	NEG	NEG	NEG	NEG	NEG
MSSA1838	NEG	NEG	NEG	NEG	NEG	NEG	NEG	NEG	NEG	NEG	NEG	NEG	POS	POS	POS	POS	POS
MSSA1944	NEG	NEG	NEG	NEG	NEG	NEG	NEG	NEG	NEG	NEG	NEG	NEG	POS	POS	POS	POS	POS
MSSA1947	NEG	NEG	NEG	NEG	NEG	NEG	NEG	NEG	NEG	NEG	NEG	NEG	POS	POS	POS	POS	POS
MSSA1964	NEG	NEG	NEG	NEG	NEG	NEG	NEG	NEG	NEG	NEG	NEG	NEG	NEG	NEG	NEG	NEG	NEG
MSSA1969	NEG	NEG	NEG	NEG	NEG	NEG	NEG	NEG	NEG	NEG	NEG	NEG	POS	POS	POS	POS	POS
MSSA2001	NEG	NEG	NEG	NEG	NEG	NEG	NEG	NEG	NEG	NEG	NEG	NEG	POS	POS	POS	POS	POS
MSSA2004	NEG	NEG	NEG	NEG	NEG	NEG	NEG	NEG	NEG	NEG	NEG	NEG	POS	POS	POS	POS	POS
MSSA2024	NEG	NEG	NEG	NEG	NEG	NEG	NEG	NEG	NEG	NEG	NEG	NEG	POS	POS	POS	POS	POS
MSSA2025	NEG	NEG	NEG	NEG	NEG	NEG	NEG	NEG	NEG	NEG	NEG	NEG	POS	POS	POS	POS	POS
MSSA2026	NEG	NEG	NEG	NEG	NEG	NEG	NEG	NEG	NEG	NEG	NEG	NEG	POS	POS	POS	POS	POS
MSSA2032	NEG	NEG	NEG	NEG	NEG	NEG	NEG	NEG	NEG	NEG	NEG	NEG	POS	POS	POS	POS	POS
MSSA2043	NEG	NEG	NEG	NEG	NEG	NEG	NEG	NEG	NEG	NEG	NEG	NEG	NEG	NEG	NEG	NEG	NEG
MSSA2061	NEG	NEG	NEG	NEG	NEG	NEG	NEG	NEG	NEG	NEG	NEG	NEG	POS	POS	POS	POS	POS
MSSA2085	NEG	NEG	NEG	NEG	NEG	NEG	NEG	NEG	NEG	NEG	NEG	NEG	POS	POS	POS	POS	POS
MSSA2098	NEG	NEG	NEG	NEG	NEG	NEG	NEG	NEG	NEG	NEG	NEG	NEG	POS	POS	POS	POS	POS
MSSA2100	NEG	NEG	NEG	NEG	NEG	NEG	NEG	NEG	NEG	NEG	NEG	NEG	NEG	NEG	NEG	NEG	NEG
MSSA2111	NEG	NEG	NEG	NEG	NEG	NEG	NEG	NEG	NEG	NEG	NEG	NEG	POS	POS	POS	POS	POS
MSSA2178	NEG	NEG	NEG	NEG	NEG	NEG	NEG	NEG	NEG	NEG	NEG	NEG	POS	POS	POS	POS	POS
MSSA2202	NEG	NEG	NEG	NEG	NEG	NEG	NEG	NEG	NEG	NEG	NEG	NEG	POS	POS	POS	POS	POS
MSSA2205	NEG	NEG	NEG	NEG	NEG	NEG	NEG	NEG	NEG	NEG	NEG	NEG	POS	POS	POS	POS	POS
MRSA3796	NEG	NEG	AMB	NEG	AMB	POS	POS	NEG	NEG	NEG	NEG	NEG	POS	POS	POS	POS	POS
MRSA3805	POS	POS	POS	POS	POS	NEG	NEG	NEG	NEG	POS	NEG	NEG	POS	POS	POS	POS	POS
MRSA3965	NEG	NEG	NEG	NEG	NEG	POS	POS	NEG	NEG	NEG	NEG	NEG	POS	POS	POS	POS	POS
MSSA1761	NEG	NEG	POS	NEG	NEG	NEG	NEG	NEG	NEG	NEG	NEG	NEG	POS	POS	POS	POS	POS
MSSA1764	NEG	NEG	NEG	NEG	NEG	NEG	NEG	NEG	NEG	NEG	NEG	NEG	POS	POS	POS	POS	POS
MSSA1765	NEG	NEG	NEG	NEG	NEG	NEG	NEG	NEG	NEG	NEG	NEG	NEG	NEG	NEG	NEG	NEG	NEG
MSSA1784	NEG	NEG	AMB	NEG	NEG	NEG	NEG	NEG	NEG	NEG	NEG	NEG	NEG	NEG	NEG	NEG	NEG
MSSA1806	NEG	NEG	NEG	NEG	NEG	NEG	NEG	NEG	NEG	NEG	NEG	NEG	POS	POS	POS	POS	POS
MSSA1807	NEG	NEG	NEG	NEG	NEG	NEG	NEG	NEG	NEG	NEG	NEG	NEG	NEG	NEG	NEG	NEG	NEG
MSSA1888	NEG	NEG	NEG	NEG	NEG	NEG	NEG	NEG	NEG	NEG	NEG	NEG	POS	POS	POS	POS	POS
MSSA1894	NEG	NEG	NEG	NEG	NEG	NEG	NEG	NEG	NEG	NEG	NEG	NEG	NEG	NEG	NEG	NEG	NEG
MSSA1922	NEG	NEG	NEG	NEG	NEG	NEG	NEG	NEG	NEG	NEG	NEG	NEG	POS	POS	POS	POS	POS
MSSA1950	NEG	NEG	NEG	NEG	NEG	NEG	NEG	NEG	NEG	NEG	NEG	NEG	POS	POS	POS	POS	POS
MSSA1954	NEG	NEG	NEG	NEG	NEG	NEG	NEG	NEG	NEG	NEG	NEG	NEG	POS	POS	POS	POS	POS
MSSA1966	NEG	NEG	NEG	NEG	NEG	NEG	NEG	NEG	NEG	NEG	NEG	NEG	POS	POS	POS	POS	POS
MSSA1972	NEG	NEG	NEG	NEG	NEG	NEG	NEG	NEG	NEG	NEG	NEG	NEG	NEG	NEG	NEG	NEG	NEG
MSSA2010	NEG	NEG	NEG	NEG	NEG	NEG	NEG	NEG	NEG	NEG	NEG	NEG	POS	POS	POS	POS	POS
MSSA2020	NEG	NEG	NEG	NEG	NEG	NEG	NEG	NEG	NEG	NEG	NEG	NEG	POS	POS	POS	POS	POS
MSSA2028	NEG	NEG	AMB	NEG	NEG	NEG	NEG	NEG	NEG	NEG	NEG	NEG	NEG	NEG	NEG	NEG	NEG
MSSA2088	NEG	NEG	NEG	NEG	NEG	NEG	NEG	NEG	NEG	NEG	NEG	NEG	NEG	NEG	NEG	NEG	NEG
MSSA2187	NEG	NEG	NEG	NEG	NEG	NEG	NEG	NEG	AMB	NEG	NEG	NEG	POS	POS	POS	POS	POS
MSSA2204	NEG	NEG	AMB	NEG	NEG	NEG	NEG	NEG	NEG	NEG	NEG	NEG	POS	POS	POS	POS	POS
MSSA2210	NEG	NEG	NEG	NEG	NEG	NEG	NEG	NEG	NEG	NEG	NEG	NEG	NEG	NEG	NEG	NEG	NEG
MSSA2223	NEG	NEG	NEG	NEG	NEG	NEG	NEG	NEG	NEG	NEG	NEG	NEG	POS	POS	POS	POS	POS
MSSA1763	NEG	NEG	NEG	NEG	NEG	NEG	NEG	NEG	NEG	NEG	NEG	NEG	POS	POS	POS	POS	NEG
MSSA1777	NEG	NEG	AMB	NEG	NEG	NEG	NEG	NEG	NEG	NEG	NEG	NEG	POS	POS	POS	POS	POS
MSSA2009	NEG	NEG	NEG	NEG	NEG	NEG	NEG	NEG	NEG	NEG	NEG	NEG	POS	POS	POS	POS	POS
MSSA2047	NEG	NEG	NEG	NEG	NEG	NEG	NEG	NEG	NEG	NEG	NEG	NEG	POS	POS	POS	POS	POS
MSSA2079	NEG	NEG	NEG	NEG	NEG	NEG	NEG	NEG	NEG	NEG	NEG	NEG	POS	POS	POS	POS	AMB
MSSA2101	NEG	NEG	NEG	NEG	NEG	NEG	NEG	NEG	NEG	NEG	NEG	NEG	POS	POS	POS	POS	POS
MSSA1821	NEG	NEG	NEG	NEG	NEG	NEG	NEG	NEG	NEG	NEG	NEG	NEG	NEG	NEG	NEG	NEG	NEG
MSSA1898	NEG	NEG	NEG	NEG	NEG	NEG	NEG	NEG	NEG	NEG	NEG	NEG	NEG	NEG	NEG	NEG	NEG
MSSA1973	NEG	NEG	NEG	NEG	NEG	NEG	NEG	NEG	NEG	NEG	NEG	NEG	POS	POS	POS	POS	POS
MSSA2071	NEG	NEG	NEG	NEG	NEG	NEG	NEG	NEG	NEG	NEG	NEG	NEG	POS	POS	POS	POS	POS
MSSA2083	NEG	NEG	NEG	NEG	NEG	NEG	NEG	NEG	NEG	NEG	NEG	NEG	NEG	NEG	NEG	NEG	NEG
MSSA2106	NEG	NEG	NEG	NEG	NEG	NEG	NEG	NEG	NEG	NEG	NEG	NEG	POS	POS	POS	POS	POS
MSSA2112	NEG	NEG	NEG	NEG	NEG	NEG	NEG	NEG	NEG	NEG	NEG	NEG	NEG	NEG	NEG	NEG	NEG
MSSA2115	NEG	NEG	NEG	NEG	NEG	NEG	NEG	NEG	NEG	NEG	NEG	NEG	NEG	NEG	NEG	NEG	NEG
MSSA2195	NEG	NEG	NEG	NEG	NEG	NEG	NEG	NEG	NEG	NEG	NEG	NEG	NEG	NEG	NEG	NEG	NEG
MSSA2207	NEG	NEG	NEG	NEG	NEG	NEG	NEG	NEG	NEG	NEG	NEG	NEG	NEG	NEG	NEG	NEG	NEG
MSSA1758	NEG	NEG	NEG	NEG	NEG	NEG	NEG	NEG	NEG	NEG	NEG	NEG	POS	POS	POS	POS	POS
MSSA1762	NEG	NEG	NEG	NEG	NEG	NEG	NEG	NEG	NEG	NEG	NEG	NEG	POS	POS	POS	POS	POS
MSSA1769	NEG	NEG	NEG	NEG	NEG	NEG	NEG	NEG	NEG	NEG	NEG	NEG	POS	POS	POS	POS	POS
MSSA1771	NEG	NEG	NEG	NEG	NEG	NEG	NEG	NEG	NEG	NEG	NEG	NEG	POS	POS	POS	POS	POS
MSSA1789	NEG	NEG	NEG	NEG	NEG	NEG	NEG	NEG	NEG	NEG	NEG	NEG	POS	POS	POS	POS	POS
MSSA1800	NEG	NEG	NEG	NEG	NEG	NEG	NEG	NEG	NEG	NEG	NEG	NEG	POS	POS	POS	POS	POS
MSSA1833	NEG	NEG	NEG	NEG	NEG	NEG	NEG	NEG	NEG	NEG	NEG	NEG	POS	POS	POS	POS	POS
MSSA1890	NEG	NEG	NEG	NEG	NEG	NEG	NEG	NEG	NEG	NEG	NEG	NEG	POS	POS	POS	POS	POS
MSSA1903	NEG	NEG	NEG	NEG	NEG	NEG	NEG	NEG	NEG	NEG	NEG	NEG	POS	POS	POS	POS	POS
MSSA1940	NEG	NEG	NEG	NEG	NEG	NEG	NEG	NEG	NEG	NEG	NEG	NEG	POS	POS	POS	POS	POS
MSSA1962	NEG	NEG	NEG	NEG	NEG	NEG	NEG	NEG	NEG	NEG	NEG	NEG	NEG	NEG	NEG	NEG	NEG
MSSA1963	NEG	NEG	NEG	NEG	NEG	NEG	NEG	NEG	NEG	NEG	NEG	NEG	NEG	NEG	NEG	NEG	NEG
MSSA2016	NEG	NEG	NEG	NEG	NEG	NEG	NEG	NEG	NEG	NEG	NEG	NEG	POS	POS	POS	POS	POS
MSSA2018	NEG	NEG	NEG	NEG	NEG	NEG	NEG	NEG	NEG	NEG	NEG	NEG	POS	POS	POS	POS	POS
MSSA2019	NEG	NEG	NEG	NEG	NEG	NEG	NEG	NEG	NEG	NEG	NEG	NEG	POS	POS	POS	AMB	POS
MSSA2029	NEG	NEG	NEG	NEG	NEG	NEG	NEG	NEG	NEG	NEG	NEG	NEG	POS	POS	POS	POS	POS
MSSA2045	NEG	NEG	NEG	NEG	NEG	NEG	NEG	NEG	NEG	NEG	NEG	NEG	POS	POS	POS	POS	POS
MSSA2048	NEG	NEG	NEG	NEG	NEG	NEG	NEG	NEG	NEG	NEG	NEG	NEG	NEG	NEG	NEG	NEG	NEG
MSSA2049	NEG	NEG	NEG	NEG	NEG	NEG	NEG	NEG	NEG	NEG	NEG	NEG	POS	POS	POS	POS	POS
MSSA2050	NEG	NEG	NEG	NEG	NEG	NEG	NEG	NEG	NEG	NEG	NEG	NEG	POS	POS	POS	POS	POS
MSSA2099	NEG	NEG	NEG	NEG	NEG	NEG	NEG	NEG	NEG	NEG	NEG	NEG	POS	POS	POS	POS	POS
MSSA2109	NEG	NEG	NEG	NEG	NEG	NEG	NEG	NEG	NEG	NEG	NEG	NEG	POS	POS	POS	POS	POS
MSSA2130	NEG	NEG	NEG	NEG	NEG	NEG	NEG	NEG	NEG	NEG	NEG	NEG	NEG	NEG	NEG	NEG	NEG
MSSA2133	NEG	NEG	NEG	NEG	NEG	NEG	NEG	NEG	NEG	NEG	NEG	NEG	POS	POS	POS	POS	POS
MSSA2139	NEG	NEG	NEG	NEG	NEG	NEG	NEG	NEG	NEG	NEG	NEG	NEG	NEG	NEG	NEG	NEG	NEG
MSSA2141	NEG	NEG	NEG	NEG	NEG	NEG	NEG	NEG	NEG	NEG	NEG	NEG	POS	POS	POS	POS	POS
MSSA2208	NEG	NEG	NEG	NEG	NEG	NEG	NEG	NEG	NEG	NEG	NEG	NEG	POS	POS	POS	POS	POS
MSSA2209	NEG	NEG	NEG	NEG	NEG	NEG	NEG	NEG	NEG	NEG	NEG	NEG	POS	POS	POS	POS	POS
MSSA1893	NEG	NEG	NEG	NEG	NEG	NEG	NEG	NEG	NEG	NEG	NEG	NEG	POS	POS	POS	POS	POS
MSSA2105	NEG	NEG	NEG	NEG	NEG	NEG	NEG	NEG	NEG	NEG	NEG	NEG	POS	POS	POS	AMB	POS
MRSA3836	NEG	NEG	NEG	NEG	NEG	NEG	NEG	NEG	NEG	NEG	NEG	NEG	POS	POS	POS	POS	POS
MRSA3840	NEG	NEG	NEG	NEG	NEG	NEG	NEG	NEG	NEG	NEG	NEG	NEG	POS	POS	POS	POS	POS
MRSA4188	NEG	NEG	NEG	NEG	NEG	NEG	NEG	NEG	NEG	NEG	NEG	NEG	POS	POS	POS	POS	POS
MSSA1814	NEG	NEG	NEG	NEG	NEG	NEG	NEG	NEG	NEG	NEG	NEG	NEG	NEG	NEG	NEG	NEG	NEG
MSSA1816	NEG	NEG	NEG	NEG	NEG	NEG	NEG	NEG	NEG	NEG	NEG	POS	POS	POS	POS	POS	NEG
MSSA1825	NEG	NEG	NEG	NEG	NEG	NEG	NEG	NEG	NEG	NEG	NEG	NEG	NEG	NEG	NEG	NEG	NEG
MSSA1834	NEG	NEG	NEG	NEG	NEG	NEG	NEG	NEG	NEG	NEG	NEG	POS	POS	POS	POS	POS	NEG
MSSA1938	NEG	NEG	NEG	NEG	NEG	NEG	NEG	NEG	NEG	NEG	NEG	NEG	POS	POS	POS	POS	POS
MSSA1948	NEG	NEG	NEG	NEG	NEG	NEG	NEG	NEG	NEG	NEG	NEG	NEG	POS	POS	POS	POS	POS
MSSA1951	NEG	NEG	NEG	NEG	NEG	NEG	NEG	NEG	NEG	NEG	NEG	NEG	POS	POS	POS	POS	AMB
MSSA2005	NEG	NEG	NEG	NEG	NEG	NEG	NEG	NEG	NEG	NEG	NEG	NEG	POS	POS	POS	POS	POS
MSSA2051	NEG	NEG	NEG	NEG	NEG	NEG	NEG	NEG	NEG	NEG	NEG	NEG	POS	POS	POS	POS	POS
MSSA2056	NEG	NEG	NEG	NEG	NEG	NEG	NEG	NEG	NEG	NEG	NEG	NEG	POS	POS	POS	POS	AMB
MSSA2066	NEG	NEG	NEG	NEG	NEG	NEG	NEG	NEG	NEG	NEG	NEG	NEG	POS	POS	POS	POS	POS
MSSA2067	NEG	NEG	NEG	NEG	NEG	NEG	NEG	NEG	NEG	NEG	NEG	NEG	NEG	NEG	NEG	NEG	NEG
MSSA2086	NEG	NEG	NEG	NEG	NEG	NEG	NEG	NEG	NEG	NEG	NEG	NEG	POS	POS	POS	POS	POS
MSSA2089	NEG	NEG	NEG	NEG	NEG	NEG	NEG	NEG	NEG	NEG	NEG	NEG	POS	POS	POS	POS	AMB
MSSA2103	NEG	NEG	NEG	NEG	NEG	NEG	NEG	NEG	NEG	NEG	NEG	NEG	POS	POS	POS	POS	POS
MSSA2173	NEG	NEG	NEG	NEG	NEG	NEG	NEG	NEG	NEG	NEG	AMB	POS	POS	POS	POS	POS	POS
MSSA2183	NEG	NEG	NEG	NEG	NEG	NEG	NEG	NEG	NEG	NEG	NEG	NEG	NEG	NEG	NEG	NEG	NEG
MSSA2196	NEG	NEG	NEG	NEG	NEG	NEG	NEG	NEG	NEG	NEG	NEG	NEG	POS	POS	POS	POS	POS
MSSA2219	NEG	NEG	NEG	NEG	NEG	NEG	NEG	NEG	NEG	NEG	NEG	NEG	POS	POS	POS	POS	POS
MSSA1753	NEG	NEG	NEG	NEG	NEG	NEG	NEG	NEG	NEG	NEG	NEG	NEG	POS	POS	POS	POS	POS
MSSA1773	NEG	NEG	NEG	NEG	NEG	NEG	NEG	NEG	NEG	NEG	NEG	NEG	POS	POS	POS	POS	POS
MSSA1811	NEG	NEG	NEG	NEG	NEG	NEG	NEG	NEG	NEG	NEG	NEG	NEG	POS	POS	POS	POS	POS
MSSA1897	NEG	NEG	NEG	NEG	NEG	NEG	NEG	NEG	NEG	NEG	NEG	NEG	NEG	NEG	NEG	NEG	NEG
MSSA1931	NEG	NEG	NEG	NEG	NEG	NEG	NEG	NEG	NEG	NEG	NEG	NEG	POS	POS	POS	POS	POS
MSSA2114	NEG	NEG	NEG	NEG	NEG	NEG	NEG	NEG	NEG	NEG	NEG	NEG	POS	POS	POS	POS	POS
MSSA1745	NEG	NEG	NEG	NEG	NEG	NEG	NEG	NEG	NEG	NEG	NEG	NEG	POS	POS	POS	POS	POS
MSSA1747	NEG	NEG	NEG	NEG	NEG	NEG	NEG	NEG	NEG	NEG	NEG	NEG	POS	POS	POS	POS	POS
MSSA1749	NEG	NEG	NEG	NEG	NEG	NEG	NEG	NEG	NEG	NEG	NEG	NEG	POS	POS	POS	POS	POS
MSSA1768	NEG	NEG	NEG	NEG	NEG	NEG	NEG	NEG	NEG	NEG	NEG	NEG	POS	POS	POS	POS	POS
MSSA1774	NEG	NEG	NEG	NEG	NEG	NEG	NEG	NEG	NEG	NEG	NEG	NEG	POS	POS	POS	POS	POS
MSSA1775	NEG	NEG	NEG	NEG	NEG	NEG	NEG	NEG	NEG	NEG	NEG	NEG	POS	POS	POS	POS	POS
MSSA1776	NEG	NEG	NEG	NEG	NEG	NEG	NEG	NEG	NEG	NEG	NEG	NEG	POS	POS	POS	POS	POS
MSSA1779	NEG	NEG	NEG	NEG	NEG	NEG	NEG	NEG	NEG	NEG	NEG	NEG	POS	POS	POS	POS	POS
MSSA1780	NEG	NEG	NEG	NEG	NEG	NEG	NEG	NEG	NEG	NEG	NEG	NEG	POS	POS	POS	POS	POS

	METHICILLIN RESISTANCE AND SCCmec TYPING, continued												RESISTANCE: PENICILLINASE				
Strain	mecI	mecR	xylR	ccrA-3	ccrB-3	merA	merB	ccrAA (MRSAZH47 probe 1)	ccrAA (MRSAZH47 probe 2)	ccrC (85-2082)	ccrA-4	ccrB-4	blaZ (probe 1)	blaZ (probe 2)	blaI	blaR (probe 1)	blaR (probe 2)
MSSA1781	NEG	NEG	NEG	NEG	NEG	NEG	NEG	NEG	NEG	NEG	NEG	NEG	POS	POS	POS	POS	POS
MSSA1786	NEG	NEG	NEG	NEG	NEG	NEG	NEG	NEG	NEG	NEG	NEG	NEG	POS	POS	POS	POS	POS
MSSA1793	NEG	NEG	NEG	NEG	NEG	NEG	NEG	NEG	NEG	NEG	NEG	NEG	POS	POS	POS	POS	POS
MSSA1803	NEG	NEG	NEG	NEG	NEG	NEG	NEG	NEG	NEG	NEG	NEG	NEG	POS	POS	POS	POS	POS
MSSA1836	NEG	NEG	NEG	NEG	NEG	NEG	NEG	NEG	NEG	NEG	NEG	NEG	POS	POS	POS	POS	POS
MSSA1839	NEG	NEG	NEG	NEG	NEG	NEG	NEG	NEG	NEG	NEG	NEG	NEG	POS	POS	POS	POS	POS
MSSA1900	NEG	NEG	NEG	NEG	NEG	NEG	NEG	NEG	NEG	NEG	NEG	NEG	POS	POS	POS	POS	POS
MSSA1910	NEG	NEG	NEG	NEG	NEG	NEG	NEG	NEG	NEG	NEG	NEG	NEG	POS	POS	POS	POS	POS
MSSA1918	NEG	NEG	NEG	NEG	NEG	NEG	NEG	NEG	NEG	NEG	NEG	NEG	POS	POS	POS	POS	POS
MSSA1921	NEG	NEG	NEG	NEG	NEG	NEG	NEG	NEG	NEG	NEG	NEG	NEG	NEG	NEG	NEG	NEG	NEG
MSSA1923	NEG	NEG	NEG	NEG	NEG	NEG	NEG	NEG	NEG	NEG	NEG	NEG	POS	POS	POS	POS	POS
MSSA1981	NEG	NEG	NEG	NEG	NEG	NEG	NEG	NEG	NEG	NEG	NEG	NEG	POS	POS	POS	POS	POS
MSSA1998	NEG	NEG	NEG	NEG	NEG	NEG	NEG	NEG	NEG	NEG	NEG	NEG	POS	POS	POS	POS	POS
MSSA2000	NEG	NEG	NEG	NEG	NEG	NEG	NEG	NEG	NEG	NEG	NEG	NEG	POS	POS	POS	POS	POS
MSSA2007	NEG	NEG	NEG	NEG	NEG	NEG	NEG	NEG	NEG	NEG	NEG	NEG	POS	POS	POS	POS	POS
MSSA2012	NEG	NEG	NEG	NEG	NEG	NEG	NEG	NEG	NEG	NEG	NEG	NEG	POS	POS	POS	POS	POS
MSSA2015	NEG	NEG	NEG	NEG	NEG	NEG	NEG	NEG	NEG	NEG	NEG	NEG	POS	POS	POS	POS	POS
MSSA2021	NEG	NEG	NEG	NEG	NEG	NEG	NEG	NEG	NEG	NEG	NEG	NEG	POS	POS	POS	POS	POS
MSSA2027	NEG	NEG	NEG	NEG	NEG	NEG	NEG	NEG	NEG	NEG	NEG	NEG	POS	POS	POS	POS	POS
MSSA2063	NEG	NEG	NEG	NEG	NEG	NEG	NEG	NEG	NEG	NEG	NEG	NEG	POS	POS	POS	POS	POS
MSSA2064	NEG	NEG	NEG	NEG	NEG	NEG	NEG	NEG	NEG	NEG	NEG	NEG	POS	POS	POS	POS	POS
MSSA2065	NEG	NEG	NEG	NEG	NEG	NEG	NEG	NEG	NEG	NEG	NEG	NEG	POS	POS	POS	POS	POS
MSSA2075	NEG	NEG	NEG	NEG	NEG	NEG	NEG	NEG	NEG	NEG	NEG	NEG	POS	POS	POS	POS	POS
MSSA2093	NEG	NEG	NEG	NEG	NEG	NEG	NEG	NEG	NEG	NEG	NEG	NEG	POS	POS	POS	POS	POS
MSSA2102	NEG	NEG	NEG	NEG	NEG	NEG	NEG	NEG	NEG	NEG	NEG	NEG	POS	POS	POS	POS	POS
MSSA2138	NEG	NEG	NEG	NEG	NEG	NEG	NEG	NEG	NEG	NEG	NEG	NEG	POS	POS	POS	POS	POS
MSSA2168	NEG	NEG	NEG	NEG	NEG	NEG	NEG	NEG	NEG	NEG	NEG	NEG	POS	POS	POS	POS	POS
MSSA2181	NEG	NEG	NEG	NEG	NEG	NEG	NEG	NEG	NEG	NEG	NEG	NEG	POS	POS	POS	POS	POS
MSSA2189	NEG	NEG	NEG	NEG	NEG	NEG	NEG	NEG	NEG	NEG	NEG	NEG	POS	POS	POS	POS	POS
MSSA2214	NEG	NEG	NEG	NEG	NEG	NEG	NEG	NEG	NEG	NEG	NEG	NEG	POS	POS	POS	POS	POS
MRSA3803	NEG	NEG	NEG	NEG	NEG	NEG	NEG	NEG	NEG	NEG	NEG	NEG	POS	POS	POS	POS	POS
MSSA1750	NEG	NEG	NEG	NEG	NEG	NEG	NEG	NEG	NEG	NEG	NEG	NEG	NEG	NEG	POS	NEG	NEG
MSSA1756	NEG	NEG	NEG	NEG	NEG	NEG	NEG	NEG	NEG	NEG	NEG	NEG	POS	POS	POS	POS	POS
MSSA1759	NEG	NEG	NEG	NEG	NEG	NEG	NEG	NEG	NEG	NEG	NEG	NEG	NEG	NEG	NEG	NEG	NEG
MSSA1767	NEG	NEG	NEG	NEG	NEG	NEG	NEG	NEG	NEG	NEG	NEG	NEG	POS	POS	POS	POS	POS
MSSA1787	NEG	NEG	NEG	NEG	NEG	NEG	NEG	NEG	NEG	NEG	NEG	NEG	POS	POS	POS	POS	POS
MSSA1790	NEG	NEG	NEG	NEG	NEG	NEG	NEG	NEG	NEG	NEG	NEG	NEG	NEG	NEG	NEG	NEG	NEG
MSSA1794	NEG	NEG	NEG	NEG	NEG	NEG	NEG	NEG	NEG	NEG	NEG	NEG	POS	POS	POS	POS	POS
MSSA1808	NEG	NEG	NEG	NEG	NEG	NEG	NEG	NEG	NEG	NEG	NEG	NEG	NEG	NEG	NEG	NEG	NEG
MSSA1812	NEG	NEG	NEG	NEG	NEG	NEG	NEG	NEG	NEG	NEG	NEG	NEG	POS	POS	POS	POS	POS
MSSA1819	NEG	NEG	NEG	NEG	NEG	NEG	NEG	NEG	NEG	NEG	NEG	NEG	POS	POS	POS	POS	POS
MSSA1824	NEG	NEG	NEG	NEG	NEG	NEG	NEG	NEG	NEG	NEG	NEG	NEG	POS	POS	POS	POS	POS
MSSA1827	NEG	NEG	NEG	NEG	NEG	NEG	NEG	NEG	NEG	NEG	NEG	NEG	POS	POS	POS	POS	POS
MSSA1889	NEG	NEG	NEG	NEG	NEG	NEG	NEG	NEG	NEG	NEG	NEG	NEG	POS	POS	POS	POS	POS
MSSA1895	NEG	NEG	NEG	NEG	NEG	NEG	NEG	NEG	NEG	NEG	NEG	NEG	POS	POS	POS	POS	POS
MSSA1904	NEG	NEG	NEG	NEG	NEG	NEG	NEG	NEG	NEG	NEG	NEG	NEG	POS	POS	POS	POS	POS
MSSA1905	NEG	NEG	NEG	NEG	NEG	NEG	NEG	NEG	NEG	NEG	NEG	NEG	POS	POS	POS	POS	POS
MSSA1907	NEG	NEG	NEG	NEG	NEG	NEG	NEG	NEG	NEG	NEG	NEG	NEG	POS	POS	POS	POS	POS
MSSA1919	NEG	NEG	NEG	NEG	NEG	NEG	NEG	NEG	NEG	NEG	NEG	NEG	POS	POS	POS	POS	POS
MSSA1920	NEG	NEG	NEG	NEG	NEG	NEG	NEG	NEG	NEG	NEG	NEG	NEG	POS	POS	POS	POS	POS
MSSA1929	NEG	NEG	NEG	NEG	NEG	NEG	NEG	NEG	NEG	NEG	NEG	NEG	POS	POS	POS	POS	POS
MSSA1930	NEG	NEG	NEG	NEG	NEG	NEG	NEG	NEG	NEG	NEG	NEG	NEG	POS	POS	POS	POS	POS
MSSA1934	NEG	NEG	NEG	NEG	NEG	NEG	NEG	NEG	NEG	NEG	NEG	NEG	POS	POS	POS	POS	POS
MSSA1946	NEG	NEG	NEG	NEG	NEG	NEG	NEG	NEG	NEG	NEG	NEG	NEG	NEG	NEG	NEG	NEG	NEG
MSSA1971	NEG	NEG	NEG	NEG	NEG	NEG	NEG	NEG	NEG	NEG	NEG	NEG	NEG	NEG	NEG	NEG	NEG
MSSA1980	NEG	NEG	NEG	NEG	NEG	NEG	NEG	NEG	NEG	NEG	NEG	NEG	POS	POS	POS	POS	POS
MSSA1982	NEG	NEG	NEG	NEG	NEG	NEG	NEG	NEG	NEG	NEG	NEG	NEG	POS	POS	POS	POS	POS
MSSA1996	NEG	NEG	NEG	NEG	NEG	NEG	NEG	NEG	NEG	NEG	NEG	NEG	POS	POS	POS	POS	POS
MSSA2002	NEG	NEG	NEG	NEG	NEG	NEG	NEG	NEG	NEG	NEG	NEG	NEG	POS	POS	POS	POS	POS
MSSA2014	NEG	NEG	NEG	NEG	NEG	NEG	NEG	NEG	NEG	NEG	NEG	NEG	NEG	NEG	NEG	NEG	NEG
MSSA2053	NEG	NEG	NEG	NEG	NEG	NEG	NEG	NEG	NEG	NEG	NEG	NEG	NEG	NEG	NEG	NEG	NEG
MSSA2054	NEG	NEG	NEG	NEG	NEG	NEG	NEG	NEG	NEG	NEG	NEG	NEG	POS	POS	POS	POS	POS
MSSA2058	NEG	NEG	NEG	NEG	NEG	NEG	NEG	NEG	NEG	NEG	NEG	NEG	NEG	NEG	NEG	NEG	NEG
MSSA2068	NEG	NEG	NEG	NEG	NEG	NEG	NEG	NEG	NEG	NEG	NEG	NEG	POS	POS	POS	POS	POS
MSSA2070	NEG	NEG	NEG	NEG	NEG	NEG	NEG	NEG	NEG	NEG	NEG	NEG	POS	POS	POS	POS	POS
MSSA2072	NEG	NEG	NEG	NEG	NEG	NEG	NEG	NEG	NEG	NEG	NEG	NEG	POS	POS	POS	POS	POS
MSSA2073	NEG	NEG	NEG	NEG	NEG	NEG	NEG	NEG	NEG	NEG	NEG	NEG	POS	POS	POS	POS	POS
MSSA2074	NEG	NEG	NEG	NEG	NEG	NEG	NEG	NEG	NEG	NEG	NEG	NEG	NEG	NEG	NEG	NEG	NEG
MSSA2107	NEG	NEG	NEG	NEG	NEG	NEG	NEG	NEG	NEG	NEG	NEG	NEG	NEG	NEG	NEG	NEG	NEG
MSSA2108	NEG	NEG	NEG	NEG	NEG	NEG	NEG	NEG	NEG	NEG	NEG	NEG	POS	POS	POS	POS	POS
MSSA2129	NEG	NEG	NEG	NEG	NEG	NEG	NEG	NEG	NEG	NEG	NEG	NEG	NEG	NEG	NEG	NEG	NEG
MSSA2135	NEG	NEG	NEG	NEG	NEG	NEG	NEG	NEG	NEG	NEG	NEG	NEG	POS	POS	POS	POS	POS
MSSA2137	NEG	NEG	NEG	NEG	NEG	NEG	NEG	NEG	NEG	NEG	NEG	NEG	NEG	NEG	NEG	NEG	NEG
MSSA2140	NEG	NEG	NEG	POS	NEG	NEG	NEG	NEG	NEG	NEG	NEG	NEG	POS	POS	POS	POS	POS
MSSA2182	NEG	NEG	NEG	NEG	NEG	NEG	NEG	NEG	NEG	NEG	NEG	NEG	NEG	NEG	NEG	NEG	NEG
MSSA2188	NEG	NEG	NEG	NEG	NEG	NEG	NEG	NEG	NEG	NEG	NEG	NEG	POS	POS	POS	POS	POS
MSSA1899	NEG	NEG	NEG	NEG	NEG	NEG	NEG	NEG	NEG	NEG	NEG	NEG	NEG	NEG	NEG	NEG	NEG
MSSA1915	NEG	NEG	NEG	NEG	NEG	NEG	NEG	NEG	NEG	NEG	NEG	NEG	POS	POS	POS	POS	POS
MSSA1949	NEG	NEG	NEG	NEG	NEG	NEG	NEG	NEG	NEG	NEG	NEG	NEG	NEG	NEG	NEG	NEG	NEG
MSSA2042	NEG	NEG	NEG	NEG	NEG	NEG	NEG	NEG	NEG	NEG	NEG	NEG	POS	POS	POS	POS	POS
MSSA2077	NEG	NEG	NEG	NEG	NEG	NEG	NEG	NEG	NEG	NEG	NEG	NEG	NEG	NEG	NEG	NEG	NEG
MSSA2095	NEG	AMB	NEG	NEG	NEG	NEG	NEG	NEG	NEG	NEG	NEG	NEG	POS	POS	POS	POS	POS
MSSA1997	NEG	NEG	NEG	NEG	NEG	NEG	NEG	NEG	NEG	NEG	NEG	NEG	POS	POS	POS	POS	POS
MSSA1810	NEG	NEG	NEG	NEG	NEG	NEG	NEG	NEG	NEG	NEG	NEG	NEG	NEG	NEG	NEG	NEG	NEG
MSSA1832	NEG	NEG	NEG	NEG	NEG	NEG	NEG	NEG	NEG	NEG	NEG	NEG	POS	POS	POS	POS	POS
MSSA1891	NEG	NEG	NEG	NEG	NEG	NEG	NEG	NEG	NEG	NEG	NEG	NEG	NEG	NEG	NEG	NEG	NEG
MSSA1892	NEG	NEG	NEG	NEG	NEG	NEG	NEG	NEG	NEG	NEG	NEG	NEG	POS	POS	POS	POS	POS
MSSA1932	NEG	NEG	NEG	NEG	NEG	NEG	NEG	NEG	NEG	NEG	NEG	NEG	POS	POS	POS	POS	POS
MSSA1942	NEG	NEG	NEG	NEG	NEG	NEG	NEG	NEG	NEG	NEG	NEG	NEG	NEG	NEG	NEG	NEG	NEG
MSSA1957	NEG	NEG	NEG	NEG	NEG	NEG	NEG	NEG	NEG	NEG	NEG	NEG	POS	POS	POS	POS	POS
MSSA1748	NEG	NEG	NEG	NEG	NEG	NEG	NEG	NEG	NEG	NEG	NEG	NEG	NEG	NEG	NEG	NEG	NEG
MSSA1754	NEG	NEG	NEG	NEG	NEG	NEG	NEG	NEG	NEG	NEG	NEG	NEG	NEG	NEG	NEG	NEG	NEG
MSSA1770	NEG	NEG	NEG	NEG	NEG	NEG	NEG	NEG	NEG	NEG	NEG	NEG	NEG	NEG	NEG	NEG	NEG
MSSA1788	NEG	NEG	NEG	NEG	NEG	NEG	NEG	NEG	NEG	NEG	NEG	NEG	NEG	NEG	NEG	NEG	NEG
MSSA1804	NEG	NEG	NEG	NEG	NEG	NEG	NEG	NEG	NEG	NEG	NEG	NEG	NEG	NEG	NEG	NEG	NEG
MSSA1818	NEG	NEG	NEG	NEG	NEG	NEG	NEG	NEG	NEG	NEG	NEG	NEG	NEG	NEG	NEG	NEG	NEG
MSSA1914	NEG	NEG	NEG	NEG	NEG	NEG	NEG	NEG	NEG	NEG	NEG	NEG	NEG	NEG	NEG	NEG	NEG
MSSA1974	NEG	NEG	NEG	NEG	NEG	NEG	NEG	NEG	NEG	NEG	NEG	NEG	NEG	NEG	NEG	NEG	NEG
MSSA2023	NEG	NEG	NEG	NEG	NEG	NEG	NEG	NEG	NEG	NEG	NEG	NEG	NEG	NEG	NEG	NEG	NEG
MSSA2030	NEG	NEG	NEG	NEG	NEG	NEG	NEG	NEG	NEG	NEG	NEG	NEG	NEG	NEG	NEG	NEG	NEG
MSSA2096	NEG	NEG	NEG	NEG	NEG	NEG	NEG	NEG	NEG	AMB	NEG	NEG	NEG	NEG	NEG	NEG	NEG
MSSA2142	NEG	NEG	NEG	NEG	NEG	NEG	NEG	NEG	NEG	NEG	NEG	NEG	NEG	NEG	NEG	NEG	NEG
MSSA2203	NEG	NEG	NEG	NEG	NEG	NEG	NEG	NEG	NEG	NEG	NEG	NEG	NEG	NEG	NEG	NEG	NEG
MSSA1901	NEG	NEG	NEG	NEG	NEG	NEG	NEG	NEG	NEG	NEG	NEG	NEG	POS	POS	POS	POS	POS
MSSA2013	NEG	NEG	NEG	NEG	NEG	NEG	NEG	NEG	NEG	NEG	NEG	NEG	POS	POS	POS	POS	POS
MSSA2031	NEG	NEG	NEG	NEG	NEG	NEG	NEG	NEG	NEG	NEG	NEG	NEG	POS	POS	POS	POS	POS
MSSA2052	NEG	NEG	NEG	NEG	NEG	NEG	NEG	NEG	NEG	NEG	NEG	NEG	POS	POS	POS	POS	AMB
MSSA2057	NEG	NEG	NEG	NEG	NEG	NEG	NEG	NEG	NEG	NEG	NEG	NEG	NEG	NEG	NEG	NEG	NEG
MSSA2081	NEG	NEG	NEG	NEG	NEG	NEG	NEG	NEG	NEG	NEG	NEG	NEG	NEG	NEG	NEG	NEG	NEG
MSSA1955	NEG	NEG	NEG	NEG	NEG	NEG	NEG	NEG	NEG	NEG	NEG	NEG	POS	POS	POS	POS	POS
MSSA1941	NEG	NEG	NEG	NEG	NEG	NEG	NEG	NEG	NEG	NEG	NEG	NEG	NEG	NEG	NEG	NEG	NEG
MRSA3838	NEG	NEG	NEG	NEG	NEG	NEG	NEG	POS	POS	POS	NEG	NEG	POS	POS	POS	POS	AMB
MRSA3839	NEG	NEG	NEG	NEG	NEG	NEG	NEG	POS	POS	POS	NEG	NEG	POS	POS	POS	POS	POS
MSSA1801	NEG	NEG	NEG	NEG	NEG	NEG	NEG	NEG	NEG	NEG	NEG	NEG	POS	POS	POS	POS	POS
MSSA2131	NEG	NEG	NEG	NEG	NEG	NEG	NEG	NEG	NEG	NEG	NEG	NEG	POS	POS	POS	POS	POS
MSSA1792	NEG	NEG	NEG	NEG	NEG	NEG	NEG	NEG	NEG	NEG	NEG	NEG	POS	POS	POS	POS	AMB
MSSA1796	NEG	NEG	NEG	NEG	NEG	NEG	NEG	NEG	NEG	NEG	NEG	NEG	NEG	NEG	AMB	NEG	NEG
MSSA1817	NEG	NEG	NEG	NEG	NEG	NEG	NEG	NEG	NEG	NEG	NEG	NEG	NEG	NEG	NEG	NEG	NEG
MSSA1911	NEG	NEG	NEG	NEG	NEG	NEG	NEG	NEG	NEG	NEG	NEG	NEG	POS	POS	POS	POS	POS
MSSA1927	NEG	NEG	NEG	NEG	NEG	NEG	NEG	NEG	NEG	NEG	NEG	NEG	NEG	NEG	NEG	NEG	NEG
MSSA1928	NEG	NEG	NEG	NEG	NEG	NEG	NEG	NEG	NEG	NEG	NEG	NEG	POS	POS	POS	POS	POS
MSSA1953	NEG	NEG	NEG	NEG	NEG	NEG	NEG	NEG	NEG	NEG	NEG	NEG	NEG	NEG	NEG	NEG	NEG
MSSA2062	NEG	NEG	NEG	NEG	NEG	NEG	NEG	NEG	NEG	NEG	NEG	NEG	POS	POS	POS	POS	AMB
MSSA2078	NEG	NEG	NEG	NEG	NEG	NEG	NEG	NEG	NEG	NEG	NEG	NEG	POS	POS	POS	POS	POS
MSSA2136	NEG	NEG	NEG	NEG	NEG	NEG	NEG	NEG	NEG	NEG	NEG	NEG	POS	POS	POS	POS	POS

	RESISTANCE: MLS-ANTIBIOTICS														RESISTANCE: AMINOGLYSOSIDES		
Strain	erm(A)	erm(B)	erm(C)	lin(A)	msr(A)	mef(A) (probe 1)	mef(A) (probe 2)	mph(C) (probe)	mph(C) (probe 2)	vat(A)	vat(B)	vga(A)	vga(A) BM3327	vgb	aacA-aphD	aadD	aphA-3
MSSA1805	NEG	NEG	NEG	NEG	NEG	NEG	NEG	NEG	NEG	NEG	NEG	NEG	NEG	NEG	NEG	NEG	NEG
MSSA1823	NEG	NEG	NEG	NEG	NEG	NEG	NEG	NEG	NEG	NEG	NEG	NEG	NEG	NEG	NEG	NEG	NEG
MSSA1837	NEG	NEG	NEG	NEG	NEG	NEG	NEG	NEG	NEG	NEG	NEG	NEG	NEG	NEG	NEG	NEG	NEG
MSSA2044	NEG	NEG	POS	NEG	NEG	NEG	NEG	NEG	NEG	NEG	NEG	NEG	NEG	NEG	NEG	NEG	NEG
MSSA2046	NEG	NEG	NEG	NEG	NEG	NEG	NEG	NEG	NEG	NEG	NEG	NEG	NEG	NEG	NEG	NEG	NEG
MSSA2059	NEG	NEG	NEG	NEG	NEG	NEG	NEG	NEG	NEG	NEG	NEG	NEG	NEG	NEG	NEG	NEG	NEG
MSSA2076	NEG	NEG	NEG	NEG	NEG	NEG	NEG	NEG	NEG	NEG	NEG	NEG	NEG	NEG	NEG	NEG	NEG
MSSA2200	POS	NEG	NEG	NEG	NEG	NEG	NEG	NEG	NEG	NEG	NEG	NEG	NEG	NEG	NEG	NEG	NEG
MRSA3797	POS	NEG	NEG	NEG	NEG	NEG	NEG	NEG	NEG	NEG	NEG	NEG	NEG	NEG	NEG	POS	NEG
MRSA3798	POS	NEG	NEG	NEG	NEG	NEG	NEG	NEG	NEG	NEG	NEG	NEG	NEG	NEG	NEG	POS	NEG
MRSA3799	POS	NEG	NEG	NEG	NEG	NEG	NEG	NEG	NEG	NEG	NEG	NEG	NEG	NEG	NEG	POS	NEG
MRSA3800	POS	NEG	NEG	NEG	NEG	NEG	NEG	NEG	NEG	NEG	NEG	NEG	NEG	NEG	NEG	POS	NEG
MRSA3801	POS	NEG	NEG	NEG	NEG	NEG	NEG	NEG	NEG	NEG	NEG	NEG	NEG	NEG	NEG	POS	NEG
MRSA3804	POS	NEG	NEG	NEG	NEG	NEG	NEG	NEG	NEG	NEG	NEG	NEG	NEG	NEG	NEG	POS	NEG
MRSA3806	POS	NEG	NEG	NEG	NEG	NEG	NEG	NEG	NEG	NEG	NEG	NEG	NEG	NEG	NEG	POS	NEG
MRSA3961	POS	NEG	NEG	NEG	NEG	NEG	NEG	NEG	NEG	NEG	NEG	NEG	NEG	NEG	NEG	POS	NEG
MRSA3962	NEG	NEG	POS	NEG	NEG	NEG	NEG	NEG	NEG	NEG	NEG	NEG	NEG	NEG	NEG	NEG	NEG
MRSA3963	POS	NEG	NEG	NEG	NEG	NEG	NEG	NEG	NEG	NEG	NEG	NEG	NEG	NEG	NEG	POS	NEG
MRSA3966	POS	NEG	NEG	NEG	NEG	NEG	NEG	NEG	NEG	NEG	NEG	NEG	NEG	NEG	NEG	POS	NEG
MRSA3978	POS	NEG	NEG	NEG	NEG	NEG	NEG	NEG	NEG	NEG	NEG	NEG	NEG	NEG	NEG	POS	NEG
MRSA3979	POS	NEG	NEG	NEG	NEG	NEG	NEG	NEG	NEG	NEG	NEG	NEG	NEG	NEG	NEG	AMB	NEG
MRSA3980	POS	NEG	NEG	NEG	NEG	NEG	NEG	NEG	NEG	NEG	NEG	NEG	NEG	NEG	NEG	POS	NEG
MRSA4179	POS	NEG	NEG	NEG	NEG	NEG	NEG	NEG	NEG	NEG	NEG	NEG	NEG	NEG	NEG	POS	NEG
MRSA4216	POS	NEG	NEG	NEG	NEG	NEG	NEG	NEG	NEG	NEG	NEG	NEG	NEG	NEG	NEG	NEG	NEG
MRSA4217	POS	NEG	NEG	NEG	NEG	NEG	NEG	NEG	NEG	NEG	NEG	NEG	NEG	NEG	NEG	NEG	NEG
MRSA4220	POS	NEG	NEG	NEG	NEG	NEG	NEG	NEG	NEG	NEG	NEG	NEG	NEG	NEG	NEG	POS	NEG
MRSA4221	POS	NEG	NEG	NEG	NEG	NEG	NEG	NEG	NEG	NEG	NEG	NEG	NEG	NEG	NEG	POS	NEG
MRSA4222	POS	NEG	NEG	NEG	NEG	NEG	NEG	NEG	NEG	NEG	NEG	NEG	NEG	NEG	NEG	POS	NEG
MSSA1751	NEG	NEG	NEG	NEG	NEG	NEG	NEG	NEG	NEG	NEG	NEG	NEG	NEG	NEG	NEG	NEG	NEG
MSSA1760	NEG	NEG	NEG	NEG	NEG	NEG	NEG	NEG	NEG	NEG	NEG	NEG	NEG	NEG	NEG	NEG	NEG
MSSA1802	NEG	NEG	NEG	NEG	NEG	NEG	NEG	NEG	NEG	NEG	NEG	NEG	NEG	NEG	NEG	NEG	NEG
MSSA1809	NEG	NEG	NEG	NEG	NEG	NEG	NEG	NEG	NEG	NEG	NEG	NEG	NEG	NEG	POS	NEG	NEG
MSSA1815	NEG	NEG	NEG	NEG	NEG	NEG	NEG	NEG	NEG	NEG	NEG	NEG	NEG	NEG	NEG	NEG	NEG
MSSA1829	NEG	NEG	NEG	NEG	NEG	NEG	NEG	NEG	NEG	NEG	NEG	NEG	NEG	NEG	NEG	NEG	NEG
MSSA1908	NEG	NEG	POS	NEG	NEG	NEG	NEG	NEG	NEG	NEG	NEG	NEG	NEG	NEG	NEG	NEG	NEG
MSSA1960	NEG	NEG	NEG	NEG	NEG	NEG	NEG	NEG	NEG	NEG	NEG	NEG	NEG	NEG	NEG	NEG	NEG
MSSA1965	NEG	NEG	NEG	NEG	NEG	NEG	NEG	NEG	NEG	NEG	NEG	NEG	NEG	NEG	NEG	NEG	NEG
MSSA1977	NEG	NEG	NEG	NEG	NEG	NEG	NEG	NEG	NEG	NEG	NEG	NEG	NEG	NEG	NEG	NEG	NEG
MSSA1978	NEG	NEG	NEG	NEG	NEG	NEG	NEG	NEG	NEG	NEG	NEG	NEG	NEG	NEG	NEG	NEG	NEG
MSSA1999	NEG	NEG	NEG	NEG	NEG	NEG	NEG	NEG	NEG	NEG	NEG	NEG	NEG	NEG	NEG	NEG	NEG
MSSA2003	NEG	NEG	NEG	NEG	NEG	NEG	NEG	NEG	NEG	NEG	NEG	NEG	NEG	NEG	NEG	NEG	NEG
MSSA2006	NEG	NEG	NEG	NEG	NEG	NEG	NEG	NEG	NEG	NEG	NEG	NEG	NEG	NEG	NEG	NEG	NEG
MSSA2008	NEG	NEG	NEG	NEG	NEG	NEG	NEG	NEG	NEG	NEG	NEG	NEG	NEG	NEG	NEG	NEG	NEG
MSSA2011	POS	NEG	NEG	NEG	NEG	NEG	NEG	NEG	NEG	NEG	NEG	NEG	NEG	NEG	NEG	POS	NEG
MSSA2017	NEG	NEG	POS	NEG	NEG	NEG	NEG	NEG	NEG	NEG	NEG	NEG	NEG	NEG	NEG	NEG	NEG
MSSA2022	POS	NEG	NEG	NEG	NEG	NEG	NEG	NEG	NEG	NEG	NEG	NEG	NEG	NEG	NEG	POS	NEG
MSSA2055	NEG	NEG	POS	NEG	NEG	NEG	NEG	NEG	NEG	NEG	NEG	NEG	NEG	NEG	NEG	NEG	NEG
MSSA2060	POS	NEG	NEG	NEG	NEG	NEG	NEG	NEG	NEG	NEG	NEG	NEG	NEG	NEG	NEG	NEG	NEG
MSSA2080	NEG	NEG	NEG	NEG	NEG	NEG	NEG	NEG	NEG	NEG	NEG	NEG	NEG	NEG	NEG	NEG	NEG
MSSA2082	NEG	NEG	NEG	NEG	NEG	NEG	NEG	NEG	NEG	NEG	NEG	NEG	NEG	NEG	NEG	NEG	NEG
MSSA2084	NEG	NEG	NEG	NEG	NEG	NEG	NEG	NEG	NEG	NEG	NEG	NEG	NEG	NEG	NEG	NEG	NEG
MSSA2087	NEG	NEG	NEG	NEG	NEG	NEG	NEG	NEG	NEG	NEG	NEG	NEG	NEG	NEG	NEG	NEG	NEG
MSSA2094	NEG	NEG	NEG	NEG	NEG	NEG	NEG	NEG	NEG	NEG	NEG	NEG	NEG	NEG	NEG	NEG	NEG
MSSA2097	NEG	NEG	NEG	NEG	NEG	NEG	NEG	NEG	NEG	NEG	NEG	NEG	NEG	NEG	NEG	NEG	NEG
MSSA2110	POS	NEG	NEG	NEG	NEG	NEG	NEG	NEG	NEG	NEG	NEG	NEG	NEG	NEG	NEG	NEG	NEG
MSSA2113	NEG	NEG	NEG	NEG	NEG	NEG	NEG	NEG	NEG	NEG	NEG	NEG	NEG	NEG	NEG	NEG	NEG
MSSA2132	NEG	NEG	NEG	NEG	NEG	NEG	NEG	NEG	NEG	NEG	NEG	NEG	NEG	NEG	NEG	NEG	NEG
MSSA2134	POS	NEG	NEG	NEG	NEG	NEG	NEG	NEG	NEG	NEG	NEG	NEG	NEG	NEG	NEG	NEG	NEG
MSSA1746	NEG	NEG	NEG	NEG	NEG	NEG	NEG	NEG	NEG	NEG	NEG	NEG	NEG	NEG	NEG	NEG	NEG
MSSA1757	NEG	NEG	NEG	NEG	NEG	NEG	NEG	NEG	NEG	NEG	NEG	NEG	NEG	NEG	NEG	NEG	NEG
MSSA1772	NEG	NEG	NEG	NEG	NEG	NEG	NEG	NEG	NEG	NEG	NEG	NEG	NEG	NEG	NEG	NEG	NEG
MSSA1795	NEG	NEG	NEG	NEG	NEG	NEG	NEG	NEG	NEG	NEG	NEG	NEG	NEG	NEG	NEG	NEG	NEG
MSSA1813	NEG	NEG	NEG	NEG	NEG	NEG	NEG	NEG	NEG	NEG	NEG	NEG	NEG	NEG	NEG	NEG	NEG
MSSA1826	NEG	NEG	NEG	NEG	NEG	NEG	NEG	NEG	NEG	NEG	NEG	NEG	NEG	NEG	NEG	NEG	NEG
MSSA1830	NEG	NEG	NEG	NEG	NEG	NEG	NEG	NEG	NEG	NEG	NEG	NEG	NEG	NEG	NEG	NEG	NEG
MSSA1838	NEG	NEG	NEG	NEG	NEG	NEG	NEG	NEG	NEG	NEG	NEG	NEG	NEG	NEG	NEG	NEG	NEG
MSSA1944	NEG	NEG	NEG	NEG	NEG	NEG	NEG	NEG	NEG	NEG	NEG	NEG	NEG	NEG	NEG	NEG	NEG
MSSA1947	NEG	NEG	NEG	NEG	NEG	NEG	NEG	NEG	NEG	NEG	NEG	NEG	NEG	NEG	NEG	NEG	NEG
MSSA1964	NEG	NEG	NEG	NEG	NEG	NEG	NEG	NEG	NEG	NEG	NEG	NEG	NEG	NEG	NEG	NEG	NEG
MSSA1969	NEG	NEG	NEG	NEG	NEG	NEG	NEG	NEG	NEG	NEG	NEG	NEG	NEG	NEG	NEG	NEG	NEG
MSSA2001	NEG	NEG	NEG	NEG	NEG	NEG	NEG	NEG	NEG	NEG	NEG	NEG	NEG	NEG	NEG	NEG	NEG
MSSA2004	NEG	NEG	NEG	NEG	NEG	NEG	NEG	NEG	NEG	NEG	NEG	NEG	NEG	NEG	NEG	NEG	NEG
MSSA2024	NEG	NEG	NEG	NEG	NEG	NEG	NEG	NEG	NEG	NEG	NEG	NEG	NEG	NEG	NEG	NEG	NEG
MSSA2025	NEG	NEG	NEG	NEG	NEG	NEG	NEG	NEG	NEG	NEG	NEG	NEG	NEG	NEG	NEG	NEG	NEG
MSSA2026	NEG	NEG	NEG	NEG	NEG	NEG	NEG	NEG	NEG	NEG	NEG	NEG	NEG	NEG	NEG	NEG	NEG
MSSA2032	NEG	NEG	NEG	NEG	NEG	NEG	NEG	NEG	NEG	NEG	NEG	NEG	NEG	NEG	NEG	NEG	NEG
MSSA2043	NEG	NEG	NEG	NEG	NEG	NEG	NEG	NEG	NEG	NEG	NEG	NEG	NEG	NEG	NEG	NEG	POS
MSSA2061	NEG	NEG	NEG	NEG	NEG	NEG	NEG	NEG	NEG	NEG	NEG	NEG	NEG	NEG	NEG	NEG	NEG
MSSA2085	NEG	NEG	NEG	NEG	NEG	NEG	NEG	NEG	NEG	NEG	NEG	NEG	NEG	NEG	NEG	NEG	NEG
MSSA2098	NEG	NEG	NEG	NEG	NEG	NEG	NEG	NEG	NEG	NEG	NEG	NEG	NEG	NEG	NEG	NEG	NEG
MSSA2100	NEG	NEG	POS	NEG	NEG	NEG	NEG	NEG	NEG	NEG	NEG	NEG	NEG	NEG	NEG	NEG	NEG
MSSA2111	NEG	NEG	NEG	NEG	NEG	NEG	NEG	NEG	NEG	NEG	NEG	NEG	NEG	NEG	NEG	NEG	NEG
MSSA2178	NEG	NEG	NEG	NEG	NEG	NEG	NEG	NEG	NEG	NEG	NEG	NEG	NEG	NEG	NEG	NEG	NEG
MSSA2202	NEG	NEG	NEG	NEG	NEG	NEG	NEG	NEG	NEG	NEG	NEG	NEG	NEG	NEG	NEG	NEG	NEG
MSSA2205	NEG	NEG	NEG	NEG	NEG	NEG	NEG	NEG	NEG	NEG	NEG	NEG	NEG	NEG	NEG	NEG	NEG
MRSA3796	POS	NEG	NEG	NEG	NEG	NEG	NEG	NEG	NEG	NEG	NEG	NEG	NEG	NEG	NEG	NEG	NEG
MRSA3805	POS	NEG	NEG	NEG	NEG	NEG	NEG	NEG	NEG	NEG	NEG	NEG	NEG	NEG	POS	NEG	NEG
MRSA3965	POS	NEG	NEG	NEG	NEG	NEG	NEG	NEG	NEG	NEG	NEG	NEG	NEG	NEG	NEG	NEG	NEG
MSSA1761	NEG	NEG	NEG	NEG	NEG	NEG	NEG	NEG	NEG	NEG	NEG	NEG	NEG	NEG	NEG	NEG	NEG
MSSA1764	NEG	NEG	NEG	NEG	NEG	NEG	NEG	NEG	NEG	NEG	NEG	NEG	NEG	NEG	NEG	NEG	NEG
MSSA1765	NEG	NEG	NEG	NEG	NEG	NEG	NEG	NEG	NEG	NEG	NEG	NEG	NEG	NEG	NEG	NEG	NEG
MSSA1784	NEG	NEG	NEG	NEG	NEG	NEG	NEG	NEG	NEG	NEG	NEG	NEG	NEG	NEG	NEG	NEG	NEG
MSSA1806	NEG	NEG	NEG	NEG	NEG	NEG	NEG	NEG	NEG	NEG	NEG	NEG	NEG	NEG	NEG	NEG	NEG
MSSA1807	NEG	NEG	NEG	NEG	NEG	NEG	NEG	NEG	NEG	NEG	NEG	NEG	NEG	NEG	NEG	NEG	NEG
MSSA1888	NEG	NEG	NEG	NEG	NEG	NEG	NEG	NEG	NEG	NEG	NEG	NEG	NEG	NEG	NEG	NEG	NEG
MSSA1894	NEG	NEG	NEG	NEG	NEG	NEG	NEG	NEG	NEG	NEG	NEG	NEG	NEG	NEG	NEG	NEG	NEG
MSSA1922	NEG	NEG	NEG	NEG	NEG	NEG	NEG	NEG	NEG	NEG	NEG	NEG	NEG	NEG	NEG	NEG	NEG
MSSA1950	POS	NEG	NEG	NEG	NEG	NEG	NEG	NEG	NEG	NEG	NEG	NEG	NEG	NEG	NEG	NEG	NEG
MSSA1954	POS	NEG	NEG	NEG	NEG	NEG	NEG	NEG	NEG	NEG	NEG	NEG	NEG	NEG	POS	POS	NEG
MSSA1966	NEG	NEG	POS	NEG	NEG	NEG	NEG	NEG	NEG	NEG	NEG	NEG	NEG	NEG	NEG	NEG	NEG
MSSA1972	NEG	NEG	NEG	NEG	NEG	NEG	NEG	NEG	NEG	NEG	NEG	NEG	NEG	NEG	NEG	NEG	NEG
MSSA2010	NEG	NEG	NEG	NEG	NEG	NEG	NEG	NEG	NEG	NEG	NEG	NEG	NEG	NEG	NEG	NEG	NEG
MSSA2020	NEG	NEG	NEG	NEG	NEG	NEG	NEG	NEG	NEG	NEG	NEG	NEG	NEG	NEG	NEG	NEG	NEG
MSSA2028	NEG	NEG	NEG	NEG	NEG	NEG	NEG	NEG	NEG	NEG	NEG	NEG	NEG	NEG	NEG	NEG	NEG
MSSA2088	NEG	NEG	NEG	NEG	NEG	NEG	NEG	NEG	NEG	NEG	NEG	NEG	NEG	NEG	NEG	NEG	NEG
MSSA2187	NEG	NEG	NEG	NEG	NEG	NEG	NEG	NEG	NEG	NEG	NEG	NEG	NEG	NEG	NEG	NEG	NEG
MSSA2204	NEG	NEG	NEG	NEG	NEG	NEG	NEG	NEG	NEG	NEG	NEG	NEG	NEG	NEG	NEG	NEG	NEG
MSSA2210	NEG	NEG	NEG	NEG	NEG	NEG	NEG	NEG	NEG	NEG	NEG	NEG	NEG	NEG	NEG	NEG	NEG
MSSA2223	NEG	NEG	POS	NEG	NEG	NEG	NEG	NEG	NEG	NEG	NEG	NEG	NEG	NEG	NEG	NEG	NEG
MSSA1763	POS	NEG	NEG	NEG	NEG	NEG	NEG	NEG	NEG	NEG	NEG	NEG	NEG	NEG	NEG	NEG	NEG
MSSA1777	NEG	NEG	NEG	NEG	NEG	NEG	NEG	NEG	NEG	NEG	NEG	NEG	NEG	NEG	NEG	NEG	NEG
MSSA2009	POS	NEG	NEG	NEG	NEG	NEG	NEG	NEG	NEG	NEG	NEG	NEG	NEG	NEG	NEG	NEG	NEG
MSSA2047	NEG	NEG	NEG	NEG	NEG	NEG	NEG	NEG	NEG	NEG	NEG	NEG	NEG	NEG	NEG	NEG	NEG
MSSA2079	NEG	NEG	NEG	NEG	NEG	NEG	NEG	NEG	NEG	NEG	NEG	NEG	NEG	NEG	NEG	NEG	NEG
MSSA2101	NEG	NEG	NEG	NEG	NEG	NEG	NEG	NEG	NEG	NEG	NEG	NEG	NEG	NEG	NEG	NEG	NEG
MSSA1821	NEG	NEG	NEG	NEG	NEG	NEG	NEG	NEG	NEG	NEG	NEG	NEG	NEG	NEG	NEG	NEG	NEG
MSSA1898	NEG	NEG	NEG	NEG	NEG	NEG	NEG	NEG	NEG	NEG	NEG	NEG	NEG	NEG	NEG	NEG	NEG
MSSA1973	NEG	NEG	NEG	NEG	NEG	NEG	NEG	NEG	NEG	NEG	NEG	NEG	NEG	NEG	NEG	NEG	NEG
MSSA2071	NEG	NEG	NEG	NEG	NEG	NEG	NEG	NEG	NEG	NEG	NEG	NEG	NEG	NEG	NEG	NEG	NEG
MSSA2083	NEG	NEG	NEG	NEG	NEG	NEG	NEG	NEG	NEG	NEG	NEG	NEG	NEG	NEG	NEG	NEG	NEG
MSSA2106	NEG	NEG	NEG	NEG	NEG	NEG	NEG	NEG	NEG	NEG	NEG	NEG	NEG	NEG	NEG	NEG	NEG
MSSA2112	NEG	NEG	NEG	NEG	NEG	NEG	NEG	NEG	NEG	NEG	NEG	NEG	NEG	NEG	NEG	NEG	NEG
MSSA2115	NEG	NEG	NEG	NEG	NEG	NEG	NEG	NEG	NEG	NEG	NEG	NEG	NEG	NEG	NEG	NEG	NEG
MSSA2195	NEG	NEG	POS	NEG	NEG	NEG	NEG	NEG	NEG	NEG	NEG	NEG	NEG	AMB	NEG	NEG	NEG
MSSA2207	NEG	NEG	NEG	NEG	NEG	NEG	NEG	NEG	NEG	NEG	NEG	NEG	NEG	NEG	NEG	NEG	NEG
MSSA1758	NEG	NEG	NEG	NEG	NEG	NEG	NEG	NEG	NEG	NEG	NEG	NEG	NEG	NEG	NEG	NEG	NEG
MSSA1762	NEG	NEG	NEG	NEG	NEG	NEG	NEG	NEG	NEG	NEG	NEG	NEG	NEG	NEG	NEG	NEG	NEG
MSSA1769	NEG	NEG	NEG	NEG	NEG	NEG	NEG	NEG	NEG	NEG	NEG	NEG	NEG	NEG	NEG	NEG	NEG
MSSA1771	NEG	NEG	NEG	NEG	NEG	NEG	NEG	NEG	NEG	NEG	NEG	NEG	NEG	NEG	NEG	NEG	NEG
MSSA1789	NEG	NEG	NEG	NEG	NEG	NEG	NEG	NEG	NEG	NEG	NEG	NEG	NEG	NEG	NEG	NEG	NEG
MSSA1800	NEG	NEG	NEG	NEG	NEG	NEG	NEG	NEG	NEG	NEG	NEG	NEG	NEG	NEG	NEG	NEG	NEG
MSSA1833	NEG	NEG	NEG	NEG	NEG	NEG	NEG	NEG	NEG	NEG	NEG	NEG	NEG	NEG	NEG	NEG	NEG
MSSA1890	NEG	NEG	NEG	NEG	NEG	NEG	NEG	NEG	NEG	NEG	NEG	NEG	NEG	NEG	NEG	NEG	NEG
MSSA1903	NEG	NEG	NEG	NEG	NEG	NEG	NEG	NEG	NEG	NEG	NEG	NEG	NEG	NEG	NEG	NEG	NEG
MSSA1940	NEG	NEG	NEG	NEG	NEG	NEG	NEG	NEG	NEG	NEG	NEG	NEG	NEG	NEG	NEG	NEG	NEG
MSSA1962	NEG	NEG	NEG	NEG	NEG	NEG	NEG	NEG	NEG	NEG	NEG	NEG	NEG	NEG	NEG	NEG	NEG
MSSA1963	NEG	NEG	NEG	NEG	NEG	NEG	NEG	NEG	NEG	NEG	NEG	NEG	NEG	NEG	NEG	NEG	NEG
MSSA2016	NEG	NEG	NEG	NEG	NEG	NEG	NEG	NEG	NEG	NEG	NEG	NEG	NEG	NEG	NEG	NEG	NEG
MSSA2018	NEG	NEG	POS	NEG	NEG	NEG	NEG	NEG	NEG	NEG	NEG	NEG	NEG	NEG	NEG	NEG	NEG
MSSA2019	NEG	NEG	NEG	NEG	NEG	NEG	NEG	NEG	NEG	NEG	NEG	NEG	NEG	NEG	NEG	NEG	NEG
MSSA2029	NEG	NEG	POS	NEG	NEG	NEG	NEG	NEG	NEG	NEG	NEG	NEG	NEG	NEG	NEG	NEG	NEG
MSSA2045	NEG	NEG	NEG	NEG	NEG	NEG	NEG	NEG	NEG	NEG	NEG	NEG	NEG	NEG	NEG	NEG	NEG
MSSA2048	NEG	NEG	NEG	NEG	NEG	NEG	NEG	NEG	NEG	NEG	NEG	NEG	NEG	NEG	NEG	NEG	NEG
MSSA2049	NEG	NEG	NEG	NEG	NEG	NEG	NEG	NEG	NEG	NEG	NEG	NEG	NEG	NEG	NEG	NEG	NEG
MSSA2050	NEG	NEG	NEG	NEG	NEG	NEG	NEG	NEG	NEG	NEG	NEG	NEG	NEG	NEG	NEG	NEG	NEG
MSSA2099	NEG	NEG	NEG	NEG	NEG	NEG	NEG	NEG	NEG	NEG	NEG	NEG	NEG	NEG	NEG	NEG	NEG
MSSA2109	NEG	NEG	NEG	NEG	NEG	NEG	NEG	NEG	NEG	NEG	NEG	NEG	NEG	NEG	NEG	NEG	NEG
MSSA2130	NEG	NEG	NEG	NEG	NEG	NEG	NEG	NEG	NEG	NEG	NEG	NEG	NEG	NEG	NEG	NEG	NEG
MSSA2133	NEG	NEG	NEG	NEG	NEG	NEG	NEG	NEG	NEG	NEG	NEG	NEG	NEG	NEG	NEG	NEG	NEG
MSSA2139	NEG	NEG	NEG	NEG	NEG	NEG	NEG	NEG	NEG	NEG	NEG	NEG	NEG	NEG	NEG	NEG	NEG
MSSA2141	NEG	NEG	NEG	NEG	NEG	NEG	NEG	NEG	NEG	NEG	NEG	NEG	NEG	NEG	NEG	NEG	NEG
MSSA2208	NEG	NEG	NEG	NEG	NEG	NEG	NEG	NEG	NEG	NEG	NEG	NEG	NEG	NEG	NEG	NEG	NEG
MSSA2209	NEG	NEG	POS	NEG	NEG	NEG	NEG	NEG	NEG	NEG	NEG	NEG	NEG	NEG	NEG	NEG	NEG
MSSA1893	NEG	NEG	NEG	NEG	NEG	NEG	NEG	NEG	NEG	NEG	NEG	NEG	NEG	NEG	NEG	NEG	NEG
MSSA2105	NEG	NEG	NEG	NEG	NEG	NEG	NEG	NEG	NEG	NEG	NEG	NEG	NEG	NEG	NEG	NEG	NEG
MRSA3836	NEG	NEG	POS	NEG	NEG	NEG	NEG	NEG	NEG	NEG	NEG	NEG	NEG	NEG	NEG	NEG	NEG
MRSA3840	NEG	NEG	POS	NEG	NEG	NEG	NEG	NEG	NEG	NEG	NEG	NEG	NEG	NEG	NEG	POS	NEG
MRSA4188	NEG	NEG	NEG	NEG	NEG	NEG	NEG	NEG	NEG	NEG	NEG	NEG	NEG	NEG	NEG	NEG	NEG
MSSA1814	NEG	NEG	NEG	NEG	NEG	NEG	NEG	NEG	NEG	NEG	NEG	NEG	NEG	NEG	NEG	NEG	NEG
MSSA1816	NEG	NEG	NEG	NEG	NEG	NEG	NEG	NEG	NEG	NEG	NEG	NEG	NEG	NEG	NEG	NEG	NEG
MSSA1825	NEG	NEG	NEG	NEG	NEG	NEG	NEG	NEG	NEG	NEG	NEG	NEG	NEG	NEG	NEG	NEG	NEG
MSSA1834	NEG	NEG	NEG	NEG	NEG	NEG	NEG	NEG	NEG	NEG	NEG	NEG	NEG	NEG	NEG	NEG	NEG
MSSA1938	NEG	NEG	NEG	NEG	NEG	NEG	NEG	NEG	NEG	NEG	NEG	NEG	NEG	NEG	NEG	NEG	NEG
MSSA1948	NEG	NEG	NEG	NEG	NEG	NEG	NEG	NEG	NEG	NEG	NEG	NEG	NEG	NEG	NEG	NEG	NEG
MSSA1951	NEG	NEG	NEG	NEG	NEG	NEG	NEG	NEG	NEG	NEG	NEG	NEG	NEG	NEG	NEG	NEG	NEG
MSSA2005	NEG	NEG	NEG	NEG	NEG	NEG	NEG	NEG	NEG	NEG	NEG	NEG	NEG	NEG	NEG	NEG	NEG
MSSA2051	NEG	NEG	NEG	NEG	NEG	NEG	NEG	NEG	NEG	NEG	NEG	NEG	NEG	NEG	NEG	NEG	NEG
MSSA2056	NEG	NEG	NEG	NEG	NEG	NEG	NEG	NEG	NEG	NEG	NEG	NEG	NEG	NEG	NEG	NEG	NEG
MSSA2066	NEG	NEG	NEG	NEG	NEG	NEG	NEG	NEG	NEG	NEG	NEG	NEG	NEG	NEG	NEG	NEG	NEG
MSSA2067	NEG	NEG	NEG	NEG	NEG	NEG	NEG	NEG	NEG	NEG	NEG	NEG	NEG	NEG	NEG	NEG	NEG
MSSA2086	NEG	NEG	NEG	NEG	NEG	NEG	NEG	NEG	NEG	NEG	NEG	NEG	NEG	NEG	NEG	NEG	NEG
MSSA2089	NEG	NEG	NEG	NEG	NEG	NEG	NEG	NEG	NEG	NEG	NEG	NEG	NEG	NEG	NEG	NEG	NEG
MSSA2103	NEG	NEG	NEG	NEG	NEG	NEG	NEG	NEG	NEG	NEG	NEG	NEG	NEG	NEG	NEG	NEG	NEG
MSSA2173	NEG	NEG	NEG	NEG	NEG	NEG	NEG	NEG	NEG	NEG	NEG	NEG	NEG	NEG	NEG	NEG	NEG
MSSA2183	NEG	NEG	NEG	NEG	NEG	NEG	NEG	NEG	NEG	NEG	NEG	NEG	NEG	NEG	NEG	NEG	NEG
MSSA2196	NEG	NEG	POS	NEG	NEG	NEG	NEG	NEG	NEG	NEG	NEG	NEG	NEG	NEG	NEG	NEG	NEG
MSSA2219	NEG	NEG	NEG	NEG	NEG	NEG	NEG	NEG	NEG	NEG	NEG	NEG	NEG	NEG	NEG	NEG	NEG
MSSA1753	NEG	NEG	NEG	NEG	NEG	NEG	NEG	NEG	NEG	NEG	NEG	NEG	NEG	NEG	NEG	NEG	NEG
MSSA1773	NEG	NEG	NEG	NEG	NEG	NEG	NEG	NEG	NEG	NEG	NEG	NEG	NEG	NEG	NEG	NEG	NEG
MSSA1811	NEG	NEG	NEG	NEG	POS	NEG	NEG	NEG	NEG	NEG	NEG	NEG	NEG	NEG	NEG	NEG	NEG
MSSA1897	NEG	NEG	NEG	NEG	NEG	NEG	NEG	NEG	NEG	NEG	NEG	NEG	NEG	NEG	NEG	NEG	NEG
MSSA1931	NEG	NEG	NEG	NEG	NEG	NEG	NEG	NEG	NEG	NEG	NEG	NEG	NEG	NEG	NEG	NEG	NEG
MSSA2114	NEG	NEG	NEG	NEG	NEG	NEG	NEG	NEG	NEG	NEG	NEG	NEG	NEG	NEG	NEG	NEG	NEG
MSSA1745	POS	NEG	NEG	NEG	NEG	NEG	NEG	NEG	NEG	NEG	NEG	NEG	NEG	NEG	NEG	NEG	NEG
MSSA1747	NEG	NEG	NEG	NEG	NEG	NEG	NEG	NEG	NEG	NEG	NEG	NEG	NEG	NEG	NEG	NEG	NEG
MSSA1749	NEG	NEG	NEG	NEG	NEG	NEG	NEG	NEG	NEG	NEG	NEG	NEG	NEG	NEG	NEG	NEG	NEG
MSSA1768	NEG	NEG	NEG	NEG	NEG	NEG	NEG	NEG	NEG	NEG	NEG	NEG	NEG	NEG	NEG	NEG	NEG
MSSA1774	NEG	NEG	NEG	NEG	NEG	NEG	NEG	NEG	NEG	NEG	NEG	NEG	NEG	NEG	NEG	NEG	NEG
MSSA1775	NEG	NEG	NEG	NEG	NEG	NEG	NEG	NEG	NEG	NEG	NEG	NEG	NEG	NEG	NEG	NEG	NEG
MSSA1776	NEG	NEG	NEG	NEG	NEG	NEG	NEG	NEG	NEG	NEG	NEG	NEG	NEG	NEG	NEG	NEG	NEG
MSSA1779	NEG	NEG	NEG	NEG	NEG	NEG	NEG	NEG	NEG	NEG	NEG	NEG	NEG	NEG	NEG	NEG	NEG
MSSA1780	POS	NEG	NEG	NEG	NEG	NEG	NEG	NEG	NEG	NEG	NEG	NEG	NEG	NEG	NEG	NEG	NEG

	RESISTANCE: MLS-ANTIBIOTICS														RESISTANCE: AMINOGLYSOSIDES		
Strain	erm(A)	erm(B)	erm(C)	lin(A)	msr(A)	mef(A) (probe 1)	mef(A) (probe 2)	mph(C) (probe)	mph(C) (probe 2)	vat(A)	vat(B)	vga(A)	vga(A) BM3327	vgb	aacA-aphD	aadD	aphA-3
MSSA1781	NEG	NEG	NEG	NEG	NEG	NEG	NEG	NEG	NEG	NEG	NEG	NEG	NEG	NEG	NEG	NEG	NEG
MSSA1786	NEG	NEG	NEG	NEG	NEG	NEG	NEG	NEG	NEG	NEG	NEG	NEG	NEG	NEG	NEG	NEG	NEG
MSSA1793	NEG	NEG	NEG	NEG	NEG	NEG	NEG	NEG	NEG	NEG	NEG	NEG	NEG	NEG	NEG	NEG	NEG
MSSA1803	NEG	NEG	NEG	NEG	NEG	NEG	NEG	NEG	NEG	NEG	NEG	NEG	NEG	NEG	NEG	NEG	NEG
MSSA1836	NEG	NEG	NEG	NEG	NEG	NEG	NEG	NEG	NEG	NEG	NEG	NEG	NEG	NEG	NEG	NEG	NEG
MSSA1839	NEG	NEG	NEG	NEG	NEG	NEG	NEG	NEG	NEG	NEG	NEG	NEG	NEG	NEG	NEG	NEG	NEG
MSSA1900	NEG	NEG	NEG	NEG	NEG	NEG	NEG	NEG	NEG	NEG	NEG	NEG	NEG	NEG	NEG	NEG	NEG
MSSA1910	NEG	NEG	NEG	NEG	NEG	NEG	NEG	NEG	NEG	NEG	NEG	NEG	NEG	NEG	NEG	NEG	NEG
MSSA1918	NEG	NEG	NEG	NEG	NEG	NEG	NEG	NEG	NEG	NEG	NEG	NEG	NEG	NEG	NEG	NEG	NEG
MSSA1921	NEG	NEG	NEG	NEG	NEG	NEG	NEG	NEG	NEG	NEG	NEG	NEG	NEG	NEG	NEG	NEG	NEG
MSSA1923	NEG	NEG	NEG	NEG	NEG	NEG	NEG	NEG	NEG	NEG	NEG	NEG	NEG	NEG	NEG	NEG	NEG
MSSA1981	NEG	NEG	NEG	NEG	NEG	NEG	NEG	NEG	NEG	NEG	NEG	NEG	NEG	NEG	NEG	NEG	NEG
MSSA1998	NEG	NEG	NEG	NEG	NEG	NEG	NEG	NEG	NEG	NEG	NEG	NEG	NEG	NEG	NEG	NEG	NEG
MSSA2000	NEG	NEG	NEG	NEG	NEG	NEG	NEG	NEG	NEG	NEG	NEG	NEG	NEG	NEG	NEG	NEG	NEG
MSSA2007	NEG	NEG	NEG	NEG	NEG	NEG	NEG	NEG	NEG	NEG	NEG	NEG	NEG	NEG	NEG	NEG	NEG
MSSA2012	POS	NEG	NEG	NEG	NEG	NEG	NEG	NEG	NEG	NEG	NEG	NEG	NEG	NEG	NEG	NEG	NEG
MSSA2015	NEG	NEG	NEG	NEG	NEG	NEG	NEG	NEG	NEG	NEG	NEG	NEG	NEG	NEG	NEG	NEG	NEG
MSSA2021	NEG	NEG	NEG	NEG	NEG	NEG	NEG	NEG	NEG	NEG	NEG	NEG	NEG	NEG	NEG	NEG	NEG
MSSA2027	NEG	NEG	NEG	NEG	NEG	NEG	NEG	NEG	NEG	NEG	NEG	NEG	NEG	NEG	NEG	NEG	NEG
MSSA2063	POS	NEG	NEG	NEG	NEG	NEG	NEG	NEG	NEG	NEG	NEG	NEG	NEG	NEG	NEG	NEG	NEG
MSSA2064	NEG	NEG	NEG	NEG	NEG	NEG	NEG	NEG	NEG	NEG	NEG	NEG	NEG	NEG	NEG	NEG	NEG
MSSA2065	NEG	NEG	NEG	NEG	NEG	NEG	NEG	NEG	NEG	NEG	NEG	NEG	NEG	NEG	NEG	NEG	NEG
MSSA2075	NEG	NEG	NEG	NEG	NEG	NEG	NEG	NEG	NEG	NEG	NEG	NEG	NEG	NEG	NEG	NEG	NEG
MSSA2093	NEG	NEG	NEG	NEG	NEG	NEG	NEG	NEG	NEG	NEG	NEG	NEG	NEG	NEG	NEG	NEG	NEG
MSSA2102	NEG	NEG	NEG	NEG	NEG	NEG	NEG	NEG	NEG	NEG	NEG	NEG	NEG	NEG	NEG	NEG	NEG
MSSA2138	NEG	NEG	NEG	NEG	NEG	NEG	NEG	NEG	NEG	NEG	NEG	NEG	NEG	NEG	NEG	NEG	NEG
MSSA2168	NEG	NEG	NEG	NEG	NEG	NEG	NEG	NEG	NEG	NEG	NEG	NEG	NEG	NEG	NEG	NEG	NEG
MSSA2181	NEG	NEG	NEG	NEG	NEG	NEG	NEG	NEG	NEG	NEG	NEG	NEG	NEG	NEG	NEG	NEG	NEG
MSSA2189	NEG	NEG	NEG	NEG	NEG	NEG	NEG	NEG	NEG	NEG	NEG	NEG	NEG	NEG	NEG	NEG	NEG
MSSA2214	NEG	NEG	NEG	NEG	NEG	NEG	NEG	NEG	NEG	NEG	NEG	NEG	NEG	NEG	NEG	NEG	NEG
MRSA3803	NEG	NEG	POS	NEG	NEG	NEG	NEG	NEG	NEG	NEG	NEG	NEG	NEG	NEG	POS	POS	NEG
MSSA1750	NEG	NEG	NEG	NEG	NEG	NEG	NEG	NEG	NEG	NEG	NEG	NEG	NEG	NEG	NEG	NEG	NEG
MSSA1756	NEG	NEG	NEG	NEG	NEG	NEG	NEG	NEG	NEG	NEG	NEG	NEG	NEG	NEG	NEG	NEG	NEG
MSSA1759	NEG	NEG	NEG	NEG	NEG	NEG	NEG	NEG	NEG	NEG	NEG	NEG	NEG	NEG	NEG	NEG	NEG
MSSA1767	NEG	NEG	NEG	NEG	NEG	NEG	NEG	NEG	NEG	NEG	NEG	NEG	NEG	NEG	NEG	NEG	NEG
MSSA1787	NEG	NEG	NEG	NEG	NEG	NEG	NEG	NEG	NEG	NEG	NEG	NEG	NEG	NEG	NEG	NEG	NEG
MSSA1790	NEG	NEG	NEG	NEG	NEG	NEG	NEG	NEG	NEG	NEG	NEG	NEG	NEG	NEG	NEG	NEG	NEG
MSSA1794	POS	NEG	NEG	NEG	NEG	NEG	NEG	NEG	NEG	NEG	NEG	NEG	NEG	NEG	NEG	NEG	NEG
MSSA1808	NEG	NEG	NEG	NEG	NEG	NEG	NEG	NEG	NEG	NEG	NEG	NEG	NEG	NEG	NEG	NEG	NEG
MSSA1812	NEG	NEG	NEG	NEG	NEG	NEG	NEG	NEG	NEG	NEG	NEG	NEG	NEG	NEG	NEG	NEG	NEG
MSSA1819	NEG	NEG	NEG	NEG	NEG	NEG	NEG	NEG	NEG	NEG	NEG	NEG	NEG	NEG	NEG	NEG	NEG
MSSA1824	NEG	NEG	NEG	NEG	NEG	NEG	NEG	NEG	NEG	NEG	NEG	NEG	NEG	NEG	NEG	NEG	NEG
MSSA1827	NEG	NEG	NEG	NEG	NEG	NEG	NEG	NEG	NEG	NEG	NEG	NEG	NEG	NEG	NEG	NEG	NEG
MSSA1889	NEG	NEG	NEG	NEG	NEG	NEG	NEG	NEG	NEG	NEG	NEG	NEG	NEG	NEG	NEG	NEG	NEG
MSSA1895	NEG	NEG	NEG	NEG	NEG	NEG	NEG	NEG	NEG	NEG	NEG	NEG	NEG	NEG	NEG	NEG	NEG
MSSA1904	NEG	NEG	NEG	NEG	NEG	NEG	NEG	NEG	NEG	NEG	NEG	NEG	NEG	NEG	NEG	NEG	NEG
MSSA1905	NEG	NEG	NEG	NEG	NEG	NEG	NEG	NEG	NEG	NEG	NEG	NEG	NEG	NEG	NEG	NEG	NEG
MSSA1907	NEG	NEG	NEG	NEG	NEG	NEG	NEG	NEG	NEG	NEG	NEG	NEG	NEG	NEG	NEG	NEG	NEG
MSSA1919	NEG	NEG	NEG	NEG	NEG	NEG	NEG	NEG	NEG	NEG	NEG	NEG	NEG	NEG	NEG	NEG	NEG
MSSA1920	POS	NEG	POS	NEG	NEG	NEG	NEG	NEG	NEG	NEG	NEG	NEG	NEG	NEG	NEG	NEG	NEG
MSSA1929	NEG	NEG	NEG	NEG	NEG	NEG	NEG	NEG	NEG	NEG	NEG	NEG	NEG	NEG	NEG	NEG	NEG
MSSA1930	NEG	NEG	NEG	NEG	NEG	NEG	NEG	NEG	NEG	NEG	NEG	NEG	NEG	NEG	NEG	NEG	NEG
MSSA1934	NEG	NEG	NEG	NEG	NEG	NEG	NEG	NEG	NEG	NEG	NEG	NEG	NEG	NEG	NEG	NEG	NEG
MSSA1946	NEG	NEG	NEG	NEG	NEG	NEG	NEG	NEG	NEG	NEG	NEG	NEG	NEG	NEG	NEG	NEG	NEG
MSSA1971	NEG	NEG	NEG	NEG	NEG	NEG	NEG	NEG	NEG	NEG	NEG	NEG	NEG	NEG	NEG	NEG	NEG
MSSA1980	NEG	NEG	NEG	NEG	NEG	NEG	NEG	NEG	NEG	NEG	NEG	NEG	NEG	NEG	NEG	NEG	NEG
MSSA1982	NEG	NEG	NEG	NEG	NEG	NEG	NEG	NEG	NEG	NEG	NEG	NEG	NEG	NEG	NEG	NEG	NEG
MSSA1996	NEG	NEG	NEG	NEG	NEG	NEG	NEG	NEG	NEG	NEG	NEG	NEG	NEG	NEG	NEG	NEG	NEG
MSSA2002	NEG	NEG	NEG	NEG	NEG	NEG	NEG	NEG	NEG	NEG	NEG	NEG	NEG	NEG	NEG	NEG	NEG
MSSA2014	NEG	NEG	NEG	NEG	NEG	NEG	NEG	NEG	NEG	NEG	NEG	NEG	NEG	NEG	NEG	NEG	NEG
MSSA2053	NEG	NEG	NEG	NEG	NEG	NEG	NEG	NEG	NEG	NEG	NEG	NEG	NEG	NEG	NEG	NEG	NEG
MSSA2054	NEG	NEG	NEG	NEG	NEG	NEG	NEG	NEG	NEG	NEG	NEG	NEG	NEG	NEG	NEG	NEG	NEG
MSSA2058	NEG	NEG	NEG	NEG	NEG	NEG	NEG	NEG	NEG	NEG	NEG	NEG	NEG	NEG	NEG	NEG	NEG
MSSA2068	NEG	NEG	NEG	NEG	NEG	NEG	NEG	NEG	NEG	NEG	NEG	NEG	NEG	NEG	NEG	NEG	NEG
MSSA2070	NEG	NEG	NEG	NEG	NEG	NEG	NEG	NEG	NEG	NEG	NEG	NEG	NEG	NEG	NEG	NEG	NEG
MSSA2072	POS	NEG	NEG	NEG	NEG	NEG	NEG	NEG	NEG	NEG	NEG	NEG	NEG	NEG	NEG	NEG	NEG
MSSA2073	NEG	NEG	NEG	NEG	NEG	NEG	NEG	NEG	NEG	NEG	NEG	NEG	NEG	NEG	NEG	NEG	NEG
MSSA2074	NEG	NEG	NEG	NEG	NEG	NEG	NEG	NEG	NEG	NEG	NEG	NEG	NEG	NEG	NEG	NEG	NEG
MSSA2107	NEG	NEG	NEG	NEG	NEG	NEG	NEG	NEG	NEG	NEG	NEG	NEG	NEG	NEG	NEG	NEG	NEG
MSSA2108	NEG	NEG	NEG	NEG	NEG	NEG	NEG	NEG	NEG	NEG	NEG	NEG	NEG	NEG	NEG	NEG	NEG
MSSA2129	NEG	NEG	NEG	NEG	NEG	NEG	NEG	NEG	NEG	NEG	NEG	NEG	NEG	NEG	NEG	NEG	NEG
MSSA2135	NEG	NEG	NEG	NEG	NEG	NEG	NEG	NEG	NEG	NEG	NEG	NEG	NEG	NEG	NEG	NEG	NEG
MSSA2137	NEG	NEG	NEG	NEG	NEG	NEG	NEG	NEG	NEG	NEG	NEG	NEG	NEG	NEG	NEG	NEG	NEG
MSSA2140	NEG	NEG	NEG	NEG	NEG	NEG	NEG	NEG	NEG	NEG	NEG	NEG	NEG	NEG	NEG	NEG	NEG
MSSA2182	NEG	NEG	NEG	NEG	NEG	NEG	NEG	NEG	NEG	NEG	NEG	NEG	NEG	NEG	NEG	NEG	NEG
MSSA2188	NEG	NEG	NEG	NEG	NEG	NEG	NEG	NEG	NEG	NEG	NEG	NEG	NEG	NEG	NEG	NEG	NEG
MSSA1899	NEG	NEG	NEG	NEG	NEG	NEG	NEG	NEG	NEG	NEG	NEG	NEG	NEG	NEG	NEG	NEG	NEG
MSSA1915	NEG	NEG	POS	NEG	NEG	NEG	NEG	NEG	NEG	NEG	NEG	NEG	NEG	NEG	NEG	NEG	NEG
MSSA1949	NEG	NEG	NEG	NEG	NEG	NEG	NEG	NEG	NEG	NEG	NEG	NEG	NEG	NEG	NEG	NEG	NEG
MSSA2042	NEG	NEG	NEG	NEG	NEG	NEG	NEG	NEG	NEG	NEG	NEG	NEG	NEG	NEG	NEG	NEG	NEG
MSSA2077	NEG	NEG	NEG	NEG	NEG	NEG	NEG	NEG	NEG	NEG	NEG	NEG	NEG	NEG	NEG	NEG	NEG
MSSA2095	NEG	NEG	NEG	NEG	NEG	NEG	NEG	NEG	NEG	NEG	NEG	NEG	NEG	NEG	NEG	NEG	NEG
MSSA1997	NEG	NEG	NEG	NEG	NEG	NEG	NEG	NEG	NEG	NEG	NEG	NEG	NEG	NEG	POS	NEG	NEG
MSSA1810	NEG	NEG	NEG	NEG	NEG	NEG	NEG	NEG	NEG	NEG	NEG	NEG	NEG	NEG	NEG	NEG	NEG
MSSA1832	AMB	NEG	NEG	NEG	NEG	NEG	NEG	NEG	NEG	NEG	NEG	NEG	NEG	NEG	NEG	NEG	NEG
MSSA1891	NEG	NEG	NEG	NEG	NEG	NEG	NEG	NEG	NEG	NEG	NEG	NEG	NEG	NEG	NEG	NEG	NEG
MSSA1892	NEG	NEG	NEG	NEG	NEG	NEG	NEG	NEG	NEG	NEG	NEG	NEG	NEG	NEG	NEG	NEG	NEG
MSSA1932	NEG	NEG	NEG	NEG	NEG	NEG	NEG	NEG	NEG	NEG	NEG	NEG	NEG	NEG	NEG	NEG	NEG
MSSA1942	NEG	NEG	NEG	NEG	NEG	NEG	NEG	NEG	NEG	NEG	NEG	NEG	NEG	NEG	NEG	NEG	NEG
MSSA1957	NEG	NEG	NEG	NEG	NEG	NEG	NEG	NEG	NEG	NEG	NEG	NEG	NEG	NEG	NEG	NEG	NEG
MSSA1748	NEG	NEG	NEG	NEG	NEG	NEG	NEG	NEG	NEG	NEG	NEG	NEG	NEG	NEG	NEG	NEG	NEG
MSSA1754	NEG	NEG	NEG	NEG	NEG	NEG	NEG	NEG	NEG	NEG	NEG	NEG	NEG	NEG	NEG	NEG	NEG
MSSA1770	NEG	NEG	NEG	NEG	NEG	NEG	NEG	NEG	NEG	NEG	NEG	NEG	NEG	NEG	NEG	NEG	NEG
MSSA1788	NEG	NEG	NEG	NEG	NEG	NEG	NEG	NEG	NEG	NEG	NEG	NEG	NEG	NEG	NEG	NEG	NEG
MSSA1804	NEG	NEG	NEG	NEG	NEG	NEG	NEG	NEG	NEG	NEG	NEG	NEG	NEG	NEG	NEG	NEG	NEG
MSSA1818	NEG	NEG	NEG	NEG	NEG	NEG	NEG	NEG	NEG	NEG	NEG	NEG	NEG	NEG	NEG	NEG	NEG
MSSA1914	NEG	NEG	NEG	NEG	NEG	NEG	NEG	NEG	NEG	NEG	NEG	NEG	NEG	NEG	NEG	NEG	NEG
MSSA1974	NEG	NEG	POS	NEG	NEG	NEG	NEG	NEG	NEG	NEG	NEG	NEG	NEG	NEG	NEG	NEG	NEG
MSSA2023	NEG	NEG	NEG	NEG	NEG	NEG	NEG	NEG	NEG	NEG	NEG	NEG	NEG	NEG	NEG	NEG	NEG
MSSA2030	NEG	NEG	NEG	NEG	NEG	NEG	NEG	NEG	NEG	NEG	NEG	NEG	NEG	NEG	NEG	NEG	NEG
MSSA2096	NEG	NEG	NEG	NEG	NEG	NEG	NEG	NEG	NEG	NEG	NEG	NEG	NEG	NEG	NEG	NEG	NEG
MSSA2142	NEG	NEG	NEG	NEG	NEG	NEG	NEG	NEG	NEG	NEG	NEG	NEG	NEG	NEG	NEG	NEG	NEG
MSSA2203	NEG	NEG	NEG	NEG	NEG	NEG	NEG	NEG	NEG	NEG	NEG	NEG	NEG	NEG	NEG	NEG	NEG
MSSA1901	NEG	NEG	NEG	NEG	NEG	NEG	NEG	NEG	NEG	NEG	NEG	NEG	NEG	NEG	NEG	NEG	NEG
MSSA2013	NEG	NEG	NEG	NEG	NEG	NEG	NEG	NEG	NEG	NEG	NEG	NEG	NEG	NEG	NEG	NEG	NEG
MSSA2031	NEG	NEG	NEG	NEG	NEG	NEG	NEG	NEG	NEG	NEG	NEG	NEG	NEG	NEG	NEG	NEG	NEG
MSSA2052	NEG	NEG	NEG	NEG	NEG	NEG	NEG	NEG	NEG	NEG	NEG	NEG	NEG	NEG	NEG	NEG	NEG
MSSA2057	NEG	NEG	NEG	NEG	NEG	NEG	NEG	NEG	NEG	NEG	NEG	NEG	NEG	NEG	NEG	NEG	NEG
MSSA2081	NEG	NEG	NEG	NEG	NEG	NEG	NEG	NEG	NEG	NEG	NEG	NEG	NEG	NEG	NEG	NEG	NEG
MSSA1955	NEG	NEG	NEG	NEG	NEG	NEG	NEG	NEG	NEG	NEG	NEG	NEG	NEG	NEG	NEG	NEG	NEG
MSSA1941	NEG	NEG	NEG	NEG	NEG	NEG	NEG	NEG	NEG	NEG	NEG	NEG	NEG	NEG	NEG	NEG	NEG
MRSA3838	NEG	NEG	NEG	NEG	NEG	NEG	NEG	NEG	NEG	NEG	NEG	NEG	NEG	NEG	NEG	NEG	NEG
MRSA3839	NEG	NEG	NEG	NEG	NEG	NEG	NEG	NEG	NEG	NEG	NEG	NEG	NEG	NEG	NEG	NEG	NEG
MSSA1801	NEG	NEG	NEG	NEG	NEG	NEG	NEG	NEG	NEG	NEG	NEG	NEG	NEG	NEG	NEG	NEG	NEG
MSSA2131	NEG	NEG	NEG	NEG	NEG	NEG	NEG	NEG	NEG	NEG	NEG	NEG	NEG	NEG	NEG	NEG	NEG
MSSA1792	NEG	NEG	NEG	NEG	NEG	NEG	NEG	NEG	NEG	NEG	NEG	NEG	NEG	NEG	NEG	NEG	AMB
MSSA1796	NEG	NEG	NEG	NEG	NEG	NEG	NEG	NEG	NEG	NEG	NEG	NEG	NEG	NEG	NEG	NEG	NEG
MSSA1817	NEG	NEG	NEG	NEG	NEG	NEG	NEG	NEG	NEG	NEG	NEG	NEG	NEG	NEG	NEG	NEG	NEG
MSSA1911	NEG	NEG	NEG	NEG	NEG	NEG	NEG	NEG	NEG	NEG	NEG	NEG	NEG	NEG	NEG	NEG	NEG
MSSA1927	NEG	NEG	NEG	NEG	NEG	NEG	NEG	NEG	NEG	NEG	NEG	NEG	NEG	NEG	NEG	NEG	NEG
MSSA1928	NEG	NEG	NEG	NEG	NEG	NEG	NEG	NEG	NEG	NEG	NEG	NEG	NEG	NEG	NEG	NEG	NEG
MSSA1953	NEG	NEG	NEG	NEG	NEG	NEG	NEG	NEG	NEG	NEG	NEG	NEG	NEG	NEG	NEG	NEG	NEG
MSSA2062	NEG	NEG	NEG	NEG	NEG	NEG	NEG	NEG	NEG	NEG	NEG	NEG	NEG	NEG	NEG	NEG	NEG
MSSA2078	NEG	NEG	NEG	NEG	NEG	NEG	NEG	NEG	NEG	NEG	NEG	NEG	NEG	NEG	NEG	NEG	NEG
MSSA2136	NEG	NEG	NEG	NEG	NEG	NEG	NEG	NEG	NEG	NEG	NEG	NEG	NEG	NEG	NEG	NEG	NEG

	RESISTANCE: MISCELLANEOUS GENES															
Strain	sat	dfrA	far1	Q6GD50	mupR	tet(K)	tet(M)	tetEfflux	cat (pC221)	cat (pC223)	cat (pMC524)	cat (pSBK203R)	cfr	fexA	fosB	fosB (plasmid)
MSSA1805	NEG	NEG	NEG	NEG	NEG	NEG	NEG	POS	NEG	NEG	NEG	NEG	NEG	NEG	NEG	NEG
MSSA1823	NEG	NEG	NEG	NEG	NEG	NEG	NEG	POS	NEG	NEG	NEG	NEG	NEG	NEG	NEG	NEG
MSSA1837	NEG	NEG	NEG	NEG	NEG	NEG	NEG	POS	NEG	NEG	NEG	NEG	NEG	NEG	NEG	NEG
MSSA2044	NEG	NEG	NEG	NEG	NEG	NEG	POS	POS	NEG	NEG	NEG	NEG	NEG	NEG	NEG	NEG
MSSA2046	NEG	NEG	NEG	NEG	NEG	NEG	NEG	POS	NEG	NEG	NEG	NEG	NEG	NEG	NEG	NEG
MSSA2059	NEG	NEG	NEG	NEG	NEG	NEG	NEG	POS	NEG	NEG	NEG	NEG	NEG	NEG	NEG	NEG
MSSA2076	NEG	NEG	NEG	NEG	NEG	NEG	NEG	POS	NEG	NEG	NEG	NEG	NEG	NEG	NEG	NEG
MSSA2200	NEG	NEG	NEG	NEG	NEG	NEG	NEG	POS	NEG	NEG	NEG	NEG	NEG	NEG	NEG	NEG
MRSA3797	NEG	NEG	NEG	NEG	NEG	NEG	NEG	POS	NEG	NEG	NEG	NEG	NEG	NEG	POS	NEG
MRSA3798	NEG	NEG	NEG	NEG	NEG	NEG	NEG	POS	NEG	NEG	NEG	NEG	NEG	NEG	POS	NEG
MRSA3799	NEG	NEG	NEG	NEG	NEG	NEG	NEG	POS	NEG	NEG	NEG	NEG	NEG	NEG	POS	NEG
MRSA3800	NEG	NEG	NEG	NEG	NEG	NEG	NEG	POS	NEG	NEG	NEG	NEG	NEG	NEG	POS	NEG
MRSA3801	NEG	NEG	NEG	NEG	NEG	NEG	NEG	POS	NEG	NEG	NEG	NEG	NEG	NEG	POS	NEG
MRSA3804	NEG	NEG	NEG	NEG	NEG	NEG	NEG	POS	NEG	NEG	NEG	NEG	NEG	NEG	POS	NEG
MRSA3806	NEG	NEG	NEG	NEG	NEG	NEG	NEG	POS	NEG	NEG	NEG	NEG	NEG	NEG	POS	NEG
MRSA3961	NEG	NEG	NEG	NEG	NEG	NEG	NEG	POS	NEG	NEG	NEG	NEG	NEG	NEG	POS	NEG
MRSA3962	NEG	POS	NEG	NEG	NEG	NEG	NEG	POS	NEG	NEG	NEG	NEG	NEG	NEG	POS	NEG
MRSA3963	NEG	NEG	NEG	NEG	NEG	NEG	NEG	POS	NEG	NEG	NEG	NEG	NEG	NEG	POS	NEG
MRSA3966	NEG	NEG	NEG	NEG	NEG	NEG	NEG	POS	NEG	NEG	NEG	NEG	NEG	NEG	POS	NEG
MRSA3978	NEG	NEG	NEG	NEG	NEG	NEG	NEG	POS	NEG	NEG	NEG	NEG	NEG	NEG	POS	NEG
MRSA3979	NEG	NEG	NEG	NEG	NEG	NEG	NEG	POS	NEG	NEG	NEG	NEG	NEG	NEG	POS	NEG
MRSA3980	NEG	NEG	NEG	NEG	NEG	NEG	NEG	POS	NEG	NEG	NEG	NEG	NEG	NEG	POS	NEG
MRSA4179	NEG	NEG	NEG	NEG	NEG	NEG	NEG	POS	NEG	NEG	NEG	NEG	NEG	NEG	POS	NEG
MRSA4216	NEG	NEG	NEG	NEG	NEG	NEG	NEG	POS	NEG	NEG	NEG	NEG	NEG	NEG	POS	NEG
MRSA4217	NEG	NEG	NEG	NEG	NEG	NEG	NEG	POS	NEG	NEG	NEG	NEG	NEG	NEG	POS	NEG
MRSA4220	NEG	NEG	NEG	NEG	NEG	NEG	NEG	POS	NEG	NEG	NEG	NEG	NEG	NEG	POS	NEG
MRSA4221	NEG	NEG	NEG	NEG	NEG	NEG	POS	POS	NEG	NEG	NEG	NEG	NEG	NEG	POS	NEG
MRSA4222	NEG	NEG	NEG	NEG	NEG	NEG	NEG	POS	NEG	NEG	NEG	NEG	NEG	NEG	POS	NEG
MSSA1751	NEG	NEG	NEG	NEG	NEG	NEG	NEG	POS	NEG	NEG	NEG	NEG	NEG	NEG	POS	NEG
MSSA1760	NEG	NEG	NEG	NEG	NEG	NEG	NEG	POS	NEG	NEG	NEG	NEG	NEG	NEG	POS	NEG
MSSA1802	NEG	NEG	NEG	NEG	NEG	NEG	NEG	POS	NEG	NEG	NEG	NEG	NEG	NEG	POS	NEG
MSSA1809	NEG	POS	NEG	NEG	NEG	NEG	NEG	POS	NEG	NEG	NEG	NEG	NEG	NEG	POS	NEG
MSSA1815	NEG	NEG	NEG	NEG	NEG	NEG	NEG	POS	NEG	NEG	NEG	NEG	NEG	NEG	POS	NEG
MSSA1829	NEG	NEG	NEG	NEG	NEG	NEG	NEG	POS	NEG	NEG	NEG	NEG	NEG	NEG	POS	NEG
MSSA1908	NEG	NEG	NEG	NEG	NEG	NEG	NEG	POS	NEG	NEG	NEG	NEG	NEG	NEG	POS	NEG
MSSA1960	NEG	NEG	NEG	NEG	NEG	NEG	NEG	POS	NEG	NEG	NEG	NEG	NEG	NEG	POS	NEG
MSSA1965	NEG	NEG	NEG	NEG	NEG	NEG	NEG	POS	NEG	NEG	NEG	NEG	NEG	NEG	POS	NEG
MSSA1977	NEG	NEG	NEG	NEG	NEG	NEG	NEG	POS	NEG	NEG	NEG	NEG	NEG	NEG	POS	NEG
MSSA1978	NEG	NEG	NEG	NEG	NEG	NEG	NEG	POS	NEG	NEG	NEG	NEG	NEG	NEG	POS	NEG
MSSA1999	NEG	NEG	NEG	NEG	NEG	NEG	POS	POS	NEG	NEG	NEG	NEG	NEG	POS	POS	NEG
MSSA2003	NEG	NEG	NEG	NEG	NEG	NEG	NEG	POS	NEG	NEG	NEG	NEG	NEG	NEG	POS	NEG
MSSA2006	NEG	NEG	NEG	NEG	NEG	NEG	NEG	POS	NEG	NEG	NEG	NEG	NEG	NEG	POS	NEG
MSSA2008	NEG	NEG	NEG	NEG	NEG	NEG	NEG	POS	NEG	NEG	NEG	NEG	NEG	NEG	POS	NEG
MSSA2011	NEG	NEG	NEG	NEG	NEG	NEG	NEG	POS	POS	NEG	NEG	NEG	NEG	NEG	POS	NEG
MSSA2017	NEG	NEG	NEG	NEG	NEG	NEG	NEG	POS	NEG	NEG	NEG	NEG	NEG	NEG	POS	NEG
MSSA2022	NEG	NEG	NEG	NEG	NEG	NEG	NEG	POS	NEG	NEG	NEG	NEG	NEG	NEG	POS	NEG
MSSA2055	NEG	NEG	NEG	NEG	NEG	NEG	NEG	POS	NEG	NEG	NEG	NEG	NEG	NEG	POS	NEG
MSSA2060	NEG	NEG	NEG	NEG	NEG	NEG	NEG	POS	NEG	NEG	NEG	NEG	NEG	NEG	POS	NEG
MSSA2080	NEG	NEG	NEG	NEG	NEG	NEG	NEG	POS	NEG	NEG	NEG	NEG	NEG	NEG	POS	NEG
MSSA2082	NEG	NEG	NEG	NEG	NEG	NEG	NEG	POS	NEG	NEG	NEG	NEG	NEG	NEG	POS	NEG
MSSA2084	NEG	NEG	NEG	NEG	NEG	NEG	POS	POS	NEG	NEG	NEG	NEG	NEG	NEG	POS	NEG
MSSA2087	NEG	NEG	NEG	NEG	NEG	NEG	NEG	POS	NEG	NEG	NEG	NEG	NEG	NEG	POS	NEG
MSSA2094	NEG	NEG	NEG	NEG	NEG	NEG	NEG	POS	NEG	NEG	NEG	NEG	NEG	NEG	POS	NEG
MSSA2097	NEG	NEG	NEG	NEG	NEG	NEG	NEG	POS	NEG	NEG	NEG	NEG	NEG	NEG	POS	NEG
MSSA2110	NEG	NEG	NEG	NEG	NEG	NEG	NEG	POS	NEG	NEG	NEG	NEG	NEG	NEG	POS	NEG
MSSA2113	NEG	NEG	NEG	NEG	NEG	NEG	NEG	POS	NEG	NEG	NEG	NEG	NEG	NEG	POS	NEG
MSSA2132	NEG	NEG	NEG	NEG	NEG	NEG	NEG	POS	NEG	NEG	NEG	NEG	NEG	NEG	POS	NEG
MSSA2134	NEG	NEG	NEG	NEG	NEG	NEG	NEG	POS	NEG	NEG	NEG	NEG	NEG	NEG	POS	NEG
MSSA1746	NEG	NEG	NEG	NEG	NEG	NEG	NEG	POS	NEG	NEG	NEG	NEG	NEG	NEG	NEG	NEG
MSSA1757	NEG	NEG	NEG	NEG	NEG	NEG	NEG	POS	NEG	NEG	NEG	NEG	NEG	NEG	NEG	NEG
MSSA1772	NEG	NEG	NEG	NEG	NEG	NEG	NEG	POS	NEG	NEG	NEG	NEG	NEG	NEG	NEG	NEG
MSSA1795	NEG	NEG	NEG	NEG	NEG	NEG	NEG	POS	NEG	NEG	NEG	NEG	NEG	NEG	NEG	NEG
MSSA1813	NEG	NEG	NEG	NEG	NEG	NEG	NEG	POS	NEG	NEG	NEG	NEG	NEG	NEG	NEG	NEG
MSSA1826	NEG	NEG	NEG	NEG	NEG	NEG	NEG	POS	NEG	NEG	NEG	NEG	NEG	NEG	NEG	NEG
MSSA1830	NEG	NEG	NEG	NEG	NEG	NEG	NEG	POS	NEG	NEG	NEG	NEG	NEG	NEG	NEG	NEG
MSSA1838	NEG	NEG	NEG	NEG	NEG	NEG	NEG	POS	NEG	NEG	NEG	NEG	NEG	NEG	NEG	NEG
MSSA1944	NEG	NEG	NEG	NEG	NEG	NEG	NEG	POS	NEG	NEG	NEG	NEG	NEG	NEG	NEG	NEG
MSSA1947	NEG	NEG	NEG	NEG	NEG	NEG	NEG	POS	NEG	NEG	NEG	NEG	NEG	NEG	NEG	NEG
MSSA1964	NEG	NEG	NEG	NEG	NEG	NEG	NEG	POS	NEG	NEG	NEG	NEG	NEG	NEG	NEG	NEG
MSSA1969	NEG	NEG	NEG	NEG	NEG	NEG	NEG	POS	NEG	NEG	NEG	NEG	NEG	NEG	NEG	NEG
MSSA2001	NEG	NEG	NEG	NEG	NEG	NEG	NEG	POS	NEG	NEG	NEG	NEG	NEG	NEG	NEG	NEG
MSSA2004	NEG	NEG	NEG	NEG	NEG	NEG	NEG	POS	NEG	NEG	NEG	NEG	NEG	NEG	NEG	NEG
MSSA2024	NEG	NEG	NEG	NEG	NEG	NEG	NEG	POS	NEG	NEG	NEG	NEG	NEG	NEG	NEG	NEG
MSSA2025	NEG	NEG	NEG	NEG	NEG	NEG	NEG	POS	NEG	NEG	NEG	NEG	NEG	NEG	NEG	NEG
MSSA2026	NEG	NEG	NEG	NEG	NEG	NEG	NEG	POS	NEG	NEG	NEG	NEG	NEG	NEG	NEG	NEG
MSSA2032	NEG	NEG	NEG	NEG	NEG	NEG	NEG	POS	NEG	NEG	NEG	NEG	NEG	NEG	NEG	NEG
MSSA2043	NEG	NEG	NEG	NEG	POS	NEG	NEG	POS	NEG	NEG	NEG	NEG	NEG	NEG	NEG	NEG
MSSA2061	NEG	NEG	NEG	NEG	NEG	NEG	NEG	POS	NEG	NEG	NEG	NEG	NEG	NEG	NEG	NEG
MSSA2085	NEG	NEG	NEG	NEG	NEG	NEG	NEG	POS	NEG	NEG	NEG	NEG	NEG	NEG	NEG	NEG
MSSA2098	NEG	NEG	NEG	NEG	NEG	NEG	NEG	POS	NEG	NEG	NEG	NEG	NEG	NEG	NEG	NEG
MSSA2100	NEG	NEG	NEG	NEG	NEG	NEG	NEG	POS	NEG	NEG	NEG	NEG	NEG	NEG	NEG	NEG
MSSA2111	NEG	NEG	NEG	NEG	NEG	NEG	NEG	POS	NEG	NEG	NEG	NEG	NEG	NEG	NEG	NEG
MSSA2178	NEG	NEG	NEG	NEG	NEG	NEG	NEG	POS	NEG	NEG	NEG	NEG	NEG	NEG	NEG	NEG
MSSA2202	NEG	NEG	NEG	NEG	NEG	NEG	NEG	POS	NEG	NEG	NEG	NEG	NEG	NEG	NEG	NEG
MSSA2205	NEG	NEG	NEG	NEG	NEG	NEG	NEG	POS	NEG	NEG	NEG	NEG	NEG	NEG	NEG	NEG
MRSA3796	NEG	NEG	NEG	NEG	NEG	NEG	NEG	POS	NEG	NEG	NEG	NEG	NEG	NEG	POS	NEG
MRSA3805	NEG	NEG	NEG	NEG	NEG	NEG	POS	POS	NEG	NEG	NEG	NEG	NEG	NEG	POS	NEG
MRSA3965	NEG	NEG	NEG	NEG	NEG	NEG	NEG	POS	NEG	NEG	NEG	NEG	NEG	NEG	POS	NEG
MSSA1761	NEG	NEG	NEG	POS	NEG	POS	NEG	POS	NEG	NEG	NEG	NEG	NEG	NEG	POS	AMB
MSSA1764	NEG	NEG	NEG	NEG	NEG	NEG	NEG	POS	NEG	NEG	NEG	NEG	NEG	NEG	POS	NEG
MSSA1765	NEG	NEG	NEG	NEG	NEG	NEG	NEG	POS	NEG	NEG	NEG	NEG	NEG	NEG	POS	NEG
MSSA1784	NEG	NEG	NEG	NEG	NEG	NEG	NEG	POS	NEG	NEG	NEG	NEG	NEG	NEG	POS	NEG
MSSA1806	NEG	NEG	NEG	NEG	NEG	POS	NEG	POS	NEG	NEG	NEG	NEG	NEG	NEG	POS	NEG
MSSA1807	NEG	NEG	NEG	NEG	NEG	NEG	NEG	POS	NEG	NEG	NEG	NEG	NEG	NEG	POS	NEG
MSSA1888	NEG	NEG	NEG	NEG	NEG	NEG	POS	POS	NEG	NEG	NEG	NEG	NEG	NEG	POS	NEG
MSSA1894	NEG	NEG	NEG	NEG	NEG	NEG	NEG	POS	NEG	NEG	NEG	NEG	NEG	NEG	POS	NEG
MSSA1922	NEG	NEG	NEG	NEG	NEG	NEG	NEG	POS	NEG	NEG	NEG	NEG	NEG	NEG	POS	NEG
MSSA1950	NEG	NEG	NEG	NEG	NEG	NEG	NEG	POS	NEG	NEG	NEG	NEG	NEG	NEG	POS	NEG
MSSA1954	NEG	POS	NEG	NEG	NEG	NEG	NEG	POS	NEG	NEG	NEG	NEG	NEG	NEG	POS	NEG
MSSA1966	NEG	NEG	NEG	NEG	NEG	NEG	POS	POS	NEG	NEG	NEG	NEG	NEG	NEG	POS	NEG
MSSA1972	NEG	NEG	NEG	NEG	NEG	NEG	NEG	POS	NEG	NEG	NEG	NEG	NEG	NEG	POS	NEG
MSSA2010	NEG	NEG	NEG	NEG	NEG	NEG	NEG	POS	NEG	NEG	NEG	NEG	NEG	NEG	POS	NEG
MSSA2020	NEG	NEG	NEG	NEG	NEG	NEG	NEG	POS	NEG	NEG	NEG	NEG	NEG	NEG	POS	NEG
MSSA2028	NEG	NEG	NEG	NEG	NEG	NEG	NEG	POS	NEG	NEG	NEG	NEG	NEG	NEG	POS	AMB
MSSA2088	NEG	NEG	NEG	NEG	NEG	NEG	NEG	POS	NEG	NEG	NEG	NEG	NEG	NEG	POS	NEG
MSSA2187	NEG	NEG	NEG	POS	NEG	NEG	NEG	POS	NEG	NEG	NEG	NEG	NEG	NEG	POS	NEG
MSSA2204	NEG	NEG	NEG	NEG	NEG	NEG	NEG	POS	NEG	NEG	NEG	NEG	NEG	NEG	POS	NEG
MSSA2210	NEG	NEG	NEG	NEG	NEG	NEG	NEG	POS	NEG	NEG	NEG	NEG	NEG	NEG	POS	NEG
MSSA2223	NEG	NEG	NEG	NEG	NEG	NEG	NEG	POS	NEG	NEG	NEG	NEG	NEG	NEG	POS	NEG
MSSA1763	NEG	NEG	NEG	NEG	NEG	NEG	NEG	POS	NEG	NEG	NEG	NEG	NEG	NEG	POS	NEG
MSSA1777	NEG	NEG	NEG	NEG	NEG	NEG	NEG	POS	NEG	NEG	NEG	NEG	NEG	NEG	POS	POS
MSSA2009	NEG	NEG	NEG	NEG	NEG	NEG	NEG	POS	NEG	NEG	NEG	NEG	NEG	NEG	POS	NEG
MSSA2047	NEG	NEG	NEG	NEG	NEG	NEG	NEG	POS	NEG	NEG	NEG	NEG	NEG	NEG	POS	NEG
MSSA2079	NEG	NEG	NEG	NEG	NEG	NEG	NEG	POS	NEG	NEG	NEG	NEG	NEG	NEG	POS	NEG
MSSA2101	NEG	NEG	NEG	NEG	NEG	NEG	NEG	POS	NEG	NEG	NEG	NEG	NEG	NEG	POS	NEG
MSSA1821	NEG	NEG	NEG	NEG	NEG	NEG	NEG	POS	NEG	NEG	NEG	NEG	NEG	NEG	POS	NEG
MSSA1898	NEG	NEG	NEG	NEG	NEG	NEG	NEG	POS	NEG	NEG	NEG	NEG	NEG	NEG	POS	NEG
MSSA1973	NEG	NEG	NEG	NEG	NEG	NEG	NEG	POS	NEG	NEG	NEG	NEG	NEG	NEG	POS	NEG
MSSA2071	NEG	NEG	NEG	NEG	NEG	NEG	NEG	POS	NEG	NEG	NEG	NEG	NEG	NEG	POS	NEG
MSSA2083	NEG	NEG	NEG	NEG	NEG	NEG	NEG	POS	NEG	NEG	NEG	NEG	NEG	NEG	POS	NEG
MSSA2106	NEG	NEG	NEG	NEG	NEG	NEG	NEG	POS	NEG	NEG	NEG	NEG	NEG	NEG	POS	NEG
MSSA2112	NEG	NEG	NEG	NEG	NEG	NEG	NEG	POS	NEG	NEG	NEG	NEG	NEG	NEG	POS	NEG
MSSA2115	NEG	NEG	NEG	NEG	NEG	NEG	NEG	POS	NEG	NEG	NEG	NEG	NEG	NEG	POS	NEG
MSSA2195	NEG	NEG	NEG	NEG	NEG	POS	NEG	POS	NEG	NEG	NEG	NEG	NEG	NEG	POS	NEG
MSSA2207	NEG	NEG	NEG	NEG	AMB	NEG	NEG	POS	NEG	NEG	NEG	NEG	NEG	NEG	POS	NEG
MSSA1758	NEG	NEG	NEG	NEG	NEG	NEG	NEG	POS	NEG	NEG	NEG	NEG	NEG	NEG	POS	NEG
MSSA1762	NEG	NEG	NEG	NEG	NEG	NEG	NEG	POS	NEG	NEG	NEG	NEG	NEG	NEG	POS	NEG
MSSA1769	NEG	NEG	NEG	NEG	NEG	NEG	NEG	POS	NEG	NEG	NEG	NEG	NEG	NEG	POS	NEG
MSSA1771	NEG	NEG	NEG	NEG	NEG	POS	NEG	POS	NEG	NEG	NEG	NEG	NEG	NEG	POS	NEG
MSSA1789	NEG	NEG	NEG	NEG	NEG	NEG	NEG	POS	NEG	NEG	NEG	NEG	NEG	NEG	POS	NEG
MSSA1800	NEG	NEG	NEG	NEG	NEG	NEG	NEG	POS	NEG	NEG	NEG	NEG	NEG	NEG	POS	NEG
MSSA1833	NEG	NEG	NEG	NEG	NEG	NEG	NEG	POS	NEG	NEG	NEG	NEG	NEG	NEG	POS	NEG
MSSA1890	NEG	NEG	NEG	NEG	NEG	NEG	NEG	POS	NEG	NEG	NEG	NEG	NEG	NEG	POS	NEG
MSSA1903	NEG	NEG	NEG	NEG	NEG	NEG	NEG	POS	NEG	NEG	NEG	NEG	NEG	NEG	POS	NEG
MSSA1940	NEG	NEG	NEG	NEG	NEG	NEG	NEG	POS	NEG	NEG	NEG	NEG	NEG	NEG	POS	NEG
MSSA1962	NEG	NEG	NEG	NEG	NEG	POS	NEG	POS	NEG	NEG	NEG	NEG	NEG	NEG	POS	NEG
MSSA1963	NEG	NEG	NEG	NEG	NEG	POS	NEG	POS	NEG	NEG	NEG	NEG	NEG	NEG	POS	NEG
MSSA2016	NEG	NEG	NEG	NEG	NEG	NEG	NEG	POS	NEG	NEG	NEG	NEG	NEG	NEG	POS	NEG
MSSA2018	NEG	NEG	NEG	NEG	NEG	NEG	NEG	POS	NEG	NEG	NEG	NEG	NEG	NEG	POS	NEG
MSSA2019	NEG	NEG	NEG	NEG	NEG	NEG	NEG	POS	NEG	NEG	NEG	NEG	NEG	NEG	POS	NEG
MSSA2029	NEG	NEG	NEG	NEG	NEG	NEG	NEG	POS	NEG	NEG	NEG	NEG	NEG	NEG	POS	NEG
MSSA2045	NEG	NEG	NEG	NEG	NEG	NEG	NEG	POS	NEG	NEG	NEG	NEG	NEG	NEG	POS	NEG
MSSA2048	NEG	NEG	NEG	NEG	NEG	NEG	NEG	POS	NEG	NEG	NEG	NEG	NEG	NEG	POS	NEG
MSSA2049	NEG	NEG	NEG	NEG	NEG	NEG	NEG	POS	NEG	NEG	NEG	NEG	NEG	NEG	POS	NEG
MSSA2050	NEG	NEG	NEG	NEG	NEG	NEG	NEG	POS	NEG	NEG	NEG	NEG	NEG	NEG	POS	NEG
MSSA2099	NEG	NEG	NEG	NEG	NEG	NEG	NEG	POS	NEG	NEG	NEG	NEG	NEG	NEG	POS	NEG
MSSA2109	NEG	NEG	NEG	NEG	NEG	NEG	NEG	POS	NEG	NEG	NEG	NEG	NEG	NEG	POS	NEG
MSSA2130	NEG	NEG	NEG	NEG	NEG	NEG	NEG	POS	NEG	NEG	NEG	NEG	NEG	NEG	POS	NEG
MSSA2133	NEG	NEG	NEG	NEG	NEG	NEG	NEG	POS	NEG	NEG	NEG	NEG	NEG	NEG	POS	NEG
MSSA2139	NEG	NEG	NEG	NEG	NEG	POS	NEG	POS	NEG	NEG	NEG	NEG	NEG	NEG	POS	NEG
MSSA2141	NEG	NEG	NEG	NEG	NEG	NEG	NEG	POS	NEG	NEG	NEG	NEG	NEG	NEG	POS	NEG
MSSA2208	NEG	NEG	NEG	NEG	NEG	NEG	NEG	POS	NEG	NEG	NEG	NEG	NEG	NEG	POS	NEG
MSSA2209	NEG	NEG	NEG	NEG	NEG	NEG	NEG	POS	NEG	NEG	NEG	NEG	NEG	NEG	POS	NEG
MSSA1893	NEG	NEG	NEG	NEG	NEG	NEG	NEG	POS	NEG	NEG	NEG	NEG	NEG	NEG	POS	NEG
MSSA2105	NEG	NEG	NEG	NEG	NEG	NEG	NEG	POS	NEG	NEG	NEG	NEG	NEG	NEG	POS	NEG
MRSA3836	NEG	NEG	NEG	NEG	NEG	NEG	NEG	NEG	NEG	NEG	NEG	NEG	NEG	NEG	NEG	NEG
MRSA3840	NEG	NEG	NEG	NEG	NEG	NEG	NEG	NEG	NEG	NEG	NEG	NEG	NEG	NEG	NEG	NEG
MRSA4188	NEG	NEG	NEG	NEG	NEG	NEG	NEG	NEG	NEG	NEG	NEG	NEG	NEG	NEG	NEG	NEG
MSSA1814	NEG	NEG	NEG	NEG	NEG	NEG	NEG	NEG	NEG	NEG	NEG	NEG	NEG	NEG	NEG	NEG
MSSA1816	NEG	NEG	NEG	NEG	NEG	NEG	NEG	NEG	NEG	NEG	NEG	NEG	NEG	NEG	NEG	NEG
MSSA1825	NEG	NEG	NEG	NEG	NEG	NEG	NEG	NEG	NEG	NEG	NEG	NEG	NEG	NEG	NEG	NEG
MSSA1834	NEG	NEG	NEG	NEG	NEG	NEG	NEG	NEG	NEG	NEG	NEG	NEG	NEG	NEG	NEG	NEG
MSSA1938	NEG	NEG	NEG	NEG	NEG	NEG	NEG	NEG	NEG	NEG	NEG	NEG	NEG	NEG	NEG	NEG
MSSA1948	NEG	NEG	NEG	NEG	NEG	NEG	NEG	NEG	NEG	NEG	NEG	NEG	NEG	NEG	NEG	NEG
MSSA1951	NEG	NEG	NEG	NEG	NEG	NEG	NEG	NEG	NEG	NEG	NEG	NEG	NEG	NEG	NEG	NEG
MSSA2005	NEG	NEG	NEG	NEG	NEG	NEG	NEG	NEG	NEG	NEG	NEG	NEG	NEG	NEG	NEG	NEG
MSSA2051	NEG	NEG	NEG	NEG	NEG	NEG	NEG	NEG	NEG	NEG	NEG	NEG	NEG	NEG	NEG	NEG
MSSA2056	NEG	NEG	NEG	NEG	NEG	NEG	NEG	NEG	NEG	NEG	NEG	NEG	NEG	NEG	NEG	NEG
MSSA2066	NEG	NEG	NEG	NEG	NEG	NEG	NEG	NEG	NEG	NEG	NEG	NEG	NEG	NEG	NEG	NEG
MSSA2067	NEG	NEG	NEG	NEG	NEG	NEG	NEG	NEG	NEG	NEG	NEG	NEG	NEG	NEG	NEG	NEG
MSSA2086	NEG	NEG	NEG	NEG	NEG	NEG	NEG	NEG	NEG	NEG	NEG	NEG	NEG	NEG	NEG	NEG
MSSA2089	NEG	NEG	AMB	NEG	NEG	NEG	NEG	NEG	NEG	NEG	NEG	NEG	NEG	NEG	NEG	NEG
MSSA2103	NEG	NEG	NEG	NEG	NEG	NEG	NEG	NEG	NEG	NEG	NEG	NEG	NEG	NEG	NEG	NEG
MSSA2173	NEG	NEG	NEG	NEG	NEG	NEG	NEG	NEG	NEG	NEG	NEG	NEG	NEG	NEG	NEG	NEG
MSSA2183	NEG	NEG	NEG	NEG	NEG	NEG	NEG	NEG	NEG	NEG	NEG	NEG	NEG	NEG	NEG	NEG
MSSA2196	NEG	NEG	NEG	NEG	NEG	POS	NEG	NEG	NEG	NEG	NEG	NEG	NEG	NEG	NEG	NEG
MSSA2219	NEG	NEG	NEG	NEG	NEG	NEG	NEG	NEG	NEG	NEG	NEG	NEG	NEG	NEG	NEG	NEG
MSSA1753	NEG	NEG	NEG	NEG	NEG	NEG	NEG	POS	NEG	NEG	NEG	NEG	NEG	NEG	POS	NEG
MSSA1773	NEG	NEG	NEG	NEG	NEG	NEG	NEG	POS	NEG	NEG	NEG	NEG	NEG	NEG	POS	NEG
MSSA1811	NEG	NEG	NEG	NEG	NEG	NEG	NEG	POS	NEG	NEG	NEG	NEG	NEG	NEG	POS	NEG
MSSA1897	NEG	NEG	NEG	NEG	NEG	NEG	NEG	POS	NEG	NEG	NEG	NEG	NEG	NEG	POS	NEG
MSSA1931	NEG	NEG	NEG	NEG	NEG	NEG	NEG	POS	NEG	NEG	NEG	NEG	NEG	NEG	POS	NEG
MSSA2114	NEG	NEG	NEG	NEG	NEG	NEG	NEG	POS	NEG	NEG	NEG	NEG	NEG	NEG	POS	NEG
MSSA1745	NEG	NEG	NEG	NEG	NEG	NEG	NEG	POS	NEG	NEG	NEG	NEG	NEG	NEG	POS	NEG
MSSA1747	NEG	NEG	NEG	NEG	NEG	NEG	NEG	POS	NEG	NEG	NEG	NEG	NEG	NEG	POS	NEG
MSSA1749	NEG	NEG	NEG	NEG	NEG	NEG	NEG	POS	NEG	NEG	NEG	NEG	NEG	NEG	POS	NEG
MSSA1768	NEG	NEG	NEG	NEG	NEG	NEG	NEG	POS	NEG	NEG	NEG	NEG	NEG	NEG	POS	NEG
MSSA1774	NEG	NEG	NEG	NEG	NEG	NEG	NEG	POS	NEG	NEG	NEG	NEG	NEG	NEG	POS	NEG
MSSA1775	NEG	NEG	NEG	NEG	NEG	NEG	NEG	POS	NEG	NEG	NEG	NEG	NEG	NEG	POS	NEG
MSSA1776	NEG	NEG	NEG	NEG	NEG	NEG	NEG	POS	NEG	NEG	NEG	NEG	NEG	NEG	POS	NEG
MSSA1779	NEG	NEG	NEG	NEG	NEG	NEG	NEG	POS	NEG	NEG	NEG	NEG	NEG	NEG	POS	NEG
MSSA1780	NEG	NEG	NEG	NEG	NEG	NEG	NEG	POS	NEG	NEG	NEG	NEG	NEG	NEG	POS	NEG

	RESISTANCE: MISCELLANEOUS GENES															
Strain	sat	dfrA	far1	Q6GD50	mupR	tet(K)	tet(M)	tetEfflux	cat (pC221)	cat (pC223)	cat (pMC524)	cat (pSBK203R)	cfr	fexA	fosB	fosB (plasmid)
MSSA1781	NEG	NEG	NEG	NEG	NEG	NEG	NEG	POS	NEG	NEG	NEG	NEG	NEG	NEG	POS	NEG
MSSA1786	NEG	NEG	NEG	NEG	NEG	NEG	NEG	POS	NEG	NEG	NEG	NEG	NEG	NEG	POS	NEG
MSSA1793	NEG	NEG	NEG	NEG	NEG	NEG	NEG	POS	NEG	NEG	NEG	NEG	NEG	NEG	POS	NEG
MSSA1803	NEG	NEG	NEG	NEG	NEG	NEG	NEG	POS	NEG	NEG	NEG	NEG	NEG	NEG	POS	NEG
MSSA1836	NEG	NEG	NEG	NEG	NEG	NEG	NEG	POS	NEG	NEG	NEG	NEG	NEG	NEG	POS	NEG
MSSA1839	NEG	NEG	NEG	NEG	NEG	NEG	NEG	POS	NEG	NEG	NEG	NEG	NEG	NEG	POS	NEG
MSSA1900	NEG	NEG	NEG	NEG	NEG	NEG	NEG	POS	NEG	NEG	NEG	NEG	NEG	NEG	POS	NEG
MSSA1910	NEG	NEG	NEG	NEG	NEG	NEG	NEG	POS	NEG	NEG	NEG	NEG	NEG	NEG	POS	NEG
MSSA1918	NEG	NEG	NEG	NEG	NEG	NEG	NEG	POS	NEG	NEG	NEG	NEG	NEG	NEG	POS	NEG
MSSA1921	NEG	NEG	NEG	NEG	NEG	NEG	NEG	POS	NEG	NEG	NEG	NEG	NEG	NEG	POS	NEG
MSSA1923	NEG	NEG	NEG	NEG	NEG	NEG	POS	POS	NEG	NEG	NEG	NEG	NEG	NEG	POS	NEG
MSSA1981	NEG	NEG	NEG	NEG	NEG	NEG	NEG	POS	NEG	NEG	NEG	NEG	NEG	NEG	POS	NEG
MSSA1998	NEG	NEG	NEG	NEG	NEG	NEG	NEG	POS	NEG	NEG	NEG	NEG	NEG	NEG	POS	NEG
MSSA2000	NEG	NEG	NEG	NEG	NEG	NEG	NEG	POS	NEG	NEG	NEG	NEG	NEG	NEG	POS	NEG
MSSA2007	NEG	NEG	NEG	NEG	NEG	NEG	NEG	POS	NEG	NEG	NEG	NEG	NEG	NEG	POS	NEG
MSSA2012	NEG	NEG	NEG	NEG	NEG	NEG	NEG	POS	NEG	NEG	NEG	NEG	NEG	NEG	POS	NEG
MSSA2015	NEG	NEG	NEG	NEG	NEG	NEG	NEG	POS	NEG	NEG	NEG	NEG	NEG	NEG	POS	NEG
MSSA2021	NEG	NEG	NEG	NEG	NEG	NEG	NEG	POS	NEG	NEG	NEG	NEG	NEG	NEG	POS	NEG
MSSA2027	NEG	NEG	NEG	NEG	NEG	NEG	NEG	POS	NEG	NEG	NEG	NEG	NEG	NEG	POS	NEG
MSSA2063	NEG	NEG	NEG	NEG	NEG	NEG	NEG	POS	NEG	NEG	NEG	NEG	NEG	NEG	POS	NEG
MSSA2064	NEG	NEG	NEG	NEG	NEG	NEG	NEG	POS	NEG	NEG	NEG	NEG	NEG	NEG	POS	NEG
MSSA2065	NEG	NEG	NEG	NEG	NEG	NEG	NEG	POS	NEG	NEG	NEG	NEG	NEG	NEG	POS	NEG
MSSA2075	NEG	NEG	NEG	NEG	NEG	NEG	NEG	POS	NEG	NEG	NEG	NEG	NEG	NEG	POS	NEG
MSSA2093	NEG	NEG	NEG	NEG	NEG	NEG	NEG	POS	NEG	NEG	NEG	NEG	NEG	NEG	POS	NEG
MSSA2102	NEG	NEG	NEG	NEG	NEG	NEG	NEG	POS	NEG	NEG	NEG	NEG	NEG	NEG	POS	NEG
MSSA2138	NEG	NEG	NEG	NEG	NEG	NEG	NEG	POS	NEG	NEG	NEG	NEG	NEG	NEG	POS	NEG
MSSA2168	NEG	NEG	NEG	AMB	NEG	NEG	NEG	POS	NEG	NEG	NEG	NEG	NEG	NEG	POS	NEG
MSSA2181	NEG	NEG	NEG	NEG	NEG	NEG	NEG	POS	NEG	NEG	NEG	NEG	NEG	NEG	POS	NEG
MSSA2189	NEG	NEG	NEG	NEG	NEG	NEG	NEG	POS	NEG	NEG	NEG	NEG	NEG	NEG	POS	AMB
MSSA2214	NEG	NEG	NEG	NEG	NEG	NEG	NEG	POS	NEG	NEG	NEG	NEG	NEG	NEG	POS	NEG
MRSA3803	NEG	NEG	NEG	NEG	POS	NEG	NEG	POS	NEG	NEG	NEG	NEG	NEG	NEG	NEG	NEG
MSSA1750	NEG	NEG	NEG	NEG	NEG	NEG	NEG	POS	NEG	NEG	NEG	NEG	NEG	NEG	AMB	NEG
MSSA1756	NEG	NEG	NEG	NEG	NEG	NEG	NEG	POS	NEG	NEG	NEG	NEG	NEG	NEG	NEG	NEG
MSSA1759	NEG	NEG	NEG	NEG	NEG	NEG	NEG	POS	NEG	NEG	NEG	NEG	NEG	NEG	NEG	NEG
MSSA1767	NEG	NEG	NEG	NEG	NEG	NEG	NEG	POS	NEG	NEG	NEG	NEG	NEG	NEG	NEG	NEG
MSSA1787	NEG	NEG	NEG	NEG	NEG	NEG	NEG	POS	NEG	NEG	NEG	NEG	NEG	NEG	NEG	NEG
MSSA1790	NEG	NEG	NEG	NEG	NEG	NEG	NEG	POS	NEG	NEG	NEG	NEG	NEG	NEG	NEG	NEG
MSSA1794	NEG	NEG	NEG	NEG	NEG	NEG	NEG	POS	NEG	NEG	NEG	NEG	NEG	NEG	NEG	NEG
MSSA1808	NEG	NEG	NEG	NEG	NEG	NEG	NEG	POS	NEG	NEG	NEG	NEG	NEG	NEG	NEG	NEG
MSSA1812	NEG	NEG	NEG	NEG	NEG	NEG	NEG	POS	NEG	NEG	NEG	NEG	NEG	NEG	NEG	NEG
MSSA1819	NEG	NEG	NEG	NEG	NEG	NEG	NEG	POS	NEG	NEG	NEG	NEG	NEG	NEG	NEG	NEG
MSSA1824	NEG	NEG	NEG	NEG	NEG	NEG	NEG	POS	NEG	NEG	NEG	NEG	NEG	NEG	NEG	NEG
MSSA1827	NEG	NEG	NEG	NEG	NEG	NEG	NEG	POS	NEG	NEG	NEG	NEG	NEG	NEG	NEG	NEG
MSSA1889	NEG	NEG	NEG	NEG	NEG	NEG	NEG	POS	NEG	NEG	NEG	NEG	NEG	NEG	NEG	NEG
MSSA1895	NEG	NEG	NEG	NEG	NEG	NEG	NEG	POS	NEG	NEG	NEG	NEG	NEG	NEG	NEG	NEG
MSSA1904	NEG	NEG	NEG	NEG	NEG	NEG	NEG	POS	NEG	NEG	NEG	NEG	NEG	NEG	NEG	NEG
MSSA1905	NEG	NEG	NEG	NEG	NEG	NEG	NEG	POS	NEG	NEG	NEG	NEG	NEG	NEG	NEG	NEG
MSSA1907	NEG	NEG	NEG	NEG	NEG	NEG	NEG	POS	NEG	NEG	NEG	NEG	NEG	NEG	NEG	NEG
MSSA1919	NEG	NEG	NEG	NEG	NEG	NEG	NEG	POS	NEG	NEG	NEG	NEG	NEG	NEG	NEG	NEG
MSSA1920	NEG	NEG	NEG	NEG	NEG	NEG	NEG	POS	NEG	NEG	NEG	NEG	NEG	NEG	NEG	NEG
MSSA1929	NEG	NEG	NEG	NEG	NEG	NEG	NEG	POS	NEG	NEG	NEG	NEG	NEG	NEG	NEG	NEG
MSSA1930	NEG	NEG	NEG	NEG	NEG	NEG	NEG	POS	NEG	NEG	NEG	NEG	NEG	NEG	NEG	NEG
MSSA1934	NEG	NEG	NEG	NEG	NEG	NEG	NEG	POS	NEG	NEG	NEG	NEG	NEG	NEG	NEG	NEG
MSSA1946	NEG	NEG	NEG	NEG	NEG	NEG	NEG	POS	NEG	NEG	NEG	NEG	NEG	NEG	NEG	NEG
MSSA1971	NEG	NEG	NEG	NEG	NEG	NEG	NEG	POS	NEG	NEG	NEG	NEG	NEG	NEG	NEG	NEG
MSSA1980	NEG	NEG	NEG	NEG	NEG	NEG	NEG	POS	NEG	NEG	NEG	NEG	NEG	NEG	NEG	NEG
MSSA1982	NEG	NEG	NEG	NEG	NEG	NEG	NEG	POS	NEG	NEG	NEG	NEG	NEG	NEG	NEG	NEG
MSSA1996	NEG	NEG	NEG	NEG	NEG	NEG	NEG	POS	NEG	NEG	NEG	NEG	NEG	NEG	NEG	NEG
MSSA2002	NEG	NEG	NEG	NEG	NEG	NEG	NEG	POS	NEG	NEG	NEG	NEG	NEG	NEG	NEG	NEG
MSSA2014	NEG	NEG	NEG	NEG	NEG	NEG	NEG	POS	NEG	NEG	NEG	NEG	NEG	NEG	NEG	NEG
MSSA2053	NEG	NEG	NEG	NEG	NEG	NEG	NEG	POS	NEG	NEG	NEG	NEG	NEG	NEG	NEG	NEG
MSSA2054	NEG	NEG	NEG	NEG	NEG	NEG	NEG	POS	NEG	NEG	NEG	NEG	NEG	NEG	NEG	NEG
MSSA2058	NEG	NEG	NEG	AMB	NEG	NEG	NEG	POS	NEG	NEG	NEG	NEG	NEG	NEG	NEG	NEG
MSSA2068	NEG	NEG	NEG	NEG	NEG	NEG	NEG	POS	NEG	NEG	NEG	NEG	NEG	NEG	NEG	NEG
MSSA2070	NEG	NEG	NEG	NEG	NEG	NEG	NEG	POS	NEG	NEG	NEG	NEG	NEG	NEG	NEG	NEG
MSSA2072	NEG	NEG	NEG	NEG	NEG	NEG	NEG	POS	NEG	NEG	NEG	NEG	NEG	NEG	NEG	NEG
MSSA2073	NEG	NEG	NEG	NEG	NEG	NEG	NEG	POS	NEG	NEG	NEG	NEG	NEG	NEG	NEG	NEG
MSSA2074	NEG	NEG	NEG	NEG	NEG	NEG	NEG	POS	NEG	NEG	NEG	NEG	NEG	NEG	NEG	NEG
MSSA2107	NEG	NEG	NEG	NEG	NEG	NEG	NEG	POS	NEG	NEG	NEG	NEG	NEG	NEG	NEG	NEG
MSSA2108	NEG	NEG	NEG	NEG	NEG	NEG	NEG	POS	NEG	NEG	NEG	NEG	NEG	NEG	NEG	NEG
MSSA2129	NEG	NEG	NEG	NEG	NEG	NEG	NEG	POS	NEG	NEG	NEG	NEG	NEG	NEG	NEG	NEG
MSSA2135	NEG	NEG	NEG	NEG	NEG	NEG	NEG	POS	NEG	NEG	NEG	NEG	NEG	NEG	NEG	NEG
MSSA2137	NEG	NEG	NEG	NEG	NEG	NEG	NEG	POS	NEG	NEG	NEG	NEG	NEG	NEG	NEG	NEG
MSSA2140	NEG	NEG	NEG	NEG	NEG	NEG	NEG	POS	NEG	NEG	NEG	NEG	NEG	NEG	NEG	NEG
MSSA2182	NEG	NEG	NEG	NEG	NEG	NEG	NEG	POS	NEG	NEG	NEG	NEG	NEG	NEG	NEG	NEG
MSSA2188	NEG	NEG	NEG	NEG	NEG	NEG	NEG	POS	NEG	NEG	NEG	NEG	NEG	NEG	NEG	NEG
MSSA1899	NEG	NEG	NEG	NEG	NEG	NEG	NEG	POS	NEG	NEG	NEG	NEG	NEG	NEG	NEG	NEG
MSSA1915	NEG	NEG	NEG	NEG	NEG	NEG	NEG	POS	NEG	NEG	NEG	NEG	NEG	NEG	NEG	NEG
MSSA1949	NEG	NEG	NEG	NEG	NEG	NEG	NEG	POS	NEG	NEG	NEG	NEG	NEG	NEG	NEG	NEG
MSSA2042	NEG	NEG	NEG	NEG	NEG	NEG	NEG	POS	NEG	NEG	NEG	NEG	NEG	NEG	NEG	NEG
MSSA2077	NEG	NEG	NEG	NEG	NEG	NEG	NEG	POS	NEG	NEG	NEG	NEG	NEG	NEG	NEG	NEG
MSSA2095	NEG	NEG	NEG	NEG	NEG	NEG	NEG	POS	NEG	NEG	NEG	NEG	NEG	NEG	NEG	NEG
MSSA1997	NEG	NEG	NEG	NEG	NEG	NEG	NEG	POS	NEG	NEG	NEG	NEG	NEG	NEG	NEG	NEG
MSSA1810	NEG	NEG	NEG	NEG	NEG	NEG	NEG	POS	NEG	NEG	NEG	NEG	NEG	NEG	NEG	NEG
MSSA1832	NEG	NEG	NEG	NEG	NEG	NEG	NEG	POS	NEG	NEG	NEG	NEG	NEG	NEG	NEG	NEG
MSSA1891	NEG	NEG	NEG	NEG	NEG	NEG	NEG	POS	NEG	NEG	NEG	NEG	NEG	NEG	NEG	NEG
MSSA1892	NEG	NEG	NEG	NEG	NEG	NEG	NEG	POS	NEG	NEG	NEG	NEG	NEG	NEG	NEG	NEG
MSSA1932	NEG	NEG	NEG	NEG	NEG	NEG	NEG	POS	NEG	NEG	NEG	NEG	NEG	NEG	NEG	NEG
MSSA1942	NEG	NEG	NEG	NEG	NEG	NEG	NEG	POS	NEG	NEG	NEG	NEG	NEG	NEG	NEG	NEG
MSSA1957	NEG	NEG	NEG	NEG	NEG	NEG	NEG	POS	NEG	NEG	NEG	NEG	NEG	NEG	NEG	NEG
MSSA1748	NEG	NEG	NEG	NEG	NEG	NEG	NEG	POS	NEG	NEG	NEG	NEG	NEG	NEG	POS	NEG
MSSA1754	NEG	NEG	NEG	NEG	NEG	NEG	NEG	POS	NEG	NEG	NEG	NEG	NEG	NEG	POS	NEG
MSSA1770	NEG	NEG	NEG	NEG	NEG	NEG	NEG	POS	NEG	NEG	NEG	NEG	NEG	NEG	POS	NEG
MSSA1788	NEG	NEG	NEG	NEG	NEG	NEG	NEG	POS	NEG	NEG	NEG	NEG	NEG	NEG	POS	NEG
MSSA1804	NEG	NEG	NEG	NEG	NEG	NEG	NEG	POS	NEG	NEG	NEG	NEG	NEG	NEG	POS	NEG
MSSA1818	NEG	NEG	NEG	NEG	NEG	NEG	NEG	POS	NEG	NEG	NEG	NEG	NEG	NEG	POS	NEG
MSSA1914	NEG	NEG	NEG	NEG	NEG	NEG	NEG	POS	NEG	NEG	NEG	NEG	NEG	NEG	POS	NEG
MSSA1974	NEG	NEG	NEG	NEG	NEG	NEG	NEG	POS	NEG	NEG	NEG	NEG	NEG	NEG	POS	NEG
MSSA2023	NEG	NEG	NEG	NEG	NEG	NEG	NEG	POS	NEG	NEG	NEG	NEG	NEG	NEG	POS	NEG
MSSA2030	NEG	NEG	NEG	NEG	NEG	POS	NEG	POS	NEG	NEG	NEG	NEG	NEG	NEG	POS	NEG
MSSA2096	NEG	NEG	NEG	NEG	NEG	NEG	NEG	POS	NEG	AMB	NEG	NEG	AMB	NEG	POS	NEG
MSSA2142	NEG	NEG	NEG	NEG	NEG	NEG	NEG	POS	NEG	NEG	NEG	NEG	NEG	NEG	POS	NEG
MSSA2203	NEG	NEG	NEG	NEG	NEG	NEG	NEG	POS	NEG	NEG	NEG	NEG	NEG	NEG	POS	NEG
MSSA1901	NEG	NEG	NEG	NEG	NEG	NEG	NEG	POS	NEG	NEG	NEG	NEG	NEG	NEG	POS	NEG
MSSA2013	NEG	NEG	NEG	NEG	NEG	NEG	NEG	POS	NEG	NEG	NEG	NEG	NEG	NEG	POS	NEG
MSSA2031	NEG	NEG	NEG	NEG	NEG	NEG	NEG	POS	NEG	NEG	NEG	NEG	NEG	NEG	POS	AMB
MSSA2052	NEG	NEG	NEG	NEG	NEG	NEG	NEG	POS	NEG	NEG	NEG	NEG	NEG	NEG	POS	NEG
MSSA2057	NEG	NEG	NEG	NEG	NEG	NEG	NEG	POS	NEG	NEG	NEG	NEG	NEG	NEG	POS	NEG
MSSA2081	NEG	NEG	NEG	NEG	NEG	NEG	NEG	POS	NEG	NEG	NEG	NEG	NEG	NEG	NEG	NEG
MSSA1955	NEG	NEG	NEG	NEG	NEG	NEG	NEG	POS	NEG	NEG	NEG	NEG	NEG	NEG	NEG	NEG
MSSA1941	NEG	NEG	NEG	NEG	NEG	NEG	NEG	POS	NEG	NEG	NEG	NEG	NEG	NEG	NEG	NEG
MRSA3838	NEG	NEG	NEG	NEG	NEG	AMB	POS	POS	NEG	NEG	NEG	NEG	NEG	NEG	NEG	NEG
MRSA3839	NEG	NEG	NEG	NEG	NEG	POS	POS	POS	NEG	NEG	NEG	NEG	NEG	NEG	NEG	NEG
MSSA1801	NEG	NEG	NEG	NEG	NEG	NEG	NEG	POS	NEG	NEG	NEG	NEG	NEG	NEG	NEG	NEG
MSSA2131	NEG	NEG	NEG	NEG	NEG	NEG	NEG	POS	NEG	NEG	NEG	NEG	NEG	NEG	NEG	NEG
MSSA1792	NEG	NEG	NEG	NEG	NEG	NEG	NEG	POS	NEG	NEG	NEG	NEG	NEG	NEG	POS	NEG
MSSA1796	NEG	NEG	NEG	NEG	NEG	NEG	NEG	POS	NEG	NEG	NEG	NEG	NEG	NEG	NEG	POS
MSSA1817	NEG	NEG	NEG	NEG	NEG	NEG	NEG	POS	NEG	NEG	NEG	NEG	NEG	NEG	NEG	NEG
MSSA1911	NEG	NEG	NEG	NEG	NEG	NEG	NEG	NEG	NEG	NEG	NEG	NEG	NEG	NEG	NEG	NEG
MSSA1927	NEG	NEG	NEG	NEG	NEG	NEG	NEG	POS	NEG	NEG	NEG	NEG	NEG	NEG	POS	NEG
MSSA1928	NEG	NEG	NEG	NEG	NEG	NEG	NEG	POS	NEG	NEG	NEG	NEG	NEG	NEG	POS	NEG
MSSA1953	NEG	NEG	NEG	NEG	NEG	NEG	NEG	POS	NEG	NEG	NEG	NEG	NEG	NEG	POS	NEG
MSSA2062	NEG	NEG	NEG	NEG	NEG	NEG	NEG	POS	NEG	NEG	NEG	NEG	NEG	NEG	POS	NEG
MSSA2078	NEG	NEG	NEG	NEG	NEG	NEG	NEG	POS	NEG	NEG	NEG	NEG	NEG	NEG	NEG	NEG
MSSA2136	NEG	NEG	NEG	NEG	NEG	NEG	NEG	POS	NEG	NEG	NEG	NEG	NEG	NEG	POS	NEG

	RESISTANCE: EFFLUX SYSTEMS						RESISTANCE: GLYCOPEPTIDES		
Strain	qacA	qacC (total)	qacC (equine)	qacC (SA5)	qacC (Ssap)	qacC (ST94)	vanA	vanB	vanZ
MSSA1805	NEG	NEG	NEG	NEG	NEG	NEG	NEG	NEG	NEG
MSSA1823	NEG	NEG	NEG	NEG	NEG	NEG	NEG	NEG	NEG
MSSA1837	NEG	NEG	NEG	NEG	NEG	NEG	NEG	NEG	NEG
MSSA2044	NEG	NEG	NEG	NEG	NEG	NEG	NEG	NEG	NEG
MSSA2046	NEG	NEG	NEG	NEG	NEG	NEG	NEG	NEG	NEG
MSSA2059	NEG	POS	NEG	NEG	NEG	NEG	NEG	NEG	NEG
MSSA2076	NEG	NEG	NEG	NEG	NEG	NEG	NEG	NEG	NEG
MSSA2200	NEG	NEG	NEG	NEG	NEG	NEG	NEG	NEG	NEG
MRSA3797	NEG	NEG	NEG	NEG	NEG	NEG	NEG	NEG	NEG
MRSA3798	NEG	NEG	NEG	NEG	NEG	NEG	NEG	NEG	NEG
MRSA3799	NEG	NEG	NEG	NEG	NEG	NEG	NEG	NEG	NEG
MRSA3800	NEG	NEG	NEG	NEG	NEG	NEG	NEG	NEG	NEG
MRSA3801	NEG	NEG	NEG	NEG	NEG	NEG	NEG	NEG	NEG
MRSA3804	NEG	NEG	NEG	NEG	NEG	NEG	NEG	NEG	NEG
MRSA3806	NEG	NEG	NEG	NEG	NEG	NEG	NEG	NEG	NEG
MRSA3961	NEG	NEG	NEG	NEG	NEG	NEG	NEG	NEG	NEG
MRSA3962	NEG	NEG	NEG	NEG	NEG	NEG	NEG	NEG	NEG
MRSA3963	NEG	NEG	NEG	NEG	NEG	NEG	NEG	NEG	NEG
MRSA3966	NEG	NEG	NEG	NEG	NEG	NEG	NEG	NEG	NEG
MRSA3978	NEG	NEG	NEG	NEG	NEG	NEG	NEG	NEG	NEG
MRSA3979	NEG	NEG	NEG	NEG	NEG	NEG	NEG	NEG	NEG
MRSA3980	NEG	NEG	NEG	NEG	NEG	NEG	NEG	NEG	NEG
MRSA4179	NEG	NEG	NEG	NEG	NEG	NEG	NEG	NEG	NEG
MRSA4216	NEG	NEG	NEG	NEG	NEG	NEG	NEG	NEG	NEG
MRSA4217	NEG	NEG	NEG	NEG	NEG	NEG	NEG	NEG	NEG
MRSA4220	NEG	NEG	NEG	NEG	NEG	NEG	NEG	NEG	NEG
MRSA4221	NEG	NEG	NEG	NEG	NEG	NEG	NEG	NEG	NEG
MRSA4222	NEG	NEG	NEG	NEG	NEG	NEG	NEG	NEG	NEG
MSSA1751	NEG	NEG	NEG	NEG	NEG	NEG	NEG	NEG	NEG
MSSA1760	NEG	NEG	NEG	NEG	NEG	NEG	NEG	NEG	NEG
MSSA1802	NEG	NEG	NEG	NEG	NEG	NEG	NEG	NEG	NEG
MSSA1809	NEG	POS	NEG	NEG	NEG	NEG	NEG	NEG	NEG
MSSA1815	NEG	NEG	NEG	NEG	NEG	NEG	NEG	NEG	NEG
MSSA1829	NEG	NEG	NEG	NEG	NEG	NEG	NEG	NEG	NEG
MSSA1908	NEG	NEG	NEG	NEG	NEG	NEG	NEG	NEG	NEG
MSSA1960	NEG	NEG	NEG	NEG	NEG	NEG	NEG	NEG	NEG
MSSA1965	NEG	NEG	NEG	NEG	NEG	NEG	NEG	NEG	NEG
MSSA1977	NEG	NEG	NEG	NEG	NEG	NEG	NEG	NEG	NEG
MSSA1978	NEG	NEG	NEG	NEG	NEG	NEG	NEG	NEG	NEG
MSSA1999	NEG	NEG	NEG	NEG	NEG	NEG	NEG	NEG	NEG
MSSA2003	NEG	NEG	NEG	NEG	NEG	NEG	NEG	NEG	NEG
MSSA2006	NEG	NEG	NEG	NEG	NEG	NEG	NEG	NEG	NEG
MSSA2008	NEG	NEG	NEG	NEG	NEG	NEG	NEG	NEG	NEG
MSSA2011	NEG	NEG	NEG	NEG	NEG	NEG	NEG	NEG	NEG
MSSA2017	NEG	NEG	NEG	NEG	NEG	NEG	NEG	NEG	NEG
MSSA2022	NEG	NEG	NEG	NEG	NEG	NEG	NEG	NEG	NEG
MSSA2055	NEG	NEG	NEG	NEG	NEG	NEG	NEG	NEG	NEG
MSSA2060	NEG	NEG	NEG	NEG	NEG	NEG	NEG	NEG	NEG
MSSA2080	NEG	NEG	NEG	NEG	NEG	NEG	NEG	NEG	NEG
MSSA2082	NEG	NEG	NEG	NEG	NEG	NEG	NEG	NEG	NEG
MSSA2084	NEG	NEG	NEG	NEG	NEG	NEG	NEG	NEG	NEG
MSSA2087	NEG	NEG	NEG	NEG	NEG	NEG	NEG	NEG	NEG
MSSA2094	NEG	NEG	NEG	NEG	NEG	NEG	NEG	NEG	NEG
MSSA2097	NEG	NEG	NEG	NEG	NEG	NEG	NEG	NEG	NEG
MSSA2110	NEG	NEG	NEG	NEG	NEG	NEG	NEG	NEG	NEG
MSSA2113	NEG	NEG	NEG	NEG	NEG	NEG	NEG	NEG	NEG
MSSA2132	NEG	NEG	NEG	NEG	NEG	NEG	NEG	NEG	NEG
MSSA2134	POS	NEG	NEG	NEG	NEG	NEG	NEG	NEG	NEG
MSSA1746	NEG	NEG	NEG	NEG	NEG	NEG	NEG	NEG	NEG
MSSA1757	NEG	NEG	NEG	NEG	NEG	NEG	NEG	NEG	NEG
MSSA1772	NEG	NEG	NEG	NEG	NEG	NEG	NEG	NEG	NEG
MSSA1795	NEG	NEG	NEG	NEG	NEG	NEG	NEG	NEG	NEG
MSSA1813	NEG	NEG	NEG	NEG	NEG	NEG	NEG	NEG	NEG
MSSA1826	NEG	NEG	NEG	NEG	NEG	NEG	NEG	NEG	NEG
MSSA1830	NEG	NEG	NEG	NEG	NEG	NEG	NEG	NEG	NEG
MSSA1838	NEG	NEG	NEG	NEG	NEG	NEG	NEG	NEG	NEG
MSSA1944	NEG	NEG	NEG	NEG	NEG	NEG	NEG	NEG	NEG
MSSA1947	NEG	NEG	NEG	NEG	NEG	NEG	NEG	NEG	NEG
MSSA1964	NEG	NEG	NEG	NEG	NEG	NEG	NEG	NEG	NEG
MSSA1969	NEG	NEG	NEG	NEG	NEG	NEG	NEG	NEG	NEG
MSSA2001	NEG	NEG	NEG	NEG	NEG	NEG	NEG	NEG	NEG
MSSA2004	NEG	NEG	NEG	NEG	NEG	NEG	NEG	NEG	NEG
MSSA2024	NEG	NEG	NEG	NEG	NEG	NEG	NEG	NEG	NEG
MSSA2025	NEG	NEG	NEG	NEG	NEG	NEG	NEG	NEG	NEG
MSSA2026	NEG	NEG	NEG	NEG	NEG	NEG	NEG	NEG	NEG
MSSA2032	NEG	NEG	NEG	NEG	NEG	NEG	NEG	NEG	NEG
MSSA2043	NEG	NEG	NEG	NEG	NEG	NEG	NEG	NEG	NEG
MSSA2061	NEG	NEG	NEG	NEG	NEG	NEG	NEG	NEG	NEG
MSSA2085	NEG	NEG	NEG	NEG	NEG	NEG	NEG	NEG	NEG
MSSA2098	NEG	NEG	NEG	NEG	NEG	NEG	NEG	NEG	NEG
MSSA2100	NEG	NEG	NEG	NEG	NEG	NEG	NEG	NEG	NEG
MSSA2111	NEG	NEG	NEG	NEG	NEG	NEG	NEG	NEG	NEG
MSSA2178	NEG	NEG	NEG	AMB	NEG	NEG	NEG	NEG	NEG
MSSA2202	NEG	NEG	NEG	NEG	NEG	NEG	NEG	NEG	NEG
MSSA2205	NEG	NEG	NEG	NEG	NEG	NEG	NEG	NEG	NEG
MRSA3796	POS	NEG	NEG	NEG	NEG	NEG	NEG	NEG	NEG
MRSA3805	NEG	POS	NEG	NEG	NEG	NEG	NEG	NEG	NEG
MRSA3965	NEG	NEG	NEG	NEG	NEG	NEG	NEG	NEG	NEG
MSSA1761	NEG	NEG	NEG	NEG	NEG	NEG	NEG	NEG	NEG
MSSA1764	NEG	NEG	NEG	NEG	NEG	NEG	NEG	NEG	NEG
MSSA1765	NEG	NEG	NEG	NEG	NEG	NEG	NEG	NEG	NEG
MSSA1784	NEG	NEG	NEG	NEG	NEG	NEG	NEG	NEG	NEG
MSSA1806	NEG	NEG	NEG	NEG	NEG	NEG	NEG	NEG	NEG
MSSA1807	NEG	NEG	NEG	NEG	NEG	NEG	NEG	NEG	NEG
MSSA1888	NEG	NEG	NEG	NEG	NEG	NEG	NEG	NEG	NEG
MSSA1894	NEG	NEG	NEG	NEG	NEG	NEG	NEG	NEG	NEG
MSSA1922	NEG	NEG	NEG	NEG	NEG	NEG	NEG	NEG	NEG
MSSA1950	NEG	NEG	NEG	NEG	NEG	NEG	NEG	NEG	NEG
MSSA1954	NEG	POS	NEG	NEG	NEG	NEG	NEG	NEG	NEG
MSSA1966	NEG	NEG	NEG	NEG	NEG	NEG	NEG	NEG	NEG
MSSA1972	NEG	NEG	NEG	NEG	NEG	NEG	NEG	NEG	NEG
MSSA2010	NEG	NEG	NEG	NEG	NEG	NEG	NEG	NEG	NEG
MSSA2020	NEG	NEG	NEG	NEG	NEG	NEG	NEG	NEG	NEG
MSSA2028	NEG	NEG	NEG	NEG	NEG	NEG	NEG	NEG	NEG
MSSA2088	NEG	NEG	NEG	NEG	NEG	NEG	NEG	NEG	NEG
MSSA2187	NEG	NEG	NEG	NEG	NEG	NEG	NEG	NEG	NEG
MSSA2204	NEG	NEG	NEG	NEG	NEG	NEG	NEG	NEG	NEG
MSSA2210	NEG	NEG	NEG	NEG	NEG	NEG	NEG	NEG	NEG
MSSA2223	NEG	NEG	NEG	NEG	NEG	NEG	NEG	NEG	NEG
MSSA1763	NEG	NEG	NEG	NEG	NEG	NEG	NEG	NEG	NEG
MSSA1777	NEG	NEG	NEG	NEG	NEG	NEG	NEG	NEG	NEG
MSSA2009	NEG	NEG	NEG	NEG	NEG	NEG	NEG	NEG	NEG
MSSA2047	NEG	NEG	NEG	NEG	NEG	NEG	NEG	NEG	NEG
MSSA2079	NEG	NEG	NEG	NEG	NEG	NEG	NEG	NEG	NEG
MSSA2101	NEG	NEG	NEG	NEG	NEG	NEG	NEG	NEG	NEG
MSSA1821	NEG	NEG	NEG	NEG	NEG	NEG	NEG	NEG	NEG
MSSA1898	NEG	NEG	NEG	NEG	NEG	NEG	NEG	NEG	NEG
MSSA1973	NEG	NEG	NEG	NEG	NEG	NEG	NEG	NEG	NEG
MSSA2071	NEG	NEG	NEG	NEG	NEG	NEG	NEG	NEG	NEG
MSSA2083	NEG	NEG	NEG	NEG	NEG	NEG	NEG	NEG	NEG
MSSA2106	NEG	NEG	NEG	NEG	NEG	NEG	NEG	NEG	NEG
MSSA2112	NEG	NEG	NEG	NEG	NEG	NEG	NEG	NEG	NEG
MSSA2115	NEG	NEG	NEG	NEG	NEG	NEG	NEG	NEG	NEG
MSSA2195	NEG	NEG	NEG	NEG	NEG	NEG	NEG	NEG	NEG
MSSA2207	NEG	NEG	NEG	NEG	NEG	NEG	NEG	NEG	NEG
MSSA1758	NEG	NEG	NEG	NEG	NEG	NEG	NEG	NEG	NEG
MSSA1762	NEG	NEG	NEG	NEG	NEG	NEG	NEG	NEG	NEG
MSSA1769	NEG	NEG	NEG	NEG	NEG	NEG	NEG	NEG	NEG
MSSA1771	NEG	NEG	NEG	NEG	NEG	NEG	NEG	NEG	NEG
MSSA1789	NEG	NEG	NEG	NEG	NEG	NEG	NEG	NEG	NEG
MSSA1800	NEG	NEG	NEG	NEG	NEG	NEG	NEG	NEG	NEG
MSSA1833	NEG	NEG	NEG	NEG	NEG	NEG	NEG	NEG	NEG
MSSA1890	NEG	NEG	NEG	NEG	NEG	NEG	NEG	NEG	NEG
MSSA1903	NEG	NEG	NEG	NEG	NEG	NEG	NEG	NEG	NEG
MSSA1940	NEG	NEG	NEG	NEG	NEG	NEG	NEG	NEG	NEG
MSSA1962	NEG	NEG	NEG	NEG	NEG	NEG	NEG	NEG	NEG
MSSA1963	NEG	NEG	NEG	NEG	NEG	NEG	NEG	NEG	NEG
MSSA2016	NEG	NEG	NEG	NEG	NEG	NEG	NEG	NEG	NEG
MSSA2018	NEG	NEG	NEG	NEG	NEG	NEG	NEG	NEG	NEG
MSSA2019	NEG	NEG	NEG	NEG	NEG	NEG	NEG	NEG	NEG
MSSA2029	NEG	NEG	NEG	NEG	NEG	NEG	NEG	NEG	NEG
MSSA2045	NEG	NEG	NEG	NEG	NEG	NEG	NEG	NEG	NEG
MSSA2048	NEG	NEG	NEG	NEG	NEG	NEG	NEG	NEG	NEG
MSSA2049	NEG	NEG	NEG	NEG	NEG	NEG	NEG	NEG	NEG
MSSA2050	NEG	NEG	NEG	NEG	NEG	NEG	NEG	NEG	NEG
MSSA2099	NEG	NEG	NEG	NEG	NEG	NEG	NEG	NEG	NEG
MSSA2109	NEG	NEG	NEG	NEG	NEG	NEG	NEG	NEG	NEG
MSSA2130	NEG	NEG	NEG	NEG	NEG	NEG	NEG	NEG	NEG
MSSA2133	NEG	POS	NEG	NEG	NEG	NEG	NEG	NEG	NEG
MSSA2139	NEG	NEG	NEG	NEG	NEG	POS	NEG	NEG	NEG
MSSA2141	NEG	NEG	NEG	NEG	NEG	NEG	NEG	NEG	NEG
MSSA2208	NEG	NEG	NEG	NEG	NEG	NEG	NEG	NEG	NEG
MSSA2209	NEG	NEG	AMB	NEG	NEG	POS	NEG	NEG	NEG
MSSA1893	NEG	NEG	NEG	NEG	NEG	NEG	NEG	NEG	NEG
MSSA2105	NEG	NEG	NEG	NEG	NEG	NEG	NEG	NEG	NEG
MRSA3836	NEG	NEG	NEG	NEG	NEG	NEG	NEG	NEG	NEG
MRSA3840	NEG	NEG	NEG	NEG	NEG	NEG	NEG	NEG	NEG
MRSA4188	NEG	NEG	NEG	NEG	NEG	NEG	NEG	NEG	NEG
MSSA1814	NEG	NEG	NEG	NEG	NEG	NEG	NEG	NEG	NEG
MSSA1816	NEG	NEG	NEG	NEG	NEG	NEG	NEG	NEG	NEG
MSSA1825	NEG	NEG	NEG	NEG	NEG	NEG	NEG	NEG	NEG
MSSA1834	NEG	NEG	NEG	NEG	NEG	NEG	NEG	NEG	NEG
MSSA1938	NEG	NEG	NEG	NEG	NEG	NEG	NEG	NEG	NEG
MSSA1948	NEG	NEG	NEG	NEG	NEG	NEG	NEG	NEG	NEG
MSSA1951	NEG	NEG	NEG	NEG	NEG	NEG	NEG	NEG	NEG
MSSA2005	NEG	NEG	NEG	NEG	NEG	NEG	NEG	NEG	NEG
MSSA2051	NEG	NEG	NEG	NEG	NEG	NEG	NEG	NEG	NEG
MSSA2056	NEG	NEG	NEG	NEG	NEG	NEG	NEG	NEG	NEG
MSSA2066	NEG	NEG	NEG	NEG	NEG	NEG	NEG	NEG	NEG
MSSA2067	NEG	NEG	NEG	NEG	NEG	NEG	NEG	NEG	NEG
MSSA2086	NEG	NEG	NEG	NEG	NEG	NEG	NEG	NEG	NEG
MSSA2089	NEG	NEG	NEG	NEG	NEG	NEG	NEG	NEG	NEG
MSSA2103	NEG	NEG	NEG	NEG	NEG	NEG	NEG	NEG	NEG
MSSA2173	NEG	NEG	NEG	NEG	NEG	NEG	NEG	NEG	NEG
MSSA2183	NEG	NEG	NEG	NEG	NEG	NEG	NEG	NEG	NEG
MSSA2196	NEG	NEG	NEG	NEG	NEG	NEG	NEG	NEG	NEG
MSSA2219	NEG	NEG	NEG	NEG	NEG	NEG	NEG	NEG	NEG
MSSA1753	NEG	NEG	NEG	NEG	NEG	NEG	NEG	NEG	NEG
MSSA1773	NEG	POS	NEG	NEG	NEG	NEG	NEG	NEG	NEG
MSSA1811	NEG	NEG	NEG	NEG	NEG	NEG	NEG	NEG	NEG
MSSA1897	NEG	NEG	NEG	NEG	NEG	NEG	NEG	NEG	NEG
MSSA1931	NEG	NEG	NEG	NEG	NEG	NEG	NEG	NEG	NEG
MSSA2114	NEG	NEG	NEG	NEG	NEG	NEG	NEG	NEG	NEG
MSSA1745	NEG	NEG	NEG	NEG	NEG	NEG	NEG	NEG	NEG
MSSA1747	NEG	NEG	NEG	NEG	NEG	NEG	NEG	NEG	NEG
MSSA1749	NEG	NEG	NEG	NEG	NEG	NEG	NEG	NEG	NEG
MSSA1768	NEG	NEG	NEG	NEG	NEG	NEG	NEG	NEG	NEG
MSSA1774	NEG	NEG	NEG	NEG	NEG	NEG	NEG	NEG	NEG
MSSA1775	NEG	NEG	NEG	NEG	NEG	NEG	NEG	NEG	NEG
MSSA1776	NEG	NEG	NEG	NEG	NEG	NEG	NEG	NEG	NEG
MSSA1779	NEG	NEG	NEG	NEG	NEG	NEG	NEG	NEG	NEG
MSSA1780	NEG	NEG	NEG	NEG	NEG	NEG	NEG	NEG	NEG

	RESISTANCE: EFFLUX SYSTEMS						RESISTANCE: GLYCOPEPTIDES		
**Strain**	**qacA**	**qacC (total)**	**qacC (equine)**	**qacC (SA5)**	**qacC (Ssap)**	**qacC (ST94)**	**vanA**	**vanB**	**vanZ**
MSSA1781	NEG	NEG	NEG	NEG	NEG	NEG	NEG	NEG	NEG
MSSA1786	NEG	NEG	NEG	NEG	NEG	NEG	NEG	NEG	NEG
MSSA1793	NEG	NEG	NEG	NEG	NEG	NEG	NEG	NEG	NEG
MSSA1803	NEG	NEG	NEG	NEG	NEG	NEG	NEG	NEG	NEG
MSSA1836	NEG	NEG	NEG	NEG	NEG	NEG	NEG	NEG	NEG
MSSA1839	NEG	NEG	NEG	NEG	NEG	NEG	NEG	NEG	NEG
MSSA1900	NEG	NEG	NEG	NEG	NEG	NEG	NEG	NEG	NEG
MSSA1910	NEG	NEG	NEG	NEG	NEG	NEG	NEG	NEG	NEG
MSSA1918	NEG	NEG	NEG	NEG	NEG	NEG	NEG	NEG	NEG
MSSA1921	NEG	NEG	NEG	NEG	NEG	NEG	NEG	NEG	NEG
MSSA1923	NEG	NEG	NEG	NEG	NEG	NEG	NEG	NEG	NEG
MSSA1981	NEG	NEG	NEG	NEG	NEG	NEG	NEG	NEG	NEG
MSSA1998	NEG	NEG	NEG	NEG	NEG	NEG	NEG	NEG	NEG
MSSA2000	NEG	NEG	NEG	NEG	NEG	NEG	NEG	NEG	NEG
MSSA2007	NEG	NEG	NEG	NEG	NEG	NEG	NEG	NEG	NEG
MSSA2012	NEG	NEG	NEG	NEG	NEG	NEG	NEG	NEG	NEG
MSSA2015	NEG	NEG	NEG	NEG	NEG	NEG	NEG	NEG	NEG
MSSA2021	NEG	NEG	NEG	NEG	NEG	NEG	NEG	NEG	NEG
MSSA2027	NEG	NEG	NEG	NEG	NEG	NEG	NEG	NEG	NEG
MSSA2063	NEG	NEG	NEG	NEG	NEG	NEG	NEG	NEG	NEG
MSSA2064	NEG	NEG	NEG	NEG	NEG	NEG	NEG	NEG	NEG
MSSA2065	NEG	NEG	NEG	NEG	NEG	NEG	NEG	NEG	NEG
MSSA2075	NEG	NEG	NEG	NEG	NEG	NEG	NEG	NEG	NEG
MSSA2093	NEG	NEG	NEG	NEG	NEG	NEG	NEG	NEG	NEG
MSSA2102	NEG	NEG	NEG	NEG	NEG	NEG	NEG	NEG	NEG
MSSA2138	NEG	NEG	NEG	NEG	NEG	NEG	NEG	NEG	NEG
MSSA2168	NEG	NEG	NEG	NEG	NEG	NEG	NEG	NEG	NEG
MSSA2181	NEG	NEG	NEG	NEG	NEG	NEG	NEG	NEG	NEG
MSSA2189	NEG	NEG	NEG	NEG	NEG	NEG	NEG	NEG	NEG
MSSA2214	NEG	NEG	NEG	NEG	NEG	NEG	NEG	NEG	NEG
MRSA3803	NEG	POS	NEG	NEG	NEG	NEG	NEG	NEG	NEG
MSSA1750	NEG	NEG	NEG	NEG	NEG	NEG	NEG	NEG	NEG
MSSA1756	NEG	NEG	NEG	NEG	NEG	NEG	NEG	NEG	NEG
MSSA1759	NEG	NEG	NEG	NEG	NEG	NEG	NEG	NEG	NEG
MSSA1767	NEG	NEG	NEG	NEG	NEG	NEG	NEG	NEG	NEG
MSSA1787	NEG	NEG	NEG	NEG	NEG	NEG	NEG	NEG	NEG
MSSA1790	NEG	NEG	NEG	NEG	NEG	NEG	NEG	NEG	NEG
MSSA1794	NEG	NEG	NEG	NEG	NEG	NEG	NEG	NEG	NEG
MSSA1808	NEG	NEG	NEG	NEG	NEG	NEG	NEG	NEG	NEG
MSSA1812	NEG	NEG	NEG	NEG	NEG	NEG	NEG	NEG	NEG
MSSA1819	NEG	NEG	NEG	NEG	NEG	NEG	NEG	NEG	NEG
MSSA1824	NEG	NEG	NEG	NEG	NEG	NEG	NEG	NEG	NEG
MSSA1827	NEG	NEG	NEG	NEG	NEG	NEG	NEG	NEG	NEG
MSSA1889	NEG	NEG	NEG	NEG	NEG	NEG	NEG	NEG	NEG
MSSA1895	NEG	NEG	NEG	NEG	NEG	NEG	NEG	NEG	NEG
MSSA1904	NEG	NEG	NEG	NEG	NEG	NEG	NEG	NEG	NEG
MSSA1905	NEG	NEG	NEG	NEG	NEG	NEG	NEG	NEG	NEG
MSSA1907	NEG	NEG	NEG	NEG	NEG	NEG	NEG	NEG	NEG
MSSA1919	NEG	NEG	NEG	NEG	NEG	NEG	NEG	NEG	NEG
MSSA1920	NEG	NEG	NEG	NEG	NEG	NEG	NEG	NEG	NEG
MSSA1929	NEG	NEG	NEG	NEG	NEG	NEG	NEG	NEG	NEG
MSSA1930	NEG	NEG	NEG	NEG	NEG	NEG	NEG	NEG	NEG
MSSA1934	NEG	NEG	NEG	NEG	NEG	NEG	NEG	NEG	NEG
MSSA1946	NEG	NEG	NEG	NEG	NEG	NEG	NEG	NEG	NEG
MSSA1971	NEG	NEG	NEG	NEG	NEG	NEG	NEG	NEG	NEG
MSSA1980	NEG	NEG	NEG	NEG	NEG	NEG	NEG	NEG	NEG
MSSA1982	NEG	NEG	NEG	NEG	NEG	NEG	NEG	NEG	NEG
MSSA1996	NEG	NEG	NEG	NEG	NEG	NEG	NEG	NEG	NEG
MSSA2002	NEG	NEG	NEG	NEG	NEG	NEG	NEG	NEG	NEG
MSSA2014	NEG	NEG	NEG	NEG	NEG	NEG	NEG	NEG	NEG
MSSA2053	NEG	NEG	NEG	NEG	NEG	NEG	NEG	NEG	NEG
MSSA2054	NEG	NEG	NEG	NEG	NEG	NEG	NEG	NEG	NEG
MSSA2058	NEG	NEG	NEG	NEG	NEG	NEG	NEG	NEG	NEG
MSSA2068	NEG	NEG	NEG	NEG	NEG	NEG	NEG	NEG	NEG
MSSA2070	NEG	NEG	NEG	NEG	NEG	NEG	NEG	NEG	NEG
MSSA2072	NEG	NEG	NEG	NEG	NEG	NEG	NEG	NEG	NEG
MSSA2073	NEG	NEG	NEG	NEG	NEG	NEG	NEG	NEG	NEG
MSSA2074	NEG	NEG	NEG	NEG	NEG	NEG	NEG	NEG	NEG
MSSA2107	NEG	NEG	NEG	NEG	NEG	NEG	NEG	NEG	NEG
MSSA2108	POS	NEG	NEG	NEG	NEG	NEG	NEG	NEG	NEG
MSSA2129	NEG	NEG	NEG	NEG	NEG	NEG	NEG	NEG	NEG
MSSA2135	NEG	NEG	NEG	NEG	NEG	NEG	NEG	NEG	NEG
MSSA2137	NEG	NEG	NEG	NEG	NEG	NEG	NEG	NEG	NEG
MSSA2140	NEG	NEG	NEG	NEG	NEG	NEG	NEG	NEG	NEG
MSSA2182	NEG	NEG	NEG	NEG	NEG	NEG	NEG	NEG	NEG
MSSA2188	NEG	NEG	NEG	NEG	NEG	NEG	NEG	NEG	NEG
MSSA1899	NEG	NEG	NEG	NEG	NEG	NEG	NEG	NEG	NEG
MSSA1915	NEG	NEG	NEG	NEG	NEG	NEG	NEG	NEG	NEG
MSSA1949	NEG	NEG	NEG	NEG	NEG	NEG	NEG	NEG	NEG
MSSA2042	NEG	NEG	NEG	NEG	NEG	NEG	NEG	NEG	NEG
MSSA2077	NEG	NEG	NEG	NEG	NEG	NEG	NEG	NEG	NEG
MSSA2095	NEG	NEG	NEG	NEG	NEG	NEG	NEG	NEG	NEG
MSSA1997	NEG	POS	NEG	NEG	NEG	NEG	NEG	NEG	NEG
MSSA1810	NEG	NEG	NEG	NEG	NEG	NEG	NEG	NEG	NEG
MSSA1832	NEG	NEG	NEG	NEG	NEG	NEG	NEG	NEG	NEG
MSSA1891	NEG	NEG	NEG	NEG	NEG	NEG	NEG	NEG	NEG
MSSA1892	NEG	NEG	NEG	NEG	NEG	NEG	NEG	NEG	NEG
MSSA1932	NEG	NEG	NEG	NEG	NEG	NEG	NEG	NEG	NEG
MSSA1942	NEG	NEG	NEG	NEG	NEG	NEG	NEG	NEG	NEG
MSSA1957	NEG	NEG	NEG	NEG	NEG	NEG	NEG	NEG	NEG
MSSA1748	NEG	NEG	NEG	NEG	NEG	NEG	NEG	NEG	NEG
MSSA1754	NEG	NEG	NEG	NEG	NEG	NEG	NEG	NEG	NEG
MSSA1770	NEG	NEG	NEG	NEG	NEG	NEG	NEG	NEG	NEG
MSSA1788	NEG	NEG	NEG	NEG	NEG	NEG	NEG	NEG	NEG
MSSA1804	NEG	NEG	NEG	NEG	NEG	NEG	NEG	NEG	NEG
MSSA1818	NEG	NEG	NEG	NEG	NEG	NEG	NEG	NEG	NEG
MSSA1914	NEG	NEG	NEG	NEG	NEG	NEG	NEG	NEG	NEG
MSSA1974	NEG	NEG	NEG	NEG	NEG	NEG	NEG	NEG	NEG
MSSA2023	NEG	NEG	NEG	NEG	NEG	NEG	NEG	NEG	NEG
MSSA2030	NEG	NEG	NEG	NEG	NEG	NEG	NEG	NEG	NEG
MSSA2096	NEG	NEG	NEG	NEG	NEG	NEG	NEG	NEG	NEG
MSSA2142	NEG	NEG	NEG	NEG	NEG	NEG	NEG	NEG	NEG
MSSA2203	NEG	NEG	NEG	NEG	NEG	NEG	NEG	NEG	NEG
MSSA1901	NEG	NEG	NEG	NEG	NEG	NEG	NEG	NEG	NEG
MSSA2013	NEG	NEG	NEG	NEG	NEG	NEG	NEG	NEG	NEG
MSSA2031	NEG	NEG	NEG	NEG	NEG	NEG	NEG	NEG	NEG
MSSA2052	NEG	NEG	NEG	NEG	NEG	NEG	NEG	NEG	NEG
MSSA2057	NEG	NEG	NEG	NEG	NEG	NEG	NEG	NEG	NEG
MSSA2081	NEG	NEG	NEG	NEG	NEG	NEG	NEG	NEG	NEG
MSSA1955	NEG	NEG	NEG	NEG	NEG	NEG	NEG	NEG	NEG
MSSA1941	NEG	NEG	NEG	NEG	NEG	NEG	NEG	NEG	NEG
MRSA3838	NEG	NEG	NEG	NEG	NEG	NEG	NEG	NEG	NEG
MRSA3839	NEG	NEG	NEG	NEG	NEG	NEG	NEG	NEG	NEG
MSSA1801	NEG	NEG	NEG	NEG	NEG	NEG	NEG	NEG	NEG
MSSA2131	NEG	NEG	NEG	NEG	NEG	NEG	NEG	NEG	NEG
MSSA1792	NEG	NEG	NEG	NEG	NEG	NEG	NEG	NEG	NEG
MSSA1796	NEG	NEG	NEG	NEG	NEG	NEG	NEG	NEG	NEG
MSSA1817	NEG	NEG	NEG	NEG	NEG	NEG	NEG	NEG	NEG
MSSA1911	NEG	NEG	NEG	NEG	NEG	NEG	NEG	NEG	NEG
MSSA1927	NEG	NEG	NEG	NEG	NEG	NEG	NEG	NEG	NEG
MSSA1928	NEG	NEG	NEG	NEG	NEG	NEG	NEG	NEG	NEG
MSSA1953	NEG	NEG	NEG	NEG	NEG	NEG	NEG	NEG	NEG
MSSA2062	NEG	NEG	NEG	NEG	NEG	NEG	NEG	NEG	NEG
MSSA2078	NEG	NEG	NEG	NEG	NEG	NEG	NEG	NEG	NEG
MSSA2136	NEG	NEG	NEG	NEG	NEG	NEG	NEG	NEG	NEG

	VIRULENCE: TOX.SCHOCK		VIRULENCE: ENTEROTOXINS																
Strain	tst1 (cons)	tst1 (other than RF122)	sea = entA	sea = entA (320E)	sea = entA (N315) = entP	seb = entB	sec = entC	sed = entD	see = entE	seg = entG	seh = entH	sei = entI	sej = entJ	sek = entK (probe 1)	sek = entK (probe 2)	sel = entL	sem = entM	sen = entN	sen = entN other than RF122
MSSA1805	NEG	NEG	NEG	NEG	NEG	NEG	NEG	NEG	NEG	NEG	POS	NEG	NEG	NEG	NEG	NEG	NEG	NEG	NEG
MSSA1823	NEG	NEG	NEG	NEG	NEG	NEG	NEG	NEG	NEG	NEG	POS	NEG	NEG	NEG	NEG	NEG	NEG	NEG	NEG
MSSA1837	NEG	NEG	POS	NEG	NEG	NEG	NEG	NEG	NEG	NEG	POS	NEG	NEG	POS	POS	NEG	NEG	NEG	NEG
MSSA2044	NEG	NEG	POS	NEG	NEG	NEG	NEG	NEG	NEG	NEG	POS	NEG	NEG	POS	POS	NEG	NEG	NEG	NEG
MSSA2046	NEG	NEG	POS	NEG	NEG	POS	NEG	NEG	NEG	NEG	POS	NEG	NEG	NEG	NEG	NEG	NEG	NEG	NEG
MSSA2059	POS	POS	POS	NEG	NEG	NEG	NEG	NEG	NEG	NEG	POS	NEG	NEG	POS	POS	NEG	NEG	NEG	NEG
MSSA2076	NEG	NEG	NEG	NEG	NEG	NEG	NEG	NEG	NEG	NEG	POS	NEG	NEG	NEG	NEG	NEG	NEG	NEG	NEG
MSSA2200	NEG	NEG	POS	NEG	NEG	NEG	NEG	NEG	NEG	NEG	POS	NEG	NEG	POS	POS	NEG	NEG	NEG	NEG
MRSA3797	NEG	NEG	NEG	NEG	POS	NEG	NEG	POS	NEG	POS	NEG	POS	POS	NEG	NEG	NEG	POS	POS	POS
MRSA3798	NEG	NEG	NEG	NEG	POS	NEG	NEG	POS	NEG	POS	NEG	POS	POS	NEG	NEG	NEG	POS	POS	POS
MRSA3799	NEG	NEG	NEG	NEG	POS	NEG	NEG	POS	NEG	POS	NEG	POS	POS	NEG	NEG	NEG	POS	POS	POS
MRSA3800	NEG	NEG	NEG	NEG	NEG	NEG	NEG	POS	NEG	POS	NEG	POS	POS	NEG	NEG	NEG	POS	POS	POS
MRSA3801	NEG	NEG	NEG	NEG	POS	NEG	NEG	POS	NEG	POS	NEG	POS	POS	NEG	NEG	NEG	POS	POS	POS
MRSA3804	NEG	NEG	NEG	NEG	POS	NEG	NEG	POS	NEG	POS	NEG	POS	POS	NEG	NEG	NEG	POS	POS	POS
MRSA3806	NEG	NEG	NEG	NEG	POS	NEG	NEG	POS	NEG	POS	NEG	POS	POS	NEG	NEG	NEG	POS	POS	POS
MRSA3961	NEG	NEG	NEG	NEG	POS	NEG	NEG	POS	NEG	POS	NEG	POS	POS	NEG	NEG	NEG	POS	POS	POS
MRSA3962	NEG	NEG	NEG	NEG	POS	NEG	NEG	NEG	NEG	POS	NEG	POS	NEG	NEG	NEG	NEG	POS	POS	POS
MRSA3963	NEG	NEG	NEG	NEG	POS	NEG	NEG	NEG	NEG	POS	NEG	POS	NEG	NEG	NEG	NEG	POS	POS	POS
MRSA3966	NEG	NEG	NEG	NEG	POS	NEG	NEG	NEG	NEG	POS	NEG	POS	NEG	NEG	NEG	NEG	POS	POS	POS
MRSA3978	NEG	NEG	NEG	NEG	POS	NEG	NEG	POS	NEG	POS	NEG	POS	POS	NEG	NEG	NEG	POS	POS	POS
MRSA3979	NEG	NEG	NEG	NEG	POS	NEG	NEG	POS	NEG	POS	NEG	POS	POS	NEG	NEG	NEG	POS	POS	POS
MRSA3980	NEG	NEG	NEG	NEG	POS	NEG	NEG	POS	NEG	POS	NEG	POS	POS	NEG	NEG	NEG	POS	POS	POS
MRSA4179	NEG	NEG	NEG	NEG	POS	NEG	NEG	NEG	NEG	POS	NEG	POS	NEG	NEG	NEG	NEG	POS	POS	POS
MRSA4216	NEG	NEG	NEG	NEG	NEG	NEG	NEG	POS	NEG	POS	NEG	POS	POS	NEG	NEG	NEG	POS	POS	POS
MRSA4217	NEG	NEG	NEG	NEG	POS	NEG	NEG	POS	NEG	POS	NEG	POS	POS	NEG	NEG	NEG	POS	POS	POS
MRSA4220	NEG	NEG	NEG	NEG	POS	NEG	NEG	NEG	NEG	POS	NEG	POS	NEG	NEG	NEG	NEG	POS	POS	POS
MRSA4221	NEG	NEG	NEG	NEG	POS	NEG	NEG	POS	NEG	POS	NEG	POS	POS	NEG	NEG	NEG	POS	POS	POS
MRSA4222	NEG	NEG	NEG	NEG	POS	NEG	NEG	POS	NEG	POS	NEG	POS	POS	NEG	NEG	AMB	POS	POS	POS
MSSA1751	NEG	NEG	NEG	NEG	POS	NEG	NEG	POS	NEG	POS	NEG	POS	POS	NEG	NEG	NEG	POS	POS	POS
MSSA1760	NEG	NEG	NEG	NEG	POS	NEG	NEG	NEG	NEG	POS	NEG	POS	NEG	NEG	NEG	NEG	POS	POS	POS
MSSA1802	NEG	NEG	NEG	NEG	POS	NEG	NEG	POS	NEG	POS	NEG	POS	POS	NEG	NEG	NEG	POS	POS	POS
MSSA1809	NEG	NEG	NEG	NEG	POS	NEG	NEG	NEG	NEG	POS	NEG	POS	NEG	NEG	NEG	NEG	POS	POS	POS
MSSA1815	POS	POS	NEG	NEG	POS	NEG	NEG	NEG	NEG	POS	NEG	POS	NEG	NEG	NEG	NEG	POS	POS	POS
MSSA1829	NEG	NEG	NEG	NEG	NEG	NEG	NEG	NEG	NEG	POS	NEG	POS	NEG	NEG	NEG	NEG	POS	POS	POS
MSSA1908	NEG	NEG	NEG	NEG	POS	NEG	NEG	NEG	NEG	POS	NEG	POS	NEG	NEG	NEG	NEG	POS	POS	POS
MSSA1960	NEG	NEG	NEG	NEG	POS	NEG	NEG	NEG	NEG	POS	NEG	POS	NEG	NEG	NEG	NEG	POS	POS	POS
MSSA1965	NEG	NEG	NEG	NEG	POS	NEG	NEG	NEG	NEG	POS	NEG	POS	NEG	NEG	NEG	NEG	POS	POS	POS
MSSA1977	NEG	NEG	NEG	NEG	NEG	NEG	NEG	NEG	NEG	POS	NEG	POS	NEG	NEG	NEG	NEG	POS	POS	POS
MSSA1978	NEG	NEG	NEG	NEG	NEG	POS	NEG	NEG	NEG	POS	NEG	POS	NEG	NEG	NEG	NEG	POS	POS	POS
MSSA1999	NEG	NEG	NEG	NEG	POS	NEG	NEG	POS	NEG	POS	NEG	POS	POS	NEG	NEG	NEG	POS	POS	POS
MSSA2003	NEG	NEG	NEG	NEG	NEG	NEG	NEG	NEG	NEG	POS	NEG	POS	NEG	NEG	NEG	NEG	POS	POS	POS
MSSA2006	NEG	NEG	NEG	NEG	POS	NEG	NEG	NEG	NEG	POS	NEG	POS	NEG	NEG	NEG	NEG	POS	POS	POS
MSSA2008	NEG	NEG	POS	NEG	NEG	NEG	NEG	NEG	NEG	POS	NEG	POS	NEG	NEG	NEG	NEG	POS	POS	POS
MSSA2011	NEG	NEG	NEG	NEG	POS	NEG	NEG	POS	NEG	POS	NEG	POS	POS	NEG	NEG	NEG	POS	POS	POS
MSSA2017	NEG	NEG	NEG	NEG	NEG	NEG	NEG	NEG	NEG	POS	NEG	POS	NEG	NEG	NEG	NEG	POS	POS	POS
MSSA2022	NEG	NEG	NEG	NEG	POS	NEG	NEG	POS	NEG	POS	NEG	POS	POS	NEG	NEG	NEG	POS	POS	POS
MSSA2055	NEG	NEG	NEG	NEG	POS	NEG	NEG	NEG	NEG	POS	NEG	POS	NEG	NEG	NEG	NEG	POS	POS	POS
MSSA2060	NEG	NEG	NEG	NEG	POS	NEG	NEG	NEG	NEG	POS	NEG	POS	NEG	NEG	NEG	NEG	POS	POS	POS
MSSA2080	NEG	NEG	NEG	NEG	POS	NEG	NEG	NEG	NEG	POS	NEG	POS	NEG	NEG	NEG	NEG	POS	POS	POS
MSSA2082	NEG	NEG	NEG	NEG	NEG	NEG	NEG	NEG	NEG	POS	NEG	POS	NEG	NEG	NEG	NEG	POS	POS	POS
MSSA2084	NEG	NEG	NEG	NEG	NEG	NEG	NEG	NEG	NEG	POS	NEG	POS	NEG	NEG	NEG	NEG	POS	POS	POS
MSSA2087	NEG	NEG	NEG	NEG	POS	NEG	NEG	NEG	NEG	POS	NEG	POS	NEG	NEG	NEG	NEG	POS	POS	POS
MSSA2094	NEG	NEG	NEG	NEG	POS	NEG	NEG	NEG	NEG	POS	NEG	POS	NEG	NEG	NEG	NEG	POS	POS	POS
MSSA2097	NEG	NEG	NEG	NEG	NEG	NEG	NEG	NEG	NEG	POS	NEG	POS	NEG	NEG	NEG	NEG	POS	POS	POS
MSSA2110	NEG	NEG	NEG	NEG	POS	NEG	NEG	NEG	NEG	POS	NEG	POS	NEG	NEG	NEG	NEG	POS	POS	POS
MSSA2113	NEG	NEG	NEG	NEG	NEG	NEG	NEG	NEG	NEG	POS	NEG	POS	NEG	NEG	NEG	NEG	POS	POS	POS
MSSA2132	NEG	NEG	NEG	NEG	POS	NEG	NEG	NEG	NEG	POS	NEG	POS	NEG	NEG	NEG	NEG	POS	POS	POS
MSSA2134	NEG	NEG	NEG	NEG	POS	NEG	NEG	NEG	NEG	POS	NEG	POS	NEG	NEG	NEG	NEG	POS	POS	POS
MSSA1746	NEG	NEG	NEG	NEG	POS	NEG	NEG	NEG	NEG	NEG	NEG	NEG	NEG	NEG	NEG	NEG	NEG	NEG	NEG
MSSA1757	NEG	NEG	NEG	NEG	POS	NEG	NEG	NEG	NEG	NEG	NEG	NEG	NEG	NEG	NEG	NEG	NEG	NEG	NEG
MSSA1772	NEG	NEG	NEG	NEG	POS	NEG	NEG	NEG	NEG	NEG	NEG	NEG	NEG	NEG	NEG	NEG	NEG	NEG	NEG
MSSA1795	NEG	NEG	NEG	NEG	POS	NEG	NEG	NEG	NEG	NEG	NEG	NEG	AMB	NEG	NEG	NEG	NEG	NEG	NEG
MSSA1813	NEG	NEG	NEG	NEG	POS	NEG	NEG	NEG	NEG	NEG	NEG	NEG	NEG	NEG	NEG	NEG	NEG	NEG	NEG
MSSA1826	NEG	NEG	NEG	NEG	POS	NEG	NEG	NEG	NEG	NEG	NEG	NEG	NEG	NEG	NEG	NEG	NEG	NEG	NEG
MSSA1830	NEG	NEG	NEG	NEG	POS	NEG	NEG	NEG	NEG	NEG	NEG	NEG	NEG	NEG	NEG	NEG	NEG	NEG	NEG
MSSA1838	NEG	NEG	NEG	NEG	POS	NEG	NEG	NEG	NEG	NEG	NEG	NEG	NEG	NEG	NEG	NEG	NEG	NEG	NEG
MSSA1944	NEG	NEG	NEG	NEG	POS	NEG	NEG	NEG	NEG	NEG	NEG	NEG	NEG	NEG	NEG	NEG	NEG	NEG	NEG
MSSA1947	NEG	NEG	NEG	NEG	POS	NEG	NEG	NEG	NEG	NEG	NEG	NEG	NEG	NEG	NEG	NEG	NEG	NEG	NEG
MSSA1964	NEG	NEG	NEG	NEG	POS	NEG	NEG	NEG	NEG	NEG	NEG	NEG	NEG	NEG	NEG	NEG	NEG	NEG	NEG
MSSA1969	NEG	NEG	NEG	NEG	POS	NEG	NEG	NEG	NEG	NEG	NEG	NEG	NEG	NEG	NEG	NEG	NEG	NEG	NEG
MSSA2001	NEG	NEG	NEG	NEG	POS	NEG	NEG	NEG	NEG	NEG	NEG	NEG	NEG	NEG	NEG	NEG	NEG	NEG	NEG
MSSA2004	NEG	NEG	NEG	NEG	POS	NEG	NEG	NEG	NEG	NEG	NEG	NEG	NEG	NEG	NEG	NEG	NEG	NEG	NEG
MSSA2024	NEG	NEG	NEG	NEG	POS	NEG	NEG	NEG	NEG	NEG	NEG	NEG	NEG	NEG	NEG	NEG	NEG	NEG	NEG
MSSA2025	NEG	NEG	NEG	NEG	POS	NEG	NEG	NEG	NEG	NEG	NEG	NEG	NEG	NEG	NEG	NEG	NEG	NEG	NEG
MSSA2026	NEG	NEG	NEG	NEG	POS	NEG	NEG	NEG	NEG	NEG	NEG	NEG	NEG	NEG	NEG	NEG	NEG	NEG	NEG
MSSA2032	NEG	NEG	NEG	NEG	POS	NEG	NEG	NEG	NEG	NEG	NEG	NEG	NEG	NEG	NEG	NEG	NEG	NEG	NEG
MSSA2043	NEG	NEG	NEG	NEG	POS	NEG	NEG	NEG	NEG	NEG	NEG	NEG	NEG	NEG	NEG	NEG	NEG	NEG	NEG
MSSA2061	NEG	NEG	NEG	NEG	POS	NEG	NEG	NEG	NEG	NEG	NEG	NEG	NEG	NEG	NEG	NEG	NEG	NEG	NEG
MSSA2085	NEG	NEG	NEG	NEG	POS	NEG	NEG	NEG	NEG	NEG	NEG	NEG	NEG	NEG	NEG	NEG	NEG	NEG	NEG
MSSA2098	NEG	NEG	NEG	NEG	POS	NEG	NEG	NEG	NEG	NEG	NEG	NEG	NEG	NEG	NEG	NEG	NEG	NEG	NEG
MSSA2100	NEG	NEG	NEG	NEG	POS	NEG	NEG	NEG	NEG	NEG	NEG	NEG	NEG	NEG	NEG	NEG	NEG	NEG	NEG
MSSA2111	NEG	NEG	NEG	NEG	POS	NEG	NEG	NEG	NEG	NEG	NEG	NEG	NEG	NEG	NEG	NEG	NEG	NEG	NEG
MSSA2178	NEG	NEG	NEG	NEG	POS	NEG	NEG	NEG	NEG	NEG	NEG	NEG	NEG	NEG	NEG	NEG	NEG	NEG	NEG
MSSA2202	NEG	NEG	NEG	NEG	POS	NEG	NEG	NEG	NEG	NEG	NEG	NEG	NEG	NEG	NEG	NEG	NEG	NEG	NEG
MSSA2205	NEG	NEG	NEG	NEG	POS	NEG	NEG	NEG	NEG	NEG	NEG	NEG	NEG	NEG	NEG	NEG	NEG	NEG	NEG
MRSA3796	NEG	NEG	POS	NEG	NEG	NEG	NEG	NEG	NEG	NEG	NEG	NEG	NEG	AMB	NEG	NEG	NEG	NEG	NEG
MRSA3805	NEG	NEG	POS	NEG	NEG	NEG	NEG	NEG	NEG	NEG	NEG	NEG	NEG	POS	POS	NEG	NEG	NEG	NEG
MRSA3965	NEG	NEG	NEG	NEG	NEG	POS	NEG	NEG	NEG	NEG	NEG	NEG	NEG	POS	POS	NEG	NEG	NEG	NEG
MSSA1761	NEG	NEG	NEG	NEG	NEG	NEG	NEG	AMB	NEG	AMB	NEG	NEG	NEG	NEG	NEG	NEG	NEG	NEG	NEG
MSSA1764	NEG	NEG	POS	NEG	NEG	NEG	NEG	POS	NEG	NEG	NEG	NEG	POS	NEG	NEG	NEG	NEG	NEG	NEG
MSSA1765	NEG	NEG	NEG	NEG	NEG	NEG	NEG	NEG	NEG	NEG	NEG	NEG	NEG	NEG	NEG	NEG	NEG	NEG	NEG
MSSA1784	NEG	NEG	NEG	NEG	NEG	NEG	NEG	NEG	NEG	NEG	NEG	NEG	NEG	NEG	NEG	NEG	NEG	NEG	NEG
MSSA1806	NEG	NEG	NEG	NEG	POS	NEG	NEG	NEG	NEG	NEG	NEG	NEG	NEG	NEG	NEG	NEG	NEG	NEG	NEG
MSSA1807	NEG	NEG	NEG	NEG	NEG	NEG	NEG	NEG	NEG	NEG	NEG	NEG	NEG	NEG	NEG	NEG	NEG	NEG	NEG
MSSA1888	NEG	NEG	NEG	NEG	NEG	POS	NEG	POS	NEG	NEG	NEG	NEG	POS	POS	POS	NEG	NEG	NEG	NEG
MSSA1894	NEG	NEG	NEG	NEG	NEG	NEG	NEG	NEG	NEG	NEG	NEG	NEG	NEG	NEG	NEG	NEG	NEG	NEG	NEG
MSSA1922	NEG	NEG	NEG	NEG	NEG	NEG	NEG	POS	NEG	NEG	NEG	NEG	POS	NEG	NEG	NEG	NEG	NEG	NEG
MSSA1950	NEG	NEG	POS	NEG	NEG	POS	NEG	POS	NEG	NEG	NEG	NEG	POS	NEG	NEG	NEG	NEG	NEG	NEG
MSSA1954	NEG	NEG	POS	NEG	NEG	POS	NEG	POS	NEG	NEG	NEG	NEG	POS	NEG	NEG	NEG	NEG	NEG	NEG
MSSA1966	NEG	NEG	NEG	NEG	NEG	POS	NEG	POS	NEG	NEG	NEG	NEG	POS	POS	POS	NEG	NEG	NEG	NEG
MSSA1972	NEG	NEG	NEG	NEG	NEG	NEG	NEG	NEG	NEG	NEG	NEG	NEG	NEG	NEG	NEG	NEG	NEG	NEG	NEG
MSSA2010	NEG	NEG	NEG	NEG	NEG	NEG	NEG	POS	NEG	NEG	NEG	NEG	POS	NEG	NEG	NEG	NEG	NEG	NEG
MSSA2020	NEG	NEG	NEG	NEG	POS	NEG	NEG	NEG	NEG	NEG	NEG	NEG	NEG	NEG	NEG	NEG	NEG	NEG	NEG
MSSA2028	NEG	NEG	NEG	NEG	NEG	POS	NEG	NEG	NEG	NEG	NEG	NEG	NEG	POS	POS	NEG	NEG	NEG	NEG
MSSA2088	NEG	NEG	POS	NEG	NEG	NEG	NEG	NEG	NEG	NEG	NEG	NEG	NEG	NEG	NEG	NEG	NEG	NEG	NEG
MSSA2187	NEG	NEG	NEG	NEG	NEG	NEG	NEG	NEG	NEG	NEG	NEG	NEG	NEG	NEG	NEG	NEG	NEG	NEG	NEG
MSSA2204	NEG	NEG	NEG	NEG	NEG	NEG	NEG	POS	NEG	NEG	NEG	NEG	POS	NEG	NEG	NEG	NEG	NEG	NEG
MSSA2210	NEG	NEG	NEG	NEG	NEG	NEG	NEG	NEG	NEG	NEG	NEG	NEG	NEG	NEG	NEG	NEG	NEG	NEG	NEG
MSSA2223	NEG	NEG	NEG	NEG	NEG	NEG	NEG	POS	NEG	NEG	NEG	NEG	POS	NEG	NEG	NEG	NEG	NEG	NEG
MSSA1763	NEG	NEG	NEG	NEG	NEG	NEG	NEG	NEG	NEG	POS	NEG	POS	NEG	NEG	NEG	NEG	POS	POS	POS
MSSA1777	NEG	NEG	NEG	NEG	NEG	POS	NEG	NEG	NEG	POS	NEG	POS	NEG	NEG	NEG	NEG	POS	POS	POS
MSSA2009	NEG	NEG	NEG	NEG	NEG	NEG	NEG	NEG	NEG	POS	NEG	POS	NEG	NEG	NEG	NEG	POS	POS	POS
MSSA2047	NEG	NEG	NEG	NEG	NEG	POS	NEG	NEG	NEG	POS	NEG	POS	NEG	NEG	NEG	NEG	POS	POS	POS
MSSA2079	NEG	NEG	NEG	NEG	NEG	POS	NEG	NEG	NEG	POS	NEG	POS	NEG	NEG	NEG	NEG	POS	POS	POS
MSSA2101	NEG	NEG	NEG	NEG	NEG	POS	NEG	NEG	NEG	POS	NEG	POS	NEG	NEG	NEG	NEG	POS	POS	POS
MSSA1821	NEG	NEG	NEG	NEG	POS	POS	NEG	NEG	NEG	NEG	NEG	NEG	NEG	NEG	NEG	NEG	NEG	NEG	NEG
MSSA1898	NEG	NEG	NEG	NEG	POS	POS	NEG	NEG	NEG	NEG	NEG	NEG	NEG	NEG	NEG	NEG	NEG	NEG	NEG
MSSA1973	NEG	NEG	NEG	NEG	POS	POS	NEG	NEG	NEG	NEG	NEG	NEG	NEG	NEG	NEG	NEG	NEG	NEG	NEG
MSSA2071	NEG	NEG	NEG	NEG	POS	POS	NEG	NEG	NEG	NEG	NEG	NEG	NEG	NEG	NEG	NEG	NEG	NEG	NEG
MSSA2083	NEG	NEG	NEG	NEG	POS	POS	NEG	NEG	NEG	NEG	NEG	NEG	NEG	NEG	NEG	NEG	NEG	NEG	NEG
MSSA2106	NEG	NEG	NEG	NEG	POS	POS	NEG	NEG	NEG	NEG	NEG	NEG	NEG	NEG	NEG	NEG	NEG	NEG	NEG
MSSA2112	NEG	NEG	NEG	NEG	POS	NEG	NEG	NEG	NEG	NEG	NEG	NEG	NEG	NEG	NEG	NEG	NEG	NEG	NEG
MSSA2115	NEG	NEG	NEG	NEG	POS	NEG	POS	NEG	NEG	NEG	NEG	NEG	NEG	NEG	NEG	POS	NEG	NEG	NEG
MSSA2195	NEG	NEG	NEG	NEG	POS	NEG	NEG	AMB	NEG	NEG	NEG	NEG	NEG	NEG	NEG	NEG	NEG	NEG	NEG
MSSA2207	NEG	NEG	NEG	NEG	POS	POS	NEG	NEG	NEG	NEG	NEG	NEG	NEG	NEG	NEG	NEG	NEG	NEG	NEG
MSSA1758	NEG	NEG	NEG	NEG	NEG	NEG	NEG	NEG	NEG	NEG	NEG	NEG	NEG	NEG	NEG	NEG	NEG	NEG	NEG
MSSA1762	NEG	NEG	NEG	NEG	NEG	NEG	NEG	NEG	NEG	NEG	NEG	NEG	NEG	NEG	NEG	NEG	NEG	NEG	NEG
MSSA1769	NEG	NEG	NEG	NEG	NEG	NEG	NEG	NEG	NEG	NEG	NEG	NEG	NEG	NEG	NEG	NEG	NEG	NEG	NEG
MSSA1771	NEG	NEG	NEG	NEG	NEG	NEG	NEG	NEG	NEG	NEG	NEG	NEG	NEG	NEG	NEG	NEG	NEG	NEG	NEG
MSSA1789	NEG	NEG	NEG	NEG	NEG	NEG	NEG	NEG	NEG	NEG	NEG	NEG	NEG	NEG	NEG	NEG	NEG	NEG	NEG
MSSA1800	NEG	NEG	NEG	NEG	NEG	NEG	NEG	NEG	NEG	NEG	NEG	NEG	NEG	NEG	NEG	NEG	NEG	NEG	NEG
MSSA1833	NEG	NEG	NEG	NEG	NEG	NEG	NEG	NEG	NEG	NEG	NEG	NEG	NEG	NEG	NEG	NEG	NEG	NEG	NEG
MSSA1890	NEG	NEG	NEG	NEG	NEG	NEG	NEG	NEG	NEG	NEG	NEG	NEG	NEG	NEG	NEG	NEG	NEG	NEG	NEG
MSSA1903	NEG	NEG	NEG	NEG	NEG	NEG	NEG	NEG	NEG	NEG	NEG	NEG	NEG	NEG	NEG	NEG	NEG	NEG	NEG
MSSA1940	NEG	NEG	NEG	NEG	NEG	NEG	NEG	NEG	NEG	NEG	NEG	NEG	NEG	NEG	NEG	NEG	NEG	NEG	NEG
MSSA1962	NEG	NEG	NEG	NEG	NEG	NEG	NEG	NEG	NEG	NEG	NEG	NEG	NEG	NEG	NEG	NEG	NEG	NEG	NEG
MSSA1963	NEG	NEG	NEG	NEG	NEG	NEG	NEG	NEG	NEG	NEG	NEG	NEG	NEG	NEG	NEG	NEG	NEG	NEG	NEG
MSSA2016	NEG	NEG	NEG	NEG	NEG	NEG	NEG	NEG	NEG	NEG	NEG	NEG	NEG	NEG	NEG	NEG	NEG	NEG	NEG
MSSA2018	NEG	NEG	NEG	NEG	NEG	NEG	NEG	NEG	NEG	NEG	NEG	NEG	NEG	NEG	NEG	NEG	NEG	NEG	NEG
MSSA2019	NEG	NEG	NEG	NEG	NEG	NEG	NEG	NEG	NEG	NEG	NEG	NEG	NEG	NEG	NEG	NEG	NEG	NEG	NEG
MSSA2029	NEG	NEG	NEG	NEG	NEG	NEG	NEG	NEG	NEG	NEG	NEG	NEG	NEG	NEG	NEG	NEG	NEG	NEG	NEG
MSSA2045	NEG	NEG	NEG	NEG	NEG	NEG	NEG	NEG	NEG	NEG	NEG	NEG	NEG	NEG	NEG	NEG	NEG	NEG	NEG
MSSA2048	NEG	NEG	NEG	NEG	NEG	NEG	NEG	NEG	NEG	NEG	NEG	NEG	NEG	NEG	NEG	NEG	NEG	NEG	NEG
MSSA2049	NEG	NEG	NEG	NEG	NEG	NEG	NEG	NEG	NEG	NEG	NEG	NEG	NEG	NEG	NEG	NEG	NEG	NEG	NEG
MSSA2050	NEG	NEG	NEG	NEG	NEG	NEG	NEG	NEG	NEG	NEG	NEG	NEG	NEG	NEG	NEG	NEG	NEG	NEG	NEG
MSSA2099	NEG	NEG	NEG	NEG	NEG	NEG	NEG	NEG	NEG	NEG	NEG	NEG	NEG	NEG	NEG	NEG	NEG	NEG	NEG
MSSA2109	NEG	NEG	NEG	NEG	NEG	NEG	NEG	NEG	NEG	NEG	NEG	NEG	NEG	NEG	NEG	NEG	NEG	NEG	NEG
MSSA2130	NEG	NEG	NEG	NEG	NEG	NEG	NEG	NEG	NEG	NEG	NEG	NEG	NEG	NEG	NEG	NEG	NEG	NEG	NEG
MSSA2133	NEG	NEG	NEG	NEG	NEG	NEG	NEG	NEG	NEG	NEG	NEG	NEG	NEG	NEG	NEG	NEG	NEG	NEG	NEG
MSSA2139	NEG	NEG	NEG	NEG	NEG	NEG	NEG	NEG	NEG	NEG	NEG	NEG	NEG	NEG	NEG	NEG	NEG	NEG	NEG
MSSA2141	NEG	NEG	NEG	NEG	NEG	NEG	NEG	NEG	NEG	NEG	NEG	NEG	NEG	NEG	NEG	AMB	NEG	NEG	NEG
MSSA2208	NEG	NEG	NEG	NEG	NEG	NEG	NEG	NEG	NEG	NEG	NEG	NEG	NEG	NEG	NEG	NEG	NEG	NEG	NEG
MSSA2209	NEG	NEG	NEG	NEG	NEG	NEG	NEG	NEG	NEG	NEG	NEG	NEG	NEG	NEG	NEG	NEG	NEG	NEG	NEG
MSSA1893	NEG	NEG	NEG	NEG	NEG	NEG	NEG	NEG	NEG	POS	NEG	POS	NEG	NEG	NEG	NEG	POS	POS	POS
MSSA2105	NEG	NEG	NEG	NEG	NEG	NEG	NEG	NEG	NEG	POS	NEG	POS	NEG	NEG	NEG	NEG	POS	POS	POS
MRSA3836	NEG	NEG	NEG	NEG	NEG	NEG	NEG	NEG	NEG	POS	NEG	POS	NEG	NEG	NEG	NEG	POS	POS	POS
MRSA3840	NEG	NEG	NEG	NEG	NEG	NEG	POS	NEG	NEG	POS	NEG	POS	NEG	NEG	NEG	POS	POS	POS	POS
MRSA4188	NEG	NEG	NEG	NEG	NEG	AMB	POS	NEG	NEG	POS	NEG	POS	NEG	NEG	NEG	POS	POS	POS	POS
MSSA1814	NEG	NEG	NEG	NEG	NEG	NEG	NEG	NEG	NEG	POS	NEG	POS	NEG	NEG	NEG	AMB	POS	POS	POS
MSSA1816	NEG	NEG	NEG	NEG	NEG	NEG	POS	NEG	NEG	POS	NEG	POS	NEG	NEG	NEG	POS	POS	POS	POS
MSSA1825	NEG	NEG	NEG	NEG	NEG	NEG	NEG	NEG	NEG	POS	NEG	POS	NEG	NEG	NEG	NEG	POS	POS	POS
MSSA1834	NEG	NEG	NEG	NEG	NEG	NEG	POS	NEG	NEG	POS	NEG	POS	NEG	NEG	NEG	POS	POS	POS	POS
MSSA1938	NEG	NEG	NEG	NEG	NEG	NEG	NEG	NEG	NEG	POS	NEG	POS	NEG	NEG	NEG	NEG	POS	POS	POS
MSSA1948	POS	POS	NEG	NEG	NEG	NEG	NEG	NEG	NEG	POS	NEG	POS	NEG	NEG	NEG	NEG	POS	POS	POS
MSSA1951	NEG	NEG	NEG	NEG	NEG	NEG	NEG	NEG	NEG	POS	NEG	POS	NEG	NEG	NEG	NEG	POS	POS	POS
MSSA2005	NEG	NEG	NEG	NEG	NEG	NEG	NEG	NEG	NEG	POS	NEG	POS	NEG	NEG	NEG	NEG	POS	POS	POS
MSSA2051	NEG	NEG	NEG	NEG	NEG	NEG	NEG	NEG	NEG	POS	NEG	POS	NEG	NEG	NEG	NEG	POS	POS	POS
MSSA2056	NEG	NEG	NEG	NEG	NEG	NEG	NEG	NEG	NEG	POS	NEG	POS	NEG	NEG	NEG	NEG	POS	POS	POS
MSSA2066	NEG	NEG	NEG	NEG	NEG	NEG	NEG	NEG	NEG	POS	NEG	POS	NEG	NEG	NEG	NEG	POS	POS	POS
MSSA2067	NEG	NEG	NEG	NEG	NEG	NEG	NEG	NEG	NEG	POS	NEG	POS	NEG	NEG	NEG	NEG	POS	POS	POS
MSSA2086	NEG	NEG	NEG	NEG	NEG	NEG	NEG	NEG	NEG	POS	NEG	POS	NEG	NEG	NEG	NEG	POS	POS	POS
MSSA2089	NEG	NEG	NEG	NEG	NEG	NEG	NEG	NEG	NEG	POS	NEG	POS	NEG	NEG	NEG	NEG	POS	POS	POS
MSSA2103	NEG	NEG	NEG	NEG	NEG	NEG	NEG	NEG	NEG	POS	NEG	POS	NEG	NEG	NEG	NEG	POS	POS	POS
MSSA2173	NEG	NEG	NEG	NEG	NEG	NEG	NEG	NEG	NEG	POS	NEG	POS	NEG	NEG	NEG	NEG	POS	POS	POS
MSSA2183	NEG	NEG	NEG	NEG	NEG	NEG	NEG	NEG	NEG	POS	NEG	POS	NEG	NEG	NEG	NEG	POS	POS	POS
MSSA2196	NEG	NEG	NEG	NEG	NEG	NEG	NEG	NEG	NEG	POS	NEG	POS	NEG	NEG	NEG	NEG	POS	POS	POS
MSSA2219	NEG	NEG	NEG	NEG	NEG	AMB	POS	NEG	NEG	POS	NEG	POS	NEG	NEG	NEG	POS	POS	POS	POS
MSSA1753	NEG	NEG	NEG	NEG	NEG	POS	NEG	NEG	NEG	POS	NEG	POS	NEG	NEG	NEG	NEG	POS	POS	POS
MSSA1773	NEG	NEG	NEG	NEG	NEG	NEG	NEG	NEG	NEG	POS	NEG	POS	NEG	NEG	NEG	NEG	POS	POS	POS
MSSA1811	NEG	NEG	NEG	NEG	NEG	NEG	NEG	NEG	NEG	POS	NEG	POS	NEG	NEG	NEG	NEG	POS	POS	POS
MSSA1897	NEG	NEG	NEG	NEG	NEG	NEG	NEG	NEG	NEG	POS	NEG	POS	NEG	NEG	NEG	NEG	POS	POS	POS
MSSA1931	NEG	NEG	NEG	NEG	NEG	POS	NEG	NEG	NEG	POS	NEG	POS	NEG	NEG	NEG	NEG	POS	POS	POS
MSSA2114	NEG	NEG	NEG	NEG	NEG	NEG	POS	NEG	NEG	POS	NEG	POS	NEG	NEG	NEG	POS	POS	POS	POS
MSSA1745	POS	POS	POS	NEG	NEG	NEG	NEG	NEG	NEG	POS	NEG	POS	NEG	NEG	NEG	NEG	POS	POS	POS
MSSA1747	POS	POS	POS	NEG	NEG	NEG	NEG	NEG	NEG	POS	NEG	POS	NEG	NEG	NEG	NEG	POS	POS	POS
MSSA1749	POS	POS	POS	NEG	NEG	NEG	NEG	NEG	NEG	POS	NEG	POS	NEG	NEG	NEG	NEG	POS	POS	POS
MSSA1768	POS	POS	NEG	NEG	NEG	NEG	POS	NEG	NEG	POS	NEG	POS	NEG	NEG	NEG	POS	POS	POS	POS
MSSA1774	POS	POS	POS	NEG	NEG	NEG	NEG	NEG	NEG	POS	NEG	POS	NEG	NEG	NEG	NEG	POS	POS	POS
MSSA1775	NEG	NEG	NEG	NEG	NEG	NEG	NEG	NEG	NEG	POS	NEG	POS	NEG	NEG	NEG	NEG	POS	POS	POS
MSSA1776	NEG	NEG	NEG	NEG	NEG	NEG	NEG	NEG	NEG	POS	NEG	POS	NEG	NEG	NEG	NEG	POS	POS	POS
MSSA1779	POS	POS	NEG	NEG	NEG	NEG	NEG	NEG	NEG	POS	NEG	POS	NEG	NEG	NEG	NEG	POS	POS	POS
MSSA1780	NEG	NEG	POS	NEG	NEG	NEG	NEG	NEG	NEG	POS	NEG	POS	NEG	NEG	NEG	NEG	POS	POS	POS

	VIRULENCE: TOX.SCHOCK		VIRULENCE: ENTEROTOXINS																
Strain	tst1 (cons)	tst1 (other than RF122)	sea = entA	sea = entA (320E)	sea = entA (N315) = entP	seb = entB	sec = entC	sed = entD	see = entE	seg = entG	seh = entH	sei = entI	sej = entJ	sek = entK (probe 1)	sek = entK (probe 2)	sel = entL	sem = entM	sen = entN	sen = entN other than RF122
~~MSSA1781~~	POS	POS	POS	NEG	NEG	NEG	NEG	NEG	NEG	POS	NEG	POS	NEG	NEG	NEG	NEG	POS	POS	POS
MSSA1786	NEG	NEG	NEG	NEG	NEG	NEG	NEG	NEG	NEG	POS	NEG	POS	NEG	NEG	NEG	NEG	POS	POS	POS
MSSA1793	POS	POS	POS	NEG	NEG	NEG	NEG	NEG	NEG	POS	NEG	POS	NEG	NEG	NEG	NEG	POS	POS	POS
MSSA1803	POS	POS	NEG	NEG	NEG	NEG	NEG	NEG	NEG	POS	NEG	POS	NEG	NEG	NEG	NEG	POS	POS	POS
MSSA1836	NEG	NEG	POS	NEG	NEG	NEG	NEG	NEG	NEG	POS	NEG	POS	NEG	NEG	NEG	NEG	POS	POS	POS
MSSA1839	POS	POS	POS	NEG	NEG	NEG	NEG	NEG	NEG	POS	NEG	POS	NEG	NEG	NEG	NEG	POS	POS	POS
MSSA1900	POS	POS	POS	NEG	NEG	NEG	NEG	NEG	NEG	POS	NEG	POS	NEG	NEG	NEG	NEG	POS	POS	POS
MSSA1910	POS	POS	NEG	NEG	NEG	NEG	NEG	NEG	NEG	POS	POS	POS	NEG	NEG	NEG	NEG	POS	POS	POS
MSSA1918	POS	POS	NEG	NEG	NEG	NEG	NEG	NEG	NEG	POS	POS	POS	NEG	NEG	NEG	NEG	POS	POS	POS
MSSA1921	NEG	NEG	NEG	NEG	NEG	NEG	NEG	NEG	NEG	POS	NEG	POS	NEG	NEG	NEG	NEG	POS	POS	POS
MSSA1923	POS	POS	NEG	NEG	NEG	NEG	POS	NEG	NEG	POS	NEG	POS	NEG	NEG	NEG	POS	POS	POS	POS
MSSA1981	POS	POS	NEG	NEG	NEG	NEG	NEG	NEG	NEG	POS	POS	POS	NEG	NEG	NEG	NEG	POS	POS	POS
MSSA1998	NEG	NEG	NEG	NEG	NEG	NEG	NEG	NEG	NEG	POS	NEG	POS	NEG	NEG	NEG	NEG	POS	POS	POS
MSSA2000	POS	POS	POS	NEG	NEG	NEG	NEG	NEG	NEG	POS	POS	POS	NEG	NEG	NEG	NEG	POS	POS	POS
MSSA2007	POS	POS	POS	NEG	NEG	NEG	NEG	NEG	NEG	POS	NEG	POS	NEG	NEG	NEG	NEG	POS	POS	POS
MSSA2012	POS	POS	NEG	NEG	NEG	NEG	NEG	NEG	NEG	POS	NEG	POS	NEG	NEG	NEG	NEG	POS	POS	POS
MSSA2015	POS	POS	POS	NEG	NEG	NEG	NEG	NEG	NEG	POS	NEG	POS	NEG	NEG	NEG	NEG	POS	POS	POS
MSSA2021	NEG	NEG	NEG	NEG	NEG	NEG	NEG	NEG	NEG	POS	NEG	POS	NEG	NEG	NEG	NEG	POS	POS	POS
MSSA2027	POS	POS	POS	NEG	NEG	NEG	NEG	NEG	NEG	POS	NEG	POS	NEG	NEG	NEG	NEG	POS	POS	POS
MSSA2063	POS	POS	POS	NEG	NEG	NEG	NEG	NEG	NEG	POS	NEG	POS	NEG	NEG	NEG	NEG	POS	POS	POS
MSSA2064	POS	POS	NEG	NEG	NEG	NEG	NEG	NEG	NEG	POS	NEG	POS	NEG	NEG	NEG	NEG	POS	POS	POS
MSSA2065	POS	POS	POS	NEG	NEG	NEG	NEG	NEG	NEG	POS	NEG	POS	NEG	NEG	NEG	NEG	POS	POS	POS
MSSA2075	POS	POS	POS	NEG	NEG	NEG	NEG	NEG	NEG	POS	NEG	POS	NEG	NEG	NEG	NEG	POS	POS	POS
MSSA2093	POS	POS	NEG	NEG	NEG	NEG	NEG	NEG	NEG	POS	POS	POS	NEG	NEG	NEG	NEG	POS	POS	POS
MSSA2102	POS	POS	NEG	NEG	NEG	NEG	NEG	NEG	NEG	POS	NEG	POS	NEG	NEG	NEG	NEG	POS	POS	POS
MSSA2138	POS	POS	NEG	NEG	NEG	NEG	NEG	NEG	NEG	POS	NEG	POS	NEG	NEG	NEG	NEG	POS	POS	POS
MSSA2168	POS	POS	POS	NEG	NEG	NEG	NEG	NEG	NEG	POS	NEG	POS	NEG	NEG	NEG	NEG	POS	POS	POS
MSSA2181	POS	POS	NEG	NEG	NEG	NEG	NEG	NEG	NEG	POS	NEG	POS	NEG	NEG	NEG	NEG	POS	POS	POS
MSSA2189	POS	POS	NEG	NEG	NEG	NEG	NEG	NEG	NEG	POS	POS	POS	NEG	NEG	NEG	NEG	POS	POS	POS
MSSA2214	POS	POS	NEG	NEG	NEG	NEG	NEG	NEG	NEG	POS	NEG	POS	NEG	NEG	NEG	NEG	POS	POS	POS
MRSA3803	NEG	NEG	NEG	NEG	NEG	NEG	POS	NEG	NEG	POS	NEG	POS	NEG	NEG	NEG	POS	POS	POS	POS
MSSA1750	NEG	NEG	NEG	NEG	NEG	NEG	NEG	NEG	NEG	POS	NEG	POS	NEG	NEG	NEG	NEG	POS	POS	POS
MSSA1756	NEG	NEG	NEG	NEG	NEG	NEG	POS	NEG	NEG	POS	NEG	POS	NEG	NEG	NEG	POS	POS	POS	POS
MSSA1759	NEG	NEG	NEG	NEG	NEG	NEG	POS	NEG	NEG	POS	NEG	POS	NEG	NEG	NEG	POS	POS	POS	POS
MSSA1767	NEG	NEG	NEG	NEG	NEG	NEG	NEG	NEG	NEG	POS	NEG	POS	NEG	NEG	NEG	NEG	POS	POS	POS
MSSA1787	NEG	NEG	NEG	NEG	NEG	POS	POS	NEG	NEG	POS	NEG	POS	NEG	NEG	NEG	POS	POS	POS	POS
MSSA1790	NEG	NEG	NEG	NEG	NEG	NEG	NEG	NEG	NEG	POS	NEG	POS	NEG	NEG	NEG	NEG	POS	POS	POS
MSSA1794	NEG	NEG	NEG	NEG	NEG	NEG	POS	NEG	NEG	POS	NEG	POS	NEG	NEG	NEG	POS	POS	POS	POS
MSSA1808	NEG	NEG	NEG	NEG	NEG	NEG	POS	NEG	NEG	POS	NEG	POS	NEG	NEG	NEG	POS	POS	POS	POS
MSSA1812	NEG	NEG	NEG	NEG	NEG	NEG	NEG	NEG	NEG	POS	NEG	POS	NEG	NEG	NEG	NEG	POS	POS	POS
MSSA1819	NEG	NEG	NEG	NEG	NEG	NEG	POS	NEG	NEG	POS	NEG	POS	NEG	NEG	NEG	POS	POS	POS	POS
MSSA1824	NEG	NEG	NEG	NEG	NEG	NEG	NEG	NEG	NEG	POS	NEG	POS	NEG	NEG	NEG	NEG	POS	POS	POS
MSSA1827	NEG	NEG	NEG	NEG	NEG	NEG	NEG	NEG	NEG	POS	NEG	POS	NEG	NEG	NEG	NEG	POS	POS	POS
MSSA1889	NEG	NEG	NEG	NEG	NEG	NEG	POS	NEG	NEG	POS	NEG	POS	NEG	NEG	NEG	POS	POS	POS	POS
MSSA1895	NEG	NEG	NEG	NEG	NEG	NEG	NEG	NEG	NEG	POS	NEG	POS	NEG	NEG	NEG	NEG	POS	POS	POS
MSSA1904	NEG	NEG	NEG	NEG	NEG	NEG	POS	NEG	NEG	POS	NEG	POS	NEG	NEG	NEG	POS	POS	POS	POS
MSSA1905	NEG	NEG	NEG	NEG	NEG	NEG	NEG	NEG	NEG	POS	NEG	POS	NEG	NEG	NEG	NEG	POS	POS	POS
MSSA1907	NEG	NEG	NEG	NEG	NEG	NEG	POS	NEG	NEG	POS	NEG	POS	NEG	NEG	NEG	POS	POS	POS	POS
MSSA1919	NEG	NEG	NEG	NEG	NEG	NEG	POS	NEG	NEG	POS	NEG	POS	NEG	NEG	NEG	POS	POS	POS	POS
MSSA1920	NEG	NEG	NEG	NEG	NEG	NEG	NEG	NEG	NEG	POS	NEG	POS	NEG	NEG	NEG	NEG	POS	POS	POS
MSSA1929	NEG	NEG	NEG	NEG	NEG	NEG	POS	NEG	NEG	POS	NEG	POS	NEG	NEG	NEG	POS	POS	POS	POS
MSSA1930	NEG	NEG	NEG	NEG	NEG	NEG	POS	NEG	NEG	POS	NEG	POS	NEG	NEG	NEG	POS	POS	POS	POS
MSSA1934	NEG	NEG	NEG	NEG	NEG	NEG	POS	NEG	NEG	POS	NEG	POS	NEG	NEG	NEG	POS	POS	POS	POS
MSSA1946	NEG	NEG	NEG	NEG	NEG	NEG	NEG	NEG	NEG	POS	NEG	POS	NEG	NEG	NEG	NEG	POS	POS	POS
MSSA1971	NEG	NEG	NEG	NEG	NEG	NEG	NEG	NEG	NEG	POS	NEG	POS	NEG	NEG	NEG	NEG	POS	POS	POS
MSSA1980	NEG	NEG	NEG	NEG	NEG	NEG	POS	NEG	NEG	POS	NEG	POS	NEG	NEG	NEG	POS	POS	POS	POS
MSSA1982	NEG	NEG	NEG	NEG	NEG	NEG	POS	NEG	NEG	POS	NEG	POS	NEG	NEG	NEG	POS	POS	POS	POS
MSSA1996	NEG	NEG	NEG	NEG	NEG	NEG	POS	NEG	NEG	POS	NEG	POS	NEG	NEG	NEG	POS	POS	POS	POS
MSSA2002	NEG	NEG	NEG	NEG	NEG	NEG	NEG	NEG	NEG	POS	NEG	POS	NEG	NEG	NEG	NEG	POS	POS	POS
MSSA2014	NEG	NEG	NEG	NEG	NEG	NEG	POS	NEG	NEG	POS	NEG	POS	NEG	NEG	NEG	POS	POS	POS	POS
MSSA2053	NEG	NEG	NEG	NEG	NEG	POS	POS	NEG	NEG	POS	NEG	POS	NEG	NEG	NEG	POS	POS	POS	POS
MSSA2054	NEG	NEG	NEG	NEG	NEG	NEG	POS	NEG	NEG	POS	NEG	POS	NEG	NEG	NEG	POS	POS	POS	POS
MSSA2058	NEG	NEG	NEG	NEG	NEG	NEG	POS	NEG	NEG	POS	NEG	POS	NEG	NEG	NEG	POS	POS	POS	POS
MSSA2068	NEG	NEG	NEG	NEG	NEG	NEG	POS	NEG	NEG	POS	NEG	POS	NEG	NEG	NEG	POS	POS	POS	POS
MSSA2070	NEG	NEG	NEG	NEG	NEG	NEG	POS	NEG	NEG	POS	NEG	POS	NEG	NEG	NEG	POS	POS	POS	POS
MSSA2072	NEG	NEG	NEG	NEG	NEG	NEG	NEG	NEG	NEG	POS	NEG	POS	NEG	NEG	NEG	NEG	POS	POS	POS
MSSA2073	NEG	NEG	NEG	NEG	NEG	NEG	NEG	NEG	NEG	POS	NEG	POS	NEG	NEG	NEG	NEG	POS	POS	POS
MSSA2074	NEG	NEG	NEG	NEG	NEG	NEG	POS	NEG	NEG	POS	NEG	POS	NEG	NEG	NEG	POS	POS	POS	POS
MSSA2107	NEG	NEG	NEG	NEG	NEG	NEG	POS	NEG	NEG	NEG	NEG	POS	NEG	NEG	NEG	POS	POS	POS	POS
MSSA2108	NEG	NEG	NEG	NEG	NEG	NEG	NEG	NEG	NEG	POS	NEG	POS	NEG	NEG	NEG	NEG	POS	POS	POS
MSSA2129	NEG	NEG	NEG	NEG	NEG	NEG	POS	NEG	NEG	POS	NEG	POS	NEG	NEG	NEG	POS	POS	POS	POS
MSSA2135	NEG	NEG	NEG	NEG	NEG	NEG	NEG	NEG	NEG	POS	NEG	POS	NEG	NEG	NEG	NEG	POS	POS	POS
MSSA2137	NEG	NEG	NEG	NEG	NEG	NEG	NEG	NEG	NEG	POS	NEG	POS	NEG	NEG	NEG	NEG	POS	POS	POS
MSSA2140	NEG	NEG	NEG	NEG	NEG	NEG	POS	NEG	NEG	POS	NEG	POS	NEG	NEG	NEG	POS	POS	POS	POS
MSSA2182	NEG	NEG	NEG	NEG	NEG	NEG	NEG	NEG	NEG	POS	NEG	POS	NEG	NEG	NEG	NEG	POS	POS	POS
MSSA2188	NEG	NEG	NEG	NEG	NEG	NEG	POS	NEG	NEG	POS	AMB	POS	NEG	NEG	NEG	POS	POS	POS	POS
MSSA1899	NEG	NEG	NEG	NEG	NEG	POS	NEG	NEG	NEG	NEG	NEG	NEG	NEG	POS	POS	NEG	NEG	NEG	NEG
MSSA1915	NEG	NEG	NEG	NEG	NEG	POS	NEG	NEG	NEG	NEG	NEG	NEG	NEG	POS	POS	NEG	NEG	NEG	NEG
MSSA1949	NEG	NEG	POS	NEG	NEG	POS	NEG	NEG	NEG	NEG	NEG	NEG	NEG	POS	POS	NEG	NEG	NEG	NEG
MSSA2042	NEG	NEG	NEG	NEG	NEG	POS	NEG	NEG	NEG	NEG	NEG	NEG	NEG	POS	POS	NEG	NEG	NEG	NEG
MSSA2077	NEG	NEG	NEG	NEG	NEG	NEG	NEG	NEG	NEG	NEG	NEG	NEG	NEG	NEG	NEG	NEG	NEG	NEG	NEG
MSSA2095	NEG	NEG	NEG	NEG	NEG	POS	NEG	NEG	NEG	NEG	NEG	NEG	NEG	POS	POS	NEG	NEG	NEG	NEG
MSSA1997	NEG	NEG	NEG	NEG	NEG	NEG	NEG	NEG	NEG	NEG	NEG	NEG	NEG	NEG	NEG	NEG	NEG	NEG	NEG
MSSA1810	NEG	NEG	NEG	NEG	NEG	NEG	NEG	NEG	NEG	NEG	NEG	NEG	NEG	NEG	NEG	NEG	NEG	NEG	NEG
MSSA1832	NEG	NEG	NEG	NEG	NEG	NEG	POS	NEG	NEG	NEG	NEG	NEG	NEG	NEG	NEG	POS	NEG	NEG	NEG
MSSA1891	NEG	NEG	NEG	NEG	NEG	NEG	NEG	NEG	NEG	NEG	NEG	NEG	NEG	NEG	NEG	NEG	NEG	NEG	NEG
MSSA1892	NEG	NEG	NEG	NEG	NEG	NEG	POS	NEG	NEG	NEG	NEG	NEG	NEG	NEG	NEG	POS	NEG	AMB	NEG
MSSA1932	NEG	NEG	NEG	NEG	NEG	NEG	POS	NEG	NEG	NEG	NEG	NEG	NEG	NEG	NEG	POS	NEG	NEG	NEG
MSSA1942	NEG	NEG	NEG	NEG	NEG	NEG	NEG	NEG	NEG	NEG	NEG	NEG	NEG	NEG	NEG	NEG	NEG	NEG	NEG
MSSA1957	NEG	NEG	NEG	NEG	NEG	NEG	NEG	NEG	NEG	NEG	NEG	NEG	NEG	NEG	NEG	NEG	NEG	NEG	NEG
MSSA1748	NEG	NEG	NEG	NEG	NEG	NEG	NEG	NEG	NEG	NEG	NEG	NEG	NEG	NEG	NEG	NEG	NEG	NEG	NEG
MSSA1754	NEG	NEG	NEG	NEG	NEG	NEG	NEG	NEG	NEG	NEG	NEG	NEG	NEG	NEG	NEG	NEG	NEG	NEG	NEG
MSSA1770	NEG	NEG	NEG	NEG	NEG	NEG	NEG	NEG	NEG	NEG	NEG	NEG	NEG	NEG	NEG	NEG	NEG	NEG	NEG
MSSA1788	NEG	NEG	NEG	NEG	NEG	NEG	NEG	NEG	NEG	NEG	NEG	NEG	NEG	NEG	NEG	NEG	NEG	NEG	NEG
MSSA1804	NEG	NEG	NEG	NEG	NEG	NEG	NEG	NEG	NEG	NEG	NEG	NEG	NEG	NEG	NEG	NEG	NEG	NEG	NEG
MSSA1818	NEG	NEG	NEG	NEG	NEG	NEG	NEG	NEG	NEG	NEG	NEG	NEG	NEG	NEG	NEG	NEG	NEG	NEG	NEG
MSSA1914	NEG	NEG	NEG	NEG	NEG	NEG	NEG	NEG	NEG	NEG	NEG	NEG	NEG	NEG	NEG	NEG	NEG	NEG	NEG
MSSA1974	NEG	NEG	NEG	NEG	NEG	NEG	NEG	NEG	NEG	NEG	NEG	NEG	NEG	NEG	NEG	NEG	NEG	NEG	NEG
MSSA2023	NEG	NEG	NEG	NEG	NEG	NEG	NEG	NEG	NEG	NEG	NEG	NEG	NEG	NEG	NEG	NEG	NEG	NEG	NEG
MSSA2030	NEG	NEG	NEG	NEG	NEG	NEG	NEG	NEG	NEG	NEG	NEG	NEG	NEG	NEG	NEG	NEG	NEG	NEG	NEG
MSSA2096	NEG	NEG	NEG	NEG	NEG	NEG	NEG	NEG	NEG	NEG	NEG	NEG	NEG	NEG	NEG	NEG	NEG	NEG	NEG
MSSA2142	NEG	NEG	NEG	NEG	NEG	NEG	NEG	NEG	NEG	NEG	NEG	NEG	NEG	NEG	NEG	NEG	NEG	NEG	NEG
MSSA2203	NEG	NEG	NEG	NEG	NEG	NEG	NEG	NEG	NEG	NEG	NEG	NEG	NEG	NEG	NEG	NEG	NEG	NEG	NEG
MSSA1901	NEG	NEG	NEG	NEG	NEG	NEG	NEG	NEG	NEG	POS	NEG	POS	NEG	NEG	NEG	NEG	POS	POS	POS
MSSA2013	NEG	NEG	NEG	NEG	NEG	NEG	NEG	NEG	NEG	POS	NEG	POS	NEG	NEG	NEG	NEG	POS	POS	POS
MSSA2031	NEG	NEG	NEG	NEG	NEG	POS	NEG	NEG	NEG	POS	NEG	POS	NEG	NEG	NEG	NEG	POS	POS	POS
MSSA2052	NEG	NEG	NEG	NEG	NEG	NEG	NEG	NEG	NEG	POS	NEG	POS	NEG	NEG	NEG	NEG	POS	POS	POS
MSSA2057	NEG	NEG	NEG	NEG	NEG	NEG	NEG	NEG	NEG	POS	NEG	POS	NEG	NEG	NEG	NEG	POS	POS	POS
MSSA2081	NEG	NEG	NEG	NEG	NEG	NEG	NEG	NEG	NEG	NEG	NEG	POS	NEG	NEG	NEG	NEG	POS	POS	NEG
MSSA1955	NEG	NEG	NEG	NEG	NEG	NEG	NEG	NEG	NEG	POS	POS	POS	NEG	NEG	NEG	NEG	POS	AMB	POS
MSSA1941	POS	POS	POS	NEG	NEG	NEG	POS	NEG	NEG	NEG	NEG	NEG	NEG	POS	POS	POS	NEG	NEG	NEG
MRSA3838	NEG	NEG	NEG	NEG	NEG	NEG	NEG	NEG	NEG	NEG	NEG	NEG	NEG	NEG	NEG	NEG	NEG	NEG	NEG
MRSA3839	NEG	NEG	NEG	NEG	NEG	NEG	NEG	NEG	NEG	NEG	NEG	NEG	NEG	NEG	NEG	NEG	NEG	NEG	NEG
MSSA1801	NEG	NEG	AMB	NEG	NEG	NEG	NEG	NEG	NEG	NEG	NEG	NEG	NEG	NEG	NEG	NEG	NEG	NEG	NEG
MSSA2131	NEG	NEG	NEG	NEG	NEG	NEG	NEG	NEG	NEG	NEG	NEG	NEG	NEG	NEG	NEG	NEG	NEG	NEG	NEG
MSSA1792	NEG	NEG	NEG	NEG	NEG	NEG	NEG	NEG	NEG	POS	NEG	POS	NEG	POS	POS	NEG	POS	POS	POS
MSSA1796	NEG	NEG	NEG	NEG	NEG	NEG	NEG	AMB	NEG	POS	NEG	NEG	NEG	NEG	NEG	NEG	NEG	NEG	NEG
MSSA1817	NEG	NEG	NEG	NEG	NEG	NEG	NEG	NEG	NEG	NEG	NEG	NEG	NEG	NEG	NEG	NEG	NEG	NEG	NEG
MSSA1911	NEG	NEG	NEG	NEG	NEG	NEG	NEG	NEG	NEG	NEG	NEG	NEG	NEG	NEG	NEG	NEG	NEG	NEG	NEG
MSSA1927	NEG	NEG	POS	NEG	NEG	NEG	NEG	NEG	NEG	NEG	NEG	NEG	NEG	NEG	NEG	NEG	NEG	NEG	NEG
MSSA1928	NEG	NEG	POS	NEG	NEG	NEG	NEG	NEG	NEG	NEG	NEG	NEG	NEG	NEG	NEG	NEG	NEG	NEG	NEG
MSSA1953	NEG	NEG	POS	NEG	NEG	NEG	NEG	NEG	NEG	NEG	NEG	NEG	NEG	NEG	NEG	NEG	NEG	NEG	NEG
MSSA2062	NEG	NEG	NEG	NEG	NEG	NEG	NEG	NEG	NEG	POS	NEG	POS	NEG	NEG	NEG	NEG	POS	POS	POS
MSSA2078	NEG	NEG	NEG	NEG	NEG	NEG	NEG	NEG	NEG	POS	NEG	POS	NEG	NEG	NEG	NEG	POS	POS	POS
MSSA2136	NEG	NEG	NEG	NEG	NEG	NEG	NEG	NEG	NEG	NEG	NEG	NEG	NEG	NEG	NEG	NEG	NEG	NEG	NEG

	VIRULENCE: ENTEROTOXINS, continued							VIRULENCE: HLG AND LEUKOCIDIN												
Strain	seo = entO	seq = entQ (probe 1)	seq = entQ (probe 2)	ser = entR	seu = entU	ORF CM14 (probe 1)	ORF CM14 (probe 2)	lukF	lukS	lukS (ST22+45)	hlgA	lukF-PV	lukF-PV (P83)	lukS-PV	lukM	lukD	lukE	lukX	lukY (var1)	lukY (var2)
MSSA1805	NEG	NEG	NEG	NEG	NEG	NEG	NEG	POS	POS	AMB	POS	NEG	NEG	NEG	NEG	POS	POS	POS	POS	NEG
MSSA1823	NEG	NEG	NEG	NEG	NEG	NEG	NEG	POS	POS	AMB	POS	NEG	NEG	NEG	NEG	POS	POS	POS	POS	NEG
MSSA1837	NEG	POS	POS	NEG	NEG	NEG	NEG	POS	POS	AMB	POS	NEG	NEG	NEG	NEG	POS	POS	POS	POS	NEG
MSSA2044	NEG	POS	POS	NEG	NEG	NEG	NEG	POS	POS	AMB	POS	NEG	NEG	NEG	NEG	POS	POS	POS	POS	NEG
MSSA2046	NEG	NEG	NEG	NEG	NEG	NEG	NEG	POS	POS	AMB	POS	NEG	NEG	NEG	NEG	POS	POS	POS	POS	NEG
MSSA2059	NEG	POS	POS	NEG	NEG	NEG	NEG	POS	POS	NEG	POS	NEG	NEG	NEG	NEG	POS	POS	POS	POS	NEG
MSSA2076	NEG	NEG	NEG	NEG	NEG	NEG	NEG	POS	POS	NEG	POS	NEG	NEG	NEG	NEG	POS	POS	POS	POS	NEG
MSSA2200	NEG	POS	POS	NEG	NEG	NEG	NEG	POS	POS	AMB	POS	NEG	NEG	NEG	NEG	POS	POS	POS	POS	NEG
MRSA3797	POS	NEG	NEG	POS	POS	NEG	NEG	POS	POS	NEG	POS	NEG	NEG	NEG	NEG	POS	POS	POS	POS	NEG
MRSA3798	POS	NEG	NEG	POS	POS	NEG	NEG	POS	POS	AMB	POS	NEG	NEG	NEG	NEG	POS	POS	POS	POS	NEG
MRSA3799	POS	NEG	NEG	POS	POS	NEG	NEG	POS	POS	AMB	POS	NEG	NEG	NEG	NEG	POS	POS	POS	POS	NEG
MRSA3800	POS	NEG	NEG	POS	POS	NEG	NEG	POS	POS	AMB	POS	NEG	NEG	NEG	NEG	POS	POS	POS	POS	NEG
MRSA3801	POS	NEG	NEG	POS	POS	NEG	NEG	POS	POS	AMB	POS	NEG	NEG	NEG	NEG	POS	POS	POS	POS	NEG
MRSA3804	POS	NEG	NEG	POS	POS	NEG	NEG	POS	POS	AMB	POS	NEG	NEG	NEG	NEG	POS	POS	POS	POS	NEG
MRSA3806	POS	NEG	NEG	POS	POS	NEG	NEG	POS	POS	AMB	POS	NEG	NEG	NEG	NEG	POS	POS	POS	POS	NEG
MRSA3961	POS	NEG	NEG	POS	POS	NEG	NEG	POS	POS	AMB	POS	NEG	NEG	NEG	NEG	POS	POS	POS	POS	NEG
MRSA3962	POS	NEG	NEG	NEG	POS	NEG	NEG	POS	POS	AMB	POS	NEG	NEG	NEG	NEG	POS	POS	POS	POS	NEG
MRSA3963	POS	NEG	NEG	NEG	POS	NEG	NEG	POS	POS	AMB	POS	NEG	NEG	NEG	NEG	POS	POS	POS	POS	NEG
MRSA3966	POS	NEG	NEG	NEG	POS	NEG	NEG	POS	POS	NEG	POS	NEG	NEG	NEG	NEG	POS	POS	POS	POS	NEG
MRSA3978	POS	NEG	NEG	POS	POS	NEG	NEG	POS	POS	AMB	POS	NEG	NEG	NEG	NEG	POS	POS	POS	POS	NEG
MRSA3979	POS	NEG	NEG	POS	POS	NEG	NEG	POS	POS	NEG	POS	NEG	NEG	NEG	NEG	POS	POS	POS	POS	NEG
MRSA3980	POS	NEG	NEG	POS	POS	NEG	NEG	POS	POS	NEG	POS	NEG	NEG	NEG	NEG	POS	POS	POS	POS	NEG
MRSA4179	POS	NEG	NEG	NEG	POS	NEG	NEG	POS	POS	AMB	POS	NEG	NEG	NEG	NEG	POS	POS	POS	POS	NEG
MRSA4216	POS	NEG	NEG	POS	POS	NEG	NEG	POS	POS	AMB	POS	NEG	NEG	NEG	NEG	POS	POS	POS	POS	NEG
MRSA4217	POS	NEG	NEG	POS	POS	NEG	NEG	POS	POS	AMB	POS	NEG	NEG	NEG	NEG	POS	POS	POS	POS	NEG
MRSA4220	POS	NEG	NEG	NEG	POS	NEG	NEG	POS	POS	AMB	POS	NEG	NEG	NEG	NEG	POS	POS	POS	POS	NEG
MRSA4221	POS	NEG	NEG	POS	POS	NEG	NEG	POS	POS	AMB	POS	NEG	NEG	NEG	NEG	POS	POS	POS	POS	NEG
MRSA4222	POS	NEG	NEG	POS	POS	NEG	NEG	POS	POS	AMB	POS	NEG	NEG	NEG	NEG	POS	POS	POS	POS	NEG
MSSA1751	POS	NEG	NEG	POS	POS	NEG	NEG	POS	POS	AMB	POS	NEG	NEG	NEG	NEG	POS	POS	POS	POS	NEG
MSSA1760	POS	NEG	NEG	NEG	POS	NEG	NEG	POS	POS	AMB	POS	NEG	NEG	NEG	NEG	POS	POS	POS	POS	NEG
MSSA1802	POS	NEG	NEG	POS	POS	NEG	NEG	POS	POS	AMB	POS	NEG	NEG	NEG	NEG	POS	POS	POS	POS	NEG
MSSA1809	POS	NEG	NEG	NEG	POS	NEG	NEG	POS	POS	AMB	POS	NEG	NEG	NEG	NEG	POS	POS	POS	POS	NEG
MSSA1815	POS	NEG	NEG	NEG	POS	NEG	NEG	POS	POS	AMB	POS	NEG	NEG	NEG	NEG	POS	POS	POS	POS	NEG
MSSA1829	POS	NEG	NEG	NEG	POS	NEG	NEG	POS	POS	AMB	POS	NEG	NEG	NEG	NEG	POS	POS	POS	POS	NEG
MSSA1908	POS	NEG	NEG	NEG	POS	NEG	NEG	POS	POS	AMB	POS	NEG	NEG	NEG	NEG	POS	POS	POS	POS	NEG
MSSA1960	POS	NEG	NEG	NEG	POS	NEG	NEG	POS	POS	NEG	POS	NEG	NEG	NEG	NEG	POS	POS	POS	POS	NEG
MSSA1965	POS	NEG	NEG	NEG	POS	NEG	NEG	POS	POS	AMB	POS	NEG	NEG	NEG	NEG	POS	POS	POS	POS	NEG
MSSA1977	POS	NEG	NEG	NEG	POS	NEG	NEG	POS	POS	AMB	POS	NEG	NEG	NEG	NEG	POS	POS	POS	POS	NEG
MSSA1978	POS	NEG	NEG	NEG	POS	NEG	NEG	POS	POS	AMB	POS	NEG	NEG	NEG	NEG	POS	POS	POS	POS	NEG
MSSA1999	POS	NEG	NEG	POS	POS	NEG	NEG	POS	POS	AMB	POS	NEG	NEG	NEG	NEG	POS	POS	POS	POS	NEG
MSSA2003	POS	NEG	NEG	NEG	POS	NEG	NEG	POS	POS	AMB	POS	NEG	NEG	NEG	NEG	POS	POS	POS	POS	NEG
MSSA2006	POS	NEG	NEG	NEG	POS	NEG	NEG	POS	POS	NEG	POS	NEG	NEG	NEG	NEG	POS	POS	POS	POS	NEG
MSSA2008	POS	NEG	NEG	NEG	POS	NEG	NEG	POS	POS	AMB	POS	NEG	NEG	NEG	NEG	POS	POS	POS	POS	NEG
MSSA2011	POS	NEG	NEG	POS	POS	NEG	NEG	POS	POS	NEG	POS	NEG	NEG	NEG	NEG	POS	POS	POS	POS	NEG
MSSA2017	POS	NEG	NEG	NEG	POS	NEG	NEG	POS	POS	NEG	POS	NEG	NEG	NEG	NEG	POS	POS	POS	AMB	NEG
MSSA2022	POS	NEG	NEG	POS	POS	NEG	NEG	POS	POS	NEG	POS	NEG	NEG	NEG	NEG	POS	POS	POS	POS	NEG
MSSA2055	POS	NEG	NEG	NEG	POS	NEG	NEG	POS	POS	AMB	POS	NEG	NEG	NEG	NEG	POS	POS	POS	POS	NEG
MSSA2060	POS	NEG	NEG	NEG	POS	NEG	NEG	POS	POS	AMB	POS	NEG	NEG	NEG	NEG	POS	POS	POS	POS	NEG
MSSA2080	POS	NEG	NEG	NEG	POS	NEG	NEG	POS	POS	NEG	POS	NEG	NEG	NEG	NEG	POS	POS	POS	POS	NEG
MSSA2082	POS	NEG	NEG	NEG	POS	NEG	NEG	POS	POS	AMB	POS	NEG	NEG	NEG	NEG	POS	POS	POS	POS	NEG
MSSA2084	POS	NEG	NEG	NEG	POS	NEG	NEG	POS	POS	NEG	AMB	NEG	NEG	NEG	NEG	POS	POS	POS	POS	NEG
MSSA2087	POS	NEG	NEG	NEG	POS	NEG	NEG	POS	POS	NEG	POS	NEG	NEG	NEG	NEG	POS	POS	POS	POS	NEG
MSSA2094	POS	NEG	NEG	NEG	POS	NEG	NEG	POS	POS	AMB	POS	NEG	NEG	NEG	NEG	POS	POS	POS	POS	NEG
MSSA2097	POS	NEG	NEG	NEG	POS	NEG	NEG	POS	POS	AMB	POS	NEG	NEG	NEG	NEG	POS	POS	POS	POS	NEG
MSSA2110	POS	NEG	NEG	NEG	POS	NEG	NEG	POS	POS	AMB	POS	NEG	NEG	NEG	NEG	POS	POS	POS	POS	NEG
MSSA2113	POS	NEG	NEG	NEG	POS	NEG	NEG	POS	POS	AMB	POS	NEG	NEG	NEG	NEG	POS	POS	POS	POS	NEG
MSSA2132	POS	NEG	NEG	NEG	POS	NEG	NEG	POS	POS	AMB	POS	NEG	NEG	NEG	NEG	POS	POS	POS	POS	NEG
MSSA2134	POS	NEG	NEG	NEG	POS	NEG	NEG	POS	POS	NEG	POS	NEG	NEG	NEG	NEG	POS	POS	POS	POS	NEG
MSSA1746	NEG	NEG	NEG	NEG	NEG	NEG	NEG	POS	AMB	POS	POS	NEG	NEG	NEG	NEG	POS	POS	POS	POS	NEG
MSSA1757	POS	NEG	NEG	NEG	NEG	NEG	NEG	POS	AMB	POS	POS	NEG	NEG	NEG	NEG	POS	POS	POS	POS	NEG
MSSA1772	NEG	NEG	NEG	NEG	NEG	NEG	NEG	POS	NEG	POS	POS	NEG	NEG	NEG	NEG	POS	POS	POS	POS	NEG
MSSA1795	NEG	NEG	NEG	NEG	NEG	NEG	NEG	POS	AMB	POS	POS	NEG	NEG	NEG	NEG	POS	POS	POS	POS	NEG
MSSA1813	NEG	NEG	NEG	NEG	NEG	NEG	NEG	POS	AMB	POS	POS	NEG	NEG	NEG	NEG	POS	POS	POS	POS	NEG
MSSA1826	NEG	NEG	NEG	NEG	NEG	NEG	NEG	POS	AMB	POS	POS	NEG	NEG	NEG	NEG	POS	POS	POS	POS	NEG
MSSA1830	NEG	NEG	NEG	NEG	NEG	NEG	NEG	POS	AMB	POS	POS	NEG	NEG	NEG	NEG	POS	POS	POS	POS	NEG
MSSA1838	NEG	NEG	NEG	NEG	NEG	NEG	NEG	POS	AMB	POS	POS	NEG	NEG	NEG	NEG	POS	POS	POS	POS	NEG
MSSA1944	NEG	NEG	NEG	NEG	NEG	NEG	NEG	POS	NEG	POS	POS	NEG	NEG	NEG	NEG	POS	POS	POS	POS	NEG
MSSA1947	NEG	NEG	NEG	NEG	NEG	NEG	NEG	POS	AMB	POS	POS	NEG	NEG	NEG	NEG	POS	POS	POS	POS	NEG
MSSA1964	NEG	NEG	NEG	NEG	NEG	NEG	NEG	POS	AMB	POS	POS	NEG	NEG	NEG	NEG	POS	POS	POS	POS	NEG
MSSA1969	NEG	NEG	NEG	NEG	NEG	NEG	NEG	POS	AMB	POS	POS	NEG	NEG	NEG	NEG	POS	POS	POS	POS	NEG
MSSA2001	NEG	NEG	NEG	NEG	NEG	NEG	NEG	POS	AMB	POS	POS	NEG	NEG	NEG	NEG	POS	POS	POS	POS	NEG
MSSA2004	NEG	NEG	NEG	NEG	NEG	NEG	NEG	POS	AMB	POS	POS	NEG	NEG	NEG	NEG	POS	POS	POS	POS	NEG
MSSA2024	NEG	NEG	NEG	NEG	NEG	NEG	NEG	POS	NEG	POS	AMB	NEG	NEG	NEG	NEG	POS	POS	POS	POS	NEG
MSSA2025	NEG	NEG	NEG	NEG	NEG	NEG	NEG	POS	NEG	POS	POS	NEG	NEG	NEG	NEG	POS	POS	POS	POS	NEG
MSSA2026	NEG	NEG	NEG	NEG	NEG	NEG	NEG	POS	NEG	POS	POS	NEG	NEG	NEG	NEG	POS	POS	POS	POS	NEG
MSSA2032	NEG	NEG	NEG	NEG	NEG	NEG	NEG	POS	NEG	POS	POS	NEG	NEG	NEG	NEG	POS	POS	POS	POS	NEG
MSSA2043	NEG	NEG	NEG	NEG	NEG	NEG	NEG	POS	AMB	POS	POS	NEG	NEG	NEG	NEG	POS	POS	POS	POS	NEG
MSSA2061	NEG	NEG	NEG	NEG	NEG	NEG	NEG	POS	AMB	POS	POS	NEG	NEG	NEG	NEG	POS	POS	POS	POS	NEG
MSSA2085	NEG	NEG	NEG	NEG	NEG	NEG	NEG	POS	AMB	POS	POS	NEG	NEG	NEG	NEG	POS	POS	POS	POS	NEG
MSSA2098	NEG	NEG	NEG	NEG	NEG	NEG	NEG	POS	AMB	POS	POS	NEG	NEG	NEG	NEG	POS	POS	POS	POS	NEG
MSSA2100	NEG	NEG	NEG	NEG	NEG	NEG	NEG	POS	AMB	POS	POS	NEG	NEG	NEG	NEG	POS	POS	POS	POS	NEG
MSSA2111	NEG	NEG	NEG	NEG	NEG	NEG	NEG	POS	AMB	POS	POS	NEG	NEG	NEG	NEG	POS	POS	POS	POS	NEG
MSSA2178	NEG	NEG	NEG	NEG	NEG	NEG	NEG	POS	NEG	POS	POS	NEG	NEG	NEG	NEG	POS	POS	POS	POS	NEG
MSSA2202	NEG	NEG	NEG	NEG	NEG	NEG	NEG	POS	NEG	POS	POS	NEG	NEG	NEG	NEG	POS	POS	POS	POS	NEG
MSSA2205	NEG	NEG	NEG	NEG	NEG	NEG	NEG	POS	NEG	POS	POS	NEG	NEG	NEG	NEG	POS	POS	POS	POS	NEG
MRSA3796	NEG	NEG	NEG	NEG	NEG	NEG	NEG	POS	POS	AMB	POS	NEG	NEG	NEG	NEG	POS	POS	POS	POS	NEG
MRSA3805	NEG	POS	POS	NEG	NEG	NEG	NEG	POS	POS	AMB	POS	NEG	NEG	NEG	NEG	POS	POS	POS	POS	NEG
MRSA3965	NEG	POS	POS	NEG	NEG	NEG	NEG	POS	POS	AMB	POS	NEG	NEG	NEG	NEG	POS	POS	POS	POS	NEG
MSSA1761	NEG	NEG	NEG	NEG	NEG	NEG	NEG	POS	POS	AMB	POS	NEG	NEG	NEG	NEG	POS	POS	POS	POS	NEG
MSSA1764	NEG	NEG	NEG	POS	NEG	NEG	NEG	POS	POS	NEG	POS	NEG	NEG	NEG	NEG	POS	POS	POS	POS	NEG
MSSA1765	NEG	NEG	NEG	NEG	NEG	NEG	NEG	POS	POS	NEG	POS	NEG	NEG	NEG	NEG	POS	POS	POS	POS	NEG
MSSA1784	NEG	NEG	NEG	NEG	NEG	NEG	NEG	POS	POS	AMB	POS	NEG	NEG	NEG	NEG	POS	POS	POS	POS	NEG
MSSA1806	NEG	NEG	NEG	NEG	NEG	NEG	NEG	POS	POS	NEG	POS	NEG	NEG	NEG	NEG	POS	POS	POS	POS	NEG
MSSA1807	NEG	NEG	NEG	NEG	NEG	NEG	NEG	POS	POS	AMB	POS	NEG	NEG	NEG	NEG	POS	POS	POS	POS	NEG
MSSA1888	NEG	POS	POS	POS	NEG	NEG	NEG	POS	POS	NEG	POS	NEG	NEG	NEG	NEG	POS	POS	POS	POS	NEG
MSSA1894	NEG	NEG	NEG	NEG	NEG	NEG	NEG	POS	POS	NEG	POS	NEG	NEG	NEG	NEG	POS	POS	POS	POS	NEG
MSSA1922	NEG	NEG	NEG	POS	NEG	NEG	NEG	POS	POS	AMB	POS	NEG	NEG	NEG	NEG	POS	POS	POS	POS	NEG
MSSA1950	NEG	NEG	NEG	POS	NEG	NEG	NEG	POS	POS	AMB	POS	NEG	NEG	NEG	NEG	POS	POS	POS	POS	NEG
MSSA1954	NEG	NEG	NEG	POS	NEG	NEG	NEG	POS	POS	AMB	POS	NEG	NEG	NEG	NEG	POS	POS	POS	POS	NEG
MSSA1966	NEG	POS	POS	POS	NEG	NEG	NEG	POS	POS	AMB	POS	NEG	NEG	NEG	NEG	POS	POS	POS	POS	NEG
MSSA1972	NEG	NEG	NEG	NEG	NEG	NEG	NEG	POS	POS	NEG	POS	NEG	NEG	NEG	NEG	POS	POS	POS	POS	NEG
MSSA2010	NEG	NEG	NEG	POS	NEG	NEG	NEG	POS	POS	NEG	POS	NEG	NEG	NEG	NEG	POS	POS	POS	POS	NEG
MSSA2020	NEG	NEG	NEG	NEG	NEG	NEG	NEG	POS	POS	NEG	POS	NEG	NEG	NEG	NEG	POS	POS	POS	POS	NEG
MSSA2028	NEG	POS	POS	NEG	NEG	NEG	NEG	POS	POS	AMB	POS	NEG	NEG	NEG	NEG	POS	POS	POS	POS	NEG
MSSA2088	NEG	NEG	NEG	NEG	NEG	NEG	NEG	POS	POS	AMB	POS	NEG	NEG	NEG	NEG	POS	POS	POS	POS	NEG
MSSA2187	NEG	NEG	NEG	NEG	NEG	NEG	NEG	POS	POS	AMB	POS	NEG	NEG	NEG	NEG	POS	POS	POS	POS	NEG
MSSA2204	NEG	NEG	NEG	POS	NEG	NEG	NEG	POS	POS	AMB	POS	NEG	NEG	NEG	NEG	POS	POS	POS	POS	NEG
MSSA2210	NEG	NEG	NEG	NEG	NEG	NEG	NEG	POS	POS	AMB	POS	NEG	NEG	NEG	NEG	POS	POS	POS	POS	NEG
MSSA2223	NEG	NEG	NEG	POS	NEG	NEG	NEG	POS	POS	AMB	POS	NEG	NEG	NEG	NEG	POS	POS	POS	POS	NEG
MSSA1763	POS	NEG	NEG	NEG	POS	NEG	NEG	POS	POS	NEG	POS	NEG	NEG	NEG	NEG	NEG	NEG	POS	POS	NEG
MSSA1777	POS	NEG	NEG	NEG	POS	NEG	NEG	POS	POS	AMB	POS	NEG	NEG	NEG	NEG	NEG	NEG	POS	POS	NEG
MSSA2009	POS	NEG	NEG	NEG	POS	NEG	NEG	POS	POS	NEG	POS	NEG	NEG	NEG	NEG	NEG	NEG	POS	POS	NEG
MSSA2047	POS	NEG	NEG	NEG	POS	NEG	NEG	POS	POS	AMB	POS	NEG	NEG	NEG	NEG	NEG	NEG	POS	POS	NEG
MSSA2079	POS	NEG	NEG	NEG	POS	NEG	NEG	POS	POS	NEG	POS	NEG	NEG	NEG	NEG	NEG	NEG	POS	POS	NEG
MSSA2101	POS	NEG	NEG	NEG	POS	NEG	NEG	POS	POS	AMB	POS	NEG	NEG	NEG	NEG	NEG	NEG	POS	POS	NEG
MSSA1821	NEG	NEG	NEG	NEG	NEG	POS	POS	POS	POS	AMB	POS	NEG	NEG	NEG	NEG	POS	POS	POS	POS	NEG
MSSA1898	NEG	NEG	NEG	NEG	NEG	POS	POS	POS	POS	AMB	POS	NEG	NEG	NEG	NEG	POS	POS	POS	POS	NEG
MSSA1973	NEG	NEG	NEG	NEG	NEG	POS	POS	POS	POS	AMB	POS	NEG	NEG	NEG	NEG	POS	POS	POS	POS	NEG
MSSA2071	NEG	NEG	NEG	NEG	NEG	POS	POS	POS	POS	AMB	POS	NEG	NEG	NEG	NEG	POS	POS	POS	POS	NEG
MSSA2083	NEG	NEG	NEG	NEG	NEG	POS	POS	POS	POS	AMB	POS	NEG	NEG	NEG	NEG	POS	POS	POS	POS	NEG
MSSA2106	NEG	NEG	NEG	NEG	NEG	POS	POS	POS	POS	AMB	POS	NEG	NEG	NEG	NEG	POS	POS	POS	POS	NEG
MSSA2112	NEG	NEG	NEG	NEG	NEG	POS	POS	POS	POS	AMB	POS	NEG	NEG	NEG	NEG	POS	POS	POS	POS	NEG
MSSA2115	NEG	NEG	NEG	NEG	NEG	POS	POS	POS	POS	AMB	POS	NEG	NEG	NEG	AMB	POS	POS	POS	POS	NEG
MSSA2195	NEG	NEG	NEG	NEG	NEG	POS	POS	POS	POS	AMB	POS	NEG	NEG	NEG	NEG	POS	POS	POS	POS	NEG
MSSA2207	NEG	NEG	NEG	NEG	NEG	POS	POS	POS	POS	AMB	POS	NEG	NEG	NEG	NEG	POS	POS	POS	POS	NEG
MSSA1758	NEG	NEG	NEG	NEG	NEG	NEG	NEG	POS	POS	AMB	POS	NEG	NEG	NEG	NEG	POS	AMB	POS	POS	NEG
MSSA1762	NEG	NEG	NEG	NEG	NEG	NEG	NEG	POS	POS	AMB	POS	NEG	NEG	NEG	NEG	POS	POS	POS	POS	NEG
MSSA1769	NEG	NEG	NEG	NEG	NEG	NEG	NEG	POS	POS	AMB	POS	NEG	NEG	NEG	NEG	POS	POS	POS	POS	NEG
MSSA1771	NEG	NEG	NEG	NEG	NEG	NEG	NEG	POS	POS	AMB	POS	NEG	NEG	NEG	NEG	POS	AMB	POS	POS	NEG
MSSA1789	NEG	NEG	NEG	NEG	NEG	NEG	NEG	POS	POS	NEG	POS	NEG	NEG	NEG	NEG	POS	AMB	POS	POS	NEG
MSSA1800	NEG	NEG	NEG	NEG	NEG	NEG	NEG	POS	POS	AMB	POS	NEG	NEG	NEG	NEG	POS	POS	POS	POS	NEG
MSSA1833	NEG	NEG	NEG	NEG	NEG	NEG	NEG	POS	POS	AMB	POS	NEG	NEG	NEG	NEG	POS	POS	POS	POS	NEG
MSSA1890	NEG	NEG	NEG	NEG	NEG	NEG	NEG	POS	POS	AMB	POS	NEG	NEG	NEG	NEG	POS	AMB	POS	POS	NEG
MSSA1903	NEG	NEG	NEG	NEG	NEG	NEG	NEG	POS	POS	AMB	POS	NEG	NEG	NEG	NEG	POS	POS	POS	POS	NEG
MSSA1940	NEG	NEG	NEG	NEG	NEG	NEG	NEG	POS	POS	AMB	POS	NEG	NEG	NEG	NEG	POS	POS	POS	POS	NEG
MSSA1962	NEG	NEG	NEG	NEG	NEG	NEG	NEG	POS	POS	NEG	POS	NEG	NEG	NEG	NEG	POS	POS	POS	POS	NEG
MSSA1963	NEG	NEG	NEG	NEG	NEG	NEG	NEG	POS	POS	AMB	POS	NEG	NEG	NEG	NEG	POS	POS	POS	POS	NEG
MSSA2016	NEG	NEG	NEG	NEG	NEG	NEG	NEG	POS	POS	NEG	POS	NEG	NEG	NEG	NEG	POS	POS	POS	POS	NEG
MSSA2018	NEG	NEG	NEG	NEG	NEG	NEG	NEG	POS	POS	NEG	POS	NEG	NEG	NEG	NEG	POS	POS	POS	POS	NEG
MSSA2019	NEG	NEG	NEG	NEG	NEG	NEG	NEG	POS	POS	NEG	POS	NEG	NEG	NEG	NEG	POS	POS	POS	POS	NEG
MSSA2029	NEG	NEG	NEG	NEG	NEG	NEG	NEG	POS	POS	AMB	POS	NEG	NEG	NEG	NEG	POS	POS	POS	POS	NEG
MSSA2045	NEG	NEG	NEG	NEG	NEG	NEG	NEG	POS	POS	AMB	POS	NEG	NEG	NEG	NEG	POS	POS	POS	POS	NEG
MSSA2048	NEG	NEG	NEG	NEG	NEG	NEG	NEG	POS	POS	AMB	POS	NEG	NEG	NEG	NEG	POS	POS	POS	POS	NEG
MSSA2049	NEG	NEG	NEG	NEG	NEG	NEG	NEG	POS	POS	AMB	POS	NEG	NEG	NEG	NEG	POS	POS	POS	POS	NEG
MSSA2050	NEG	NEG	NEG	NEG	NEG	NEG	NEG	POS	POS	AMB	POS	NEG	NEG	NEG	NEG	POS	POS	POS	POS	NEG
MSSA2099	NEG	NEG	NEG	NEG	NEG	NEG	NEG	POS	POS	NEG	POS	NEG	NEG	NEG	NEG	POS	NEG	POS	POS	NEG
MSSA2109	NEG	NEG	NEG	NEG	NEG	NEG	NEG	POS	POS	NEG	POS	NEG	NEG	NEG	NEG	POS	NEG	POS	POS	NEG
MSSA2130	NEG	NEG	NEG	NEG	NEG	NEG	NEG	POS	POS	AMB	POS	NEG	NEG	NEG	NEG	POS	POS	POS	POS	NEG
MSSA2133	NEG	NEG	NEG	NEG	NEG	NEG	NEG	POS	POS	AMB	POS	NEG	NEG	NEG	NEG	POS	POS	POS	POS	NEG
MSSA2139	NEG	NEG	NEG	NEG	NEG	NEG	NEG	POS	POS	AMB	POS	NEG	NEG	NEG	NEG	POS	POS	POS	POS	NEG
MSSA2141	NEG	NEG	NEG	NEG	NEG	NEG	NEG	POS	POS	AMB	POS	NEG	NEG	NEG	NEG	POS	POS	POS	POS	NEG
MSSA2208	NEG	NEG	NEG	NEG	NEG	NEG	NEG	POS	POS	AMB	POS	NEG	NEG	NEG	NEG	POS	POS	POS	POS	NEG
MSSA2209	NEG	NEG	NEG	NEG	NEG	NEG	NEG	POS	POS	AMB	POS	NEG	NEG	NEG	NEG	POS	POS	POS	POS	NEG
MSSA1893	POS	NEG	NEG	NEG	POS	NEG	NEG	POS	POS	NEG	POS	NEG	NEG	NEG	NEG	NEG	POS	POS	POS	NEG
MSSA2105	POS	NEG	NEG	NEG	POS	NEG	NEG	POS	POS	NEG	POS	NEG	NEG	NEG	NEG	NEG	POS	POS	POS	NEG
MRSA3836	POS	NEG	NEG	NEG	POS	NEG	NEG	POS	AMB	POS	AMB	NEG	NEG	NEG	NEG	NEG	NEG	POS	POS	NEG
MRSA3840	POS	NEG	NEG	NEG	POS	NEG	NEG	POS	AMB	POS	AMB	NEG	NEG	NEG	NEG	NEG	NEG	POS	POS	NEG
MRSA4188	POS	NEG	NEG	NEG	POS	NEG	NEG	POS	AMB	POS	POS	NEG	NEG	NEG	NEG	AMB	NEG	POS	POS	NEG
MSSA1814	POS	NEG	NEG	NEG	POS	NEG	NEG	POS	AMB	POS	AMB	NEG	NEG	NEG	NEG	NEG	NEG	POS	POS	NEG
MSSA1816	POS	NEG	NEG	NEG	POS	NEG	NEG	NEG	NEG	POS	NEG	NEG	NEG	NEG	NEG	NEG	NEG	POS	POS	NEG
MSSA1825	POS	NEG	NEG	NEG	POS	NEG	NEG	POS	AMB	POS	AMB	NEG	NEG	NEG	NEG	NEG	NEG	POS	POS	NEG
MSSA1834	POS	NEG	NEG	NEG	POS	NEG	NEG	NEG	NEG	POS	NEG	NEG	NEG	NEG	NEG	NEG	NEG	POS	POS	NEG
MSSA1938	POS	NEG	NEG	NEG	POS	NEG	NEG	POS	AMB	POS	AMB	NEG	NEG	NEG	NEG	NEG	NEG	POS	POS	NEG
MSSA1948	POS	NEG	NEG	NEG	POS	NEG	NEG	POS	NEG	POS	POS	NEG	NEG	NEG	NEG	NEG	NEG	POS	POS	NEG
MSSA1951	POS	NEG	NEG	NEG	POS	NEG	NEG	AMB	NEG	POS	NEG	NEG	NEG	NEG	NEG	NEG	NEG	POS	POS	NEG
MSSA2005	POS	NEG	NEG	NEG	POS	NEG	NEG	POS	AMB	POS	AMB	NEG	NEG	NEG	NEG	NEG	NEG	POS	POS	NEG
MSSA2051	POS	NEG	NEG	NEG	POS	NEG	NEG	POS	AMB	POS	POS	NEG	NEG	NEG	NEG	NEG	NEG	POS	POS	NEG
MSSA2056	POS	NEG	NEG	NEG	POS	NEG	NEG	POS	AMB	POS	POS	NEG	NEG	NEG	NEG	NEG	NEG	POS	POS	NEG
MSSA2066	POS	NEG	NEG	NEG	POS	NEG	NEG	POS	AMB	POS	POS	NEG	NEG	NEG	NEG	NEG	NEG	POS	POS	NEG
MSSA2067	POS	NEG	NEG	NEG	POS	NEG	NEG	AMB	NEG	POS	AMB	NEG	NEG	NEG	NEG	NEG	NEG	POS	POS	NEG
MSSA2086	POS	NEG	NEG	NEG	POS	NEG	NEG	AMB	NEG	POS	AMB	NEG	NEG	NEG	NEG	NEG	NEG	POS	POS	NEG
MSSA2089	POS	NEG	NEG	NEG	POS	NEG	NEG	AMB	NEG	POS	NEG	NEG	NEG	NEG	NEG	NEG	NEG	POS	POS	NEG
MSSA2103	POS	NEG	NEG	NEG	POS	NEG	NEG	AMB	NEG	POS	AMB	NEG	NEG	NEG	NEG	NEG	NEG	POS	POS	NEG
MSSA2173	POS	NEG	NEG	NEG	POS	NEG	NEG	POS	AMB	POS	POS	NEG	NEG	NEG	NEG	NEG	NEG	POS	POS	NEG
MSSA2183	POS	NEG	NEG	NEG	POS	NEG	NEG	POS	AMB	POS	POS	NEG	NEG	NEG	NEG	NEG	NEG	POS	POS	NEG
MSSA2196	POS	NEG	NEG	NEG	POS	NEG	NEG	POS	AMB	POS	POS	NEG	NEG	NEG	NEG	NEG	NEG	POS	POS	NEG
MSSA2219	POS	NEG	NEG	NEG	POS	NEG	NEG	POS	NEG	POS	POS	NEG	NEG	NEG	NEG	NEG	NEG	POS	POS	NEG
MSSA1753	POS	NEG	NEG	NEG	POS	NEG	NEG	POS	POS	NEG	POS	NEG	NEG	NEG	NEG	POS	POS	POS	POS	NEG
MSSA1773	POS	NEG	NEG	NEG	POS	NEG	NEG	POS	POS	AMB	POS	NEG	NEG	NEG	NEG	POS	POS	POS	POS	NEG
MSSA1811	POS	NEG	NEG	NEG	POS	NEG	NEG	POS	POS	AMB	POS	NEG	NEG	NEG	NEG	POS	POS	POS	POS	NEG
MSSA1897	POS	NEG	NEG	NEG	POS	NEG	NEG	POS	POS	AMB	POS	NEG	NEG	NEG	NEG	POS	POS	POS	POS	NEG
MSSA1931	POS	NEG	NEG	NEG	POS	NEG	NEG	POS	POS	AMB	POS	NEG	NEG	NEG	NEG	POS	POS	POS	POS	NEG
MSSA2114	POS	NEG	NEG	NEG	POS	NEG	NEG	POS	POS	AMB	POS	NEG	NEG	NEG	NEG	POS	POS	POS	POS	NEG
MSSA1745	POS	NEG	NEG	NEG	POS	NEG	NEG	POS	POS	AMB	POS	NEG	NEG	NEG	NEG	NEG	NEG	POS	NEG	POS
MSSA1747	POS	NEG	NEG	NEG	POS	NEG	NEG	POS	POS	AMB	POS	NEG	NEG	NEG	NEG	NEG	NEG	POS	NEG	POS
MSSA1749	POS	NEG	NEG	NEG	POS	NEG	NEG	POS	POS	AMB	POS	NEG	NEG	NEG	NEG	NEG	NEG	POS	NEG	POS
MSSA1768	POS	NEG	NEG	NEG	POS	NEG	NEG	POS	POS	NEG	POS	NEG	NEG	NEG	NEG	NEG	NEG	POS	NEG	POS
MSSA1774	POS	NEG	NEG	NEG	POS	NEG	NEG	POS	POS	NEG	POS	NEG	NEG	NEG	NEG	NEG	NEG	POS	NEG	POS
MSSA1775	POS	NEG	NEG	NEG	POS	NEG	NEG	POS	POS	AMB	POS	NEG	NEG	NEG	NEG	NEG	NEG	POS	NEG	POS
MSSA1776	POS	NEG	NEG	NEG	POS	NEG	NEG	POS	POS	AMB	POS	NEG	NEG	NEG	NEG	NEG	NEG	POS	NEG	POS
MSSA1779	POS	NEG	NEG	NEG	POS	NEG	NEG	POS	POS	NEG	AMB	NEG	NEG	NEG	NEG	NEG	NEG	POS	NEG	POS
MSSA1780	POS	NEG	NEG	NEG	POS	NEG	NEG	POS	POS	AMB	POS	NEG	NEG	NEG	NEG	NEG	NEG	POS	NEG	POS

	VIRULENCE: ENTEROTOXINS, continued							VIRULENCE: HLG AND LEUKOCIDIN												
Strain	seo = entO	seq = entQ (probe 1)	seq = entQ (probe 2)	ser = entR	seu = entU	ORF CM14 (probe 1)	ORF CM14 (probe 2)	lukF	lukS	lukS (ST22+45)	hlgA	lukF-PV	lukF-PV (P83)	lukS-PV	lukM	lukD	lukE	lukX	lukY (var1)	lukY (var2)
MSSA1781	POS	NEG	NEG	NEG	POS	NEG	NEG	POS	POS	AMB	POS	NEG	NEG	NEG	NEG	NEG	NEG	POS	NEG	POS
MSSA1786	POS	NEG	NEG	NEG	POS	NEG	NEG	POS	POS	AMB	POS	NEG	NEG	NEG	NEG	NEG	NEG	POS	NEG	POS
MSSA1793	POS	NEG	NEG	NEG	POS	NEG	NEG	POS	POS	NEG	POS	NEG	NEG	NEG	NEG	NEG	NEG	POS	NEG	POS
MSSA1803	POS	NEG	NEG	NEG	POS	NEG	NEG	POS	POS	AMB	POS	NEG	NEG	NEG	NEG	NEG	NEG	POS	NEG	POS
MSSA1836	POS	NEG	NEG	NEG	POS	NEG	NEG	POS	POS	AMB	POS	NEG	NEG	NEG	NEG	NEG	NEG	POS	NEG	POS
MSSA1839	POS	NEG	NEG	NEG	POS	NEG	NEG	POS	POS	AMB	POS	NEG	NEG	NEG	NEG	NEG	NEG	POS	NEG	POS
MSSA1900	POS	NEG	NEG	NEG	POS	NEG	NEG	POS	POS	AMB	POS	NEG	NEG	NEG	NEG	NEG	NEG	NEG	NEG	NEG
MSSA1910	POS	NEG	NEG	NEG	POS	NEG	NEG	POS	POS	AMB	POS	NEG	NEG	NEG	NEG	NEG	NEG	POS	NEG	POS
MSSA1918	POS	NEG	NEG	NEG	POS	NEG	NEG	POS	POS	NEG	POS	NEG	NEG	NEG	NEG	NEG	NEG	POS	NEG	POS
MSSA1921	POS	NEG	NEG	NEG	POS	NEG	NEG	POS	POS	AMB	POS	NEG	NEG	NEG	NEG	NEG	NEG	POS	NEG	POS
MSSA1923	POS	NEG	NEG	NEG	POS	NEG	NEG	POS	POS	AMB	POS	NEG	NEG	NEG	NEG	NEG	NEG	POS	NEG	POS
MSSA1981	POS	NEG	NEG	NEG	POS	NEG	NEG	POS	POS	AMB	POS	NEG	NEG	NEG	NEG	NEG	NEG	POS	NEG	POS
MSSA1998	POS	NEG	NEG	NEG	POS	NEG	NEG	POS	POS	AMB	POS	NEG	NEG	NEG	NEG	NEG	NEG	POS	NEG	POS
MSSA2000	POS	NEG	NEG	NEG	POS	NEG	NEG	POS	POS	NEG	POS	NEG	NEG	NEG	NEG	NEG	NEG	POS	NEG	POS
MSSA2007	POS	NEG	NEG	NEG	POS	NEG	NEG	POS	POS	AMB	POS	NEG	NEG	NEG	NEG	NEG	NEG	POS	NEG	POS
MSSA2012	POS	NEG	NEG	NEG	POS	NEG	NEG	POS	POS	AMB	POS	NEG	NEG	NEG	NEG	NEG	NEG	POS	NEG	POS
MSSA2015	POS	NEG	NEG	NEG	POS	NEG	NEG	POS	POS	NEG	AMB	NEG	NEG	NEG	NEG	NEG	NEG	POS	NEG	POS
MSSA2021	POS	NEG	NEG	NEG	POS	NEG	NEG	POS	POS	NEG	AMB	NEG	NEG	NEG	NEG	NEG	NEG	POS	NEG	POS
MSSA2027	POS	NEG	NEG	NEG	POS	NEG	NEG	POS	POS	NEG	POS	NEG	NEG	NEG	NEG	NEG	NEG	POS	NEG	POS
MSSA2063	POS	NEG	NEG	NEG	POS	NEG	NEG	POS	POS	NEG	POS	NEG	NEG	NEG	NEG	NEG	NEG	POS	NEG	POS
MSSA2064	POS	NEG	NEG	NEG	POS	NEG	NEG	POS	POS	AMB	POS	NEG	NEG	NEG	NEG	NEG	NEG	POS	NEG	POS
MSSA2065	POS	NEG	NEG	NEG	POS	NEG	NEG	POS	POS	AMB	POS	NEG	NEG	NEG	NEG	NEG	NEG	POS	NEG	POS
MSSA2075	POS	NEG	NEG	NEG	POS	NEG	NEG	POS	POS	AMB	POS	NEG	NEG	NEG	NEG	NEG	NEG	POS	NEG	POS
MSSA2093	POS	NEG	NEG	NEG	POS	NEG	NEG	POS	POS	AMB	POS	NEG	NEG	NEG	NEG	NEG	NEG	POS	NEG	POS
MSSA2102	POS	NEG	NEG	NEG	POS	NEG	NEG	POS	POS	AMB	POS	NEG	NEG	NEG	NEG	NEG	NEG	POS	NEG	POS
MSSA2138	POS	NEG	NEG	NEG	POS	NEG	NEG	POS	POS	AMB	POS	NEG	NEG	NEG	NEG	NEG	NEG	POS	NEG	POS
MSSA2168	POS	NEG	NEG	NEG	POS	NEG	NEG	POS	POS	AMB	POS	NEG	NEG	NEG	NEG	NEG	NEG	POS	NEG	POS
MSSA2181	POS	NEG	NEG	NEG	POS	NEG	NEG	POS	POS	AMB	POS	NEG	NEG	NEG	NEG	NEG	NEG	POS	NEG	POS
MSSA2189	POS	NEG	NEG	NEG	POS	NEG	NEG	POS	POS	AMB	POS	NEG	NEG	NEG	NEG	NEG	NEG	POS	NEG	POS
MSSA2214	POS	NEG	NEG	NEG	POS	NEG	NEG	POS	POS	AMB	POS	NEG	NEG	NEG	NEG	NEG	NEG	POS	NEG	POS
MRSA3803	POS	NEG	NEG	NEG	POS	NEG	NEG	POS	AMB	POS	POS	NEG	NEG	NEG	NEG	NEG	NEG	POS	NEG	POS
MSSA1750	POS	NEG	NEG	NEG	POS	NEG	NEG	POS	AMB	POS	POS	NEG	NEG	NEG	NEG	NEG	NEG	POS	NEG	POS
MSSA1756	POS	NEG	NEG	NEG	POS	NEG	NEG	POS	AMB	POS	POS	NEG	NEG	NEG	NEG	NEG	NEG	POS	NEG	POS
MSSA1759	POS	NEG	NEG	NEG	POS	NEG	NEG	POS	AMB	POS	POS	NEG	NEG	NEG	NEG	NEG	NEG	POS	NEG	POS
MSSA1767	POS	NEG	NEG	NEG	POS	NEG	NEG	POS	AMB	POS	POS	NEG	NEG	NEG	NEG	NEG	NEG	POS	NEG	POS
MSSA1787	POS	NEG	NEG	NEG	POS	NEG	NEG	POS	AMB	POS	POS	NEG	NEG	NEG	NEG	NEG	NEG	POS	NEG	POS
MSSA1790	POS	NEG	NEG	NEG	POS	NEG	NEG	POS	AMB	POS	POS	NEG	NEG	NEG	NEG	NEG	NEG	POS	NEG	POS
MSSA1794	POS	NEG	NEG	NEG	POS	NEG	NEG	POS	NEG	POS	POS	NEG	NEG	NEG	NEG	NEG	NEG	POS	NEG	POS
MSSA1808	POS	NEG	NEG	NEG	POS	NEG	NEG	POS	AMB	POS	POS	NEG	NEG	NEG	NEG	NEG	NEG	POS	NEG	POS
MSSA1812	POS	NEG	NEG	NEG	POS	NEG	NEG	POS	AMB	POS	POS	NEG	NEG	NEG	NEG	NEG	NEG	POS	NEG	POS
MSSA1819	POS	NEG	NEG	NEG	POS	NEG	NEG	POS	AMB	POS	POS	NEG	NEG	NEG	NEG	NEG	NEG	POS	NEG	POS
MSSA1824	POS	NEG	NEG	NEG	POS	NEG	NEG	POS	AMB	POS	POS	NEG	AMB	NEG	NEG	NEG	NEG	POS	NEG	POS
MSSA1827	POS	NEG	NEG	NEG	POS	NEG	NEG	POS	AMB	POS	POS	NEG	NEG	NEG	NEG	NEG	NEG	POS	NEG	POS
MSSA1889	POS	NEG	NEG	NEG	POS	NEG	NEG	POS	AMB	POS	POS	NEG	NEG	NEG	NEG	NEG	NEG	POS	NEG	POS
MSSA1895	POS	NEG	NEG	NEG	POS	NEG	NEG	POS	NEG	POS	POS	NEG	NEG	NEG	NEG	NEG	NEG	POS	NEG	POS
MSSA1904	POS	NEG	NEG	NEG	POS	NEG	NEG	POS	AMB	POS	POS	NEG	NEG	NEG	NEG	NEG	NEG	POS	NEG	POS
MSSA1905	POS	NEG	NEG	NEG	POS	NEG	NEG	POS	NEG	POS	POS	NEG	NEG	NEG	NEG	NEG	NEG	POS	NEG	POS
MSSA1907	POS	NEG	NEG	NEG	POS	NEG	NEG	POS	NEG	POS	POS	NEG	NEG	NEG	NEG	NEG	NEG	POS	NEG	POS
MSSA1919	POS	NEG	NEG	NEG	POS	NEG	NEG	POS	AMB	POS	POS	NEG	NEG	NEG	NEG	NEG	NEG	POS	NEG	POS
MSSA1920	POS	NEG	NEG	NEG	POS	NEG	NEG	POS	AMB	POS	POS	NEG	NEG	NEG	NEG	NEG	NEG	POS	NEG	POS
MSSA1929	POS	NEG	NEG	NEG	POS	NEG	NEG	POS	AMB	POS	POS	NEG	NEG	NEG	NEG	NEG	NEG	POS	NEG	POS
MSSA1930	POS	NEG	NEG	NEG	POS	NEG	NEG	POS	NEG	POS	POS	NEG	NEG	NEG	NEG	NEG	NEG	POS	NEG	POS
MSSA1934	POS	NEG	NEG	NEG	POS	NEG	NEG	POS	NEG	NEG	POS	NEG	NEG	NEG	NEG	NEG	NEG	POS	NEG	POS
MSSA1946	POS	NEG	NEG	NEG	POS	NEG	NEG	POS	NEG	POS	POS	NEG	NEG	NEG	NEG	NEG	NEG	POS	NEG	POS
MSSA1971	POS	NEG	NEG	NEG	POS	NEG	NEG	POS	AMB	POS	POS	NEG	NEG	NEG	NEG	NEG	NEG	POS	NEG	POS
MSSA1980	POS	NEG	NEG	NEG	POS	NEG	NEG	POS	NEG	POS	POS	NEG	NEG	NEG	NEG	NEG	NEG	POS	NEG	POS
MSSA1982	POS	NEG	NEG	NEG	POS	NEG	NEG	POS	AMB	POS	POS	NEG	NEG	NEG	NEG	NEG	NEG	POS	NEG	POS
MSSA1996	POS	NEG	NEG	NEG	POS	NEG	NEG	POS	AMB	POS	POS	NEG	NEG	NEG	NEG	NEG	NEG	POS	NEG	POS
MSSA2002	POS	NEG	NEG	NEG	POS	NEG	NEG	POS	AMB	POS	POS	NEG	NEG	NEG	NEG	NEG	NEG	POS	NEG	POS
MSSA2014	POS	NEG	NEG	NEG	POS	NEG	NEG	POS	AMB	POS	POS	NEG	NEG	NEG	NEG	NEG	NEG	POS	NEG	POS
MSSA2053	POS	NEG	NEG	NEG	POS	NEG	NEG	POS	AMB	POS	POS	NEG	NEG	NEG	NEG	NEG	NEG	POS	NEG	POS
MSSA2054	POS	NEG	NEG	NEG	POS	NEG	NEG	POS	NEG	POS	POS	NEG	NEG	NEG	NEG	NEG	NEG	POS	NEG	POS
MSSA2058	POS	NEG	NEG	NEG	POS	NEG	NEG	POS	NEG	POS	POS	NEG	NEG	NEG	NEG	NEG	NEG	POS	NEG	POS
MSSA2068	POS	NEG	NEG	NEG	POS	NEG	NEG	POS	AMB	POS	POS	NEG	NEG	NEG	NEG	NEG	NEG	POS	NEG	POS
MSSA2070	POS	NEG	NEG	NEG	POS	NEG	NEG	POS	AMB	POS	POS	NEG	NEG	NEG	NEG	NEG	NEG	POS	NEG	POS
MSSA2072	POS	NEG	NEG	NEG	POS	NEG	NEG	POS	AMB	POS	POS	NEG	NEG	NEG	NEG	NEG	NEG	POS	NEG	POS
MSSA2073	POS	NEG	NEG	NEG	POS	NEG	NEG	POS	NEG	POS	POS	NEG	NEG	NEG	NEG	NEG	NEG	POS	NEG	POS
MSSA2074	POS	NEG	NEG	NEG	POS	NEG	NEG	POS	AMB	POS	POS	NEG	NEG	NEG	NEG	NEG	NEG	POS	NEG	POS
MSSA2107	POS	NEG	NEG	NEG	POS	NEG	NEG	POS	AMB	POS	POS	NEG	NEG	NEG	NEG	NEG	NEG	POS	NEG	POS
MSSA2108	POS	NEG	NEG	NEG	POS	NEG	NEG	POS	NEG	POS	POS	NEG	NEG	NEG	NEG	NEG	NEG	POS	NEG	POS
MSSA2129	POS	NEG	NEG	NEG	POS	NEG	NEG	POS	AMB	POS	POS	NEG	NEG	NEG	NEG	NEG	NEG	POS	NEG	POS
MSSA2135	POS	NEG	NEG	NEG	POS	NEG	NEG	POS	AMB	POS	POS	NEG	NEG	NEG	NEG	NEG	NEG	POS	NEG	POS
MSSA2137	POS	NEG	NEG	NEG	POS	NEG	NEG	POS	AMB	POS	POS	NEG	NEG	NEG	NEG	NEG	NEG	POS	NEG	POS
MSSA2140	POS	NEG	NEG	NEG	POS	NEG	NEG	POS	AMB	POS	POS	NEG	NEG	NEG	NEG	NEG	NEG	POS	NEG	POS
MSSA2182	POS	NEG	NEG	NEG	POS	NEG	NEG	POS	NEG	POS	POS	NEG	NEG	NEG	NEG	NEG	NEG	POS	NEG	POS
MSSA2188	POS	NEG	NEG	NEG	POS	NEG	NEG	POS	NEG	POS	POS	NEG	NEG	NEG	NEG	NEG	NEG	POS	NEG	POS
MSSA1899	NEG	POS	POS	NEG	NEG	NEG	NEG	POS	POS	AMB	POS	NEG	NEG	NEG	NEG	NEG	NEG	POS	POS	NEG
MSSA1915	NEG	POS	POS	NEG	NEG	NEG	NEG	POS	POS	AMB	POS	NEG	NEG	NEG	NEG	NEG	NEG	POS	POS	NEG
MSSA1949	NEG	POS	POS	NEG	NEG	NEG	NEG	POS	POS	AMB	POS	NEG	NEG	NEG	NEG	NEG	NEG	POS	POS	NEG
MSSA2042	NEG	POS	POS	NEG	NEG	NEG	NEG	POS	POS	NEG	POS	NEG	NEG	NEG	NEG	NEG	NEG	POS	POS	NEG
MSSA2077	NEG	NEG	NEG	NEG	NEG	NEG	NEG	POS	POS	NEG	POS	NEG	NEG	NEG	NEG	NEG	NEG	POS	POS	NEG
MSSA2095	NEG	POS	POS	NEG	NEG	NEG	NEG	POS	POS	AMB	POS	NEG	NEG	NEG	NEG	NEG	NEG	POS	POS	NEG
MSSA1997	NEG	NEG	NEG	NEG	NEG	NEG	NEG	POS	POS	NEG	POS	NEG	NEG	NEG	NEG	POS	POS	POS	POS	NEG
MSSA1810	NEG	NEG	NEG	NEG	NEG	NEG	NEG	POS	POS	NEG	POS	NEG	NEG	NEG	NEG	POS	NEG	POS	POS	NEG
MSSA1832	NEG	NEG	NEG	NEG	NEG	NEG	NEG	POS	POS	AMB	POS	NEG	NEG	NEG	NEG	POS	POS	POS	POS	NEG
MSSA1891	NEG	NEG	NEG	NEG	NEG	NEG	NEG	POS	POS	AMB	POS	NEG	NEG	NEG	NEG	POS	POS	POS	POS	NEG
MSSA1892	NEG	NEG	NEG	NEG	NEG	NEG	NEG	POS	POS	AMB	POS	NEG	NEG	NEG	NEG	POS	POS	POS	POS	NEG
MSSA1932	NEG	NEG	NEG	NEG	NEG	NEG	NEG	POS	POS	AMB	POS	NEG	NEG	NEG	NEG	POS	POS	POS	POS	NEG
MSSA1942	NEG	NEG	NEG	NEG	NEG	NEG	NEG	POS	POS	AMB	POS	NEG	NEG	NEG	NEG	POS	POS	POS	POS	NEG
MSSA1957	NEG	NEG	NEG	NEG	NEG	NEG	NEG	POS	POS	AMB	POS	NEG	NEG	NEG	NEG	POS	POS	POS	POS	NEG
MSSA1748	NEG	NEG	NEG	NEG	NEG	NEG	NEG	POS	POS	AMB	POS	NEG	NEG	NEG	NEG	POS	POS	POS	POS	NEG
MSSA1754	NEG	NEG	NEG	NEG	NEG	NEG	NEG	POS	POS	AMB	POS	NEG	NEG	NEG	NEG	POS	POS	POS	POS	NEG
MSSA1770	NEG	NEG	NEG	NEG	NEG	NEG	NEG	POS	POS	AMB	POS	NEG	NEG	NEG	NEG	POS	POS	POS	POS	NEG
MSSA1788	NEG	NEG	NEG	NEG	NEG	NEG	NEG	POS	POS	NEG	POS	NEG	NEG	NEG	NEG	POS	POS	POS	POS	NEG
MSSA1804	NEG	NEG	NEG	NEG	NEG	NEG	NEG	POS	POS	AMB	POS	NEG	NEG	NEG	NEG	POS	POS	POS	POS	NEG
MSSA1818	NEG	NEG	NEG	NEG	NEG	NEG	NEG	POS	POS	AMB	POS	NEG	NEG	NEG	NEG	POS	POS	POS	POS	NEG
MSSA1914	NEG	NEG	NEG	NEG	NEG	NEG	NEG	POS	POS	AMB	POS	NEG	NEG	NEG	NEG	POS	POS	POS	POS	NEG
MSSA1974	NEG	NEG	NEG	NEG	NEG	NEG	NEG	POS	POS	NEG	POS	NEG	NEG	NEG	NEG	POS	POS	POS	POS	NEG
MSSA2023	NEG	NEG	NEG	NEG	NEG	NEG	NEG	POS	POS	AMB	POS	NEG	NEG	NEG	NEG	POS	POS	POS	POS	NEG
MSSA2030	NEG	NEG	NEG	NEG	NEG	NEG	NEG	POS	POS	AMB	POS	NEG	NEG	NEG	NEG	POS	POS	POS	POS	NEG
MSSA2096	NEG	NEG	NEG	AMB	NEG	AMB	NEG	POS	POS	NEG	AMB	NEG	NEG	NEG	NEG	POS	NEG	POS	POS	NEG
MSSA2142	NEG	NEG	NEG	NEG	NEG	NEG	NEG	POS	POS	AMB	POS	NEG	NEG	NEG	NEG	POS	POS	POS	POS	NEG
MSSA2203	NEG	NEG	NEG	NEG	NEG	NEG	NEG	POS	POS	AMB	POS	NEG	NEG	NEG	NEG	POS	POS	POS	POS	NEG
MSSA1901	POS	NEG	NEG	NEG	POS	POS	POS	POS	POS	AMB	POS	POS	NEG	POS	NEG	POS	POS	POS	POS	NEG
MSSA2013	POS	NEG	NEG	NEG	POS	POS	POS	POS	POS	AMB	POS	NEG	NEG	NEG	NEG	POS	POS	POS	POS	NEG
MSSA2031	POS	NEG	NEG	NEG	POS	POS	POS	POS	POS	AMB	POS	NEG	NEG	NEG	NEG	POS	POS	POS	POS	NEG
MSSA2052	POS	NEG	NEG	NEG	POS	POS	POS	POS	POS	AMB	POS	NEG	NEG	NEG	NEG	POS	POS	POS	POS	NEG
MSSA2057	POS	NEG	NEG	NEG	POS	POS	POS	POS	POS	AMB	POS	NEG	NEG	NEG	NEG	POS	POS	POS	POS	NEG
MSSA2081	POS	NEG	NEG	NEG	POS	NEG	NEG	POS	POS	NEG	POS	NEG	NEG	NEG	NEG	POS	POS	POS	POS	NEG
MSSA1955	POS	NEG	NEG	NEG	POS	NEG	NEG	POS	POS	AMB	POS	NEG	NEG	NEG	NEG	NEG	NEG	POS	POS	NEG
MSSA1941	NEG	POS	POS	NEG	NEG	POS	NEG	POS	NEG	POS	POS	NEG	NEG	NEG	NEG	NEG	POS	POS	POS	NEG
MRSA3838	NEG	NEG	NEG	NEG	NEG	NEG	NEG	POS	POS	AMB	AMB	NEG	NEG	NEG	NEG	NEG	NEG	POS	POS	NEG
MRSA3839	NEG	NEG	NEG	NEG	NEG	NEG	NEG	POS	POS	AMB	POS	NEG	NEG	NEG	NEG	NEG	NEG	POS	POS	NEG
MSSA1801	NEG	NEG	NEG	NEG	NEG	NEG	NEG	POS	POS	NEG	NEG	NEG	NEG	NEG	NEG	NEG	POS	POS	NEG	NEG
MSSA2131	NEG	NEG	NEG	NEG	NEG	NEG	NEG	POS	POS	AMB	POS	NEG	NEG	NEG	NEG	NEG	NEG	POS	POS	NEG
MSSA1792	POS	POS	POS	NEG	POS	NEG	NEG	POS	POS	NEG	POS	NEG	NEG	NEG	NEG	POS	POS	POS	POS	NEG
MSSA1796	NEG	NEG	NEG	NEG	NEG	NEG	NEG	POS	POS	AMB	POS	NEG	NEG	NEG	NEG	POS	POS	POS	POS	NEG
MSSA1817	NEG	NEG	NEG	NEG	NEG	NEG	NEG	POS	POS	AMB	POS	NEG	NEG	NEG	NEG	POS	POS	POS	POS	NEG
MSSA1911	AMB	NEG	NEG	NEG	NEG	AMB	NEG	NEG	NEG	NEG	NEG	NEG	NEG	NEG	NEG	NEG	NEG	NEG	NEG	NEG
MSSA1927	NEG	NEG	NEG	NEG	NEG	NEG	NEG	POS	POS	AMB	POS	NEG	NEG	NEG	NEG	POS	POS	POS	POS	NEG
MSSA1928	NEG	NEG	NEG	NEG	NEG	NEG	NEG	POS	POS	AMB	POS	NEG	NEG	NEG	NEG	POS	POS	POS	POS	NEG
MSSA1953	NEG	NEG	NEG	NEG	NEG	NEG	NEG	POS	POS	NEG	POS	NEG	NEG	NEG	NEG	POS	POS	POS	POS	NEG
MSSA2062	POS	NEG	NEG	NEG	POS	NEG	NEG	POS	POS	NEG	POS	NEG	NEG	NEG	NEG	POS	POS	POS	POS	NEG
MSSA2078	POS	NEG	NEG	NEG	POS	NEG	NEG	POS	POS	AMB	POS	NEG	NEG	NEG	NEG	NEG	POS	POS	POS	NEG
MSSA2136	NEG	NEG	NEG	NEG	NEG	NEG	NEG	POS	POS	AMB	POS	NEG	NEG	NEG	NEG	NEG	NEG	POS	POS	NEG

	VIRULENCE: HAEMOLYSINS									VIRULENCE: HLB-CONVERTING PHAGES				VIRULENCE: EXFOLIATIVE TOXINS			VIRULENCE: EPITHEL. DIFF. INHIBITORS		
Strain	hl	hla	hld	hlIII (cons)	hlIII (Other than RF122)	hlb (probe 1)	hlb (probe 2)	hlb (probe 3)	un-truncated hlb	sak	chp (probe 1)	chp (probe 2)	scn	etA	etB	etD	edinA	edinB	edinC
MSSA1805	POS	POS	POS	POS	POS	POS	POS	POS	NEG	POS	POS	POS	POS	NEG	NEG	NEG	NEG	NEG	NEG
MSSA1823	POS	POS	POS	POS	POS	POS	POS	POS	NEG	POS	NEG	NEG	POS	NEG	NEG	NEG	NEG	NEG	NEG
MSSA1837	POS	POS	POS	POS	POS	POS	POS	POS	NEG	POS	NEG	NEG	POS	NEG	NEG	NEG	NEG	NEG	NEG
MSSA2044	POS	POS	POS	POS	POS	POS	POS	POS	NEG	POS	NEG	NEG	POS	NEG	NEG	NEG	NEG	NEG	NEG
MSSA2046	POS	POS	POS	POS	POS	POS	POS	POS	NEG	POS	NEG	NEG	POS	NEG	NEG	NEG	NEG	NEG	NEG
MSSA2059	POS	POS	POS	POS	POS	POS	POS	POS	NEG	POS	NEG	NEG	POS	NEG	NEG	NEG	NEG	NEG	NEG
MSSA2076	POS	POS	POS	POS	POS	POS	POS	POS	NEG	POS	POS	POS	POS	NEG	NEG	NEG	NEG	NEG	NEG
MSSA2200	POS	POS	POS	POS	POS	POS	POS	POS	NEG	POS	NEG	NEG	POS	NEG	NEG	NEG	NEG	NEG	NEG
MRSA3797	POS	POS	POS	POS	POS	AMB	POS	AMB	NEG	POS	POS	POS	POS	NEG	NEG	NEG	NEG	NEG	NEG
MRSA3798	POS	POS	POS	POS	POS	POS	POS	POS	NEG	POS	POS	POS	POS	NEG	NEG	NEG	NEG	NEG	NEG
MRSA3799	POS	POS	POS	POS	POS	AMB	POS	AMB	NEG	POS	POS	POS	POS	NEG	NEG	NEG	NEG	NEG	NEG
MRSA3800	POS	POS	POS	POS	POS	POS	POS	POS	NEG	POS	POS	POS	POS	NEG	NEG	NEG	NEG	NEG	NEG
MRSA3801	POS	POS	POS	POS	POS	POS	POS	POS	NEG	POS	POS	POS	POS	NEG	NEG	NEG	NEG	NEG	NEG
MRSA3804	POS	POS	POS	POS	POS	POS	POS	POS	NEG	POS	POS	POS	POS	NEG	NEG	NEG	NEG	NEG	NEG
MRSA3806	POS	POS	POS	POS	POS	POS	POS	POS	NEG	POS	POS	POS	POS	NEG	NEG	NEG	NEG	NEG	NEG
MRSA3961	POS	POS	POS	POS	POS	POS	POS	POS	NEG	POS	POS	POS	POS	NEG	NEG	NEG	NEG	NEG	NEG
MRSA3962	POS	POS	POS	POS	POS	POS	POS	POS	NEG	POS	POS	POS	POS	NEG	NEG	NEG	NEG	NEG	NEG
MRSA3963	POS	NEG	POS	POS	POS	POS	POS	POS	NEG	POS	POS	POS	POS	NEG	NEG	NEG	NEG	NEG	NEG
MRSA3966	POS	NEG	POS	POS	POS	POS	POS	AMB	NEG	POS	POS	POS	POS	NEG	NEG	NEG	NEG	NEG	NEG
MRSA3978	POS	POS	POS	POS	POS	POS	POS	POS	NEG	POS	POS	POS	POS	NEG	NEG	NEG	NEG	NEG	NEG
MRSA3979	POS	POS	POS	POS	POS	AMB	POS	NEG	NEG	POS	POS	POS	POS	NEG	NEG	NEG	NEG	NEG	NEG
MRSA3980	POS	POS	POS	POS	POS	POS	POS	AMB	NEG	POS	POS	POS	POS	NEG	NEG	NEG	NEG	NEG	NEG
MRSA4179	POS	NEG	POS	POS	POS	POS	POS	POS	NEG	POS	POS	POS	POS	NEG	NEG	NEG	NEG	NEG	NEG
MRSA4216	POS	POS	POS	POS	POS	POS	POS	POS	NEG	POS	POS	POS	POS	NEG	NEG	NEG	NEG	NEG	NEG
MRSA4217	POS	POS	POS	POS	POS	POS	POS	POS	NEG	POS	POS	POS	POS	NEG	NEG	NEG	NEG	NEG	NEG
MRSA4220	POS	POS	POS	POS	POS	POS	POS	POS	NEG	POS	POS	POS	POS	NEG	NEG	NEG	NEG	NEG	NEG
MRSA4221	POS	POS	POS	POS	POS	POS	POS	POS	NEG	POS	POS	POS	POS	NEG	NEG	NEG	NEG	NEG	NEG
MRSA4222	POS	POS	POS	POS	POS	POS	POS	POS	NEG	POS	POS	POS	POS	NEG	NEG	NEG	AMB	NEG	NEG
MSSA1751	POS	POS	POS	POS	POS	POS	POS	POS	NEG	POS	POS	POS	POS	NEG	NEG	NEG	NEG	NEG	NEG
MSSA1760	POS	POS	POS	POS	POS	POS	POS	POS	NEG	POS	POS	POS	POS	NEG	NEG	NEG	NEG	NEG	NEG
MSSA1802	POS	POS	POS	POS	POS	POS	POS	POS	NEG	POS	POS	POS	POS	NEG	NEG	NEG	NEG	NEG	NEG
MSSA1809	POS	POS	POS	POS	POS	POS	POS	POS	NEG	POS	POS	POS	POS	NEG	NEG	NEG	NEG	NEG	NEG
MSSA1815	POS	POS	POS	POS	POS	POS	POS	POS	NEG	POS	POS	POS	POS	NEG	NEG	NEG	NEG	NEG	NEG
MSSA1829	POS	POS	POS	POS	POS	POS	POS	POS	NEG	POS	POS	POS	POS	NEG	NEG	NEG	NEG	NEG	NEG
MSSA1908	POS	POS	POS	POS	POS	POS	POS	POS	NEG	POS	POS	POS	POS	NEG	NEG	NEG	NEG	NEG	NEG
MSSA1960	POS	POS	POS	POS	POS	AMB	POS	AMB	NEG	POS	POS	POS	POS	NEG	NEG	NEG	NEG	NEG	NEG
MSSA1965	POS	POS	POS	POS	POS	POS	POS	POS	NEG	POS	POS	POS	POS	NEG	NEG	NEG	NEG	NEG	NEG
MSSA1977	POS	POS	POS	POS	POS	POS	POS	AMB	NEG	POS	NEG	NEG	POS	NEG	NEG	NEG	NEG	NEG	NEG
MSSA1978	POS	POS	POS	POS	POS	POS	POS	POS	NEG	POS	POS	POS	POS	NEG	NEG	NEG	NEG	NEG	NEG
MSSA1999	POS	POS	POS	POS	POS	POS	POS	POS	NEG	POS	NEG	NEG	POS	NEG	NEG	NEG	NEG	NEG	NEG
MSSA2003	POS	POS	POS	POS	POS	POS	POS	POS	NEG	POS	POS	POS	POS	NEG	NEG	NEG	NEG	NEG	NEG
MSSA2006	POS	POS	POS	POS	POS	POS	POS	POS	NEG	POS	POS	POS	POS	NEG	NEG	NEG	NEG	NEG	NEG
MSSA2008	POS	POS	POS	POS	POS	POS	POS	POS	NEG	POS	POS	POS	POS	NEG	NEG	NEG	NEG	NEG	NEG
MSSA2011	POS	POS	POS	POS	POS	AMB	POS	NEG	NEG	POS	POS	POS	POS	NEG	NEG	NEG	NEG	NEG	NEG
MSSA2017	POS	POS	POS	POS	POS	POS	POS	AMB	NEG	POS	POS	POS	POS	NEG	NEG	NEG	NEG	NEG	NEG
MSSA2022	POS	POS	POS	POS	POS	AMB	POS	AMB	NEG	POS	POS	POS	POS	NEG	NEG	NEG	NEG	NEG	NEG
MSSA2055	POS	POS	POS	POS	POS	POS	POS	POS	NEG	POS	POS	POS	POS	NEG	NEG	NEG	NEG	NEG	NEG
MSSA2060	POS	POS	POS	POS	POS	POS	POS	POS	NEG	POS	POS	POS	POS	NEG	NEG	NEG	NEG	NEG	NEG
MSSA2080	POS	POS	POS	POS	POS	NEG	POS	NEG	NEG	POS	POS	POS	POS	NEG	NEG	NEG	NEG	NEG	NEG
MSSA2082	POS	POS	POS	POS	POS	POS	POS	POS	NEG	POS	NEG	NEG	POS	NEG	NEG	NEG	NEG	NEG	NEG
MSSA2084	POS	POS	POS	POS	POS	AMB	POS	NEG	NEG	POS	POS	POS	POS	NEG	NEG	NEG	NEG	NEG	NEG
MSSA2087	POS	POS	POS	POS	POS	POS	POS	AMB	NEG	POS	NEG	NEG	POS	NEG	NEG	NEG	NEG	NEG	NEG
MSSA2094	POS	POS	POS	POS	POS	POS	POS	POS	NEG	POS	POS	POS	POS	NEG	NEG	NEG	NEG	NEG	NEG
MSSA2097	POS	POS	POS	POS	POS	POS	POS	POS	NEG	POS	NEG	NEG	POS	NEG	NEG	NEG	NEG	NEG	NEG
MSSA2110	POS	POS	POS	POS	POS	POS	POS	POS	NEG	POS	POS	POS	POS	NEG	NEG	NEG	NEG	NEG	NEG
MSSA2113	POS	POS	POS	POS	POS	POS	POS	POS	NEG	POS	POS	POS	POS	NEG	NEG	NEG	NEG	NEG	NEG
MSSA2132	POS	POS	POS	POS	POS	POS	POS	POS	NEG	POS	POS	POS	POS	NEG	NEG	NEG	NEG	NEG	NEG
MSSA2134	POS	POS	POS	POS	POS	POS	POS	AMB	NEG	POS	POS	POS	POS	NEG	NEG	NEG	NEG	NEG	NEG
MSSA1746	POS	POS	POS	POS	POS	POS	POS	AMB	NEG	POS	NEG	NEG	POS	NEG	NEG	NEG	NEG	NEG	NEG
MSSA1757	POS	POS	POS	POS	POS	POS	POS	POS	NEG	POS	NEG	NEG	POS	NEG	NEG	NEG	NEG	NEG	NEG
MSSA1772	POS	POS	POS	POS	POS	AMB	POS	AMB	NEG	POS	NEG	NEG	POS	NEG	NEG	NEG	NEG	NEG	NEG
MSSA1795	POS	POS	POS	POS	POS	POS	POS	POS	NEG	POS	NEG	NEG	POS	NEG	NEG	NEG	NEG	NEG	NEG
MSSA1813	POS	POS	POS	POS	POS	POS	POS	POS	NEG	POS	NEG	NEG	POS	NEG	NEG	NEG	NEG	NEG	NEG
MSSA1826	POS	POS	POS	POS	POS	POS	POS	POS	NEG	POS	NEG	NEG	POS	NEG	NEG	NEG	NEG	NEG	NEG
MSSA1830	POS	POS	POS	POS	POS	POS	POS	POS	NEG	POS	NEG	NEG	POS	NEG	NEG	NEG	NEG	NEG	NEG
MSSA1838	POS	POS	POS	POS	POS	POS	POS	POS	NEG	POS	NEG	NEG	POS	NEG	NEG	NEG	NEG	NEG	NEG
MSSA1944	POS	POS	POS	POS	POS	POS	POS	AMB	NEG	POS	NEG	NEG	POS	NEG	NEG	NEG	NEG	NEG	NEG
MSSA1947	POS	POS	POS	POS	POS	POS	POS	POS	NEG	POS	NEG	NEG	POS	NEG	NEG	NEG	NEG	NEG	NEG
MSSA1964	POS	POS	POS	POS	POS	POS	POS	POS	NEG	POS	NEG	NEG	POS	NEG	NEG	NEG	NEG	NEG	NEG
MSSA1969	POS	POS	POS	POS	POS	POS	POS	POS	NEG	POS	NEG	NEG	POS	NEG	NEG	NEG	NEG	NEG	NEG
MSSA2001	POS	POS	POS	POS	POS	POS	POS	POS	NEG	POS	NEG	NEG	POS	NEG	NEG	NEG	NEG	NEG	NEG
MSSA2004	POS	POS	POS	POS	POS	POS	POS	POS	NEG	POS	NEG	NEG	POS	NEG	NEG	NEG	NEG	NEG	NEG
MSSA2024	POS	POS	POS	POS	POS	AMB	POS	AMB	NEG	POS	NEG	NEG	POS	NEG	NEG	NEG	NEG	NEG	NEG
MSSA2025	POS	POS	POS	POS	POS	POS	POS	POS	NEG	POS	NEG	NEG	POS	NEG	NEG	NEG	NEG	NEG	NEG
MSSA2026	POS	POS	POS	POS	POS	POS	POS	POS	NEG	POS	NEG	NEG	POS	NEG	NEG	NEG	NEG	NEG	NEG
MSSA2032	POS	POS	POS	POS	POS	POS	POS	AMB	NEG	POS	NEG	NEG	POS	NEG	NEG	NEG	NEG	NEG	NEG
MSSA2043	POS	POS	POS	POS	POS	POS	POS	POS	NEG	POS	NEG	NEG	POS	NEG	NEG	NEG	NEG	NEG	NEG
MSSA2061	POS	POS	POS	POS	POS	POS	POS	POS	NEG	POS	NEG	NEG	POS	NEG	NEG	NEG	NEG	NEG	NEG
MSSA2085	POS	POS	POS	POS	POS	POS	POS	POS	NEG	POS	NEG	NEG	POS	NEG	NEG	NEG	NEG	NEG	NEG
MSSA2098	POS	POS	POS	POS	POS	POS	POS	POS	NEG	POS	NEG	NEG	POS	NEG	NEG	NEG	NEG	NEG	NEG
MSSA2100	POS	POS	POS	POS	POS	POS	POS	POS	NEG	POS	NEG	NEG	POS	NEG	NEG	NEG	NEG	NEG	NEG
MSSA2111	POS	POS	POS	POS	POS	POS	POS	POS	NEG	POS	NEG	NEG	POS	NEG	NEG	NEG	NEG	NEG	NEG
MSSA2178	POS	POS	POS	POS	POS	POS	POS	POS	NEG	POS	NEG	NEG	POS	NEG	NEG	NEG	NEG	NEG	NEG
MSSA2202	POS	POS	POS	POS	POS	POS	POS	POS	NEG	POS	NEG	NEG	POS	NEG	NEG	NEG	NEG	NEG	NEG
MSSA2205	POS	POS	POS	POS	POS	POS	POS	POS	NEG	POS	NEG	NEG	POS	NEG	NEG	NEG	NEG	NEG	NEG
MRSA3796	POS	POS	POS	POS	POS	POS	POS	POS	NEG	POS	NEG	NEG	POS	NEG	NEG	NEG	NEG	NEG	NEG
MRSA3805	POS	POS	POS	POS	POS	POS	POS	POS	POS	NEG	NEG	NEG	NEG	NEG	NEG	NEG	NEG	NEG	NEG
MRSA3965	POS	POS	POS	POS	POS	POS	POS	POS	NEG	POS	POS	POS	POS	NEG	NEG	NEG	NEG	NEG	NEG
MSSA1761	POS	POS	POS	POS	POS	POS	POS	POS	POS	NEG	NEG	NEG	POS	NEG	NEG	NEG	NEG	NEG	NEG
MSSA1764	POS	POS	POS	POS	POS	POS	POS	POS	NEG	POS	NEG	NEG	POS	NEG	NEG	NEG	NEG	NEG	NEG
MSSA1765	POS	POS	POS	POS	POS	POS	POS	POS	POS	NEG	NEG	NEG	NEG	NEG	NEG	NEG	NEG	NEG	NEG
MSSA1784	POS	POS	POS	POS	POS	POS	POS	POS	NEG	POS	POS	POS	POS	NEG	NEG	NEG	NEG	NEG	NEG
MSSA1806	POS	POS	POS	POS	POS	POS	POS	POS	NEG	POS	NEG	NEG	POS	NEG	NEG	NEG	NEG	NEG	NEG
MSSA1807	POS	POS	POS	POS	POS	POS	POS	POS	NEG	POS	NEG	NEG	POS	NEG	NEG	NEG	NEG	NEG	NEG
MSSA1888	POS	POS	POS	POS	POS	POS	POS	POS	NEG	POS	NEG	NEG	POS	NEG	NEG	NEG	NEG	NEG	NEG
MSSA1894	POS	POS	POS	POS	POS	POS	POS	POS	NEG	POS	POS	POS	POS	NEG	NEG	NEG	NEG	NEG	NEG
MSSA1922	POS	POS	POS	POS	POS	POS	POS	POS	NEG	POS	NEG	NEG	POS	NEG	NEG	NEG	NEG	NEG	NEG
MSSA1950	POS	POS	POS	POS	POS	POS	POS	POS	NEG	POS	NEG	NEG	POS	NEG	NEG	NEG	NEG	NEG	NEG
MSSA1954	POS	POS	POS	POS	POS	POS	POS	POS	NEG	POS	NEG	NEG	POS	NEG	NEG	NEG	NEG	NEG	NEG
MSSA1966	POS	POS	POS	POS	POS	POS	POS	POS	NEG	POS	NEG	NEG	POS	NEG	NEG	NEG	NEG	NEG	NEG
MSSA1972	POS	POS	POS	POS	POS	POS	POS	POS	NEG	POS	NEG	NEG	POS	NEG	NEG	NEG	NEG	NEG	NEG
MSSA2010	POS	POS	POS	POS	POS	POS	POS	POS	NEG	POS	NEG	NEG	POS	NEG	NEG	NEG	NEG	NEG	NEG
MSSA2020	POS	POS	POS	POS	POS	POS	POS	POS	NEG	POS	NEG	NEG	POS	NEG	NEG	NEG	NEG	NEG	NEG
MSSA2028	POS	POS	POS	POS	POS	POS	POS	POS	NEG	POS	POS	POS	POS	NEG	NEG	NEG	NEG	NEG	NEG
MSSA2088	POS	POS	POS	POS	POS	POS	POS	POS	NEG	POS	NEG	NEG	POS	NEG	NEG	NEG	NEG	NEG	NEG
MSSA2187	POS	POS	POS	POS	POS	POS	POS	POS	NEG	POS	NEG	NEG	POS	NEG	NEG	NEG	NEG	NEG	NEG
MSSA2204	POS	POS	POS	POS	POS	POS	POS	POS	NEG	POS	NEG	NEG	POS	NEG	NEG	NEG	NEG	NEG	NEG
MSSA2210	POS	POS	POS	POS	POS	POS	POS	POS	NEG	POS	NEG	NEG	POS	NEG	NEG	NEG	NEG	NEG	NEG
MSSA2223	POS	POS	POS	POS	POS	POS	POS	POS	NEG	POS	NEG	NEG	POS	NEG	NEG	NEG	NEG	NEG	NEG
MSSA1763	POS	POS	POS	POS	POS	POS	POS	POS	NEG	POS	POS	POS	POS	AMB	NEG	NEG	NEG	NEG	NEG
MSSA1777	POS	POS	POS	POS	POS	POS	POS	POS	NEG	POS	POS	POS	POS	NEG	NEG	NEG	NEG	NEG	NEG
MSSA2009	POS	POS	POS	POS	POS	POS	POS	POS	NEG	POS	POS	POS	POS	POS	NEG	NEG	NEG	NEG	NEG
MSSA2047	POS	POS	POS	POS	POS	POS	POS	POS	NEG	POS	POS	POS	POS	NEG	NEG	NEG	NEG	NEG	NEG
MSSA2079	POS	POS	POS	POS	POS	POS	POS	POS	NEG	POS	POS	POS	POS	NEG	NEG	NEG	NEG	NEG	NEG
MSSA2101	POS	POS	POS	POS	POS	POS	POS	POS	NEG	POS	POS	POS	POS	NEG	NEG	NEG	NEG	NEG	NEG
MSSA1821	POS	POS	POS	POS	POS	POS	POS	POS	NEG	POS	NEG	NEG	POS	NEG	NEG	NEG	NEG	NEG	NEG
MSSA1898	POS	POS	POS	POS	POS	POS	POS	POS	NEG	POS	NEG	NEG	POS	NEG	NEG	NEG	NEG	NEG	NEG
MSSA1973	POS	POS	POS	POS	POS	POS	POS	POS	NEG	POS	NEG	NEG	POS	NEG	NEG	NEG	NEG	NEG	NEG
MSSA2071	POS	POS	POS	POS	POS	POS	POS	POS	NEG	POS	NEG	NEG	POS	NEG	NEG	NEG	NEG	NEG	NEG
MSSA2083	POS	POS	POS	POS	POS	POS	POS	POS	NEG	POS	NEG	NEG	POS	NEG	NEG	NEG	NEG	NEG	NEG
MSSA2106	POS	POS	POS	POS	POS	POS	POS	POS	NEG	POS	NEG	NEG	POS	NEG	NEG	NEG	NEG	NEG	NEG
MSSA2112	POS	POS	POS	POS	POS	POS	POS	POS	NEG	POS	NEG	NEG	POS	NEG	NEG	NEG	NEG	NEG	NEG
MSSA2115	POS	POS	POS	POS	POS	POS	POS	POS	NEG	POS	NEG	NEG	POS	NEG	NEG	NEG	NEG	NEG	NEG
MSSA2195	POS	POS	POS	POS	POS	POS	POS	POS	NEG	POS	NEG	NEG	POS	NEG	NEG	NEG	NEG	NEG	NEG
MSSA2207	POS	POS	POS	POS	POS	POS	POS	POS	NEG	POS	NEG	NEG	POS	NEG	NEG	NEG	NEG	NEG	NEG
MSSA1758	POS	POS	POS	POS	POS	NEG	NEG	NEG	NEG	NEG	POS	POS	POS	NEG	NEG	NEG	NEG	NEG	NEG
MSSA1762	POS	POS	POS	POS	POS	NEG	NEG	POS	NEG	NEG	POS	POS	POS	NEG	NEG	NEG	NEG	NEG	NEG
MSSA1769	POS	POS	POS	POS	POS	NEG	NEG	POS	NEG	NEG	POS	POS	POS	POS	NEG	AMB	NEG	NEG	NEG
MSSA1771	POS	POS	POS	POS	POS	NEG	NEG	NEG	NEG	NEG	POS	POS	POS	NEG	NEG	NEG	NEG	NEG	NEG
MSSA1789	POS	POS	POS	POS	POS	NEG	NEG	NEG	NEG	NEG	POS	POS	POS	NEG	NEG	NEG	NEG	NEG	NEG
MSSA1800	POS	POS	POS	POS	POS	NEG	NEG	NEG	NEG	NEG	POS	POS	POS	NEG	NEG	NEG	AMB	NEG	NEG
MSSA1833	POS	POS	POS	POS	POS	NEG	NEG	AMB	NEG	NEG	POS	POS	POS	NEG	NEG	NEG	NEG	NEG	NEG
MSSA1890	POS	POS	POS	POS	POS	NEG	NEG	NEG	NEG	NEG	POS	POS	POS	NEG	NEG	NEG	NEG	NEG	NEG
MSSA1903	POS	POS	POS	POS	POS	NEG	NEG	POS	NEG	NEG	POS	POS	POS	NEG	NEG	NEG	NEG	NEG	NEG
MSSA1940	POS	POS	POS	POS	POS	NEG	NEG	AMB	NEG	NEG	POS	POS	POS	NEG	NEG	NEG	NEG	NEG	NEG
MSSA1962	POS	POS	POS	POS	POS	NEG	NEG	NEG	NEG	NEG	POS	POS	POS	NEG	NEG	NEG	NEG	NEG	NEG
MSSA1963	POS	POS	POS	POS	POS	NEG	NEG	POS	NEG	NEG	POS	POS	POS	NEG	NEG	NEG	NEG	NEG	NEG
MSSA2016	POS	POS	POS	POS	POS	NEG	NEG	NEG	NEG	NEG	POS	POS	POS	NEG	NEG	NEG	NEG	NEG	NEG
MSSA2018	POS	POS	POS	POS	POS	AMB	NEG	NEG	NEG	NEG	POS	POS	POS	NEG	NEG	NEG	NEG	NEG	NEG
MSSA2019	POS	POS	POS	POS	POS	NEG	NEG	AMB	NEG	NEG	POS	POS	POS	NEG	NEG	NEG	NEG	NEG	NEG
MSSA2029	POS	POS	POS	POS	POS	NEG	NEG	AMB	NEG	NEG	POS	POS	POS	NEG	NEG	NEG	NEG	NEG	NEG
MSSA2045	POS	POS	POS	POS	POS	NEG	NEG	AMB	NEG	NEG	POS	POS	POS	NEG	NEG	NEG	NEG	NEG	NEG
MSSA2048	POS	POS	POS	POS	POS	NEG	NEG	AMB	NEG	NEG	POS	POS	POS	NEG	NEG	NEG	NEG	NEG	NEG
MSSA2049	POS	POS	POS	POS	POS	NEG	NEG	AMB	NEG	NEG	POS	POS	POS	NEG	NEG	NEG	NEG	NEG	NEG
MSSA2050	POS	POS	POS	POS	POS	NEG	NEG	POS	NEG	NEG	POS	POS	POS	NEG	NEG	NEG	NEG	NEG	NEG
MSSA2099	POS	POS	POS	POS	POS	NEG	NEG	NEG	NEG	POS	POS	POS	POS	NEG	NEG	NEG	NEG	NEG	NEG
MSSA2109	POS	POS	POS	POS	POS	NEG	NEG	NEG	NEG	NEG	POS	POS	POS	NEG	NEG	NEG	NEG	NEG	NEG
MSSA2130	POS	POS	POS	POS	POS	NEG	NEG	POS	NEG	NEG	POS	POS	POS	NEG	NEG	NEG	NEG	NEG	NEG
MSSA2133	POS	POS	POS	POS	POS	NEG	NEG	AMB	NEG	NEG	POS	POS	POS	NEG	NEG	NEG	NEG	NEG	NEG
MSSA2139	POS	POS	POS	POS	POS	NEG	NEG	AMB	NEG	NEG	POS	POS	POS	NEG	NEG	NEG	NEG	NEG	NEG
MSSA2141	POS	POS	POS	POS	POS	NEG	NEG	POS	NEG	NEG	POS	POS	POS	NEG	NEG	NEG	NEG	NEG	NEG
MSSA2208	POS	POS	POS	POS	POS	NEG	NEG	AMB	NEG	NEG	POS	POS	POS	NEG	NEG	NEG	NEG	NEG	NEG
MSSA2209	POS	POS	POS	POS	POS	NEG	NEG	AMB	NEG	NEG	POS	POS	POS	NEG	NEG	NEG	NEG	NEG	NEG
MSSA1893	POS	POS	POS	POS	POS	AMB	POS	AMB	NEG	POS	POS	POS	POS	NEG	NEG	NEG	NEG	NEG	NEG
MSSA2105	POS	POS	POS	POS	POS	POS	POS	POS	NEG	POS	POS	POS	POS	NEG	NEG	NEG	NEG	NEG	NEG
MRSA3836	POS	POS	POS	NEG	NEG	POS	POS	POS	NEG	POS	POS	POS	POS	NEG	NEG	NEG	NEG	NEG	NEG
MRSA3840	POS	POS	POS	NEG	NEG	POS	POS	POS	NEG	POS	POS	POS	POS	NEG	NEG	NEG	NEG	NEG	NEG
MRSA4188	POS	POS	POS	NEG	NEG	POS	POS	POS	AMB	POS	POS	POS	POS	NEG	NEG	NEG	NEG	NEG	NEG
MSSA1814	POS	POS	POS	NEG	NEG	POS	POS	POS	POS	NEG	NEG	NEG	NEG	NEG	NEG	NEG	NEG	NEG	NEG
MSSA1816	POS	NEG	POS	NEG	NEG	POS	POS	POS	NEG	POS	POS	POS	POS	NEG	NEG	NEG	NEG	NEG	NEG
MSSA1825	POS	POS	POS	NEG	NEG	POS	POS	POS	NEG	POS	POS	POS	POS	NEG	NEG	NEG	NEG	NEG	NEG
MSSA1834	POS	NEG	POS	NEG	NEG	POS	POS	POS	NEG	POS	POS	POS	POS	NEG	NEG	NEG	NEG	NEG	NEG
MSSA1938	POS	POS	POS	NEG	NEG	POS	POS	POS	POS	NEG	NEG	NEG	NEG	NEG	NEG	NEG	NEG	NEG	NEG
MSSA1948	POS	POS	POS	NEG	NEG	POS	POS	POS	NEG	POS	POS	POS	POS	NEG	NEG	NEG	NEG	NEG	NEG
MSSA1951	POS	POS	POS	NEG	NEG	POS	POS	POS	NEG	POS	POS	POS	POS	NEG	NEG	NEG	NEG	NEG	NEG
MSSA2005	POS	POS	POS	NEG	NEG	POS	POS	POS	NEG	POS	POS	POS	POS	NEG	NEG	NEG	NEG	NEG	NEG
MSSA2051	POS	POS	POS	NEG	NEG	POS	POS	POS	NEG	POS	POS	POS	POS	NEG	NEG	NEG	NEG	NEG	NEG
MSSA2056	POS	POS	POS	NEG	NEG	POS	POS	POS	POS	NEG	NEG	NEG	NEG	NEG	NEG	NEG	NEG	NEG	NEG
MSSA2066	POS	POS	POS	NEG	NEG	POS	POS	POS	NEG	POS	POS	POS	POS	NEG	NEG	NEG	NEG	NEG	NEG
MSSA2067	POS	POS	POS	NEG	NEG	POS	POS	POS	NEG	POS	POS	POS	POS	NEG	NEG	NEG	NEG	NEG	NEG
MSSA2086	POS	POS	POS	NEG	NEG	POS	POS	POS	POS	NEG	NEG	NEG	NEG	NEG	NEG	NEG	NEG	NEG	NEG
MSSA2089	POS	POS	POS	NEG	NEG	POS	POS	POS	NEG	POS	POS	POS	POS	NEG	NEG	NEG	NEG	NEG	NEG
MSSA2103	POS	POS	POS	NEG	NEG	POS	POS	POS	NEG	POS	POS	POS	POS	NEG	NEG	NEG	NEG	NEG	NEG
MSSA2173	POS	POS	POS	NEG	NEG	POS	POS	POS	NEG	POS	POS	POS	POS	NEG	NEG	NEG	NEG	NEG	NEG
MSSA2183	POS	POS	POS	NEG	NEG	POS	POS	POS	POS	NEG	NEG	NEG	NEG	NEG	NEG	NEG	NEG	NEG	NEG
MSSA2196	POS	POS	POS	NEG	NEG	POS	POS	POS	NEG	POS	POS	POS	POS	NEG	NEG	NEG	NEG	NEG	NEG
MSSA2219	POS	POS	POS	NEG	NEG	POS	POS	POS	NEG	POS	POS	POS	POS	NEG	NEG	NEG	NEG	NEG	NEG
MSSA1753	POS	POS	POS	POS	POS	AMB	POS	NEG	NEG	POS	POS	POS	POS	NEG	NEG	POS	NEG	POS	NEG
MSSA1773	POS	POS	POS	POS	POS	POS	POS	AMB	NEG	POS	POS	POS	POS	NEG	NEG	POS	NEG	POS	NEG
MSSA1811	POS	POS	POS	POS	POS	POS	POS	POS	NEG	POS	POS	POS	POS	NEG	NEG	POS	NEG	POS	NEG
MSSA1897	POS	POS	POS	POS	POS	POS	POS	POS	NEG	POS	POS	POS	POS	NEG	NEG	POS	NEG	POS	NEG
MSSA1931	POS	POS	POS	POS	POS	POS	POS	POS	NEG	POS	POS	POS	POS	NEG	NEG	POS	NEG	POS	NEG
MSSA2114	POS	POS	POS	POS	POS	POS	POS	AMB	POS	NEG	NEG	NEG	NEG	NEG	NEG	POS	NEG	POS	NEG
MSSA1745	POS	POS	POS	POS	POS	POS	POS	POS	NEG	POS	POS	POS	POS	NEG	NEG	NEG	NEG	NEG	NEG
MSSA1747	POS	POS	POS	POS	POS	NEG	AMB	NEG	NEG	POS	POS	POS	POS	NEG	NEG	NEG	NEG	NEG	NEG
MSSA1749	POS	POS	POS	POS	POS	AMB	POS	NEG	NEG	POS	POS	POS	POS	NEG	NEG	NEG	NEG	NEG	NEG
MSSA1768	POS	POS	POS	POS	POS	POS	POS	POS	NEG	POS	POS	POS	POS	NEG	NEG	NEG	NEG	NEG	NEG
MSSA1774	POS	POS	POS	POS	POS	NEG	POS	NEG	NEG	POS	POS	POS	POS	NEG	NEG	NEG	NEG	NEG	NEG
MSSA1775	POS	POS	POS	POS	POS	POS	POS	POS	POS	NEG	NEG	NEG	NEG	NEG	NEG	NEG	NEG	NEG	NEG
MSSA1776	POS	POS	POS	POS	POS	POS	POS	POS	POS	NEG	NEG	NEG	NEG	NEG	NEG	NEG	NEG	NEG	NEG
MSSA1779	POS	POS	POS	POS	POS	NEG	AMB	NEG	NEG	POS	POS	POS	POS	NEG	NEG	NEG	NEG	NEG	NEG
MSSA1780	POS	POS	POS	POS	POS	POS	POS	AMB	NEG	POS	POS	POS	POS	NEG	NEG	NEG	NEG	NEG	NEG

Strain	VIRULENCE: HAEMOLYSINS									VIRULENCE: HLB-CONVERTING PHAGES				VIRULENCE: EXFOLIATIVE TOXINS			VIRULENCE: EPITHEL. DIFF. INHIBITORS		
Strain	hl	hla	hld	hlIII (cons)	hlIII (Other than RF122)	hlb (probe 1)	hlb (probe 2)	hlb (probe 3)	un-truncated hlb	sak	chp (probe 1)	chp (probe 2)	scn	etA	etB	etD	edinA	edinB	edinC
~~MSSA1781~~	POS	POS	POS	POS	POS	POS	POS	POS	NEG	POS	POS	POS	POS	NEG	NEG	NEG	NEG	NEG	NEG
MSSA1786	POS	POS	POS	POS	POS	POS	POS	POS	NEG	POS	POS	POS	POS	NEG	NEG	NEG	NEG	NEG	NEG
MSSA1793	POS	POS	POS	POS	POS	NEG	AMB	NEG	NEG	POS	POS	POS	POS	NEG	NEG	NEG	NEG	NEG	NEG
MSSA1803	POS	POS	POS	POS	POS	POS	POS	AMB	NEG	POS	POS	POS	POS	NEG	NEG	NEG	NEG	NEG	NEG
MSSA1836	POS	POS	POS	POS	POS	AMB	POS	AMB	NEG	POS	POS	POS	POS	NEG	NEG	NEG	NEG	NEG	NEG
MSSA1839	POS	POS	POS	POS	POS	AMB	POS	AMB	NEG	POS	POS	POS	POS	NEG	NEG	NEG	NEG	NEG	NEG
MSSA1900	POS	POS	POS	POS	POS	NEG	NEG	NEG	NEG	POS	POS	POS	POS	NEG	NEG	NEG	NEG	NEG	NEG
MSSA1910	POS	POS	POS	POS	POS	POS	POS	AMB	NEG	POS	NEG	NEG	POS	NEG	NEG	NEG	NEG	NEG	NEG
MSSA1918	POS	POS	POS	POS	POS	AMB	POS	NEG	NEG	POS	POS	POS	POS	NEG	NEG	NEG	NEG	NEG	NEG
MSSA1921	POS	POS	POS	POS	POS	POS	POS	POS	NEG	POS	POS	POS	POS	NEG	NEG	NEG	NEG	NEG	NEG
MSSA1923	POS	POS	POS	POS	POS	POS	POS	POS	NEG	POS	POS	POS	POS	NEG	NEG	NEG	NEG	NEG	NEG
MSSA1981	POS	POS	POS	POS	POS	POS	POS	POS	NEG	POS	NEG	NEG	POS	NEG	NEG	NEG	NEG	NEG	NEG
MSSA1998	POS	POS	POS	POS	POS	AMB	POS	NEG	POS	NEG	NEG	NEG	NEG	NEG	NEG	NEG	NEG	NEG	NEG
MSSA2000	POS	POS	POS	POS	POS	NEG	NEG	NEG	NEG	POS	POS	POS	POS	NEG	NEG	NEG	NEG	NEG	NEG
MSSA2007	POS	POS	POS	POS	POS	POS	POS	AMB	NEG	POS	POS	POS	POS	NEG	NEG	NEG	NEG	NEG	NEG
MSSA2012	POS	POS	POS	POS	POS	POS	POS	NEG	NEG	POS	POS	POS	POS	NEG	NEG	NEG	NEG	NEG	NEG
MSSA2015	POS	POS	POS	POS	POS	NEG	NEG	NEG	NEG	POS	POS	POS	POS	NEG	NEG	NEG	NEG	NEG	NEG
MSSA2021	POS	POS	POS	POS	POS	NEG	NEG	NEG	NEG	NEG	NEG	NEG	NEG	NEG	NEG	NEG	NEG	NEG	NEG
MSSA2027	POS	POS	POS	POS	POS	AMB	POS	NEG	NEG	POS	POS	POS	POS	NEG	NEG	NEG	NEG	NEG	NEG
MSSA2063	POS	POS	POS	POS	POS	AMB	POS	NEG	NEG	POS	POS	POS	POS	NEG	NEG	NEG	NEG	NEG	NEG
MSSA2064	POS	POS	POS	POS	POS	POS	POS	POS	NEG	POS	POS	POS	POS	NEG	NEG	NEG	NEG	NEG	NEG
MSSA2065	POS	POS	POS	POS	POS	AMB	POS	AMB	NEG	POS	POS	POS	POS	NEG	NEG	NEG	NEG	NEG	NEG
MSSA2075	POS	POS	POS	POS	POS	AMB	AMB	NEG	NEG	POS	POS	POS	POS	NEG	NEG	NEG	NEG	NEG	NEG
MSSA2093	POS	POS	POS	POS	POS	AMB	POS	AMB	POS	NEG	NEG	NEG	NEG	NEG	NEG	NEG	NEG	NEG	NEG
MSSA2102	POS	POS	POS	POS	POS	AMB	POS	NEG	NEG	POS	POS	POS	POS	NEG	NEG	NEG	NEG	NEG	NEG
MSSA2138	POS	POS	POS	POS	POS	POS	POS	POS	NEG	POS	POS	POS	POS	NEG	NEG	NEG	NEG	NEG	NEG
MSSA2168	POS	POS	POS	POS	POS	POS	POS	AMB	NEG	POS	POS	POS	POS	NEG	NEG	NEG	NEG	NEG	NEG
MSSA2181	POS	POS	POS	POS	POS	POS	POS	POS	NEG	POS	POS	POS	POS	NEG	NEG	NEG	NEG	NEG	NEG
MSSA2189	POS	POS	POS	POS	POS	POS	POS	POS	POS	POS	POS	POS	POS	NEG	NEG	NEG	NEG	NEG	NEG
MSSA2214	POS	POS	POS	POS	POS	AMB	POS	NEG	POS	POS	NEG	NEG	POS	NEG	NEG	NEG	NEG	NEG	NEG
MRSA3803	POS	POS	POS	POS	NEG	NEG	NEG	NEG	NEG	POS	POS	POS	POS	NEG	NEG	NEG	NEG	NEG	NEG
MSSA1750	POS	POS	POS	POS	NEG	NEG	NEG	NEG	NEG	POS	POS	POS	POS	NEG	NEG	NEG	NEG	NEG	NEG
MSSA1756	POS	POS	POS	POS	NEG	NEG	NEG	NEG	NEG	POS	POS	POS	POS	NEG	NEG	NEG	NEG	NEG	NEG
MSSA1759	POS	POS	POS	POS	NEG	NEG	NEG	NEG	NEG	POS	POS	POS	POS	NEG	NEG	NEG	NEG	NEG	NEG
MSSA1767	POS	POS	POS	POS	NEG	NEG	NEG	NEG	NEG	POS	POS	POS	POS	NEG	NEG	NEG	NEG	NEG	NEG
MSSA1787	POS	POS	POS	POS	NEG	NEG	NEG	NEG	NEG	POS	POS	POS	POS	NEG	NEG	NEG	NEG	NEG	NEG
MSSA1790	POS	POS	POS	POS	NEG	POS	POS	NEG	NEG	POS	POS	POS	POS	NEG	NEG	NEG	NEG	NEG	NEG
MSSA1794	POS	POS	POS	POS	NEG	NEG	NEG	NEG	POS	NEG	NEG	NEG	NEG	NEG	NEG	NEG	NEG	NEG	NEG
MSSA1808	POS	POS	POS	POS	NEG	NEG	NEG	NEG	NEG	POS	POS	POS	POS	NEG	NEG	NEG	NEG	NEG	NEG
MSSA1812	POS	POS	POS	POS	NEG	NEG	NEG	NEG	NEG	POS	POS	POS	POS	NEG	NEG	NEG	NEG	NEG	NEG
MSSA1819	POS	POS	POS	POS	NEG	NEG	NEG	NEG	NEG	POS	POS	POS	POS	NEG	NEG	NEG	NEG	NEG	NEG
MSSA1824	POS	POS	POS	POS	NEG	NEG	NEG	NEG	NEG	POS	POS	POS	POS	NEG	NEG	NEG	NEG	NEG	NEG
MSSA1827	POS	POS	POS	POS	NEG	NEG	NEG	NEG	NEG	POS	POS	POS	POS	NEG	NEG	NEG	NEG	NEG	NEG
MSSA1889	POS	POS	POS	POS	NEG	NEG	NEG	NEG	NEG	POS	POS	POS	POS	NEG	NEG	NEG	NEG	NEG	NEG
MSSA1895	POS	POS	POS	POS	NEG	NEG	NEG	NEG	NEG	POS	POS	POS	POS	NEG	NEG	NEG	NEG	NEG	NEG
MSSA1904	POS	POS	POS	POS	NEG	NEG	NEG	NEG	NEG	POS	POS	POS	POS	NEG	NEG	NEG	NEG	NEG	NEG
MSSA1905	POS	POS	POS	POS	NEG	NEG	NEG	NEG	NEG	POS	POS	POS	POS	NEG	NEG	NEG	NEG	NEG	NEG
MSSA1907	POS	POS	POS	POS	NEG	NEG	NEG	NEG	NEG	POS	POS	POS	POS	NEG	NEG	NEG	NEG	NEG	NEG
MSSA1919	POS	POS	POS	POS	NEG	NEG	NEG	NEG	NEG	POS	POS	POS	POS	NEG	NEG	NEG	NEG	NEG	NEG
MSSA1920	POS	POS	POS	POS	NEG	AMB	POS	NEG	NEG	POS	POS	POS	POS	NEG	NEG	NEG	NEG	NEG	NEG
MSSA1929	POS	POS	POS	POS	NEG	NEG	NEG	NEG	NEG	POS	POS	POS	POS	NEG	NEG	NEG	NEG	NEG	NEG
MSSA1930	POS	POS	POS	POS	NEG	NEG	NEG	NEG	NEG	POS	POS	POS	POS	NEG	NEG	NEG	NEG	NEG	NEG
MSSA1934	POS	POS	POS	POS	NEG	NEG	NEG	NEG	NEG	POS	POS	POS	POS	NEG	NEG	NEG	NEG	NEG	NEG
MSSA1946	POS	POS	POS	POS	NEG	AMB	POS	NEG	NEG	POS	POS	POS	POS	NEG	NEG	NEG	NEG	NEG	NEG
MSSA1971	POS	POS	POS	POS	NEG	NEG	NEG	NEG	POS	NEG	NEG	NEG	NEG	NEG	NEG	NEG	NEG	NEG	NEG
MSSA1980	POS	POS	POS	POS	NEG	NEG	NEG	NEG	NEG	POS	POS	POS	POS	NEG	NEG	NEG	NEG	NEG	NEG
MSSA1982	POS	POS	POS	POS	NEG	NEG	NEG	NEG	NEG	POS	POS	POS	POS	NEG	NEG	NEG	NEG	NEG	NEG
MSSA1996	POS	POS	POS	POS	NEG	NEG	NEG	NEG	NEG	POS	POS	POS	POS	NEG	NEG	NEG	NEG	NEG	NEG
MSSA2002	POS	POS	POS	POS	NEG	AMB	POS	NEG	NEG	POS	POS	POS	POS	NEG	NEG	NEG	NEG	NEG	NEG
MSSA2014	POS	POS	POS	POS	NEG	NEG	NEG	NEG	NEG	POS	POS	POS	POS	NEG	NEG	NEG	NEG	NEG	NEG
MSSA2053	POS	POS	POS	POS	NEG	NEG	NEG	NEG	NEG	POS	POS	POS	POS	NEG	NEG	NEG	NEG	NEG	NEG
MSSA2054	POS	POS	POS	POS	NEG	NEG	NEG	NEG	NEG	POS	POS	POS	POS	NEG	NEG	NEG	NEG	NEG	NEG
MSSA2058	AMB	AMB	POS	POS	NEG	NEG	NEG	NEG	NEG	POS	POS	POS	POS	NEG	NEG	NEG	NEG	NEG	NEG
MSSA2068	POS	POS	POS	POS	NEG	NEG	NEG	NEG	NEG	POS	POS	POS	POS	NEG	NEG	NEG	NEG	NEG	NEG
MSSA2070	POS	POS	POS	POS	NEG	NEG	NEG	NEG	NEG	POS	POS	POS	POS	NEG	NEG	NEG	NEG	NEG	NEG
MSSA2072	POS	POS	POS	POS	NEG	AMB	POS	NEG	NEG	POS	POS	POS	POS	NEG	NEG	NEG	NEG	NEG	NEG
MSSA2073	NEG	AMB	POS	POS	NEG	NEG	NEG	NEG	NEG	POS	POS	POS	POS	NEG	NEG	NEG	NEG	NEG	NEG
MSSA2074	POS	POS	POS	POS	NEG	NEG	NEG	NEG	NEG	POS	POS	POS	POS	NEG	NEG	NEG	NEG	NEG	NEG
MSSA2107	POS	POS	POS	POS	NEG	NEG	NEG	NEG	NEG	POS	POS	POS	POS	NEG	NEG	NEG	NEG	NEG	NEG
MSSA2108	POS	POS	POS	POS	NEG	NEG	AMB	NEG	NEG	POS	POS	POS	POS	NEG	NEG	NEG	NEG	NEG	NEG
MSSA2129	POS	POS	POS	POS	NEG	NEG	NEG	NEG	NEG	POS	POS	POS	POS	NEG	NEG	NEG	NEG	NEG	NEG
MSSA2135	POS	POS	POS	POS	NEG	NEG	NEG	NEG	NEG	POS	POS	POS	POS	NEG	NEG	NEG	NEG	NEG	NEG
MSSA2137	POS	POS	POS	POS	NEG	NEG	NEG	NEG	POS	NEG	NEG	NEG	NEG	NEG	NEG	NEG	NEG	NEG	NEG
MSSA2140	POS	POS	POS	POS	NEG	NEG	NEG	NEG	NEG	POS	POS	POS	POS	NEG	NEG	NEG	NEG	NEG	NEG
MSSA2182	POS	POS	POS	POS	NEG	AMB	POS	NEG	NEG	POS	POS	POS	POS	NEG	NEG	NEG	NEG	NEG	NEG
MSSA2188	POS	POS	POS	POS	NEG	NEG	NEG	NEG	NEG	POS	POS	POS	POS	NEG	NEG	NEG	NEG	NEG	NEG
MSSA1899	POS	POS	POS	POS	NEG	NEG	NEG	POS	POS	NEG	POS	POS	POS	NEG	NEG	NEG	NEG	NEG	NEG
MSSA1915	POS	POS	POS	POS	NEG	NEG	NEG	POS	POS	NEG	POS	POS	POS	NEG	NEG	NEG	NEG	NEG	NEG
MSSA1949	POS	POS	POS	POS	NEG	NEG	NEG	POS	POS	POS	POS	POS	POS	NEG	NEG	NEG	NEG	NEG	NEG
MSSA2042	POS	POS	POS	POS	NEG	NEG	NEG	AMB	NEG	POS	POS	POS	POS	NEG	NEG	NEG	NEG	NEG	NEG
MSSA2077	POS	POS	POS	POS	NEG	NEG	NEG	NEG	POS	NEG	POS	POS	POS	NEG	NEG	NEG	NEG	NEG	NEG
MSSA2095	POS	POS	POS	POS	NEG	AMB	NEG	POS	POS	NEG	POS	POS	POS	NEG	NEG	NEG	NEG	NEG	NEG
MSSA1997	POS	POS	POS	POS	POS	POS	POS	POS	NEG	POS	NEG	NEG	POS	NEG	NEG	NEG	NEG	NEG	NEG
MSSA1810	POS	POS	POS	POS	POS	POS	POS	POS	NEG	POS	POS	POS	POS	NEG	NEG	POS	NEG	POS	NEG
MSSA1832	POS	POS	POS	POS	POS	POS	POS	POS	NEG	POS	NEG	NEG	POS	NEG	NEG	NEG	NEG	NEG	NEG
MSSA1891	POS	POS	POS	POS	POS	POS	POS	POS	AMB	POS	POS	POS	POS	NEG	NEG	NEG	NEG	NEG	NEG
MSSA1892	POS	POS	POS	POS	POS	POS	POS	POS	NEG	POS	NEG	NEG	POS	NEG	NEG	NEG	NEG	NEG	NEG
MSSA1932	POS	POS	POS	POS	POS	POS	POS	POS	NEG	POS	NEG	NEG	POS	NEG	NEG	NEG	NEG	NEG	NEG
MSSA1942	POS	POS	POS	POS	POS	POS	POS	POS	POS	NEG	NEG	NEG	NEG	NEG	NEG	NEG	NEG	NEG	NEG
MSSA1957	POS	POS	POS	POS	POS	POS	POS	POS	NEG	POS	NEG	NEG	POS	NEG	NEG	NEG	NEG	NEG	NEG
MSSA1748	POS	POS	POS	POS	POS	POS	POS	POS	NEG	POS	NEG	NEG	POS	NEG	NEG	NEG	NEG	NEG	NEG
MSSA1754	POS	POS	POS	POS	POS	POS	POS	POS	NEG	POS	NEG	NEG	POS	NEG	NEG	NEG	NEG	NEG	NEG
MSSA1770	POS	POS	POS	POS	POS	POS	POS	POS	NEG	POS	NEG	NEG	POS	NEG	NEG	NEG	NEG	NEG	NEG
MSSA1788	POS	POS	POS	POS	POS	POS	POS	POS	NEG	POS	NEG	NEG	POS	NEG	NEG	NEG	NEG	NEG	NEG
MSSA1804	POS	POS	POS	POS	POS	POS	POS	POS	NEG	POS	NEG	NEG	POS	NEG	NEG	NEG	NEG	NEG	NEG
MSSA1818	POS	POS	POS	POS	POS	POS	POS	POS	NEG	POS	NEG	NEG	POS	NEG	NEG	NEG	NEG	NEG	NEG
MSSA1914	POS	POS	POS	POS	POS	POS	POS	POS	NEG	POS	NEG	NEG	POS	NEG	NEG	NEG	NEG	NEG	NEG
MSSA1974	POS	POS	POS	POS	POS	POS	POS	POS	NEG	POS	NEG	NEG	POS	NEG	NEG	NEG	NEG	NEG	NEG
MSSA2023	POS	POS	POS	POS	POS	POS	POS	POS	NEG	POS	NEG	NEG	POS	NEG	NEG	NEG	NEG	NEG	NEG
MSSA2030	POS	POS	POS	POS	POS	POS	POS	POS	NEG	POS	NEG	NEG	POS	NEG	NEG	NEG	NEG	NEG	NEG
MSSA2096	POS	AMB	POS	POS	POS	POS	POS	AMB	NEG	POS	NEG	NEG	POS	NEG	NEG	NEG	NEG	NEG	NEG
MSSA2142	POS	POS	POS	POS	POS	POS	POS	POS	NEG	POS	NEG	NEG	POS	NEG	NEG	NEG	NEG	NEG	NEG
MSSA2203	POS	POS	POS	POS	POS	POS	POS	POS	NEG	POS	NEG	NEG	POS	NEG	NEG	NEG	NEG	NEG	NEG
MSSA1901	POS	POS	POS	POS	NEG	POS	POS	POS	NEG	POS	NEG	NEG	POS	NEG	NEG	NEG	NEG	NEG	NEG
MSSA2013	POS	POS	POS	POS	NEG	POS	POS	POS	NEG	POS	NEG	NEG	POS	NEG	NEG	NEG	NEG	NEG	NEG
MSSA2031	POS	POS	POS	POS	NEG	POS	POS	POS	AMB	POS	NEG	NEG	POS	NEG	NEG	NEG	NEG	NEG	NEG
MSSA2052	POS	POS	POS	POS	NEG	POS	POS	POS	NEG	POS	NEG	NEG	POS	POS	NEG	NEG	NEG	NEG	NEG
MSSA2057	POS	POS	POS	POS	NEG	POS	POS	POS	NEG	POS	NEG	NEG	POS	POS	POS	NEG	NEG	NEG	POS
MSSA2081	POS	POS	POS	POS	NEG	NEG	NEG	POS	NEG	POS	POS	POS	POS	NEG	NEG	NEG	NEG	NEG	NEG
MSSA1955	POS	POS	POS	POS	NEG	POS	POS	POS	NEG	POS	POS	POS	POS	NEG	NEG	NEG	NEG	NEG	NEG
MSSA1941	POS	POS	POS	POS	NEG	NEG	NEG	NEG	NEG	POS	POS	POS	POS	NEG	NEG	NEG	NEG	NEG	NEG
MRSA3838	POS	POS	POS	POS	POS	NEG	NEG	NEG	AMB	NEG	NEG	NEG	NEG	NEG	NEG	NEG	NEG	NEG	NEG
MRSA3839	POS	POS	POS	POS	POS	NEG	NEG	NEG	POS	NEG	NEG	NEG	NEG	NEG	NEG	NEG	NEG	NEG	NEG
MSSA1801	POS	POS	POS	POS	POS	NEG	NEG	NEG	NEG	POS	POS	POS	POS	NEG	NEG	POS	NEG	POS	NEG
MSSA2131	POS	POS	POS	POS	POS	POS	POS	NEG	NEG	NEG	POS	POS	POS	NEG	NEG	NEG	NEG	NEG	NEG
MSSA1792	POS	POS	POS	POS	POS	POS	POS	POS	NEG	POS	POS	POS	POS	NEG	NEG	NEG	NEG	NEG	NEG
MSSA1796	POS	POS	POS	POS	NEG	POS	POS	POS	POS	NEG	NEG	NEG	NEG	NEG	NEG	NEG	NEG	NEG	NEG
MSSA1817	POS	POS	POS	POS	NEG	NEG	NEG	POS	POS	NEG	NEG	NEG	NEG	NEG	NEG	POS	NEG	POS	NEG
MSSA1911	NEG	NEG	POS	NEG	NEG	NEG	NEG	NEG	NEG	POS	POS	POS	POS	NEG	NEG	NEG	NEG	NEG	NEG
MSSA1927	POS	POS	POS	POS	POS	POS	POS	POS	NEG	POS	NEG	NEG	POS	NEG	NEG	NEG	NEG	NEG	NEG
MSSA1928	POS	POS	POS	POS	POS	POS	POS	POS	NEG	POS	NEG	NEG	POS	NEG	NEG	NEG	NEG	NEG	NEG
MSSA1953	POS	POS	POS	POS	POS	POS	POS	POS	NEG	POS	NEG	NEG	POS	NEG	NEG	NEG	NEG	NEG	NEG
MSSA2062	POS	POS	POS	POS	POS	POS	POS	POS	NEG	POS	POS	POS	POS	NEG	NEG	NEG	NEG	NEG	NEG
MSSA2078	POS	POS	POS	POS	NEG	POS	POS	NEG	NEG	POS	POS	POS	POS	NEG	NEG	NEG	NEG	NEG	NEG
MSSA2136	POS	POS	POS	POS	POS	POS	POS	POS	NEG	POS	POS	POS	POS	NEG	NEG	NEG	NEG	NEG	NEG

	VIRULENCE: ACME LOCUS				VIRULENCE: PROTEASES										
Strain	arcA-SCC	arcB-SCC	arcC-SCC	arcD-SCC	aur (cons)	aur (Other than MRSA252)	aur MRSA252	splA	splB	splE	sspA	sspB (probe 1)	sspB (probe 2)	sspP (probe 1)	sspP (probe 2)
MSSA1805	NEG	NEG	NEG	NEG	POS	POS	NEG	POS	POS	POS	POS	POS	POS	POS	POS
MSSA1823	NEG	NEG	NEG	NEG	POS	POS	NEG	POS	POS	POS	POS	POS	POS	POS	POS
MSSA1837	NEG	NEG	NEG	NEG	POS	POS	NEG	POS	POS	POS	POS	POS	POS	POS	POS
MSSA2044	NEG	NEG	NEG	NEG	POS	POS	NEG	POS	POS	POS	POS	POS	POS	POS	POS
MSSA2046	NEG	NEG	NEG	NEG	POS	POS	NEG	POS	POS	POS	POS	POS	POS	POS	POS
MSSA2059	NEG	NEG	NEG	NEG	POS	POS	NEG	POS	POS	POS	POS	POS	POS	POS	POS
MSSA2076	NEG	NEG	NEG	NEG	POS	POS	NEG	POS	POS	POS	POS	POS	POS	POS	POS
MSSA2200	NEG	NEG	NEG	NEG	POS	POS	NEG	POS	POS	POS	POS	POS	POS	POS	POS
MRSA3797	NEG	NEG	NEG	NEG	POS	POS	NEG	POS	POS	NEG	POS	POS	POS	POS	POS
MRSA3798	NEG	NEG	NEG	NEG	POS	POS	NEG	POS	POS	NEG	POS	POS	POS	POS	POS
MRSA3799	NEG	NEG	NEG	NEG	POS	POS	NEG	POS	POS	NEG	POS	POS	POS	POS	POS
MRSA3800	NEG	NEG	NEG	NEG	POS	POS	NEG	POS	POS	NEG	POS	POS	POS	POS	POS
MRSA3801	NEG	NEG	NEG	NEG	POS	POS	NEG	POS	POS	NEG	POS	POS	POS	POS	POS
MRSA3804	NEG	NEG	NEG	NEG	POS	POS	NEG	POS	POS	NEG	POS	POS	POS	POS	POS
MRSA3806	NEG	NEG	NEG	NEG	POS	POS	NEG	POS	POS	NEG	POS	POS	POS	POS	POS
MRSA3961	NEG	NEG	NEG	NEG	POS	POS	NEG	POS	POS	NEG	POS	POS	POS	POS	POS
MRSA3962	NEG	NEG	NEG	NEG	POS	POS	NEG	POS	POS	NEG	POS	POS	POS	POS	POS
MRSA3963	NEG	NEG	NEG	NEG	POS	POS	NEG	POS	POS	NEG	POS	POS	POS	POS	POS
MRSA3966	NEG	NEG	NEG	NEG	POS	POS	NEG	POS	POS	NEG	POS	POS	POS	POS	POS
MRSA3978	NEG	NEG	NEG	NEG	POS	POS	NEG	POS	POS	NEG	POS	POS	POS	POS	POS
MRSA3979	NEG	NEG	NEG	NEG	POS	POS	NEG	POS	POS	NEG	POS	POS	POS	POS	POS
MRSA3980	NEG	NEG	NEG	NEG	POS	POS	NEG	POS	POS	NEG	POS	POS	POS	POS	POS
MRSA4179	NEG	NEG	NEG	NEG	POS	POS	NEG	POS	POS	NEG	POS	POS	POS	POS	POS
MRSA4216	NEG	NEG	NEG	NEG	POS	POS	NEG	POS	POS	NEG	POS	POS	POS	POS	POS
MRSA4217	NEG	NEG	NEG	NEG	POS	POS	NEG	POS	POS	NEG	POS	POS	POS	POS	POS
MRSA4220	NEG	NEG	NEG	NEG	POS	POS	NEG	POS	POS	NEG	POS	POS	POS	POS	POS
MRSA4221	NEG	NEG	NEG	NEG	POS	POS	NEG	POS	POS	NEG	POS	POS	POS	POS	POS
MRSA4222	NEG	NEG	NEG	NEG	POS	POS	NEG	POS	POS	NEG	POS	POS	POS	POS	POS
MSSA1751	NEG	NEG	NEG	NEG	POS	POS	NEG	POS	POS	NEG	POS	POS	POS	POS	POS
MSSA1760	NEG	NEG	NEG	NEG	POS	POS	NEG	POS	POS	NEG	POS	POS	POS	POS	POS
MSSA1802	NEG	NEG	NEG	NEG	POS	POS	NEG	POS	POS	NEG	POS	POS	POS	POS	POS
MSSA1809	NEG	NEG	NEG	NEG	POS	POS	NEG	POS	POS	NEG	POS	POS	POS	POS	POS
MSSA1815	NEG	NEG	NEG	NEG	POS	POS	NEG	POS	POS	NEG	POS	POS	POS	POS	POS
MSSA1829	NEG	NEG	NEG	NEG	POS	POS	NEG	POS	POS	NEG	POS	POS	POS	POS	POS
MSSA1908	NEG	NEG	NEG	NEG	POS	POS	NEG	POS	POS	NEG	POS	POS	POS	POS	POS
MSSA1960	NEG	NEG	NEG	NEG	POS	POS	NEG	POS	POS	NEG	POS	POS	POS	POS	POS
MSSA1965	NEG	NEG	NEG	NEG	POS	POS	NEG	POS	POS	NEG	POS	POS	POS	POS	POS
MSSA1977	NEG	NEG	NEG	NEG	POS	POS	NEG	POS	POS	NEG	POS	POS	POS	POS	POS
MSSA1978	NEG	NEG	NEG	NEG	POS	POS	NEG	POS	POS	NEG	POS	POS	POS	POS	POS
MSSA1999	NEG	NEG	NEG	NEG	POS	POS	NEG	POS	POS	NEG	POS	POS	POS	POS	POS
MSSA2003	NEG	NEG	NEG	NEG	POS	POS	NEG	POS	POS	NEG	POS	POS	POS	POS	POS
MSSA2006	NEG	NEG	NEG	NEG	POS	POS	NEG	POS	POS	NEG	POS	POS	POS	POS	POS
MSSA2008	NEG	NEG	NEG	NEG	POS	POS	NEG	POS	POS	NEG	POS	POS	POS	POS	POS
MSSA2011	NEG	NEG	NEG	NEG	POS	POS	NEG	POS	POS	NEG	POS	POS	POS	POS	POS
MSSA2017	NEG	NEG	NEG	NEG	POS	POS	NEG	POS	POS	NEG	POS	POS	POS	POS	POS
MSSA2022	NEG	NEG	NEG	NEG	POS	POS	NEG	POS	POS	NEG	POS	POS	POS	POS	POS
MSSA2055	NEG	NEG	NEG	NEG	POS	POS	NEG	POS	POS	NEG	POS	POS	POS	POS	POS
MSSA2060	NEG	NEG	NEG	NEG	POS	POS	NEG	POS	POS	NEG	POS	POS	POS	POS	POS
MSSA2080	NEG	NEG	NEG	NEG	POS	POS	NEG	POS	POS	NEG	POS	POS	POS	POS	POS
MSSA2082	NEG	NEG	NEG	NEG	POS	POS	NEG	POS	POS	NEG	POS	POS	POS	POS	POS
MSSA2084	NEG	NEG	NEG	NEG	POS	POS	NEG	POS	POS	NEG	POS	POS	POS	POS	POS
MSSA2087	NEG	NEG	NEG	NEG	POS	POS	NEG	POS	POS	NEG	POS	POS	POS	POS	POS
MSSA2094	NEG	NEG	NEG	NEG	POS	POS	NEG	POS	POS	NEG	POS	POS	POS	POS	POS
MSSA2097	NEG	NEG	NEG	NEG	POS	POS	NEG	POS	POS	NEG	POS	POS	POS	POS	POS
MSSA2110	NEG	NEG	NEG	NEG	POS	POS	NEG	POS	POS	NEG	POS	POS	POS	POS	POS
MSSA2113	NEG	NEG	NEG	NEG	POS	POS	NEG	POS	POS	NEG	POS	POS	POS	POS	POS
MSSA2132	NEG	NEG	NEG	NEG	POS	POS	NEG	POS	POS	NEG	POS	POS	POS	POS	POS
MSSA2134	NEG	NEG	NEG	NEG	POS	POS	NEG	POS	POS	NEG	POS	POS	POS	POS	POS
MSSA1746	NEG	NEG	NEG	NEG	POS	POS	NEG	POS	POS	POS	POS	POS	POS	POS	POS
MSSA1757	NEG	NEG	NEG	NEG	POS	POS	NEG	POS	POS	POS	POS	POS	POS	POS	POS
MSSA1772	NEG	NEG	NEG	NEG	POS	POS	NEG	POS	POS	POS	POS	POS	POS	POS	POS
MSSA1795	NEG	NEG	NEG	NEG	POS	POS	NEG	POS	POS	POS	POS	POS	POS	POS	POS
MSSA1813	NEG	NEG	NEG	NEG	POS	POS	NEG	POS	POS	POS	POS	POS	POS	POS	POS
MSSA1826	NEG	NEG	NEG	NEG	POS	POS	NEG	POS	POS	POS	POS	POS	POS	POS	POS
MSSA1830	NEG	NEG	NEG	NEG	POS	POS	NEG	POS	POS	POS	POS	POS	POS	POS	POS
MSSA1838	NEG	NEG	NEG	NEG	POS	POS	NEG	POS	POS	POS	POS	POS	POS	POS	POS
MSSA1944	NEG	NEG	NEG	NEG	POS	POS	NEG	POS	POS	POS	POS	POS	POS	POS	POS
MSSA1947	NEG	NEG	NEG	NEG	POS	POS	NEG	POS	POS	POS	POS	POS	POS	POS	POS
MSSA1964	NEG	NEG	NEG	NEG	POS	POS	NEG	POS	POS	POS	POS	POS	POS	POS	POS
MSSA1969	NEG	NEG	NEG	NEG	POS	POS	NEG	POS	POS	POS	POS	POS	POS	POS	POS
MSSA2001	NEG	NEG	NEG	NEG	POS	POS	NEG	POS	POS	POS	POS	POS	POS	POS	POS
MSSA2004	NEG	NEG	NEG	NEG	POS	POS	NEG	POS	POS	POS	POS	POS	POS	POS	POS
MSSA2024	NEG	NEG	NEG	NEG	POS	POS	NEG	POS	POS	POS	POS	POS	POS	POS	POS
MSSA2025	NEG	NEG	NEG	NEG	POS	POS	NEG	POS	POS	POS	POS	POS	POS	POS	POS
MSSA2026	NEG	NEG	NEG	NEG	POS	POS	NEG	POS	POS	POS	POS	POS	POS	POS	POS
MSSA2032	NEG	NEG	NEG	NEG	POS	POS	NEG	POS	POS	POS	POS	POS	POS	POS	POS
MSSA2043	NEG	NEG	NEG	NEG	POS	POS	NEG	POS	POS	POS	POS	POS	POS	POS	POS
MSSA2061	NEG	NEG	NEG	NEG	POS	POS	NEG	POS	POS	POS	POS	POS	POS	POS	POS
MSSA2085	NEG	NEG	NEG	NEG	POS	POS	NEG	POS	POS	POS	POS	POS	POS	POS	POS
MSSA2098	NEG	NEG	NEG	NEG	POS	POS	NEG	POS	POS	POS	POS	POS	POS	POS	POS
MSSA2100	NEG	NEG	NEG	NEG	POS	POS	NEG	POS	POS	POS	POS	POS	POS	POS	POS
MSSA2111	NEG	NEG	NEG	NEG	POS	POS	NEG	POS	POS	POS	POS	POS	POS	POS	POS
MSSA2178	NEG	NEG	NEG	NEG	POS	POS	NEG	POS	POS	POS	POS	POS	POS	POS	POS
MSSA2202	NEG	NEG	NEG	NEG	POS	POS	NEG	POS	POS	POS	POS	POS	POS	POS	POS
MSSA2205	NEG	NEG	NEG	NEG	POS	POS	NEG	POS	POS	POS	POS	POS	POS	POS	POS
MRSA3796	NEG	NEG	NEG	NEG	POS	POS	NEG	POS	POS	POS	POS	POS	POS	POS	POS
MRSA3805	NEG	NEG	NEG	NEG	POS	NEG	POS	POS	POS	NEG	POS	POS	POS	POS	POS
MRSA3965	NEG	NEG	NEG	NEG	POS	POS	NEG	POS	POS	NEG	POS	POS	POS	POS	POS
MSSA1761	NEG	NEG	NEG	NEG	POS	POS	NEG	POS	NEG	POS	POS	POS	POS	POS	POS
MSSA1764	NEG	NEG	NEG	NEG	POS	POS	NEG	POS	POS	POS	POS	POS	POS	POS	POS
MSSA1765	NEG	NEG	NEG	NEG	POS	POS	NEG	POS	POS	POS	POS	POS	POS	POS	POS
MSSA1784	NEG	NEG	NEG	NEG	POS	POS	NEG	POS	POS	POS	POS	POS	POS	POS	POS
MSSA1806	NEG	NEG	NEG	NEG	POS	POS	NEG	POS	POS	POS	POS	POS	POS	POS	POS
MSSA1807	NEG	NEG	NEG	NEG	POS	POS	NEG	POS	POS	POS	POS	POS	POS	POS	POS
MSSA1888	NEG	NEG	NEG	NEG	POS	POS	NEG	POS	POS	NEG	POS	POS	POS	POS	POS
MSSA1894	NEG	NEG	NEG	NEG	POS	POS	NEG	POS	POS	POS	POS	POS	POS	POS	POS
MSSA1922	NEG	NEG	NEG	NEG	POS	POS	NEG	POS	POS	POS	POS	POS	POS	POS	POS
MSSA1950	NEG	NEG	NEG	NEG	POS	POS	NEG	POS	POS	POS	POS	POS	POS	POS	POS
MSSA1954	NEG	NEG	NEG	NEG	POS	POS	NEG	POS	POS	POS	POS	POS	POS	POS	POS
MSSA1966	NEG	NEG	NEG	NEG	POS	POS	NEG	POS	POS	NEG	POS	POS	POS	POS	POS
MSSA1972	NEG	NEG	NEG	NEG	POS	POS	NEG	POS	POS	POS	POS	POS	POS	POS	POS
MSSA2010	NEG	NEG	NEG	NEG	POS	POS	NEG	POS	POS	NEG	POS	POS	POS	POS	POS
MSSA2020	NEG	NEG	NEG	NEG	POS	POS	NEG	POS	POS	POS	POS	POS	POS	POS	POS
MSSA2028	NEG	NEG	NEG	NEG	POS	POS	NEG	POS	POS	POS	POS	POS	POS	POS	POS
MSSA2088	NEG	NEG	NEG	NEG	POS	POS	NEG	POS	POS	NEG	POS	POS	POS	POS	POS
MSSA2187	NEG	NEG	NEG	NEG	POS	POS	NEG	POS	NEG	POS	POS	POS	POS	POS	POS
MSSA2204	NEG	NEG	NEG	NEG	POS	POS	NEG	POS	POS	POS	POS	POS	POS	POS	POS
MSSA2210	NEG	NEG	NEG	NEG	POS	POS	NEG	POS	POS	POS	POS	POS	POS	POS	POS
MSSA2223	NEG	NEG	NEG	NEG	POS	POS	NEG	POS	POS	POS	POS	POS	POS	POS	POS
MSSA1763	NEG	NEG	NEG	NEG	POS	POS	NEG	NEG	NEG	NEG	POS	POS	POS	POS	POS
MSSA1777	NEG	NEG	NEG	NEG	POS	POS	NEG	NEG	NEG	NEG	POS	POS	POS	POS	POS
MSSA2009	NEG	NEG	NEG	NEG	POS	POS	NEG	NEG	NEG	NEG	POS	POS	POS	POS	POS
MSSA2047	NEG	NEG	NEG	NEG	POS	POS	NEG	NEG	NEG	NEG	POS	POS	POS	POS	POS
MSSA2079	NEG	NEG	NEG	NEG	POS	POS	NEG	NEG	NEG	NEG	POS	POS	POS	POS	POS
MSSA2101	NEG	NEG	NEG	NEG	POS	POS	NEG	NEG	NEG	NEG	POS	POS	POS	POS	POS
MSSA1821	NEG	NEG	NEG	NEG	POS	POS	NEG	POS	POS	POS	POS	POS	POS	POS	POS
MSSA1898	NEG	NEG	NEG	NEG	POS	POS	NEG	POS	POS	POS	POS	POS	POS	POS	POS
MSSA1973	NEG	NEG	NEG	NEG	POS	POS	NEG	POS	POS	POS	POS	POS	POS	POS	POS
MSSA2071	NEG	NEG	NEG	NEG	POS	POS	NEG	POS	POS	POS	POS	POS	POS	POS	POS
MSSA2083	NEG	NEG	NEG	NEG	POS	POS	NEG	POS	POS	POS	POS	POS	POS	POS	POS
MSSA2106	NEG	NEG	NEG	NEG	POS	POS	NEG	POS	POS	POS	POS	POS	POS	POS	POS
MSSA2112	NEG	NEG	NEG	NEG	POS	POS	NEG	POS	POS	POS	POS	POS	POS	POS	POS
MSSA2115	NEG	NEG	NEG	NEG	POS	POS	NEG	POS	POS	POS	POS	POS	POS	POS	POS
MSSA2195	NEG	NEG	NEG	NEG	POS	POS	NEG	POS	POS	POS	POS	POS	POS	POS	POS
MSSA2207	NEG	NEG	NEG	NEG	POS	POS	NEG	POS	POS	POS	POS	POS	POS	POS	POS
MSSA1758	NEG	NEG	NEG	NEG	POS	POS	NEG	POS	POS	POS	POS	POS	POS	POS	POS
MSSA1762	NEG	NEG	NEG	NEG	POS	POS	NEG	POS	POS	POS	POS	POS	POS	POS	POS
MSSA1769	NEG	NEG	NEG	NEG	POS	POS	NEG	POS	POS	POS	POS	POS	POS	POS	POS
MSSA1771	NEG	NEG	NEG	NEG	POS	POS	NEG	POS	POS	POS	POS	POS	POS	POS	POS
MSSA1789	NEG	NEG	NEG	NEG	POS	POS	NEG	POS	POS	POS	POS	POS	POS	POS	POS
MSSA1800	NEG	NEG	NEG	NEG	POS	POS	NEG	POS	POS	POS	POS	POS	POS	POS	POS
MSSA1833	NEG	NEG	NEG	NEG	POS	POS	NEG	POS	POS	POS	POS	POS	POS	POS	POS
MSSA1890	NEG	NEG	NEG	NEG	POS	POS	NEG	POS	POS	POS	POS	POS	POS	POS	POS
MSSA1903	NEG	NEG	NEG	NEG	POS	POS	NEG	POS	POS	POS	POS	POS	POS	POS	POS
MSSA1940	NEG	NEG	NEG	NEG	POS	POS	NEG	POS	POS	POS	POS	POS	POS	POS	POS
MSSA1962	NEG	NEG	NEG	NEG	POS	POS	NEG	POS	POS	POS	POS	POS	POS	POS	POS
MSSA1963	NEG	NEG	NEG	NEG	POS	POS	NEG	POS	POS	POS	POS	POS	POS	POS	POS
MSSA2016	NEG	NEG	NEG	NEG	POS	POS	NEG	POS	POS	POS	POS	POS	POS	POS	POS
MSSA2018	NEG	NEG	NEG	NEG	POS	POS	NEG	POS	POS	NEG	POS	POS	POS	POS	POS
MSSA2019	NEG	NEG	NEG	NEG	POS	POS	NEG	POS	POS	POS	POS	POS	POS	POS	POS
MSSA2029	NEG	NEG	NEG	NEG	POS	POS	NEG	POS	POS	POS	POS	POS	POS	POS	POS
MSSA2045	NEG	NEG	NEG	NEG	POS	POS	NEG	POS	POS	POS	POS	POS	POS	POS	POS
MSSA2048	NEG	NEG	NEG	NEG	POS	POS	NEG	POS	POS	POS	POS	POS	POS	POS	POS
MSSA2049	NEG	NEG	NEG	NEG	POS	POS	NEG	POS	POS	POS	POS	POS	POS	POS	POS
MSSA2050	NEG	NEG	NEG	NEG	POS	POS	NEG	POS	POS	POS	POS	POS	POS	POS	POS
MSSA2099	NEG	NEG	NEG	NEG	POS	POS	NEG	POS	POS	POS	POS	POS	POS	POS	POS
MSSA2109	NEG	NEG	NEG	NEG	POS	POS	NEG	POS	POS	POS	POS	POS	POS	POS	POS
MSSA2130	NEG	NEG	NEG	NEG	POS	POS	NEG	POS	POS	POS	POS	POS	POS	POS	POS
MSSA2133	NEG	NEG	NEG	NEG	POS	POS	NEG	POS	POS	POS	POS	POS	POS	POS	POS
MSSA2139	NEG	NEG	NEG	NEG	POS	POS	NEG	POS	POS	POS	POS	POS	POS	POS	POS
MSSA2141	NEG	NEG	NEG	NEG	POS	POS	NEG	POS	POS	POS	POS	POS	POS	POS	POS
MSSA2208	NEG	NEG	NEG	NEG	POS	POS	NEG	POS	POS	POS	POS	POS	POS	POS	POS
MSSA2209	NEG	NEG	NEG	NEG	POS	POS	NEG	POS	POS	NEG	POS	POS	POS	POS	POS
MSSA1893	NEG	NEG	NEG	NEG	POS	POS	NEG	POS	POS	NEG	POS	POS	POS	POS	POS
MSSA2105	NEG	NEG	NEG	NEG	POS	POS	NEG	POS	POS	NEG	POS	POS	POS	POS	POS
MRSA3836	NEG	NEG	NEG	NEG	POS	NEG	POS	NEG	NEG	NEG	POS	POS	POS	POS	POS
MRSA3840	NEG	NEG	NEG	NEG	POS	NEG	POS	NEG	NEG	NEG	POS	POS	POS	POS	POS
MRSA4188	NEG	NEG	NEG	NEG	POS	NEG	POS	NEG	NEG	NEG	POS	POS	POS	POS	POS
MSSA1814	NEG	NEG	NEG	NEG	POS	NEG	POS	NEG	NEG	NEG	POS	POS	POS	POS	POS
MSSA1816	NEG	NEG	NEG	NEG	POS	NEG	POS	NEG	NEG	NEG	POS	POS	AMB	POS	POS
MSSA1825	NEG	NEG	NEG	NEG	POS	NEG	POS	NEG	NEG	NEG	POS	POS	POS	POS	POS
MSSA1834	NEG	NEG	NEG	NEG	POS	NEG	POS	NEG	NEG	NEG	POS	POS	NEG	POS	POS
MSSA1938	NEG	NEG	NEG	NEG	POS	NEG	POS	NEG	NEG	NEG	POS	POS	POS	POS	POS
MSSA1948	NEG	NEG	NEG	NEG	POS	NEG	POS	NEG	NEG	NEG	POS	POS	POS	POS	POS
MSSA1951	NEG	NEG	NEG	NEG	POS	NEG	POS	NEG	NEG	NEG	POS	POS	POS	POS	POS
MSSA2005	NEG	NEG	NEG	NEG	POS	NEG	POS	NEG	NEG	NEG	POS	POS	POS	POS	POS
MSSA2051	NEG	NEG	NEG	NEG	POS	NEG	POS	NEG	NEG	NEG	POS	POS	POS	POS	POS
MSSA2056	NEG	NEG	NEG	NEG	POS	NEG	POS	NEG	NEG	NEG	POS	POS	POS	POS	POS
MSSA2066	NEG	NEG	NEG	NEG	POS	NEG	POS	NEG	NEG	NEG	POS	POS	POS	POS	POS
MSSA2067	NEG	NEG	NEG	NEG	POS	NEG	POS	NEG	NEG	NEG	POS	POS	POS	POS	POS
MSSA2086	NEG	NEG	NEG	NEG	POS	NEG	POS	NEG	NEG	NEG	POS	POS	POS	POS	POS
MSSA2089	NEG	NEG	NEG	NEG	POS	NEG	POS	NEG	NEG	NEG	POS	POS	POS	POS	POS
MSSA2103	NEG	NEG	NEG	NEG	POS	NEG	POS	NEG	NEG	NEG	POS	POS	AMB	POS	POS
MSSA2173	NEG	NEG	NEG	NEG	POS	NEG	POS	NEG	AMB	AMB	POS	POS	POS	POS	POS
MSSA2183	NEG	NEG	NEG	NEG	POS	NEG	POS	NEG	NEG	NEG	POS	POS	POS	POS	POS
MSSA2196	NEG	NEG	NEG	NEG	POS	NEG	POS	NEG	NEG	NEG	POS	POS	POS	POS	POS
MSSA2219	NEG	NEG	NEG	NEG	POS	NEG	POS	NEG	NEG	NEG	POS	POS	POS	POS	POS
MSSA1753	NEG	NEG	NEG	NEG	POS	POS	NEG	POS	POS	POS	POS	POS	POS	POS	POS
MSSA1773	NEG	NEG	NEG	NEG	POS	POS	NEG	POS	POS	POS	POS	POS	POS	POS	POS
MSSA1811	NEG	NEG	NEG	NEG	POS	POS	NEG	POS	POS	POS	POS	POS	POS	POS	POS
MSSA1897	NEG	NEG	NEG	NEG	POS	POS	NEG	POS	POS	POS	POS	POS	POS	POS	POS
MSSA1931	NEG	NEG	NEG	NEG	POS	POS	NEG	POS	POS	POS	POS	POS	POS	POS	POS
MSSA2114	NEG	NEG	NEG	NEG	POS	POS	NEG	POS	POS	POS	POS	POS	POS	POS	POS
MSSA1745	NEG	NEG	NEG	NEG	POS	NEG	POS	NEG	NEG	POS	POS	POS	POS	POS	POS
MSSA1747	NEG	NEG	NEG	NEG	POS	NEG	POS	NEG	NEG	POS	POS	POS	POS	POS	POS
MSSA1749	NEG	NEG	NEG	NEG	POS	NEG	POS	NEG	NEG	POS	POS	POS	POS	POS	POS
MSSA1768	NEG	NEG	NEG	NEG	POS	NEG	POS	NEG	NEG	POS	POS	POS	POS	POS	POS
MSSA1774	NEG	NEG	NEG	NEG	POS	NEG	POS	NEG	NEG	POS	POS	POS	POS	POS	POS
MSSA1775	NEG	NEG	NEG	NEG	POS	NEG	POS	NEG	NEG	POS	POS	POS	POS	POS	POS
MSSA1776	NEG	NEG	NEG	NEG	POS	NEG	POS	NEG	NEG	POS	POS	POS	POS	POS	POS
MSSA1779	NEG	NEG	NEG	NEG	POS	NEG	POS	NEG	NEG	POS	POS	POS	POS	POS	POS
MSSA1780	NEG	NEG	NEG	NEG	POS	NEG	POS	NEG	NEG	POS	POS	POS	POS	POS	POS

	VIRULENCE: ACME LOCUS				VIRULENCE: PROTEASES											
Strain	arcA-SCC	arcB-SCC	arcC-SCC	arcD-SCC	aur (cons)	aur (Other than MRSA252)	aur MRSA252	splA	splB	splE	sspA	sspB (probe 1)	sspB (probe 2)	sspP (probe 1)	sspP (probe 2)
MSSA1781	NEG	NEG	NEG	NEG	POS	NEG	POS	NEG	NEG	POS	POS	POS	POS	POS	POS
MSSA1786	NEG	NEG	NEG	NEG	POS	NEG	POS	NEG	NEG	POS	POS	POS	POS	POS	POS
MSSA1793	NEG	NEG	NEG	NEG	POS	NEG	POS	NEG	NEG	POS	POS	POS	POS	POS	POS
MSSA1803	NEG	NEG	NEG	NEG	POS	NEG	POS	NEG	NEG	POS	POS	POS	POS	POS	POS
MSSA1836	NEG	NEG	NEG	NEG	POS	NEG	POS	NEG	NEG	POS	POS	POS	POS	POS	POS
MSSA1839	NEG	NEG	NEG	NEG	POS	NEG	POS	NEG	NEG	POS	POS	POS	POS	POS	POS
MSSA1900	NEG	NEG	NEG	NEG	POS	NEG	POS	NEG	NEG	POS	POS	POS	POS	POS	POS
MSSA1910	NEG	NEG	NEG	NEG	POS	NEG	POS	NEG	NEG	POS	POS	POS	POS	POS	POS
MSSA1918	NEG	NEG	NEG	NEG	POS	NEG	POS	NEG	NEG	POS	POS	POS	POS	POS	POS
MSSA1921	NEG	NEG	NEG	NEG	POS	NEG	POS	NEG	NEG	POS	POS	POS	POS	POS	POS
MSSA1923	NEG	NEG	NEG	NEG	POS	NEG	POS	NEG	NEG	POS	POS	POS	POS	POS	POS
MSSA1981	NEG	NEG	NEG	NEG	POS	NEG	POS	NEG	NEG	POS	POS	POS	POS	POS	POS
MSSA1998	NEG	NEG	NEG	NEG	POS	NEG	POS	NEG	NEG	POS	POS	POS	POS	POS	POS
MSSA2000	NEG	NEG	NEG	NEG	POS	NEG	POS	NEG	NEG	POS	POS	POS	POS	POS	POS
MSSA2007	NEG	NEG	NEG	NEG	POS	NEG	POS	NEG	NEG	POS	POS	POS	POS	POS	POS
MSSA2012	NEG	NEG	NEG	NEG	POS	NEG	POS	NEG	NEG	POS	POS	POS	POS	POS	POS
MSSA2015	NEG	NEG	NEG	NEG	POS	NEG	POS	NEG	NEG	POS	POS	POS	POS	POS	POS
MSSA2021	NEG	NEG	NEG	NEG	POS	NEG	POS	NEG	NEG	POS	POS	POS	POS	POS	POS
MSSA2027	NEG	NEG	NEG	NEG	POS	NEG	POS	NEG	NEG	POS	POS	POS	POS	POS	POS
MSSA2063	NEG	NEG	NEG	NEG	POS	NEG	POS	NEG	NEG	POS	POS	POS	POS	POS	POS
MSSA2064	NEG	NEG	NEG	NEG	POS	NEG	POS	NEG	NEG	POS	POS	POS	POS	POS	POS
MSSA2065	NEG	NEG	NEG	NEG	POS	NEG	POS	NEG	NEG	POS	POS	POS	POS	POS	POS
MSSA2075	NEG	NEG	NEG	NEG	POS	NEG	POS	NEG	NEG	POS	POS	POS	POS	POS	POS
MSSA2093	NEG	NEG	NEG	NEG	POS	NEG	POS	NEG	NEG	POS	POS	POS	POS	POS	POS
MSSA2102	NEG	NEG	NEG	NEG	POS	NEG	POS	NEG	NEG	POS	POS	POS	POS	POS	POS
MSSA2138	NEG	NEG	NEG	NEG	POS	NEG	POS	NEG	NEG	POS	POS	POS	POS	POS	POS
MSSA2168	NEG	NEG	NEG	NEG	POS	NEG	POS	NEG	NEG	POS	POS	POS	POS	POS	POS
MSSA2181	NEG	NEG	NEG	NEG	POS	NEG	POS	NEG	NEG	POS	POS	POS	POS	POS	POS
MSSA2189	NEG	NEG	NEG	NEG	POS	NEG	POS	NEG	NEG	POS	POS	POS	POS	POS	POS
MSSA2214	NEG	NEG	NEG	NEG	POS	NEG	POS	NEG	NEG	POS	POS	POS	POS	POS	POS
MRSA3803	NEG	NEG	NEG	NEG	POS	NEG	POS	NEG	NEG	NEG	POS	POS	POS	POS	POS
MSSA1750	NEG	NEG	NEG	NEG	POS	NEG	POS	NEG	NEG	NEG	POS	POS	POS	POS	POS
MSSA1756	NEG	NEG	NEG	NEG	POS	NEG	POS	NEG	NEG	NEG	POS	POS	POS	POS	POS
MSSA1759	NEG	NEG	NEG	NEG	AMB	NEG	POS	NEG	NEG	NEG	POS	POS	POS	POS	POS
MSSA1767	NEG	NEG	NEG	NEG	POS	NEG	POS	NEG	NEG	NEG	POS	POS	POS	POS	POS
MSSA1787	NEG	NEG	NEG	NEG	POS	NEG	POS	NEG	NEG	NEG	POS	POS	POS	POS	POS
MSSA1790	NEG	NEG	NEG	NEG	POS	NEG	POS	NEG	NEG	NEG	POS	POS	POS	POS	POS
MSSA1794	NEG	NEG	NEG	NEG	NEG	NEG	POS	NEG	NEG	NEG	POS	POS	POS	POS	POS
MSSA1808	NEG	NEG	NEG	NEG	AMB	NEG	POS	NEG	NEG	NEG	POS	POS	POS	POS	POS
MSSA1812	NEG	NEG	NEG	NEG	AMB	NEG	POS	NEG	NEG	NEG	POS	POS	POS	POS	POS
MSSA1819	NEG	NEG	NEG	NEG	AMB	NEG	POS	NEG	NEG	NEG	POS	POS	POS	POS	POS
MSSA1824	NEG	NEG	NEG	NEG	NEG	NEG	POS	NEG	NEG	NEG	POS	POS	POS	POS	POS
MSSA1827	NEG	NEG	NEG	NEG	AMB	NEG	POS	NEG	NEG	NEG	POS	POS	POS	POS	POS
MSSA1889	NEG	NEG	NEG	NEG	NEG	NEG	POS	NEG	NEG	NEG	POS	POS	POS	POS	POS
MSSA1895	NEG	NEG	NEG	NEG	NEG	NEG	POS	NEG	NEG	NEG	POS	POS	POS	POS	POS
MSSA1904	NEG	NEG	NEG	NEG	NEG	NEG	POS	NEG	NEG	NEG	POS	POS	POS	POS	POS
MSSA1905	NEG	NEG	NEG	NEG	NEG	NEG	POS	NEG	NEG	NEG	POS	POS	POS	POS	POS
MSSA1907	NEG	NEG	NEG	NEG	NEG	NEG	POS	NEG	NEG	NEG	POS	POS	POS	POS	POS
MSSA1919	NEG	NEG	NEG	NEG	NEG	NEG	POS	NEG	NEG	NEG	POS	POS	POS	POS	POS
MSSA1920	NEG	NEG	NEG	NEG	AMB	NEG	POS	NEG	NEG	NEG	POS	POS	POS	POS	POS
MSSA1929	NEG	NEG	NEG	NEG	AMB	NEG	POS	NEG	NEG	NEG	POS	POS	POS	POS	POS
MSSA1930	NEG	NEG	NEG	NEG	AMB	NEG	POS	NEG	NEG	NEG	POS	POS	POS	POS	POS
MSSA1934	NEG	NEG	NEG	NEG	AMB	NEG	POS	NEG	NEG	NEG	POS	POS	POS	POS	POS
MSSA1946	NEG	NEG	NEG	NEG	AMB	NEG	POS	NEG	NEG	NEG	POS	POS	POS	POS	POS
MSSA1971	NEG	NEG	NEG	NEG	AMB	NEG	POS	NEG	NEG	NEG	POS	POS	POS	POS	POS
MSSA1980	NEG	NEG	NEG	NEG	NEG	NEG	POS	NEG	NEG	NEG	POS	POS	POS	POS	POS
MSSA1982	NEG	NEG	NEG	NEG	AMB	NEG	POS	NEG	NEG	NEG	POS	POS	POS	POS	POS
MSSA1996	NEG	NEG	NEG	NEG	AMB	NEG	POS	NEG	NEG	NEG	POS	POS	POS	POS	POS
MSSA2002	NEG	NEG	NEG	NEG	AMB	NEG	POS	NEG	NEG	NEG	POS	POS	POS	POS	POS
MSSA2014	NEG	NEG	NEG	NEG	AMB	NEG	POS	NEG	NEG	NEG	POS	POS	POS	POS	POS
MSSA2053	NEG	NEG	NEG	NEG	AMB	NEG	POS	NEG	NEG	NEG	POS	POS	POS	POS	POS
MSSA2054	NEG	NEG	NEG	NEG	AMB	NEG	POS	NEG	NEG	NEG	POS	POS	POS	POS	POS
MSSA2058	NEG	NEG	NEG	NEG	NEG	NEG	POS	NEG	NEG	NEG	POS	POS	POS	POS	POS
MSSA2068	NEG	NEG	NEG	NEG	POS	NEG	POS	NEG	NEG	NEG	POS	POS	POS	POS	POS
MSSA2070	NEG	NEG	NEG	NEG	AMB	NEG	POS	NEG	NEG	NEG	POS	POS	POS	POS	POS
MSSA2072	NEG	NEG	NEG	NEG	AMB	NEG	POS	NEG	NEG	NEG	POS	POS	POS	POS	POS
MSSA2073	NEG	NEG	NEG	NEG	NEG	NEG	POS	NEG	NEG	NEG	POS	POS	POS	POS	POS
MSSA2074	NEG	NEG	NEG	NEG	AMB	NEG	POS	NEG	NEG	NEG	POS	POS	POS	POS	POS
MSSA2107	NEG	NEG	NEG	NEG	POS	NEG	POS	NEG	NEG	NEG	POS	POS	POS	POS	POS
MSSA2108	NEG	NEG	NEG	NEG	NEG	NEG	POS	NEG	NEG	NEG	POS	POS	POS	POS	POS
MSSA2129	NEG	NEG	NEG	NEG	AMB	NEG	POS	NEG	NEG	NEG	POS	POS	POS	POS	POS
MSSA2135	NEG	NEG	NEG	NEG	POS	NEG	POS	NEG	NEG	NEG	POS	POS	POS	POS	POS
MSSA2137	NEG	NEG	NEG	NEG	POS	NEG	POS	NEG	NEG	NEG	POS	POS	POS	POS	POS
MSSA2140	NEG	NEG	NEG	NEG	POS	NEG	POS	NEG	NEG	NEG	POS	POS	POS	POS	POS
MSSA2182	NEG	NEG	NEG	NEG	POS	NEG	POS	NEG	NEG	NEG	POS	POS	POS	POS	POS
MSSA2188	NEG	NEG	NEG	NEG	POS	NEG	POS	NEG	NEG	NEG	POS	POS	POS	POS	POS
MSSA1899	NEG	NEG	NEG	NEG	POS	AMB	NEG	NEG	NEG	NEG	POS	POS	POS	POS	POS
MSSA1915	NEG	NEG	NEG	NEG	POS	NEG	NEG	NEG	NEG	NEG	POS	POS	POS	POS	POS
MSSA1949	NEG	NEG	NEG	NEG	POS	NEG	NEG	NEG	NEG	NEG	POS	POS	POS	POS	POS
MSSA2042	NEG	NEG	NEG	NEG	POS	NEG	NEG	NEG	NEG	NEG	POS	POS	POS	POS	POS
MSSA2077	NEG	NEG	NEG	NEG	POS	NEG	NEG	NEG	NEG	NEG	POS	POS	POS	POS	POS
MSSA2095	NEG	NEG	NEG	NEG	POS	AMB	NEG	NEG	NEG	NEG	POS	POS	POS	POS	POS
MSSA1997	NEG	NEG	NEG	NEG	POS	POS	NEG	POS	POS	NEG	POS	POS	POS	POS	POS
MSSA1810	NEG	NEG	NEG	NEG	POS	POS	NEG	NEG	POS	POS	POS	POS	POS	POS	POS
MSSA1832	NEG	NEG	NEG	NEG	POS	POS	NEG	POS	POS	POS	POS	POS	POS	POS	POS
MSSA1891	NEG	NEG	NEG	NEG	POS	POS	NEG	POS	POS	POS	POS	POS	POS	POS	POS
MSSA1892	NEG	NEG	NEG	NEG	POS	POS	NEG	POS	POS	POS	POS	POS	POS	POS	POS
MSSA1932	NEG	NEG	NEG	NEG	POS	POS	NEG	POS	POS	POS	POS	POS	POS	POS	POS
MSSA1942	NEG	NEG	NEG	NEG	POS	POS	NEG	POS	POS	POS	POS	POS	POS	POS	POS
MSSA1957	NEG	NEG	NEG	NEG	POS	POS	NEG	POS	POS	POS	POS	POS	POS	POS	POS
MSSA1748	NEG	NEG	NEG	NEG	POS	POS	NEG	POS	POS	POS	POS	POS	POS	POS	POS
MSSA1754	NEG	NEG	NEG	NEG	POS	POS	NEG	POS	POS	POS	POS	POS	POS	POS	POS
MSSA1770	NEG	NEG	NEG	NEG	POS	POS	NEG	POS	POS	POS	POS	POS	POS	POS	POS
MSSA1788	NEG	NEG	NEG	NEG	POS	POS	NEG	POS	POS	POS	POS	POS	POS	POS	POS
MSSA1804	NEG	NEG	NEG	NEG	POS	POS	NEG	POS	POS	POS	POS	POS	POS	POS	POS
MSSA1818	NEG	NEG	NEG	NEG	POS	POS	NEG	POS	POS	POS	POS	POS	POS	POS	POS
MSSA1914	NEG	NEG	NEG	NEG	POS	POS	NEG	POS	POS	POS	POS	POS	POS	POS	POS
MSSA1974	NEG	NEG	NEG	NEG	POS	POS	NEG	POS	POS	POS	POS	POS	POS	POS	POS
MSSA2023	NEG	NEG	NEG	NEG	POS	POS	NEG	POS	POS	POS	POS	POS	POS	POS	POS
MSSA2030	NEG	NEG	NEG	NEG	POS	POS	NEG	POS	POS	POS	POS	POS	POS	POS	POS
MSSA2096	NEG	NEG	NEG	NEG	POS	POS	NEG	AMB	NEG	POS	POS	POS	POS	POS	POS
MSSA2142	NEG	NEG	NEG	NEG	POS	POS	NEG	POS	POS	POS	POS	POS	POS	POS	POS
MSSA2203	NEG	NEG	NEG	NEG	POS	POS	NEG	POS	POS	POS	POS	POS	POS	POS	POS
MSSA1901	NEG	NEG	NEG	NEG	POS	POS	NEG	POS	POS	NEG	POS	POS	POS	POS	POS
MSSA2013	NEG	NEG	NEG	NEG	POS	POS	NEG	POS	POS	NEG	POS	POS	POS	POS	POS
MSSA2031	NEG	NEG	NEG	NEG	POS	POS	NEG	POS	POS	NEG	POS	POS	POS	POS	POS
MSSA2052	NEG	NEG	NEG	NEG	POS	POS	NEG	POS	POS	AMB	POS	POS	POS	POS	POS
MSSA2057	NEG	NEG	NEG	NEG	POS	POS	NEG	POS	POS	NEG	POS	POS	POS	POS	POS
MSSA2081	NEG	NEG	NEG	NEG	POS	POS	NEG	POS	POS	POS	POS	POS	POS	POS	POS
MSSA1955	NEG	NEG	NEG	NEG	POS	POS	NEG	NEG	NEG	NEG	POS	POS	POS	POS	POS
MSSA1941	NEG	NEG	NEG	NEG	POS	NEG	POS	POS	POS	POS	POS	POS	POS	POS	POS
MRSA3838	NEG	NEG	NEG	NEG	POS	NEG	POS	NEG	NEG	NEG	POS	POS	POS	POS	POS
MRSA3839	NEG	NEG	NEG	NEG	POS	NEG	POS	NEG	NEG	NEG	POS	POS	POS	POS	POS
MSSA1801	NEG	NEG	NEG	NEG	POS	NEG	POS	POS	NEG	POS	POS	POS	POS	POS	POS
MSSA2131	NEG	NEG	NEG	NEG	POS	NEG	POS	NEG	NEG	NEG	POS	POS	POS	POS	POS
MSSA1792	NEG	NEG	NEG	NEG	POS	POS	NEG	POS	POS	POS	POS	POS	POS	POS	POS
MSSA1796	NEG	NEG	NEG	AMB	POS	POS	NEG	POS	POS	POS	POS	POS	POS	POS	POS
MSSA1817	NEG	NEG	NEG	NEG	POS	POS	NEG	POS	POS	POS	POS	POS	POS	POS	POS
MSSA1911	NEG	NEG	NEG	NEG	NEG	NEG	NEG	NEG	NEG	NEG	NEG	NEG	NEG	NEG	NEG
MSSA1927	NEG	NEG	NEG	NEG	POS	POS	NEG	POS	POS	POS	POS	POS	POS	POS	POS
MSSA1928	NEG	NEG	NEG	NEG	POS	POS	NEG	POS	POS	POS	POS	POS	POS	POS	POS
MSSA1953	NEG	NEG	NEG	NEG	POS	POS	NEG	POS	POS	POS	POS	POS	POS	POS	POS
MSSA2062	NEG	NEG	NEG	NEG	POS	POS	NEG	POS	POS	POS	POS	POS	POS	POS	POS
MSSA2078	NEG	NEG	NEG	NEG	POS	NEG	POS	POS	POS	NEG	POS	POS	POS	POS	POS
MSSA2136	NEG	NEG	NEG	NEG	POS	POS	NEG	NEG	NEG	NEG	POS	POS	POS	POS	POS

	VIRULENCE : STAPHYLOCOCCAL SUPERANTIGEN/ENTEROTOXIN-LIKE GENES (SET/SSL)											
Strain	setC	ssl1/set6 probe 1_11	ssl1/set6 probe 2_11	ssl1/set6 probe 1_12	ssl1/set6 probe 2_12	ssl1/set6 probe 4_11	ssl01-RF122	ssl02/set7	ssl02/set7 (MRSA252)	ssl03/set8 probe 1	ssl03/set8 probe 2	ssl03/set8 (MRSA252, SAR0424)
MSSA1805	POS	NEG	POS	NEG	POS	NEG	NEG	POS	NEG	POS	POS	NEG
MSSA1823	POS	NEG	POS	NEG	POS	NEG	NEG	POS	NEG	POS	POS	NEG
MSSA1837	POS	NEG	POS	NEG	POS	NEG	NEG	POS	NEG	POS	POS	NEG
MSSA2044	POS	NEG	POS	NEG	POS	NEG	NEG	POS	NEG	POS	POS	NEG
MSSA2046	POS	NEG	POS	NEG	POS	NEG	NEG	POS	NEG	POS	POS	NEG
MSSA2059	POS	NEG	POS	NEG	POS	NEG	NEG	POS	NEG	POS	POS	NEG
MSSA2076	POS	NEG	POS	NEG	POS	NEG	NEG	POS	NEG	POS	POS	NEG
MSSA2200	POS	NEG	POS	NEG	POS	NEG	NEG	POS	NEG	POS	POS	NEG
MRSA3797	POS	POS	NEG	POS	NEG	POS	NEG	POS	NEG	POS	POS	NEG
MRSA3798	POS	POS	AMB	POS	NEG	POS	NEG	POS	AMB	POS	POS	NEG
MRSA3799	POS	POS	NEG	POS	NEG	POS	NEG	POS	NEG	POS	POS	NEG
MRSA3800	POS	POS	AMB	POS	NEG	POS	NEG	POS	AMB	POS	POS	NEG
MRSA3801	POS	POS	POS	POS	NEG	POS	NEG	POS	AMB	POS	POS	NEG
MRSA3804	POS	POS	AMB	POS	NEG	POS	NEG	POS	AMB	POS	POS	NEG
MRSA3806	POS	POS	NEG	POS	NEG	POS	NEG	POS	NEG	POS	POS	NEG
MRSA3961	POS	POS	NEG	POS	NEG	POS	NEG	POS	NEG	POS	POS	NEG
MRSA3962	POS	POS	NEG	POS	NEG	POS	NEG	POS	NEG	POS	POS	NEG
MRSA3963	POS	POS	NEG	POS	NEG	POS	NEG	POS	NEG	POS	POS	NEG
MRSA3966	POS	POS	NEG	POS	NEG	POS	NEG	POS	NEG	POS	POS	NEG
MRSA3978	POS	POS	NEG	POS	NEG	POS	NEG	POS	NEG	POS	POS	NEG
MRSA3979	POS	POS	NEG	POS	NEG	AMB	NEG	POS	NEG	POS	POS	NEG
MRSA3980	POS	POS	NEG	POS	NEG	AMB	NEG	POS	NEG	POS	POS	NEG
MRSA4179	POS	POS	NEG	POS	NEG	POS	NEG	POS	NEG	POS	POS	NEG
MRSA4216	POS	POS	NEG	POS	NEG	POS	NEG	POS	NEG	POS	POS	NEG
MRSA4217	POS	POS	NEG	POS	NEG	POS	NEG	POS	NEG	POS	POS	NEG
MRSA4220	POS	POS	NEG	POS	NEG	POS	NEG	POS	NEG	POS	POS	NEG
MRSA4221	POS	POS	NEG	POS	NEG	POS	NEG	POS	NEG	POS	POS	NEG
MRSA4222	POS	POS	NEG	POS	NEG	POS	NEG	POS	NEG	POS	POS	NEG
MSSA1751	POS	POS	AMB	POS	NEG	POS	NEG	POS	AMB	POS	POS	NEG
MSSA1760	POS	POS	AMB	POS	NEG	POS	NEG	POS	AMB	POS	POS	NEG
MSSA1802	POS	POS	NEG	POS	NEG	POS	NEG	POS	AMB	POS	POS	NEG
MSSA1809	POS	POS	NEG	POS	NEG	POS	NEG	POS	AMB	POS	POS	NEG
MSSA1815	POS	POS	NEG	POS	NEG	POS	NEG	POS	AMB	POS	POS	NEG
MSSA1829	POS	POS	NEG	POS	NEG	POS	NEG	POS	AMB	POS	POS	NEG
MSSA1908	POS	POS	NEG	POS	NEG	POS	NEG	POS	NEG	POS	POS	NEG
MSSA1960	POS	POS	NEG	POS	NEG	POS	NEG	POS	NEG	POS	POS	NEG
MSSA1965	POS	POS	NEG	POS	NEG	POS	NEG	POS	NEG	POS	POS	NEG
MSSA1977	POS	POS	NEG	POS	NEG	POS	NEG	POS	NEG	POS	POS	NEG
MSSA1978	POS	POS	AMB	POS	NEG	POS	NEG	POS	AMB	POS	POS	NEG
MSSA1999	POS	POS	NEG	POS	NEG	POS	NEG	POS	NEG	POS	POS	NEG
MSSA2003	POS	POS	NEG	POS	NEG	POS	NEG	POS	NEG	POS	POS	NEG
MSSA2006	POS	POS	NEG	POS	NEG	POS	NEG	POS	NEG	POS	POS	NEG
MSSA2008	POS	POS	NEG	POS	NEG	POS	NEG	POS	NEG	POS	POS	NEG
MSSA2011	POS	POS	NEG	POS	NEG	POS	NEG	POS	NEG	POS	POS	NEG
MSSA2017	POS	POS	NEG	POS	NEG	AMB	NEG	POS	NEG	POS	POS	NEG
MSSA2022	POS	POS	NEG	POS	NEG	POS	NEG	POS	NEG	POS	POS	NEG
MSSA2055	POS	POS	NEG	POS	NEG	POS	NEG	POS	NEG	POS	POS	NEG
MSSA2060	POS	POS	NEG	POS	NEG	POS	NEG	POS	AMB	POS	POS	NEG
MSSA2080	POS	POS	NEG	POS	NEG	AMB	NEG	POS	NEG	POS	POS	NEG
MSSA2082	POS	POS	AMB	POS	NEG	POS	NEG	POS	AMB	POS	POS	NEG
MSSA2084	POS	POS	NEG	POS	NEG	AMB	NEG	POS	NEG	POS	POS	NEG
MSSA2087	POS	POS	NEG	POS	NEG	AMB	NEG	POS	NEG	POS	POS	NEG
MSSA2094	POS	POS	NEG	POS	NEG	POS	NEG	POS	NEG	POS	POS	NEG
MSSA2097	POS	POS	NEG	POS	NEG	POS	NEG	POS	NEG	POS	POS	NEG
MSSA2110	POS	POS	NEG	POS	NEG	POS	NEG	POS	NEG	POS	POS	NEG
MSSA2113	POS	POS	NEG	POS	NEG	POS	NEG	POS	NEG	POS	POS	NEG
MSSA2132	POS	POS	NEG	POS	NEG	POS	NEG	POS	NEG	POS	POS	NEG
MSSA2134	POS	POS	NEG	POS	NEG	POS	NEG	POS	NEG	POS	POS	NEG
MSSA1746	POS	POS	NEG	NEG	POS	NEG	NEG	POS	NEG	POS	POS	NEG
MSSA1757	POS	POS	NEG	NEG	POS	NEG	NEG	POS	AMB	POS	POS	NEG
MSSA1772	POS	POS	NEG	NEG	POS	NEG	NEG	POS	NEG	POS	POS	NEG
MSSA1795	POS	POS	NEG	NEG	POS	NEG	NEG	POS	NEG	POS	POS	NEG
MSSA1813	POS	POS	NEG	NEG	POS	NEG	NEG	POS	NEG	POS	POS	NEG
MSSA1826	POS	POS	NEG	NEG	POS	NEG	NEG	POS	NEG	POS	POS	NEG
MSSA1830	POS	POS	NEG	NEG	POS	NEG	NEG	POS	NEG	POS	POS	NEG
MSSA1838	POS	POS	NEG	NEG	POS	NEG	NEG	POS	AMB	POS	POS	NEG
MSSA1944	POS	POS	NEG	NEG	POS	NEG	NEG	POS	NEG	POS	POS	NEG
MSSA1947	POS	POS	NEG	NEG	POS	NEG	NEG	POS	NEG	POS	POS	NEG
MSSA1964	POS	POS	NEG	NEG	POS	NEG	NEG	POS	NEG	POS	POS	NEG
MSSA1969	POS	POS	NEG	NEG	POS	NEG	NEG	POS	NEG	POS	POS	NEG
MSSA2001	POS	POS	NEG	NEG	POS	NEG	NEG	POS	NEG	POS	POS	NEG
MSSA2004	POS	POS	NEG	NEG	POS	NEG	NEG	POS	NEG	POS	POS	NEG
MSSA2024	POS	POS	NEG	NEG	NEG	NEG	NEG	POS	NEG	AMB	AMB	NEG
MSSA2025	POS	POS	NEG	NEG	POS	NEG	NEG	POS	NEG	POS	POS	NEG
MSSA2026	POS	POS	NEG	NEG	POS	NEG	NEG	POS	NEG	POS	POS	NEG
MSSA2032	POS	POS	NEG	NEG	POS	NEG	NEG	POS	NEG	POS	POS	NEG
MSSA2043	POS	POS	NEG	NEG	POS	NEG	NEG	POS	NEG	POS	POS	NEG
MSSA2061	POS	POS	NEG	NEG	POS	NEG	NEG	POS	NEG	POS	POS	NEG
MSSA2085	POS	POS	NEG	NEG	POS	NEG	NEG	POS	NEG	POS	POS	NEG
MSSA2098	POS	POS	NEG	NEG	POS	NEG	NEG	POS	NEG	POS	POS	NEG
MSSA2100	POS	POS	NEG	NEG	POS	NEG	NEG	POS	NEG	POS	POS	NEG
MSSA2111	POS	POS	NEG	NEG	POS	NEG	NEG	POS	NEG	POS	POS	NEG
MSSA2178	POS	POS	NEG	NEG	POS	NEG	NEG	POS	NEG	POS	POS	NEG
MSSA2202	POS	POS	NEG	NEG	POS	NEG	NEG	POS	NEG	POS	POS	NEG
MSSA2205	POS	POS	NEG	NEG	POS	NEG	NEG	POS	NEG	POS	POS	NEG
MRSA3796	POS	POS	NEG	POS	NEG	POS	NEG	POS	AMB	POS	POS	NEG
MRSA3805	POS	POS	NEG	AMB	NEG	POS	NEG	POS	NEG	POS	POS	NEG
MRSA3965	POS	POS	NEG	NEG	NEG	POS	NEG	POS	NEG	POS	POS	NEG
MSSA1761	POS	POS	NEG	POS	NEG	POS	NEG	POS	AMB	POS	POS	NEG
MSSA1764	POS	POS	NEG	NEG	NEG	POS	NEG	POS	NEG	POS	POS	NEG
MSSA1765	POS	POS	NEG	NEG	NEG	POS	NEG	POS	NEG	POS	POS	NEG
MSSA1784	POS	POS	NEG	POS	NEG	POS	NEG	POS	AMB	POS	POS	NEG
MSSA1806	POS	POS	NEG	AMB	NEG	POS	NEG	POS	NEG	POS	POS	NEG
MSSA1807	POS	POS	NEG	POS	NEG	POS	NEG	POS	AMB	POS	POS	NEG
MSSA1888	POS	POS	NEG	NEG	NEG	POS	NEG	POS	NEG	POS	POS	NEG
MSSA1894	POS	POS	NEG	NEG	NEG	POS	NEG	POS	NEG	POS	POS	NEG
MSSA1922	POS	POS	NEG	NEG	NEG	POS	NEG	POS	NEG	POS	POS	NEG
MSSA1950	POS	POS	NEG	AMB	NEG	POS	NEG	POS	NEG	POS	POS	NEG
MSSA1954	POS	POS	NEG	AMB	NEG	POS	NEG	POS	NEG	POS	POS	NEG
MSSA1966	POS	POS	NEG	AMB	NEG	POS	NEG	POS	NEG	POS	POS	NEG
MSSA1972	POS	POS	NEG	NEG	NEG	POS	NEG	POS	NEG	POS	POS	NEG
MSSA2010	POS	POS	NEG	NEG	NEG	POS	NEG	POS	NEG	POS	POS	NEG
MSSA2020	POS	POS	NEG	NEG	NEG	POS	NEG	POS	NEG	POS	POS	NEG
MSSA2028	POS	POS	NEG	POS	NEG	POS	NEG	POS	AMB	POS	POS	NEG
MSSA2088	POS	POS	NEG	POS	NEG	POS	NEG	POS	AMB	POS	POS	NEG
MSSA2187	POS	POS	NEG	POS	NEG	POS	NEG	POS	NEG	POS	POS	NEG
MSSA2204	POS	POS	NEG	POS	NEG	POS	NEG	POS	NEG	POS	POS	NEG
MSSA2210	POS	POS	NEG	NEG	NEG	POS	NEG	POS	NEG	POS	POS	NEG
MSSA2223	POS	POS	NEG	AMB	NEG	POS	NEG	POS	NEG	POS	POS	NEG
MSSA1763	POS	NEG	NEG	NEG	POS	NEG	POS	POS	NEG	NEG	AMB	NEG
MSSA1777	POS	AMB	POS	NEG	POS	NEG	POS	POS	NEG	POS	POS	NEG
MSSA2009	POS	NEG	AMB	NEG	POS	NEG	POS	POS	NEG	AMB	AMB	NEG
MSSA2047	POS	NEG	AMB	NEG	POS	NEG	POS	POS	NEG	NEG	AMB	NEG
MSSA2079	POS	NEG	NEG	NEG	POS	NEG	POS	POS	NEG	NEG	NEG	NEG
MSSA2101	POS	NEG	AMB	NEG	POS	NEG	POS	POS	NEG	AMB	AMB	NEG
MSSA1821	POS	NEG	NEG	AMB	NEG	POS	POS	POS	NEG	POS	POS	NEG
MSSA1898	POS	NEG	NEG	AMB	NEG	POS	POS	POS	NEG	POS	POS	NEG
MSSA1973	POS	NEG	NEG	NEG	NEG	POS	POS	POS	NEG	NEG	NEG	NEG
MSSA2071	POS	NEG	NEG	NEG	NEG	POS	POS	POS	NEG	POS	POS	NEG
MSSA2083	POS	NEG	NEG	NEG	NEG	POS	POS	POS	NEG	POS	POS	NEG
MSSA2106	POS	NEG	NEG	NEG	NEG	POS	POS	POS	NEG	POS	POS	NEG
MSSA2112	POS	NEG	NEG	NEG	NEG	POS	POS	POS	NEG	POS	POS	NEG
MSSA2115	POS	NEG	NEG	AMB	NEG	POS	POS	POS	NEG	POS	POS	NEG
MSSA2195	POS	NEG	NEG	AMB	NEG	POS	POS	POS	NEG	POS	POS	NEG
MSSA2207	POS	NEG	NEG	AMB	NEG	POS	POS	POS	NEG	NEG	NEG	NEG
MSSA1758	POS	NEG	NEG	NEG	NEG	POS	NEG	POS	NEG	POS	POS	NEG
MSSA1762	POS	NEG	POS	NEG	NEG	POS	NEG	POS	AMB	POS	POS	NEG
MSSA1769	POS	NEG	POS	NEG	NEG	POS	AMB	POS	AMB	POS	POS	NEG
MSSA1771	POS	NEG	NEG	NEG	NEG	POS	NEG	POS	NEG	POS	POS	NEG
MSSA1789	POS	NEG	NEG	NEG	NEG	POS	NEG	POS	NEG	POS	POS	NEG
MSSA1800	POS	NEG	AMB	NEG	NEG	POS	NEG	POS	NEG	POS	POS	NEG
MSSA1833	POS	NEG	POS	NEG	NEG	POS	NEG	POS	AMB	POS	POS	NEG
MSSA1890	POS	NEG	NEG	NEG	NEG	POS	NEG	POS	NEG	POS	POS	NEG
MSSA1903	POS	NEG	AMB	NEG	NEG	POS	NEG	POS	NEG	POS	POS	NEG
MSSA1940	POS	NEG	AMB	NEG	NEG	POS	NEG	POS	NEG	POS	POS	NEG
MSSA1962	POS	NEG	NEG	NEG	NEG	POS	NEG	POS	NEG	POS	POS	NEG
MSSA1963	POS	NEG	POS	NEG	NEG	POS	NEG	POS	NEG	POS	POS	NEG
MSSA2016	POS	NEG	NEG	NEG	NEG	POS	NEG	POS	NEG	POS	POS	NEG
MSSA2018	POS	NEG	AMB	NEG	NEG	POS	NEG	POS	NEG	POS	POS	NEG
MSSA2019	POS	NEG	NEG	NEG	NEG	POS	NEG	POS	NEG	POS	POS	NEG
MSSA2029	POS	NEG	POS	NEG	NEG	POS	AMB	POS	NEG	POS	POS	NEG
MSSA2045	POS	NEG	AMB	NEG	NEG	POS	NEG	POS	NEG	POS	POS	NEG
MSSA2048	POS	NEG	AMB	NEG	NEG	POS	NEG	POS	NEG	POS	POS	NEG
MSSA2049	POS	NEG	AMB	NEG	NEG	POS	NEG	POS	NEG	POS	POS	NEG
MSSA2050	POS	NEG	POS	NEG	NEG	POS	AMB	POS	AMB	POS	POS	NEG
MSSA2099	POS	NEG	NEG	NEG	NEG	AMB	NEG	POS	NEG	POS	POS	NEG
MSSA2109	POS	NEG	NEG	NEG	NEG	POS	NEG	POS	NEG	POS	POS	NEG
MSSA2130	POS	NEG	AMB	NEG	NEG	POS	NEG	NEG	NEG	POS	POS	NEG
MSSA2133	POS	NEG	AMB	NEG	NEG	POS	NEG	POS	NEG	POS	POS	NEG
MSSA2139	POS	NEG	AMB	NEG	NEG	POS	NEG	POS	NEG	POS	POS	NEG
MSSA2141	POS	NEG	POS	NEG	NEG	POS	AMB	POS	AMB	POS	POS	NEG
MSSA2208	POS	NEG	AMB	NEG	NEG	POS	NEG	POS	NEG	POS	POS	NEG
MSSA2209	POS	NEG	AMB	NEG	NEG	POS	NEG	POS	NEG	POS	POS	NEG
MSSA1893	POS	NEG	NEG	NEG	POS	NEG	POS	POS	NEG	NEG	NEG	NEG
MSSA2105	POS	NEG	AMB	NEG	POS	NEG	POS	POS	NEG	NEG	NEG	NEG
MRSA3836	POS	NEG	POS	POS	NEG	NEG	NEG	AMB	POS	NEG	NEG	NEG
MRSA3840	POS	NEG	POS	POS	NEG	NEG	NEG	AMB	POS	NEG	NEG	NEG
MRSA4188	POS	NEG	POS	POS	NEG	NEG	NEG	AMB	POS	POS	NEG	AMB
MSSA1814	POS	NEG	POS	POS	NEG	NEG	NEG	AMB	POS	NEG	NEG	NEG
MSSA1816	POS	NEG	POS	NEG	NEG	NEG	NEG	POS	AMB	NEG	NEG	NEG
MSSA1825	POS	NEG	POS	POS	NEG	NEG	NEG	AMB	POS	NEG	NEG	NEG
MSSA1834	POS	NEG	POS	NEG	NEG	NEG	NEG	POS	AMB	NEG	NEG	NEG
MSSA1938	POS	NEG	POS	POS	NEG	NEG	NEG	AMB	POS	NEG	NEG	NEG
MSSA1948	POS	NEG	POS	POS	NEG	NEG	NEG	AMB	POS	NEG	NEG	NEG
MSSA1951	POS	NEG	POS	NEG	NEG	NEG	NEG	POS	AMB	NEG	NEG	NEG
MSSA2005	POS	NEG	POS	POS	NEG	NEG	NEG	AMB	POS	NEG	NEG	NEG
MSSA2051	POS	NEG	POS	POS	NEG	NEG	NEG	AMB	POS	NEG	NEG	AMB
MSSA2056	POS	NEG	POS	POS	NEG	NEG	NEG	AMB	POS	NEG	NEG	NEG
MSSA2066	POS	NEG	POS	POS	NEG	NEG	NEG	AMB	POS	NEG	NEG	NEG
MSSA2067	POS	NEG	POS	AMB	NEG	NEG	NEG	AMB	POS	NEG	NEG	NEG
MSSA2086	POS	NEG	POS	AMB	NEG	NEG	NEG	POS	AMB	NEG	NEG	NEG
MSSA2089	POS	NEG	POS	POS	NEG	NEG	NEG	POS	AMB	NEG	NEG	NEG
MSSA2103	POS	NEG	POS	NEG	NEG	NEG	NEG	AMB	POS	NEG	NEG	NEG
MSSA2173	POS	NEG	POS	POS	NEG	AMB	NEG	AMB	POS	NEG	NEG	NEG
MSSA2183	POS	NEG	POS	POS	NEG	NEG	NEG	AMB	POS	NEG	NEG	NEG
MSSA2196	POS	NEG	POS	POS	NEG	NEG	NEG	AMB	POS	NEG	NEG	NEG
MSSA2219	POS	NEG	POS	POS	NEG	NEG	NEG	AMB	POS	NEG	NEG	POS
MSSA1753	POS	NEG	NEG	NEG	NEG	POS	NEG	POS	NEG	POS	POS	NEG
MSSA1773	POS	NEG	NEG	NEG	NEG	POS	NEG	POS	NEG	POS	POS	NEG
MSSA1811	POS	NEG	POS	AMB	NEG	POS	NEG	POS	NEG	POS	POS	NEG
MSSA1897	POS	NEG	POS	AMB	NEG	POS	NEG	POS	NEG	POS	POS	NEG
MSSA1931	POS	NEG	AMB	NEG	NEG	POS	NEG	POS	NEG	POS	POS	NEG
MSSA2114	POS	NEG	POS	NEG	NEG	POS	NEG	POS	NEG	POS	POS	NEG
MSSA1745	NEG	NEG	POS	POS	NEG	POS	NEG	AMB	POS	NEG	AMB	POS
MSSA1747	NEG	NEG	POS	POS	NEG	POS	NEG	NEG	POS	NEG	NEG	POS
MSSA1749	NEG	NEG	POS	POS	NEG	POS	NEG	NEG	POS	NEG	NEG	POS
MSSA1768	NEG	NEG	POS	POS	NEG	POS	NEG	NEG	POS	NEG	NEG	NEG
MSSA1774	NEG	NEG	POS	POS	NEG	POS	NEG	NEG	POS	NEG	NEG	POS
MSSA1775	NEG	NEG	POS	POS	NEG	POS	NEG	NEG	POS	NEG	AMB	POS
MSSA1776	NEG	NEG	POS	POS	NEG	POS	NEG	NEG	POS	NEG	AMB	POS
MSSA1779	NEG	NEG	POS	POS	NEG	POS	NEG	NEG	POS	NEG	NEG	POS
MSSA1780	NEG	NEG	POS	POS	NEG	POS	NEG	NEG	POS	NEG	NEG	NEG

	VIRULENCE : STAPHYLOCOCCAL SUPERANTIGEN/ENTEROTOXIN-LIKE GENES (SET/SSL)											
Strain	setC	ssl1/set6 probe 1_11	ssl1/set6 probe 2_11	ssl1/set6 probe 1_12	ssl1/set6 probe 2_12	ssl1/set6 probe 4_11	ssl01-RF122	ssl02/set7	ssl02/set7 (MRSA252)	ssl03/set8 probe 1	ssl03/set8 probe 2	ssl03/set8 (MRSA252, SAR0424)
MSSA1781	NEG	NEG	POS	POS	NEG	POS	NEG	NEG	POS	NEG	NEG	POS
MSSA1786	NEG	NEG	POS	POS	NEG	POS	NEG	NEG	POS	NEG	AMB	POS
MSSA1793	NEG	NEG	POS	POS	NEG	AMB	NEG	NEG	POS	NEG	NEG	POS
MSSA1803	NEG	NEG	POS	POS	NEG	POS	NEG	NEG	POS	NEG	NEG	POS
MSSA1836	NEG	NEG	POS	POS	NEG	POS	NEG	NEG	POS	NEG	NEG	POS
MSSA1839	NEG	NEG	POS	POS	NEG	POS	NEG	AMB	POS	NEG	NEG	POS
MSSA1900	NEG	NEG	POS	POS	NEG	POS	NEG	NEG	POS	NEG	AMB	POS
MSSA1910	NEG	NEG	POS	POS	NEG	POS	NEG	NEG	POS	NEG	NEG	POS
MSSA1918	NEG	NEG	POS	POS	NEG	POS	NEG	NEG	POS	NEG	NEG	POS
MSSA1921	NEG	NEG	POS	POS	NEG	POS	NEG	NEG	POS	NEG	NEG	POS
MSSA1923	NEG	NEG	POS	POS	NEG	POS	NEG	NEG	POS	NEG	NEG	NEG
MSSA1981	NEG	NEG	POS	POS	NEG	POS	NEG	NEG	POS	NEG	NEG	POS
MSSA1998	NEG	NEG	POS	POS	NEG	POS	NEG	NEG	POS	NEG	NEG	POS
MSSA2000	NEG	NEG	POS	POS	NEG	AMB	NEG	NEG	POS	NEG	NEG	POS
MSSA2007	NEG	NEG	POS	POS	NEG	POS	NEG	NEG	POS	NEG	NEG	POS
MSSA2012	NEG	NEG	POS	POS	NEG	POS	NEG	NEG	POS	NEG	NEG	POS
MSSA2015	NEG	NEG	POS	POS	NEG	NEG	NEG	NEG	POS	NEG	NEG	POS
MSSA2021	NEG	NEG	POS	POS	NEG	NEG	NEG	NEG	POS	NEG	NEG	POS
MSSA2027	NEG	NEG	POS	POS	NEG	POS	NEG	NEG	POS	NEG	NEG	POS
MSSA2063	NEG	NEG	POS	POS	NEG	POS	NEG	NEG	POS	NEG	NEG	POS
MSSA2064	NEG	NEG	POS	POS	NEG	POS	NEG	NEG	POS	NEG	NEG	POS
MSSA2065	NEG	NEG	POS	POS	NEG	POS	NEG	NEG	POS	NEG	AMB	POS
MSSA2075	NEG	NEG	POS	POS	NEG	POS	NEG	NEG	POS	NEG	NEG	POS
MSSA2093	NEG	NEG	POS	POS	NEG	POS	NEG	NEG	POS	NEG	NEG	POS
MSSA2102	NEG	NEG	POS	POS	NEG	POS	NEG	NEG	POS	NEG	NEG	POS
MSSA2138	NEG	NEG	POS	POS	NEG	POS	NEG	NEG	POS	NEG	NEG	POS
MSSA2168	NEG	NEG	POS	POS	NEG	POS	NEG	NEG	POS	NEG	NEG	POS
MSSA2181	NEG	NEG	POS	POS	NEG	POS	NEG	NEG	POS	NEG	NEG	POS
MSSA2189	NEG	NEG	POS	POS	NEG	POS	NEG	NEG	POS	NEG	AMB	POS
MSSA2214	NEG	NEG	POS	POS	NEG	POS	NEG	NEG	POS	NEG	NEG	POS
MRSA3803	AMB	NEG	POS	AMB	NEG	POS	NEG	NEG	POS	NEG	NEG	NEG
MSSA1750	POS	NEG	POS	POS	NEG	POS	NEG	AMB	POS	AMB	AMB	NEG
MSSA1756	POS	NEG	POS	POS	NEG	POS	NEG	AMB	POS	NEG	NEG	NEG
MSSA1759	POS	NEG	POS	AMB	NEG	POS	NEG	NEG	POS	NEG	NEG	NEG
MSSA1767	POS	NEG	POS	POS	NEG	POS	NEG	AMB	POS	NEG	NEG	NEG
MSSA1787	AMB	NEG	POS	AMB	NEG	POS	NEG	NEG	POS	NEG	NEG	NEG
MSSA1790	POS	NEG	POS	AMB	NEG	POS	NEG	NEG	POS	NEG	NEG	NEG
MSSA1794	NEG	NEG	POS	NEG	NEG	NEG	NEG	NEG	POS	NEG	NEG	NEG
MSSA1808	AMB	NEG	POS	AMB	NEG	POS	NEG	NEG	POS	NEG	NEG	NEG
MSSA1812	AMB	NEG	POS	AMB	NEG	POS	NEG	NEG	POS	NEG	NEG	NEG
MSSA1819	POS	NEG	POS	POS	NEG	POS	NEG	AMB	POS	NEG	NEG	NEG
MSSA1824	POS	NEG	POS	AMB	NEG	POS	NEG	AMB	POS	NEG	NEG	NEG
MSSA1827	POS	NEG	POS	AMB	NEG	POS	NEG	AMB	POS	NEG	NEG	NEG
MSSA1889	AMB	NEG	POS	NEG	NEG	AMB	NEG	NEG	POS	NEG	NEG	NEG
MSSA1895	NEG	NEG	POS	NEG	NEG	NEG	NEG	NEG	POS	NEG	NEG	NEG
MSSA1904	AMB	NEG	POS	NEG	NEG	AMB	NEG	NEG	POS	NEG	NEG	NEG
MSSA1905	NEG	NEG	POS	NEG	NEG	NEG	NEG	NEG	POS	NEG	NEG	NEG
MSSA1907	NEG	NEG	POS	NEG	NEG	NEG	NEG	NEG	POS	NEG	NEG	NEG
MSSA1919	AMB	NEG	POS	AMB	NEG	POS	NEG	NEG	POS	NEG	NEG	NEG
MSSA1920	NEG	NEG	POS	AMB	NEG	AMB	NEG	NEG	POS	NEG	NEG	NEG
MSSA1929	AMB	NEG	POS	NEG	NEG	AMB	NEG	NEG	POS	NEG	NEG	NEG
MSSA1930	NEG	NEG	POS	NEG	NEG	AMB	NEG	NEG	POS	NEG	NEG	NEG
MSSA1934	AMB	NEG	POS	NEG	NEG	AMB	NEG	NEG	POS	NEG	NEG	NEG
MSSA1946	NEG	NEG	POS	NEG	NEG	AMB	NEG	NEG	POS	NEG	NEG	NEG
MSSA1971	AMB	NEG	POS	AMB	NEG	POS	NEG	NEG	POS	NEG	NEG	NEG
MSSA1980	NEG	NEG	POS	NEG	NEG	NEG	NEG	NEG	POS	NEG	NEG	NEG
MSSA1982	AMB	NEG	POS	AMB	NEG	POS	NEG	NEG	POS	NEG	NEG	NEG
MSSA1996	AMB	NEG	POS	NEG	NEG	AMB	NEG	NEG	POS	NEG	NEG	NEG
MSSA2002	AMB	NEG	POS	NEG	NEG	AMB	NEG	NEG	POS	NEG	NEG	NEG
MSSA2014	AMB	NEG	POS	NEG	NEG	AMB	NEG	NEG	POS	NEG	NEG	NEG
MSSA2053	AMB	NEG	POS	NEG	NEG	AMB	NEG	NEG	POS	NEG	NEG	NEG
MSSA2054	NEG	NEG	POS	NEG	NEG	AMB	NEG	NEG	POS	NEG	NEG	NEG
MSSA2058	NEG	NEG	AMB	NEG	NEG	NEG	NEG	NEG	POS	NEG	NEG	NEG
MSSA2068	POS	NEG	POS	AMB	NEG	POS	NEG	NEG	POS	NEG	NEG	NEG
MSSA2070	AMB	NEG	POS	AMB	NEG	AMB	NEG	NEG	POS	NEG	NEG	NEG
MSSA2072	AMB	NEG	POS	AMB	NEG	POS	NEG	NEG	POS	NEG	NEG	NEG
MSSA2073	NEG	NEG	POS	NEG	NEG	NEG	NEG	NEG	POS	NEG	NEG	NEG
MSSA2074	AMB	NEG	POS	AMB	NEG	POS	NEG	NEG	POS	NEG	NEG	NEG
MSSA2107	POS	NEG	POS	AMB	NEG	POS	NEG	NEG	POS	NEG	NEG	NEG
MSSA2108	NEG	NEG	POS	NEG	NEG	NEG	NEG	NEG	POS	NEG	NEG	NEG
MSSA2129	AMB	NEG	POS	AMB	NEG	AMB	NEG	NEG	POS	NEG	NEG	NEG
MSSA2135	POS	NEG	POS	AMB	NEG	POS	NEG	NEG	POS	NEG	NEG	NEG
MSSA2137	POS	NEG	POS	AMB	NEG	POS	NEG	NEG	POS	NEG	NEG	NEG
MSSA2140	POS	NEG	POS	AMB	NEG	POS	NEG	NEG	POS	NEG	NEG	NEG
MSSA2182	NEG	NEG	POS	NEG	NEG	NEG	NEG	NEG	POS	NEG	NEG	NEG
MSSA2188	NEG	NEG	POS	NEG	NEG	NEG	NEG	NEG	POS	NEG	NEG	NEG
MSSA1899	POS	POS	NEG	POS	NEG	POS	NEG	POS	NEG	POS	POS	NEG
MSSA1915	POS	POS	NEG	POS	NEG	POS	NEG	POS	NEG	POS	POS	NEG
MSSA1949	POS	POS	NEG	POS	NEG	POS	NEG	POS	NEG	POS	POS	NEG
MSSA2042	POS	POS	NEG	POS	NEG	POS	NEG	NEG	NEG	POS	POS	NEG
MSSA2077	POS	POS	NEG	POS	NEG	AMB	NEG	NEG	NEG	POS	POS	NEG
MSSA2095	POS	POS	NEG	POS	NEG	POS	NEG	POS	NEG	POS	POS	NEG
MSSA1997	POS	NEG	NEG	NEG	NEG	POS	NEG	POS	NEG	POS	POS	NEG
MSSA1810	POS	NEG	POS	NEG	NEG	NEG	NEG	POS	NEG	NEG	POS	NEG
MSSA1832	POS	NEG	NEG	NEG	NEG	NEG	POS	POS	AMB	AMB	AMB	NEG
MSSA1891	POS	NEG	NEG	NEG	NEG	NEG	POS	POS	NEG	AMB	AMB	NEG
MSSA1892	POS	NEG	NEG	NEG	NEG	NEG	POS	POS	NEG	AMB	AMB	NEG
MSSA1932	POS	NEG	NEG	NEG	NEG	NEG	POS	POS	NEG	AMB	AMB	NEG
MSSA1942	POS	NEG	NEG	NEG	NEG	NEG	POS	POS	NEG	NEG	AMB	NEG
MSSA1957	POS	NEG	NEG	NEG	NEG	NEG	POS	POS	NEG	NEG	NEG	NEG
MSSA1748	POS	NEG	POS	NEG	POS	NEG	NEG	POS	NEG	POS	POS	NEG
MSSA1754	POS	NEG	POS	NEG	POS	NEG	NEG	POS	NEG	POS	POS	NEG
MSSA1770	POS	NEG	POS	NEG	POS	NEG	NEG	POS	NEG	POS	POS	NEG
MSSA1788	POS	NEG	POS	NEG	POS	NEG	NEG	POS	NEG	POS	POS	NEG
MSSA1804	POS	NEG	POS	NEG	POS	NEG	NEG	POS	NEG	POS	POS	NEG
MSSA1818	POS	NEG	POS	NEG	POS	NEG	NEG	POS	NEG	POS	POS	NEG
MSSA1914	POS	NEG	POS	NEG	POS	NEG	NEG	POS	NEG	POS	POS	NEG
MSSA1974	POS	NEG	POS	NEG	POS	NEG	NEG	POS	NEG	POS	POS	NEG
MSSA2023	POS	NEG	POS	NEG	POS	NEG	NEG	POS	NEG	POS	POS	NEG
MSSA2030	POS	NEG	POS	NEG	POS	NEG	NEG	POS	NEG	POS	POS	NEG
MSSA2096	POS	NEG	POS	NEG	POS	NEG	NEG	NEG	NEG	POS	POS	NEG
MSSA2142	POS	NEG	POS	NEG	POS	NEG	NEG	POS	NEG	POS	POS	NEG
MSSA2203	POS	NEG	POS	NEG	POS	NEG	NEG	POS	NEG	POS	POS	NEG
MSSA1901	POS	POS	NEG	POS	NEG	POS	NEG	POS	NEG	POS	POS	NEG
MSSA2013	POS	POS	NEG	POS	NEG	NEG	NEG	POS	NEG	POS	POS	NEG
MSSA2031	POS	POS	NEG	POS	NEG	POS	AMB	POS	NEG	POS	POS	NEG
MSSA2052	POS	POS	NEG	POS	NEG	POS	NEG	POS	NEG	POS	POS	NEG
MSSA2057	POS	POS	NEG	POS	NEG	POS	NEG	POS	NEG	POS	POS	NEG
MSSA2081	POS	NEG	POS	NEG	NEG	POS	NEG	POS	NEG	POS	POS	NEG
MSSA1955	POS	NEG	POS	NEG	POS	NEG	NEG	POS	NEG	POS	POS	NEG
MSSA1941	POS	POS	NEG	POS	NEG	POS	NEG	NEG	POS	NEG	NEG	NEG
MRSA3838	POS	POS	NEG	POS	NEG	POS	NEG	NEG	POS	NEG	NEG	NEG
MRSA3839	POS	POS	NEG	POS	NEG	POS	NEG	NEG	POS	NEG	NEG	NEG
MSSA1801	POS	NEG	POS	NEG	NEG	NEG	NEG	NEG	POS	NEG	NEG	NEG
MSSA2131	POS	POS	NEG	POS	NEG	POS	NEG	NEG	POS	NEG	NEG	NEG
MSSA1792	POS	POS	NEG	NEG	NEG	NEG	NEG	POS	NEG	POS	POS	NEG
MSSA1796	POS	POS	POS	NEG	POS	NEG	NEG	POS	NEG	POS	POS	NEG
MSSA1817	POS	NEG	NEG	NEG	POS	NEG	POS	POS	NEG	POS	POS	NEG
MSSA1911	POS	NEG	NEG	NEG	POS	NEG	NEG	NEG	NEG	NEG	NEG	NEG
MSSA1927	POS	NEG	NEG	NEG	NEG	POS	POS	POS	NEG	POS	POS	NEG
MSSA1928	POS	NEG	NEG	NEG	NEG	POS	POS	POS	NEG	POS	POS	NEG
MSSA1953	POS	NEG	NEG	NEG	NEG	POS	POS	POS	NEG	POS	POS	NEG
MSSA2062	POS	POS	NEG	NEG	NEG	NEG	NEG	POS	NEG	POS	POS	NEG
MSSA2078	POS	POS	NEG	POS	NEG	POS	NEG	AMB	POS	NEG	NEG	NEG
MSSA2136	POS	NEG	POS	AMB	NEG	POS	NEG	POS	NEG	POS	POS	NEG

Strain	VIRULENCE : STAPHYLOCOCCAL SUPERANTIGEN/ENTEROTOXIN-LIKE GENES (SET/SSL), continued												
	ssl04/set9	ssl04/set9 (MRSA252, SAR0425)	ssl05/set3 probe 1	ssl05/set3 probe 612	ssl05/set3 (RF122)	ssl05/set3 (MRSA252)	ssl6/set21	ssl06 (NCTC8325+MW2)	ssl07/set1	ssl07/set1 (MRSA252)	ssl07/set1 (AF188836)	ssl08/set12 (probe 1)	ssl08/set12 (probe 2)
MSSA1805	POS	NEG	POS	POS	AMB	NEG	POS	POS	POS	AMB	NEG	POS	POS
MSSA1823	POS	NEG	POS	POS	AMB	NEG	POS	POS	POS	AMB	NEG	POS	POS
MSSA1837	POS	NEG	POS	POS	AMB	NEG	POS	POS	POS	AMB	NEG	POS	POS
MSSA2044	POS	NEG	POS	POS	AMB	NEG	POS	POS	POS	AMB	NEG	POS	POS
MSSA2046	POS	NEG	POS	POS	AMB	NEG	POS	POS	POS	AMB	NEG	POS	POS
MSSA2059	POS	NEG	POS	POS	NEG	NEG	POS	POS	POS	NEG	NEG	POS	POS
MSSA2076	POS	NEG	POS	POS	NEG	NEG	POS	POS	POS	AMB	NEG	POS	POS
MSSA2200	POS	NEG	POS	POS	AMB	NEG	POS	POS	POS	AMB	NEG	POS	POS
MRSA3797	POS	NEG	POS	POS	NEG	NEG	NEG	NEG	POS	AMB	NEG	POS	POS
MRSA3798	POS	NEG	POS	POS	AMB	NEG	NEG	NEG	POS	AMB	AMB	POS	POS
MRSA3799	POS	NEG	POS	POS	AMB	NEG	NEG	NEG	POS	AMB	NEG	POS	POS
MRSA3800	POS	NEG	POS	POS	AMB	NEG	NEG	NEG	POS	AMB	AMB	POS	POS
MRSA3801	POS	NEG	POS	POS	AMB	NEG	NEG	NEG	POS	AMB	AMB	POS	POS
MRSA3804	POS	NEG	POS	POS	AMB	NEG	NEG	NEG	POS	AMB	AMB	POS	POS
MRSA3806	POS	NEG	POS	POS	AMB	NEG	NEG	NEG	POS	AMB	NEG	POS	POS
MRSA3961	POS	NEG	POS	POS	AMB	NEG	NEG	NEG	POS	AMB	NEG	POS	POS
MRSA3962	POS	NEG	POS	POS	AMB	NEG	NEG	NEG	POS	AMB	NEG	POS	POS
MRSA3963	POS	NEG	POS	POS	AMB	NEG	NEG	NEG	POS	AMB	NEG	POS	POS
MRSA3966	POS	NEG	POS	POS	NEG	NEG	NEG	NEG	POS	AMB	NEG	POS	POS
MRSA3978	POS	NEG	POS	POS	AMB	NEG	NEG	NEG	POS	AMB	NEG	POS	POS
MRSA3979	POS	NEG	POS	POS	NEG	NEG	NEG	NEG	POS	AMB	NEG	POS	POS
MRSA3980	POS	NEG	POS	POS	NEG	NEG	NEG	NEG	POS	AMB	NEG	POS	POS
MRSA4179	POS	NEG	POS	POS	AMB	NEG	NEG	NEG	POS	AMB	NEG	POS	POS
MRSA4216	POS	NEG	POS	POS	AMB	NEG	NEG	NEG	POS	AMB	NEG	POS	POS
MRSA4217	POS	NEG	POS	POS	AMB	NEG	NEG	NEG	POS	AMB	NEG	POS	POS
MRSA4220	POS	NEG	POS	POS	AMB	NEG	NEG	NEG	POS	AMB	NEG	POS	POS
MRSA4221	POS	NEG	POS	POS	AMB	NEG	NEG	NEG	POS	AMB	NEG	POS	POS
MRSA4222	POS	NEG	POS	POS	AMB	NEG	NEG	NEG	POS	AMB	NEG	POS	POS
MSSA1751	POS	NEG	POS	POS	AMB	NEG	NEG	NEG	POS	AMB	AMB	POS	POS
MSSA1760	POS	NEG	POS	POS	AMB	NEG	NEG	NEG	POS	AMB	AMB	POS	POS
MSSA1802	POS	NEG	POS	POS	AMB	NEG	NEG	NEG	POS	AMB	NEG	POS	POS
MSSA1809	POS	NEG	POS	POS	AMB	NEG	NEG	NEG	POS	AMB	NEG	POS	POS
MSSA1815	POS	NEG	POS	POS	AMB	NEG	NEG	NEG	POS	AMB	NEG	POS	POS
MSSA1829	POS	NEG	POS	POS	AMB	NEG	NEG	NEG	POS	AMB	AMB	POS	POS
MSSA1908	POS	NEG	POS	POS	AMB	NEG	NEG	NEG	POS	AMB	NEG	POS	POS
MSSA1960	POS	NEG	POS	POS	NEG	NEG	NEG	NEG	POS	NEG	NEG	POS	POS
MSSA1965	POS	NEG	POS	POS	AMB	NEG	NEG	NEG	POS	AMB	NEG	POS	POS
MSSA1977	POS	NEG	POS	POS	AMB	NEG	NEG	NEG	POS	AMB	NEG	POS	POS
MSSA1978	POS	NEG	POS	POS	AMB	NEG	NEG	NEG	POS	AMB	AMB	POS	POS
MSSA1999	POS	NEG	POS	POS	AMB	NEG	NEG	NEG	POS	AMB	NEG	POS	POS
MSSA2003	POS	NEG	POS	POS	AMB	NEG	NEG	NEG	POS	AMB	NEG	POS	POS
MSSA2006	POS	NEG	POS	POS	AMB	NEG	NEG	NEG	POS	AMB	NEG	POS	POS
MSSA2008	POS	NEG	POS	POS	AMB	NEG	NEG	NEG	POS	AMB	NEG	POS	POS
MSSA2011	POS	NEG	POS	POS	NEG	NEG	NEG	NEG	POS	NEG	NEG	POS	POS
MSSA2017	POS	NEG	POS	POS	NEG	NEG	NEG	NEG	POS	NEG	NEG	POS	POS
MSSA2022	POS	NEG	POS	POS	NEG	NEG	NEG	NEG	POS	NEG	NEG	POS	POS
MSSA2055	POS	NEG	POS	POS	AMB	NEG	NEG	NEG	POS	AMB	NEG	POS	POS
MSSA2060	POS	NEG	POS	POS	AMB	NEG	NEG	NEG	POS	AMB	NEG	POS	POS
MSSA2080	POS	NEG	POS	POS	NEG	NEG	NEG	NEG	POS	NEG	NEG	POS	POS
MSSA2082	POS	NEG	POS	POS	AMB	NEG	NEG	NEG	POS	AMB	AMB	POS	POS
MSSA2084	POS	NEG	POS	POS	NEG	NEG	NEG	NEG	POS	AMB	NEG	POS	POS
MSSA2087	POS	NEG	POS	POS	NEG	NEG	NEG	NEG	POS	AMB	NEG	POS	POS
MSSA2094	POS	NEG	POS	POS	AMB	NEG	NEG	NEG	POS	AMB	NEG	POS	POS
MSSA2097	POS	NEG	POS	POS	AMB	NEG	NEG	NEG	POS	AMB	NEG	POS	POS
MSSA2110	POS	NEG	POS	POS	AMB	NEG	NEG	NEG	POS	AMB	NEG	POS	POS
MSSA2113	POS	NEG	POS	POS	AMB	NEG	NEG	NEG	POS	AMB	NEG	POS	POS
MSSA2132	POS	NEG	POS	POS	AMB	NEG	NEG	NEG	POS	AMB	NEG	POS	POS
MSSA2134	POS	NEG	POS	POS	NEG	NEG	NEG	NEG	POS	AMB	NEG	POS	POS
MSSA1746	POS	NEG	POS	POS	NEG	NEG	POS	POS	POS	AMB	NEG	POS	POS
MSSA1757	POS	NEG	POS	POS	NEG	NEG	POS	POS	POS	AMB	AMB	POS	POS
MSSA1772	POS	NEG	POS	POS	NEG	NEG	POS	POS	POS	AMB	NEG	POS	POS
MSSA1795	POS	NEG	POS	POS	NEG	NEG	POS	POS	POS	AMB	NEG	POS	POS
MSSA1813	POS	NEG	POS	POS	NEG	NEG	POS	POS	POS	AMB	NEG	POS	POS
MSSA1826	POS	NEG	POS	POS	NEG	NEG	POS	POS	POS	AMB	NEG	POS	POS
MSSA1830	POS	NEG	POS	POS	NEG	NEG	POS	POS	POS	AMB	NEG	POS	POS
MSSA1838	POS	NEG	POS	POS	NEG	NEG	POS	POS	POS	AMB	NEG	POS	POS
MSSA1944	POS	NEG	POS	POS	NEG	NEG	POS	POS	POS	AMB	NEG	POS	POS
MSSA1947	POS	NEG	POS	POS	NEG	NEG	POS	POS	POS	AMB	NEG	POS	POS
MSSA1964	POS	NEG	POS	POS	NEG	NEG	POS	POS	POS	AMB	NEG	POS	POS
MSSA1969	POS	NEG	POS	POS	NEG	NEG	POS	POS	POS	AMB	NEG	POS	POS
MSSA2001	POS	NEG	POS	POS	NEG	NEG	POS	POS	POS	AMB	NEG	POS	POS
MSSA2004	POS	NEG	POS	POS	NEG	NEG	POS	POS	POS	AMB	NEG	POS	POS
MSSA2024	POS	NEG	POS	POS	NEG	NEG	POS	POS	POS	NEG	NEG	POS	POS
MSSA2025	POS	NEG	POS	POS	NEG	NEG	POS	POS	POS	AMB	NEG	POS	POS
MSSA2026	POS	NEG	POS	POS	NEG	NEG	POS	POS	POS	AMB	NEG	POS	POS
MSSA2032	POS	NEG	POS	POS	NEG	NEG	POS	POS	POS	AMB	NEG	POS	POS
MSSA2043	POS	NEG	POS	POS	NEG	NEG	POS	POS	POS	AMB	NEG	POS	POS
MSSA2061	POS	NEG	POS	POS	NEG	NEG	POS	POS	POS	AMB	NEG	POS	POS
MSSA2085	POS	NEG	POS	POS	NEG	NEG	POS	POS	POS	AMB	NEG	POS	POS
MSSA2098	POS	NEG	POS	POS	NEG	NEG	POS	POS	POS	AMB	NEG	POS	POS
MSSA2100	POS	NEG	POS	POS	NEG	NEG	POS	POS	POS	AMB	NEG	POS	POS
MSSA2111	POS	NEG	POS	POS	NEG	NEG	POS	POS	POS	AMB	AMB	POS	POS
MSSA2178	POS	NEG	POS	POS	NEG	NEG	POS	POS	POS	AMB	NEG	POS	POS
MSSA2202	POS	NEG	POS	POS	NEG	NEG	POS	POS	POS	AMB	NEG	POS	POS
MSSA2205	POS	NEG	POS	POS	NEG	NEG	POS	POS	POS	AMB	NEG	POS	POS
MRSA3796	POS	NEG	POS	POS	AMB	NEG	POS	POS	POS	AMB	AMB	POS	POS
MRSA3805	POS	NEG	POS	POS	AMB	NEG	POS	POS	POS	AMB	AMB	POS	POS
MRSA3965	POS	NEG	NEG	NEG	NEG	NEG	NEG	NEG	NEG	NEG	NEG	NEG	NEG
MSSA1761	POS	AMB	POS	POS	AMB	NEG	POS	POS	POS	AMB	AMB	POS	POS
MSSA1764	POS	NEG	POS	POS	NEG	NEG	POS	POS	POS	AMB	NEG	POS	POS
MSSA1765	POS	NEG	POS	POS	AMB	NEG	POS	POS	POS	NEG	NEG	POS	POS
MSSA1784	POS	NEG	POS	POS	AMB	NEG	POS	POS	POS	AMB	AMB	POS	POS
MSSA1806	POS	NEG	POS	POS	AMB	NEG	POS	POS	POS	AMB	NEG	POS	POS
MSSA1807	POS	NEG	POS	POS	AMB	NEG	POS	POS	POS	NEG	NEG	POS	POS
MSSA1888	POS	NEG	POS	POS	AMB	NEG	POS	POS	POS	NEG	NEG	POS	POS
MSSA1894	POS	NEG	POS	POS	NEG	NEG	POS	POS	POS	NEG	NEG	POS	POS
MSSA1922	POS	NEG	POS	POS	AMB	NEG	POS	POS	POS	NEG	NEG	POS	POS
MSSA1950	POS	NEG	POS	POS	AMB	NEG	POS	POS	POS	AMB	NEG	POS	POS
MSSA1954	POS	NEG	POS	POS	AMB	NEG	POS	POS	POS	AMB	NEG	POS	POS
MSSA1966	POS	NEG	POS	POS	AMB	NEG	POS	POS	POS	NEG	NEG	POS	POS
MSSA1972	POS	NEG	POS	POS	AMB	NEG	POS	POS	POS	NEG	NEG	POS	POS
MSSA2010	POS	NEG	POS	POS	AMB	NEG	POS	POS	POS	NEG	NEG	POS	POS
MSSA2020	POS	NEG	POS	POS	AMB	NEG	POS	POS	POS	AMB	NEG	POS	POS
MSSA2028	POS	NEG	POS	POS	AMB	NEG	POS	POS	POS	NEG	NEG	POS	POS
MSSA2088	POS	NEG	POS	POS	AMB	NEG	POS	POS	POS	AMB	AMB	POS	POS
MSSA2187	POS	NEG	POS	POS	AMB	NEG	POS	POS	POS	AMB	NEG	POS	POS
MSSA2204	POS	NEG	POS	POS	AMB	NEG	POS	POS	POS	NEG	NEG	POS	POS
MSSA2210	POS	NEG	POS	POS	AMB	NEG	POS	POS	POS	AMB	NEG	POS	POS
MSSA2223	POS	NEG	POS	POS	AMB	NEG	POS	POS	POS	NEG	NEG	POS	POS
MSSA1763	POS	NEG	NEG	POS	NEG	NEG	POS	POS	POS	NEG	NEG	POS	POS
MSSA1777	POS	AMB	NEG	POS	AMB	NEG	POS	POS	POS	AMB	AMB	POS	POS
MSSA2009	POS	NEG	NEG	POS	AMB	NEG	POS	POS	POS	AMB	NEG	POS	POS
MSSA2047	POS	NEG	NEG	POS	AMB	NEG	POS	POS	POS	NEG	NEG	POS	POS
MSSA2079	POS	NEG	NEG	POS	NEG	NEG	POS	POS	POS	AMB	NEG	POS	POS
MSSA2101	POS	NEG	NEG	POS	AMB	NEG	POS	POS	POS	AMB	NEG	POS	POS
MSSA1821	POS	NEG	POS	POS	AMB	NEG	NEG	NEG	POS	NEG	NEG	POS	POS
MSSA1898	POS	NEG	POS	POS	AMB	NEG	NEG	NEG	POS	NEG	NEG	POS	POS
MSSA1973	POS	NEG	POS	POS	AMB	NEG	NEG	NEG	POS	NEG	NEG	POS	POS
MSSA2071	POS	NEG	POS	POS	AMB	NEG	NEG	NEG	POS	NEG	NEG	POS	POS
MSSA2083	POS	NEG	POS	POS	AMB	NEG	NEG	NEG	POS	NEG	NEG	POS	POS
MSSA2106	POS	NEG	POS	POS	AMB	NEG	NEG	NEG	POS	NEG	NEG	POS	POS
MSSA2112	POS	NEG	POS	POS	AMB	NEG	NEG	NEG	POS	NEG	NEG	POS	POS
MSSA2115	POS	NEG	POS	POS	AMB	NEG	NEG	NEG	POS	NEG	NEG	POS	POS
MSSA2195	POS	NEG	POS	POS	AMB	NEG	NEG	NEG	POS	NEG	NEG	POS	POS
MSSA2207	POS	NEG	POS	POS	AMB	AMB	NEG	NEG	POS	NEG	NEG	POS	POS
MSSA1758	POS	NEG	POS	AMB	POS	NEG	POS	POS	POS	AMB	NEG	NEG	POS
MSSA1762	POS	NEG	POS	POS	AMB	NEG	POS	POS	POS	AMB	AMB	POS	POS
MSSA1769	POS	NEG	POS	POS	AMB	NEG	POS	POS	POS	AMB	AMB	POS	POS
MSSA1771	POS	NEG	POS	AMB	POS	NEG	POS	POS	POS	AMB	NEG	NEG	POS
MSSA1789	POS	NEG	POS	AMB	POS	NEG	POS	POS	POS	AMB	NEG	NEG	POS
MSSA1800	POS	NEG	POS	AMB	POS	NEG	POS	POS	POS	AMB	NEG	AMB	POS
MSSA1833	POS	NEG	POS	POS	AMB	NEG	POS	POS	POS	AMB	AMB	POS	POS
MSSA1890	POS	NEG	POS	AMB	POS	NEG	POS	POS	POS	AMB	NEG	NEG	POS
MSSA1903	POS	NEG	POS	POS	AMB	NEG	POS	POS	POS	AMB	AMB	AMB	POS
MSSA1940	POS	NEG	POS	AMB	POS	NEG	POS	POS	POS	AMB	NEG	AMB	POS
MSSA1962	POS	NEG	POS	AMB	POS	NEG	POS	POS	POS	AMB	NEG	NEG	POS
MSSA1963	POS	NEG	POS	AMB	POS	NEG	POS	POS	POS	AMB	NEG	POS	POS
MSSA2016	POS	NEG	POS	AMB	POS	NEG	POS	POS	POS	AMB	NEG	NEG	POS
MSSA2018	POS	NEG	POS	AMB	POS	NEG	POS	POS	POS	AMB	NEG	NEG	POS
MSSA2019	POS	NEG	POS	AMB	POS	NEG	POS	POS	POS	AMB	NEG	NEG	POS
MSSA2029	POS	NEG	POS	AMB	POS	NEG	POS	POS	POS	AMB	NEG	AMB	POS
MSSA2045	POS	NEG	POS	AMB	POS	NEG	POS	POS	POS	AMB	NEG	AMB	POS
MSSA2048	POS	NEG	POS	AMB	POS	NEG	POS	POS	POS	AMB	NEG	NEG	POS
MSSA2049	POS	NEG	POS	AMB	POS	NEG	POS	POS	POS	AMB	NEG	AMB	POS
MSSA2050	POS	NEG	POS	POS	AMB	NEG	POS	POS	POS	AMB	AMB	POS	POS
MSSA2099	POS	NEG	POS	AMB	POS	NEG	POS	POS	POS	NEG	NEG	NEG	POS
MSSA2109	POS	NEG	POS	AMB	POS	NEG	POS	POS	POS	NEG	NEG	NEG	POS
MSSA2130	POS	NEG	POS	AMB	POS	NEG	POS	POS	POS	AMB	AMB	POS	POS
MSSA2133	POS	NEG	POS	AMB	POS	NEG	POS	POS	POS	AMB	NEG	AMB	POS
MSSA2139	POS	NEG	POS	AMB	POS	NEG	POS	POS	POS	AMB	NEG	AMB	POS
MSSA2141	POS	NEG	POS	POS	AMB	NEG	POS	POS	POS	AMB	NEG	POS	POS
MSSA2208	POS	NEG	POS	AMB	POS	NEG	POS	POS	POS	AMB	NEG	NEG	POS
MSSA2209	POS	NEG	POS	AMB	POS	NEG	POS	POS	POS	AMB	NEG	AMB	POS
MSSA1893	POS	NEG	NEG	POS	NEG	NEG	POS	POS	POS	AMB	NEG	POS	POS
MSSA2105	POS	NEG	NEG	POS	AMB	NEG	POS	POS	POS	AMB	NEG	POS	POS
MRSA3836	NEG	POS	POS	NEG	NEG	POS	NEG	NEG	AMB	AMB	POS	NEG	NEG
MRSA3840	NEG	POS	POS	NEG	NEG	POS	NEG	AMB	AMB	AMB	POS	NEG	NEG
MRSA4188	NEG	POS	POS	NEG	POS	POS	NEG	POS	AMB	POS	AMB	NEG	NEG
MSSA1814	NEG	POS	POS	NEG	NEG	POS	NEG	NEG	AMB	AMB	POS	NEG	NEG
MSSA1816	NEG	POS	POS	NEG	NEG	POS	NEG	NEG	NEG	AMB	POS	NEG	NEG
MSSA1825	NEG	POS	POS	NEG	NEG	POS	NEG	AMB	AMB	AMB	POS	NEG	NEG
MSSA1834	NEG	POS	POS	NEG	NEG	POS	NEG	NEG	NEG	AMB	POS	NEG	NEG
MSSA1938	NEG	POS	POS	NEG	NEG	POS	NEG	NEG	AMB	AMB	POS	NEG	NEG
MSSA1948	NEG	POS	POS	NEG	NEG	POS	NEG	NEG	AMB	AMB	POS	NEG	NEG
MSSA1951	NEG	POS	POS	NEG	NEG	POS	NEG	NEG	NEG	AMB	POS	NEG	NEG
MSSA2005	NEG	POS	POS	NEG	NEG	POS	NEG	NEG	AMB	AMB	POS	NEG	NEG
MSSA2051	NEG	POS	POS	NEG	NEG	POS	NEG	AMB	AMB	AMB	POS	NEG	NEG
MSSA2056	NEG	POS	POS	NEG	NEG	POS	NEG	NEG	NEG	AMB	POS	NEG	NEG
MSSA2066	NEG	POS	POS	NEG	POS	POS	NEG	AMB	AMB	AMB	POS	NEG	NEG
MSSA2067	NEG	POS	POS	NEG	NEG	POS	NEG	NEG	AMB	AMB	POS	NEG	NEG
MSSA2086	NEG	POS	POS	NEG	NEG	POS	NEG	NEG	AMB	AMB	POS	NEG	NEG
MSSA2089	NEG	POS	POS	NEG	NEG	POS	NEG	NEG	NEG	AMB	POS	NEG	NEG
MSSA2103	NEG	POS	POS	NEG	NEG	POS	NEG	NEG	AMB	AMB	POS	NEG	NEG
MSSA2173	NEG	POS	POS	NEG	NEG	POS	NEG	NEG	AMB	AMB	POS	NEG	NEG
MSSA2183	NEG	POS	POS	NEG	NEG	POS	NEG	NEG	AMB	AMB	POS	NEG	NEG
MSSA2196	NEG	POS	POS	NEG	NEG	POS	NEG	NEG	AMB	AMB	POS	NEG	NEG
MSSA2219	NEG	POS	POS	NEG	POS	POS	NEG	AMB	AMB	AMB	POS	NEG	NEG
MSSA1753	POS	NEG	POS	AMB	POS	NEG	NEG	NEG	POS	NEG	NEG	POS	POS
MSSA1773	POS	NEG	POS	AMB	POS	NEG	NEG	NEG	POS	NEG	NEG	POS	POS
MSSA1811	POS	NEG	POS	AMB	POS	NEG	NEG	NEG	POS	NEG	NEG	POS	POS
MSSA1897	POS	NEG	POS	POS	AMB	NEG	NEG	NEG	POS	NEG	NEG	POS	POS
MSSA1931	POS	NEG	POS	AMB	POS	NEG	NEG	NEG	POS	NEG	NEG	POS	POS
MSSA2114	POS	NEG	POS	POS	AMB	NEG	NEG	NEG	POS	NEG	NEG	POS	POS
MSSA1745	NEG	POS	NEG	NEG	POS	POS	NEG	POS	AMB	POS	AMB	NEG	NEG
MSSA1747	NEG	POS	NEG	NEG	NEG	POS	NEG	AMB	NEG	POS	NEG	NEG	NEG
MSSA1749	NEG	POS	NEG	NEG	NEG	POS	NEG	NEG	NEG	POS	NEG	NEG	NEG
MSSA1768	NEG	NEG	NEG	NEG	NEG	POS	NEG	NEG	NEG	POS	NEG	NEG	NEG
MSSA1774	NEG	POS	NEG	NEG	NEG	POS	NEG	NEG	NEG	POS	NEG	NEG	NEG
MSSA1775	NEG	POS	NEG	NEG	NEG	POS	NEG	POS	NEG	POS	AMB	NEG	NEG
MSSA1776	NEG	POS	NEG	NEG	POS	POS	NEG	POS	AMB	POS	AMB	NEG	NEG
MSSA1779	NEG	POS	NEG	NEG	NEG	POS	NEG	NEG	NEG	POS	NEG	NEG	NEG
MSSA1780	NEG	POS	NEG	NEG	NEG	POS	NEG	AMB	NEG	POS	NEG	NEG	NEG

Strain	VIRULENCE : STAPHYLOCOCCAL SUPERANTIGEN/ENTEROTOXIN-LIKE GENES (SET/SSL), continued												
Strain	ssl04/set9	ssl04/set9 (MRSA252, SAR0425)	ssl05/set3 probe 1	ssl05/set3 probe 612	ssl05/set3 (RF122)	ssl05/set3 (MRSA252)	ssl6/set21	ssl06 (NCTC8325+MW2)	ssl07/set1	ssl07/set1 (MRSA252)	ssl07/set1 (AF188836)	ssl08/set12 (probe 1)	ssl08/set12 (probe 2)
~~MSSA1781~~	NEG	POS	NEG	NEG	POS	POS	NEG	POS	NEG	POS	AMB	NEG	NEG
MSSA1786	NEG	POS	NEG	NEG	POS	POS	NEG	POS	NEG	POS	AMB	NEG	NEG
MSSA1793	NEG	POS	NEG	NEG	NEG	POS	NEG	NEG	NEG	POS	NEG	NEG	NEG
MSSA1803	NEG	POS	NEG	NEG	NEG	POS	NEG	AMB	NEG	POS	NEG	NEG	NEG
MSSA1836	NEG	POS	NEG	NEG	NEG	POS	NEG	POS	NEG	POS	NEG	NEG	NEG
MSSA1839	NEG	POS	NEG	NEG	NEG	POS	NEG	POS	NEG	POS	AMB	NEG	NEG
MSSA1900	NEG	POS	NEG	NEG	NEG	POS	NEG	NEG	NEG	POS	NEG	NEG	NEG
MSSA1910	NEG	POS	NEG	NEG	NEG	POS	NEG	NEG	NEG	POS	NEG	NEG	NEG
MSSA1918	NEG	POS	NEG	NEG	NEG	POS	NEG	NEG	NEG	POS	NEG	NEG	NEG
MSSA1921	NEG	POS	NEG	NEG	NEG	POS	NEG	NEG	NEG	POS	NEG	NEG	NEG
MSSA1923	NEG	NEG	NEG	NEG	NEG	POS	NEG	AMB	NEG	POS	NEG	NEG	NEG
MSSA1981	NEG	POS	NEG	NEG	NEG	POS	NEG	NEG	AMB	POS	NEG	NEG	NEG
MSSA1998	NEG	POS	NEG	NEG	NEG	POS	NEG	NEG	NEG	POS	NEG	NEG	NEG
MSSA2000	NEG	POS	NEG	NEG	NEG	POS	NEG	NEG	NEG	POS	NEG	NEG	NEG
MSSA2007	NEG	POS	NEG	NEG	NEG	POS	NEG	AMB	AMB	POS	AMB	NEG	NEG
MSSA2012	NEG	POS	NEG	NEG	NEG	POS	NEG	NEG	NEG	POS	NEG	NEG	NEG
MSSA2015	NEG	POS	NEG	NEG	NEG	POS	NEG	NEG	NEG	POS	NEG	NEG	NEG
MSSA2021	NEG	POS	NEG	NEG	NEG	POS	NEG	NEG	NEG	POS	NEG	NEG	NEG
MSSA2027	NEG	POS	NEG	NEG	NEG	POS	NEG	NEG	NEG	POS	NEG	NEG	NEG
MSSA2063	NEG	POS	NEG	NEG	NEG	POS	NEG	NEG	NEG	POS	NEG	NEG	NEG
MSSA2064	NEG	POS	NEG	NEG	NEG	POS	NEG	AMB	NEG	POS	NEG	NEG	NEG
MSSA2065	NEG	POS	NEG	NEG	NEG	POS	NEG	NEG	NEG	POS	NEG	NEG	NEG
MSSA2075	NEG	POS	NEG	NEG	NEG	POS	NEG	NEG	NEG	POS	NEG	NEG	NEG
MSSA2093	NEG	POS	NEG	NEG	NEG	POS	NEG	AMB	NEG	POS	NEG	NEG	NEG
MSSA2102	NEG	POS	NEG	NEG	NEG	POS	NEG	NEG	NEG	POS	NEG	NEG	NEG
MSSA2138	NEG	POS	NEG	NEG	NEG	POS	NEG	AMB	NEG	POS	NEG	NEG	NEG
MSSA2168	NEG	POS	NEG	NEG	NEG	POS	NEG	NEG	NEG	POS	NEG	NEG	NEG
MSSA2181	NEG	POS	NEG	NEG	NEG	POS	NEG	NEG	NEG	POS	NEG	NEG	NEG
MSSA2189	NEG	POS	NEG	NEG	NEG	POS	NEG	POS	NEG	POS	NEG	NEG	NEG
MSSA2214	NEG	POS	NEG	NEG	NEG	POS	NEG	NEG	NEG	POS	NEG	NEG	NEG
MRSA3803	NEG	POS	NEG	NEG	NEG	POS	NEG	POS	NEG	AMB	POS	NEG	NEG
MSSA1750	NEG	POS	NEG	NEG	NEG	POS	NEG	POS	NEG	AMB	POS	NEG	NEG
MSSA1756	NEG	POS	NEG	NEG	NEG	POS	NEG	POS	NEG	AMB	POS	NEG	NEG
MSSA1759	NEG	POS	NEG	NEG	NEG	POS	NEG	POS	NEG	AMB	POS	NEG	NEG
MSSA1767	NEG	POS	NEG	NEG	NEG	POS	NEG	POS	NEG	AMB	POS	NEG	NEG
MSSA1787	NEG	POS	NEG	NEG	NEG	POS	NEG	AMB	NEG	NEG	POS	NEG	NEG
MSSA1790	NEG	POS	NEG	NEG	NEG	POS	NEG	AMB	NEG	NEG	NEG	NEG	NEG
MSSA1794	NEG	POS	NEG	NEG	NEG	POS	NEG	NEG	NEG	NEG	POS	NEG	NEG
MSSA1808	NEG	POS	NEG	NEG	NEG	POS	NEG	POS	NEG	NEG	POS	NEG	NEG
MSSA1812	NEG	POS	NEG	NEG	NEG	POS	NEG	POS	NEG	NEG	POS	NEG	NEG
MSSA1819	NEG	POS	NEG	NEG	NEG	POS	NEG	POS	NEG	AMB	POS	NEG	NEG
MSSA1824	NEG	POS	NEG	NEG	NEG	POS	NEG	POS	NEG	AMB	POS	NEG	NEG
MSSA1827	NEG	POS	NEG	NEG	NEG	POS	NEG	AMB	NEG	NEG	POS	NEG	NEG
MSSA1889	NEG	POS	NEG	NEG	NEG	POS	NEG	AMB	NEG	NEG	POS	NEG	NEG
MSSA1895	NEG	POS	NEG	NEG	NEG	POS	NEG	NEG	NEG	NEG	NEG	NEG	NEG
MSSA1904	NEG	POS	NEG	NEG	NEG	POS	NEG	NEG	NEG	NEG	POS	NEG	NEG
MSSA1905	NEG	POS	NEG	NEG	NEG	POS	NEG	NEG	NEG	NEG	POS	NEG	NEG
MSSA1907	NEG	POS	NEG	NEG	NEG	POS	NEG	NEG	NEG	NEG	POS	NEG	NEG
MSSA1919	NEG	POS	NEG	NEG	NEG	POS	NEG	POS	NEG	NEG	POS	NEG	NEG
MSSA1920	NEG	POS	NEG	NEG	NEG	POS	NEG	NEG	NEG	NEG	NEG	NEG	NEG
MSSA1929	NEG	POS	NEG	NEG	NEG	POS	NEG	AMB	NEG	NEG	POS	NEG	NEG
MSSA1930	NEG	POS	NEG	NEG	NEG	POS	NEG	AMB	NEG	NEG	POS	NEG	NEG
MSSA1934	NEG	POS	NEG	NEG	NEG	POS	NEG	AMB	NEG	NEG	POS	NEG	NEG
MSSA1946	NEG	POS	NEG	NEG	NEG	POS	NEG	NEG	NEG	NEG	NEG	NEG	NEG
MSSA1971	NEG	POS	NEG	NEG	NEG	POS	NEG	AMB	NEG	NEG	POS	NEG	NEG
MSSA1980	NEG	POS	NEG	NEG	NEG	POS	NEG	NEG	NEG	NEG	POS	NEG	NEG
MSSA1982	NEG	POS	NEG	NEG	NEG	POS	NEG	AMB	NEG	NEG	POS	NEG	NEG
MSSA1996	NEG	POS	NEG	NEG	NEG	POS	NEG	NEG	NEG	NEG	POS	NEG	NEG
MSSA2002	NEG	POS	NEG	NEG	NEG	POS	NEG	NEG	NEG	NEG	NEG	NEG	NEG
MSSA2014	NEG	POS	NEG	NEG	NEG	POS	NEG	AMB	NEG	NEG	POS	NEG	NEG
MSSA2053	NEG	POS	NEG	NEG	NEG	POS	NEG	AMB	NEG	NEG	POS	NEG	NEG
MSSA2054	NEG	POS	NEG	NEG	NEG	POS	NEG	NEG	NEG	NEG	POS	NEG	NEG
MSSA2058	NEG	POS	NEG	NEG	NEG	AMB	NEG	NEG	NEG	NEG	POS	NEG	NEG
MSSA2068	NEG	POS	NEG	NEG	NEG	POS	NEG	POS	NEG	AMB	POS	NEG	NEG
MSSA2070	NEG	POS	AMB	NEG	NEG	POS	NEG	AMB	NEG	NEG	POS	NEG	NEG
MSSA2072	NEG	POS	NEG	NEG	NEG	POS	NEG	NEG	NEG	NEG	NEG	NEG	NEG
MSSA2073	NEG	POS	NEG	NEG	NEG	POS	NEG	NEG	NEG	NEG	NEG	NEG	NEG
MSSA2074	NEG	POS	NEG	NEG	NEG	POS	NEG	POS	NEG	NEG	POS	NEG	NEG
MSSA2107	NEG	POS	NEG	NEG	NEG	POS	NEG	POS	NEG	NEG	POS	NEG	NEG
MSSA2108	NEG	POS	NEG	NEG	NEG	POS	NEG	NEG	NEG	NEG	NEG	NEG	NEG
MSSA2129	NEG	POS	NEG	NEG	NEG	POS	NEG	AMB	NEG	NEG	POS	NEG	NEG
MSSA2135	NEG	POS	NEG	NEG	NEG	POS	NEG	POS	NEG	NEG	POS	NEG	NEG
MSSA2137	NEG	POS	NEG	NEG	NEG	POS	NEG	POS	NEG	AMB	POS	NEG	NEG
MSSA2140	NEG	POS	NEG	NEG	NEG	POS	NEG	AMB	NEG	NEG	POS	NEG	NEG
MSSA2182	NEG	POS	NEG	NEG	NEG	POS	NEG	NEG	NEG	NEG	NEG	NEG	NEG
MSSA2188	NEG	POS	NEG	NEG	NEG	POS	NEG	AMB	NEG	NEG	POS	NEG	NEG
MSSA1899	POS	NEG	POS	AMB	POS	NEG	NEG	NEG	POS	AMB	NEG	POS	POS
MSSA1915	POS	NEG	POS	AMB	POS	NEG	NEG	NEG	POS	AMB	AMB	POS	POS
MSSA1949	POS	NEG	POS	AMB	POS	NEG	NEG	NEG	POS	AMB	NEG	POS	POS
MSSA2042	POS	NEG	POS	AMB	POS	NEG	NEG	NEG	POS	AMB	NEG	POS	POS
MSSA2077	POS	NEG	POS	AMB	POS	NEG	NEG	NEG	POS	AMB	NEG	POS	POS
MSSA2095	POS	NEG	POS	POS	AMB	NEG	NEG	NEG	POS	AMB	AMB	POS	POS
MSSA1997	POS	NEG	NEG	POS	AMB	NEG	POS	POS	POS	AMB	NEG	POS	POS
MSSA1810	POS	NEG	POS	POS	NEG	NEG	NEG	NEG	POS	NEG	NEG	POS	POS
MSSA1832	POS	NEG	POS	POS	AMB	NEG	NEG	AMB	POS	AMB	AMB	POS	POS
MSSA1891	POS	NEG	POS	AMB	POS	NEG	NEG	NEG	POS	AMB	NEG	POS	POS
MSSA1892	POS	NEG	POS	AMB	POS	NEG	NEG	NEG	POS	AMB	NEG	POS	POS
MSSA1932	POS	NEG	POS	AMB	POS	NEG	NEG	NEG	POS	AMB	NEG	POS	POS
MSSA1942	POS	NEG	POS	AMB	POS	NEG	NEG	NEG	POS	AMB	NEG	POS	POS
MSSA1957	POS	NEG	POS	AMB	POS	NEG	NEG	NEG	POS	AMB	NEG	POS	POS
MSSA1748	POS	NEG	NEG	POS	AMB	NEG	NEG	NEG	POS	AMB	AMB	POS	POS
MSSA1754	POS	NEG	NEG	POS	AMB	NEG	NEG	NEG	POS	AMB	NEG	POS	POS
MSSA1770	POS	NEG	NEG	POS	NEG	NEG	NEG	NEG	POS	AMB	NEG	POS	POS
MSSA1788	POS	NEG	NEG	POS	NEG	NEG	NEG	NEG	POS	AMB	NEG	POS	POS
MSSA1804	POS	NEG	NEG	POS	AMB	NEG	NEG	NEG	POS	AMB	NEG	POS	POS
MSSA1818	POS	NEG	NEG	POS	AMB	NEG	NEG	NEG	POS	AMB	NEG	POS	POS
MSSA1914	POS	NEG	NEG	POS	AMB	NEG	NEG	NEG	POS	AMB	AMB	POS	POS
MSSA1974	POS	NEG	NEG	POS	NEG	NEG	NEG	NEG	POS	NEG	NEG	POS	POS
MSSA2023	POS	NEG	NEG	POS	AMB	NEG	NEG	NEG	POS	AMB	NEG	POS	POS
MSSA2030	POS	NEG	NEG	POS	AMB	NEG	NEG	NEG	POS	AMB	NEG	POS	POS
MSSA2096	POS	NEG	NEG	POS	NEG	NEG	NEG	NEG	POS	AMB	NEG	POS	POS
MSSA2142	POS	NEG	NEG	POS	AMB	NEG	NEG	NEG	POS	AMB	NEG	POS	POS
MSSA2203	POS	NEG	NEG	POS	AMB	NEG	NEG	NEG	POS	AMB	NEG	POS	POS
MSSA1901	POS	NEG	NEG	AMB	POS	NEG	POS	POS	POS	NEG	NEG	POS	POS
MSSA2013	POS	NEG	NEG	AMB	POS	NEG	POS	POS	POS	AMB	NEG	POS	POS
MSSA2031	POS	AMB	NEG	POS	AMB	NEG	POS	POS	POS	AMB	NEG	POS	POS
MSSA2052	POS	NEG	NEG	AMB	POS	NEG	POS	POS	POS	NEG	NEG	POS	POS
MSSA2057	POS	NEG	NEG	AMB	POS	NEG	POS	POS	POS	NEG	NEG	POS	POS
MSSA2081	POS	NEG	POS	AMB	POS	NEG	NEG	NEG	POS	AMB	NEG	POS	POS
MSSA1955	POS	NEG	NEG	NEG	POS	NEG	NEG	NEG	POS	AMB	AMB	POS	POS
MSSA1941	NEG	POS	NEG	NEG	NEG	POS	NEG	NEG	NEG	AMB	POS	NEG	NEG
MRSA3838	NEG	POS	NEG	NEG	NEG	POS	NEG	NEG	NEG	AMB	POS	NEG	NEG
MRSA3839	NEG	POS	NEG	NEG	NEG	POS	NEG	AMB	AMB	AMB	POS	NEG	NEG
MSSA1801	NEG	POS	NEG	NEG	NEG	POS	NEG	NEG	NEG	POS	NEG	NEG	NEG
MSSA2131	NEG	POS	NEG	NEG	NEG	POS	NEG	AMB	AMB	AMB	POS	NEG	NEG
MSSA1792	POS	NEG	POS	AMB	POS	NEG	NEG	NEG	POS	AMB	NEG	POS	POS
MSSA1796	POS	AMB	NEG	POS	AMB	NEG	POS	POS	POS	AMB	AMB	POS	POS
MSSA1817	POS	NEG	POS	POS	AMB	NEG	NEG	NEG	POS	AMB	AMB	POS	POS
MSSA1911	NEG	POS	NEG	NEG	NEG	NEG	NEG	POS	NEG	NEG	NEG	NEG	NEG
MSSA1927	POS	NEG	POS	AMB	POS	NEG	NEG	NEG	POS	NEG	NEG	POS	POS
MSSA1928	POS	NEG	POS	POS	AMB	NEG	NEG	NEG	POS	NEG	NEG	POS	POS
MSSA1953	POS	NEG	POS	AMB	POS	NEG	NEG	NEG	POS	NEG	NEG	POS	POS
MSSA2062	POS	NEG	POS	AMB	POS	NEG	NEG	NEG	POS	AMB	NEG	POS	POS
MSSA2078	NEG	POS	NEG	NEG	NEG	POS	NEG	POS	NEG	POS	AMB	NEG	NEG
MSSA2136	NEG	POS	POS	AMB	POS	NEG	POS	POS	POS	AMB	AMB	POS	POS

	VIRULENCE : STAPHYLOCOCCAL SUPERANTIGEN/ENTEROTOXIN-LIKE GENES (SET/SSL), continued														
Strain	ssl09/set5 (probe 1)	ssl09/set5 (probe 2)	ssl09/set5 (MRSA252)	ssl10/set4	ssl10/set4 (RF122)	ssl10/set4 (MRSA252)	ssl11/set2 (COL)	ssl11/set2 (Mu50/N315)	ssl11/set2 (MW2/MSSA476)	ssl11/set2 (MRSA252)	setB3	setB3 (MRSA252)	setB2	setB2 (MRSA252)	setB1
MSSA1805	POS	POS	NEG	POS	NEG	NEG	NEG	NEG	POS	NEG	POS	NEG	POS	NEG	POS
MSSA1823	POS	POS	NEG	POS	NEG	NEG	NEG	NEG	POS	NEG	POS	NEG	POS	NEG	POS
MSSA1837	POS	POS	NEG	POS	NEG	NEG	NEG	NEG	POS	NEG	POS	NEG	POS	NEG	POS
MSSA2044	POS	POS	NEG	POS	NEG	AMB	NEG	NEG	POS	NEG	POS	NEG	POS	NEG	POS
MSSA2046	NEG	NEG	NEG	POS	NEG	AMB	NEG	NEG	POS	NEG	POS	NEG	POS	NEG	POS
MSSA2059	POS	POS	NEG	POS	NEG	NEG	NEG	NEG	POS	NEG	POS	NEG	POS	NEG	POS
MSSA2076	POS	POS	NEG	POS	NEG	AMB	NEG	NEG	POS	NEG	POS	NEG	POS	NEG	POS
MSSA2200	POS	POS	NEG	POS	NEG	AMB	NEG	NEG	POS	NEG	POS	NEG	POS	NEG	POS
MRSA3797	POS	POS	NEG	POS	NEG	NEG	NEG	POS	NEG	NEG	POS	NEG	POS	NEG	POS
MRSA3798	POS	POS	AMB	POS	AMB	AMB	NEG	POS	NEG	NEG	POS	NEG	POS	NEG	POS
MRSA3799	POS	POS	NEG	POS	NEG	NEG	NEG	POS	NEG	NEG	POS	NEG	POS	NEG	POS
MRSA3800	POS	POS	NEG	POS	AMB	AMB	NEG	POS	NEG	NEG	POS	NEG	POS	NEG	POS
MRSA3801	POS	POS	NEG	POS	AMB	AMB	NEG	POS	NEG	NEG	POS	NEG	POS	NEG	POS
MRSA3804	POS	POS	NEG	POS	AMB	AMB	NEG	POS	NEG	NEG	POS	NEG	POS	NEG	POS
MRSA3806	POS	POS	NEG	POS	NEG	AMB	NEG	POS	NEG	NEG	POS	NEG	POS	NEG	POS
MRSA3961	POS	POS	NEG	POS	NEG	AMB	NEG	POS	NEG	NEG	POS	NEG	POS	NEG	POS
MRSA3962	POS	POS	NEG	POS	NEG	AMB	NEG	POS	NEG	NEG	POS	NEG	POS	NEG	POS
MRSA3963	POS	POS	NEG	POS	NEG	AMB	NEG	POS	NEG	NEG	POS	NEG	POS	NEG	POS
MRSA3966	POS	POS	NEG	POS	NEG	NEG	NEG	POS	NEG	NEG	POS	NEG	POS	NEG	POS
MRSA3978	POS	POS	NEG	POS	NEG	AMB	NEG	POS	NEG	NEG	POS	NEG	POS	AMB	POS
MRSA3979	POS	POS	NEG	POS	NEG	NEG	NEG	POS	NEG	NEG	POS	NEG	POS	NEG	POS
MRSA3980	POS	POS	NEG	POS	NEG	NEG	NEG	POS	NEG	NEG	POS	NEG	POS	NEG	POS
MRSA4179	POS	POS	NEG	POS	NEG	AMB	NEG	POS	NEG	NEG	POS	NEG	POS	NEG	POS
MRSA4216	POS	POS	NEG	POS	NEG	AMB	NEG	POS	NEG	NEG	POS	NEG	POS	NEG	POS
MRSA4217	POS	POS	NEG	POS	NEG	NEG	NEG	POS	NEG	NEG	POS	NEG	POS	NEG	POS
MRSA4220	POS	POS	NEG	POS	NEG	AMB	NEG	POS	NEG	NEG	POS	NEG	POS	NEG	POS
MRSA4221	POS	POS	NEG	POS	NEG	AMB	NEG	POS	NEG	NEG	POS	NEG	POS	NEG	POS
MRSA4222	POS	POS	NEG	POS	NEG	NEG	NEG	POS	NEG	NEG	POS	AMB	POS	NEG	POS
MSSA1751	POS	POS	NEG	POS	AMB	AMB	NEG	POS	NEG	NEG	POS	NEG	POS	NEG	POS
MSSA1760	POS	POS	NEG	POS	AMB	AMB	NEG	POS	NEG	NEG	POS	NEG	POS	NEG	POS
MSSA1802	POS	POS	NEG	POS	NEG	NEG	NEG	POS	NEG	NEG	POS	NEG	POS	NEG	POS
MSSA1809	POS	POS	NEG	POS	NEG	NEG	NEG	POS	NEG	NEG	POS	NEG	POS	NEG	POS
MSSA1815	POS	POS	NEG	POS	AMB	AMB	NEG	POS	NEG	NEG	POS	NEG	POS	NEG	POS
MSSA1829	POS	POS	NEG	POS	AMB	NEG	NEG	POS	NEG	NEG	POS	NEG	POS	NEG	POS
MSSA1908	POS	POS	NEG	POS	NEG	AMB	NEG	POS	NEG	NEG	POS	NEG	POS	NEG	POS
MSSA1960	POS	POS	NEG	POS	NEG	NEG	NEG	POS	NEG	NEG	POS	NEG	POS	NEG	POS
MSSA1965	POS	POS	NEG	POS	NEG	AMB	NEG	POS	NEG	NEG	POS	NEG	POS	NEG	POS
MSSA1977	POS	POS	NEG	POS	NEG	NEG	NEG	POS	NEG	NEG	POS	NEG	POS	NEG	POS
MSSA1978	POS	POS	NEG	POS	AMB	AMB	NEG	POS	NEG	NEG	POS	NEG	POS	NEG	POS
MSSA1999	POS	POS	NEG	POS	NEG	AMB	NEG	POS	NEG	NEG	POS	NEG	POS	NEG	POS
MSSA2003	POS	POS	NEG	POS	NEG	AMB	NEG	POS	NEG	NEG	POS	NEG	POS	NEG	POS
MSSA2006	POS	POS	NEG	POS	NEG	AMB	NEG	POS	NEG	NEG	POS	NEG	POS	NEG	POS
MSSA2008	POS	POS	NEG	POS	NEG	AMB	NEG	POS	NEG	NEG	POS	NEG	POS	NEG	POS
MSSA2011	POS	POS	NEG	POS	NEG	NEG	NEG	POS	NEG	NEG	POS	NEG	POS	NEG	POS
MSSA2017	POS	POS	NEG	POS	NEG	NEG	NEG	POS	NEG	NEG	POS	NEG	POS	NEG	POS
MSSA2022	POS	POS	NEG	POS	NEG	NEG	NEG	POS	NEG	NEG	POS	NEG	POS	NEG	POS
MSSA2055	POS	POS	NEG	POS	NEG	AMB	NEG	POS	NEG	NEG	POS	NEG	POS	NEG	POS
MSSA2060	POS	POS	NEG	POS	NEG	AMB	NEG	POS	NEG	NEG	POS	NEG	POS	NEG	POS
MSSA2080	POS	POS	NEG	POS	NEG	NEG	NEG	POS	NEG	NEG	POS	NEG	POS	NEG	POS
MSSA2082	POS	POS	NEG	POS	AMB	AMB	NEG	POS	NEG	NEG	POS	NEG	POS	NEG	POS
MSSA2084	POS	POS	NEG	POS	NEG	NEG	NEG	POS	NEG	NEG	POS	NEG	POS	NEG	POS
MSSA2087	POS	POS	NEG	POS	NEG	NEG	NEG	POS	NEG	NEG	POS	NEG	POS	NEG	POS
MSSA2094	POS	POS	NEG	POS	NEG	AMB	NEG	POS	NEG	NEG	POS	NEG	POS	NEG	POS
MSSA2097	POS	POS	NEG	POS	NEG	AMB	NEG	POS	NEG	NEG	POS	NEG	POS	NEG	POS
MSSA2110	POS	POS	NEG	POS	NEG	AMB	NEG	POS	NEG	NEG	POS	NEG	POS	NEG	POS
MSSA2113	POS	POS	NEG	POS	NEG	AMB	NEG	POS	NEG	NEG	POS	NEG	POS	NEG	POS
MSSA2132	POS	POS	NEG	POS	NEG	AMB	NEG	POS	NEG	NEG	POS	NEG	POS	NEG	POS
MSSA2134	POS	POS	NEG	POS	NEG	AMB	NEG	POS	NEG	NEG	POS	NEG	POS	NEG	POS
MSSA1746	POS	POS	NEG	POS	NEG	NEG	NEG	NEG	NEG	NEG	POS	NEG	POS	NEG	POS
MSSA1757	POS	POS	NEG	POS	AMB	AMB	NEG	NEG	NEG	NEG	POS	NEG	POS	NEG	POS
MSSA1772	POS	POS	NEG	POS	NEG	NEG	NEG	NEG	NEG	NEG	POS	NEG	POS	NEG	POS
MSSA1795	POS	POS	NEG	POS	AMB	AMB	NEG	NEG	NEG	NEG	POS	NEG	POS	NEG	POS
MSSA1813	POS	POS	NEG	POS	NEG	NEG	NEG	NEG	NEG	NEG	POS	NEG	POS	NEG	POS
MSSA1826	POS	POS	NEG	POS	NEG	NEG	NEG	NEG	NEG	NEG	POS	NEG	POS	NEG	POS
MSSA1830	POS	POS	NEG	POS	NEG	NEG	NEG	NEG	NEG	NEG	POS	NEG	POS	NEG	POS
MSSA1838	POS	POS	NEG	POS	NEG	NEG	NEG	NEG	NEG	NEG	POS	NEG	POS	NEG	POS
MSSA1944	POS	POS	NEG	POS	NEG	NEG	NEG	NEG	NEG	NEG	POS	NEG	POS	NEG	POS
MSSA1947	POS	POS	NEG	POS	NEG	AMB	NEG	NEG	NEG	NEG	POS	NEG	POS	NEG	POS
MSSA1964	POS	POS	NEG	POS	NEG	AMB	NEG	NEG	NEG	NEG	POS	NEG	POS	NEG	POS
MSSA1969	POS	POS	NEG	POS	NEG	NEG	NEG	NEG	NEG	NEG	POS	NEG	POS	NEG	POS
MSSA2001	POS	POS	NEG	POS	NEG	AMB	NEG	NEG	NEG	NEG	POS	NEG	POS	NEG	POS
MSSA2004	POS	POS	NEG	POS	NEG	AMB	NEG	NEG	NEG	NEG	POS	NEG	POS	NEG	POS
MSSA2024	POS	POS	NEG	POS	NEG	NEG	NEG	NEG	NEG	NEG	POS	NEG	POS	NEG	POS
MSSA2025	POS	POS	NEG	POS	NEG	NEG	NEG	NEG	NEG	NEG	POS	NEG	POS	NEG	POS
MSSA2026	POS	POS	NEG	POS	NEG	NEG	NEG	NEG	NEG	NEG	POS	NEG	POS	NEG	POS
MSSA2032	POS	POS	NEG	POS	NEG	NEG	NEG	NEG	NEG	NEG	POS	NEG	POS	NEG	POS
MSSA2043	POS	POS	NEG	POS	NEG	AMB	NEG	NEG	NEG	NEG	POS	NEG	POS	NEG	POS
MSSA2061	POS	POS	NEG	POS	NEG	AMB	NEG	NEG	NEG	NEG	POS	NEG	POS	NEG	POS
MSSA2085	POS	POS	NEG	POS	NEG	AMB	NEG	NEG	NEG	NEG	POS	NEG	POS	NEG	POS
MSSA2098	POS	POS	NEG	POS	NEG	AMB	NEG	NEG	NEG	NEG	POS	NEG	POS	NEG	POS
MSSA2100	POS	POS	NEG	POS	NEG	AMB	NEG	NEG	NEG	NEG	POS	NEG	POS	NEG	POS
MSSA2111	POS	POS	NEG	POS	NEG	AMB	NEG	NEG	NEG	NEG	POS	NEG	POS	NEG	POS
MSSA2178	POS	POS	NEG	POS	NEG	AMB	NEG	NEG	NEG	NEG	POS	NEG	POS	NEG	POS
MSSA2202	POS	POS	NEG	POS	NEG	NEG	NEG	NEG	NEG	NEG	POS	NEG	POS	NEG	POS
MSSA2205	POS	POS	NEG	POS	NEG	AMB	NEG	NEG	NEG	NEG	POS	NEG	POS	NEG	POS
MRSA3796	POS	POS	NEG	POS	AMB	AMB	POS	NEG	NEG	NEG	POS	NEG	POS	NEG	POS
MRSA3805	POS	POS	NEG	POS	NEG	AMB	POS	NEG	NEG	NEG	POS	NEG	POS	NEG	POS
MRSA3965	POS	POS	NEG	POS	NEG	NEG	POS	NEG	NEG	NEG	POS	NEG	POS	NEG	POS
MSSA1761	POS	POS	NEG	POS	AMB	AMB	POS	NEG	NEG	NEG	POS	NEG	POS	NEG	POS
MSSA1764	POS	POS	NEG	POS	NEG	NEG	POS	NEG	NEG	NEG	POS	NEG	POS	NEG	POS
MSSA1765	POS	POS	NEG	POS	NEG	AMB	POS	NEG	NEG	NEG	POS	NEG	POS	NEG	POS
MSSA1784	POS	POS	NEG	POS	AMB	AMB	POS	NEG	NEG	NEG	POS	NEG	POS	NEG	POS
MSSA1806	POS	POS	NEG	POS	NEG	AMB	POS	NEG	NEG	NEG	POS	NEG	POS	NEG	POS
MSSA1807	POS	POS	NEG	POS	AMB	NEG	POS	NEG	NEG	NEG	POS	NEG	POS	NEG	POS
MSSA1888	POS	POS	NEG	POS	NEG	NEG	POS	NEG	NEG	NEG	POS	NEG	POS	NEG	POS
MSSA1894	POS	POS	NEG	POS	NEG	NEG	POS	NEG	NEG	NEG	POS	NEG	POS	NEG	POS
MSSA1922	POS	POS	NEG	POS	NEG	AMB	POS	NEG	NEG	NEG	POS	NEG	POS	NEG	POS
MSSA1950	POS	POS	NEG	POS	NEG	AMB	POS	NEG	NEG	NEG	POS	NEG	POS	NEG	POS
MSSA1954	POS	POS	NEG	POS	NEG	NEG	POS	NEG	NEG	NEG	POS	NEG	POS	NEG	POS
MSSA1966	POS	POS	NEG	POS	NEG	AMB	POS	NEG	NEG	NEG	POS	NEG	POS	NEG	POS
MSSA1972	POS	POS	NEG	POS	NEG	NEG	POS	NEG	NEG	NEG	POS	NEG	POS	NEG	POS
MSSA2010	POS	POS	NEG	POS	NEG	AMB	POS	NEG	NEG	NEG	POS	NEG	POS	NEG	POS
MSSA2020	POS	POS	NEG	POS	NEG	NEG	POS	NEG	NEG	NEG	POS	NEG	POS	NEG	POS
MSSA2028	POS	POS	NEG	POS	AMB	AMB	POS	NEG	NEG	NEG	POS	NEG	POS	NEG	POS
MSSA2088	POS	POS	NEG	POS	AMB	AMB	POS	NEG	NEG	NEG	POS	NEG	POS	NEG	POS
MSSA2187	POS	POS	NEG	POS	NEG	AMB	POS	NEG	NEG	NEG	POS	NEG	POS	NEG	POS
MSSA2204	POS	POS	NEG	POS	AMB	AMB	POS	NEG	NEG	NEG	POS	NEG	POS	NEG	POS
MSSA2210	POS	POS	NEG	POS	NEG	NEG	POS	NEG	NEG	NEG	POS	NEG	POS	NEG	POS
MSSA2223	POS	POS	NEG	POS	NEG	AMB	POS	NEG	NEG	NEG	POS	NEG	POS	NEG	POS
MSSA1763	POS	POS	NEG	POS	NEG	NEG	NEG	NEG	NEG	NEG	POS	NEG	POS	NEG	POS
MSSA1777	POS	POS	NEG	POS	AMB	AMB	NEG	NEG	NEG	NEG	POS	NEG	POS	NEG	POS
MSSA2009	POS	POS	NEG	POS	NEG	AMB	NEG	NEG	NEG	NEG	POS	NEG	POS	NEG	POS
MSSA2047	POS	POS	NEG	POS	NEG	AMB	NEG	NEG	NEG	NEG	POS	NEG	POS	NEG	POS
MSSA2079	POS	POS	NEG	POS	NEG	NEG	NEG	NEG	NEG	NEG	POS	NEG	POS	NEG	POS
MSSA2101	POS	POS	NEG	POS	NEG	NEG	NEG	NEG	NEG	NEG	POS	NEG	POS	NEG	POS
MSSA1821	POS	POS	NEG	POS	NEG	NEG	POS	NEG	NEG	NEG	POS	NEG	POS	NEG	POS
MSSA1898	POS	POS	NEG	POS	NEG	NEG	POS	NEG	NEG	NEG	POS	NEG	POS	NEG	POS
MSSA1973	POS	POS	NEG	POS	NEG	AMB	POS	NEG	NEG	NEG	POS	NEG	POS	NEG	POS
MSSA2071	POS	POS	NEG	POS	NEG	AMB	POS	NEG	NEG	NEG	POS	NEG	POS	POS	POS
MSSA2083	POS	POS	NEG	POS	NEG	AMB	POS	NEG	NEG	NEG	POS	NEG	POS	NEG	POS
MSSA2106	POS	POS	NEG	POS	NEG	AMB	POS	NEG	NEG	NEG	POS	NEG	POS	NEG	POS
MSSA2112	POS	POS	NEG	POS	NEG	AMB	POS	NEG	NEG	NEG	POS	NEG	POS	NEG	POS
MSSA2115	POS	POS	NEG	POS	NEG	AMB	POS	NEG	NEG	NEG	POS	NEG	POS	NEG	POS
MSSA2195	POS	POS	NEG	POS	NEG	AMB	POS	NEG	NEG	NEG	POS	NEG	POS	NEG	POS
MSSA2207	POS	POS	NEG	POS	AMB	AMB	POS	NEG	NEG	NEG	POS	NEG	POS	NEG	POS
MSSA1758	POS	POS	NEG	AMB	POS	NEG	NEG	NEG	NEG	POS	POS	NEG	AMB	NEG	POS
MSSA1762	POS	POS	NEG	AMB	POS	AMB	NEG	NEG	NEG	POS	POS	NEG	POS	AMB	POS
MSSA1769	POS	POS	NEG	POS	AMB	AMB	NEG	NEG	NEG	POS	POS	NEG	POS	NEG	POS
MSSA1771	POS	POS	NEG	AMB	POS	NEG	NEG	NEG	NEG	POS	POS	NEG	AMB	NEG	POS
MSSA1789	POS	POS	NEG	AMB	POS	NEG	NEG	NEG	NEG	POS	POS	NEG	AMB	NEG	POS
MSSA1800	POS	POS	NEG	AMB	POS	NEG	NEG	NEG	NEG	POS	POS	NEG	POS	NEG	POS
MSSA1833	POS	POS	NEG	POS	AMB	NEG	NEG	NEG	NEG	POS	POS	NEG	POS	NEG	POS
MSSA1890	POS	POS	NEG	POS	AMB	NEG	NEG	NEG	NEG	POS	POS	NEG	AMB	NEG	POS
MSSA1903	POS	POS	NEG	AMB	POS	AMB	NEG	NEG	NEG	POS	POS	NEG	POS	NEG	POS
MSSA1940	POS	POS	NEG	AMB	POS	NEG	NEG	NEG	NEG	POS	POS	NEG	POS	NEG	POS
MSSA1962	POS	POS	NEG	AMB	POS	NEG	NEG	NEG	NEG	POS	POS	NEG	POS	NEG	POS
MSSA1963	POS	POS	NEG	POS	AMB	NEG	NEG	NEG	NEG	POS	POS	NEG	POS	NEG	POS
MSSA2016	POS	POS	NEG	AMB	POS	NEG	NEG	NEG	NEG	POS	POS	NEG	POS	NEG	POS
MSSA2018	POS	POS	NEG	AMB	POS	NEG	NEG	NEG	NEG	POS	POS	NEG	POS	NEG	POS
MSSA2019	POS	POS	NEG	AMB	POS	NEG	NEG	NEG	NEG	POS	POS	NEG	AMB	NEG	POS
MSSA2029	POS	POS	NEG	POS	AMB	NEG	NEG	NEG	NEG	POS	POS	NEG	POS	NEG	POS
MSSA2045	POS	POS	NEG	POS	AMB	NEG	NEG	NEG	NEG	POS	POS	NEG	POS	NEG	POS
MSSA2048	POS	POS	NEG	POS	AMB	NEG	NEG	NEG	NEG	POS	POS	NEG	POS	NEG	POS
MSSA2049	POS	POS	NEG	AMB	POS	NEG	NEG	NEG	NEG	POS	POS	NEG	POS	NEG	POS
MSSA2050	POS	POS	NEG	POS	AMB	AMB	NEG	NEG	NEG	POS	POS	NEG	POS	NEG	POS
MSSA2099	AMB	POS	NEG	AMB	POS	NEG	NEG	NEG	NEG	POS	POS	NEG	NEG	NEG	AMB
MSSA2109	POS	POS	NEG	AMB	POS	NEG	NEG	NEG	NEG	POS	POS	NEG	NEG	NEG	POS
MSSA2130	POS	POS	NEG	AMB	POS	NEG	NEG	NEG	NEG	POS	POS	NEG	POS	NEG	POS
MSSA2133	POS	POS	NEG	AMB	POS	NEG	NEG	NEG	NEG	POS	POS	NEG	POS	NEG	POS
MSSA2139	POS	POS	NEG	AMB	POS	NEG	NEG	NEG	NEG	POS	POS	NEG	POS	NEG	POS
MSSA2141	POS	POS	NEG	AMB	POS	AMB	NEG	NEG	NEG	POS	POS	NEG	POS	NEG	POS
MSSA2208	POS	POS	NEG	POS	AMB	NEG	NEG	NEG	NEG	POS	POS	NEG	POS	NEG	POS
MSSA2209	POS	POS	NEG	AMB	POS	NEG	NEG	NEG	NEG	POS	POS	NEG	POS	NEG	POS
MSSA1893	POS	POS	NEG	POS	NEG	NEG	NEG	NEG	NEG	NEG	POS	NEG	POS	NEG	POS
MSSA2105	POS	POS	NEG	POS	NEG	AMB	NEG	NEG	NEG	NEG	POS	NEG	POS	NEG	POS
MRSA3836	AMB	POS	NEG	NEG	NEG	NEG	NEG	NEG	NEG	NEG	NEG	NEG	NEG	NEG	NEG
MRSA3840	POS	POS	NEG	NEG	NEG	NEG	NEG	NEG	NEG	NEG	NEG	NEG	NEG	NEG	NEG
MRSA4188	POS	POS	NEG	POS	NEG	AMB	NEG	NEG	NEG	NEG	NEG	NEG	NEG	NEG	AMB
MSSA1814	POS	POS	NEG	NEG	NEG	NEG	NEG	NEG	NEG	AMB	NEG	NEG	NEG	NEG	NEG
MSSA1816	NEG	NEG	NEG	NEG	NEG	NEG	NEG	NEG	NEG	NEG	NEG	NEG	NEG	NEG	NEG
MSSA1825	AMB	POS	NEG	NEG	NEG	NEG	NEG	NEG	NEG	NEG	NEG	NEG	NEG	NEG	NEG
MSSA1834	NEG	NEG	NEG	NEG	NEG	NEG	NEG	NEG	NEG	NEG	NEG	NEG	NEG	NEG	NEG
MSSA1938	POS	POS	NEG	NEG	NEG	NEG	NEG	NEG	NEG	NEG	NEG	NEG	NEG	NEG	NEG
MSSA1948	POS	POS	NEG	NEG	NEG	NEG	NEG	NEG	NEG	NEG	NEG	NEG	NEG	NEG	NEG
MSSA1951	NEG	AMB	NEG	NEG	NEG	NEG	NEG	NEG	NEG	NEG	NEG	NEG	NEG	NEG	NEG
MSSA2005	AMB	POS	NEG	NEG	NEG	NEG	NEG	NEG	NEG	NEG	NEG	NEG	NEG	NEG	NEG
MSSA2051	POS	POS	NEG	POS	NEG	NEG	NEG	NEG	NEG	NEG	NEG	NEG	NEG	NEG	NEG
MSSA2056	POS	POS	NEG	NEG	NEG	NEG	NEG	NEG	NEG	NEG	NEG	NEG	NEG	NEG	NEG
MSSA2066	POS	POS	NEG	POS	NEG	NEG	NEG	NEG	NEG	NEG	NEG	NEG	NEG	NEG	NEG
MSSA2067	NEG	POS	NEG	NEG	NEG	NEG	NEG	NEG	NEG	NEG	NEG	NEG	NEG	NEG	NEG
MSSA2086	NEG	AMB	NEG	NEG	NEG	NEG	NEG	NEG	NEG	NEG	NEG	NEG	NEG	NEG	NEG
MSSA2089	NEG	AMB	NEG	NEG	NEG	NEG	NEG	NEG	NEG	NEG	NEG	NEG	NEG	NEG	NEG
MSSA2103	NEG	NEG	NEG	NEG	NEG	NEG	NEG	NEG	NEG	NEG	NEG	NEG	NEG	NEG	NEG
MSSA2173	POS	POS	NEG	POS	NEG	NEG	NEG	NEG	NEG	NEG	NEG	NEG	NEG	NEG	NEG
MSSA2183	POS	POS	NEG	POS	NEG	NEG	NEG	NEG	NEG	NEG	NEG	NEG	NEG	NEG	NEG
MSSA2196	POS	POS	NEG	POS	NEG	NEG	NEG	NEG	NEG	NEG	NEG	NEG	NEG	NEG	NEG
MSSA2219	POS	POS	NEG	POS	NEG	NEG	NEG	NEG	NEG	NEG	NEG	NEG	NEG	NEG	NEG
MSSA1753	POS	POS	NEG	AMB	POS	NEG	NEG	NEG	NEG	NEG	POS	NEG	POS	NEG	POS
MSSA1773	POS	POS	NEG	POS	AMB	NEG	NEG	NEG	NEG	NEG	POS	NEG	POS	NEG	POS
MSSA1811	POS	POS	NEG	AMB	POS	NEG	NEG	NEG	NEG	NEG	POS	NEG	POS	NEG	POS
MSSA1897	POS	POS	NEG	POS	AMB	AMB	NEG	NEG	NEG	NEG	POS	NEG	POS	NEG	POS
MSSA1931	POS	POS	NEG	POS	AMB	NEG	NEG	NEG	NEG	NEG	POS	NEG	POS	NEG	POS
MSSA2114	POS	POS	NEG	POS	AMB	AMB	NEG	NEG	NEG	NEG	POS	NEG	POS	NEG	POS
MSSA1745	NEG	NEG	POS	AMB	AMB	POS	NEG	NEG	NEG	POS	NEG	POS	NEG	POS	POS
MSSA1747	NEG	NEG	POS	AMB	NEG	POS	NEG	NEG	NEG	POS	NEG	POS	NEG	POS	POS
MSSA1749	NEG	NEG	POS	AMB	NEG	POS	NEG	NEG	NEG	POS	NEG	POS	NEG	POS	POS
MSSA1768	NEG	NEG	POS	AMB	NEG	POS	NEG	NEG	NEG	POS	NEG	POS	NEG	POS	POS
MSSA1774	NEG	NEG	POS	AMB	NEG	POS	NEG	NEG	NEG	POS	NEG	POS	NEG	POS	POS
MSSA1775	NEG	NEG	POS	AMB	NEG	POS	NEG	NEG	NEG	POS	NEG	POS	NEG	POS	POS
MSSA1776	NEG	NEG	POS	AMB	NEG	POS	NEG	NEG	NEG	POS	NEG	POS	NEG	POS	POS
MSSA1779	NEG	NEG	POS	AMB	NEG	POS	NEG	NEG	NEG	POS	NEG	POS	NEG	POS	POS
MSSA1780	NEG	NEG	POS	AMB	NEG	POS	NEG	NEG	NEG	POS	NEG	POS	NEG	POS	POS

	VIRULENCE : STAPHYLOCOCCAL SUPERANTIGEN/ENTEROTOXIN-LIKE GENES (SET/SSL), continued														
**Strain**	**ssl09/set5** (probe 1)	**ssl09/set5** (probe 2)	**ssl09/set5** (MRSA252)	**ssl10/set4**	**ssl10/set4** (RF122)	**ssl10/set4** (MRSA252)	**ssl11/set2** (COL)	**ssl11/set2** (Mu50/N315)	**ssl11/set2** (MW2/MSSA476)	**ssl11/set2** (MRSA252)	**setB3**	**setB3** (MRSA252)	**setB2**	**setB2** (MRSA252)	**setB1**
MSSA1781	NEG	NEG	POS	AMB	NEG	POS	NEG	NEG	NEG	POS	NEG	POS	NEG	POS	POS
MSSA1786	NEG	NEG	POS	AMB	NEG	POS	NEG	NEG	NEG	POS	NEG	POS	NEG	POS	POS
MSSA1793	NEG	NEG	POS	AMB	NEG	POS	NEG	NEG	NEG	POS	NEG	POS	NEG	POS	POS
MSSA1803	NEG	NEG	POS	AMB	NEG	POS	NEG	NEG	NEG	POS	NEG	POS	NEG	POS	POS
MSSA1836	NEG	NEG	POS	AMB	NEG	POS	NEG	NEG	NEG	POS	NEG	POS	NEG	POS	POS
MSSA1839	NEG	NEG	POS	AMB	NEG	POS	NEG	NEG	NEG	POS	NEG	POS	NEG	POS	POS
MSSA1900	NEG	NEG	POS	AMB	NEG	POS	NEG	NEG	NEG	POS	NEG	POS	NEG	POS	POS
MSSA1910	NEG	NEG	POS	AMB	NEG	POS	NEG	NEG	NEG	POS	NEG	POS	NEG	POS	POS
MSSA1918	NEG	NEG	POS	AMB	NEG	POS	NEG	NEG	NEG	POS	NEG	POS	NEG	POS	POS
MSSA1921	NEG	NEG	POS	AMB	NEG	POS	NEG	NEG	NEG	POS	NEG	POS	NEG	POS	POS
MSSA1923	NEG	NEG	POS	AMB	NEG	POS	NEG	NEG	NEG	POS	NEG	POS	NEG	POS	POS
MSSA1981	NEG	NEG	POS	AMB	NEG	POS	NEG	NEG	NEG	POS	NEG	POS	NEG	POS	POS
MSSA1998	NEG	NEG	POS	AMB	NEG	POS	NEG	NEG	NEG	POS	NEG	POS	NEG	POS	POS
MSSA2000	NEG	NEG	POS	AMB	NEG	POS	NEG	NEG	NEG	POS	NEG	POS	NEG	POS	POS
MSSA2007	NEG	NEG	POS	POS	NEG	AMB	NEG	NEG	NEG	POS	NEG	POS	NEG	POS	POS
MSSA2012	NEG	NEG	POS	AMB	NEG	POS	NEG	NEG	NEG	POS	NEG	POS	NEG	POS	POS
MSSA2015	NEG	NEG	POS	AMB	NEG	POS	NEG	NEG	NEG	POS	NEG	POS	NEG	POS	POS
MSSA2021	NEG	NEG	POS	AMB	NEG	POS	NEG	NEG	NEG	POS	NEG	POS	NEG	POS	POS
MSSA2027	NEG	NEG	POS	AMB	NEG	POS	NEG	NEG	NEG	POS	NEG	POS	NEG	POS	POS
MSSA2063	NEG	NEG	POS	AMB	NEG	POS	NEG	NEG	NEG	POS	NEG	POS	NEG	POS	POS
MSSA2064	NEG	NEG	POS	AMB	NEG	POS	NEG	NEG	NEG	POS	NEG	POS	NEG	POS	POS
MSSA2065	NEG	NEG	POS	AMB	NEG	POS	NEG	NEG	NEG	POS	NEG	POS	NEG	POS	POS
MSSA2075	NEG	NEG	POS	AMB	NEG	POS	NEG	NEG	NEG	POS	NEG	POS	NEG	POS	POS
MSSA2093	NEG	NEG	POS	AMB	NEG	POS	NEG	NEG	NEG	POS	NEG	POS	NEG	POS	POS
MSSA2102	NEG	NEG	POS	AMB	NEG	POS	NEG	NEG	NEG	POS	NEG	POS	NEG	POS	POS
MSSA2138	NEG	NEG	POS	AMB	NEG	POS	NEG	NEG	NEG	POS	NEG	POS	NEG	POS	POS
MSSA2168	NEG	NEG	POS	AMB	NEG	POS	NEG	NEG	NEG	POS	NEG	POS	NEG	POS	POS
MSSA2181	NEG	NEG	POS	AMB	NEG	POS	NEG	NEG	NEG	POS	NEG	POS	NEG	POS	POS
MSSA2189	NEG	NEG	POS	AMB	NEG	POS	NEG	NEG	NEG	POS	NEG	POS	NEG	POS	POS
MSSA2214	NEG	NEG	POS	AMB	NEG	POS	NEG	NEG	NEG	POS	NEG	POS	NEG	POS	POS
MRSA3803	NEG	NEG	POS	AMB	NEG	POS	NEG	NEG	NEG	NEG	NEG	POS	NEG	AMB	NEG
MSSA1750	NEG	NEG	POS	AMB	NEG	POS	NEG	NEG	NEG	NEG	NEG	POS	NEG	POS	AMB
MSSA1756	NEG	NEG	POS	POS	AMB	AMB	NEG	NEG	NEG	NEG	NEG	POS	NEG	AMB	NEG
MSSA1759	NEG	NEG	POS	POS	NEG	AMB	NEG	NEG	NEG	NEG	NEG	POS	NEG	NEG	NEG
MSSA1767	NEG	NEG	POS	AMB	AMB	POS	NEG	NEG	NEG	NEG	NEG	POS	NEG	AMB	NEG
MSSA1787	NEG	NEG	POS	AMB	NEG	POS	NEG	NEG	NEG	NEG	NEG	POS	NEG	NEG	NEG
MSSA1790	NEG	NEG	POS	AMB	NEG	POS	NEG	NEG	NEG	NEG	NEG	POS	NEG	NEG	NEG
MSSA1794	NEG	NEG	POS	AMB	NEG	POS	NEG	NEG	NEG	NEG	NEG	POS	NEG	NEG	NEG
MSSA1808	NEG	NEG	POS	AMB	NEG	POS	NEG	NEG	NEG	NEG	NEG	POS	NEG	AMB	NEG
MSSA1812	NEG	NEG	POS	AMB	NEG	POS	NEG	NEG	NEG	NEG	NEG	POS	NEG	POS	NEG
MSSA1819	NEG	NEG	POS	AMB	NEG	POS	NEG	NEG	NEG	NEG	NEG	POS	NEG	POS	NEG
MSSA1824	NEG	NEG	POS	AMB	NEG	POS	NEG	NEG	NEG	NEG	NEG	POS	NEG	AMB	NEG
MSSA1827	NEG	NEG	POS	AMB	NEG	POS	NEG	NEG	NEG	NEG	NEG	POS	NEG	POS	NEG
MSSA1889	NEG	NEG	POS	POS	NEG	AMB	NEG	NEG	NEG	NEG	NEG	POS	NEG	NEG	NEG
MSSA1895	NEG	NEG	POS	AMB	NEG	POS	NEG	NEG	NEG	NEG	NEG	POS	NEG	NEG	NEG
MSSA1904	NEG	NEG	POS	AMB	NEG	POS	NEG	NEG	NEG	NEG	NEG	POS	NEG	NEG	NEG
MSSA1905	NEG	NEG	POS	AMB	NEG	POS	NEG	NEG	NEG	NEG	NEG	POS	NEG	NEG	NEG
MSSA1907	NEG	NEG	POS	AMB	NEG	POS	NEG	NEG	NEG	NEG	NEG	POS	NEG	NEG	NEG
MSSA1919	NEG	NEG	POS	AMB	NEG	POS	NEG	NEG	NEG	NEG	NEG	POS	NEG	AMB	NEG
MSSA1920	NEG	NEG	POS	AMB	NEG	POS	NEG	NEG	NEG	NEG	NEG	POS	NEG	NEG	NEG
MSSA1929	NEG	NEG	POS	AMB	NEG	POS	NEG	NEG	NEG	NEG	NEG	POS	NEG	NEG	NEG
MSSA1930	NEG	NEG	POS	AMB	NEG	POS	NEG	NEG	NEG	NEG	NEG	POS	NEG	NEG	NEG
MSSA1934	NEG	NEG	POS	AMB	NEG	POS	NEG	NEG	NEG	NEG	NEG	POS	NEG	NEG	NEG
MSSA1946	NEG	NEG	POS	AMB	NEG	POS	NEG	NEG	NEG	NEG	NEG	POS	NEG	NEG	NEG
MSSA1971	NEG	NEG	POS	AMB	NEG	POS	NEG	NEG	NEG	NEG	NEG	POS	NEG	NEG	NEG
MSSA1980	NEG	NEG	POS	AMB	NEG	POS	NEG	NEG	NEG	NEG	NEG	POS	NEG	NEG	NEG
MSSA1982	NEG	NEG	POS	AMB	NEG	POS	NEG	NEG	NEG	NEG	NEG	POS	NEG	AMB	NEG
MSSA1996	NEG	NEG	POS	AMB	NEG	POS	NEG	NEG	NEG	NEG	NEG	POS	NEG	NEG	NEG
MSSA2002	NEG	NEG	POS	AMB	NEG	POS	NEG	NEG	NEG	NEG	NEG	POS	NEG	NEG	NEG
MSSA2014	NEG	NEG	POS	AMB	NEG	POS	NEG	NEG	NEG	NEG	NEG	POS	NEG	NEG	NEG
MSSA2053	NEG	NEG	POS	AMB	NEG	POS	NEG	NEG	NEG	NEG	NEG	POS	NEG	NEG	NEG
MSSA2054	NEG	NEG	POS	AMB	NEG	POS	NEG	NEG	NEG	NEG	NEG	POS	NEG	NEG	NEG
MSSA2058	NEG	NEG	POS	AMB	NEG	POS	NEG	NEG	NEG	NEG	NEG	POS	NEG	NEG	NEG
MSSA2068	NEG	NEG	POS	POS	AMB	AMB	NEG	NEG	NEG	NEG	NEG	POS	NEG	NEG	NEG
MSSA2070	NEG	NEG	POS	AMB	NEG	POS	AMB	NEG	NEG	NEG	NEG	POS	NEG	NEG	NEG
MSSA2072	NEG	NEG	POS	AMB	NEG	POS	NEG	NEG	NEG	NEG	NEG	POS	NEG	AMB	NEG
MSSA2073	NEG	NEG	POS	AMB	NEG	POS	NEG	NEG	NEG	NEG	NEG	POS	NEG	NEG	NEG
MSSA2074	NEG	NEG	POS	AMB	NEG	POS	NEG	NEG	NEG	NEG	NEG	POS	NEG	POS	NEG
MSSA2107	NEG	NEG	POS	AMB	NEG	POS	NEG	NEG	NEG	NEG	NEG	POS	NEG	AMB	NEG
MSSA2108	NEG	NEG	POS	AMB	NEG	POS	NEG	NEG	NEG	NEG	NEG	POS	NEG	NEG	NEG
MSSA2129	NEG	NEG	POS	AMB	NEG	POS	NEG	NEG	NEG	NEG	NEG	POS	NEG	AMB	NEG
MSSA2135	NEG	NEG	POS	AMB	NEG	POS	NEG	NEG	NEG	NEG	NEG	POS	NEG	AMB	NEG
MSSA2137	NEG	NEG	POS	AMB	NEG	POS	NEG	NEG	NEG	NEG	NEG	POS	NEG	AMB	NEG
MSSA2140	NEG	NEG	POS	AMB	NEG	POS	NEG	NEG	NEG	NEG	NEG	POS	NEG	AMB	NEG
MSSA2182	NEG	NEG	POS	AMB	NEG	POS	NEG	NEG	NEG	NEG	NEG	POS	NEG	NEG	NEG
MSSA2188	NEG	NEG	POS	AMB	NEG	POS	NEG	NEG	NEG	NEG	NEG	POS	NEG	NEG	NEG
MSSA1899	POS	POS	NEG	AMB	POS	NEG	NEG	NEG	NEG	NEG	POS	NEG	POS	NEG	POS
MSSA1915	POS	POS	NEG	AMB	POS	NEG	NEG	NEG	NEG	NEG	POS	NEG	POS	NEG	POS
MSSA1949	POS	POS	NEG	AMB	POS	NEG	NEG	NEG	NEG	NEG	POS	NEG	POS	NEG	POS
MSSA2042	POS	POS	NEG	AMB	POS	NEG	NEG	NEG	NEG	NEG	POS	NEG	POS	NEG	POS
MSSA2077	POS	POS	NEG	AMB	POS	NEG	NEG	NEG	NEG	NEG	POS	NEG	POS	NEG	POS
MSSA2095	POS	POS	NEG	POS	AMB	NEG	NEG	NEG	NEG	NEG	POS	NEG	POS	NEG	POS
MSSA1997	POS	POS	NEG	POS	NEG	AMB	NEG	NEG	NEG	NEG	POS	NEG	POS	NEG	POS
MSSA1810	POS	POS	NEG	POS	NEG	NEG	NEG	NEG	NEG	NEG	POS	NEG	POS	NEG	POS
MSSA1832	POS	POS	NEG	POS	NEG	NEG	NEG	NEG	NEG	NEG	POS	NEG	POS	NEG	POS
MSSA1891	POS	POS	NEG	POS	NEG	AMB	NEG	NEG	NEG	NEG	POS	NEG	POS	NEG	POS
MSSA1892	POS	POS	NEG	POS	NEG	AMB	NEG	NEG	NEG	NEG	POS	NEG	POS	NEG	POS
MSSA1932	POS	POS	NEG	POS	NEG	AMB	NEG	NEG	NEG	NEG	POS	NEG	POS	NEG	POS
MSSA1942	POS	POS	NEG	POS	NEG	AMB	NEG	NEG	NEG	NEG	POS	NEG	POS	NEG	POS
MSSA1957	POS	POS	NEG	POS	NEG	NEG	NEG	NEG	NEG	NEG	POS	NEG	POS	NEG	POS
MSSA1748	POS	POS	NEG	POS	AMB	AMB	NEG	NEG	NEG	NEG	POS	NEG	POS	NEG	POS
MSSA1754	POS	POS	NEG	POS	NEG	AMB	NEG	NEG	NEG	NEG	POS	NEG	POS	NEG	POS
MSSA1770	POS	POS	NEG	POS	NEG	NEG	NEG	NEG	NEG	NEG	POS	NEG	POS	NEG	POS
MSSA1788	POS	POS	NEG	POS	NEG	NEG	NEG	NEG	NEG	NEG	POS	NEG	POS	NEG	POS
MSSA1804	POS	POS	NEG	POS	NEG	NEG	NEG	NEG	NEG	NEG	POS	NEG	POS	NEG	POS
MSSA1818	POS	POS	NEG	POS	AMB	NEG	NEG	NEG	NEG	NEG	POS	NEG	POS	NEG	POS
MSSA1914	POS	POS	NEG	POS	AMB	AMB	NEG	NEG	NEG	NEG	POS	NEG	POS	NEG	POS
MSSA1974	POS	POS	NEG	POS	NEG	NEG	NEG	NEG	NEG	NEG	POS	NEG	POS	NEG	POS
MSSA2023	POS	POS	NEG	POS	NEG	AMB	NEG	NEG	NEG	NEG	POS	NEG	POS	NEG	POS
MSSA2030	POS	POS	NEG	POS	AMB	AMB	NEG	NEG	NEG	NEG	POS	NEG	POS	NEG	POS
MSSA2096	AMB	POS	NEG	POS	NEG	NEG	NEG	NEG	NEG	NEG	POS	NEG	POS	NEG	POS
MSSA2142	POS	POS	NEG	POS	NEG	AMB	NEG	NEG	NEG	NEG	POS	NEG	POS	NEG	POS
MSSA2203	POS	POS	NEG	POS	NEG	NEG	NEG	NEG	NEG	NEG	POS	NEG	POS	NEG	POS
MSSA1901	POS	POS	NEG	AMB	POS	NEG	NEG	NEG	NEG	NEG	POS	NEG	POS	NEG	POS
MSSA2013	POS	POS	NEG	POS	AMB	AMB	NEG	NEG	NEG	NEG	POS	NEG	POS	NEG	POS
MSSA2031	POS	POS	NEG	POS	AMB	AMB	NEG	NEG	NEG	NEG	POS	NEG	POS	NEG	POS
MSSA2052	POS	POS	NEG	POS	AMB	NEG	NEG	NEG	NEG	NEG	POS	NEG	POS	NEG	POS
MSSA2057	POS	POS	NEG	POS	AMB	NEG	NEG	NEG	NEG	NEG	POS	NEG	POS	NEG	POS
MSSA2081	POS	POS	NEG	AMB	POS	NEG	NEG	NEG	NEG	NEG	POS	NEG	POS	NEG	POS
MSSA1955	POS	POS	NEG	AMB	POS	NEG	NEG	NEG	NEG	POS	POS	NEG	POS	NEG	POS
MSSA1941	NEG	NEG	POS	AMB	NEG	POS	NEG	NEG	NEG	NEG	NEG	POS	NEG	NEG	NEG
MRSA3838	NEG	NEG	POS	AMB	NEG	POS	NEG	NEG	NEG	NEG	NEG	POS	NEG	POS	POS
MRSA3839	NEG	NEG	POS	AMB	NEG	POS	NEG	NEG	NEG	AMB	NEG	POS	NEG	POS	POS
MSSA1801	NEG	NEG	POS	AMB	NEG	POS	NEG	NEG	NEG	NEG	NEG	POS	NEG	POS	POS
MSSA2131	NEG	NEG	POS	AMB	AMB	POS	NEG	NEG	NEG	POS	NEG	POS	NEG	POS	POS
MSSA1792	POS	POS	NEG	POS	NEG	NEG	NEG	NEG	NEG	NEG	POS	NEG	POS	NEG	POS
MSSA1796	POS	POS	NEG	POS	AMB	AMB	NEG	NEG	NEG	NEG	POS	NEG	POS	NEG	POS
MSSA1817	POS	POS	NEG	AMB	POS	NEG	NEG	NEG	NEG	NEG	POS	NEG	POS	NEG	POS
MSSA1911	POS	POS	NEG	POS	NEG	NEG	NEG	NEG	NEG	NEG	NEG	NEG	NEG	NEG	NEG
MSSA1927	POS	POS	NEG	POS	AMB	AMB	NEG	POS	NEG	NEG	POS	NEG	POS	NEG	POS
MSSA1928	POS	POS	NEG	POS	AMB	AMB	NEG	POS	NEG	NEG	POS	NEG	POS	NEG	POS
MSSA1953	POS	POS	NEG	AMB	POS	NEG	NEG	POS	NEG	NEG	POS	NEG	POS	NEG	POS
MSSA2062	POS	POS	NEG	POS	NEG	NEG	NEG	NEG	NEG	NEG	POS	NEG	POS	NEG	POS
MSSA2078	NEG	NEG	POS	AMB	NEG	POS	NEG	NEG	NEG	NEG	NEG	POS	NEG	POS	AMB
MSSA2136	POS	POS	NEG	POS	AMB	AMB	NEG	NEG	NEG	NEG	POS	NEG	POS	NEG	POS

	CAPSULE- AND BIOFILM-ASSOCIATED GENES															
**Strain**	**capH1**	**capJ1**	**capK1**	**capH5**	**capJ5** (probe 1)	**capJ5** (probe 2)	**capK5**	**capH8**	**capI8**	**capJ8**	**capK8** (probe 1)	**capK8** (probe 2)	**icaA**	**icaC**	**icaD**	**bap**
MSSA1805	NEG	NEG	NEG	NEG	NEG	NEG	NEG	POS	POS	POS	POS	POS	POS	POS	POS	NEG
MSSA1823	NEG	NEG	NEG	NEG	NEG	NEG	NEG	POS	POS	POS	POS	POS	POS	POS	POS	NEG
MSSA1837	NEG	NEG	NEG	NEG	NEG	NEG	NEG	POS	POS	POS	POS	POS	POS	POS	POS	NEG
MSSA2044	NEG	NEG	NEG	NEG	NEG	NEG	NEG	POS	POS	POS	POS	POS	POS	POS	POS	NEG
MSSA2046	NEG	NEG	NEG	NEG	NEG	NEG	NEG	POS	POS	POS	POS	POS	POS	POS	POS	NEG
MSSA2059	NEG	NEG	NEG	NEG	NEG	NEG	NEG	POS	POS	POS	POS	POS	POS	POS	POS	NEG
MSSA2076	NEG	NEG	NEG	NEG	NEG	NEG	NEG	POS	POS	POS	POS	POS	POS	POS	POS	NEG
MSSA2200	NEG	NEG	NEG	NEG	NEG	NEG	NEG	POS	POS	POS	POS	POS	POS	POS	POS	NEG
MRSA3797	NEG	AMB	NEG	POS	POS	POS	POS	NEG	AMB	NEG	NEG	NEG	POS	POS	POS	NEG
MRSA3798	NEG	NEG	NEG	POS	POS	POS	POS	NEG	NEG	NEG	NEG	NEG	POS	POS	POS	NEG
MRSA3799	NEG	NEG	NEG	POS	POS	POS	POS	NEG	AMB	NEG	NEG	NEG	POS	POS	POS	NEG
MRSA3800	NEG	NEG	NEG	POS	POS	POS	POS	NEG	NEG	NEG	NEG	NEG	POS	POS	POS	NEG
MRSA3801	NEG	NEG	NEG	POS	POS	POS	POS	NEG	NEG	NEG	NEG	NEG	POS	POS	POS	NEG
MRSA3804	NEG	NEG	NEG	POS	POS	POS	POS	NEG	NEG	NEG	NEG	NEG	POS	POS	POS	NEG
MRSA3806	NEG	NEG	NEG	POS	POS	POS	POS	NEG	NEG	NEG	NEG	NEG	POS	POS	POS	NEG
MRSA3961	NEG	NEG	NEG	POS	POS	POS	POS	NEG	NEG	NEG	NEG	NEG	POS	POS	POS	NEG
MRSA3962	NEG	NEG	NEG	POS	POS	POS	POS	NEG	NEG	NEG	NEG	NEG	POS	POS	POS	NEG
MRSA3963	NEG	NEG	NEG	POS	POS	POS	POS	NEG	NEG	NEG	NEG	NEG	POS	POS	POS	NEG
MRSA3966	NEG	NEG	NEG	POS	POS	POS	POS	NEG	NEG	NEG	NEG	NEG	POS	POS	POS	NEG
MRSA3978	NEG	NEG	NEG	POS	POS	POS	POS	NEG	NEG	NEG	NEG	NEG	POS	POS	POS	NEG
MRSA3979	NEG	NEG	NEG	POS	POS	POS	POS	NEG	NEG	NEG	NEG	NEG	POS	POS	POS	NEG
MRSA3980	NEG	NEG	NEG	POS	POS	POS	POS	NEG	NEG	NEG	NEG	NEG	POS	POS	POS	NEG
MRSA4179	NEG	NEG	NEG	POS	POS	POS	POS	NEG	NEG	NEG	NEG	NEG	POS	POS	POS	NEG
MRSA4216	NEG	NEG	NEG	POS	POS	POS	POS	NEG	NEG	NEG	NEG	NEG	POS	POS	POS	NEG
MRSA4217	NEG	NEG	NEG	POS	POS	POS	POS	NEG	NEG	NEG	NEG	NEG	POS	POS	POS	NEG
MRSA4220	NEG	NEG	NEG	POS	POS	POS	POS	NEG	NEG	NEG	NEG	NEG	POS	POS	POS	NEG
MRSA4221	NEG	NEG	NEG	POS	POS	POS	POS	NEG	NEG	NEG	NEG	NEG	POS	POS	POS	NEG
MRSA4222	NEG	NEG	NEG	POS	POS	POS	POS	NEG	NEG	NEG	NEG	NEG	POS	POS	POS	NEG
MSSA1751	NEG	NEG	NEG	POS	POS	POS	POS	NEG	NEG	NEG	NEG	NEG	POS	POS	POS	NEG
MSSA1760	NEG	NEG	NEG	POS	POS	POS	POS	NEG	NEG	NEG	NEG	NEG	POS	POS	POS	NEG
MSSA1802	NEG	AMB	NEG	POS	POS	POS	POS	NEG	POS	NEG	NEG	NEG	POS	POS	POS	NEG
MSSA1809	NEG	NEG	NEG	POS	POS	POS	POS	NEG	POS	NEG	NEG	NEG	POS	POS	POS	NEG
MSSA1815	NEG	NEG	NEG	POS	POS	POS	POS	NEG	AMB	NEG	NEG	NEG	POS	POS	POS	NEG
MSSA1829	NEG	NEG	NEG	POS	POS	POS	POS	NEG	AMB	NEG	NEG	NEG	POS	POS	POS	NEG
MSSA1908	NEG	NEG	NEG	POS	POS	POS	POS	NEG	NEG	NEG	NEG	NEG	POS	POS	POS	NEG
MSSA1960	NEG	AMB	NEG	POS	POS	POS	POS	NEG	AMB	NEG	NEG	NEG	POS	POS	POS	NEG
MSSA1965	NEG	NEG	NEG	POS	POS	POS	POS	NEG	NEG	NEG	NEG	NEG	POS	POS	POS	NEG
MSSA1977	NEG	NEG	NEG	POS	POS	POS	POS	NEG	AMB	NEG	NEG	NEG	POS	POS	POS	NEG
MSSA1978	NEG	NEG	NEG	POS	POS	POS	POS	NEG	NEG	NEG	NEG	NEG	POS	POS	POS	NEG
MSSA1999	NEG	NEG	NEG	POS	POS	POS	POS	NEG	NEG	NEG	NEG	NEG	POS	POS	POS	NEG
MSSA2003	NEG	NEG	NEG	POS	POS	POS	POS	NEG	NEG	NEG	NEG	NEG	POS	POS	POS	NEG
MSSA2006	NEG	NEG	NEG	POS	POS	POS	POS	NEG	NEG	NEG	NEG	NEG	POS	POS	POS	NEG
MSSA2008	NEG	NEG	NEG	POS	POS	POS	POS	NEG	NEG	NEG	NEG	NEG	POS	POS	POS	NEG
MSSA2011	NEG	NEG	NEG	POS	POS	POS	POS	NEG	AMB	NEG	NEG	NEG	POS	POS	POS	NEG
MSSA2017	NEG	NEG	NEG	POS	POS	POS	POS	NEG	NEG	NEG	NEG	NEG	POS	POS	POS	NEG
MSSA2022	NEG	NEG	NEG	POS	POS	POS	POS	NEG	AMB	NEG	NEG	NEG	POS	POS	POS	NEG
MSSA2055	NEG	NEG	NEG	POS	POS	POS	POS	NEG	NEG	NEG	NEG	NEG	POS	POS	POS	NEG
MSSA2060	NEG	NEG	NEG	POS	POS	POS	POS	NEG	NEG	NEG	NEG	NEG	POS	POS	POS	NEG
MSSA2080	NEG	NEG	NEG	POS	POS	POS	POS	NEG	NEG	NEG	NEG	NEG	POS	POS	POS	NEG
MSSA2082	NEG	NEG	NEG	POS	POS	POS	POS	NEG	NEG	NEG	NEG	NEG	POS	POS	POS	NEG
MSSA2084	NEG	NEG	NEG	POS	POS	POS	POS	NEG	NEG	NEG	NEG	NEG	POS	POS	POS	NEG
MSSA2087	NEG	NEG	NEG	POS	POS	POS	POS	NEG	NEG	NEG	NEG	NEG	POS	POS	POS	NEG
MSSA2094	NEG	NEG	NEG	POS	POS	POS	POS	NEG	NEG	NEG	NEG	NEG	POS	POS	POS	NEG
MSSA2097	NEG	NEG	NEG	POS	POS	POS	POS	NEG	NEG	NEG	NEG	NEG	POS	POS	POS	NEG
MSSA2110	NEG	NEG	NEG	POS	POS	POS	POS	NEG	NEG	NEG	NEG	NEG	POS	POS	POS	NEG
MSSA2113	NEG	NEG	NEG	POS	POS	POS	POS	NEG	NEG	NEG	NEG	NEG	POS	POS	POS	NEG
MSSA2132	NEG	NEG	NEG	POS	POS	POS	POS	NEG	NEG	NEG	NEG	NEG	POS	POS	POS	NEG
MSSA2134	NEG	NEG	NEG	POS	POS	POS	POS	NEG	NEG	NEG	NEG	NEG	POS	POS	POS	NEG
MSSA1746	NEG	NEG	NEG	NEG	NEG	NEG	NEG	POS	POS	POS	POS	POS	POS	POS	POS	NEG
MSSA1757	NEG	NEG	NEG	NEG	NEG	NEG	NEG	POS	POS	POS	POS	POS	POS	POS	POS	NEG
MSSA1772	NEG	NEG	NEG	NEG	NEG	NEG	NEG	POS	POS	POS	POS	POS	POS	POS	POS	NEG
MSSA1795	NEG	NEG	NEG	NEG	NEG	NEG	NEG	POS	POS	POS	POS	POS	POS	POS	POS	NEG
MSSA1813	NEG	AMB	NEG	NEG	NEG	NEG	NEG	POS	POS	POS	POS	POS	POS	POS	POS	NEG
MSSA1826	NEG	NEG	NEG	NEG	NEG	NEG	NEG	POS	POS	POS	POS	POS	POS	POS	POS	NEG
MSSA1830	NEG	NEG	NEG	NEG	NEG	NEG	NEG	POS	POS	POS	POS	POS	POS	POS	POS	NEG
MSSA1838	NEG	NEG	NEG	NEG	NEG	NEG	NEG	POS	POS	POS	POS	POS	POS	POS	POS	NEG
MSSA1944	NEG	NEG	NEG	NEG	NEG	NEG	NEG	POS	POS	POS	POS	POS	POS	POS	POS	NEG
MSSA1947	NEG	NEG	NEG	NEG	NEG	NEG	NEG	POS	POS	POS	POS	POS	POS	POS	POS	NEG
MSSA1964	NEG	NEG	NEG	NEG	NEG	NEG	NEG	POS	POS	POS	POS	POS	POS	POS	POS	NEG
MSSA1969	NEG	NEG	NEG	NEG	NEG	NEG	NEG	POS	POS	POS	POS	POS	POS	POS	POS	NEG
MSSA2001	NEG	NEG	NEG	NEG	NEG	NEG	NEG	POS	POS	POS	POS	POS	POS	POS	POS	NEG
MSSA2004	NEG	NEG	NEG	NEG	NEG	NEG	NEG	POS	POS	POS	POS	POS	POS	POS	POS	NEG
MSSA2024	NEG	NEG	NEG	NEG	NEG	NEG	NEG	POS	POS	POS	POS	POS	POS	POS	POS	NEG
MSSA2025	NEG	NEG	NEG	NEG	NEG	NEG	NEG	POS	POS	POS	POS	POS	POS	POS	POS	NEG
MSSA2026	NEG	NEG	NEG	NEG	NEG	NEG	NEG	POS	POS	POS	POS	POS	POS	POS	POS	NEG
MSSA2032	NEG	NEG	NEG	NEG	NEG	NEG	NEG	POS	POS	POS	POS	POS	POS	POS	POS	NEG
MSSA2043	NEG	NEG	NEG	NEG	NEG	NEG	NEG	POS	POS	POS	POS	POS	POS	POS	POS	NEG
MSSA2061	NEG	NEG	NEG	NEG	NEG	NEG	NEG	POS	POS	POS	POS	POS	POS	POS	POS	NEG
MSSA2085	NEG	NEG	NEG	NEG	NEG	NEG	NEG	POS	POS	POS	POS	POS	POS	POS	POS	NEG
MSSA2098	NEG	NEG	NEG	NEG	NEG	NEG	NEG	POS	POS	POS	POS	POS	POS	POS	POS	NEG
MSSA2100	NEG	NEG	NEG	NEG	NEG	NEG	NEG	POS	POS	POS	POS	POS	POS	POS	POS	NEG
MSSA2111	NEG	NEG	NEG	NEG	NEG	NEG	NEG	POS	POS	POS	POS	POS	POS	POS	POS	NEG
MSSA2178	NEG	NEG	NEG	NEG	NEG	NEG	NEG	POS	POS	POS	POS	POS	POS	POS	POS	NEG
MSSA2202	NEG	NEG	NEG	NEG	NEG	NEG	NEG	POS	POS	POS	POS	POS	POS	POS	POS	NEG
MSSA2205	NEG	NEG	NEG	NEG	NEG	NEG	NEG	POS	POS	POS	POS	POS	POS	POS	POS	NEG
MRSA3796	NEG	NEG	NEG	POS	POS	POS	POS	NEG	NEG	NEG	NEG	NEG	POS	POS	POS	NEG
MRSA3805	NEG	NEG	NEG	NEG	NEG	NEG	NEG	POS	POS	POS	POS	POS	POS	POS	POS	NEG
MRSA3965	NEG	NEG	NEG	POS	POS	POS	POS	NEG	NEG	NEG	NEG	NEG	POS	POS	POS	NEG
MSSA1761	NEG	NEG	NEG	POS	POS	POS	POS	NEG	NEG	NEG	NEG	NEG	POS	POS	POS	NEG
MSSA1764	NEG	NEG	NEG	POS	POS	POS	POS	NEG	NEG	NEG	NEG	NEG	POS	POS	POS	NEG
MSSA1765	NEG	NEG	NEG	POS	POS	POS	POS	NEG	NEG	NEG	NEG	NEG	POS	POS	POS	NEG
MSSA1784	NEG	NEG	NEG	POS	POS	POS	POS	NEG	NEG	NEG	NEG	NEG	POS	POS	POS	NEG
MSSA1806	NEG	NEG	NEG	POS	POS	POS	POS	NEG	NEG	NEG	NEG	NEG	POS	POS	POS	NEG
MSSA1807	NEG	NEG	NEG	POS	POS	POS	POS	NEG	AMB	NEG	NEG	NEG	POS	POS	POS	NEG
MSSA1888	NEG	NEG	NEG	POS	POS	POS	POS	NEG	AMB	NEG	NEG	NEG	POS	POS	POS	NEG
MSSA1894	NEG	NEG	NEG	POS	POS	POS	POS	NEG	NEG	NEG	NEG	NEG	POS	POS	POS	NEG
MSSA1922	NEG	NEG	NEG	POS	POS	POS	POS	NEG	NEG	NEG	NEG	NEG	POS	POS	POS	NEG
MSSA1950	NEG	NEG	NEG	POS	POS	POS	POS	NEG	NEG	NEG	NEG	NEG	POS	POS	POS	NEG
MSSA1954	NEG	NEG	NEG	POS	POS	POS	POS	NEG	AMB	NEG	NEG	NEG	POS	POS	POS	NEG
MSSA1966	NEG	NEG	NEG	POS	POS	POS	POS	NEG	NEG	NEG	NEG	NEG	POS	POS	POS	NEG
MSSA1972	NEG	NEG	NEG	POS	POS	POS	POS	NEG	NEG	NEG	NEG	NEG	POS	POS	POS	NEG
MSSA2010	NEG	NEG	NEG	POS	POS	POS	POS	NEG	NEG	NEG	NEG	NEG	POS	POS	POS	NEG
MSSA2020	NEG	NEG	NEG	POS	POS	POS	POS	NEG	NEG	NEG	NEG	NEG	POS	POS	POS	NEG
MSSA2028	NEG	NEG	NEG	POS	POS	POS	POS	NEG	AMB	NEG	NEG	NEG	POS	POS	POS	NEG
MSSA2088	NEG	NEG	NEG	POS	POS	POS	POS	NEG	NEG	NEG	NEG	NEG	POS	POS	POS	NEG
MSSA2187	NEG	NEG	NEG	POS	POS	POS	POS	NEG	NEG	NEG	NEG	NEG	POS	POS	POS	NEG
MSSA2204	NEG	NEG	NEG	POS	POS	POS	POS	NEG	NEG	NEG	NEG	NEG	POS	POS	POS	NEG
MSSA2210	NEG	NEG	NEG	POS	POS	POS	POS	NEG	NEG	NEG	NEG	NEG	POS	POS	POS	NEG
MSSA2223	NEG	NEG	NEG	POS	POS	POS	POS	NEG	NEG	NEG	NEG	NEG	POS	POS	POS	NEG
MSSA1763	NEG	NEG	NEG	POS	POS	POS	POS	NEG	NEG	NEG	NEG	NEG	POS	POS	POS	NEG
MSSA1777	NEG	NEG	NEG	POS	POS	POS	POS	NEG	NEG	NEG	NEG	NEG	POS	POS	POS	NEG
MSSA2009	NEG	NEG	NEG	POS	POS	POS	POS	NEG	NEG	NEG	NEG	NEG	POS	POS	POS	NEG
MSSA2047	NEG	NEG	NEG	POS	POS	POS	POS	NEG	NEG	NEG	NEG	NEG	POS	POS	POS	NEG
MSSA2079	NEG	NEG	NEG	POS	POS	POS	POS	NEG	NEG	NEG	NEG	NEG	POS	POS	POS	NEG
MSSA2101	NEG	NEG	NEG	POS	POS	POS	POS	NEG	NEG	NEG	NEG	NEG	POS	POS	POS	NEG
MSSA1821	NEG	NEG	NEG	NEG	NEG	NEG	NEG	POS	POS	POS	POS	POS	POS	POS	POS	NEG
MSSA1898	NEG	NEG	NEG	NEG	NEG	NEG	NEG	POS	POS	POS	POS	POS	POS	POS	POS	NEG
MSSA1973	NEG	NEG	NEG	NEG	NEG	NEG	NEG	POS	POS	POS	POS	POS	POS	POS	POS	NEG
MSSA2071	NEG	NEG	NEG	NEG	NEG	NEG	NEG	POS	POS	POS	POS	POS	POS	POS	POS	NEG
MSSA2083	NEG	NEG	NEG	NEG	NEG	NEG	NEG	POS	POS	POS	POS	POS	POS	POS	POS	NEG
MSSA2106	NEG	NEG	NEG	NEG	NEG	NEG	NEG	POS	POS	POS	POS	POS	POS	POS	POS	NEG
MSSA2112	NEG	NEG	NEG	NEG	NEG	NEG	NEG	POS	POS	POS	POS	POS	POS	POS	POS	NEG
MSSA2115	NEG	NEG	NEG	NEG	NEG	NEG	NEG	POS	POS	POS	POS	POS	POS	POS	POS	NEG
MSSA2195	NEG	NEG	NEG	NEG	NEG	NEG	NEG	POS	POS	POS	POS	POS	POS	POS	POS	NEG
MSSA2207	NEG	NEG	NEG	NEG	NEG	NEG	NEG	POS	POS	POS	POS	POS	POS	POS	POS	NEG
MSSA1758	NEG	NEG	NEG	NEG	NEG	NEG	NEG	POS	POS	POS	POS	POS	POS	POS	POS	NEG
MSSA1762	NEG	NEG	NEG	NEG	NEG	NEG	NEG	POS	POS	POS	POS	POS	POS	POS	POS	NEG
MSSA1769	NEG	NEG	NEG	NEG	NEG	NEG	NEG	POS	POS	POS	POS	POS	POS	POS	POS	NEG
MSSA1771	NEG	NEG	NEG	NEG	NEG	NEG	NEG	POS	POS	POS	POS	POS	POS	POS	POS	NEG
MSSA1789	NEG	NEG	NEG	NEG	NEG	NEG	NEG	POS	POS	POS	POS	POS	POS	POS	POS	NEG
MSSA1800	NEG	NEG	NEG	NEG	NEG	NEG	NEG	POS	POS	POS	POS	POS	POS	POS	POS	NEG
MSSA1833	NEG	NEG	NEG	NEG	NEG	NEG	NEG	POS	POS	POS	POS	POS	POS	POS	POS	NEG
MSSA1890	NEG	NEG	NEG	NEG	NEG	NEG	NEG	POS	POS	POS	POS	POS	POS	POS	POS	NEG
MSSA1903	NEG	NEG	NEG	NEG	NEG	NEG	NEG	POS	POS	POS	POS	POS	POS	POS	POS	NEG
MSSA1940	NEG	NEG	NEG	NEG	NEG	NEG	NEG	POS	POS	POS	POS	POS	POS	POS	POS	NEG
MSSA1962	NEG	NEG	NEG	NEG	NEG	NEG	NEG	POS	POS	POS	POS	POS	POS	POS	POS	NEG
MSSA1963	NEG	NEG	NEG	NEG	NEG	NEG	NEG	POS	POS	POS	POS	POS	POS	POS	POS	NEG
MSSA2016	NEG	NEG	NEG	NEG	NEG	NEG	NEG	POS	POS	POS	POS	POS	POS	POS	POS	NEG
MSSA2018	NEG	NEG	NEG	NEG	NEG	NEG	NEG	POS	POS	POS	POS	POS	POS	POS	POS	NEG
MSSA2019	NEG	NEG	NEG	NEG	NEG	NEG	NEG	POS	POS	POS	POS	POS	POS	POS	POS	NEG
MSSA2029	NEG	NEG	NEG	NEG	NEG	NEG	NEG	POS	POS	POS	POS	POS	POS	POS	POS	NEG
MSSA2045	NEG	NEG	NEG	NEG	NEG	NEG	NEG	POS	POS	POS	POS	POS	POS	POS	POS	NEG
MSSA2048	NEG	NEG	NEG	NEG	NEG	NEG	NEG	POS	POS	POS	POS	POS	POS	POS	POS	NEG
MSSA2049	NEG	NEG	NEG	NEG	NEG	NEG	NEG	POS	POS	POS	POS	POS	POS	POS	POS	NEG
MSSA2050	NEG	NEG	NEG	NEG	NEG	NEG	NEG	POS	POS	POS	POS	POS	POS	POS	POS	NEG
MSSA2099	NEG	NEG	NEG	NEG	NEG	NEG	NEG	POS	POS	POS	POS	POS	POS	POS	POS	NEG
MSSA2109	NEG	NEG	NEG	NEG	NEG	NEG	NEG	POS	POS	POS	POS	POS	POS	POS	POS	NEG
MSSA2130	NEG	NEG	NEG	NEG	NEG	NEG	NEG	POS	POS	POS	POS	POS	POS	POS	POS	NEG
MSSA2133	NEG	NEG	NEG	NEG	NEG	NEG	NEG	POS	POS	POS	POS	POS	POS	POS	POS	NEG
MSSA2139	NEG	NEG	NEG	NEG	NEG	NEG	NEG	POS	POS	POS	POS	POS	POS	POS	POS	NEG
MSSA2141	NEG	NEG	NEG	NEG	NEG	NEG	NEG	POS	POS	POS	POS	POS	POS	POS	POS	NEG
MSSA2208	NEG	NEG	NEG	NEG	NEG	NEG	NEG	POS	POS	POS	POS	POS	POS	POS	POS	NEG
MSSA2209	NEG	NEG	NEG	NEG	NEG	NEG	NEG	POS	POS	POS	POS	POS	POS	POS	POS	NEG
MSSA1893	NEG	NEG	NEG	POS	POS	POS	POS	NEG	AMB	NEG	NEG	NEG	POS	POS	POS	NEG
MSSA2105	NEG	NEG	NEG	POS	POS	POS	POS	NEG	NEG	NEG	NEG	NEG	POS	POS	POS	NEG
MRSA3836	NEG	NEG	NEG	POS	POS	POS	POS	NEG	AMB	NEG	NEG	NEG	POS	POS	POS	NEG
MRSA3840	NEG	NEG	NEG	POS	POS	POS	POS	NEG	AMB	NEG	NEG	NEG	POS	POS	POS	NEG
MRSA4188	NEG	NEG	NEG	POS	POS	POS	POS	NEG	NEG	NEG	NEG	NEG	POS	POS	POS	NEG
MSSA1814	NEG	AMB	NEG	POS	POS	POS	POS	NEG	AMB	NEG	NEG	NEG	POS	POS	POS	NEG
MSSA1816	NEG	NEG	NEG	POS	POS	POS	POS	NEG	NEG	NEG	NEG	NEG	POS	POS	POS	NEG
MSSA1825	NEG	AMB	NEG	POS	POS	POS	POS	NEG	AMB	NEG	NEG	NEG	POS	POS	POS	NEG
MSSA1834	NEG	NEG	NEG	POS	POS	POS	POS	NEG	NEG	NEG	NEG	NEG	POS	POS	POS	NEG
MSSA1938	NEG	NEG	NEG	POS	POS	POS	POS	NEG	NEG	NEG	NEG	NEG	POS	POS	POS	NEG
MSSA1948	NEG	NEG	NEG	POS	POS	POS	POS	NEG	NEG	NEG	NEG	NEG	POS	POS	POS	NEG
MSSA1951	NEG	NEG	NEG	POS	POS	POS	POS	NEG	NEG	NEG	NEG	NEG	POS	POS	POS	NEG
MSSA2005	NEG	NEG	NEG	POS	POS	POS	POS	NEG	NEG	NEG	NEG	NEG	POS	POS	POS	NEG
MSSA2051	NEG	NEG	NEG	POS	POS	POS	POS	NEG	NEG	NEG	NEG	NEG	POS	POS	POS	NEG
MSSA2056	NEG	NEG	NEG	POS	POS	POS	POS	NEG	AMB	NEG	NEG	NEG	POS	POS	POS	NEG
MSSA2066	NEG	NEG	NEG	POS	POS	POS	POS	NEG	NEG	NEG	NEG	NEG	POS	POS	POS	NEG
MSSA2067	NEG	NEG	NEG	POS	POS	POS	POS	NEG	NEG	NEG	NEG	NEG	POS	POS	POS	NEG
MSSA2086	NEG	NEG	NEG	POS	POS	POS	POS	NEG	NEG	NEG	NEG	NEG	POS	POS	POS	NEG
MSSA2089	NEG	NEG	NEG	POS	POS	POS	POS	NEG	NEG	NEG	NEG	NEG	POS	POS	POS	NEG
MSSA2103	NEG	NEG	NEG	POS	POS	POS	POS	NEG	NEG	NEG	NEG	NEG	POS	POS	POS	NEG
MSSA2173	NEG	NEG	NEG	POS	POS	POS	POS	NEG	NEG	NEG	NEG	NEG	POS	POS	POS	NEG
MSSA2183	NEG	NEG	NEG	POS	POS	POS	POS	NEG	NEG	NEG	NEG	NEG	POS	POS	POS	NEG
MSSA2196	NEG	NEG	NEG	POS	POS	POS	POS	NEG	NEG	NEG	NEG	NEG	POS	POS	POS	NEG
MSSA2219	NEG	NEG	NEG	POS	POS	POS	POS	NEG	NEG	NEG	NEG	NEG	POS	POS	POS	NEG
MSSA1753	NEG	NEG	NEG	POS	POS	POS	POS	NEG	NEG	NEG	NEG	NEG	POS	POS	POS	NEG
MSSA1773	NEG	NEG	NEG	POS	POS	POS	POS	NEG	NEG	NEG	NEG	NEG	POS	POS	POS	NEG
MSSA1811	NEG	NEG	NEG	POS	POS	POS	POS	NEG	POS	NEG	NEG	NEG	POS	POS	POS	NEG
MSSA1897	NEG	NEG	NEG	POS	POS	POS	POS	NEG	NEG	NEG	NEG	NEG	POS	POS	POS	NEG
MSSA1931	NEG	NEG	NEG	POS	POS	POS	POS	NEG	NEG	NEG	NEG	NEG	POS	POS	POS	NEG
MSSA2114	NEG	NEG	NEG	POS	POS	POS	POS	NEG	NEG	NEG	NEG	NEG	POS	POS	POS	NEG
MSSA1745	NEG	NEG	NEG	NEG	NEG	NEG	NEG	POS	POS	POS	POS	POS	POS	POS	POS	NEG
MSSA1747	NEG	NEG	NEG	NEG	NEG	NEG	NEG	POS	POS	POS	POS	POS	POS	POS	POS	NEG
MSSA1749	NEG	NEG	NEG	NEG	NEG	NEG	NEG	POS	POS	POS	POS	POS	POS	POS	POS	NEG
MSSA1768	NEG	NEG	NEG	NEG	NEG	NEG	NEG	POS	POS	POS	POS	POS	POS	POS	POS	NEG
MSSA1774	NEG	NEG	NEG	NEG	NEG	NEG	NEG	POS	POS	POS	POS	POS	POS	POS	POS	NEG
MSSA1775	NEG	NEG	NEG	NEG	NEG	NEG	NEG	POS	POS	POS	POS	POS	POS	POS	POS	NEG
MSSA1776	NEG	NEG	NEG	NEG	NEG	NEG	NEG	POS	POS	POS	POS	POS	POS	POS	POS	NEG
MSSA1779	NEG	NEG	NEG	NEG	NEG	NEG	NEG	POS	POS	POS	POS	POS	POS	POS	POS	NEG
MSSA1780	NEG	NEG	NEG	NEG	NEG	NEG	NEG	POS	POS	POS	POS	POS	POS	POS	POS	NEG

	CAPSULE- AND BIOFILM-ASSOCIATED GENES															
Strain	capH1	capJ1	capK1	capH5	capJ5 (probe 1)	capJ5 (probe 2)	capK5	capH8	capI8	capJ8	capK8 (probe 1)	capK8 (probe 2)	icaA	icaC	icaD	bap
MSSA1781	NEG	NEG	NEG	NEG	NEG	NEG	NEG	POS	POS	POS	POS	POS	POS	POS	POS	NEG
MSSA1786	NEG	NEG	NEG	NEG	NEG	NEG	NEG	POS	POS	POS	POS	POS	POS	POS	POS	NEG
MSSA1793	NEG	NEG	NEG	NEG	NEG	NEG	NEG	POS	POS	POS	POS	POS	POS	POS	POS	NEG
MSSA1803	NEG	NEG	NEG	NEG	NEG	NEG	NEG	POS	POS	POS	POS	POS	POS	POS	POS	NEG
MSSA1836	NEG	NEG	NEG	NEG	NEG	NEG	NEG	POS	POS	POS	POS	POS	POS	POS	POS	NEG
MSSA1839	NEG	NEG	NEG	NEG	NEG	NEG	NEG	POS	POS	POS	POS	POS	POS	POS	POS	NEG
MSSA1900	NEG	NEG	NEG	NEG	NEG	NEG	NEG	POS	POS	POS	POS	POS	POS	POS	POS	NEG
MSSA1910	NEG	NEG	NEG	NEG	NEG	NEG	NEG	POS	POS	POS	POS	POS	POS	POS	POS	NEG
MSSA1918	NEG	NEG	NEG	NEG	NEG	NEG	NEG	POS	POS	POS	POS	POS	POS	POS	POS	NEG
MSSA1921	NEG	NEG	NEG	NEG	NEG	NEG	NEG	POS	POS	POS	POS	POS	POS	POS	POS	NEG
MSSA1923	NEG	NEG	NEG	NEG	NEG	NEG	NEG	POS	POS	POS	POS	POS	POS	POS	POS	NEG
MSSA1981	NEG	NEG	NEG	NEG	NEG	NEG	NEG	POS	POS	POS	POS	POS	POS	POS	POS	NEG
MSSA1998	NEG	NEG	NEG	NEG	NEG	NEG	NEG	POS	POS	POS	POS	POS	POS	POS	POS	NEG
MSSA2000	NEG	NEG	NEG	NEG	NEG	NEG	NEG	POS	POS	POS	POS	POS	POS	POS	POS	NEG
MSSA2007	NEG	NEG	NEG	NEG	NEG	NEG	NEG	POS	POS	POS	POS	POS	POS	POS	POS	NEG
MSSA2012	NEG	NEG	NEG	NEG	NEG	NEG	NEG	POS	POS	POS	POS	POS	POS	POS	POS	NEG
MSSA2015	NEG	NEG	NEG	NEG	NEG	NEG	NEG	POS	POS	POS	POS	POS	POS	POS	POS	NEG
MSSA2021	NEG	NEG	NEG	NEG	NEG	NEG	NEG	POS	POS	POS	POS	POS	POS	POS	POS	NEG
MSSA2027	NEG	NEG	NEG	NEG	NEG	NEG	NEG	POS	POS	POS	POS	POS	POS	POS	POS	NEG
MSSA2063	NEG	NEG	NEG	NEG	NEG	NEG	NEG	POS	POS	POS	POS	POS	POS	POS	POS	AMB
MSSA2064	NEG	NEG	NEG	NEG	NEG	NEG	NEG	POS	POS	POS	POS	POS	POS	POS	POS	NEG
MSSA2065	NEG	NEG	NEG	NEG	NEG	NEG	NEG	POS	POS	POS	POS	POS	NEG	NEG	NEG	NEG
MSSA2075	NEG	NEG	NEG	NEG	NEG	NEG	NEG	POS	POS	POS	POS	POS	POS	POS	POS	NEG
MSSA2093	NEG	NEG	NEG	NEG	NEG	NEG	NEG	POS	POS	POS	POS	POS	POS	POS	POS	NEG
MSSA2102	NEG	NEG	NEG	NEG	NEG	NEG	NEG	POS	POS	POS	POS	POS	POS	POS	POS	NEG
MSSA2138	NEG	NEG	NEG	NEG	NEG	NEG	NEG	POS	POS	POS	POS	POS	POS	POS	POS	NEG
MSSA2168	NEG	NEG	NEG	NEG	NEG	NEG	NEG	POS	POS	POS	POS	POS	POS	POS	POS	NEG
MSSA2181	NEG	NEG	NEG	NEG	NEG	NEG	NEG	POS	POS	POS	POS	POS	POS	POS	POS	NEG
MSSA2189	NEG	NEG	NEG	NEG	NEG	NEG	NEG	POS	POS	POS	POS	POS	POS	POS	POS	NEG
MSSA2214	NEG	NEG	NEG	NEG	NEG	NEG	NEG	POS	POS	POS	POS	POS	POS	POS	POS	NEG
MRSA3803	NEG	NEG	NEG	NEG	NEG	NEG	NEG	POS	POS	POS	POS	POS	POS	POS	POS	NEG
MSSA1750	NEG	NEG	NEG	NEG	NEG	NEG	NEG	POS	POS	POS	POS	POS	POS	POS	POS	NEG
MSSA1756	NEG	NEG	NEG	NEG	NEG	NEG	NEG	POS	POS	POS	POS	POS	POS	POS	POS	NEG
MSSA1759	NEG	NEG	NEG	NEG	NEG	NEG	NEG	POS	POS	POS	POS	POS	POS	POS	POS	NEG
MSSA1767	NEG	NEG	NEG	NEG	NEG	NEG	NEG	POS	POS	POS	POS	POS	POS	POS	POS	NEG
MSSA1787	NEG	NEG	NEG	NEG	NEG	NEG	NEG	POS	POS	POS	POS	POS	POS	POS	POS	NEG
MSSA1790	NEG	NEG	NEG	NEG	NEG	NEG	NEG	POS	POS	POS	POS	POS	POS	POS	POS	NEG
MSSA1794	NEG	NEG	NEG	NEG	NEG	NEG	NEG	POS	POS	POS	POS	POS	POS	POS	POS	NEG
MSSA1808	NEG	AMB	NEG	NEG	NEG	NEG	NEG	POS	POS	POS	POS	POS	POS	POS	POS	NEG
MSSA1812	NEG	AMB	NEG	NEG	NEG	NEG	NEG	POS	POS	POS	POS	POS	POS	POS	POS	NEG
MSSA1819	NEG	NEG	NEG	NEG	NEG	NEG	NEG	POS	POS	POS	POS	POS	POS	POS	POS	NEG
MSSA1824	NEG	NEG	NEG	NEG	NEG	NEG	NEG	POS	POS	POS	POS	POS	POS	POS	POS	NEG
MSSA1827	NEG	NEG	NEG	NEG	NEG	NEG	NEG	POS	POS	POS	POS	POS	POS	POS	POS	NEG
MSSA1889	NEG	NEG	NEG	NEG	NEG	NEG	NEG	POS	POS	POS	POS	POS	POS	POS	POS	NEG
MSSA1895	NEG	NEG	NEG	NEG	NEG	NEG	NEG	POS	POS	POS	POS	POS	POS	POS	POS	NEG
MSSA1904	NEG	NEG	NEG	NEG	NEG	NEG	NEG	POS	POS	POS	POS	POS	POS	POS	POS	NEG
MSSA1905	NEG	NEG	NEG	NEG	NEG	NEG	NEG	POS	POS	POS	POS	POS	POS	POS	POS	NEG
MSSA1907	NEG	NEG	NEG	NEG	NEG	NEG	NEG	POS	POS	POS	POS	POS	POS	POS	POS	NEG
MSSA1919	NEG	NEG	NEG	NEG	NEG	NEG	NEG	POS	POS	POS	POS	POS	POS	POS	POS	NEG
MSSA1920	NEG	NEG	NEG	NEG	NEG	NEG	NEG	POS	POS	POS	POS	POS	POS	POS	POS	NEG
MSSA1929	NEG	NEG	NEG	NEG	NEG	NEG	NEG	POS	POS	POS	POS	POS	POS	POS	POS	NEG
MSSA1930	NEG	NEG	NEG	NEG	NEG	NEG	NEG	POS	POS	POS	POS	POS	POS	POS	POS	NEG
MSSA1934	NEG	NEG	NEG	NEG	NEG	NEG	NEG	POS	POS	POS	POS	POS	POS	POS	POS	NEG
MSSA1946	NEG	NEG	NEG	NEG	NEG	NEG	NEG	POS	POS	POS	POS	POS	POS	POS	POS	NEG
MSSA1971	NEG	NEG	NEG	NEG	NEG	NEG	NEG	POS	POS	POS	POS	POS	POS	POS	POS	NEG
MSSA1980	NEG	NEG	NEG	NEG	NEG	NEG	NEG	POS	POS	POS	POS	POS	POS	POS	POS	NEG
MSSA1982	NEG	NEG	NEG	NEG	NEG	NEG	NEG	POS	POS	POS	POS	POS	POS	POS	POS	NEG
MSSA1996	NEG	NEG	NEG	NEG	NEG	NEG	NEG	POS	POS	POS	POS	POS	POS	POS	POS	NEG
MSSA2002	NEG	NEG	NEG	NEG	NEG	NEG	NEG	POS	POS	POS	POS	POS	POS	POS	POS	NEG
MSSA2014	NEG	NEG	NEG	NEG	NEG	NEG	NEG	POS	POS	POS	POS	POS	POS	POS	POS	NEG
MSSA2053	NEG	NEG	NEG	NEG	NEG	NEG	NEG	POS	POS	POS	POS	POS	POS	POS	POS	NEG
MSSA2054	NEG	NEG	NEG	NEG	NEG	NEG	NEG	POS	POS	POS	POS	POS	POS	POS	POS	NEG
MSSA2058	NEG	NEG	NEG	NEG	NEG	NEG	NEG	POS	POS	POS	POS	POS	POS	POS	POS	NEG
MSSA2068	NEG	NEG	NEG	NEG	NEG	NEG	NEG	POS	POS	POS	POS	POS	POS	POS	POS	NEG
MSSA2070	NEG	NEG	NEG	NEG	NEG	NEG	NEG	POS	POS	POS	POS	POS	POS	POS	POS	NEG
MSSA2072	NEG	NEG	NEG	NEG	NEG	NEG	NEG	POS	POS	POS	POS	POS	POS	POS	POS	NEG
MSSA2073	NEG	NEG	NEG	NEG	NEG	NEG	NEG	POS	POS	POS	POS	POS	POS	POS	POS	NEG
MSSA2074	NEG	NEG	NEG	NEG	NEG	NEG	NEG	POS	POS	POS	POS	POS	POS	POS	POS	NEG
MSSA2107	NEG	NEG	NEG	NEG	NEG	NEG	NEG	POS	POS	POS	POS	POS	POS	POS	POS	NEG
MSSA2108	NEG	NEG	NEG	NEG	NEG	NEG	NEG	POS	POS	POS	POS	POS	POS	POS	POS	NEG
MSSA2129	NEG	NEG	NEG	NEG	NEG	NEG	NEG	POS	POS	POS	POS	POS	POS	POS	POS	NEG
MSSA2135	NEG	NEG	NEG	NEG	NEG	NEG	NEG	POS	POS	POS	POS	POS	POS	POS	POS	NEG
MSSA2137	NEG	NEG	NEG	NEG	NEG	NEG	NEG	POS	POS	POS	POS	POS	POS	POS	POS	NEG
MSSA2140	NEG	NEG	NEG	NEG	NEG	NEG	NEG	POS	POS	POS	POS	POS	POS	POS	POS	NEG
MSSA2182	NEG	NEG	NEG	NEG	NEG	NEG	NEG	POS	POS	POS	POS	POS	POS	POS	POS	NEG
MSSA2188	NEG	NEG	NEG	NEG	NEG	NEG	NEG	POS	POS	POS	POS	POS	POS	POS	POS	NEG
MSSA1899	NEG	NEG	NEG	NEG	NEG	NEG	NEG	POS	POS	POS	POS	POS	POS	POS	POS	NEG
MSSA1915	NEG	NEG	NEG	NEG	NEG	NEG	NEG	POS	POS	POS	POS	POS	POS	POS	POS	NEG
MSSA1949	NEG	NEG	NEG	NEG	NEG	NEG	NEG	POS	POS	POS	POS	POS	POS	POS	POS	NEG
MSSA2042	NEG	NEG	NEG	NEG	NEG	NEG	NEG	POS	POS	POS	POS	POS	POS	POS	POS	NEG
MSSA2077	NEG	NEG	NEG	NEG	NEG	NEG	NEG	POS	POS	POS	POS	POS	POS	POS	POS	NEG
MSSA2095	NEG	NEG	NEG	NEG	NEG	NEG	NEG	POS	POS	POS	POS	POS	POS	POS	POS	NEG
MSSA1997	NEG	NEG	NEG	NEG	NEG	NEG	NEG	POS	POS	POS	POS	POS	POS	POS	POS	NEG
MSSA1810	NEG	NEG	NEG	POS	POS	POS	POS	NEG	NEG	NEG	NEG	NEG	POS	POS	POS	NEG
MSSA1832	NEG	NEG	NEG	POS	POS	POS	POS	NEG	AMB	NEG	NEG	NEG	POS	POS	POS	NEG
MSSA1891	NEG	NEG	NEG	POS	POS	POS	POS	NEG	NEG	NEG	NEG	NEG	POS	POS	POS	NEG
MSSA1892	NEG	NEG	NEG	POS	POS	POS	POS	NEG	NEG	NEG	NEG	NEG	POS	POS	POS	NEG
MSSA1932	NEG	NEG	NEG	POS	POS	POS	POS	NEG	NEG	NEG	NEG	NEG	POS	POS	POS	NEG
MSSA1942	NEG	NEG	NEG	POS	POS	POS	POS	NEG	NEG	NEG	NEG	NEG	POS	POS	POS	NEG
MSSA1957	NEG	NEG	NEG	POS	POS	POS	POS	NEG	AMB	NEG	NEG	NEG	POS	POS	POS	NEG
MSSA1748	NEG	NEG	NEG	NEG	NEG	NEG	NEG	POS	POS	POS	POS	POS	POS	POS	POS	NEG
MSSA1754	NEG	NEG	NEG	NEG	NEG	NEG	NEG	POS	POS	POS	POS	POS	POS	POS	POS	NEG
MSSA1770	NEG	NEG	NEG	NEG	NEG	NEG	NEG	POS	POS	POS	POS	POS	POS	POS	POS	NEG
MSSA1788	NEG	NEG	NEG	NEG	NEG	NEG	NEG	POS	POS	POS	POS	POS	POS	POS	POS	NEG
MSSA1804	NEG	NEG	NEG	NEG	NEG	NEG	NEG	POS	POS	POS	POS	POS	POS	POS	POS	NEG
MSSA1818	NEG	NEG	NEG	NEG	NEG	NEG	NEG	POS	POS	POS	POS	POS	POS	POS	POS	NEG
MSSA1914	NEG	NEG	NEG	NEG	NEG	NEG	NEG	POS	POS	POS	POS	POS	POS	POS	POS	NEG
MSSA1974	NEG	NEG	NEG	NEG	NEG	NEG	NEG	POS	POS	POS	POS	POS	POS	POS	POS	NEG
MSSA2023	NEG	NEG	NEG	NEG	NEG	NEG	NEG	POS	POS	POS	POS	POS	POS	POS	POS	NEG
MSSA2030	NEG	NEG	NEG	NEG	NEG	NEG	NEG	POS	POS	POS	POS	POS	POS	POS	POS	NEG
MSSA2096	NEG	NEG	NEG	NEG	NEG	NEG	NEG	POS	POS	POS	POS	POS	POS	POS	POS	NEG
MSSA2142	NEG	NEG	NEG	NEG	NEG	NEG	NEG	POS	POS	POS	POS	POS	POS	POS	POS	NEG
MSSA2203	NEG	NEG	NEG	NEG	NEG	NEG	NEG	POS	POS	POS	POS	POS	POS	POS	POS	NEG
MSSA1901	NEG	NEG	NEG	NEG	NEG	NEG	NEG	POS	POS	POS	POS	POS	POS	POS	POS	NEG
MSSA2013	NEG	NEG	NEG	NEG	NEG	NEG	NEG	POS	POS	POS	POS	POS	POS	POS	POS	NEG
MSSA2031	NEG	AMB	NEG	NEG	NEG	NEG	NEG	POS	POS	POS	POS	POS	POS	POS	POS	NEG
MSSA2052	NEG	NEG	NEG	NEG	NEG	NEG	NEG	POS	POS	POS	POS	POS	POS	POS	POS	NEG
MSSA2057	NEG	NEG	NEG	NEG	NEG	NEG	NEG	POS	POS	POS	POS	POS	POS	POS	POS	NEG
MSSA2081	NEG	NEG	NEG	NEG	NEG	NEG	NEG	POS	POS	POS	POS	POS	POS	POS	POS	NEG
MSSA1955	NEG	NEG	NEG	POS	POS	POS	POS	NEG	AMB	NEG	NEG	NEG	POS	POS	POS	NEG
MSSA1941	NEG	NEG	NEG	NEG	NEG	NEG	NEG	POS	POS	POS	POS	POS	POS	POS	POS	NEG
MRSA3838	NEG	NEG	NEG	POS	POS	POS	POS	NEG	AMB	NEG	NEG	NEG	POS	POS	POS	NEG
MRSA3839	NEG	NEG	NEG	POS	POS	POS	POS	NEG	AMB	NEG	NEG	NEG	POS	POS	POS	NEG
MSSA1801	NEG	NEG	NEG	POS	POS	POS	POS	NEG	NEG	NEG	NEG	NEG	AMB	POS	POS	NEG
MSSA2131	NEG	NEG	NEG	POS	POS	POS	POS	NEG	NEG	NEG	NEG	NEG	POS	POS	POS	NEG
MSSA1792	NEG	NEG	NEG	POS	POS	POS	POS	NEG	NEG	NEG	NEG	NEG	POS	POS	POS	NEG
MSSA1796	NEG	NEG	NEG	POS	POS	POS	POS	NEG	NEG	NEG	NEG	NEG	POS	POS	POS	NEG
MSSA1817	NEG	NEG	NEG	NEG	NEG	AMB	NEG	POS	POS	POS	POS	POS	POS	POS	POS	NEG
MSSA1911	NEG	NEG	NEG	NEG	NEG	NEG	NEG	NEG	NEG	NEG	NEG	NEG	AMB	NEG	NEG	NEG
MSSA1927	NEG	NEG	NEG	NEG	NEG	NEG	NEG	POS	POS	POS	POS	POS	POS	POS	POS	NEG
MSSA1928	NEG	NEG	NEG	NEG	NEG	NEG	NEG	POS	POS	POS	POS	POS	POS	POS	POS	NEG
MSSA1953	NEG	NEG	NEG	NEG	NEG	NEG	NEG	POS	POS	POS	POS	POS	POS	POS	POS	NEG
MSSA2062	NEG	NEG	NEG	POS	POS	POS	POS	NEG	NEG	NEG	NEG	NEG	POS	POS	POS	NEG
MSSA2078	NEG	NEG	NEG	POS	POS	POS	POS	NEG	NEG	NEG	NEG	NEG	POS	POS	POS	NEG
MSSA2136	NEG	NEG	NEG	POS	POS	POS	POS	NEG	NEG	NEG	NEG	NEG	POS	POS	POS	NEG

	ADHAESION FACTORS / GENES ENCODING MICROBIAL SURFACE COMPONENTS RECOGNIZING ADHESIVE MATRIX MOLECULES (MSCRAMM GENES)													
Strain	bbp (total)	bbp (COL+MW2)	bbp (MRSA252)	bbp (Mu50)	bbp (RF122)	bbp (ST45)	clfA (total)	clfA (COL+ RF122)	clfA (MRSA252)	clfA (Mu50 + MW2)	clfB (total)	clfB (COL+ Mu50)	clfB (MW2)	clfB (RF122)
MSSA1805	POS	POS	NEG	NEG	NEG	NEG	POS	NEG	NEG	POS	POS	NEG	POS	AMB
MSSA1823	POS	POS	NEG	NEG	NEG	NEG	POS	NEG	NEG	POS	POS	NEG	POS	AMB
MSSA1837	POS	POS	NEG	NEG	NEG	NEG	POS	NEG	NEG	POS	POS	NEG	POS	AMB
MSSA2044	POS	POS	NEG	NEG	NEG	NEG	POS	NEG	NEG	POS	POS	NEG	POS	AMB
MSSA2046	POS	POS	NEG	NEG	NEG	NEG	POS	NEG	NEG	POS	POS	NEG	POS	AMB
MSSA2059	POS	POS	NEG	NEG	NEG	NEG	POS	NEG	NEG	POS	POS	NEG	POS	AMB
MSSA2076	POS	POS	NEG	NEG	NEG	NEG	POS	NEG	NEG	POS	POS	NEG	POS	AMB
MSSA2200	POS	POS	NEG	NEG	NEG	NEG	POS	NEG	NEG	POS	POS	NEG	POS	AMB
MRSA3797	POS	NEG	NEG	POS	NEG	NEG	POS	NEG	NEG	POS	POS	POS	NEG	NEG
MRSA3798	POS	NEG	NEG	POS	NEG	NEG	POS	NEG	NEG	POS	POS	POS	NEG	NEG
MRSA3799	POS	NEG	NEG	POS	NEG	NEG	POS	NEG	NEG	POS	POS	POS	NEG	NEG
MRSA3800	POS	NEG	NEG	POS	NEG	NEG	POS	NEG	NEG	POS	POS	POS	NEG	NEG
MRSA3801	POS	NEG	NEG	POS	NEG	NEG	POS	NEG	NEG	POS	POS	POS	NEG	NEG
MRSA3804	POS	NEG	NEG	POS	NEG	NEG	POS	NEG	NEG	POS	POS	POS	NEG	NEG
MRSA3806	POS	NEG	NEG	POS	NEG	NEG	POS	NEG	NEG	POS	POS	POS	NEG	NEG
MRSA3961	POS	NEG	NEG	POS	NEG	AMB	POS	NEG	NEG	POS	POS	POS	NEG	NEG
MRSA3962	POS	NEG	NEG	POS	NEG	NEG	POS	NEG	NEG	POS	POS	POS	NEG	NEG
MRSA3963	POS	NEG	NEG	POS	NEG	NEG	POS	NEG	NEG	POS	POS	POS	NEG	NEG
MRSA3966	POS	NEG	NEG	POS	NEG	NEG	POS	NEG	NEG	POS	POS	POS	NEG	NEG
MRSA3978	POS	NEG	NEG	POS	NEG	NEG	POS	NEG	NEG	POS	POS	POS	NEG	NEG
MRSA3979	POS	NEG	NEG	POS	NEG	NEG	POS	NEG	NEG	POS	POS	POS	NEG	NEG
MRSA3980	POS	NEG	NEG	POS	NEG	NEG	POS	NEG	NEG	POS	POS	POS	NEG	NEG
MRSA4179	POS	NEG	NEG	POS	NEG	NEG	POS	NEG	NEG	POS	POS	POS	NEG	NEG
MRSA4216	POS	NEG	NEG	POS	NEG	NEG	POS	NEG	NEG	POS	POS	POS	NEG	NEG
MRSA4217	POS	NEG	NEG	POS	NEG	NEG	POS	NEG	NEG	POS	POS	POS	NEG	NEG
MRSA4220	POS	NEG	NEG	POS	NEG	NEG	POS	NEG	NEG	POS	POS	POS	NEG	NEG
MRSA4221	POS	NEG	NEG	POS	NEG	NEG	POS	NEG	NEG	POS	POS	POS	NEG	NEG
MRSA4222	NEG	NEG	NEG	NEG	NEG	NEG	POS	NEG	NEG	POS	POS	POS	NEG	NEG
MSSA1751	POS	NEG	NEG	POS	NEG	NEG	POS	NEG	NEG	POS	POS	POS	NEG	NEG
MSSA1760	POS	NEG	NEG	POS	NEG	NEG	POS	NEG	NEG	POS	POS	POS	NEG	NEG
MSSA1802	POS	NEG	NEG	POS	NEG	NEG	POS	NEG	NEG	POS	POS	POS	NEG	NEG
MSSA1809	POS	NEG	NEG	POS	NEG	NEG	POS	NEG	NEG	POS	POS	POS	NEG	NEG
MSSA1815	POS	NEG	NEG	POS	NEG	NEG	POS	NEG	NEG	POS	POS	POS	NEG	NEG
MSSA1829	POS	NEG	NEG	POS	NEG	NEG	POS	NEG	NEG	POS	POS	POS	NEG	NEG
MSSA1908	POS	NEG	NEG	POS	NEG	NEG	POS	NEG	NEG	POS	POS	POS	NEG	NEG
MSSA1960	POS	NEG	NEG	POS	NEG	NEG	POS	NEG	NEG	POS	POS	POS	NEG	NEG
MSSA1965	POS	NEG	NEG	POS	NEG	NEG	POS	NEG	NEG	POS	POS	POS	NEG	NEG
MSSA1977	POS	NEG	NEG	POS	NEG	NEG	POS	NEG	NEG	POS	POS	POS	NEG	NEG
MSSA1978	NEG	NEG	NEG	NEG	NEG	NEG	POS	NEG	NEG	POS	POS	POS	NEG	NEG
MSSA1999	POS	NEG	NEG	POS	NEG	NEG	POS	NEG	NEG	POS	POS	POS	NEG	NEG
MSSA2003	POS	NEG	NEG	POS	NEG	NEG	POS	NEG	NEG	POS	POS	POS	NEG	NEG
MSSA2006	POS	NEG	NEG	POS	NEG	NEG	POS	NEG	NEG	POS	POS	POS	NEG	NEG
MSSA2008	POS	NEG	NEG	POS	NEG	NEG	POS	NEG	NEG	POS	POS	POS	NEG	NEG
MSSA2011	POS	NEG	NEG	POS	NEG	NEG	POS	NEG	NEG	POS	POS	POS	NEG	NEG
MSSA2017	POS	NEG	NEG	POS	NEG	NEG	POS	NEG	NEG	POS	POS	POS	NEG	NEG
MSSA2022	POS	NEG	NEG	POS	NEG	NEG	POS	NEG	NEG	POS	POS	POS	NEG	NEG
MSSA2055	POS	NEG	NEG	POS	NEG	NEG	POS	NEG	NEG	POS	POS	POS	NEG	NEG
MSSA2060	POS	NEG	NEG	POS	NEG	NEG	POS	NEG	NEG	POS	POS	POS	NEG	NEG
MSSA2080	POS	NEG	NEG	POS	NEG	NEG	POS	NEG	NEG	POS	POS	POS	NEG	NEG
MSSA2082	POS	AMB	NEG	POS	NEG	NEG	POS	NEG	NEG	POS	POS	POS	NEG	NEG
MSSA2084	POS	NEG	NEG	POS	NEG	NEG	POS	NEG	NEG	POS	POS	POS	NEG	NEG
MSSA2087	POS	NEG	NEG	POS	NEG	NEG	POS	NEG	NEG	POS	POS	POS	NEG	NEG
MSSA2094	POS	NEG	NEG	POS	NEG	NEG	POS	NEG	NEG	POS	POS	POS	NEG	NEG
MSSA2097	POS	NEG	NEG	POS	NEG	NEG	POS	NEG	NEG	POS	POS	POS	NEG	NEG
MSSA2110	POS	NEG	NEG	POS	NEG	NEG	POS	NEG	NEG	POS	POS	POS	NEG	NEG
MSSA2113	POS	NEG	NEG	POS	NEG	NEG	POS	NEG	NEG	POS	POS	POS	NEG	NEG
MSSA2132	POS	NEG	NEG	POS	NEG	NEG	POS	NEG	NEG	POS	POS	POS	NEG	NEG
MSSA2134	POS	NEG	NEG	POS	NEG	NEG	POS	NEG	NEG	POS	POS	POS	NEG	NEG
MSSA1746	POS	POS	AMB	NEG	NEG	NEG	POS	POS	AMB	AMB	POS	NEG	NEG	NEG
MSSA1757	POS	POS	AMB	NEG	NEG	NEG	POS	POS	AMB	AMB	POS	NEG	NEG	POS
MSSA1772	POS	POS	AMB	NEG	NEG	NEG	POS	POS	NEG	AMB	POS	NEG	NEG	NEG
MSSA1795	POS	POS	AMB	NEG	NEG	NEG	POS	POS	AMB	AMB	POS	NEG	NEG	POS
MSSA1813	POS	POS	AMB	NEG	NEG	NEG	POS	POS	AMB	AMB	POS	NEG	NEG	NEG
MSSA1826	POS	POS	AMB	NEG	NEG	NEG	POS	POS	AMB	AMB	POS	NEG	NEG	NEG
MSSA1830	POS	POS	AMB	NEG	NEG	NEG	POS	POS	AMB	AMB	POS	NEG	NEG	NEG
MSSA1838	POS	POS	AMB	NEG	NEG	NEG	POS	POS	AMB	AMB	POS	NEG	NEG	NEG
MSSA1944	POS	POS	AMB	NEG	NEG	NEG	POS	POS	AMB	AMB	POS	NEG	NEG	NEG
MSSA1947	POS	POS	AMB	NEG	NEG	NEG	POS	POS	AMB	AMB	POS	NEG	NEG	NEG
MSSA1964	POS	POS	AMB	NEG	NEG	NEG	POS	POS	AMB	AMB	POS	NEG	NEG	POS
MSSA1969	POS	POS	AMB	NEG	NEG	NEG	POS	POS	AMB	AMB	POS	NEG	NEG	NEG
MSSA2001	POS	POS	AMB	NEG	NEG	NEG	POS	POS	AMB	AMB	POS	NEG	NEG	POS
MSSA2004	POS	POS	AMB	NEG	NEG	NEG	POS	POS	AMB	AMB	POS	NEG	NEG	POS
MSSA2024	POS	POS	AMB	NEG	NEG	NEG	POS	POS	NEG	AMB	POS	NEG	NEG	NEG
MSSA2025	POS	POS	AMB	NEG	NEG	NEG	POS	POS	AMB	AMB	POS	NEG	NEG	NEG
MSSA2026	POS	POS	AMB	NEG	NEG	NEG	POS	POS	AMB	AMB	POS	NEG	NEG	NEG
MSSA2032	POS	POS	AMB	NEG	NEG	NEG	POS	POS	AMB	AMB	POS	NEG	NEG	NEG
MSSA2043	POS	POS	AMB	NEG	NEG	NEG	POS	POS	AMB	AMB	POS	NEG	NEG	NEG
MSSA2061	POS	POS	AMB	NEG	NEG	NEG	POS	POS	AMB	AMB	POS	NEG	NEG	NEG
MSSA2085	POS	POS	AMB	NEG	NEG	NEG	POS	POS	AMB	AMB	POS	NEG	NEG	NEG
MSSA2098	POS	POS	AMB	NEG	NEG	NEG	POS	POS	AMB	AMB	POS	NEG	NEG	POS
MSSA2100	POS	POS	AMB	NEG	NEG	NEG	POS	POS	AMB	AMB	POS	NEG	NEG	POS
MSSA2111	POS	POS	AMB	NEG	NEG	NEG	POS	POS	AMB	AMB	POS	NEG	NEG	POS
MSSA2178	POS	POS	AMB	NEG	NEG	NEG	POS	POS	AMB	AMB	POS	NEG	NEG	POS
MSSA2202	POS	POS	AMB	NEG	NEG	NEG	POS	POS	AMB	AMB	POS	NEG	NEG	NEG
MSSA2205	POS	POS	AMB	NEG	NEG	NEG	POS	POS	AMB	AMB	POS	NEG	NEG	NEG
MRSA3796	NEG	NEG	NEG	NEG	NEG	NEG	POS	AMB	AMB	POS	POS	POS	NEG	NEG
MRSA3805	POS	POS	NEG	NEG	NEG	NEG	POS	POS	AMB	AMB	POS	NEG	NEG	POS
MRSA3965	POS	POS	NEG	NEG	NEG	NEG	POS	POS	AMB	AMB	POS	POS	NEG	NEG
MSSA1761	POS	POS	NEG	AMB	NEG	NEG	POS	AMB	AMB	POS	POS	POS	NEG	NEG
MSSA1764	POS	POS	NEG	NEG	NEG	NEG	POS	POS	NEG	AMB	POS	POS	NEG	NEG
MSSA1765	NEG	NEG	NEG	NEG	NEG	NEG	POS	POS	AMB	AMB	POS	POS	NEG	NEG
MSSA1784	POS	POS	NEG	AMB	NEG	NEG	POS	AMB	AMB	POS	POS	POS	NEG	NEG
MSSA1806	POS	POS	NEG	NEG	NEG	NEG	POS	POS	AMB	AMB	POS	POS	NEG	NEG
MSSA1807	POS	POS	NEG	NEG	NEG	NEG	POS	POS	AMB	AMB	POS	POS	NEG	NEG
MSSA1888	POS	POS	NEG	NEG	NEG	NEG	POS	POS	AMB	AMB	POS	POS	NEG	NEG
MSSA1894	POS	POS	NEG	NEG	NEG	NEG	POS	POS	NEG	AMB	POS	POS	NEG	NEG
MSSA1922	POS	POS	NEG	NEG	NEG	NEG	POS	POS	AMB	AMB	POS	POS	NEG	NEG
MSSA1950	POS	POS	NEG	NEG	NEG	NEG	POS	POS	AMB	AMB	POS	POS	NEG	NEG
MSSA1954	POS	POS	NEG	NEG	NEG	NEG	POS	POS	NEG	AMB	POS	POS	NEG	NEG
MSSA1966	POS	POS	NEG	NEG	NEG	NEG	POS	POS	AMB	AMB	POS	POS	NEG	NEG
MSSA1972	POS	POS	NEG	NEG	NEG	NEG	POS	POS	AMB	AMB	POS	POS	NEG	NEG
MSSA2010	POS	POS	NEG	NEG	NEG	NEG	POS	POS	AMB	AMB	POS	POS	NEG	NEG
MSSA2020	POS	POS	NEG	NEG	NEG	NEG	POS	POS	AMB	AMB	POS	POS	NEG	NEG
MSSA2028	POS	POS	NEG	AMB	NEG	AMB	POS	POS	AMB	AMB	POS	POS	NEG	NEG
MSSA2088	POS	POS	NEG	NEG	NEG	NEG	POS	POS	AMB	AMB	POS	POS	NEG	NEG
MSSA2187	POS	POS	NEG	NEG	NEG	NEG	POS	POS	AMB	AMB	POS	POS	NEG	NEG
MSSA2204	POS	POS	NEG	NEG	NEG	NEG	POS	AMB	AMB	POS	POS	POS	NEG	NEG
MSSA2210	POS	POS	NEG	NEG	NEG	NEG	POS	POS	AMB	AMB	POS	POS	NEG	NEG
MSSA2223	POS	POS	NEG	NEG	NEG	NEG	POS	POS	AMB	AMB	POS	POS	NEG	NEG
MSSA1763	NEG	NEG	NEG	NEG	NEG	NEG	POS	NEG	NEG	POS	POS	NEG	NEG	NEG
MSSA1777	POS	NEG	NEG	POS	NEG	NEG	POS	NEG	NEG	POS	POS	NEG	NEG	POS
MSSA2009	NEG	NEG	NEG	NEG	NEG	NEG	POS	NEG	NEG	POS	POS	NEG	NEG	POS
MSSA2047	POS	NEG	NEG	POS	NEG	NEG	POS	NEG	NEG	POS	POS	NEG	NEG	POS
MSSA2079	POS	NEG	NEG	POS	NEG	NEG	POS	NEG	NEG	POS	POS	NEG	NEG	NEG
MSSA2101	POS	NEG	NEG	POS	NEG	NEG	POS	NEG	NEG	POS	POS	NEG	NEG	POS
MSSA1821	NEG	NEG	NEG	NEG	NEG	NEG	POS	POS	AMB	AMB	POS	NEG	NEG	NEG
MSSA1898	NEG	NEG	NEG	NEG	NEG	NEG	POS	POS	AMB	AMB	POS	NEG	NEG	NEG
MSSA1973	NEG	NEG	NEG	NEG	NEG	NEG	POS	POS	AMB	AMB	POS	NEG	NEG	POS
MSSA2071	NEG	NEG	NEG	NEG	NEG	NEG	POS	POS	AMB	AMB	POS	NEG	NEG	POS
MSSA2083	NEG	NEG	NEG	NEG	NEG	NEG	POS	POS	AMB	AMB	POS	NEG	NEG	POS
MSSA2106	NEG	NEG	NEG	NEG	NEG	NEG	POS	POS	AMB	AMB	POS	NEG	NEG	POS
MSSA2112	POS	NEG	NEG	POS	NEG	NEG	POS	POS	AMB	AMB	POS	NEG	NEG	POS
MSSA2115	POS	NEG	NEG	POS	NEG	NEG	POS	POS	AMB	AMB	POS	NEG	NEG	POS
MSSA2195	NEG	NEG	NEG	NEG	NEG	NEG	POS	POS	AMB	AMB	POS	NEG	NEG	POS
MSSA2207	NEG	NEG	NEG	NEG	NEG	NEG	POS	POS	AMB	AMB	POS	NEG	NEG	POS
MSSA1758	POS	NEG	NEG	POS	NEG	NEG	POS	NEG	NEG	POS	POS	NEG	POS	AMB
MSSA1762	POS	NEG	NEG	POS	NEG	NEG	POS	NEG	NEG	POS	POS	NEG	POS	AMB
MSSA1769	POS	NEG	NEG	POS	NEG	NEG	POS	NEG	NEG	POS	POS	NEG	POS	AMB
MSSA1771	POS	NEG	NEG	POS	NEG	NEG	POS	NEG	NEG	POS	POS	NEG	POS	AMB
MSSA1789	POS	NEG	NEG	POS	NEG	NEG	POS	NEG	NEG	POS	POS	NEG	POS	AMB
MSSA1800	NEG	NEG	NEG	NEG	NEG	NEG	POS	NEG	NEG	POS	POS	NEG	POS	AMB
MSSA1833	POS	NEG	NEG	POS	NEG	NEG	POS	NEG	NEG	POS	POS	NEG	POS	AMB
MSSA1890	POS	NEG	NEG	POS	NEG	NEG	POS	NEG	NEG	POS	POS	NEG	POS	AMB
MSSA1903	POS	NEG	NEG	POS	NEG	NEG	POS	NEG	NEG	POS	POS	NEG	POS	AMB
MSSA1940	POS	NEG	NEG	POS	NEG	NEG	POS	NEG	NEG	POS	POS	NEG	POS	AMB
MSSA1962	POS	NEG	NEG	POS	NEG	NEG	POS	NEG	NEG	POS	POS	NEG	POS	AMB
MSSA1963	POS	NEG	NEG	POS	NEG	NEG	POS	NEG	NEG	POS	POS	NEG	POS	AMB
MSSA2016	POS	NEG	NEG	POS	NEG	NEG	POS	NEG	NEG	POS	POS	NEG	POS	AMB
MSSA2018	POS	NEG	NEG	POS	NEG	NEG	POS	NEG	NEG	POS	POS	NEG	POS	AMB
MSSA2019	POS	NEG	NEG	POS	NEG	NEG	POS	NEG	NEG	POS	POS	NEG	POS	AMB
MSSA2029	POS	NEG	NEG	POS	NEG	NEG	POS	NEG	NEG	POS	POS	NEG	AMB	POS
MSSA2045	POS	NEG	NEG	POS	NEG	NEG	POS	NEG	NEG	POS	POS	NEG	POS	AMB
MSSA2048	POS	NEG	NEG	POS	NEG	NEG	POS	NEG	NEG	POS	POS	NEG	POS	AMB
MSSA2049	POS	NEG	NEG	POS	NEG	NEG	POS	NEG	NEG	POS	POS	NEG	POS	AMB
MSSA2050	POS	NEG	NEG	POS	NEG	NEG	POS	NEG	NEG	POS	POS	NEG	POS	AMB
MSSA2099	POS	NEG	NEG	POS	NEG	NEG	POS	NEG	NEG	POS	POS	NEG	POS	AMB
MSSA2109	POS	NEG	NEG	POS	NEG	NEG	POS	NEG	NEG	POS	POS	NEG	POS	AMB
MSSA2130	POS	NEG	NEG	POS	NEG	NEG	POS	NEG	NEG	POS	POS	NEG	POS	AMB
MSSA2133	POS	NEG	NEG	POS	NEG	NEG	POS	NEG	NEG	POS	POS	NEG	POS	AMB
MSSA2139	NEG	NEG	NEG	NEG	NEG	NEG	POS	NEG	NEG	POS	POS	NEG	POS	AMB
MSSA2141	POS	NEG	NEG	POS	NEG	NEG	POS	NEG	NEG	POS	POS	NEG	POS	AMB
MSSA2208	POS	NEG	NEG	POS	NEG	NEG	POS	NEG	NEG	POS	POS	NEG	POS	AMB
MSSA2209	POS	NEG	NEG	POS	NEG	NEG	POS	NEG	NEG	POS	POS	NEG	POS	AMB
MSSA1893	POS	NEG	NEG	NEG	NEG	NEG	POS	NEG	NEG	POS	POS	NEG	NEG	NEG
MSSA2105	POS	NEG	NEG	NEG	NEG	NEG	POS	NEG	NEG	POS	POS	NEG	NEG	NEG
MRSA3836	POS	NEG	NEG	NEG	NEG	NEG	POS	NEG	NEG	POS	POS	NEG	NEG	NEG
MRSA3840	POS	NEG	NEG	NEG	NEG	NEG	POS	NEG	NEG	POS	POS	NEG	NEG	NEG
MRSA4188	POS	NEG	NEG	POS	NEG	NEG	POS	NEG	NEG	POS	POS	NEG	NEG	POS
MSSA1814	POS	NEG	NEG	NEG	NEG	NEG	POS	NEG	NEG	POS	POS	NEG	NEG	NEG
MSSA1816	POS	NEG	NEG	NEG	NEG	NEG	POS	NEG	NEG	POS	POS	NEG	NEG	NEG
MSSA1825	POS	NEG	NEG	NEG	NEG	NEG	POS	NEG	NEG	POS	POS	NEG	NEG	POS
MSSA1834	POS	NEG	NEG	NEG	NEG	NEG	POS	NEG	NEG	POS	POS	NEG	NEG	NEG
MSSA1938	POS	NEG	NEG	NEG	NEG	NEG	POS	NEG	NEG	POS	POS	NEG	NEG	NEG
MSSA1948	POS	NEG	NEG	NEG	NEG	NEG	POS	NEG	NEG	POS	POS	NEG	NEG	NEG
MSSA1951	POS	NEG	NEG	NEG	NEG	NEG	POS	NEG	NEG	POS	POS	NEG	NEG	NEG
MSSA2005	POS	NEG	NEG	NEG	NEG	NEG	POS	NEG	NEG	POS	POS	NEG	NEG	POS
MSSA2051	POS	NEG	NEG	NEG	NEG	NEG	POS	NEG	NEG	POS	POS	NEG	NEG	POS
MSSA2056	POS	NEG	NEG	NEG	NEG	NEG	POS	NEG	NEG	POS	POS	NEG	NEG	NEG
MSSA2066	POS	NEG	NEG	NEG	NEG	NEG	POS	NEG	NEG	POS	POS	NEG	NEG	POS
MSSA2067	POS	NEG	NEG	NEG	NEG	NEG	POS	NEG	NEG	POS	POS	NEG	NEG	NEG
MSSA2086	POS	NEG	NEG	NEG	NEG	NEG	POS	NEG	NEG	POS	POS	NEG	NEG	NEG
MSSA2089	POS	NEG	NEG	NEG	NEG	NEG	POS	NEG	NEG	POS	POS	NEG	NEG	NEG
MSSA2103	NEG	NEG	NEG	NEG	NEG	NEG	POS	NEG	NEG	POS	POS	NEG	NEG	NEG
MSSA2173	POS	NEG	NEG	NEG	NEG	NEG	POS	NEG	NEG	POS	POS	NEG	NEG	NEG
MSSA2183	POS	NEG	NEG	NEG	NEG	NEG	POS	NEG	NEG	POS	POS	NEG	NEG	POS
MSSA2196	POS	NEG	NEG	NEG	NEG	NEG	POS	NEG	NEG	POS	POS	NEG	NEG	POS
MSSA2219	POS	NEG	NEG	NEG	NEG	NEG	POS	NEG	NEG	POS	POS	NEG	NEG	POS
MSSA1753	POS	POS	NEG	NEG	NEG	NEG	POS	NEG	NEG	POS	POS	NEG	NEG	NEG
MSSA1773	POS	POS	NEG	NEG	NEG	NEG	POS	NEG	NEG	POS	POS	NEG	NEG	NEG
MSSA1811	POS	POS	NEG	NEG	NEG	NEG	POS	NEG	NEG	POS	POS	NEG	NEG	NEG
MSSA1897	POS	POS	NEG	NEG	NEG	NEG	POS	NEG	NEG	POS	POS	NEG	NEG	POS
MSSA1931	POS	POS	NEG	NEG	NEG	NEG	POS	NEG	NEG	POS	POS	NEG	NEG	NEG
MSSA2114	POS	POS	NEG	NEG	NEG	NEG	POS	NEG	NEG	POS	POS	NEG	NEG	POS
MSSA1745	POS	AMB	POS	NEG	NEG	NEG	POS	POS	AMB	NEG	POS	NEG	NEG	POS
MSSA1747	POS	NEG	POS	NEG	NEG	NEG	POS	AMB	POS	NEG	POS	NEG	NEG	NEG
MSSA1749	POS	NEG	POS	NEG	NEG	NEG	POS	AMB	POS	NEG	POS	NEG	NEG	NEG
MSSA1768	POS	NEG	POS	NEG	NEG	NEG	POS	AMB	POS	NEG	POS	NEG	NEG	NEG
MSSA1774	POS	NEG	POS	NEG	NEG	NEG	POS	AMB	POS	NEG	POS	NEG	NEG	NEG
MSSA1775	POS	NEG	POS	NEG	NEG	NEG	POS	AMB	POS	NEG	POS	NEG	NEG	POS
MSSA1776	POS	NEG	POS	NEG	NEG	NEG	POS	AMB	POS	NEG	POS	NEG	NEG	POS
MSSA1779	POS	NEG	POS	NEG	NEG	NEG	POS	AMB	POS	NEG	POS	NEG	NEG	NEG
MSSA1780	POS	NEG	POS	NEG	NEG	NEG	POS	AMB	POS	NEG	POS	NEG	NEG	POS

	ADHAESION FACTORS / GENES ENCODING MICROBIAL SURFACE COMPONENTS RECOGNIZING ADHESIVE MATRIX MOLECULES (MSCRAMM GENES)													
Strain	bbp (total)	bbp (COL+MW2)	bbp (MRSA252)	bbp (Mu50)	bbp (RF122)	bbp (ST45)	clfA (total)	clfA (COL+ RF122)	clfA (MRSA252)	clfA (Mu50 + MW2)	clfB (total)	clfB (COL+ Mu50)	clfB (MW2)	clfB (RF122)
MSSA1781	POS	NEG	POS	NEG	NEG	NEG	POS	AMB	POS	NEG	POS	NEG	NEG	POS
MSSA1786	POS	NEG	POS	NEG	NEG	NEG	POS	POS	AMB	NEG	POS	NEG	NEG	POS
MSSA1793	NEG	NEG	NEG	NEG	NEG	NEG	POS	AMB	POS	NEG	POS	NEG	NEG	NEG
MSSA1803	POS	NEG	POS	NEG	NEG	NEG	POS	AMB	POS	NEG	POS	NEG	NEG	NEG
MSSA1836	POS	NEG	POS	NEG	NEG	NEG	POS	AMB	POS	NEG	POS	NEG	NEG	NEG
MSSA1839	POS	NEG	POS	NEG	NEG	NEG	POS	AMB	POS	NEG	POS	NEG	NEG	POS
MSSA1900	NEG	NEG	NEG	NEG	NEG	NEG	POS	AMB	POS	NEG	POS	NEG	NEG	POS
MSSA1910	NEG	NEG	NEG	NEG	NEG	NEG	POS	AMB	POS	NEG	POS	NEG	POS	AMB
MSSA1918	POS	NEG	POS	NEG	NEG	NEG	POS	AMB	POS	NEG	POS	NEG	POS	AMB
MSSA1921	NEG	NEG	NEG	NEG	NEG	NEG	POS	AMB	POS	NEG	POS	NEG	NEG	NEG
MSSA1923	POS	NEG	POS	NEG	NEG	NEG	POS	AMB	POS	NEG	POS	NEG	NEG	POS
MSSA1981	POS	NEG	POS	NEG	NEG	NEG	POS	AMB	POS	NEG	POS	NEG	AMB	POS
MSSA1998	POS	NEG	POS	NEG	NEG	NEG	POS	AMB	POS	NEG	POS	NEG	NEG	POS
MSSA2000	POS	NEG	POS	NEG	NEG	NEG	POS	AMB	POS	NEG	POS	NEG	POS	AMB
MSSA2007	POS	NEG	POS	NEG	NEG	NEG	POS	AMB	POS	NEG	POS	NEG	NEG	POS
MSSA2012	POS	NEG	POS	NEG	NEG	NEG	POS	AMB	POS	NEG	POS	NEG	NEG	POS
MSSA2015	POS	NEG	POS	NEG	NEG	NEG	POS	AMB	POS	NEG	POS	NEG	NEG	NEG
MSSA2021	POS	NEG	POS	NEG	NEG	NEG	POS	NEG	POS	NEG	POS	NEG	NEG	NEG
MSSA2027	NEG	NEG	NEG	NEG	NEG	NEG	POS	AMB	POS	NEG	POS	NEG	NEG	NEG
MSSA2063	POS	NEG	POS	NEG	NEG	NEG	POS	AMB	POS	NEG	POS	NEG	NEG	NEG
MSSA2064	POS	NEG	POS	NEG	NEG	NEG	POS	AMB	POS	NEG	POS	NEG	NEG	POS
MSSA2065	POS	NEG	POS	NEG	NEG	NEG	POS	AMB	POS	NEG	POS	NEG	NEG	NEG
MSSA2075	POS	NEG	POS	NEG	NEG	NEG	POS	AMB	POS	NEG	POS	NEG	NEG	NEG
MSSA2093	POS	NEG	POS	NEG	NEG	NEG	POS	AMB	POS	NEG	POS	NEG	POS	AMB
MSSA2102	NEG	NEG	NEG	NEG	NEG	NEG	POS	AMB	POS	NEG	POS	NEG	NEG	NEG
MSSA2138	POS	NEG	POS	NEG	NEG	NEG	POS	AMB	POS	NEG	POS	NEG	NEG	POS
MSSA2168	POS	NEG	POS	NEG	NEG	NEG	POS	AMB	POS	NEG	POS	NEG	NEG	NEG
MSSA2181	POS	NEG	POS	NEG	NEG	NEG	POS	AMB	POS	NEG	POS	NEG	NEG	NEG
MSSA2189	POS	NEG	POS	NEG	NEG	NEG	POS	AMB	POS	NEG	POS	NEG	AMB	POS
MSSA2214	POS	NEG	POS	NEG	NEG	NEG	POS	AMB	POS	NEG	POS	NEG	NEG	NEG
MRSA3803	POS	NEG	NEG	AMB	NEG	POS	POS	AMB	NEG	POS	POS	NEG	POS	AMB
MSSA1750	POS	AMB	NEG	NEG	NEG	POS	POS	POS	AMB	AMB	POS	NEG	POS	AMB
MSSA1756	POS	AMB	NEG	AMB	NEG	POS	POS	AMB	NEG	POS	POS	NEG	POS	AMB
MSSA1759	POS	NEG	NEG	NEG	NEG	POS	POS	AMB	NEG	POS	POS	NEG	POS	AMB
MSSA1767	NEG	NEG	NEG	NEG	NEG	NEG	POS	AMB	NEG	POS	POS	NEG	POS	AMB
MSSA1787	POS	NEG	NEG	NEG	NEG	POS	POS	AMB	NEG	POS	POS	NEG	POS	AMB
MSSA1790	POS	NEG	NEG	NEG	NEG	POS	POS	AMB	NEG	POS	POS	NEG	POS	AMB
MSSA1794	POS	NEG	NEG	NEG	NEG	POS	POS	NEG	NEG	POS	POS	NEG	POS	AMB
MSSA1808	POS	NEG	NEG	NEG	NEG	POS	POS	NEG	NEG	POS	POS	NEG	POS	AMB
MSSA1812	POS	NEG	NEG	NEG	NEG	POS	POS	NEG	NEG	POS	POS	NEG	POS	AMB
MSSA1819	POS	NEG	NEG	NEG	NEG	POS	POS	AMB	NEG	POS	POS	NEG	POS	AMB
MSSA1824	POS	NEG	NEG	NEG	NEG	POS	POS	AMB	NEG	POS	POS	NEG	POS	AMB
MSSA1827	NEG	NEG	NEG	NEG	NEG	NEG	POS	NEG	NEG	POS	POS	NEG	POS	AMB
MSSA1889	NEG	NEG	NEG	NEG	NEG	NEG	POS	NEG	NEG	POS	POS	NEG	POS	AMB
MSSA1895	NEG	NEG	NEG	NEG	NEG	NEG	POS	NEG	NEG	POS	POS	NEG	POS	AMB
MSSA1904	POS	NEG	NEG	NEG	NEG	POS	POS	AMB	NEG	POS	POS	NEG	POS	AMB
MSSA1905	NEG	NEG	NEG	NEG	NEG	NEG	POS	NEG	NEG	POS	POS	NEG	POS	AMB
MSSA1907	POS	NEG	NEG	NEG	NEG	POS	POS	NEG	NEG	POS	POS	NEG	POS	AMB
MSSA1919	POS	NEG	NEG	NEG	NEG	POS	POS	NEG	NEG	POS	POS	NEG	POS	AMB
MSSA1920	POS	NEG	NEG	NEG	NEG	POS	POS	AMB	NEG	POS	POS	NEG	POS	AMB
MSSA1929	POS	NEG	NEG	NEG	NEG	POS	POS	AMB	NEG	POS	POS	NEG	POS	AMB
MSSA1930	POS	NEG	NEG	NEG	NEG	POS	POS	AMB	NEG	POS	POS	NEG	POS	AMB
MSSA1934	POS	NEG	NEG	NEG	NEG	POS	POS	AMB	NEG	POS	POS	NEG	POS	AMB
MSSA1946	POS	NEG	NEG	NEG	NEG	POS	POS	NEG	NEG	POS	POS	NEG	POS	AMB
MSSA1971	POS	NEG	NEG	NEG	NEG	POS	POS	AMB	NEG	POS	POS	NEG	POS	AMB
MSSA1980	POS	NEG	NEG	NEG	NEG	POS	POS	NEG	NEG	POS	POS	NEG	POS	AMB
MSSA1982	NEG	NEG	NEG	NEG	NEG	NEG	POS	NEG	NEG	POS	POS	NEG	POS	AMB
MSSA1996	POS	NEG	NEG	NEG	NEG	POS	POS	AMB	NEG	POS	POS	NEG	POS	AMB
MSSA2002	POS	NEG	NEG	NEG	NEG	POS	POS	AMB	NEG	POS	POS	NEG	POS	AMB
MSSA2014	POS	NEG	NEG	NEG	NEG	POS	POS	AMB	NEG	POS	POS	NEG	POS	AMB
MSSA2053	POS	NEG	NEG	NEG	NEG	POS	POS	NEG	NEG	POS	POS	NEG	POS	AMB
MSSA2054	POS	NEG	NEG	NEG	NEG	POS	POS	NEG	NEG	POS	POS	NEG	POS	AMB
MSSA2058	POS	NEG	NEG	NEG	NEG	POS	POS	NEG	NEG	POS	POS	NEG	POS	AMB
MSSA2068	POS	NEG	NEG	NEG	NEG	POS	POS	AMB	NEG	POS	POS	NEG	POS	AMB
MSSA2070	POS	NEG	NEG	NEG	NEG	POS	POS	AMB	NEG	POS	POS	NEG	POS	AMB
MSSA2072	POS	NEG	NEG	NEG	NEG	POS	POS	AMB	NEG	POS	POS	NEG	POS	AMB
MSSA2073	POS	NEG	NEG	NEG	NEG	POS	POS	NEG	NEG	POS	POS	NEG	POS	AMB
MSSA2074	POS	NEG	NEG	AMB	NEG	POS	POS	AMB	NEG	POS	POS	NEG	POS	AMB
MSSA2107	POS	NEG	NEG	NEG	NEG	POS	POS	AMB	NEG	POS	POS	NEG	POS	AMB
MSSA2108	POS	NEG	NEG	NEG	NEG	POS	POS	NEG	NEG	POS	POS	NEG	POS	AMB
MSSA2129	POS	NEG	NEG	NEG	NEG	POS	POS	AMB	NEG	POS	POS	NEG	POS	AMB
MSSA2135	POS	NEG	NEG	NEG	NEG	POS	POS	AMB	NEG	POS	POS	NEG	POS	AMB
MSSA2137	NEG	NEG	NEG	NEG	NEG	NEG	POS	AMB	NEG	POS	POS	NEG	POS	AMB
MSSA2140	POS	NEG	NEG	NEG	NEG	POS	POS	AMB	NEG	POS	POS	NEG	POS	AMB
MSSA2182	POS	NEG	NEG	NEG	NEG	POS	POS	AMB	NEG	POS	POS	NEG	POS	AMB
MSSA2188	NEG	NEG	NEG	NEG	NEG	NEG	POS	AMB	NEG	POS	POS	NEG	POS	AMB
MSSA1899	POS	NEG	NEG	NEG	NEG	NEG	POS	POS	AMB	AMB	POS	NEG	AMB	POS
MSSA1915	POS	NEG	NEG	NEG	NEG	NEG	POS	POS	AMB	AMB	POS	NEG	AMB	POS
MSSA1949	POS	NEG	NEG	NEG	NEG	NEG	POS	POS	AMB	AMB	POS	NEG	AMB	POS
MSSA2042	POS	NEG	NEG	NEG	NEG	NEG	POS	POS	AMB	AMB	POS	NEG	AMB	POS
MSSA2077	POS	NEG	NEG	NEG	NEG	NEG	POS	POS	NEG	AMB	POS	NEG	AMB	POS
MSSA2095	POS	NEG	NEG	NEG	NEG	NEG	POS	AMB	AMB	POS	POS	NEG	POS	AMB
MSSA1997	NEG	NEG	NEG	NEG	NEG	NEG	POS	NEG	NEG	POS	POS	NEG	NEG	POS
MSSA1810	POS	NEG	NEG	POS	NEG	NEG	POS	NEG	NEG	POS	AMB	NEG	NEG	NEG
MSSA1832	POS	NEG	NEG	POS	NEG	NEG	POS	POS	AMB	AMB	POS	NEG	NEG	NEG
MSSA1891	POS	NEG	NEG	POS	NEG	NEG	POS	AMB	AMB	POS	POS	NEG	NEG	POS
MSSA1892	POS	NEG	NEG	POS	NEG	NEG	POS	POS	AMB	AMB	POS	NEG	NEG	POS
MSSA1932	POS	NEG	NEG	POS	NEG	NEG	POS	POS	AMB	AMB	POS	NEG	NEG	POS
MSSA1942	NEG	NEG	NEG	NEG	NEG	NEG	POS	POS	AMB	AMB	POS	NEG	NEG	POS
MSSA1957	POS	NEG	NEG	POS	NEG	NEG	POS	POS	NEG	AMB	POS	NEG	NEG	NEG
MSSA1748	POS	POS	AMB	NEG	NEG	NEG	POS	POS	AMB	AMB	POS	NEG	NEG	POS
MSSA1754	POS	POS	AMB	NEG	NEG	NEG	POS	POS	AMB	AMB	POS	NEG	NEG	POS
MSSA1770	POS	POS	AMB	NEG	NEG	NEG	POS	POS	AMB	AMB	POS	NEG	NEG	NEG
MSSA1788	POS	POS	AMB	NEG	NEG	NEG	POS	POS	NEG	AMB	POS	NEG	NEG	NEG
MSSA1804	POS	POS	AMB	NEG	NEG	NEG	POS	POS	AMB	AMB	POS	NEG	NEG	NEG
MSSA1818	POS	POS	AMB	NEG	NEG	NEG	POS	POS	AMB	AMB	POS	NEG	NEG	NEG
MSSA1914	NEG	NEG	NEG	NEG	NEG	NEG	POS	AMB	AMB	POS	POS	NEG	NEG	POS
MSSA1974	POS	POS	AMB	NEG	NEG	NEG	POS	POS	NEG	AMB	POS	NEG	NEG	NEG
MSSA2023	POS	POS	AMB	NEG	NEG	NEG	POS	POS	AMB	AMB	POS	NEG	NEG	POS
MSSA2030	POS	POS	AMB	NEG	NEG	NEG	POS	POS	AMB	AMB	POS	NEG	NEG	POS
MSSA2096	POS	POS	AMB	NEG	NEG	NEG	POS	POS	NEG	AMB	NEG	NEG	NEG	NEG
MSSA2142	POS	POS	AMB	NEG	NEG	NEG	POS	AMB	AMB	POS	POS	NEG	NEG	POS
MSSA2203	POS	POS	AMB	NEG	NEG	NEG	POS	POS	AMB	AMB	POS	NEG	NEG	NEG
MSSA1901	POS	NEG	POS	NEG	NEG	NEG	POS	NEG	NEG	POS	POS	NEG	NEG	NEG
MSSA2013	AMB	NEG	POS	NEG	NEG	AMB	POS	NEG	NEG	POS	POS	NEG	NEG	POS
MSSA2031	POS	NEG	POS	NEG	NEG	AMB	POS	NEG	NEG	POS	POS	NEG	NEG	POS
MSSA2052	POS	NEG	POS	NEG	NEG	NEG	POS	NEG	NEG	POS	POS	NEG	NEG	NEG
MSSA2057	POS	NEG	POS	NEG	NEG	NEG	POS	NEG	NEG	POS	POS	NEG	NEG	NEG
MSSA2081	POS	NEG	NEG	NEG	NEG	NEG	POS	POS	AMB	AMB	POS	NEG	AMB	POS
MSSA1955	NEG	NEG	NEG	NEG	NEG	NEG	POS	POS	AMB	AMB	POS	NEG	NEG	POS
MSSA1941	POS	NEG	NEG	NEG	NEG	POS	POS	NEG	NEG	POS	POS	NEG	POS	AMB
MRSA3838	POS	NEG	NEG	NEG	NEG	POS	POS	POS	NEG	AMB	POS	NEG	POS	AMB
MRSA3839	NEG	NEG	NEG	NEG	NEG	NEG	POS	POS	NEG	AMB	POS	NEG	POS	AMB
MSSA1801	POS	NEG	NEG	NEG	NEG	NEG	POS	POS	NEG	AMB	POS	NEG	POS	AMB
MSSA2131	POS	NEG	NEG	NEG	NEG	POS	POS	POS	AMB	AMB	POS	NEG	POS	AMB
MSSA1792	POS	NEG	NEG	POS	NEG	NEG	POS	POS	NEG	AMB	POS	NEG	NEG	NEG
MSSA1796	POS	NEG	NEG	NEG	NEG	NEG	POS	NEG	NEG	POS	POS	AMB	POS	AMB
MSSA1817	POS	NEG	NEG	NEG	NEG	POS	POS	NEG	NEG	POS	POS	NEG	NEG	POS
MSSA1911	POS	NEG	NEG	NEG	NEG	NEG	NEG	NEG	NEG	NEG	NEG	POS	NEG	NEG
MSSA1927	POS	POS	NEG	NEG	NEG	NEG	POS	NEG	NEG	POS	POS	NEG	NEG	POS
MSSA1928	POS	POS	NEG	NEG	NEG	NEG	POS	NEG	NEG	POS	POS	NEG	NEG	POS
MSSA1953	POS	POS	NEG	NEG	NEG	NEG	POS	NEG	NEG	POS	POS	NEG	NEG	NEG
MSSA2062	POS	NEG	NEG	POS	NEG	NEG	POS	POS	AMB	AMB	POS	NEG	NEG	NEG
MSSA2078	NEG	NEG	NEG	NEG	NEG	NEG	POS	POS	AMB	AMB	POS	NEG	POS	AMB
MSSA2136	POS	NEG	NEG	POS	NEG	NEG	POS	NEG	NEG	POS	POS	NEG	NEG	POS

	ADHAESION FACTORS / GENES ENCODING MICROBIAL SURFACE COMPONENTS RECOGNIZING ADHESIVE MATRIX MOLECULES (MSCRAMM GENES), continued													
Strain	cna	ebh (total)	ebpS (probe 612)	ebpS (probe 614)	ebpS (01-1111)	ebpS (COL)	eno	fib	fib (MRSA252)	fnbA (total)	fnbA (COL)	fnbA (MRSA252)	fnbA (Mu50+ MW2)	fnbA (RF122)
MSSA1805	POS	POS	POS	POS	NEG	NEG	POS	POS	NEG	POS	NEG	NEG	POS	NEG
MSSA1823	POS	POS	POS	POS	NEG	NEG	POS	POS	NEG	POS	NEG	NEG	POS	NEG
MSSA1837	POS	POS	POS	POS	NEG	NEG	POS	POS	NEG	POS	NEG	NEG	POS	NEG
MSSA2044	POS	POS	POS	POS	NEG	NEG	POS	POS	NEG	POS	NEG	NEG	POS	NEG
MSSA2046	POS	POS	POS	POS	NEG	NEG	POS	POS	NEG	POS	NEG	NEG	POS	NEG
MSSA2059	POS	POS	POS	POS	NEG	NEG	POS	POS	NEG	POS	NEG	NEG	POS	NEG
MSSA2076	POS	POS	POS	POS	NEG	NEG	POS	POS	NEG	POS	NEG	NEG	POS	NEG
MSSA2200	POS	POS	POS	POS	NEG	NEG	POS	POS	NEG	POS	NEG	NEG	POS	NEG
MRSA3797	NEG	POS	POS	POS	NEG	NEG	POS	POS	NEG	POS	NEG	NEG	POS	NEG
MRSA3798	NEG	POS	POS	POS	NEG	NEG	POS	POS	NEG	POS	NEG	NEG	POS	NEG
MRSA3799	NEG	POS	POS	POS	NEG	NEG	POS	POS	NEG	POS	NEG	NEG	POS	NEG
MRSA3800	NEG	POS	POS	POS	NEG	NEG	POS	POS	NEG	POS	NEG	NEG	POS	NEG
MRSA3801	NEG	POS	POS	POS	NEG	NEG	POS	POS	NEG	POS	NEG	NEG	POS	NEG
MRSA3804	NEG	POS	POS	POS	NEG	NEG	POS	POS	NEG	POS	NEG	NEG	POS	NEG
MRSA3806	NEG	POS	POS	POS	NEG	NEG	POS	POS	NEG	POS	NEG	NEG	POS	NEG
MRSA3961	NEG	POS	POS	POS	NEG	NEG	POS	POS	NEG	POS	NEG	NEG	POS	NEG
MRSA3962	NEG	POS	POS	POS	NEG	NEG	POS	POS	NEG	POS	NEG	NEG	POS	NEG
MRSA3963	NEG	POS	POS	POS	NEG	NEG	POS	POS	NEG	POS	NEG	NEG	POS	NEG
MRSA3966	NEG	POS	POS	POS	NEG	NEG	POS	POS	NEG	POS	NEG	NEG	POS	NEG
MRSA3978	NEG	POS	POS	POS	NEG	NEG	POS	POS	NEG	POS	NEG	NEG	POS	NEG
MRSA3979	NEG	POS	AMB	POS	NEG	NEG	POS	POS	NEG	POS	NEG	NEG	POS	NEG
MRSA3980	NEG	POS	POS	POS	NEG	NEG	POS	POS	NEG	POS	NEG	NEG	POS	NEG
MRSA4179	NEG	POS	POS	POS	NEG	NEG	POS	POS	NEG	POS	NEG	NEG	POS	NEG
MRSA4216	NEG	POS	POS	POS	NEG	NEG	POS	POS	NEG	POS	NEG	NEG	POS	NEG
MRSA4217	NEG	POS	POS	POS	NEG	NEG	POS	POS	NEG	POS	NEG	NEG	POS	NEG
MRSA4220	NEG	POS	POS	POS	NEG	NEG	POS	POS	NEG	POS	NEG	NEG	POS	NEG
MRSA4221	NEG	POS	POS	POS	NEG	NEG	POS	POS	NEG	POS	AMB	NEG	POS	NEG
MRSA4222	NEG	POS	POS	POS	NEG	NEG	POS	POS	NEG	POS	NEG	NEG	POS	NEG
MSSA1751	NEG	POS	POS	POS	NEG	NEG	POS	POS	NEG	POS	NEG	NEG	POS	NEG
MSSA1760	NEG	POS	POS	POS	NEG	NEG	POS	POS	NEG	POS	NEG	NEG	POS	NEG
MSSA1802	NEG	POS	POS	POS	NEG	NEG	POS	POS	NEG	POS	NEG	NEG	POS	NEG
MSSA1809	NEG	POS	POS	POS	NEG	NEG	POS	POS	NEG	POS	NEG	NEG	POS	NEG
MSSA1815	NEG	POS	POS	POS	NEG	NEG	POS	POS	NEG	POS	NEG	NEG	POS	NEG
MSSA1829	NEG	POS	POS	POS	NEG	NEG	POS	POS	NEG	POS	NEG	NEG	POS	NEG
MSSA1908	NEG	POS	POS	POS	NEG	NEG	POS	POS	NEG	POS	NEG	NEG	POS	NEG
MSSA1960	NEG	POS	POS	POS	NEG	NEG	POS	POS	NEG	POS	NEG	NEG	POS	NEG
MSSA1965	NEG	POS	POS	POS	NEG	NEG	POS	POS	NEG	POS	NEG	NEG	POS	NEG
MSSA1977	NEG	POS	POS	POS	NEG	NEG	POS	POS	NEG	POS	NEG	NEG	POS	NEG
MSSA1978	NEG	POS	POS	POS	NEG	NEG	POS	POS	NEG	POS	NEG	NEG	POS	NEG
MSSA1999	NEG	POS	POS	POS	NEG	NEG	POS	POS	NEG	POS	NEG	NEG	POS	NEG
MSSA2003	NEG	POS	POS	POS	NEG	NEG	POS	POS	NEG	POS	NEG	NEG	POS	NEG
MSSA2006	NEG	POS	POS	POS	NEG	NEG	POS	POS	NEG	POS	NEG	NEG	POS	NEG
MSSA2008	NEG	POS	POS	POS	NEG	NEG	POS	POS	NEG	POS	NEG	NEG	POS	NEG
MSSA2011	NEG	POS	POS	POS	NEG	NEG	POS	POS	NEG	POS	NEG	NEG	POS	NEG
MSSA2017	NEG	POS	POS	POS	NEG	NEG	POS	POS	NEG	POS	NEG	NEG	POS	NEG
MSSA2022	NEG	POS	POS	POS	NEG	NEG	POS	POS	NEG	POS	NEG	NEG	POS	NEG
MSSA2055	NEG	POS	POS	POS	NEG	NEG	POS	POS	NEG	POS	NEG	NEG	POS	NEG
MSSA2060	NEG	POS	POS	POS	NEG	NEG	POS	POS	NEG	POS	NEG	NEG	POS	NEG
MSSA2080	NEG	POS	POS	POS	NEG	NEG	POS	POS	NEG	POS	NEG	NEG	POS	NEG
MSSA2082	NEG	POS	POS	POS	NEG	NEG	POS	POS	NEG	POS	NEG	NEG	POS	NEG
MSSA2084	NEG	POS	AMB	POS	NEG	NEG	POS	POS	NEG	POS	NEG	NEG	POS	NEG
MSSA2087	NEG	POS	POS	POS	NEG	NEG	POS	POS	NEG	POS	NEG	NEG	POS	NEG
MSSA2094	NEG	POS	POS	POS	NEG	NEG	POS	POS	NEG	POS	NEG	NEG	POS	NEG
MSSA2097	NEG	POS	POS	POS	NEG	NEG	POS	POS	NEG	POS	NEG	NEG	POS	NEG
MSSA2110	NEG	POS	POS	POS	NEG	NEG	POS	POS	NEG	POS	NEG	NEG	POS	NEG
MSSA2113	NEG	POS	POS	POS	NEG	NEG	POS	POS	AMB	POS	NEG	NEG	POS	NEG
MSSA2132	NEG	POS	POS	POS	NEG	NEG	POS	POS	NEG	POS	NEG	NEG	POS	NEG
MSSA2134	NEG	POS	POS	POS	NEG	NEG	POS	POS	NEG	POS	NEG	NEG	POS	NEG
MSSA1746	NEG	POS	POS	POS	NEG	POS	POS	POS	NEG	POS	NEG	NEG	NEG	NEG
MSSA1757	NEG	POS	POS	POS	NEG	POS	POS	POS	NEG	POS	NEG	NEG	NEG	NEG
MSSA1772	NEG	POS	POS	POS	NEG	POS	POS	POS	NEG	NEG	NEG	NEG	NEG	NEG
MSSA1795	NEG	POS	POS	POS	NEG	POS	POS	POS	NEG	POS	NEG	NEG	NEG	NEG
MSSA1813	NEG	POS	POS	POS	NEG	POS	POS	POS	NEG	POS	NEG	NEG	NEG	NEG
MSSA1826	NEG	POS	POS	POS	NEG	POS	POS	POS	NEG	POS	NEG	NEG	NEG	NEG
MSSA1830	NEG	POS	POS	POS	NEG	POS	POS	POS	NEG	POS	NEG	NEG	NEG	NEG
MSSA1838	NEG	POS	POS	POS	NEG	POS	POS	POS	NEG	POS	NEG	NEG	NEG	NEG
MSSA1944	NEG	POS	POS	POS	NEG	POS	POS	POS	NEG	POS	NEG	NEG	NEG	NEG
MSSA1947	NEG	POS	POS	POS	NEG	POS	POS	POS	NEG	POS	NEG	NEG	NEG	NEG
MSSA1964	NEG	POS	POS	POS	NEG	POS	POS	POS	NEG	POS	NEG	NEG	NEG	NEG
MSSA1969	NEG	POS	POS	POS	NEG	POS	POS	POS	NEG	POS	NEG	NEG	NEG	NEG
MSSA2001	NEG	POS	POS	POS	NEG	POS	POS	POS	NEG	POS	NEG	NEG	NEG	NEG
MSSA2004	NEG	POS	POS	POS	NEG	POS	POS	POS	NEG	POS	NEG	NEG	NEG	NEG
MSSA2024	NEG	POS	POS	POS	NEG	POS	POS	POS	NEG	POS	NEG	NEG	NEG	NEG
MSSA2025	NEG	POS	POS	POS	NEG	POS	POS	POS	NEG	POS	NEG	NEG	NEG	NEG
MSSA2026	NEG	POS	POS	POS	NEG	POS	POS	POS	NEG	POS	NEG	NEG	NEG	NEG
MSSA2032	NEG	POS	POS	POS	NEG	POS	POS	POS	NEG	POS	NEG	NEG	NEG	NEG
MSSA2043	NEG	POS	POS	POS	NEG	POS	POS	POS	NEG	POS	NEG	NEG	NEG	NEG
MSSA2061	NEG	POS	POS	POS	NEG	POS	POS	POS	NEG	POS	NEG	NEG	NEG	NEG
MSSA2085	NEG	POS	POS	POS	NEG	POS	POS	POS	NEG	POS	NEG	NEG	NEG	NEG
MSSA2098	NEG	POS	POS	POS	NEG	POS	POS	POS	NEG	POS	NEG	NEG	NEG	NEG
MSSA2100	NEG	POS	POS	POS	NEG	POS	POS	POS	NEG	POS	NEG	NEG	NEG	NEG
MSSA2111	NEG	POS	POS	POS	NEG	POS	POS	POS	NEG	POS	NEG	NEG	NEG	NEG
MSSA2178	NEG	POS	POS	POS	NEG	POS	POS	POS	NEG	POS	NEG	NEG	NEG	NEG
MSSA2202	NEG	POS	POS	POS	NEG	POS	POS	POS	NEG	POS	NEG	NEG	NEG	NEG
MSSA2205	NEG	POS	POS	POS	NEG	POS	POS	POS	NEG	POS	NEG	NEG	NEG	NEG
MRSA3796	NEG	POS	POS	POS	NEG	POS	POS	POS	NEG	POS	POS	NEG	NEG	NEG
MRSA3805	POS	POS	POS	POS	NEG	POS	POS	POS	NEG	POS	POS	NEG	NEG	NEG
MRSA3965	NEG	POS	POS	POS	NEG	POS	POS	POS	NEG	POS	POS	NEG	NEG	NEG
MSSA1761	NEG	POS	POS	POS	NEG	POS	POS	POS	NEG	POS	POS	NEG	NEG	NEG
MSSA1764	NEG	POS	POS	POS	NEG	POS	POS	POS	NEG	POS	POS	NEG	NEG	NEG
MSSA1765	NEG	POS	POS	POS	NEG	POS	POS	POS	NEG	POS	POS	NEG	NEG	NEG
MSSA1784	NEG	POS	POS	POS	NEG	POS	POS	POS	NEG	POS	POS	NEG	NEG	NEG
MSSA1806	NEG	POS	POS	POS	NEG	POS	POS	POS	NEG	POS	POS	NEG	NEG	NEG
MSSA1807	NEG	POS	POS	POS	NEG	POS	POS	POS	NEG	POS	POS	NEG	NEG	NEG
MSSA1888	NEG	POS	POS	POS	NEG	POS	POS	POS	NEG	POS	POS	NEG	NEG	NEG
MSSA1894	NEG	POS	POS	POS	NEG	POS	POS	POS	NEG	POS	POS	NEG	NEG	NEG
MSSA1922	NEG	POS	POS	POS	NEG	POS	POS	POS	NEG	POS	POS	NEG	NEG	NEG
MSSA1950	NEG	POS	POS	POS	NEG	POS	POS	POS	NEG	POS	POS	NEG	NEG	NEG
MSSA1954	NEG	POS	POS	POS	NEG	POS	POS	POS	NEG	POS	POS	NEG	NEG	NEG
MSSA1966	NEG	POS	POS	POS	NEG	POS	POS	POS	NEG	POS	POS	NEG	NEG	NEG
MSSA1972	NEG	POS	POS	POS	NEG	POS	POS	POS	NEG	POS	POS	NEG	NEG	NEG
MSSA2010	NEG	POS	POS	POS	NEG	POS	POS	POS	NEG	POS	POS	NEG	NEG	NEG
MSSA2020	NEG	POS	POS	POS	NEG	POS	POS	POS	NEG	POS	POS	NEG	NEG	NEG
MSSA2028	NEG	POS	POS	POS	NEG	POS	POS	POS	NEG	POS	POS	NEG	NEG	NEG
MSSA2088	NEG	POS	POS	POS	NEG	POS	POS	POS	NEG	POS	POS	NEG	NEG	NEG
MSSA2187	NEG	POS	POS	POS	NEG	POS	POS	POS	NEG	POS	POS	NEG	NEG	NEG
MSSA2204	NEG	POS	POS	POS	NEG	POS	POS	POS	NEG	POS	POS	NEG	NEG	NEG
MSSA2210	NEG	POS	POS	POS	NEG	POS	POS	POS	NEG	POS	POS	NEG	NEG	NEG
MSSA2223	NEG	POS	POS	POS	NEG	POS	POS	POS	NEG	POS	POS	NEG	NEG	NEG
MSSA1763	NEG	POS	POS	POS	NEG	NEG	POS	POS	NEG	POS	NEG	POS	NEG	NEG
MSSA1777	NEG	POS	POS	POS	NEG	AMB	POS	POS	NEG	POS	NEG	POS	NEG	NEG
MSSA2009	NEG	POS	POS	POS	NEG	NEG	POS	POS	NEG	POS	NEG	POS	NEG	NEG
MSSA2047	NEG	POS	POS	POS	NEG	NEG	POS	POS	NEG	POS	NEG	POS	NEG	NEG
MSSA2079	NEG	POS	POS	POS	NEG	NEG	POS	POS	NEG	POS	NEG	POS	NEG	NEG
MSSA2101	NEG	POS	POS	POS	NEG	NEG	POS	POS	NEG	POS	NEG	POS	NEG	NEG
MSSA1821	POS	POS	POS	POS	NEG	NEG	POS	POS	NEG	POS	NEG	NEG	NEG	NEG
MSSA1898	POS	POS	POS	POS	NEG	NEG	POS	POS	NEG	POS	NEG	NEG	NEG	NEG
MSSA1973	POS	POS	POS	POS	NEG	NEG	POS	POS	NEG	POS	NEG	NEG	NEG	NEG
MSSA2071	POS	POS	POS	POS	NEG	NEG	POS	POS	NEG	POS	NEG	NEG	NEG	NEG
MSSA2083	POS	POS	POS	POS	NEG	NEG	POS	POS	NEG	POS	NEG	NEG	NEG	NEG
MSSA2106	POS	POS	POS	POS	NEG	NEG	POS	POS	NEG	POS	NEG	NEG	NEG	NEG
MSSA2112	POS	POS	POS	POS	NEG	NEG	POS	POS	NEG	POS	NEG	NEG	NEG	NEG
MSSA2115	POS	POS	POS	POS	NEG	NEG	POS	POS	NEG	POS	NEG	NEG	NEG	NEG
MSSA2195	POS	POS	POS	POS	NEG	NEG	POS	POS	NEG	POS	NEG	NEG	NEG	NEG
MSSA2207	POS	POS	POS	POS	NEG	NEG	POS	POS	NEG	POS	NEG	NEG	NEG	NEG
MSSA1758	NEG	POS	POS	POS	NEG	POS	POS	POS	NEG	POS	POS	NEG	NEG	NEG
MSSA1762	NEG	POS	POS	POS	NEG	POS	POS	POS	NEG	POS	POS	NEG	NEG	NEG
MSSA1769	NEG	POS	POS	POS	NEG	POS	POS	POS	NEG	POS	POS	NEG	NEG	NEG
MSSA1771	NEG	POS	POS	POS	NEG	POS	POS	POS	NEG	POS	POS	NEG	NEG	NEG
MSSA1789	NEG	POS	POS	POS	NEG	POS	POS	POS	NEG	POS	POS	NEG	NEG	NEG
MSSA1800	NEG	POS	POS	POS	NEG	POS	POS	POS	NEG	POS	POS	NEG	NEG	NEG
MSSA1833	NEG	POS	POS	POS	NEG	POS	POS	POS	NEG	POS	POS	NEG	NEG	NEG
MSSA1890	NEG	POS	POS	POS	NEG	POS	POS	POS	NEG	POS	POS	NEG	NEG	NEG
MSSA1903	NEG	POS	POS	POS	NEG	POS	POS	POS	NEG	POS	POS	NEG	NEG	NEG
MSSA1940	NEG	POS	POS	POS	NEG	POS	POS	POS	NEG	POS	POS	NEG	NEG	NEG
MSSA1962	NEG	POS	POS	POS	NEG	POS	POS	POS	NEG	POS	POS	NEG	NEG	NEG
MSSA1963	NEG	POS	POS	POS	NEG	POS	POS	POS	NEG	POS	POS	NEG	NEG	NEG
MSSA2016	NEG	POS	POS	POS	NEG	POS	POS	POS	NEG	POS	POS	NEG	NEG	NEG
MSSA2018	NEG	POS	POS	POS	NEG	POS	POS	POS	NEG	POS	POS	NEG	NEG	NEG
MSSA2019	NEG	POS	POS	POS	NEG	POS	POS	POS	NEG	POS	POS	NEG	NEG	NEG
MSSA2029	NEG	POS	POS	POS	NEG	POS	POS	POS	NEG	POS	POS	NEG	NEG	NEG
MSSA2045	NEG	POS	POS	POS	NEG	POS	POS	POS	NEG	POS	POS	NEG	NEG	NEG
MSSA2048	NEG	POS	POS	POS	NEG	POS	POS	POS	NEG	POS	POS	NEG	NEG	NEG
MSSA2049	NEG	POS	POS	POS	NEG	POS	POS	POS	NEG	POS	POS	NEG	NEG	NEG
MSSA2050	NEG	POS	POS	POS	NEG	POS	POS	POS	NEG	POS	POS	NEG	NEG	NEG
MSSA2099	NEG	POS	POS	POS	NEG	AMB	POS	POS	NEG	POS	POS	NEG	NEG	NEG
MSSA2109	NEG	POS	POS	POS	NEG	POS	POS	POS	NEG	POS	POS	NEG	NEG	NEG
MSSA2130	NEG	POS	POS	POS	NEG	POS	POS	POS	NEG	POS	POS	NEG	NEG	NEG
MSSA2133	NEG	POS	POS	POS	NEG	POS	POS	POS	NEG	POS	POS	NEG	NEG	NEG
MSSA2139	NEG	POS	POS	POS	NEG	POS	POS	POS	NEG	POS	POS	NEG	NEG	NEG
MSSA2141	NEG	POS	POS	POS	NEG	POS	POS	POS	NEG	POS	POS	NEG	NEG	NEG
MSSA2208	NEG	POS	POS	POS	NEG	POS	POS	POS	NEG	POS	POS	NEG	NEG	NEG
MSSA2209	NEG	POS	POS	POS	NEG	POS	POS	POS	NEG	POS	POS	NEG	NEG	NEG
MSSA1893	NEG	POS	POS	POS	NEG	POS	POS	POS	NEG	POS	NEG	POS	NEG	NEG
MSSA2105	NEG	POS	POS	POS	NEG	POS	POS	POS	NEG	POS	NEG	POS	NEG	NEG
MRSA3836	POS	NEG	POS	POS	NEG	POS	POS	NEG	POS	POS	NEG	NEG	POS	NEG
MRSA3840	POS	NEG	POS	POS	NEG	POS	POS	NEG	POS	POS	NEG	NEG	POS	NEG
MRSA4188	POS	NEG	POS	POS	NEG	POS	POS	NEG	POS	POS	NEG	NEG	POS	NEG
MSSA1814	POS	NEG	POS	POS	NEG	POS	POS	NEG	POS	NEG	NEG	NEG	NEG	NEG
MSSA1816	POS	NEG	POS	POS	NEG	POS	POS	NEG	POS	POS	NEG	NEG	POS	NEG
MSSA1825	POS	NEG	POS	POS	NEG	POS	POS	NEG	POS	POS	NEG	NEG	POS	NEG
MSSA1834	POS	NEG	POS	POS	NEG	POS	POS	NEG	POS	POS	NEG	NEG	POS	NEG
MSSA1938	POS	NEG	POS	POS	NEG	POS	POS	NEG	POS	POS	NEG	NEG	POS	NEG
MSSA1948	POS	NEG	POS	POS	NEG	POS	POS	NEG	POS	POS	NEG	NEG	POS	NEG
MSSA1951	POS	NEG	POS	POS	NEG	POS	POS	NEG	POS	POS	NEG	NEG	POS	NEG
MSSA2005	POS	NEG	POS	POS	NEG	POS	POS	NEG	POS	POS	NEG	NEG	POS	NEG
MSSA2051	POS	NEG	POS	POS	NEG	POS	POS	NEG	POS	POS	NEG	NEG	POS	NEG
MSSA2056	POS	NEG	POS	POS	NEG	POS	POS	NEG	POS	POS	NEG	NEG	POS	NEG
MSSA2066	POS	NEG	POS	POS	NEG	POS	POS	NEG	POS	POS	NEG	NEG	POS	NEG
MSSA2067	POS	NEG	POS	POS	NEG	POS	POS	NEG	POS	NEG	NEG	NEG	NEG	NEG
MSSA2086	POS	NEG	POS	POS	NEG	POS	POS	NEG	POS	POS	NEG	NEG	POS	NEG
MSSA2089	POS	NEG	POS	POS	NEG	POS	POS	NEG	POS	POS	NEG	NEG	POS	NEG
MSSA2103	POS	NEG	POS	POS	NEG	POS	POS	NEG	POS	POS	NEG	NEG	POS	NEG
MSSA2173	POS	NEG	POS	POS	NEG	POS	POS	NEG	POS	POS	NEG	NEG	POS	NEG
MSSA2183	POS	NEG	POS	POS	NEG	POS	POS	NEG	POS	POS	NEG	NEG	POS	NEG
MSSA2196	POS	NEG	POS	POS	NEG	POS	POS	NEG	POS	POS	NEG	NEG	POS	NEG
MSSA2219	POS	NEG	POS	POS	NEG	POS	POS	NEG	POS	POS	NEG	NEG	POS	NEG
MSSA1753	NEG	POS	POS	POS	NEG	POS	POS	POS	NEG	POS	NEG	NEG	POS	NEG
MSSA1773	NEG	POS	POS	POS	NEG	POS	POS	POS	NEG	POS	NEG	NEG	POS	NEG
MSSA1811	NEG	POS	POS	POS	NEG	POS	POS	POS	NEG	POS	NEG	NEG	POS	NEG
MSSA1897	NEG	POS	POS	POS	NEG	POS	POS	POS	NEG	POS	NEG	NEG	POS	NEG
MSSA1931	NEG	POS	POS	POS	NEG	POS	POS	POS	NEG	POS	NEG	NEG	POS	NEG
MSSA2114	NEG	POS	POS	POS	NEG	POS	POS	POS	NEG	POS	NEG	NEG	POS	NEG
MSSA1745	POS	POS	POS	POS	NEG	NEG	POS	NEG	POS	POS	NEG	POS	NEG	NEG
MSSA1747	POS	POS	POS	POS	NEG	NEG	POS	NEG	POS	POS	NEG	POS	NEG	NEG
MSSA1749	POS	POS	POS	POS	NEG	NEG	POS	NEG	POS	POS	NEG	POS	NEG	NEG
MSSA1768	POS	POS	POS	POS	NEG	NEG	POS	NEG	POS	POS	NEG	POS	NEG	NEG
MSSA1774	POS	POS	POS	POS	NEG	NEG	POS	NEG	POS	POS	NEG	POS	NEG	NEG
MSSA1775	POS	POS	POS	POS	NEG	NEG	POS	NEG	POS	POS	NEG	POS	NEG	NEG
MSSA1776	POS	POS	POS	POS	NEG	NEG	POS	NEG	POS	POS	NEG	POS	NEG	NEG
MSSA1779	POS	POS	POS	POS	NEG	NEG	POS	NEG	POS	POS	NEG	POS	NEG	NEG
MSSA1780	POS	POS	POS	POS	NEG	NEG	POS	NEG	POS	POS	NEG	POS	NEG	NEG

	ADHAESION FACTORS / GENES ENCODING MICROBIAL SURFACE COMPONENTS RECOGNIZING ADHESIVE MATRIX MOLECULES (MSCRAMM GENES), continued													
Strain	cna	ebh (total)	ebpS (probe 612)	ebpS (probe 614)	ebpS (01-1111)	ebpS (COL)	eno	fib	fib (MRSA252)	fnbA (total)	fnbA (COL)	fnbA (MRSA252)	fnbA (Mu50+ MW2)	fnbA (RF122)
~~MSSA1781~~	POS	POS	POS	POS	NEG	NEG	POS	NEG	POS	POS	NEG	POS	NEG	NEG
MSSA1786	POS	POS	POS	POS	NEG	NEG	POS	NEG	POS	POS	NEG	POS	NEG	NEG
MSSA1793	POS	POS	POS	POS	NEG	NEG	POS	NEG	POS	POS	NEG	POS	NEG	NEG
MSSA1803	POS	POS	POS	POS	NEG	NEG	POS	NEG	POS	POS	NEG	POS	NEG	NEG
MSSA1836	POS	POS	POS	POS	NEG	NEG	POS	NEG	POS	POS	NEG	POS	NEG	NEG
MSSA1839	POS	POS	POS	POS	NEG	NEG	POS	NEG	POS	POS	NEG	POS	NEG	NEG
MSSA1900	POS	POS	POS	POS	NEG	NEG	POS	NEG	POS	POS	NEG	POS	NEG	NEG
MSSA1910	NEG	POS	POS	POS	NEG	NEG	POS	NEG	POS	POS	NEG	POS	NEG	NEG
MSSA1918	NEG	POS	POS	POS	NEG	NEG	POS	NEG	POS	POS	NEG	POS	NEG	NEG
MSSA1921	POS	POS	POS	POS	NEG	NEG	POS	NEG	POS	POS	NEG	POS	NEG	NEG
MSSA1923	POS	POS	POS	POS	NEG	NEG	POS	NEG	POS	POS	NEG	POS	NEG	NEG
MSSA1981	NEG	POS	POS	POS	NEG	NEG	POS	NEG	POS	POS	NEG	POS	NEG	NEG
MSSA1998	POS	POS	POS	POS	NEG	NEG	POS	NEG	POS	POS	NEG	POS	NEG	NEG
MSSA2000	NEG	POS	POS	POS	NEG	NEG	POS	NEG	POS	POS	NEG	POS	NEG	NEG
MSSA2007	POS	POS	POS	POS	NEG	NEG	POS	NEG	POS	POS	NEG	POS	NEG	NEG
MSSA2012	POS	POS	POS	POS	NEG	NEG	POS	NEG	POS	POS	NEG	POS	NEG	NEG
MSSA2015	POS	POS	POS	POS	NEG	NEG	POS	NEG	POS	POS	NEG	POS	NEG	NEG
MSSA2021	POS	POS	POS	POS	NEG	NEG	POS	NEG	POS	POS	NEG	POS	NEG	NEG
MSSA2027	POS	POS	POS	POS	NEG	NEG	POS	NEG	POS	POS	NEG	POS	NEG	NEG
MSSA2063	POS	POS	POS	POS	NEG	NEG	POS	NEG	POS	POS	NEG	POS	NEG	NEG
MSSA2064	POS	POS	POS	POS	NEG	NEG	POS	NEG	POS	POS	NEG	POS	NEG	NEG
MSSA2065	POS	POS	POS	POS	NEG	NEG	POS	NEG	POS	POS	NEG	POS	NEG	NEG
MSSA2075	POS	POS	POS	POS	NEG	NEG	POS	NEG	POS	POS	NEG	POS	NEG	NEG
MSSA2093	NEG	POS	POS	POS	NEG	NEG	POS	NEG	POS	POS	NEG	POS	NEG	NEG
MSSA2102	POS	POS	POS	POS	NEG	NEG	POS	NEG	POS	POS	NEG	POS	NEG	NEG
MSSA2138	POS	POS	POS	POS	NEG	NEG	POS	NEG	POS	POS	NEG	POS	NEG	NEG
MSSA2168	POS	POS	POS	POS	NEG	NEG	POS	NEG	POS	POS	NEG	POS	NEG	NEG
MSSA2181	POS	POS	POS	POS	NEG	NEG	POS	NEG	POS	POS	NEG	POS	NEG	NEG
MSSA2189	NEG	POS	POS	POS	NEG	NEG	POS	NEG	POS	POS	NEG	POS	NEG	NEG
MSSA2214	POS	POS	POS	POS	NEG	NEG	POS	NEG	POS	POS	NEG	POS	NEG	NEG
MRSA3803	POS	POS	NEG	POS	POS	NEG	POS	NEG	POS	POS	NEG	NEG	POS	NEG
MSSA1750	POS	POS	NEG	POS	POS	POS	POS	NEG	POS	POS	NEG	NEG	POS	NEG
MSSA1756	POS	POS	NEG	POS	POS	NEG	POS	NEG	POS	POS	NEG	NEG	POS	NEG
MSSA1759	POS	POS	NEG	POS	POS	NEG	POS	NEG	POS	POS	NEG	NEG	POS	NEG
MSSA1767	POS	POS	NEG	POS	POS	NEG	POS	NEG	POS	POS	NEG	NEG	POS	NEG
MSSA1787	POS	POS	NEG	POS	POS	NEG	POS	NEG	POS	POS	NEG	NEG	POS	NEG
MSSA1790	POS	POS	NEG	POS	POS	NEG	POS	NEG	POS	POS	NEG	NEG	POS	NEG
MSSA1794	POS	POS	NEG	POS	POS	NEG	POS	NEG	POS	POS	NEG	NEG	POS	NEG
MSSA1808	POS	POS	NEG	POS	POS	NEG	POS	NEG	POS	POS	NEG	NEG	POS	NEG
MSSA1812	POS	POS	NEG	POS	POS	NEG	POS	NEG	POS	POS	NEG	NEG	POS	NEG
MSSA1819	POS	POS	NEG	POS	POS	NEG	POS	NEG	POS	POS	NEG	NEG	POS	NEG
MSSA1824	POS	POS	NEG	POS	POS	NEG	POS	NEG	POS	POS	NEG	NEG	POS	NEG
MSSA1827	POS	POS	NEG	POS	POS	NEG	POS	NEG	POS	POS	NEG	NEG	POS	NEG
MSSA1889	POS	POS	NEG	POS	POS	NEG	POS	NEG	POS	POS	NEG	NEG	POS	NEG
MSSA1895	POS	POS	NEG	POS	POS	NEG	POS	NEG	POS	POS	NEG	NEG	POS	NEG
MSSA1904	POS	POS	NEG	POS	POS	NEG	POS	NEG	POS	POS	NEG	NEG	POS	NEG
MSSA1905	POS	POS	NEG	POS	POS	NEG	POS	NEG	POS	POS	NEG	NEG	POS	NEG
MSSA1907	POS	POS	NEG	POS	POS	NEG	POS	NEG	AMB	POS	NEG	NEG	POS	NEG
MSSA1919	POS	POS	NEG	POS	POS	NEG	POS	NEG	POS	POS	NEG	NEG	POS	NEG
MSSA1920	POS	POS	NEG	POS	POS	NEG	POS	NEG	POS	POS	NEG	NEG	POS	NEG
MSSA1929	POS	POS	NEG	POS	POS	NEG	POS	NEG	POS	POS	NEG	NEG	POS	NEG
MSSA1930	POS	POS	NEG	POS	POS	NEG	POS	NEG	POS	POS	NEG	NEG	POS	NEG
MSSA1934	POS	POS	NEG	POS	POS	NEG	POS	NEG	POS	POS	NEG	NEG	POS	NEG
MSSA1946	POS	POS	NEG	POS	POS	NEG	POS	NEG	POS	POS	NEG	NEG	POS	NEG
MSSA1971	POS	POS	NEG	POS	POS	NEG	POS	NEG	POS	POS	NEG	NEG	POS	NEG
MSSA1980	POS	POS	NEG	POS	POS	NEG	POS	NEG	POS	POS	NEG	NEG	POS	NEG
MSSA1982	POS	POS	NEG	POS	POS	NEG	POS	NEG	POS	POS	NEG	NEG	POS	NEG
MSSA1996	POS	POS	NEG	POS	POS	NEG	POS	NEG	POS	POS	NEG	NEG	POS	NEG
MSSA2002	POS	POS	NEG	POS	POS	NEG	POS	NEG	POS	POS	NEG	NEG	POS	NEG
MSSA2014	POS	POS	NEG	POS	POS	NEG	POS	NEG	POS	POS	NEG	NEG	POS	NEG
MSSA2053	POS	POS	NEG	POS	POS	NEG	POS	NEG	POS	POS	NEG	NEG	POS	NEG
MSSA2054	POS	POS	NEG	POS	POS	NEG	POS	NEG	POS	POS	NEG	NEG	POS	NEG
MSSA2058	POS	POS	NEG	POS	POS	NEG	POS	NEG	AMB	POS	NEG	NEG	POS	NEG
MSSA2068	POS	POS	NEG	POS	POS	NEG	POS	NEG	POS	POS	NEG	NEG	POS	NEG
MSSA2070	POS	POS	NEG	POS	POS	NEG	POS	NEG	POS	POS	NEG	NEG	POS	NEG
MSSA2072	POS	POS	NEG	POS	POS	NEG	POS	NEG	POS	POS	NEG	NEG	POS	NEG
MSSA2073	POS	POS	NEG	POS	POS	NEG	POS	NEG	AMB	POS	NEG	NEG	POS	NEG
MSSA2074	POS	POS	NEG	POS	POS	NEG	POS	NEG	POS	POS	NEG	NEG	POS	NEG
MSSA2107	POS	POS	NEG	POS	POS	NEG	POS	NEG	POS	POS	NEG	NEG	POS	NEG
MSSA2108	POS	POS	NEG	POS	POS	NEG	POS	NEG	POS	POS	NEG	NEG	POS	NEG
MSSA2129	POS	POS	NEG	POS	POS	NEG	POS	NEG	POS	POS	NEG	NEG	POS	NEG
MSSA2135	POS	POS	NEG	POS	POS	NEG	POS	NEG	POS	POS	NEG	NEG	POS	NEG
MSSA2137	POS	POS	NEG	POS	POS	NEG	POS	NEG	POS	POS	NEG	POS	NEG	NEG
MSSA2140	POS	POS	NEG	POS	POS	NEG	POS	NEG	POS	POS	NEG	NEG	POS	NEG
MSSA2182	POS	POS	NEG	POS	POS	NEG	POS	NEG	POS	POS	NEG	NEG	POS	NEG
MSSA2188	POS	POS	NEG	POS	POS	NEG	POS	NEG	POS	POS	NEG	NEG	POS	NEG
MSSA1899	NEG	POS	POS	POS	NEG	POS	POS	POS	NEG	POS	NEG	POS	NEG	NEG
MSSA1915	NEG	POS	POS	POS	NEG	POS	POS	POS	NEG	POS	NEG	POS	NEG	NEG
MSSA1949	NEG	POS	POS	POS	NEG	POS	POS	POS	NEG	POS	NEG	POS	NEG	NEG
MSSA2042	NEG	POS	POS	POS	NEG	POS	POS	POS	NEG	POS	NEG	POS	NEG	NEG
MSSA2077	NEG	POS	POS	POS	NEG	POS	POS	POS	NEG	POS	NEG	POS	NEG	NEG
MSSA2095	AMB	POS	POS	POS	NEG	POS	POS	POS	NEG	POS	NEG	POS	NEG	NEG
MSSA1997	NEG	POS	POS	POS	NEG	POS	POS	POS	NEG	POS	NEG	NEG	NEG	NEG
MSSA1810	NEG	POS	POS	POS	NEG	NEG	POS	POS	NEG	POS	NEG	POS	NEG	NEG
MSSA1832	NEG	POS	POS	POS	NEG	NEG	POS	POS	NEG	POS	NEG	NEG	NEG	NEG
MSSA1891	NEG	POS	POS	POS	AMB	NEG	POS	POS	NEG	POS	NEG	NEG	NEG	NEG
MSSA1892	NEG	POS	POS	POS	NEG	NEG	POS	POS	NEG	POS	NEG	NEG	NEG	NEG
MSSA1932	NEG	POS	POS	POS	NEG	NEG	POS	POS	NEG	POS	NEG	NEG	NEG	NEG
MSSA1942	NEG	POS	POS	POS	NEG	NEG	POS	POS	NEG	POS	NEG	NEG	NEG	NEG
MSSA1957	NEG	POS	POS	POS	NEG	NEG	POS	POS	NEG	POS	NEG	NEG	NEG	NEG
MSSA1748	NEG	POS	POS	POS	NEG	NEG	POS	POS	NEG	POS	NEG	POS	NEG	NEG
MSSA1754	NEG	POS	POS	POS	NEG	NEG	POS	POS	NEG	POS	NEG	POS	NEG	NEG
MSSA1770	NEG	POS	POS	POS	NEG	NEG	POS	POS	NEG	POS	NEG	POS	NEG	NEG
MSSA1788	NEG	POS	POS	POS	NEG	NEG	POS	POS	NEG	POS	NEG	POS	NEG	NEG
MSSA1804	NEG	POS	POS	POS	NEG	NEG	POS	POS	NEG	POS	NEG	POS	NEG	NEG
MSSA1818	NEG	POS	POS	POS	NEG	NEG	POS	POS	NEG	POS	NEG	POS	NEG	NEG
MSSA1914	NEG	POS	POS	POS	NEG	AMB	POS	POS	NEG	POS	NEG	POS	NEG	NEG
MSSA1974	NEG	POS	POS	POS	NEG	NEG	POS	POS	NEG	POS	NEG	POS	NEG	NEG
MSSA2023	NEG	POS	POS	POS	NEG	NEG	POS	POS	NEG	POS	NEG	POS	NEG	NEG
MSSA2030	NEG	POS	POS	POS	NEG	NEG	POS	POS	NEG	POS	NEG	POS	NEG	NEG
MSSA2096	NEG	POS	AMB	POS	NEG	NEG	POS	POS	NEG	POS	NEG	POS	NEG	NEG
MSSA2142	NEG	POS	POS	POS	NEG	NEG	POS	POS	NEG	POS	NEG	POS	NEG	NEG
MSSA2203	NEG	POS	POS	POS	NEG	AMB	POS	POS	NEG	POS	NEG	POS	NEG	NEG
MSSA1901	POS	POS	NEG	POS	POS	NEG	POS	POS	NEG	POS	NEG	NEG	NEG	NEG
MSSA2013	POS	POS	NEG	POS	POS	NEG	POS	POS	NEG	POS	NEG	NEG	NEG	NEG
MSSA2031	POS	POS	NEG	POS	POS	AMB	POS	POS	NEG	POS	NEG	NEG	NEG	NEG
MSSA2052	POS	POS	NEG	POS	POS	NEG	POS	POS	NEG	POS	NEG	NEG	NEG	NEG
MSSA2057	POS	POS	NEG	POS	POS	NEG	POS	POS	NEG	POS	NEG	NEG	NEG	NEG
MSSA2081	POS	POS	POS	POS	NEG	NEG	POS	POS	NEG	POS	NEG	NEG	NEG	NEG
MSSA1955	POS	POS	NEG	POS	POS	NEG	POS	POS	NEG	POS	POS	NEG	NEG	NEG
MSSA1941	POS	POS	POS	POS	NEG	NEG	POS	NEG	POS	POS	NEG	NEG	NEG	NEG
MRSA3838	POS	POS	NEG	POS	AMB	POS	POS	AMB	POS	POS	NEG	NEG	NEG	NEG
MRSA3839	POS	POS	NEG	POS	POS	POS	POS	NEG	POS	POS	NEG	NEG	NEG	NEG
MSSA1801	NEG	POS	NEG	NEG	AMB	NEG	POS	NEG	POS	AMB	NEG	NEG	NEG	NEG
MSSA2131	POS	POS	NEG	POS	POS	POS	POS	NEG	POS	POS	NEG	NEG	NEG	NEG
MSSA1792	NEG	POS	POS	POS	NEG	NEG	POS	POS	NEG	POS	POS	NEG	NEG	NEG
MSSA1796	NEG	POS	POS	POS	NEG	NEG	POS	POS	NEG	POS	NEG	POS	NEG	NEG
MSSA1817	NEG	POS	POS	POS	NEG	POS	POS	POS	NEG	POS	POS	NEG	NEG	NEG
MSSA1911	NEG	NEG	NEG	NEG	NEG	NEG	POS	NEG	NEG	POS	NEG	NEG	NEG	NEG
MSSA1927	POS	POS	POS	POS	NEG	NEG	POS	POS	NEG	POS	NEG	POS	NEG	NEG
MSSA1928	POS	POS	POS	POS	NEG	NEG	POS	POS	NEG	POS	NEG	POS	NEG	NEG
MSSA1953	POS	POS	POS	POS	NEG	NEG	POS	POS	NEG	POS	NEG	POS	NEG	NEG
MSSA2062	NEG	POS	POS	POS	NEG	NEG	POS	POS	NEG	POS	POS	NEG	NEG	NEG
MSSA2078	NEG	POS	NEG	POS	POS	NEG	POS	NEG	POS	POS	NEG	NEG	POS	NEG
MSSA2136	POS	POS	NEG	POS	POS	POS	POS	POS	NEG	POS	NEG	NEG	NEG	NEG

	ADHAESION FACTORS / GENES ENCODING MICROBIAL SURFACE COMPONENTS RECOGNIZING ADHESIVE MATRIX MOLECULES (MSCRAMM GENES), continued											
Strain	fnbB (COL)	fnbB (COL+ Mu50+ MW2)	fnbB (Mu50)	fnbB (MW2)	fnbB (ST15)	fnbB (ST45-2)	map (COL)	map (MRSA252)	map (Mu50+ MW2)	sasG (COL+Mu50)	sasG (MW2)	sasG (Other than MRSA252+ RF122)
MSSA1805	NEG	NEG	NEG	NEG	NEG	NEG	NEG	NEG	POS	NEG	POS	POS
MSSA1823	NEG	AMB	NEG	POS	NEG	NEG	NEG	NEG	NEG	NEG	POS	POS
MSSA1837	NEG	NEG	NEG	NEG	NEG	NEG	NEG	NEG	POS	NEG	POS	POS
MSSA2044	NEG	AMB	NEG	POS	NEG	NEG	NEG	NEG	POS	NEG	POS	POS
MSSA2046	NEG	AMB	NEG	POS	NEG	NEG	NEG	NEG	POS	NEG	POS	POS
MSSA2059	NEG	AMB	NEG	POS	NEG	NEG	NEG	NEG	NEG	NEG	POS	POS
MSSA2076	NEG	AMB	NEG	POS	NEG	NEG	NEG	NEG	NEG	NEG	POS	POS
MSSA2200	NEG	AMB	NEG	POS	NEG	NEG	NEG	NEG	NEG	NEG	POS	POS
MRSA3797	NEG	POS	AMB	NEG	NEG	NEG	NEG	NEG	POS	POS	NEG	POS
MRSA3798	NEG	AMB	POS	NEG	NEG	NEG	POS	NEG	POS	POS	NEG	POS
MRSA3799	NEG	POS	AMB	NEG	NEG	NEG	NEG	NEG	POS	POS	NEG	POS
MRSA3800	NEG	AMB	POS	NEG	AMB	NEG	POS	NEG	POS	POS	NEG	POS
MRSA3801	NEG	AMB	POS	NEG	AMB	NEG	POS	NEG	POS	POS	NEG	POS
MRSA3804	NEG	AMB	POS	NEG	NEG	NEG	POS	NEG	POS	POS	NEG	POS
MRSA3806	NEG	AMB	POS	NEG	NEG	NEG	AMB	NEG	POS	POS	NEG	POS
MRSA3961	NEG	AMB	POS	NEG	NEG	NEG	AMB	NEG	POS	POS	NEG	POS
MRSA3962	NEG	AMB	POS	NEG	NEG	NEG	AMB	NEG	POS	POS	NEG	POS
MRSA3963	NEG	AMB	POS	NEG	NEG	NEG	AMB	NEG	POS	POS	NEG	POS
MRSA3966	NEG	POS	AMB	NEG	NEG	NEG	NEG	NEG	POS	POS	NEG	POS
MRSA3978	NEG	POS	AMB	NEG	NEG	NEG	AMB	NEG	POS	POS	NEG	POS
MRSA3979	NEG	POS	AMB	NEG	NEG	NEG	NEG	NEG	POS	POS	NEG	POS
MRSA3980	NEG	POS	AMB	NEG	NEG	NEG	NEG	NEG	POS	POS	NEG	POS
MRSA4179	NEG	POS	AMB	NEG	NEG	NEG	NEG	NEG	POS	POS	NEG	POS
MRSA4216	NEG	POS	AMB	NEG	NEG	NEG	AMB	NEG	POS	POS	NEG	POS
MRSA4217	NEG	AMB	POS	NEG	NEG	NEG	POS	NEG	POS	POS	NEG	POS
MRSA4220	NEG	AMB	POS	NEG	NEG	NEG	AMB	NEG	POS	POS	NEG	POS
MRSA4221	NEG	AMB	POS	NEG	NEG	NEG	POS	NEG	POS	POS	NEG	POS
MRSA4222	NEG	AMB	POS	NEG	NEG	NEG	AMB	NEG	POS	POS	NEG	POS
MSSA1751	NEG	AMB	POS	NEG	AMB	NEG	POS	NEG	POS	POS	NEG	POS
MSSA1760	NEG	AMB	POS	NEG	AMB	NEG	POS	NEG	POS	POS	NEG	POS
MSSA1802	NEG	POS	AMB	NEG	NEG	NEG	AMB	NEG	POS	POS	NEG	POS
MSSA1809	NEG	POS	AMB	NEG	NEG	NEG	AMB	NEG	POS	POS	NEG	POS
MSSA1815	NEG	AMB	POS	NEG	NEG	NEG	POS	NEG	POS	POS	NEG	POS
MSSA1829	NEG	POS	AMB	NEG	NEG	NEG	AMB	NEG	POS	POS	NEG	POS
MSSA1908	NEG	AMB	POS	NEG	NEG	NEG	POS	NEG	POS	POS	NEG	POS
MSSA1960	NEG	POS	AMB	NEG	NEG	NEG	AMB	NEG	POS	POS	NEG	POS
MSSA1965	NEG	POS	AMB	NEG	NEG	NEG	AMB	NEG	POS	POS	NEG	POS
MSSA1977	NEG	POS	AMB	NEG	NEG	NEG	AMB	NEG	POS	POS	NEG	POS
MSSA1978	NEG	AMB	POS	NEG	NEG	NEG	POS	NEG	POS	POS	NEG	POS
MSSA1999	NEG	POS	AMB	NEG	NEG	NEG	AMB	NEG	POS	POS	NEG	POS
MSSA2003	NEG	POS	AMB	NEG	NEG	NEG	AMB	NEG	POS	POS	NEG	POS
MSSA2006	NEG	POS	AMB	NEG	NEG	NEG	NEG	NEG	POS	POS	NEG	POS
MSSA2008	NEG	POS	AMB	NEG	NEG	NEG	AMB	NEG	POS	POS	NEG	POS
MSSA2011	NEG	POS	AMB	NEG	NEG	NEG	AMB	NEG	POS	POS	NEG	POS
MSSA2017	NEG	POS	AMB	NEG	NEG	NEG	NEG	NEG	POS	POS	NEG	POS
MSSA2022	NEG	POS	AMB	NEG	NEG	NEG	AMB	NEG	POS	POS	NEG	POS
MSSA2055	NEG	POS	AMB	NEG	NEG	NEG	NEG	NEG	POS	POS	NEG	POS
MSSA2060	NEG	POS	AMB	NEG	NEG	NEG	AMB	NEG	POS	POS	NEG	POS
MSSA2080	NEG	POS	AMB	NEG	NEG	NEG	NEG	NEG	POS	POS	NEG	POS
MSSA2082	NEG	AMB	POS	NEG	AMB	NEG	POS	AMB	POS	POS	NEG	POS
MSSA2084	NEG	POS	AMB	NEG	NEG	NEG	NEG	NEG	POS	POS	NEG	POS
MSSA2087	NEG	POS	AMB	NEG	NEG	NEG	NEG	NEG	POS	POS	NEG	POS
MSSA2094	NEG	POS	AMB	NEG	NEG	NEG	AMB	NEG	POS	POS	NEG	POS
MSSA2097	NEG	POS	AMB	NEG	NEG	NEG	AMB	NEG	POS	POS	NEG	POS
MSSA2110	NEG	AMB	POS	NEG	NEG	NEG	AMB	NEG	POS	POS	NEG	POS
MSSA2113	NEG	NEG	POS	NEG	NEG	NEG	AMB	NEG	POS	POS	NEG	POS
MSSA2132	NEG	AMB	POS	NEG	AMB	NEG	POS	NEG	POS	POS	NEG	POS
MSSA2134	NEG	POS	AMB	NEG	NEG	NEG	NEG	NEG	POS	POS	NEG	POS
MSSA1746	NEG	AMB	NEG	NEG	NEG	POS	NEG	NEG	POS	NEG	NEG	NEG
MSSA1757	NEG	AMB	NEG	NEG	NEG	POS	NEG	NEG	POS	NEG	NEG	NEG
MSSA1772	NEG	AMB	NEG	NEG	NEG	POS	NEG	NEG	POS	NEG	NEG	NEG
MSSA1795	NEG	AMB	NEG	NEG	NEG	POS	NEG	NEG	POS	NEG	NEG	NEG
MSSA1813	NEG	AMB	NEG	NEG	NEG	POS	NEG	NEG	POS	NEG	NEG	NEG
MSSA1826	NEG	AMB	NEG	NEG	NEG	POS	NEG	NEG	POS	NEG	NEG	NEG
MSSA1830	NEG	AMB	NEG	NEG	NEG	POS	NEG	NEG	POS	NEG	NEG	NEG
MSSA1838	NEG	AMB	NEG	NEG	NEG	POS	NEG	NEG	POS	NEG	NEG	NEG
MSSA1944	NEG	AMB	NEG	NEG	NEG	POS	NEG	NEG	POS	NEG	NEG	NEG
MSSA1947	NEG	AMB	NEG	NEG	NEG	POS	NEG	NEG	POS	NEG	NEG	NEG
MSSA1964	NEG	AMB	NEG	NEG	NEG	POS	NEG	NEG	POS	NEG	NEG	NEG
MSSA1969	NEG	AMB	NEG	NEG	NEG	POS	NEG	NEG	POS	NEG	NEG	NEG
MSSA2001	NEG	AMB	NEG	NEG	NEG	POS	NEG	NEG	POS	NEG	NEG	NEG
MSSA2004	NEG	AMB	NEG	NEG	NEG	POS	NEG	NEG	POS	NEG	NEG	NEG
MSSA2024	NEG	AMB	NEG	NEG	NEG	POS	NEG	NEG	POS	NEG	NEG	NEG
MSSA2025	NEG	AMB	NEG	NEG	NEG	POS	NEG	NEG	POS	NEG	NEG	NEG
MSSA2026	NEG	AMB	NEG	NEG	NEG	POS	NEG	NEG	POS	NEG	NEG	NEG
MSSA2032	NEG	AMB	NEG	NEG	NEG	POS	NEG	NEG	POS	NEG	NEG	NEG
MSSA2043	NEG	AMB	NEG	NEG	NEG	POS	NEG	NEG	POS	NEG	NEG	NEG
MSSA2061	NEG	AMB	NEG	NEG	NEG	POS	NEG	NEG	POS	NEG	NEG	NEG
MSSA2085	NEG	AMB	NEG	NEG	NEG	POS	NEG	NEG	POS	NEG	NEG	NEG
MSSA2098	NEG	AMB	NEG	NEG	NEG	POS	NEG	NEG	POS	NEG	NEG	NEG
MSSA2100	NEG	AMB	NEG	NEG	NEG	POS	NEG	NEG	POS	NEG	NEG	NEG
MSSA2111	NEG	AMB	NEG	NEG	NEG	POS	NEG	NEG	POS	NEG	NEG	NEG
MSSA2178	NEG	AMB	NEG	NEG	NEG	POS	NEG	NEG	POS	NEG	NEG	NEG
MSSA2202	NEG	AMB	NEG	NEG	NEG	POS	NEG	NEG	POS	NEG	NEG	NEG
MSSA2205	NEG	AMB	NEG	NEG	NEG	POS	NEG	NEG	POS	NEG	NEG	NEG
MRSA3796	POS	AMB	AMB	NEG	NEG	NEG	POS	NEG	AMB	POS	NEG	POS
MRSA3805	NEG	NEG	NEG	NEG	NEG	NEG	POS	NEG	AMB	POS	NEG	POS
MRSA3965	POS	AMB	NEG	NEG	NEG	NEG	POS	NEG	NEG	POS	NEG	POS
MSSA1761	POS	AMB	AMB	AMB	NEG	NEG	POS	NEG	POS	POS	NEG	POS
MSSA1764	POS	AMB	NEG	NEG	NEG	NEG	POS	NEG	NEG	POS	NEG	POS
MSSA1765	NEG	NEG	NEG	NEG	NEG	NEG	POS	NEG	NEG	POS	NEG	POS
MSSA1784	POS	AMB	AMB	NEG	NEG	NEG	POS	NEG	AMB	POS	NEG	POS
MSSA1806	POS	AMB	NEG	NEG	NEG	NEG	POS	NEG	NEG	POS	NEG	POS
MSSA1807	POS	AMB	NEG	NEG	NEG	NEG	POS	NEG	NEG	POS	NEG	POS
MSSA1888	POS	AMB	NEG	NEG	NEG	NEG	POS	NEG	NEG	POS	NEG	POS
MSSA1894	POS	AMB	NEG	NEG	NEG	NEG	POS	NEG	NEG	POS	NEG	POS
MSSA1922	POS	AMB	NEG	NEG	NEG	NEG	POS	NEG	NEG	POS	NEG	POS
MSSA1950	POS	AMB	NEG	NEG	NEG	NEG	POS	NEG	NEG	POS	NEG	POS
MSSA1954	POS	AMB	NEG	NEG	NEG	NEG	POS	NEG	NEG	POS	NEG	POS
MSSA1966	POS	AMB	NEG	NEG	NEG	NEG	POS	NEG	NEG	POS	NEG	POS
MSSA1972	POS	AMB	NEG	NEG	NEG	NEG	POS	NEG	NEG	POS	NEG	POS
MSSA2010	POS	AMB	NEG	NEG	NEG	NEG	POS	NEG	NEG	POS	NEG	POS
MSSA2020	NEG	NEG	NEG	NEG	NEG	NEG	POS	NEG	NEG	POS	NEG	POS
MSSA2028	POS	AMB	AMB	NEG	NEG	NEG	POS	NEG	AMB	POS	NEG	POS
MSSA2088	POS	AMB	AMB	NEG	NEG	NEG	POS	NEG	AMB	POS	NEG	POS
MSSA2187	POS	AMB	AMB	NEG	NEG	NEG	POS	NEG	NEG	POS	NEG	POS
MSSA2204	POS	AMB	AMB	NEG	NEG	NEG	POS	NEG	POS	POS	NEG	POS
MSSA2210	POS	AMB	NEG	NEG	NEG	NEG	POS	NEG	NEG	POS	NEG	POS
MSSA2223	POS	AMB	NEG	NEG	NEG	NEG	POS	NEG	NEG	POS	NEG	POS
MSSA1763	NEG	NEG	NEG	NEG	NEG	NEG	POS	NEG	POS	NEG	NEG	NEG
MSSA1777	NEG	AMB	POS	NEG	NEG	NEG	POS	POS	POS	NEG	NEG	NEG
MSSA2009	NEG	POS	NEG	NEG	NEG	NEG	POS	NEG	POS	NEG	NEG	NEG
MSSA2047	NEG	POS	AMB	NEG	NEG	NEG	POS	NEG	POS	NEG	NEG	NEG
MSSA2079	NEG	POS	NEG	NEG	NEG	NEG	POS	NEG	POS	NEG	NEG	NEG
MSSA2101	NEG	NEG	NEG	NEG	NEG	NEG	POS	NEG	POS	NEG	NEG	NEG
MSSA1821	NEG	AMB	NEG	NEG	NEG	POS	POS	NEG	POS	NEG	NEG	NEG
MSSA1898	NEG	AMB	NEG	NEG	NEG	POS	POS	NEG	POS	NEG	NEG	NEG
MSSA1973	NEG	AMB	NEG	NEG	NEG	POS	POS	NEG	POS	NEG	NEG	NEG
MSSA2071	NEG	AMB	NEG	NEG	NEG	POS	POS	NEG	POS	NEG	NEG	NEG
MSSA2083	NEG	AMB	NEG	NEG	NEG	POS	POS	NEG	POS	NEG	NEG	NEG
MSSA2106	NEG	AMB	NEG	NEG	NEG	POS	POS	NEG	POS	NEG	NEG	NEG
MSSA2112	NEG	AMB	NEG	NEG	NEG	POS	POS	NEG	POS	NEG	NEG	NEG
MSSA2115	NEG	AMB	NEG	NEG	NEG	POS	POS	NEG	POS	NEG	NEG	NEG
MSSA2195	NEG	AMB	NEG	NEG	NEG	POS	POS	NEG	POS	NEG	NEG	NEG
MSSA2207	NEG	AMB	NEG	NEG	NEG	POS	POS	NEG	POS	NEG	NEG	NEG
MSSA1758	NEG	POS	AMB	NEG	AMB	NEG	POS	NEG	POS	NEG	POS	POS
MSSA1762	NEG	AMB	POS	NEG	AMB	NEG	POS	NEG	POS	NEG	POS	POS
MSSA1769	NEG	AMB	POS	NEG	AMB	NEG	POS	NEG	POS	NEG	POS	POS
MSSA1771	NEG	POS	AMB	NEG	AMB	NEG	POS	NEG	POS	NEG	POS	POS
MSSA1789	NEG	POS	AMB	NEG	AMB	NEG	POS	NEG	POS	NEG	POS	POS
MSSA1800	NEG	POS	AMB	NEG	AMB	NEG	POS	NEG	POS	NEG	POS	POS
MSSA1833	NEG	POS	AMB	NEG	AMB	NEG	POS	NEG	POS	NEG	POS	POS
MSSA1890	NEG	POS	AMB	NEG	AMB	NEG	AMB	NEG	POS	NEG	POS	POS
MSSA1903	NEG	POS	AMB	NEG	AMB	NEG	POS	NEG	POS	NEG	POS	POS
MSSA1940	NEG	AMB	POS	NEG	AMB	NEG	POS	NEG	POS	NEG	POS	POS
MSSA1962	NEG	POS	AMB	NEG	AMB	NEG	POS	NEG	POS	NEG	POS	POS
MSSA1963	NEG	AMB	POS	NEG	AMB	NEG	POS	NEG	POS	NEG	POS	POS
MSSA2016	NEG	POS	AMB	NEG	AMB	NEG	POS	NEG	POS	NEG	POS	POS
MSSA2018	NEG	POS	AMB	NEG	AMB	NEG	POS	NEG	POS	NEG	POS	POS
MSSA2019	NEG	POS	AMB	NEG	AMB	NEG	POS	NEG	POS	NEG	POS	POS
MSSA2029	NEG	AMB	POS	NEG	AMB	NEG	POS	NEG	POS	NEG	POS	POS
MSSA2045	NEG	POS	AMB	NEG	AMB	NEG	POS	NEG	POS	NEG	POS	POS
MSSA2048	NEG	POS	AMB	NEG	AMB	NEG	POS	NEG	POS	NEG	POS	POS
MSSA2049	NEG	POS	AMB	NEG	AMB	NEG	POS	NEG	POS	NEG	POS	POS
MSSA2050	NEG	AMB	AMB	NEG	POS	NEG	POS	NEG	POS	NEG	POS	POS
MSSA2099	NEG	POS	AMB	NEG	AMB	NEG	NEG	NEG	POS	NEG	POS	POS
MSSA2109	NEG	POS	AMB	NEG	NEG	NEG	AMB	NEG	POS	NEG	POS	POS
MSSA2130	NEG	POS	AMB	NEG	AMB	NEG	POS	NEG	POS	NEG	POS	POS
MSSA2133	NEG	POS	AMB	NEG	AMB	NEG	POS	NEG	POS	NEG	POS	POS
MSSA2139	NEG	NEG	NEG	NEG	NEG	NEG	POS	NEG	POS	NEG	POS	POS
MSSA2141	NEG	AMB	POS	NEG	AMB	NEG	POS	NEG	POS	NEG	POS	POS
MSSA2208	NEG	AMB	POS	NEG	AMB	NEG	POS	NEG	POS	NEG	POS	POS
MSSA2209	NEG	AMB	POS	NEG	AMB	NEG	NEG	NEG	POS	NEG	POS	POS
MSSA1893	NEG	POS	AMB	NEG	AMB	NEG	NEG	NEG	POS	POS	NEG	POS
MSSA2105	NEG	AMB	POS	NEG	AMB	NEG	NEG	NEG	POS	POS	NEG	POS
MRSA3836	NEG	NEG	NEG	NEG	NEG	NEG	NEG	NEG	POS	NEG	POS	POS
MRSA3840	NEG	NEG	NEG	NEG	NEG	NEG	NEG	NEG	POS	NEG	POS	POS
MRSA4188	NEG	NEG	NEG	NEG	NEG	NEG	NEG	NEG	POS	NEG	POS	POS
MSSA1814	NEG	AMB	NEG	POS	NEG	NEG	NEG	NEG	POS	NEG	POS	POS
MSSA1816	NEG	AMB	NEG	POS	NEG	NEG	NEG	NEG	NEG	NEG	POS	POS
MSSA1825	NEG	NEG	NEG	NEG	NEG	NEG	NEG	NEG	POS	NEG	POS	POS
MSSA1834	NEG	AMB	NEG	POS	NEG	NEG	NEG	NEG	NEG	NEG	POS	POS
MSSA1938	NEG	AMB	NEG	POS	NEG	NEG	NEG	NEG	POS	NEG	POS	POS
MSSA1948	NEG	NEG	NEG	NEG	NEG	NEG	NEG	NEG	POS	NEG	POS	POS
MSSA1951	NEG	AMB	NEG	POS	NEG	NEG	NEG	NEG	POS	NEG	POS	POS
MSSA2005	NEG	NEG	NEG	NEG	NEG	NEG	NEG	NEG	POS	NEG	POS	POS
MSSA2051	NEG	AMB	NEG	POS	NEG	NEG	NEG	NEG	POS	NEG	POS	POS
MSSA2056	NEG	AMB	NEG	POS	NEG	NEG	NEG	NEG	POS	NEG	POS	POS
MSSA2066	NEG	AMB	NEG	POS	NEG	NEG	NEG	NEG	POS	NEG	POS	POS
MSSA2067	NEG	AMB	NEG	POS	NEG	NEG	NEG	NEG	POS	NEG	POS	POS
MSSA2086	NEG	AMB	NEG	POS	NEG	NEG	NEG	NEG	POS	NEG	POS	POS
MSSA2089	NEG	AMB	NEG	POS	NEG	NEG	NEG	NEG	POS	NEG	POS	POS
MSSA2103	NEG	AMB	NEG	POS	NEG	NEG	NEG	NEG	AMB	NEG	POS	POS
MSSA2173	NEG	AMB	NEG	POS	NEG	NEG	NEG	NEG	POS	NEG	POS	POS
MSSA2183	NEG	AMB	NEG	POS	NEG	NEG	NEG	NEG	POS	NEG	POS	POS
MSSA2196	NEG	AMB	NEG	POS	NEG	NEG	NEG	NEG	POS	NEG	POS	POS
MSSA2219	NEG	NEG	NEG	NEG	NEG	NEG	NEG	NEG	POS	NEG	POS	POS
MSSA1753	NEG	AMB	POS	NEG	NEG	NEG	POS	NEG	POS	NEG	NEG	NEG
MSSA1773	NEG	AMB	POS	NEG	NEG	NEG	AMB	NEG	POS	NEG	NEG	NEG
MSSA1811	NEG	AMB	POS	NEG	NEG	NEG	POS	NEG	POS	NEG	NEG	NEG
MSSA1897	NEG	NEG	NEG	NEG	NEG	NEG	POS	NEG	POS	NEG	NEG	NEG
MSSA1931	NEG	AMB	POS	NEG	NEG	NEG	POS	NEG	POS	NEG	NEG	NEG
MSSA2114	NEG	AMB	POS	NEG	NEG	NEG	POS	NEG	POS	NEG	NEG	NEG
MSSA1745	NEG	NEG	NEG	NEG	NEG	NEG	NEG	POS	NEG	NEG	NEG	NEG
MSSA1747	NEG	NEG	NEG	NEG	NEG	NEG	NEG	POS	NEG	NEG	NEG	NEG
MSSA1749	NEG	NEG	NEG	NEG	NEG	NEG	NEG	POS	NEG	NEG	NEG	NEG
MSSA1768	NEG	POS	AMB	NEG	NEG	NEG	NEG	POS	NEG	NEG	NEG	NEG
MSSA1774	NEG	NEG	NEG	NEG	NEG	NEG	NEG	POS	NEG	NEG	NEG	NEG
MSSA1775	NEG	AMB	POS	NEG	NEG	NEG	NEG	POS	NEG	NEG	NEG	NEG
MSSA1776	NEG	NEG	NEG	NEG	NEG	NEG	NEG	POS	NEG	NEG	NEG	NEG
MSSA1779	NEG	NEG	NEG	NEG	NEG	NEG	NEG	POS	NEG	NEG	NEG	NEG
MSSA1780	NEG	NEG	NEG	NEG	NEG	NEG	NEG	POS	NEG	NEG	NEG	NEG

	ADHAESION FACTORS / GENES ENCODING MICROBIAL SURFACE COMPONENTS RECOGNIZING ADHESIVE MATRIX MOLECULES (MSCRAMM GENES), continued											
Strain	fnbB (COL)	fnbB (COL+ Mu50+ MW2)	fnbB (Mu50)	fnbB (MW2)	fnbB (ST15)	fnbB (ST45-2)	map (COL)	map (MRSA252)	map (Mu50+ MW2)	sasG (COL+Mu50)	sasG (MW2)	sasG (Other than MRSA252+ RF122)
MSSA1781	NEG	NEG	NEG	NEG	NEG	NEG	NEG	POS	NEG	NEG	NEG	NEG
MSSA1786	NEG	NEG	NEG	NEG	NEG	NEG	NEG	POS	NEG	NEG	NEG	NEG
MSSA1793	NEG	NEG	NEG	NEG	NEG	NEG	NEG	POS	NEG	NEG	NEG	NEG
MSSA1803	NEG	NEG	NEG	NEG	NEG	NEG	NEG	POS	NEG	NEG	NEG	NEG
MSSA1836	NEG	NEG	NEG	NEG	NEG	NEG	NEG	POS	NEG	NEG	NEG	NEG
MSSA1839	NEG	NEG	NEG	NEG	NEG	NEG	NEG	POS	NEG	NEG	NEG	NEG
MSSA1900	NEG	NEG	NEG	NEG	NEG	NEG	NEG	POS	NEG	NEG	NEG	NEG
MSSA1910	NEG	NEG	NEG	NEG	NEG	NEG	NEG	POS	NEG	NEG	NEG	NEG
MSSA1918	NEG	NEG	NEG	NEG	NEG	NEG	NEG	POS	NEG	NEG	NEG	NEG
MSSA1921	NEG	AMB	POS	NEG	NEG	NEG	NEG	POS	NEG	NEG	NEG	NEG
MSSA1923	NEG	POS	AMB	NEG	NEG	NEG	NEG	POS	NEG	NEG	NEG	NEG
MSSA1981	NEG	NEG	NEG	NEG	NEG	NEG	NEG	POS	NEG	NEG	NEG	NEG
MSSA1998	NEG	NEG	NEG	NEG	NEG	NEG	NEG	POS	NEG	NEG	NEG	NEG
MSSA2000	NEG	NEG	NEG	NEG	NEG	NEG	NEG	POS	NEG	NEG	NEG	NEG
MSSA2007	NEG	NEG	NEG	NEG	NEG	NEG	NEG	POS	NEG	NEG	NEG	NEG
MSSA2012	NEG	NEG	NEG	NEG	NEG	NEG	NEG	POS	NEG	NEG	NEG	NEG
MSSA2015	NEG	NEG	NEG	NEG	NEG	NEG	NEG	POS	NEG	NEG	NEG	NEG
MSSA2021	NEG	NEG	NEG	NEG	NEG	NEG	NEG	POS	NEG	NEG	NEG	NEG
MSSA2027	NEG	NEG	NEG	NEG	NEG	NEG	NEG	POS	NEG	NEG	NEG	NEG
MSSA2063	NEG	NEG	NEG	NEG	NEG	NEG	NEG	POS	NEG	NEG	NEG	NEG
MSSA2064	NEG	NEG	NEG	NEG	NEG	NEG	NEG	POS	NEG	NEG	NEG	NEG
MSSA2065	NEG	NEG	NEG	NEG	NEG	NEG	NEG	POS	NEG	NEG	NEG	NEG
MSSA2075	NEG	NEG	NEG	NEG	NEG	NEG	NEG	POS	NEG	NEG	NEG	NEG
MSSA2093	NEG	NEG	NEG	NEG	NEG	NEG	NEG	POS	NEG	NEG	NEG	NEG
MSSA2102	NEG	NEG	NEG	NEG	NEG	NEG	NEG	POS	NEG	NEG	NEG	NEG
MSSA2138	NEG	NEG	NEG	NEG	NEG	NEG	NEG	POS	NEG	NEG	NEG	NEG
MSSA2168	NEG	NEG	NEG	NEG	NEG	NEG	NEG	POS	NEG	NEG	NEG	NEG
MSSA2181	NEG	AMB	POS	NEG	NEG	NEG	NEG	POS	NEG	NEG	NEG	NEG
MSSA2189	NEG	NEG	NEG	NEG	NEG	NEG	NEG	POS	NEG	NEG	NEG	NEG
MSSA2214	NEG	NEG	NEG	NEG	NEG	NEG	NEG	POS	NEG	NEG	NEG	NEG
MRSA3803	NEG	AMB	POS	NEG	AMB	NEG	NEG	POS	NEG	NEG	NEG	NEG
MSSA1750	NEG	AMB	POS	NEG	AMB	NEG	AMB	POS	AMB	NEG	NEG	NEG
MSSA1756	NEG	POS	AMB	NEG	AMB	NEG	NEG	POS	NEG	NEG	NEG	NEG
MSSA1759	NEG	AMB	POS	NEG	AMB	NEG	NEG	POS	NEG	NEG	NEG	NEG
MSSA1767	NEG	AMB	POS	NEG	AMB	NEG	NEG	POS	NEG	NEG	NEG	NEG
MSSA1787	NEG	NEG	NEG	NEG	NEG	NEG	NEG	NEG	NEG	NEG	NEG	NEG
MSSA1790	NEG	POS	AMB	NEG	NEG	NEG	NEG	POS	NEG	NEG	NEG	NEG
MSSA1794	NEG	POS	AMB	NEG	NEG	NEG	NEG	NEG	NEG	NEG	NEG	NEG
MSSA1808	NEG	POS	AMB	NEG	NEG	NEG	NEG	POS	NEG	NEG	NEG	NEG
MSSA1812	NEG	POS	AMB	NEG	NEG	NEG	NEG	POS	NEG	NEG	NEG	NEG
MSSA1819	NEG	POS	AMB	NEG	NEG	NEG	AMB	POS	NEG	NEG	NEG	NEG
MSSA1824	NEG	POS	AMB	NEG	NEG	NEG	NEG	POS	NEG	NEG	NEG	NEG
MSSA1827	NEG	POS	AMB	NEG	NEG	NEG	NEG	POS	NEG	NEG	NEG	NEG
MSSA1889	NEG	AMB	POS	NEG	NEG	NEG	NEG	POS	NEG	NEG	NEG	NEG
MSSA1895	NEG	POS	AMB	NEG	NEG	NEG	NEG	POS	NEG	NEG	NEG	NEG
MSSA1904	NEG	POS	AMB	NEG	NEG	NEG	NEG	POS	NEG	NEG	NEG	NEG
MSSA1905	NEG	POS	AMB	NEG	NEG	NEG	NEG	POS	NEG	NEG	NEG	NEG
MSSA1907	NEG	POS	AMB	NEG	NEG	NEG	NEG	POS	NEG	NEG	NEG	NEG
MSSA1919	NEG	POS	AMB	NEG	NEG	NEG	NEG	NEG	NEG	NEG	NEG	NEG
MSSA1920	NEG	POS	AMB	NEG	NEG	NEG	NEG	POS	NEG	NEG	NEG	NEG
MSSA1929	NEG	POS	AMB	NEG	NEG	NEG	NEG	POS	NEG	NEG	NEG	NEG
MSSA1930	NEG	POS	AMB	NEG	NEG	NEG	NEG	POS	NEG	NEG	NEG	NEG
MSSA1934	NEG	POS	AMB	NEG	NEG	NEG	NEG	POS	NEG	NEG	NEG	NEG
MSSA1946	NEG	POS	AMB	NEG	NEG	NEG	NEG	POS	NEG	NEG	NEG	NEG
MSSA1971	NEG	AMB	POS	NEG	NEG	NEG	NEG	POS	NEG	NEG	NEG	NEG
MSSA1980	NEG	POS	AMB	NEG	NEG	NEG	NEG	POS	NEG	NEG	NEG	NEG
MSSA1982	NEG	POS	AMB	NEG	NEG	NEG	NEG	POS	NEG	NEG	NEG	NEG
MSSA1996	NEG	POS	AMB	NEG	NEG	NEG	NEG	POS	NEG	NEG	NEG	NEG
MSSA2002	NEG	POS	AMB	NEG	NEG	NEG	NEG	POS	NEG	NEG	NEG	NEG
MSSA2014	NEG	POS	AMB	NEG	NEG	NEG	NEG	POS	NEG	NEG	NEG	NEG
MSSA2053	NEG	POS	AMB	NEG	NEG	NEG	NEG	NEG	NEG	NEG	NEG	NEG
MSSA2054	NEG	POS	AMB	NEG	NEG	NEG	NEG	POS	NEG	NEG	NEG	NEG
MSSA2058	NEG	POS	AMB	NEG	NEG	NEG	NEG	POS	NEG	NEG	NEG	NEG
MSSA2068	NEG	POS	AMB	NEG	AMB	NEG	NEG	POS	NEG	NEG	NEG	NEG
MSSA2070	NEG	POS	AMB	NEG	NEG	NEG	NEG	POS	NEG	NEG	NEG	NEG
MSSA2072	NEG	POS	AMB	NEG	NEG	NEG	NEG	POS	NEG	NEG	NEG	NEG
MSSA2073	NEG	AMB	POS	NEG	NEG	NEG	NEG	POS	NEG	NEG	NEG	NEG
MSSA2074	NEG	NEG	NEG	NEG	NEG	NEG	NEG	POS	NEG	NEG	NEG	NEG
MSSA2107	NEG	POS	AMB	NEG	AMB	NEG	NEG	POS	NEG	NEG	NEG	NEG
MSSA2108	NEG	POS	AMB	NEG	NEG	NEG	NEG	POS	NEG	NEG	NEG	NEG
MSSA2129	NEG	POS	AMB	NEG	NEG	NEG	NEG	POS	NEG	NEG	NEG	NEG
MSSA2135	NEG	POS	AMB	NEG	AMB	NEG	NEG	POS	NEG	NEG	NEG	NEG
MSSA2137	NEG	AMB	NEG	NEG	NEG	POS	NEG	POS	NEG	NEG	POS	POS
MSSA2140	NEG	POS	AMB	NEG	NEG	NEG	NEG	POS	NEG	NEG	NEG	NEG
MSSA2182	NEG	POS	AMB	NEG	NEG	NEG	NEG	POS	NEG	NEG	NEG	NEG
MSSA2188	NEG	POS	AMB	NEG	NEG	NEG	NEG	POS	NEG	NEG	NEG	NEG
MSSA1899	NEG	POS	NEG	NEG	NEG	NEG	POS	NEG	POS	NEG	POS	POS
MSSA1915	NEG	POS	NEG	NEG	NEG	NEG	POS	NEG	POS	NEG	POS	POS
MSSA1949	NEG	POS	NEG	NEG	NEG	NEG	POS	NEG	POS	NEG	POS	POS
MSSA2042	NEG	POS	NEG	NEG	NEG	NEG	POS	NEG	POS	NEG	POS	POS
MSSA2077	NEG	POS	NEG	NEG	NEG	NEG	POS	NEG	AMB	NEG	POS	POS
MSSA2095	NEG	POS	NEG	NEG	NEG	NEG	POS	NEG	POS	NEG	POS	POS
MSSA1997	NEG	POS	AMB	NEG	NEG	NEG	NEG	NEG	POS	NEG	POS	POS
MSSA1810	NEG	POS	AMB	NEG	NEG	NEG	NEG	NEG	NEG	POS	NEG	POS
MSSA1832	NEG	POS	AMB	NEG	NEG	NEG	POS	AMB	POS	POS	NEG	POS
MSSA1891	NEG	AMB	POS	NEG	NEG	NEG	POS	NEG	POS	POS	NEG	POS
MSSA1892	NEG	POS	AMB	NEG	NEG	NEG	POS	NEG	POS	POS	NEG	POS
MSSA1932	NEG	POS	AMB	NEG	NEG	NEG	POS	NEG	POS	POS	NEG	POS
MSSA1942	NEG	POS	AMB	NEG	NEG	NEG	POS	NEG	POS	POS	NEG	POS
MSSA1957	NEG	POS	AMB	NEG	NEG	NEG	POS	NEG	POS	POS	NEG	POS
MSSA1748	NEG	POS	NEG	NEG	NEG	NEG	NEG	NEG	POS	NEG	NEG	NEG
MSSA1754	NEG	POS	NEG	NEG	NEG	NEG	NEG	NEG	POS	NEG	NEG	NEG
MSSA1770	NEG	POS	NEG	NEG	NEG	NEG	NEG	NEG	POS	NEG	NEG	NEG
MSSA1788	NEG	POS	NEG	NEG	NEG	NEG	NEG	NEG	POS	NEG	NEG	NEG
MSSA1804	NEG	POS	NEG	NEG	NEG	NEG	NEG	NEG	POS	NEG	NEG	NEG
MSSA1818	NEG	POS	NEG	NEG	NEG	NEG	NEG	NEG	POS	NEG	NEG	NEG
MSSA1914	NEG	POS	AMB	NEG	NEG	NEG	NEG	NEG	POS	NEG	NEG	NEG
MSSA1974	NEG	POS	NEG	NEG	NEG	NEG	NEG	NEG	POS	NEG	NEG	NEG
MSSA2023	NEG	POS	NEG	NEG	NEG	NEG	NEG	NEG	POS	NEG	NEG	NEG
MSSA2030	NEG	POS	NEG	NEG	NEG	NEG	NEG	NEG	POS	NEG	NEG	NEG
MSSA2096	NEG	POS	NEG	NEG	NEG	NEG	NEG	NEG	POS	NEG	NEG	NEG
MSSA2142	NEG	POS	NEG	NEG	NEG	NEG	NEG	NEG	POS	NEG	NEG	NEG
MSSA2203	NEG	NEG	NEG	NEG	NEG	NEG	NEG	NEG	POS	NEG	NEG	NEG
MSSA1901	NEG	NEG	NEG	NEG	NEG	NEG	POS	NEG	NEG	NEG	NEG	NEG
MSSA2013	NEG	POS	NEG	NEG	NEG	NEG	POS	NEG	AMB	NEG	NEG	NEG
MSSA2031	NEG	NEG	NEG	NEG	NEG	NEG	POS	NEG	POS	NEG	NEG	NEG
MSSA2052	NEG	POS	NEG	NEG	NEG	NEG	POS	NEG	NEG	NEG	NEG	NEG
MSSA2057	NEG	NEG	NEG	NEG	NEG	NEG	POS	NEG	AMB	NEG	NEG	NEG
MSSA2081	NEG	AMB	NEG	NEG	NEG	POS	POS	NEG	POS	NEG	NEG	NEG
MSSA1955	NEG	NEG	NEG	NEG	NEG	NEG	POS	NEG	AMB	NEG	POS	POS
MSSA1941	NEG	NEG	NEG	NEG	NEG	NEG	NEG	POS	NEG	NEG	NEG	NEG
MRSA3838	NEG	POS	NEG	NEG	NEG	NEG	POS	POS	NEG	NEG	NEG	NEG
MRSA3839	NEG	POS	NEG	NEG	NEG	NEG	POS	POS	NEG	NEG	NEG	NEG
MSSA1801	NEG	POS	NEG	NEG	NEG	NEG	POS	POS	NEG	NEG	NEG	NEG
MSSA2131	NEG	POS	NEG	NEG	NEG	NEG	POS	POS	AMB	NEG	NEG	NEG
MSSA1792	NEG	POS	NEG	NEG	NEG	NEG	NEG	NEG	POS	NEG	POS	POS
MSSA1796	NEG	POS	AMB	NEG	NEG	NEG	POS	NEG	NEG	POS	NEG	POS
MSSA1817	NEG	AMB	NEG	NEG	NEG	POS	POS	NEG	POS	NEG	POS	POS
MSSA1911	NEG	POS	NEG	NEG	NEG	NEG	NEG	NEG	NEG	NEG	NEG	NEG
MSSA1927	NEG	POS	AMB	NEG	NEG	NEG	NEG	NEG	POS	NEG	POS	POS
MSSA1928	NEG	AMB	POS	NEG	NEG	NEG	NEG	NEG	POS	NEG	POS	POS
MSSA1953	NEG	POS	AMB	NEG	NEG	NEG	NEG	NEG	POS	NEG	POS	POS
MSSA2062	NEG	POS	NEG	NEG	NEG	NEG	NEG	NEG	POS	NEG	POS	POS
MSSA2078	NEG	AMB	POS	NEG	NEG	NEG	POS	POS	POS	NEG	NEG	NEG
MSSA2136	NEG	AMB	POS	NEG	NEG	NEG	NEG	NEG	POS	NEG	POS	POS

	ADHAESION FACTORS / GENES ENCODING MICROBIAL SURFACE COMPONENTS RECOGNIZING ADHESIVE MATRIX MOLECULES (MSCRAMM GENES), continued														
Strain	sdrC (total)	sdrC-B1	sdrC (COL)	sdrC (Mu50)	sdrC (MW2+ MRSA252 + RF122)	sdrC (Other than MRSA252+RF122)	sdrD (COL+MW2)	sdrD (Mu50)	sdrD (other1)	sdrD (Other than MRSA252 +RF122)	vwb (total)	vwb (COL+MW2)	vwb (MRSA252)	vwb (Mu50)	vwb (RF122)
MSSA1805	POS	NEG	NEG	NEG	POS	POS	POS	NEG	NEG	POS	POS	POS	NEG	NEG	NEG
MSSA1823	POS	NEG	NEG	NEG	POS	POS	POS	NEG	NEG	POS	POS	POS	NEG	NEG	NEG
MSSA1837	POS	NEG	NEG	NEG	POS	POS	POS	NEG	NEG	POS	POS	POS	NEG	NEG	NEG
MSSA2044	POS	NEG	NEG	NEG	POS	POS	POS	NEG	NEG	POS	POS	POS	NEG	NEG	NEG
MSSA2046	POS	NEG	NEG	NEG	POS	POS	POS	NEG	NEG	POS	POS	POS	NEG	NEG	NEG
MSSA2059	POS	NEG	NEG	NEG	POS	POS	POS	NEG	NEG	POS	POS	POS	NEG	NEG	NEG
MSSA2076	POS	NEG	NEG	NEG	POS	POS	POS	NEG	NEG	POS	POS	POS	NEG	NEG	NEG
MSSA2200	POS	NEG	NEG	NEG	POS	POS	POS	NEG	NEG	POS	POS	POS	NEG	NEG	NEG
MRSA3797	POS	NEG	NEG	POS	NEG	POS	NEG	POS	NEG	POS	POS	NEG	NEG	POS	NEG
MRSA3798	POS	NEG	NEG	POS	NEG	POS	NEG	POS	NEG	POS	POS	NEG	NEG	POS	NEG
MRSA3799	POS	NEG	NEG	POS	NEG	POS	NEG	POS	NEG	POS	POS	NEG	NEG	POS	NEG
MRSA3800	POS	NEG	NEG	POS	NEG	POS	NEG	POS	NEG	POS	POS	NEG	NEG	POS	NEG
MRSA3801	POS	NEG	NEG	POS	NEG	POS	NEG	POS	NEG	POS	POS	NEG	NEG	POS	NEG
MRSA3804	POS	NEG	NEG	POS	NEG	POS	NEG	POS	NEG	POS	POS	NEG	NEG	POS	NEG
MRSA3806	POS	NEG	NEG	POS	NEG	POS	NEG	POS	NEG	POS	POS	NEG	NEG	POS	NEG
MRSA3961	POS	NEG	NEG	POS	NEG	POS	NEG	POS	NEG	POS	POS	NEG	NEG	POS	NEG
MRSA3962	POS	NEG	NEG	POS	NEG	POS	NEG	POS	NEG	POS	POS	NEG	NEG	POS	NEG
MRSA3963	POS	NEG	NEG	POS	NEG	POS	NEG	POS	NEG	POS	POS	NEG	NEG	POS	NEG
MRSA3966	POS	NEG	NEG	POS	NEG	POS	NEG	POS	NEG	POS	POS	NEG	NEG	POS	NEG
MRSA3978	POS	NEG	NEG	POS	NEG	POS	NEG	POS	NEG	POS	POS	NEG	NEG	POS	NEG
MRSA3979	POS	NEG	NEG	POS	NEG	POS	NEG	POS	NEG	POS	POS	NEG	NEG	POS	NEG
MRSA3980	POS	NEG	NEG	POS	NEG	POS	NEG	POS	NEG	POS	POS	NEG	NEG	POS	NEG
MRSA4179	POS	NEG	NEG	POS	NEG	POS	NEG	POS	NEG	POS	POS	NEG	NEG	POS	NEG
MRSA4216	POS	NEG	NEG	POS	NEG	POS	NEG	POS	NEG	POS	POS	NEG	NEG	POS	NEG
MRSA4217	POS	NEG	NEG	POS	NEG	POS	NEG	POS	NEG	POS	POS	NEG	NEG	POS	NEG
MRSA4220	POS	NEG	NEG	POS	NEG	POS	NEG	POS	NEG	POS	POS	NEG	NEG	POS	NEG
MRSA4221	POS	NEG	NEG	POS	NEG	POS	NEG	POS	NEG	POS	POS	NEG	NEG	POS	NEG
MRSA4222	POS	NEG	NEG	POS	NEG	POS	NEG	POS	NEG	POS	POS	NEG	NEG	POS	NEG
MSSA1751	POS	NEG	NEG	POS	NEG	POS	NEG	POS	NEG	POS	POS	NEG	NEG	POS	NEG
MSSA1760	POS	NEG	NEG	POS	AMB	POS	NEG	POS	NEG	POS	POS	NEG	NEG	POS	NEG
MSSA1802	POS	NEG	NEG	POS	NEG	POS	NEG	POS	NEG	POS	POS	NEG	NEG	POS	NEG
MSSA1809	POS	NEG	NEG	POS	NEG	POS	NEG	POS	NEG	POS	POS	NEG	NEG	POS	NEG
MSSA1815	POS	NEG	NEG	POS	NEG	POS	NEG	POS	NEG	POS	POS	NEG	NEG	POS	NEG
MSSA1829	POS	NEG	NEG	POS	NEG	POS	NEG	POS	NEG	POS	POS	NEG	NEG	POS	NEG
MSSA1908	POS	NEG	NEG	POS	NEG	POS	NEG	POS	NEG	POS	POS	NEG	NEG	POS	NEG
MSSA1960	POS	NEG	NEG	POS	NEG	POS	NEG	POS	NEG	POS	POS	NEG	NEG	POS	NEG
MSSA1965	POS	NEG	NEG	POS	NEG	POS	NEG	POS	NEG	POS	POS	NEG	NEG	POS	NEG
MSSA1977	POS	NEG	NEG	POS	NEG	POS	NEG	NEG	NEG	NEG	POS	NEG	NEG	POS	NEG
MSSA1978	POS	NEG	NEG	POS	NEG	POS	NEG	POS	NEG	POS	POS	NEG	NEG	POS	NEG
MSSA1999	POS	NEG	NEG	POS	NEG	POS	NEG	POS	NEG	POS	POS	NEG	NEG	POS	NEG
MSSA2003	POS	NEG	NEG	POS	NEG	POS	NEG	POS	NEG	POS	POS	NEG	NEG	POS	NEG
MSSA2006	POS	NEG	NEG	POS	NEG	POS	NEG	POS	NEG	POS	POS	NEG	NEG	POS	NEG
MSSA2008	POS	NEG	NEG	POS	NEG	POS	NEG	POS	NEG	POS	POS	NEG	NEG	POS	NEG
MSSA2011	POS	NEG	NEG	POS	NEG	POS	NEG	POS	NEG	POS	POS	NEG	NEG	POS	NEG
MSSA2017	POS	NEG	NEG	POS	NEG	POS	NEG	POS	NEG	POS	POS	NEG	NEG	POS	NEG
MSSA2022	POS	NEG	NEG	POS	NEG	POS	NEG	POS	NEG	POS	POS	NEG	NEG	POS	NEG
MSSA2055	POS	NEG	NEG	POS	NEG	POS	NEG	POS	NEG	POS	POS	NEG	NEG	POS	NEG
MSSA2060	POS	NEG	NEG	POS	NEG	POS	NEG	POS	NEG	POS	POS	NEG	NEG	POS	NEG
MSSA2080	POS	NEG	NEG	POS	NEG	POS	NEG	POS	NEG	POS	POS	NEG	NEG	POS	NEG
MSSA2082	POS	NEG	NEG	POS	AMB	POS	NEG	POS	NEG	POS	POS	NEG	NEG	POS	NEG
MSSA2084	POS	NEG	NEG	POS	NEG	POS	NEG	POS	NEG	POS	POS	NEG	NEG	POS	NEG
MSSA2087	POS	NEG	NEG	POS	NEG	POS	NEG	POS	NEG	POS	POS	NEG	NEG	POS	NEG
MSSA2094	POS	NEG	NEG	POS	NEG	POS	NEG	POS	NEG	POS	POS	NEG	NEG	POS	NEG
MSSA2097	POS	NEG	NEG	POS	NEG	POS	NEG	NEG	NEG	NEG	POS	NEG	NEG	POS	NEG
MSSA2110	POS	NEG	NEG	POS	NEG	POS	NEG	POS	NEG	POS	POS	NEG	NEG	POS	NEG
MSSA2113	POS	NEG	NEG	POS	NEG	POS	NEG	POS	NEG	POS	POS	NEG	NEG	POS	NEG
MSSA2132	POS	NEG	NEG	POS	NEG	POS	NEG	POS	NEG	POS	POS	NEG	NEG	POS	NEG
MSSA2134	POS	NEG	NEG	POS	NEG	POS	NEG	POS	NEG	POS	POS	NEG	NEG	POS	NEG
MSSA1746	POS	NEG	POS	NEG	NEG	POS	NEG	POS	NEG	POS	POS	NEG	NEG	NEG	NEG
MSSA1757	POS	NEG	POS	NEG	NEG	POS	NEG	POS	NEG	POS	POS	NEG	NEG	NEG	NEG
MSSA1772	POS	NEG	POS	NEG	NEG	POS	NEG	POS	NEG	POS	POS	NEG	NEG	NEG	NEG
MSSA1795	POS	NEG	POS	NEG	NEG	POS	NEG	POS	NEG	POS	POS	NEG	NEG	AMB	NEG
MSSA1813	POS	NEG	POS	NEG	NEG	POS	NEG	POS	NEG	POS	POS	NEG	NEG	NEG	NEG
MSSA1826	POS	NEG	POS	NEG	NEG	POS	NEG	POS	NEG	POS	POS	NEG	NEG	NEG	NEG
MSSA1830	POS	NEG	POS	NEG	NEG	POS	NEG	POS	NEG	POS	POS	NEG	NEG	AMB	NEG
MSSA1838	POS	NEG	POS	NEG	NEG	POS	NEG	POS	NEG	POS	POS	NEG	NEG	NEG	NEG
MSSA1944	POS	NEG	POS	NEG	NEG	POS	NEG	POS	NEG	POS	POS	NEG	NEG	NEG	NEG
MSSA1947	POS	NEG	POS	NEG	NEG	POS	NEG	POS	NEG	POS	POS	NEG	NEG	NEG	NEG
MSSA1964	POS	NEG	POS	NEG	NEG	POS	NEG	POS	NEG	POS	POS	NEG	NEG	NEG	NEG
MSSA1969	POS	NEG	POS	NEG	NEG	POS	NEG	POS	NEG	POS	POS	NEG	NEG	AMB	NEG
MSSA2001	POS	NEG	POS	NEG	NEG	POS	NEG	POS	NEG	POS	POS	NEG	NEG	NEG	NEG
MSSA2004	POS	NEG	POS	NEG	NEG	POS	NEG	POS	NEG	POS	POS	NEG	NEG	NEG	NEG
MSSA2024	POS	NEG	POS	NEG	NEG	POS	NEG	POS	NEG	POS	POS	NEG	NEG	NEG	NEG
MSSA2025	POS	NEG	POS	NEG	NEG	POS	NEG	POS	NEG	POS	POS	NEG	NEG	NEG	NEG
MSSA2026	POS	NEG	POS	NEG	NEG	POS	NEG	POS	NEG	POS	POS	NEG	NEG	NEG	NEG
MSSA2032	POS	NEG	POS	NEG	NEG	POS	NEG	POS	NEG	POS	POS	NEG	NEG	NEG	NEG
MSSA2043	POS	NEG	POS	NEG	NEG	POS	NEG	POS	NEG	POS	POS	NEG	NEG	NEG	NEG
MSSA2061	POS	NEG	POS	NEG	NEG	POS	NEG	POS	NEG	POS	POS	NEG	NEG	NEG	NEG
MSSA2085	POS	NEG	POS	NEG	NEG	POS	NEG	POS	NEG	POS	POS	NEG	NEG	NEG	NEG
MSSA2098	POS	NEG	POS	NEG	NEG	POS	NEG	POS	NEG	POS	POS	NEG	NEG	NEG	NEG
MSSA2100	POS	NEG	POS	NEG	NEG	POS	NEG	POS	NEG	POS	POS	NEG	NEG	NEG	NEG
MSSA2111	POS	NEG	POS	NEG	NEG	POS	NEG	POS	NEG	POS	POS	NEG	NEG	AMB	NEG
MSSA2178	POS	NEG	POS	NEG	NEG	POS	NEG	POS	NEG	POS	POS	NEG	NEG	NEG	NEG
MSSA2202	POS	NEG	POS	NEG	NEG	POS	NEG	POS	NEG	POS	POS	NEG	NEG	NEG	NEG
MSSA2205	POS	NEG	POS	NEG	NEG	POS	NEG	POS	NEG	POS	POS	NEG	NEG	NEG	NEG
MRSA3796	POS	NEG	POS	NEG	NEG	POS	NEG	NEG	NEG	NEG	POS	POS	NEG	NEG	NEG
MRSA3805	POS	NEG	POS	NEG	NEG	POS	POS	NEG	NEG	POS	POS	POS	NEG	NEG	NEG
MRSA3965	POS	NEG	POS	NEG	NEG	POS	POS	NEG	NEG	POS	POS	POS	NEG	NEG	NEG
MSSA1761	POS	NEG	POS	AMB	AMB	POS	NEG	NEG	NEG	NEG	POS	POS	NEG	NEG	NEG
MSSA1764	POS	NEG	POS	NEG	NEG	POS	POS	NEG	NEG	POS	POS	POS	NEG	NEG	NEG
MSSA1765	POS	NEG	POS	NEG	NEG	POS	POS	NEG	NEG	POS	POS	POS	NEG	NEG	NEG
MSSA1784	POS	NEG	POS	NEG	NEG	POS	NEG	NEG	NEG	NEG	POS	POS	NEG	NEG	NEG
MSSA1806	POS	NEG	POS	NEG	NEG	POS	POS	NEG	NEG	POS	POS	POS	NEG	NEG	NEG
MSSA1807	POS	NEG	POS	NEG	NEG	POS	POS	NEG	NEG	POS	POS	POS	NEG	NEG	NEG
MSSA1888	POS	NEG	POS	NEG	NEG	POS	POS	NEG	NEG	POS	POS	POS	NEG	NEG	NEG
MSSA1894	POS	NEG	POS	NEG	NEG	POS	POS	NEG	NEG	POS	POS	POS	NEG	NEG	NEG
MSSA1922	POS	NEG	POS	NEG	NEG	POS	POS	NEG	NEG	POS	POS	POS	NEG	NEG	NEG
MSSA1950	POS	NEG	POS	NEG	NEG	POS	POS	NEG	NEG	POS	POS	POS	NEG	NEG	NEG
MSSA1954	POS	NEG	POS	NEG	NEG	POS	POS	NEG	NEG	POS	POS	POS	NEG	NEG	NEG
MSSA1966	POS	NEG	POS	NEG	NEG	POS	POS	NEG	NEG	POS	POS	POS	NEG	NEG	NEG
MSSA1972	POS	NEG	POS	NEG	NEG	POS	POS	NEG	NEG	POS	POS	POS	NEG	NEG	NEG
MSSA2010	POS	NEG	POS	NEG	NEG	POS	POS	NEG	NEG	POS	POS	POS	NEG	NEG	NEG
MSSA2020	POS	NEG	POS	NEG	NEG	POS	POS	NEG	NEG	POS	POS	POS	NEG	NEG	NEG
MSSA2028	POS	NEG	POS	NEG	NEG	POS	POS	NEG	NEG	POS	POS	POS	NEG	NEG	NEG
MSSA2088	POS	NEG	POS	NEG	NEG	POS	POS	NEG	NEG	POS	POS	POS	NEG	NEG	NEG
MSSA2187	POS	NEG	POS	NEG	NEG	POS	NEG	NEG	NEG	NEG	POS	POS	NEG	NEG	NEG
MSSA2204	POS	NEG	POS	AMB	NEG	POS	POS	NEG	NEG	POS	POS	POS	NEG	NEG	NEG
MSSA2210	POS	NEG	POS	NEG	NEG	POS	POS	NEG	NEG	POS	POS	POS	NEG	NEG	NEG
MSSA2223	POS	NEG	POS	NEG	NEG	POS	POS	NEG	NEG	POS	POS	POS	NEG	NEG	NEG
MSSA1763	POS	NEG	NEG	POS	AMB	POS	NEG	NEG	POS	POS	POS	NEG	POS	NEG	NEG
MSSA1777	POS	NEG	NEG	POS	POS	POS	NEG	NEG	NEG	NEG	POS	NEG	POS	NEG	NEG
MSSA2009	POS	NEG	NEG	POS	POS	POS	NEG	NEG	POS	POS	POS	NEG	POS	NEG	NEG
MSSA2047	POS	NEG	NEG	POS	POS	POS	NEG	NEG	NEG	NEG	POS	NEG	POS	NEG	NEG
MSSA2079	POS	NEG	NEG	POS	AMB	POS	NEG	NEG	NEG	NEG	POS	NEG	POS	NEG	NEG
MSSA2101	POS	NEG	NEG	POS	POS	POS	NEG	NEG	NEG	NEG	POS	NEG	POS	NEG	NEG
MSSA1821	POS	NEG	POS	NEG	NEG	POS	NEG	NEG	NEG	NEG	POS	NEG	NEG	NEG	NEG
MSSA1898	POS	NEG	POS	NEG	NEG	POS	NEG	NEG	NEG	NEG	POS	NEG	NEG	NEG	NEG
MSSA1973	POS	NEG	POS	NEG	NEG	POS	NEG	NEG	NEG	NEG	POS	NEG	NEG	NEG	NEG
MSSA2071	POS	NEG	POS	NEG	NEG	POS	NEG	NEG	NEG	NEG	POS	NEG	NEG	NEG	NEG
MSSA2083	POS	NEG	POS	NEG	NEG	POS	NEG	NEG	NEG	NEG	POS	NEG	NEG	NEG	NEG
MSSA2106	POS	NEG	POS	NEG	NEG	POS	NEG	NEG	NEG	NEG	POS	NEG	NEG	NEG	NEG
MSSA2112	POS	NEG	POS	NEG	NEG	POS	POS	NEG	NEG	POS	POS	NEG	NEG	NEG	NEG
MSSA2115	POS	NEG	POS	NEG	NEG	POS	POS	NEG	NEG	POS	POS	NEG	NEG	NEG	NEG
MSSA2195	POS	NEG	POS	NEG	NEG	POS	NEG	NEG	NEG	NEG	POS	NEG	NEG	NEG	NEG
MSSA2207	POS	NEG	POS	NEG	NEG	POS	NEG	NEG	NEG	NEG	POS	NEG	NEG	NEG	NEG
MSSA1758	POS	NEG	POS	NEG	NEG	POS	NEG	POS	NEG	POS	POS	NEG	NEG	NEG	NEG
MSSA1762	POS	NEG	POS	NEG	NEG	POS	NEG	POS	NEG	POS	POS	NEG	NEG	NEG	NEG
MSSA1769	POS	NEG	POS	NEG	NEG	POS	NEG	POS	NEG	POS	POS	NEG	NEG	NEG	NEG
MSSA1771	POS	NEG	POS	NEG	NEG	POS	NEG	POS	NEG	POS	POS	NEG	NEG	NEG	NEG
MSSA1789	POS	NEG	POS	NEG	NEG	POS	NEG	POS	NEG	POS	POS	NEG	NEG	NEG	NEG
MSSA1800	POS	NEG	POS	NEG	NEG	POS	NEG	POS	NEG	POS	POS	NEG	NEG	NEG	NEG
MSSA1833	POS	NEG	POS	NEG	NEG	POS	NEG	POS	NEG	POS	POS	NEG	NEG	NEG	NEG
MSSA1890	POS	NEG	POS	NEG	NEG	POS	NEG	POS	NEG	POS	POS	NEG	NEG	NEG	NEG
MSSA1903	POS	NEG	POS	NEG	NEG	POS	NEG	POS	NEG	POS	POS	NEG	NEG	NEG	NEG
MSSA1940	POS	NEG	POS	NEG	NEG	POS	NEG	POS	AMB	POS	POS	NEG	NEG	NEG	NEG
MSSA1962	POS	NEG	POS	NEG	NEG	POS	NEG	POS	NEG	POS	POS	NEG	NEG	NEG	NEG
MSSA1963	POS	NEG	POS	NEG	NEG	POS	NEG	POS	NEG	POS	POS	NEG	NEG	NEG	NEG
MSSA2016	POS	NEG	POS	NEG	NEG	POS	NEG	POS	NEG	POS	POS	NEG	NEG	NEG	NEG
MSSA2018	POS	NEG	POS	NEG	NEG	POS	NEG	POS	NEG	POS	POS	NEG	NEG	NEG	NEG
MSSA2019	POS	NEG	POS	NEG	NEG	POS	NEG	POS	NEG	POS	POS	NEG	NEG	POS	NEG
MSSA2029	POS	NEG	POS	NEG	NEG	POS	NEG	POS	NEG	POS	POS	NEG	NEG	NEG	NEG
MSSA2045	POS	NEG	POS	NEG	NEG	POS	NEG	POS	NEG	POS	POS	NEG	NEG	NEG	NEG
MSSA2048	POS	NEG	POS	NEG	NEG	POS	NEG	POS	NEG	POS	POS	NEG	NEG	NEG	NEG
MSSA2049	POS	NEG	POS	NEG	NEG	POS	NEG	POS	NEG	POS	POS	NEG	NEG	POS	NEG
MSSA2050	POS	NEG	POS	NEG	NEG	POS	NEG	POS	NEG	POS	POS	NEG	NEG	POS	NEG
MSSA2099	POS	NEG	POS	NEG	NEG	POS	NEG	POS	NEG	POS	POS	NEG	NEG	NEG	NEG
MSSA2109	POS	NEG	POS	NEG	NEG	POS	NEG	POS	NEG	POS	POS	NEG	NEG	NEG	NEG
MSSA2130	POS	NEG	POS	NEG	NEG	POS	NEG	POS	NEG	POS	POS	NEG	NEG	NEG	NEG
MSSA2133	POS	NEG	POS	NEG	NEG	POS	NEG	POS	NEG	POS	POS	NEG	NEG	NEG	NEG
MSSA2139	POS	NEG	POS	NEG	NEG	POS	NEG	NEG	NEG	NEG	POS	NEG	NEG	NEG	NEG
MSSA2141	POS	NEG	POS	NEG	NEG	POS	NEG	NEG	NEG	NEG	POS	NEG	NEG	NEG	NEG
MSSA2208	POS	NEG	POS	NEG	NEG	POS	NEG	POS	NEG	POS	POS	NEG	NEG	POS	NEG
MSSA2209	POS	NEG	POS	NEG	NEG	POS	NEG	POS	NEG	NEG	POS	NEG	NEG	NEG	NEG
MSSA1893	POS	NEG	NEG	POS	AMB	POS	NEG	NEG	POS	POS	POS	NEG	NEG	POS	NEG
MSSA2105	POS	NEG	NEG	POS	POS	POS	NEG	NEG	POS	POS	POS	NEG	NEG	POS	NEG
MRSA3836	POS	NEG	NEG	POS	NEG	POS	NEG	NEG	POS	POS	POS	NEG	NEG	NEG	POS
MRSA3840	POS	NEG	NEG	POS	NEG	POS	NEG	NEG	POS	POS	POS	NEG	NEG	NEG	POS
MRSA4188	POS	NEG	NEG	POS	AMB	POS	NEG	NEG	POS	POS	POS	NEG	NEG	NEG	POS
MSSA1814	POS	NEG	NEG	POS	NEG	POS	NEG	NEG	NEG	NEG	POS	NEG	NEG	NEG	POS
MSSA1816	POS	NEG	NEG	POS	NEG	POS	NEG	NEG	POS	POS	POS	NEG	NEG	NEG	POS
MSSA1825	POS	NEG	NEG	POS	NEG	POS	NEG	NEG	POS	POS	POS	NEG	NEG	NEG	POS
MSSA1834	POS	NEG	NEG	POS	NEG	POS	NEG	NEG	POS	POS	POS	NEG	NEG	NEG	POS
MSSA1938	POS	NEG	NEG	POS	NEG	POS	NEG	NEG	POS	POS	POS	NEG	NEG	NEG	POS
MSSA1948	POS	NEG	NEG	POS	NEG	POS	NEG	NEG	POS	POS	POS	NEG	NEG	NEG	POS
MSSA1951	POS	NEG	NEG	POS	NEG	POS	NEG	NEG	POS	POS	POS	NEG	NEG	NEG	POS
MSSA2005	POS	NEG	NEG	POS	NEG	POS	NEG	NEG	POS	POS	POS	NEG	NEG	NEG	POS
MSSA2051	POS	NEG	NEG	POS	NEG	POS	NEG	NEG	POS	POS	POS	NEG	NEG	NEG	POS
MSSA2056	POS	NEG	NEG	POS	NEG	POS	NEG	NEG	POS	POS	POS	NEG	NEG	NEG	POS
MSSA2066	POS	NEG	NEG	POS	NEG	POS	NEG	NEG	POS	POS	POS	NEG	NEG	NEG	POS
MSSA2067	POS	NEG	NEG	POS	NEG	POS	NEG	NEG	NEG	NEG	POS	NEG	NEG	NEG	POS
MSSA2086	POS	NEG	NEG	POS	NEG	POS	NEG	NEG	POS	POS	POS	NEG	NEG	NEG	POS
MSSA2089	POS	NEG	NEG	POS	NEG	POS	NEG	NEG	POS	POS	POS	NEG	NEG	NEG	POS
MSSA2103	POS	NEG	NEG	POS	NEG	POS	NEG	NEG	POS	POS	POS	NEG	NEG	NEG	POS
MSSA2173	POS	NEG	NEG	POS	NEG	POS	NEG	NEG	POS	POS	POS	NEG	NEG	NEG	POS
MSSA2183	POS	NEG	NEG	POS	NEG	POS	NEG	NEG	NEG	NEG	POS	NEG	NEG	NEG	POS
MSSA2196	POS	NEG	NEG	POS	NEG	POS	NEG	NEG	POS	POS	POS	NEG	NEG	NEG	POS
MSSA2219	POS	NEG	NEG	POS	NEG	POS	NEG	NEG	POS	POS	POS	NEG	NEG	NEG	POS
MSSA1753	POS	NEG	NEG	NEG	POS	POS	POS	NEG	NEG	POS	POS	NEG	NEG	NEG	NEG
MSSA1773	POS	NEG	NEG	NEG	POS	POS	POS	NEG	NEG	POS	POS	NEG	NEG	NEG	NEG
MSSA1811	POS	NEG	NEG	NEG	POS	POS	POS	NEG	NEG	POS	POS	NEG	NEG	NEG	NEG
MSSA1897	POS	NEG	NEG	NEG	POS	POS	POS	NEG	NEG	POS	POS	NEG	NEG	NEG	NEG
MSSA1931	POS	NEG	NEG	NEG	POS	POS	POS	NEG	NEG	POS	POS	NEG	NEG	NEG	NEG
MSSA2114	POS	NEG	NEG	NEG	POS	POS	POS	NEG	NEG	POS	POS	NEG	NEG	NEG	NEG
MSSA1745	POS	NEG	NEG	NEG	NEG	NEG	NEG	NEG	POS	POS	POS	NEG	POS	NEG	NEG
MSSA1747	POS	NEG	NEG	NEG	NEG	NEG	NEG	NEG	POS	POS	POS	NEG	POS	NEG	NEG
MSSA1749	POS	NEG	NEG	NEG	NEG	NEG	NEG	NEG	NEG	NEG	POS	NEG	POS	NEG	NEG
MSSA1768	POS	NEG	NEG	NEG	NEG	NEG	NEG	NEG	POS	POS	POS	NEG	POS	NEG	NEG
MSSA1774	POS	NEG	NEG	NEG	NEG	NEG	NEG	NEG	POS	POS	POS	NEG	POS	NEG	NEG
MSSA1775	POS	NEG	NEG	NEG	NEG	NEG	NEG	NEG	POS	POS	POS	NEG	POS	NEG	NEG
MSSA1776	POS	NEG	NEG	NEG	AMB	NEG	NEG	NEG	NEG	NEG	POS	NEG	POS	NEG	NEG
MSSA1779	POS	NEG	NEG	NEG	NEG	NEG	NEG	NEG	POS	POS	POS	NEG	POS	NEG	NEG
MSSA1780	POS	NEG	NEG	NEG	NEG	NEG	NEG	NEG	POS	POS	POS	NEG	POS	NEG	NEG

Strain	sdrC (total)	sdrC-B1	sdrC (COL)	sdrC (Mu50)	sdrC (MW2+ MRSA252 + RF122)	sdrC (Other than MRSA252+RF122)	sdrD (COL+MW2)	sdrD (Mu50)	sdrD (other1)	sdrD (Other than MRSA252 +RF122)	vwb (total)	vwb (COL+MW2)	vwb (MRSA252)	vwb (Mu50)	vwb (RF122)
	ADHAESION FACTORS / GENES ENCODING MICROBIAL SURFACE COMPONENTS RECOGNIZING ADHESIVE MATRIX MOLECULES (MSCRAMM GENES), continued														
MSSA1781	POS	NEG	NEG	NEG	NEG	NEG	NEG	NEG	POS	POS	POS	NEG	POS	NEG	NEG
MSSA1786	POS	NEG	NEG	NEG	NEG	NEG	NEG	NEG	NEG	NEG	POS	NEG	POS	NEG	NEG
MSSA1793	POS	NEG	NEG	NEG	NEG	NEG	NEG	NEG	POS	POS	POS	NEG	POS	NEG	NEG
MSSA1803	POS	NEG	NEG	NEG	NEG	NEG	NEG	NEG	POS	POS	POS	NEG	POS	NEG	NEG
MSSA1836	POS	NEG	NEG	NEG	NEG	NEG	NEG	NEG	POS	POS	POS	NEG	POS	NEG	NEG
MSSA1839	POS	NEG	NEG	NEG	NEG	NEG	NEG	NEG	POS	POS	POS	NEG	POS	NEG	NEG
MSSA1900	POS	NEG	NEG	NEG	NEG	NEG	NEG	NEG	POS	POS	POS	NEG	POS	NEG	NEG
MSSA1910	POS	NEG	NEG	NEG	NEG	NEG	NEG	NEG	POS	POS	POS	NEG	POS	NEG	NEG
MSSA1918	POS	NEG	NEG	NEG	NEG	NEG	NEG	NEG	POS	POS	POS	NEG	POS	NEG	NEG
MSSA1921	POS	NEG	NEG	NEG	NEG	NEG	NEG	NEG	NEG	NEG	POS	NEG	POS	NEG	NEG
MSSA1923	POS	NEG	NEG	NEG	NEG	NEG	NEG	NEG	POS	POS	POS	NEG	POS	NEG	NEG
MSSA1981	POS	NEG	NEG	NEG	NEG	NEG	NEG	NEG	POS	POS	POS	NEG	POS	NEG	NEG
MSSA1998	POS	NEG	NEG	NEG	NEG	NEG	NEG	NEG	POS	POS	POS	NEG	POS	NEG	NEG
MSSA2000	POS	NEG	NEG	NEG	NEG	NEG	NEG	NEG	POS	POS	POS	NEG	POS	NEG	NEG
MSSA2007	POS	NEG	NEG	NEG	AMB	NEG	NEG	NEG	POS	POS	POS	NEG	POS	NEG	NEG
MSSA2012	POS	NEG	NEG	NEG	NEG	NEG	NEG	NEG	POS	POS	POS	NEG	POS	NEG	NEG
MSSA2015	POS	NEG	NEG	NEG	NEG	NEG	NEG	NEG	POS	POS	POS	NEG	POS	NEG	NEG
MSSA2021	POS	NEG	NEG	NEG	NEG	NEG	NEG	NEG	POS	POS	POS	NEG	POS	NEG	NEG
MSSA2027	POS	NEG	NEG	NEG	NEG	NEG	NEG	NEG	POS	POS	POS	NEG	POS	NEG	NEG
MSSA2063	POS	NEG	NEG	NEG	NEG	NEG	NEG	NEG	POS	POS	POS	NEG	POS	NEG	NEG
MSSA2064	POS	NEG	NEG	NEG	NEG	NEG	NEG	NEG	POS	POS	POS	NEG	POS	NEG	NEG
MSSA2065	POS	NEG	NEG	NEG	NEG	NEG	NEG	NEG	POS	POS	POS	NEG	POS	NEG	NEG
MSSA2075	POS	NEG	NEG	NEG	NEG	NEG	NEG	NEG	POS	POS	POS	NEG	POS	NEG	NEG
MSSA2093	POS	NEG	NEG	NEG	NEG	NEG	NEG	NEG	NEG	NEG	POS	NEG	POS	NEG	NEG
MSSA2102	POS	NEG	NEG	NEG	NEG	NEG	NEG	NEG	POS	POS	POS	NEG	POS	NEG	NEG
MSSA2138	POS	NEG	NEG	NEG	NEG	NEG	NEG	NEG	POS	POS	POS	NEG	POS	NEG	NEG
MSSA2168	POS	NEG	NEG	NEG	NEG	NEG	NEG	NEG	POS	POS	POS	NEG	POS	NEG	NEG
MSSA2181	POS	NEG	NEG	NEG	NEG	NEG	NEG	NEG	NEG	NEG	POS	NEG	POS	NEG	NEG
MSSA2189	POS	NEG	NEG	NEG	NEG	NEG	NEG	NEG	POS	POS	POS	NEG	POS	NEG	NEG
MSSA2214	POS	NEG	NEG	NEG	NEG	NEG	NEG	NEG	POS	POS	POS	NEG	POS	NEG	NEG
MRSA3803	POS	POS	NEG	NEG	NEG	NEG	POS	NEG	NEG	POS	POS	NEG	POS	NEG	NEG
MSSA1750	POS	POS	NEG	AMB	AMB	NEG	POS	NEG	NEG	POS	POS	NEG	POS	NEG	NEG
MSSA1756	POS	POS	NEG	NEG	AMB	NEG	POS	NEG	NEG	POS	POS	NEG	POS	NEG	NEG
MSSA1759	POS	POS	NEG	NEG	NEG	NEG	POS	NEG	NEG	POS	NEG	NEG	POS	NEG	NEG
MSSA1767	POS	POS	NEG	NEG	NEG	NEG	NEG	NEG	NEG	NEG	AMB	NEG	POS	NEG	NEG
MSSA1787	POS	POS	NEG	NEG	NEG	NEG	POS	NEG	NEG	POS	AMB	NEG	POS	NEG	NEG
MSSA1790	POS	POS	NEG	NEG	NEG	NEG	POS	NEG	NEG	POS	AMB	NEG	POS	NEG	NEG
MSSA1794	POS	POS	NEG	NEG	NEG	NEG	POS	NEG	NEG	POS	NEG	NEG	POS	NEG	NEG
MSSA1808	POS	POS	NEG	NEG	NEG	NEG	POS	NEG	NEG	POS	NEG	NEG	POS	NEG	NEG
MSSA1812	POS	POS	NEG	NEG	NEG	NEG	POS	NEG	NEG	POS	NEG	NEG	POS	NEG	NEG
MSSA1819	POS	POS	NEG	NEG	NEG	NEG	POS	NEG	NEG	POS	NEG	NEG	POS	NEG	NEG
MSSA1824	POS	POS	NEG	NEG	NEG	NEG	POS	NEG	NEG	POS	NEG	NEG	POS	NEG	NEG
MSSA1827	POS	POS	NEG	NEG	NEG	NEG	NEG	NEG	NEG	NEG	NEG	NEG	POS	NEG	NEG
MSSA1889	POS	POS	NEG	NEG	NEG	NEG	POS	NEG	NEG	POS	NEG	NEG	POS	NEG	NEG
MSSA1895	POS	POS	NEG	NEG	NEG	NEG	POS	NEG	NEG	POS	NEG	NEG	POS	NEG	NEG
MSSA1904	POS	POS	NEG	NEG	NEG	NEG	POS	NEG	NEG	POS	NEG	NEG	POS	NEG	NEG
MSSA1905	POS	POS	NEG	NEG	NEG	NEG	POS	NEG	NEG	POS	NEG	NEG	POS	NEG	NEG
MSSA1907	POS	POS	NEG	NEG	NEG	NEG	POS	NEG	NEG	POS	NEG	NEG	POS	NEG	NEG
MSSA1919	POS	POS	NEG	NEG	NEG	NEG	POS	NEG	NEG	POS	NEG	NEG	POS	NEG	NEG
MSSA1920	POS	POS	NEG	NEG	NEG	NEG	POS	NEG	NEG	POS	NEG	NEG	POS	NEG	NEG
MSSA1929	POS	POS	NEG	NEG	NEG	NEG	POS	NEG	NEG	POS	NEG	NEG	POS	NEG	NEG
MSSA1930	POS	POS	NEG	NEG	NEG	NEG	POS	NEG	NEG	POS	AMB	NEG	POS	NEG	NEG
MSSA1934	POS	POS	NEG	NEG	NEG	NEG	POS	NEG	NEG	POS	NEG	NEG	POS	NEG	NEG
MSSA1946	POS	POS	NEG	NEG	NEG	NEG	POS	NEG	NEG	POS	NEG	NEG	POS	NEG	NEG
MSSA1971	POS	POS	AMB	NEG	NEG	NEG	POS	NEG	NEG	POS	AMB	NEG	POS	NEG	NEG
MSSA1980	POS	POS	NEG	NEG	NEG	NEG	POS	NEG	NEG	POS	NEG	NEG	POS	NEG	NEG
MSSA1982	POS	POS	NEG	NEG	NEG	NEG	NEG	NEG	NEG	NEG	NEG	NEG	POS	NEG	NEG
MSSA1996	POS	POS	NEG	NEG	NEG	NEG	POS	NEG	NEG	POS	NEG	NEG	POS	NEG	NEG
MSSA2002	POS	POS	NEG	NEG	NEG	NEG	POS	NEG	NEG	POS	NEG	NEG	POS	NEG	NEG
MSSA2014	POS	POS	NEG	NEG	NEG	NEG	POS	NEG	NEG	POS	NEG	NEG	POS	NEG	NEG
MSSA2053	POS	POS	NEG	NEG	NEG	NEG	NEG	NEG	NEG	NEG	NEG	NEG	POS	NEG	NEG
MSSA2054	POS	POS	NEG	NEG	NEG	NEG	POS	NEG	NEG	POS	NEG	NEG	POS	NEG	NEG
MSSA2058	POS	POS	NEG	NEG	NEG	NEG	POS	NEG	NEG	POS	NEG	NEG	POS	NEG	NEG
MSSA2068	POS	POS	NEG	NEG	NEG	NEG	POS	NEG	NEG	POS	NEG	NEG	POS	NEG	NEG
MSSA2070	POS	POS	NEG	NEG	NEG	NEG	POS	NEG	NEG	POS	NEG	NEG	POS	NEG	NEG
MSSA2072	POS	POS	NEG	NEG	NEG	NEG	POS	NEG	NEG	POS	NEG	NEG	POS	NEG	NEG
MSSA2073	POS	POS	NEG	NEG	NEG	NEG	POS	NEG	NEG	POS	NEG	NEG	POS	NEG	NEG
MSSA2074	POS	POS	NEG	NEG	NEG	NEG	NEG	NEG	NEG	NEG	AMB	NEG	POS	NEG	NEG
MSSA2107	POS	POS	NEG	NEG	NEG	NEG	POS	NEG	NEG	POS	AMB	NEG	POS	NEG	NEG
MSSA2108	POS	POS	NEG	NEG	NEG	NEG	POS	NEG	NEG	POS	NEG	NEG	POS	NEG	NEG
MSSA2129	POS	POS	NEG	NEG	NEG	NEG	POS	NEG	NEG	POS	NEG	NEG	POS	NEG	NEG
MSSA2135	POS	POS	NEG	NEG	NEG	NEG	NEG	NEG	NEG	NEG	AMB	NEG	POS	NEG	NEG
MSSA2137	POS	POS	NEG	NEG	NEG	NEG	NEG	NEG	NEG	NEG	POS	NEG	NEG	NEG	NEG
MSSA2140	POS	POS	NEG	NEG	NEG	NEG	POS	NEG	NEG	POS	NEG	NEG	POS	NEG	NEG
MSSA2182	POS	POS	NEG	NEG	NEG	NEG	POS	NEG	NEG	POS	AMB	NEG	POS	NEG	NEG
MSSA2188	POS	POS	NEG	NEG	NEG	NEG	POS	NEG	NEG	POS	NEG	NEG	POS	NEG	NEG
MSSA1899	POS	NEG	NEG	NEG	POS	NEG	NEG	NEG	NEG	POS	POS	NEG	NEG	NEG	NEG
MSSA1915	POS	NEG	NEG	NEG	POS	NEG	NEG	NEG	NEG	POS	POS	NEG	NEG	NEG	NEG
MSSA1949	POS	NEG	NEG	NEG	POS	NEG	NEG	NEG	NEG	POS	POS	NEG	NEG	NEG	NEG
MSSA2042	POS	NEG	NEG	NEG	POS	NEG	NEG	NEG	NEG	POS	POS	NEG	NEG	NEG	NEG
MSSA2077	POS	NEG	NEG	NEG	POS	NEG	NEG	NEG	NEG	POS	POS	NEG	NEG	NEG	NEG
MSSA2095	POS	NEG	NEG	NEG	POS	NEG	NEG	NEG	NEG	POS	POS	NEG	NEG	NEG	NEG
MSSA1997	POS	NEG	NEG	POS	POS	POS	NEG	NEG	NEG	NEG	POS	NEG	NEG	POS	NEG
MSSA1810	POS	NEG	POS	NEG	NEG	POS	POS	NEG	NEG	NEG	POS	POS	NEG	NEG	NEG
MSSA1832	POS	NEG	NEG	POS	POS	POS	NEG	POS	NEG	POS	POS	NEG	NEG	NEG	POS
MSSA1891	POS	NEG	NEG	POS	POS	POS	NEG	NEG	NEG	NEG	POS	NEG	NEG	NEG	POS
MSSA1892	POS	NEG	NEG	POS	POS	POS	NEG	POS	NEG	POS	POS	NEG	NEG	NEG	POS
MSSA1932	POS	NEG	NEG	POS	POS	POS	NEG	POS	NEG	POS	POS	NEG	NEG	NEG	POS
MSSA1942	POS	NEG	NEG	POS	POS	POS	NEG	POS	NEG	POS	POS	NEG	NEG	NEG	POS
MSSA1957	POS	NEG	NEG	POS	POS	POS	NEG	POS	NEG	POS	POS	NEG	NEG	NEG	POS
MSSA1748	POS	NEG	NEG	POS	NEG	POS	POS	NEG	NEG	POS	POS	NEG	NEG	NEG	NEG
MSSA1754	POS	NEG	NEG	POS	NEG	POS	POS	NEG	NEG	POS	POS	NEG	NEG	NEG	NEG
MSSA1770	POS	NEG	NEG	POS	NEG	POS	POS	NEG	NEG	POS	POS	NEG	NEG	NEG	NEG
MSSA1788	POS	NEG	NEG	POS	NEG	POS	POS	NEG	NEG	POS	POS	NEG	NEG	NEG	NEG
MSSA1804	POS	NEG	NEG	POS	NEG	POS	POS	NEG	NEG	POS	POS	NEG	NEG	NEG	NEG
MSSA1818	POS	NEG	NEG	POS	NEG	POS	POS	NEG	NEG	POS	POS	NEG	NEG	NEG	NEG
MSSA1914	POS	NEG	NEG	POS	NEG	POS	POS	NEG	NEG	POS	POS	NEG	NEG	NEG	NEG
MSSA1974	POS	NEG	NEG	POS	NEG	POS	POS	NEG	NEG	POS	POS	NEG	NEG	NEG	NEG
MSSA2023	POS	NEG	NEG	POS	NEG	POS	POS	NEG	NEG	POS	POS	NEG	NEG	NEG	NEG
MSSA2030	POS	NEG	NEG	POS	NEG	POS	POS	NEG	NEG	POS	POS	NEG	NEG	NEG	NEG
MSSA2096	POS	NEG	NEG	POS	NEG	POS	AMB	NEG	NEG	POS	POS	NEG	NEG	NEG	NEG
MSSA2142	POS	NEG	NEG	POS	NEG	POS	POS	NEG	NEG	POS	POS	NEG	NEG	NEG	NEG
MSSA2203	POS	NEG	NEG	POS	NEG	POS	POS	NEG	NEG	POS	POS	NEG	NEG	NEG	NEG
MSSA1901	POS	NEG	POS	NEG	NEG	NEG	NEG	NEG	NEG	POS	POS	POS	NEG	NEG	NEG
MSSA2013	POS	NEG	POS	NEG	NEG	NEG	NEG	NEG	NEG	POS	POS	POS	NEG	NEG	NEG
MSSA2031	POS	NEG	POS	NEG	NEG	NEG	NEG	NEG	NEG	POS	POS	POS	NEG	NEG	NEG
MSSA2052	POS	NEG	POS	NEG	NEG	NEG	NEG	NEG	NEG	POS	POS	POS	NEG	NEG	NEG
MSSA2057	POS	NEG	POS	NEG	NEG	NEG	NEG	NEG	NEG	POS	POS	POS	NEG	NEG	NEG
MSSA2081	POS	NEG	NEG	NEG	POS	NEG	POS	NEG	NEG	POS	POS	NEG	NEG	NEG	NEG
MSSA1955	POS	NEG	NEG	NEG	NEG	NEG	NEG	NEG	NEG	NEG	POS	NEG	NEG	NEG	NEG
MSSA1941	POS	POS	NEG	NEG	NEG	NEG	NEG	NEG	POS	POS	POS	POS	POS	NEG	NEG
MRSA3838	POS	NEG	NEG	NEG	NEG	NEG	NEG	NEG	NEG	POS	POS	NEG	NEG	NEG	NEG
MRSA3839	POS	NEG	NEG	AMB	AMB	NEG	NEG	NEG	NEG	POS	POS	NEG	NEG	NEG	NEG
MSSA1801	POS	NEG	NEG	NEG	NEG	NEG	NEG	NEG	NEG	POS	POS	NEG	NEG	NEG	NEG
MSSA2131	POS	NEG	NEG	POS	POS	NEG	NEG	NEG	NEG	POS	POS	NEG	NEG	NEG	NEG
MSSA1792	POS	NEG	NEG	POS	AMB	POS	NEG	POS	NEG	POS	POS	NEG	NEG	NEG	NEG
MSSA1796	POS	NEG	POS	NEG	NEG	NEG	NEG	NEG	POS	POS	POS	NEG	NEG	POS	NEG
MSSA1817	POS	NEG	NEG	POS	NEG	NEG	NEG	NEG	NEG	NEG	POS	NEG	NEG	NEG	NEG
MSSA1911	POS	NEG	NEG	AMB	NEG	NEG	NEG	NEG	POS	POS	NEG	NEG	NEG	NEG	NEG
MSSA1927	POS	NEG	POS	NEG	NEG	POS	NEG	POS	NEG	POS	POS	NEG	POS	NEG	NEG
MSSA1928	POS	NEG	POS	NEG	NEG	POS	NEG	POS	NEG	POS	POS	NEG	POS	NEG	NEG
MSSA1953	POS	NEG	POS	NEG	NEG	POS	NEG	POS	NEG	POS	POS	NEG	POS	NEG	NEG
MSSA2062	POS	NEG	NEG	POS	POS	POS	NEG	POS	NEG	POS	POS	NEG	NEG	NEG	NEG
MSSA2078	POS	NEG	NEG	NEG	NEG	NEG	NEG	NEG	NEG	NEG	POS	NEG	NEG	NEG	POS
MSSA2136	POS	NEG	NEG	POS	POS	POS	NEG	NEG	POS	POS	POS	NEG	NEG	NEG	NEG

	TYPE I RESTRICTION-MODIFICATION SYSTEM, SINGLE SEQUENCE SPECIFICITY PROTEIN												
Strain	hsdS1 (RF122)	hsdS2 (ST5+8)	hsdS2 (MW2+476)	hsdS2 (RF122)	hsdS2 (MRSA252)	hsdS3 (all other than RF122+252)	hsdS3 (ST8+ST1+RF122)	hsdS3 (Mu50+N315)	hsdS3 (CC51+ MRSA252)	hsdS3 (MRSA252)	hsdSx (CC25)	hsdSx (CC15)	hsdSx (etd)
MSSA1805	NEG	NEG	POS	NEG	NEG	POS	POS	NEG	NEG	NEG	POS	NEG	NEG
MSSA1823	NEG	NEG	POS	NEG	NEG	POS	POS	NEG	NEG	NEG	POS	NEG	NEG
MSSA1837	NEG	NEG	POS	NEG	NEG	POS	POS	NEG	NEG	NEG	POS	NEG	NEG
MSSA2044	NEG	NEG	POS	NEG	NEG	POS	POS	NEG	NEG	NEG	POS	NEG	NEG
MSSA2046	NEG	NEG	POS	NEG	NEG	POS	POS	NEG	NEG	NEG	POS	NEG	NEG
MSSA2059	NEG	NEG	POS	NEG	NEG	POS	POS	NEG	NEG	NEG	POS	NEG	NEG
MSSA2076	NEG	NEG	POS	NEG	NEG	POS	POS	NEG	NEG	NEG	POS	NEG	NEG
MSSA2200	NEG	NEG	POS	NEG	NEG	POS	POS	NEG	NEG	NEG	POS	NEG	NEG
MRSA3797	NEG	POS	NEG	NEG	NEG	POS	NEG	POS	NEG	NEG	POS	NEG	NEG
MRSA3798	NEG	POS	POS	NEG	NEG	POS	NEG	POS	NEG	NEG	POS	AMB	NEG
MRSA3799	NEG	POS	NEG	NEG	NEG	POS	NEG	POS	NEG	NEG	POS	NEG	NEG
MRSA3800	NEG	POS	AMB	NEG	NEG	POS	NEG	POS	NEG	NEG	POS	AMB	NEG
MRSA3801	NEG	POS	POS	NEG	NEG	POS	NEG	POS	NEG	NEG	POS	POS	NEG
MRSA3804	NEG	POS	POS	NEG	NEG	POS	NEG	POS	NEG	NEG	POS	AMB	AMB
MRSA3806	NEG	POS	AMB	NEG	NEG	POS	NEG	POS	NEG	NEG	POS	AMB	NEG
MRSA3961	NEG	POS	AMB	NEG	NEG	POS	NEG	POS	NEG	NEG	POS	AMB	NEG
MRSA3962	NEG	POS	AMB	NEG	NEG	POS	NEG	POS	NEG	NEG	POS	AMB	NEG
MRSA3963	NEG	POS	AMB	NEG	NEG	POS	NEG	POS	NEG	NEG	POS	AMB	NEG
MRSA3966	NEG	POS	NEG	NEG	NEG	POS	NEG	POS	NEG	NEG	POS	NEG	NEG
MRSA3978	NEG	POS	NEG	NEG	NEG	POS	NEG	POS	NEG	NEG	POS	NEG	NEG
MRSA3979	NEG	POS	NEG	NEG	NEG	POS	NEG	POS	NEG	NEG	POS	NEG	NEG
MRSA3980	NEG	POS	NEG	NEG	NEG	POS	NEG	POS	NEG	NEG	POS	NEG	NEG
MRSA4179	NEG	POS	NEG	NEG	NEG	POS	NEG	POS	NEG	NEG	POS	NEG	NEG
MRSA4216	NEG	POS	NEG	NEG	NEG	POS	NEG	POS	NEG	NEG	POS	NEG	NEG
MRSA4217	NEG	POS	NEG	NEG	NEG	POS	NEG	POS	NEG	NEG	POS	NEG	NEG
MRSA4220	NEG	POS	POS	NEG	NEG	POS	NEG	POS	NEG	NEG	POS	AMB	NEG
MRSA4221	NEG	POS	POS	NEG	NEG	POS	NEG	POS	NEG	NEG	POS	AMB	NEG
MRSA4222	NEG	POS	NEG	NEG	NEG	POS	NEG	POS	NEG	NEG	POS	NEG	NEG
MSSA1751	NEG	POS	POS	NEG	NEG	POS	NEG	POS	NEG	NEG	POS	POS	NEG
MSSA1760	NEG	POS	POS	NEG	NEG	POS	NEG	POS	NEG	NEG	POS	AMB	NEG
MSSA1802	NEG	POS	NEG	NEG	NEG	POS	NEG	POS	NEG	NEG	POS	NEG	NEG
MSSA1809	NEG	POS	NEG	NEG	NEG	POS	NEG	POS	NEG	NEG	POS	NEG	NEG
MSSA1815	NEG	POS	NEG	NEG	NEG	POS	NEG	POS	NEG	NEG	POS	NEG	NEG
MSSA1829	NEG	POS	NEG	NEG	NEG	POS	NEG	POS	NEG	NEG	POS	NEG	NEG
MSSA1908	NEG	POS	AMB	NEG	NEG	POS	NEG	POS	NEG	NEG	POS	AMB	NEG
MSSA1960	NEG	POS	NEG	NEG	NEG	POS	NEG	POS	NEG	NEG	POS	NEG	NEG
MSSA1965	NEG	POS	AMB	NEG	NEG	POS	NEG	POS	NEG	NEG	POS	NEG	NEG
MSSA1977	NEG	POS	NEG	NEG	NEG	POS	NEG	POS	NEG	NEG	POS	NEG	NEG
MSSA1978	NEG	POS	AMB	NEG	NEG	POS	NEG	POS	NEG	NEG	POS	NEG	NEG
MSSA1999	NEG	POS	NEG	NEG	NEG	POS	NEG	POS	NEG	NEG	POS	NEG	NEG
MSSA2003	NEG	POS	AMB	NEG	NEG	POS	NEG	POS	NEG	NEG	POS	NEG	NEG
MSSA2006	NEG	POS	AMB	NEG	NEG	POS	NEG	POS	NEG	NEG	POS	NEG	NEG
MSSA2008	NEG	POS	AMB	NEG	NEG	POS	NEG	POS	NEG	NEG	POS	NEG	NEG
MSSA2011	NEG	POS	NEG	NEG	NEG	POS	NEG	POS	NEG	NEG	POS	NEG	NEG
MSSA2017	NEG	POS	NEG	NEG	NEG	POS	NEG	POS	NEG	NEG	POS	NEG	NEG
MSSA2022	NEG	POS	NEG	NEG	NEG	POS	NEG	POS	NEG	NEG	POS	NEG	NEG
MSSA2055	NEG	POS	NEG	NEG	NEG	POS	NEG	POS	NEG	NEG	POS	NEG	NEG
MSSA2060	NEG	POS	NEG	NEG	NEG	POS	NEG	POS	NEG	NEG	POS	NEG	NEG
MSSA2080	NEG	POS	NEG	NEG	NEG	POS	NEG	POS	NEG	NEG	POS	NEG	NEG
MSSA2082	NEG	POS	AMB	NEG	NEG	POS	NEG	POS	NEG	NEG	POS	NEG	NEG
MSSA2084	NEG	POS	NEG	NEG	NEG	POS	NEG	POS	NEG	NEG	POS	NEG	NEG
MSSA2087	NEG	POS	NEG	NEG	NEG	POS	NEG	POS	NEG	NEG	POS	NEG	NEG
MSSA2094	NEG	POS	NEG	NEG	NEG	POS	NEG	POS	NEG	NEG	POS	NEG	NEG
MSSA2097	NEG	POS	NEG	NEG	NEG	POS	NEG	POS	NEG	NEG	POS	NEG	NEG
MSSA2110	NEG	POS	AMB	NEG	NEG	POS	NEG	POS	NEG	NEG	POS	NEG	NEG
MSSA2113	NEG	POS	AMB	NEG	NEG	POS	NEG	POS	NEG	NEG	POS	AMB	NEG
MSSA2132	NEG	POS	AMB	NEG	NEG	POS	NEG	POS	NEG	NEG	POS	NEG	NEG
MSSA2134	NEG	POS	NEG	NEG	NEG	POS	NEG	POS	NEG	NEG	POS	NEG	NEG
MSSA1746	NEG	NEG	NEG	NEG	NEG	NEG	NEG	NEG	NEG	NEG	NEG	NEG	POS
MSSA1757	NEG	NEG	POS	NEG	NEG	NEG	NEG	NEG	NEG	NEG	AMB	POS	POS
MSSA1772	NEG	NEG	NEG	NEG	NEG	NEG	NEG	NEG	NEG	NEG	NEG	NEG	POS
MSSA1795	NEG	NEG	AMB	NEG	NEG	NEG	NEG	NEG	NEG	NEG	AMB	POS	POS
MSSA1813	NEG	NEG	NEG	NEG	NEG	NEG	NEG	NEG	NEG	NEG	NEG	NEG	POS
MSSA1826	NEG	NEG	NEG	NEG	NEG	NEG	NEG	NEG	NEG	NEG	NEG	NEG	POS
MSSA1830	NEG	NEG	NEG	NEG	NEG	NEG	NEG	NEG	NEG	NEG	NEG	NEG	POS
MSSA1838	NEG	NEG	NEG	NEG	NEG	NEG	NEG	NEG	NEG	NEG	NEG	NEG	POS
MSSA1944	NEG	NEG	NEG	NEG	NEG	NEG	NEG	NEG	NEG	NEG	NEG	NEG	POS
MSSA1947	NEG	NEG	NEG	NEG	NEG	NEG	NEG	NEG	NEG	NEG	NEG	NEG	POS
MSSA1964	NEG	NEG	AMB	NEG	NEG	NEG	NEG	NEG	NEG	NEG	NEG	AMB	POS
MSSA1969	NEG	NEG	NEG	NEG	NEG	NEG	NEG	NEG	NEG	NEG	NEG	NEG	POS
MSSA2001	NEG	NEG	AMB	NEG	NEG	NEG	NEG	NEG	NEG	NEG	AMB	AMB	POS
MSSA2004	NEG	NEG	AMB	NEG	NEG	NEG	NEG	NEG	NEG	NEG	AMB	AMB	POS
MSSA2024	NEG	NEG	NEG	NEG	NEG	NEG	NEG	NEG	NEG	NEG	NEG	NEG	AMB
MSSA2025	NEG	NEG	NEG	NEG	NEG	NEG	NEG	NEG	NEG	NEG	NEG	NEG	POS
MSSA2026	NEG	NEG	NEG	NEG	NEG	NEG	NEG	NEG	NEG	NEG	NEG	NEG	POS
MSSA2032	NEG	NEG	NEG	NEG	NEG	NEG	NEG	NEG	NEG	NEG	NEG	NEG	POS
MSSA2043	NEG	NEG	NEG	NEG	NEG	NEG	NEG	NEG	NEG	NEG	NEG	NEG	POS
MSSA2061	NEG	NEG	NEG	NEG	NEG	NEG	NEG	NEG	NEG	NEG	NEG	NEG	POS
MSSA2085	NEG	NEG	NEG	NEG	NEG	NEG	NEG	NEG	NEG	NEG	NEG	NEG	POS
MSSA2098	NEG	NEG	NEG	NEG	NEG	NEG	NEG	NEG	NEG	NEG	NEG	AMB	POS
MSSA2100	NEG	NEG	NEG	NEG	NEG	NEG	NEG	NEG	NEG	NEG	NEG	AMB	POS
MSSA2111	NEG	NEG	AMB	NEG	NEG	NEG	NEG	NEG	NEG	NEG	AMB	POS	POS
MSSA2178	NEG	NEG	POS	NEG	NEG	NEG	NEG	NEG	NEG	NEG	NEG	POS	POS
MSSA2202	NEG	NEG	NEG	NEG	NEG	NEG	NEG	NEG	NEG	NEG	NEG	NEG	POS
MSSA2205	NEG	NEG	NEG	AMB	NEG	NEG	NEG	NEG	NEG	NEG	NEG	AMB	POS
MRSA3796	NEG	POS	AMB	NEG	NEG	POS	POS	NEG	NEG	NEG	POS	AMB	NEG
MRSA3805	NEG	POS	NEG	NEG	NEG	POS	POS	NEG	NEG	NEG	POS	NEG	NEG
MRSA3965	NEG	POS	NEG	NEG	NEG	POS	POS	NEG	NEG	NEG	POS	NEG	NEG
MSSA1761	NEG	NEG	POS	NEG	NEG	POS	POS	NEG	NEG	NEG	POS	POS	AMB
MSSA1764	NEG	POS	NEG	NEG	NEG	POS	POS	NEG	NEG	NEG	POS	NEG	NEG
MSSA1765	NEG	POS	AMB	NEG	NEG	POS	POS	NEG	NEG	NEG	POS	NEG	NEG
MSSA1784	NEG	POS	POS	NEG	NEG	POS	POS	NEG	NEG	NEG	POS	AMB	AMB
MSSA1806	NEG	POS	AMB	NEG	NEG	POS	POS	NEG	NEG	NEG	POS	NEG	NEG
MSSA1807	NEG	POS	NEG	NEG	NEG	POS	POS	NEG	NEG	NEG	POS	NEG	NEG
MSSA1888	NEG	NEG	NEG	NEG	NEG	POS	POS	NEG	NEG	NEG	POS	NEG	NEG
MSSA1894	NEG	POS	NEG	NEG	NEG	POS	POS	NEG	NEG	NEG	POS	NEG	NEG
MSSA1922	NEG	POS	AMB	NEG	NEG	POS	POS	NEG	NEG	NEG	POS	NEG	NEG
MSSA1950	NEG	POS	AMB	NEG	NEG	POS	POS	NEG	NEG	NEG	POS	AMB	NEG
MSSA1954	NEG	POS	NEG	NEG	NEG	POS	POS	NEG	NEG	NEG	POS	NEG	NEG
MSSA1966	NEG	NEG	POS	NEG	NEG	POS	POS	NEG	NEG	NEG	POS	NEG	NEG
MSSA1972	NEG	POS	AMB	NEG	NEG	POS	POS	NEG	NEG	NEG	POS	NEG	NEG
MSSA2010	NEG	POS	AMB	NEG	NEG	POS	POS	NEG	NEG	NEG	POS	NEG	NEG
MSSA2020	NEG	POS	NEG	NEG	NEG	POS	POS	NEG	NEG	NEG	POS	NEG	NEG
MSSA2028	NEG	POS	POS	NEG	NEG	POS	POS	NEG	NEG	NEG	POS	NEG	NEG
MSSA2088	NEG	POS	NEG	NEG	NEG	POS	POS	NEG	NEG	NEG	POS	NEG	NEG
MSSA2187	NEG	NEG	NEG	NEG	NEG	POS	POS	NEG	NEG	NEG	POS	NEG	NEG
MSSA2204	NEG	POS	AMB	NEG	NEG	POS	POS	NEG	NEG	NEG	POS	AMB	NEG
MSSA2210	NEG	POS	NEG	NEG	NEG	POS	POS	NEG	NEG	NEG	POS	NEG	NEG
MSSA2223	NEG	POS	NEG	NEG	NEG	POS	POS	NEG	NEG	NEG	POS	NEG	NEG
MSSA1763	NEG	NEG	POS	NEG	NEG	NEG	NEG	NEG	NEG	NEG	NEG	NEG	NEG
MSSA1777	NEG	NEG	POS	NEG	NEG	AMB	NEG	NEG	NEG	NEG	AMB	AMB	NEG
MSSA2009	NEG	NEG	POS	NEG	NEG	NEG	NEG	NEG	NEG	NEG	NEG	NEG	NEG
MSSA2047	NEG	NEG	POS	NEG	NEG	NEG	NEG	NEG	NEG	NEG	NEG	NEG	NEG
MSSA2079	NEG	NEG	POS	NEG	NEG	NEG	NEG	NEG	NEG	NEG	NEG	NEG	NEG
MSSA2101	NEG	NEG	POS	NEG	NEG	NEG	NEG	NEG	NEG	NEG	NEG	NEG	NEG
MSSA1821	NEG	POS	NEG	NEG	NEG	AMB	NEG	NEG	POS	NEG	POS	NEG	NEG
MSSA1898	NEG	POS	NEG	NEG	NEG	NEG	NEG	NEG	POS	NEG	POS	NEG	NEG
MSSA1973	NEG	POS	AMB	NEG	NEG	NEG	NEG	NEG	POS	NEG	POS	NEG	NEG
MSSA2071	NEG	POS	NEG	NEG	NEG	AMB	NEG	NEG	POS	NEG	POS	AMB	NEG
MSSA2083	NEG	POS	NEG	NEG	NEG	AMB	NEG	NEG	POS	NEG	POS	NEG	NEG
MSSA2106	NEG	POS	AMB	NEG	NEG	AMB	NEG	NEG	POS	NEG	POS	NEG	NEG
MSSA2112	NEG	POS	POS	NEG	NEG	AMB	NEG	NEG	POS	NEG	POS	POS	NEG
MSSA2115	NEG	POS	POS	NEG	NEG	AMB	NEG	NEG	POS	NEG	POS	POS	NEG
MSSA2195	NEG	POS	NEG	NEG	NEG	POS	NEG	NEG	POS	NEG	POS	AMB	NEG
MSSA2207	NEG	POS	AMB	NEG	NEG	POS	NEG	NEG	POS	NEG	POS	AMB	NEG
MSSA1758	NEG	NEG	NEG	NEG	NEG	NEG	NEG	NEG	NEG	NEG	NEG	POS	NEG
MSSA1762	NEG	NEG	AMB	NEG	NEG	NEG	NEG	NEG	NEG	NEG	NEG	POS	AMB
MSSA1769	NEG	NEG	AMB	NEG	NEG	NEG	NEG	NEG	NEG	NEG	NEG	POS	AMB
MSSA1771	NEG	NEG	NEG	NEG	NEG	NEG	NEG	NEG	NEG	NEG	NEG	POS	NEG
MSSA1789	NEG	NEG	NEG	NEG	NEG	NEG	NEG	NEG	NEG	NEG	NEG	POS	NEG
MSSA1800	NEG	NEG	NEG	NEG	NEG	NEG	NEG	NEG	NEG	NEG	NEG	POS	NEG
MSSA1833	NEG	NEG	NEG	NEG	NEG	NEG	NEG	NEG	NEG	NEG	AMB	POS	NEG
MSSA1890	NEG	NEG	NEG	NEG	NEG	NEG	NEG	NEG	NEG	NEG	NEG	POS	NEG
MSSA1903	NEG	NEG	NEG	NEG	NEG	NEG	NEG	NEG	NEG	NEG	NEG	POS	NEG
MSSA1940	NEG	NEG	AMB	NEG	NEG	NEG	NEG	NEG	NEG	NEG	NEG	POS	NEG
MSSA1962	NEG	NEG	NEG	NEG	NEG	NEG	NEG	NEG	NEG	NEG	NEG	POS	NEG
MSSA1963	NEG	NEG	AMB	NEG	NEG	NEG	NEG	NEG	NEG	NEG	NEG	POS	NEG
MSSA2016	NEG	NEG	NEG	NEG	NEG	NEG	NEG	NEG	NEG	NEG	NEG	POS	NEG
MSSA2018	NEG	NEG	NEG	NEG	NEG	NEG	NEG	NEG	NEG	NEG	AMB	POS	NEG
MSSA2019	NEG	NEG	NEG	NEG	NEG	NEG	NEG	NEG	NEG	NEG	NEG	POS	NEG
MSSA2029	NEG	NEG	NEG	NEG	NEG	NEG	NEG	NEG	NEG	NEG	AMB	POS	NEG
MSSA2045	NEG	NEG	NEG	NEG	NEG	NEG	NEG	NEG	NEG	NEG	NEG	POS	NEG
MSSA2048	NEG	NEG	NEG	NEG	NEG	NEG	NEG	NEG	NEG	NEG	NEG	POS	NEG
MSSA2049	NEG	NEG	NEG	NEG	NEG	NEG	NEG	NEG	NEG	NEG	NEG	POS	NEG
MSSA2050	NEG	NEG	NEG	NEG	NEG	AMB	NEG	NEG	NEG	NEG	POS	POS	NEG
MSSA2099	NEG	NEG	NEG	NEG	NEG	NEG	NEG	NEG	NEG	NEG	NEG	POS	NEG
MSSA2109	NEG	NEG	NEG	NEG	NEG	NEG	NEG	NEG	NEG	NEG	NEG	POS	NEG
MSSA2130	NEG	NEG	AMB	NEG	NEG	NEG	NEG	NEG	NEG	NEG	NEG	POS	NEG
MSSA2133	NEG	NEG	NEG	NEG	NEG	NEG	NEG	NEG	NEG	NEG	NEG	POS	NEG
MSSA2139	NEG	NEG	NEG	NEG	NEG	NEG	NEG	NEG	NEG	NEG	NEG	POS	NEG
MSSA2141	NEG	NEG	AMB	NEG	NEG	NEG	NEG	NEG	NEG	NEG	AMB	POS	NEG
MSSA2208	NEG	NEG	NEG	NEG	NEG	NEG	NEG	NEG	NEG	NEG	NEG	POS	NEG
MSSA2209	NEG	NEG	NEG	NEG	NEG	NEG	NEG	NEG	NEG	NEG	NEG	POS	NEG
MSSA1893	NEG	NEG	POS	NEG	NEG	NEG	NEG	NEG	NEG	NEG	NEG	NEG	NEG
MSSA2105	NEG	NEG	POS	NEG	NEG	NEG	NEG	NEG	NEG	NEG	NEG	NEG	NEG
MRSA3836	NEG	POS	NEG	NEG	NEG	AMB	NEG	NEG	NEG	NEG	POS	NEG	NEG
MRSA3840	NEG	POS	NEG	NEG	NEG	AMB	NEG	NEG	NEG	NEG	POS	NEG	NEG
MRSA4188	NEG	POS	POS	NEG	NEG	POS	NEG	NEG	NEG	NEG	POS	POS	NEG
MSSA1814	NEG	POS	NEG	NEG	NEG	NEG	NEG	NEG	NEG	NEG	POS	NEG	NEG
MSSA1816	NEG	POS	NEG	NEG	NEG	NEG	NEG	NEG	NEG	NEG	NEG	NEG	NEG
MSSA1825	NEG	POS	NEG	NEG	NEG	AMB	NEG	NEG	NEG	NEG	POS	NEG	NEG
MSSA1834	NEG	POS	NEG	NEG	NEG	NEG	NEG	NEG	NEG	NEG	NEG	NEG	NEG
MSSA1938	NEG	POS	NEG	NEG	NEG	NEG	NEG	NEG	NEG	NEG	NEG	NEG	NEG
MSSA1948	NEG	POS	AMB	NEG	NEG	NEG	NEG	NEG	NEG	NEG	AMB	AMB	NEG
MSSA1951	NEG	POS	NEG	NEG	NEG	NEG	NEG	NEG	NEG	NEG	AMB	NEG	NEG
MSSA2005	NEG	POS	AMB	NEG	NEG	NEG	NEG	NEG	NEG	NEG	AMB	NEG	NEG
MSSA2051	NEG	POS	NEG	NEG	NEG	POS	NEG	NEG	NEG	NEG	POS	AMB	NEG
MSSA2056	NEG	POS	NEG	NEG	NEG	NEG	NEG	NEG	NEG	NEG	NEG	NEG	NEG
MSSA2066	NEG	POS	AMB	NEG	NEG	AMB	NEG	NEG	NEG	NEG	POS	AMB	NEG
MSSA2067	NEG	POS	NEG	NEG	NEG	NEG	NEG	NEG	NEG	NEG	NEG	NEG	NEG
MSSA2086	NEG	POS	NEG	NEG	NEG	NEG	NEG	NEG	NEG	NEG	AMB	NEG	NEG
MSSA2089	NEG	POS	NEG	NEG	NEG	NEG	NEG	NEG	NEG	NEG	AMB	NEG	NEG
MSSA2103	NEG	POS	NEG	NEG	NEG	NEG	NEG	NEG	NEG	NEG	NEG	NEG	NEG
MSSA2173	NEG	POS	AMB	NEG	NEG	NEG	NEG	NEG	NEG	NEG	AMB	AMB	NEG
MSSA2183	NEG	POS	NEG	NEG	NEG	AMB	NEG	NEG	NEG	NEG	POS	NEG	NEG
MSSA2196	NEG	POS	NEG	NEG	NEG	AMB	NEG	NEG	NEG	NEG	POS	AMB	NEG
MSSA2219	NEG	POS	NEG	NEG	NEG	POS	NEG	NEG	NEG	NEG	POS	AMB	NEG
MSSA1753	NEG	NEG	NEG	NEG	NEG	POS	NEG	POS	NEG	NEG	POS	NEG	POS
MSSA1773	NEG	NEG	NEG	NEG	NEG	POS	NEG	POS	NEG	NEG	POS	NEG	POS
MSSA1811	NEG	NEG	NEG	NEG	NEG	POS	NEG	POS	NEG	NEG	POS	NEG	POS
MSSA1897	NEG	NEG	POS	NEG	NEG	POS	NEG	POS	NEG	NEG	POS	NEG	POS
MSSA1931	NEG	NEG	NEG	NEG	NEG	POS	NEG	POS	NEG	NEG	POS	NEG	POS
MSSA2114	NEG	NEG	POS	NEG	NEG	POS	NEG	POS	NEG	NEG	POS	AMB	POS
MSSA1745	NEG	NEG	NEG	NEG	POS	AMB	NEG	NEG	POS	POS	POS	AMB	NEG
MSSA1747	NEG	NEG	NEG	NEG	POS	NEG	NEG	NEG	POS	POS	POS	NEG	NEG
MSSA1749	NEG	NEG	AMB	NEG	POS	NEG	NEG	NEG	POS	POS	POS	NEG	NEG
MSSA1768	NEG	NEG	NEG	NEG	POS	NEG	NEG	NEG	POS	POS	AMB	NEG	NEG
MSSA1774	NEG	NEG	NEG	NEG	POS	NEG	NEG	NEG	POS	POS	AMB	NEG	NEG
MSSA1775	NEG	NEG	POS	NEG	POS	NEG	NEG	NEG	POS	POS	POS	POS	NEG
MSSA1776	NEG	NEG	POS	NEG	POS	AMB	NEG	NEG	POS	POS	POS	POS	NEG
MSSA1779	NEG	NEG	NEG	NEG	POS	NEG	NEG	NEG	POS	POS	AMB	NEG	NEG
MSSA1780	NEG	NEG	NEG	NEG	POS	NEG	NEG	NEG	POS	POS	POS	AMB	NEG

	TYPE I RESTRICTION-MODIFICATION SYSTEM, SINGLE SEQUENCE SPECIFICITY PROTEIN												
Strain	hsdS1 (RF122)	hsdS2 (ST5+8)	hsdS2 (MW2+476)	hsdS2 (RF122)	hsdS2 (MRSA252)	hsdS3 (all other than RF122+252)	hsdS3 (ST8+ST1+RF122)	hsdS3 (Mu50+N315)	hsdS3 (CC51+ MRSA252)	hsdS3 (MRSA252)	hsdSx (CC25)	hsdSx (CC15)	hsdSx (etd)
MSSA1781	NEG	NEG	AMB	NEG	POS	NEG	NEG	NEG	POS	POS	POS	POS	NEG
MSSA1786	NEG	NEG	AMB	NEG	POS	NEG	NEG	NEG	POS	POS	POS	AMB	NEG
MSSA1793	NEG	NEG	NEG	NEG	POS	NEG	NEG	NEG	POS	POS	AMB	NEG	NEG
MSSA1803	NEG	NEG	NEG	NEG	POS	NEG	NEG	NEG	POS	POS	POS	NEG	NEG
MSSA1836	NEG	NEG	NEG	NEG	POS	NEG	NEG	NEG	POS	POS	POS	NEG	NEG
MSSA1839	NEG	NEG	NEG	NEG	POS	NEG	NEG	NEG	POS	POS	POS	NEG	NEG
MSSA1900	NEG	NEG	NEG	NEG	POS	NEG	NEG	NEG	POS	POS	POS	AMB	NEG
MSSA1910	NEG	NEG	NEG	NEG	POS	NEG	NEG	NEG	POS	POS	POS	AMB	NEG
MSSA1918	NEG	NEG	NEG	NEG	POS	NEG	NEG	NEG	POS	POS	POS	NEG	NEG
MSSA1921	NEG	NEG	NEG	NEG	POS	NEG	NEG	NEG	POS	POS	POS	NEG	NEG
MSSA1923	NEG	NEG	NEG	NEG	POS	NEG	NEG	NEG	POS	POS	POS	NEG	NEG
MSSA1981	NEG	NEG	NEG	NEG	POS	NEG	NEG	NEG	POS	POS	POS	NEG	NEG
MSSA1998	NEG	NEG	NEG	NEG	POS	NEG	NEG	NEG	POS	POS	POS	AMB	NEG
MSSA2000	NEG	NEG	NEG	NEG	POS	NEG	NEG	NEG	POS	POS	AMB	NEG	NEG
MSSA2007	NEG	NEG	NEG	NEG	POS	NEG	NEG	NEG	POS	POS	POS	AMB	NEG
MSSA2012	NEG	NEG	AMB	NEG	POS	NEG	NEG	NEG	POS	POS	POS	AMB	NEG
MSSA2015	NEG	NEG	NEG	NEG	POS	NEG	NEG	NEG	POS	POS	NEG	AMB	NEG
MSSA2021	NEG	NEG	NEG	NEG	POS	NEG	NEG	NEG	POS	POS	NEG	NEG	NEG
MSSA2027	NEG	NEG	NEG	NEG	POS	NEG	NEG	NEG	POS	POS	POS	NEG	NEG
MSSA2063	NEG	NEG	NEG	NEG	POS	NEG	NEG	NEG	POS	POS	POS	NEG	NEG
MSSA2064	NEG	NEG	AMB	NEG	POS	NEG	NEG	NEG	POS	POS	POS	AMB	NEG
MSSA2065	NEG	NEG	NEG	NEG	POS	NEG	NEG	NEG	POS	POS	AMB	NEG	NEG
MSSA2075	NEG	NEG	NEG	NEG	POS	NEG	NEG	NEG	POS	POS	POS	NEG	NEG
MSSA2093	NEG	NEG	NEG	NEG	POS	NEG	NEG	NEG	POS	POS	POS	AMB	NEG
MSSA2102	NEG	NEG	NEG	NEG	POS	NEG	NEG	NEG	POS	POS	POS	NEG	NEG
MSSA2138	NEG	NEG	AMB	NEG	POS	NEG	NEG	NEG	POS	POS	POS	AMB	NEG
MSSA2168	NEG	NEG	AMB	NEG	POS	NEG	NEG	NEG	POS	POS	POS	POS	NEG
MSSA2181	NEG	NEG	NEG	NEG	POS	NEG	NEG	NEG	POS	POS	POS	NEG	NEG
MSSA2189	NEG	NEG	POS	NEG	POS	NEG	NEG	NEG	POS	POS	POS	POS	NEG
MSSA2214	NEG	NEG	NEG	NEG	POS	NEG	NEG	NEG	POS	POS	POS	NEG	NEG
MRSA3803	NEG	NEG	AMB	NEG	POS	NEG	NEG	NEG	NEG	NEG	AMB	POS	NEG
MSSA1750	NEG	NEG	POS	NEG	POS	NEG	NEG	NEG	NEG	NEG	POS	POS	POS
MSSA1756	NEG	NEG	NEG	NEG	POS	NEG	NEG	NEG	NEG	NEG	POS	POS	AMB
MSSA1759	NEG	NEG	AMB	NEG	POS	NEG	NEG	NEG	NEG	NEG	POS	POS	NEG
MSSA1767	NEG	NEG	POS	NEG	POS	NEG	NEG	NEG	NEG	NEG	POS	AMB	NEG
MSSA1787	NEG	NEG	NEG	NEG	POS	NEG	NEG	NEG	NEG	NEG	AMB	AMB	NEG
MSSA1790	NEG	NEG	NEG	NEG	POS	NEG	NEG	NEG	NEG	NEG	POS	POS	NEG
MSSA1794	NEG	NEG	NEG	NEG	POS	NEG	NEG	NEG	NEG	NEG	NEG	NEG	NEG
MSSA1808	NEG	NEG	NEG	NEG	POS	NEG	NEG	NEG	NEG	NEG	AMB	NEG	NEG
MSSA1812	NEG	NEG	NEG	NEG	POS	NEG	NEG	NEG	NEG	NEG	AMB	NEG	NEG
MSSA1819	NEG	NEG	NEG	NEG	POS	NEG	NEG	NEG	NEG	NEG	POS	NEG	NEG
MSSA1824	NEG	NEG	NEG	NEG	POS	NEG	NEG	NEG	NEG	NEG	POS	NEG	NEG
MSSA1827	NEG	NEG	NEG	NEG	POS	NEG	NEG	NEG	NEG	NEG	POS	NEG	NEG
MSSA1889	NEG	NEG	NEG	NEG	POS	NEG	NEG	NEG	NEG	NEG	AMB	NEG	NEG
MSSA1895	NEG	NEG	NEG	NEG	POS	NEG	NEG	NEG	NEG	NEG	NEG	NEG	NEG
MSSA1904	NEG	NEG	AMB	NEG	POS	NEG	NEG	NEG	NEG	NEG	AMB	NEG	NEG
MSSA1905	NEG	NEG	NEG	NEG	POS	NEG	NEG	NEG	NEG	NEG	NEG	NEG	NEG
MSSA1907	NEG	NEG	AMB	NEG	POS	NEG	NEG	NEG	NEG	NEG	NEG	NEG	NEG
MSSA1919	NEG	NEG	AMB	NEG	POS	NEG	NEG	NEG	NEG	NEG	AMB	NEG	NEG
MSSA1920	AMB	NEG	NEG	NEG	POS	NEG	NEG	NEG	NEG	NEG	AMB	NEG	NEG
MSSA1929	NEG	NEG	NEG	NEG	POS	NEG	NEG	NEG	NEG	NEG	AMB	NEG	NEG
MSSA1930	NEG	NEG	AMB	NEG	POS	NEG	NEG	NEG	NEG	NEG	AMB	AMB	NEG
MSSA1934	NEG	NEG	NEG	NEG	POS	NEG	NEG	NEG	NEG	NEG	AMB	AMB	NEG
MSSA1946	NEG	NEG	NEG	NEG	POS	NEG	NEG	NEG	NEG	NEG	NEG	NEG	NEG
MSSA1971	NEG	NEG	AMB	NEG	POS	NEG	NEG	NEG	NEG	NEG	AMB	POS	NEG
MSSA1980	NEG	NEG	NEG	NEG	POS	NEG	NEG	NEG	NEG	NEG	NEG	NEG	NEG
MSSA1982	NEG	NEG	NEG	NEG	POS	NEG	NEG	NEG	NEG	NEG	AMB	NEG	NEG
MSSA1996	NEG	NEG	NEG	NEG	POS	NEG	NEG	NEG	NEG	NEG	AMB	NEG	NEG
MSSA2002	NEG	NEG	NEG	NEG	POS	NEG	NEG	NEG	NEG	NEG	AMB	AMB	NEG
MSSA2014	NEG	NEG	NEG	NEG	POS	NEG	NEG	NEG	NEG	NEG	AMB	AMB	NEG
MSSA2053	NEG	NEG	NEG	NEG	POS	NEG	NEG	NEG	NEG	NEG	AMB	NEG	NEG
MSSA2054	NEG	NEG	NEG	NEG	POS	NEG	NEG	NEG	NEG	NEG	NEG	NEG	NEG
MSSA2058	NEG	NEG	NEG	NEG	POS	NEG	NEG	NEG	NEG	NEG	NEG	NEG	NEG
MSSA2068	NEG	NEG	AMB	NEG	POS	NEG	NEG	NEG	NEG	NEG	POS	AMB	NEG
MSSA2070	NEG	NEG	NEG	NEG	POS	NEG	NEG	NEG	NEG	NEG	AMB	NEG	NEG
MSSA2072	NEG	NEG	NEG	NEG	POS	NEG	NEG	NEG	NEG	NEG	AMB	AMB	NEG
MSSA2073	NEG	NEG	NEG	NEG	POS	NEG	NEG	NEG	NEG	NEG	NEG	NEG	NEG
MSSA2074	NEG	NEG	POS	NEG	POS	NEG	NEG	NEG	NEG	NEG	AMB	POS	NEG
MSSA2107	NEG	NEG	NEG	NEG	POS	NEG	NEG	NEG	NEG	NEG	POS	AMB	NEG
MSSA2108	NEG	NEG	NEG	NEG	POS	NEG	NEG	NEG	NEG	NEG	NEG	NEG	NEG
MSSA2129	NEG	NEG	NEG	NEG	POS	NEG	NEG	NEG	NEG	NEG	POS	AMB	NEG
MSSA2135	NEG	NEG	NEG	NEG	POS	NEG	NEG	NEG	NEG	NEG	POS	AMB	NEG
MSSA2137	NEG	NEG	AMB	NEG	POS	POS	NEG	NEG	POS	NEG	POS	AMB	NEG
MSSA2140	NEG	NEG	AMB	NEG	POS	NEG	NEG	NEG	NEG	NEG	AMB	AMB	NEG
MSSA2182	NEG	NEG	NEG	NEG	POS	NEG	NEG	NEG	NEG	NEG	NEG	AMB	NEG
MSSA2188	NEG	NEG	NEG	NEG	POS	NEG	NEG	NEG	NEG	NEG	AMB	NEG	NEG
MSSA1899	NEG	NEG	NEG	NEG	NEG	NEG	NEG	NEG	POS	NEG	NEG	AMB	POS
MSSA1915	NEG	NEG	NEG	NEG	NEG	NEG	NEG	NEG	POS	NEG	NEG	NEG	POS
MSSA1949	NEG	NEG	AMB	NEG	NEG	NEG	NEG	NEG	POS	NEG	NEG	AMB	POS
MSSA2042	NEG	NEG	NEG	NEG	NEG	NEG	NEG	NEG	POS	NEG	NEG	NEG	POS
MSSA2077	NEG	NEG	NEG	NEG	NEG	NEG	NEG	NEG	POS	NEG	NEG	NEG	POS
MSSA2095	NEG	NEG	NEG	NEG	NEG	AMB	NEG	NEG	POS	NEG	NEG	POS	POS
MSSA1997	POS	NEG	NEG	NEG	NEG	POS	NEG	NEG	NEG	POS	POS	NEG	NEG
MSSA1810	NEG	NEG	NEG	NEG	NEG	NEG	NEG	NEG	POS	NEG	NEG	NEG	POS
MSSA1832	NEG	NEG	NEG	NEG	NEG	AMB	NEG	NEG	NEG	NEG	POS	NEG	NEG
MSSA1891	NEG	NEG	AMB	NEG	NEG	AMB	NEG	NEG	NEG	NEG	POS	AMB	NEG
MSSA1892	NEG	NEG	AMB	NEG	NEG	AMB	NEG	NEG	NEG	NEG	POS	AMB	NEG
MSSA1932	NEG	NEG	NEG	NEG	NEG	AMB	NEG	NEG	NEG	NEG	POS	NEG	NEG
MSSA1942	NEG	NEG	NEG	NEG	NEG	AMB	NEG	NEG	NEG	NEG	POS	NEG	NEG
MSSA1957	NEG	NEG	NEG	NEG	NEG	NEG	NEG	NEG	NEG	NEG	POS	NEG	NEG
MSSA1748	NEG	POS	AMB	NEG	NEG	AMB	NEG	NEG	NEG	NEG	POS	NEG	POS
MSSA1754	NEG	POS	POS	NEG	NEG	AMB	NEG	NEG	NEG	NEG	POS	NEG	POS
MSSA1770	NEG	POS	NEG	NEG	NEG	NEG	NEG	NEG	NEG	NEG	POS	NEG	POS
MSSA1788	NEG	POS	NEG	NEG	NEG	NEG	NEG	NEG	NEG	NEG	AMB	NEG	POS
MSSA1804	NEG	POS	NEG	NEG	NEG	POS	NEG	NEG	NEG	NEG	POS	NEG	POS
MSSA1818	NEG	POS	NEG	NEG	NEG	NEG	NEG	NEG	NEG	NEG	POS	NEG	POS
MSSA1914	NEG	POS	AMB	NEG	NEG	POS	NEG	NEG	NEG	NEG	POS	POS	POS
MSSA1974	NEG	POS	NEG	NEG	NEG	NEG	NEG	NEG	NEG	NEG	AMB	NEG	POS
MSSA2023	NEG	POS	NEG	NEG	NEG	AMB	NEG	NEG	NEG	NEG	POS	NEG	POS
MSSA2030	NEG	POS	NEG	NEG	NEG	AMB	NEG	NEG	NEG	NEG	POS	NEG	POS
MSSA2096	NEG	POS	NEG	NEG	NEG	NEG	NEG	NEG	NEG	NEG	NEG	NEG	POS
MSSA2142	NEG	POS	POS	NEG	NEG	NEG	NEG	NEG	NEG	NEG	POS	AMB	POS
MSSA2203	NEG	POS	NEG	NEG	NEG	NEG	NEG	NEG	NEG	NEG	POS	NEG	POS
MSSA1901	NEG	POS	AMB	NEG	NEG	NEG	NEG	NEG	POS	NEG	POS	NEG	NEG
MSSA2013	NEG	POS	AMB	NEG	NEG	NEG	NEG	NEG	POS	NEG	POS	AMB	NEG
MSSA2031	NEG	NEG	AMB	NEG	NEG	AMB	NEG	NEG	POS	NEG	POS	AMB	NEG
MSSA2052	NEG	POS	NEG	NEG	NEG	NEG	NEG	NEG	POS	NEG	POS	NEG	NEG
MSSA2057	NEG	POS	NEG	NEG	NEG	NEG	NEG	NEG	POS	NEG	POS	NEG	NEG
MSSA2081	NEG	NEG	NEG	NEG	NEG	POS	NEG	NEG	POS	NEG	NEG	AMB	POS
MSSA1955	NEG	NEG	POS	NEG	NEG	NEG	NEG	NEG	NEG	NEG	NEG	NEG	NEG
MSSA1941	POS	NEG	AMB	NEG	NEG	NEG	NEG	NEG	NEG	POS	NEG	NEG	NEG
MRSA3838	NEG	NEG	NEG	NEG	NEG	NEG	NEG	NEG	NEG	NEG	NEG	POS	NEG
MRSA3839	NEG	NEG	NEG	NEG	NEG	NEG	NEG	NEG	NEG	NEG	NEG	POS	NEG
MSSA1801	NEG	POS	NEG	NEG	NEG	NEG	NEG	NEG	NEG	NEG	NEG	NEG	NEG
MSSA2131	NEG	NEG	NEG	NEG	NEG	NEG	NEG	NEG	NEG	NEG	NEG	POS	NEG
MSSA1792	NEG	NEG	NEG	NEG	NEG	NEG	NEG	NEG	POS	NEG	POS	NEG	NEG
MSSA1796	NEG	NEG	AMB	NEG	NEG	AMB	NEG	AMB	POS	NEG	POS	POS	POS
MSSA1817	NEG	NEG	NEG	POS	NEG	NEG	NEG	NEG	POS	NEG	POS	NEG	NEG
MSSA1911	POS	POS	NEG	NEG	NEG	NEG	NEG	NEG	NEG	NEG	NEG	NEG	NEG
MSSA1927	NEG	NEG	NEG	NEG	NEG	POS	POS	NEG	POS	NEG	POS	AMB	NEG
MSSA1928	NEG	NEG	NEG	NEG	NEG	POS	POS	NEG	POS	NEG	POS	NEG	NEG
MSSA1953	NEG	NEG	NEG	NEG	NEG	POS	POS	NEG	POS	NEG	POS	NEG	NEG
MSSA2062	NEG	NEG	NEG	NEG	NEG	NEG	NEG	NEG	POS	NEG	POS	NEG	NEG
MSSA2078	NEG	POS	AMB	NEG	NEG	NEG	NEG	NEG	NEG	NEG	POS	NEG	NEG
MSSA2136	NEG	NEG	AMB	NEG	NEG	POS	NEG	NEG	NEG	NEG	POS	AMB	NEG

	HYALURONATE LYASE								IMMUNODOMI- NANT ANTIGEN B		DEFENSIN RESISTANCE		TRANSFERRIN BINDING PROTEIN			PUTATIVE TRANSPORTER				MISCELLANEOUS GENES		
Strain	hysA1 (MRSA252)	hysA1 (MRSA252+RF122 and/or hysA2 (cons)	hysA1 (MRSA252+RF122) and/or hysA2 (COL+USA300)	hysA2 (Other than MRSA252)	hysA2 (COL+ USA300+ NCTC8325)	hysA2 (Other than COL+ USA300+ NCTC8325) (probe 1)	hysA2 (Other than COL+ USA300+ NCTC8325) (probe 2)	hysA2 (MRSA252)	isaB	isaB (MRSA252)	mprF (COL+MW2)	mprF (Mu50+252)	isdA	isdA (MRSA252)	isdA (Other than MRSA252)	lmrP (Other than RF122) (probe 1)	lmrP (Other than RF122) (probe 2)	lmrP (RF122) (probe 1)	lmrP (RF122) (probe 2)	Q2FXC0	Q2YUB3	Q7A4X2
MSSA1805	NEG	POS	NEG	POS	NEG	POS	POS	NEG	POS	AMB	POS	AMB	POS	NEG	POS	POS	POS	NEG	NEG	POS	NEG	NEG
MSSA1823	NEG	POS	NEG	POS	NEG	POS	POS	AMB	POS	AMB	POS	AMB	POS	NEG	POS	POS	POS	NEG	NEG	POS	NEG	NEG
MSSA1837	NEG	POS	NEG	POS	NEG	POS	POS	AMB	POS	AMB	POS	AMB	POS	NEG	POS	POS	POS	NEG	NEG	POS	NEG	NEG
MSSA2044	NEG	POS	NEG	POS	NEG	POS	POS	NEG	POS	AMB	POS	AMB	POS	NEG	POS	POS	POS	NEG	NEG	POS	NEG	NEG
MSSA2046	NEG	POS	NEG	POS	NEG	POS	POS	AMB	POS	AMB	POS	AMB	POS	NEG	POS	POS	POS	NEG	NEG	POS	NEG	NEG
MSSA2059	NEG	POS	NEG	POS	NEG	POS	POS	NEG	POS	AMB	POS	NEG	POS	NEG	POS	POS	POS	NEG	NEG	POS	NEG	NEG
MSSA2076	NEG	POS	NEG	POS	NEG	POS	POS	NEG	POS	AMB	POS	AMB	POS	NEG	POS	POS	POS	NEG	NEG	POS	NEG	NEG
MSSA2200	NEG	POS	NEG	POS	NEG	POS	POS	NEG	POS	AMB	POS	AMB	POS	NEG	POS	POS	POS	NEG	NEG	POS	NEG	NEG
MRSA3797	NEG	POS	NEG	POS	NEG	POS	POS	NEG	POS	AMB	AMB	POS	POS	NEG	POS	POS	POS	NEG	NEG	NEG	NEG	NEG
MRSA3798	NEG	POS	NEG	POS	NEG	POS	POS	AMB	POS	AMB	POS	AMB	POS	NEG	POS	POS	POS	NEG	NEG	NEG	AMB	AMB
MRSA3799	NEG	POS	NEG	POS	NEG	POS	POS	NEG	POS	AMB	AMB	POS	POS	NEG	POS	POS	POS	NEG	NEG	NEG	NEG	NEG
MRSA3800	NEG	POS	NEG	POS	NEG	POS	POS	AMB	POS	AMB	POS	AMB	POS	NEG	POS	POS	POS	NEG	NEG	NEG	AMB	AMB
MRSA3801	NEG	POS	NEG	POS	NEG	POS	POS	AMB	POS	AMB	AMB	POS	POS	NEG	POS	POS	POS	NEG	NEG	NEG	AMB	AMB
MRSA3804	NEG	POS	NEG	POS	NEG	POS	POS	AMB	POS	AMB	POS	AMB	POS	NEG	POS	POS	POS	NEG	NEG	NEG	AMB	AMB
MRSA3806	NEG	POS	NEG	POS	NEG	POS	POS	NEG	POS	AMB	AMB	POS	POS	NEG	POS	POS	POS	NEG	NEG	NEG	NEG	NEG
MRSA3961	NEG	POS	NEG	POS	NEG	POS	POS	NEG	POS	AMB	AMB	POS	POS	NEG	POS	POS	POS	NEG	NEG	NEG	NEG	NEG
MRSA3962	NEG	POS	NEG	POS	NEG	POS	POS	NEG	POS	AMB	AMB	POS	POS	NEG	POS	POS	POS	NEG	NEG	NEG	NEG	NEG
MRSA3963	NEG	POS	NEG	POS	NEG	POS	POS	NEG	POS	AMB	AMB	POS	POS	NEG	POS	POS	POS	NEG	NEG	NEG	NEG	NEG
MRSA3966	NEG	POS	NEG	POS	NEG	POS	POS	NEG	POS	AMB	AMB	POS	POS	NEG	POS	POS	POS	NEG	NEG	NEG	NEG	NEG
MRSA3978	NEG	POS	NEG	POS	NEG	POS	POS	NEG	POS	AMB	AMB	POS	POS	NEG	POS	POS	POS	NEG	NEG	NEG	NEG	NEG
MRSA3979	NEG	POS	NEG	POS	NEG	POS	POS	NEG	POS	AMB	NEG	POS	POS	NEG	POS	POS	POS	NEG	NEG	NEG	NEG	NEG
MRSA3980	NEG	POS	NEG	POS	NEG	POS	POS	NEG	POS	AMB	AMB	POS	POS	NEG	POS	POS	POS	NEG	NEG	NEG	NEG	NEG
MRSA4179	NEG	POS	NEG	POS	NEG	POS	POS	NEG	POS	AMB	AMB	POS	POS	NEG	POS	POS	POS	NEG	NEG	NEG	NEG	NEG
MRSA4216	NEG	POS	NEG	POS	NEG	POS	POS	NEG	POS	AMB	AMB	POS	POS	NEG	POS	POS	POS	NEG	NEG	NEG	NEG	NEG
MRSA4217	NEG	POS	NEG	POS	NEG	POS	POS	NEG	POS	AMB	AMB	POS	POS	NEG	POS	POS	POS	NEG	NEG	NEG	AMB	AMB
MRSA4220	NEG	POS	NEG	POS	NEG	POS	POS	NEG	POS	AMB	AMB	POS	POS	NEG	POS	POS	POS	NEG	NEG	NEG	NEG	NEG
MRSA4221	NEG	POS	NEG	POS	NEG	POS	POS	NEG	POS	AMB	AMB	POS	POS	NEG	POS	POS	POS	NEG	NEG	NEG	NEG	NEG
MRSA4222	NEG	POS	NEG	POS	NEG	POS	POS	NEG	POS	AMB	AMB	POS	POS	NEG	POS	POS	POS	NEG	NEG	NEG	NEG	NEG
MSSA1751	NEG	POS	NEG	POS	NEG	POS	POS	AMB	POS	AMB	POS	AMB	POS	NEG	POS	POS	POS	NEG	NEG	NEG	AMB	AMB
MSSA1760	NEG	POS	NEG	POS	NEG	POS	POS	POS	POS	AMB	POS	AMB	POS	NEG	POS	POS	POS	NEG	NEG	NEG	AMB	AMB
MSSA1802	NEG	POS	NEG	POS	NEG	POS	POS	NEG	POS	AMB	AMB	POS	POS	NEG	POS	POS	POS	NEG	NEG	NEG	NEG	NEG
MSSA1809	NEG	POS	NEG	POS	NEG	POS	POS	NEG	POS	AMB	AMB	POS	POS	NEG	POS	POS	POS	NEG	NEG	NEG	NEG	NEG
MSSA1815	NEG	POS	NEG	POS	NEG	POS	POS	AMB	POS	AMB	AMB	POS	POS	NEG	POS	POS	POS	NEG	NEG	NEG	NEG	NEG
MSSA1829	NEG	POS	NEG	POS	NEG	POS	POS	NEG	POS	AMB	AMB	POS	POS	NEG	POS	POS	POS	NEG	NEG	NEG	NEG	NEG
MSSA1908	NEG	POS	NEG	POS	NEG	POS	POS	NEG	POS	AMB	AMB	POS	POS	NEG	POS	POS	POS	NEG	NEG	NEG	NEG	NEG
MSSA1960	NEG	POS	NEG	POS	NEG	POS	POS	NEG	POS	AMB	AMB	POS	POS	NEG	POS	POS	POS	NEG	NEG	NEG	NEG	NEG
MSSA1965	NEG	POS	NEG	POS	NEG	POS	POS	NEG	POS	AMB	AMB	POS	POS	NEG	POS	POS	POS	NEG	NEG	NEG	NEG	NEG
MSSA1977	NEG	POS	NEG	POS	NEG	POS	POS	NEG	POS	AMB	AMB	POS	POS	NEG	POS	POS	POS	NEG	NEG	NEG	NEG	NEG
MSSA1978	NEG	POS	NEG	POS	NEG	POS	POS	AMB	POS	AMB	AMB	POS	POS	NEG	POS	POS	POS	NEG	NEG	NEG	AMB	AMB
MSSA1999	NEG	POS	NEG	POS	NEG	POS	POS	NEG	POS	AMB	AMB	POS	POS	NEG	POS	POS	POS	NEG	NEG	NEG	NEG	NEG
MSSA2003	NEG	POS	NEG	POS	NEG	POS	POS	NEG	POS	AMB	AMB	POS	POS	NEG	POS	POS	POS	NEG	NEG	NEG	NEG	NEG
MSSA2006	NEG	POS	NEG	POS	NEG	POS	POS	NEG	POS	AMB	AMB	POS	POS	NEG	POS	POS	POS	NEG	NEG	NEG	NEG	NEG
MSSA2008	NEG	POS	NEG	POS	NEG	POS	POS	NEG	POS	AMB	AMB	POS	POS	NEG	POS	POS	POS	NEG	NEG	NEG	NEG	NEG
MSSA2011	NEG	POS	NEG	POS	NEG	POS	POS	NEG	POS	AMB	AMB	POS	POS	NEG	POS	POS	POS	NEG	NEG	NEG	NEG	NEG
MSSA2017	NEG	POS	NEG	POS	NEG	POS	POS	NEG	POS	AMB	AMB	POS	POS	NEG	POS	POS	POS	NEG	NEG	NEG	NEG	NEG
MSSA2022	NEG	POS	NEG	POS	NEG	POS	POS	NEG	POS	AMB	AMB	POS	POS	NEG	POS	POS	POS	NEG	NEG	NEG	NEG	NEG
MSSA2055	NEG	POS	NEG	POS	NEG	POS	POS	NEG	POS	AMB	AMB	POS	POS	NEG	POS	POS	POS	NEG	NEG	NEG	NEG	NEG
MSSA2060	NEG	POS	NEG	POS	NEG	POS	POS	NEG	POS	AMB	AMB	POS	POS	NEG	POS	POS	POS	NEG	NEG	NEG	NEG	NEG
MSSA2080	NEG	POS	NEG	POS	NEG	POS	POS	NEG	POS	AMB	NEG	POS	POS	NEG	POS	POS	POS	NEG	NEG	NEG	NEG	NEG
MSSA2082	NEG	POS	NEG	POS	NEG	POS	POS	NEG	POS	AMB	AMB	POS	POS	NEG	POS	POS	POS	NEG	NEG	NEG	AMB	AMB
MSSA2084	NEG	POS	NEG	POS	NEG	POS	POS	NEG	POS	AMB	NEG	POS	POS	NEG	POS	POS	POS	NEG	NEG	NEG	NEG	NEG
MSSA2087	NEG	POS	NEG	POS	NEG	POS	POS	NEG	POS	AMB	AMB	POS	POS	NEG	POS	POS	POS	NEG	NEG	NEG	NEG	NEG
MSSA2094	NEG	POS	NEG	POS	NEG	POS	POS	NEG	POS	AMB	AMB	POS	POS	NEG	POS	POS	POS	NEG	NEG	NEG	NEG	NEG
MSSA2097	NEG	POS	NEG	POS	NEG	POS	POS	NEG	POS	AMB	AMB	POS	POS	NEG	POS	POS	POS	NEG	NEG	NEG	NEG	NEG
MSSA2110	NEG	POS	NEG	POS	NEG	POS	POS	NEG	POS	AMB	AMB	POS	POS	NEG	POS	POS	POS	NEG	NEG	NEG	NEG	NEG
MSSA2113	NEG	POS	NEG	POS	NEG	POS	POS	NEG	POS	AMB	AMB	POS	POS	NEG	POS	POS	POS	NEG	NEG	NEG	NEG	NEG
MSSA2132	NEG	POS	NEG	POS	NEG	POS	POS	NEG	POS	AMB	AMB	POS	POS	NEG	POS	POS	POS	NEG	NEG	NEG	NEG	NEG
MSSA2134	NEG	POS	NEG	POS	NEG	POS	POS	NEG	POS	AMB	AMB	POS	POS	NEG	POS	POS	POS	NEG	NEG	NEG	NEG	NEG
MSSA1746	NEG	POS	NEG	NEG	NEG	POS	POS	NEG	POS	AMB	POS	AMB	POS	NEG	POS	POS	POS	NEG	NEG	NEG	NEG	NEG
MSSA1757	NEG	POS	NEG	NEG	NEG	POS	POS	NEG	POS	AMB	POS	AMB	POS	NEG	POS	POS	POS	NEG	NEG	NEG	AMB	AMB
MSSA1772	NEG	POS	NEG	NEG	NEG	POS	POS	NEG	POS	AMB	POS	AMB	POS	NEG	POS	POS	POS	NEG	NEG	NEG	NEG	NEG
MSSA1795	NEG	POS	NEG	NEG	NEG	POS	POS	NEG	POS	AMB	POS	AMB	POS	NEG	POS	POS	POS	NEG	NEG	NEG	AMB	AMB
MSSA1813	NEG	POS	NEG	NEG	NEG	POS	POS	NEG	POS	AMB	POS	AMB	POS	NEG	POS	POS	POS	NEG	NEG	NEG	NEG	NEG
MSSA1826	NEG	POS	NEG	NEG	NEG	POS	POS	NEG	POS	AMB	POS	AMB	POS	NEG	POS	POS	POS	NEG	NEG	NEG	NEG	NEG
MSSA1830	NEG	POS	NEG	NEG	NEG	POS	POS	NEG	POS	AMB	POS	AMB	POS	NEG	POS	POS	POS	NEG	NEG	NEG	NEG	NEG
MSSA1838	NEG	POS	NEG	NEG	NEG	POS	POS	NEG	POS	AMB	POS	AMB	POS	NEG	POS	POS	POS	NEG	NEG	NEG	NEG	NEG
MSSA1944	NEG	POS	NEG	NEG	NEG	POS	POS	NEG	POS	AMB	POS	AMB	POS	NEG	POS	POS	POS	NEG	NEG	NEG	NEG	NEG
MSSA1947	NEG	POS	NEG	NEG	NEG	POS	POS	NEG	POS	AMB	POS	AMB	POS	NEG	POS	POS	POS	NEG	NEG	NEG	NEG	NEG
MSSA1964	NEG	POS	NEG	NEG	NEG	POS	POS	NEG	POS	AMB	POS	AMB	POS	NEG	POS	POS	POS	NEG	NEG	NEG	NEG	NEG
MSSA1969	NEG	POS	NEG	NEG	NEG	POS	POS	NEG	POS	AMB	POS	AMB	POS	NEG	POS	POS	POS	NEG	NEG	NEG	NEG	NEG
MSSA2001	NEG	POS	NEG	NEG	NEG	POS	POS	NEG	POS	AMB	POS	AMB	POS	NEG	POS	POS	POS	NEG	NEG	NEG	AMB	AMB
MSSA2004	NEG	POS	NEG	NEG	NEG	POS	POS	NEG	POS	AMB	POS	AMB	POS	NEG	POS	POS	POS	NEG	NEG	NEG	AMB	AMB
MSSA2024	NEG	POS	NEG	NEG	NEG	POS	POS	NEG	POS	AMB	POS	NEG	POS	NEG	POS	POS	POS	NEG	NEG	NEG	NEG	NEG
MSSA2025	NEG	POS	NEG	NEG	NEG	POS	POS	NEG	POS	AMB	POS	AMB	POS	NEG	POS	POS	POS	NEG	NEG	NEG	NEG	NEG
MSSA2026	NEG	POS	NEG	NEG	NEG	POS	POS	NEG	POS	AMB	POS	AMB	POS	NEG	POS	POS	POS	NEG	NEG	NEG	NEG	NEG
MSSA2032	NEG	POS	NEG	NEG	NEG	POS	POS	NEG	POS	AMB	POS	AMB	POS	NEG	POS	POS	POS	NEG	NEG	NEG	NEG	NEG
MSSA2043	NEG	POS	NEG	NEG	NEG	POS	POS	NEG	POS	AMB	POS	AMB	POS	NEG	POS	POS	POS	NEG	NEG	NEG	NEG	NEG
MSSA2061	NEG	POS	NEG	NEG	NEG	POS	POS	NEG	POS	AMB	POS	AMB	POS	NEG	POS	POS	POS	NEG	NEG	NEG	NEG	NEG
MSSA2085	NEG	POS	NEG	NEG	NEG	POS	POS	NEG	POS	AMB	POS	AMB	POS	NEG	POS	POS	POS	NEG	NEG	NEG	NEG	NEG
MSSA2098	NEG	POS	NEG	NEG	NEG	POS	POS	NEG	POS	AMB	POS	AMB	POS	NEG	POS	POS	POS	NEG	NEG	NEG	NEG	NEG
MSSA2100	NEG	POS	NEG	NEG	NEG	POS	POS	NEG	POS	AMB	POS	AMB	POS	NEG	POS	POS	POS	NEG	NEG	NEG	NEG	NEG
MSSA2111	NEG	POS	NEG	NEG	NEG	POS	POS	NEG	POS	AMB	POS	AMB	POS	NEG	POS	POS	POS	NEG	NEG	NEG	AMB	AMB
MSSA2178	NEG	POS	NEG	NEG	NEG	POS	POS	NEG	POS	AMB	POS	AMB	POS	NEG	POS	POS	POS	NEG	NEG	NEG	NEG	NEG
MSSA2202	NEG	POS	NEG	NEG	NEG	POS	POS	NEG	POS	AMB	POS	AMB	POS	NEG	POS	POS	POS	NEG	NEG	NEG	NEG	NEG
MSSA2205	NEG	POS	NEG	NEG	NEG	POS	POS	NEG	POS	AMB	POS	AMB	POS	NEG	POS	POS	POS	NEG	NEG	NEG	NEG	NEG
MRSA3796	NEG	POS	POS	POS	POS	NEG	NEG	POS	POS	AMB	POS	AMB	POS	NEG	POS	POS	POS	NEG	NEG	POS	AMB	AMB
MRSA3805	NEG	POS	POS	POS	POS	NEG	NEG	AMB	NEG	POS	POS	AMB	POS	NEG	POS	POS	POS	NEG	NEG	POS	NEG	NEG
MRSA3965	NEG	POS	POS	POS	POS	NEG	NEG	NEG	POS	AMB	POS	AMB	POS	NEG	POS	POS	POS	NEG	NEG	POS	NEG	NEG
MSSA1761	NEG	POS	POS	POS	POS	NEG	NEG	NEG	POS	AMB	POS	AMB	POS	AMB	POS	POS	POS	NEG	NEG	POS	POS	POS
MSSA1764	NEG	POS	POS	POS	POS	NEG	NEG	NEG	POS	AMB	POS	AMB	POS	NEG	POS	POS	POS	NEG	NEG	POS	NEG	NEG
MSSA1765	NEG	POS	POS	POS	POS	NEG	NEG	NEG	POS	AMB	POS	AMB	POS	NEG	POS	POS	POS	NEG	NEG	POS	NEG	NEG
MSSA1784	NEG	POS	POS	POS	POS	NEG	NEG	AMB	POS	AMB	POS	AMB	POS	NEG	POS	POS	POS	NEG	NEG	POS	AMB	AMB
MSSA1806	NEG	POS	POS	POS	POS	NEG	NEG	NEG	POS	AMB	POS	AMB	POS	NEG	POS	POS	POS	NEG	NEG	POS	NEG	NEG
MSSA1807	NEG	POS	POS	POS	POS	NEG	NEG	AMB	POS	AMB	POS	AMB	POS	NEG	POS	POS	POS	NEG	NEG	POS	NEG	NEG
MSSA1888	NEG	POS	POS	POS	POS	NEG	NEG	NEG	POS	AMB	POS	AMB	POS	NEG	POS	POS	POS	NEG	NEG	POS	NEG	NEG
MSSA1894	NEG	POS	POS	POS	POS	NEG	NEG	NEG	POS	AMB	POS	NEG	POS	NEG	POS	POS	POS	NEG	NEG	POS	NEG	NEG
MSSA1922	NEG	POS	POS	POS	POS	NEG	NEG	AMB	POS	AMB	POS	AMB	POS	NEG	POS	POS	POS	NEG	NEG	POS	NEG	NEG
MSSA1950	NEG	POS	POS	POS	POS	NEG	NEG	NEG	POS	AMB	POS	AMB	POS	NEG	POS	POS	POS	NEG	NEG	POS	NEG	NEG
MSSA1954	NEG	POS	POS	POS	POS	NEG	NEG	NEG	POS	AMB	POS	AMB	POS	NEG	POS	POS	POS	NEG	NEG	POS	NEG	NEG
MSSA1966	NEG	POS	POS	POS	POS	NEG	NEG	AMB	POS	AMB	POS	AMB	POS	NEG	POS	POS	POS	NEG	NEG	POS	NEG	NEG
MSSA1972	NEG	POS	POS	POS	POS	NEG	NEG	NEG	POS	AMB	POS	AMB	POS	NEG	POS	POS	POS	NEG	NEG	POS	NEG	NEG
MSSA2010	NEG	POS	POS	POS	POS	NEG	NEG	NEG	POS	AMB	POS	AMB	POS	NEG	POS	POS	POS	NEG	NEG	POS	NEG	NEG
MSSA2020	NEG	POS	POS	POS	POS	NEG	NEG	NEG	POS	AMB	POS	AMB	POS	NEG	POS	POS	POS	NEG	NEG	AMB	NEG	NEG
MSSA2028	NEG	POS	POS	POS	POS	NEG	NEG	AMB	POS	AMB	POS	AMB	POS	NEG	POS	POS	POS	NEG	NEG	POS	AMB	AMB
MSSA2088	NEG	POS	POS	POS	POS	NEG	NEG	AMB	POS	AMB	POS	AMB	POS	NEG	POS	POS	POS	NEG	NEG	POS	NEG	NEG
MSSA2187	NEG	POS	POS	POS	POS	NEG	NEG	NEG	POS	AMB	POS	AMB	POS	NEG	POS	POS	POS	NEG	NEG	POS	NEG	NEG
MSSA2204	NEG	POS	POS	POS	POS	NEG	NEG	NEG	POS	AMB	POS	AMB	POS	NEG	POS	POS	POS	NEG	NEG	POS	AMB	AMB
MSSA2210	NEG	POS	POS	POS	POS	NEG	NEG	NEG	POS	AMB	POS	AMB	POS	NEG	POS	POS	POS	NEG	NEG	POS	NEG	NEG
MSSA2223	NEG	POS	POS	POS	POS	NEG	NEG	AMB	POS	AMB	POS	AMB	POS	NEG	POS	POS	POS	NEG	NEG	POS	NEG	NEG
MSSA1763	NEG	POS	NEG	NEG	NEG	POS	POS	NEG	POS	AMB	NEG	POS	POS	NEG	POS	POS	POS	NEG	NEG	NEG	NEG	NEG
MSSA1777	NEG	POS	NEG	NEG	NEG	POS	POS	NEG	POS	AMB	POS	AMB	POS	AMB	POS	POS	POS	NEG	NEG	NEG	POS	POS
MSSA2009	NEG	POS	NEG	NEG	NEG	POS	POS	NEG	POS	AMB	AMB	POS	POS	NEG	POS	POS	POS	NEG	NEG	NEG	NEG	NEG
MSSA2047	NEG	POS	NEG	NEG	NEG	POS	POS	NEG	POS	AMB	AMB	POS	POS	NEG	POS	POS	POS	NEG	NEG	NEG	NEG	NEG
MSSA2079	NEG	POS	NEG	NEG	NEG	POS	POS	NEG	POS	AMB	AMB	POS	POS	NEG	POS	POS	POS	NEG	NEG	NEG	NEG	NEG
MSSA2101	NEG	POS	NEG	NEG	NEG	POS	POS	NEG	POS	AMB	AMB	POS	POS	NEG	POS	POS	POS	NEG	NEG	NEG	NEG	NEG
MSSA1821	NEG	POS	NEG	NEG	NEG	POS	POS	NEG	POS	AMB	POS	AMB	POS	NEG	POS	POS	POS	NEG	NEG	NEG	NEG	NEG
MSSA1898	AMB	POS	NEG	NEG	NEG	POS	POS	NEG	POS	AMB	POS	AMB	POS	NEG	POS	POS	POS	NEG	NEG	NEG	NEG	NEG
MSSA1973	NEG	POS	NEG	NEG	NEG	POS	POS	NEG	POS	AMB	POS	AMB	POS	NEG	POS	POS	POS	NEG	NEG	NEG	NEG	NEG
MSSA2071	NEG	POS	NEG	NEG	NEG	POS	POS	NEG	POS	AMB	POS	AMB	POS	NEG	POS	POS	POS	NEG	NEG	NEG	NEG	NEG
MSSA2083	NEG	POS	NEG	NEG	NEG	POS	POS	NEG	POS	AMB	POS	AMB	POS	NEG	POS	POS	POS	NEG	NEG	NEG	NEG	NEG
MSSA2106	NEG	POS	NEG	NEG	NEG	POS	POS	NEG	POS	AMB	POS	AMB	POS	NEG	POS	POS	POS	NEG	NEG	NEG	NEG	NEG
MSSA2112	NEG	POS	NEG	NEG	NEG	POS	POS	NEG	POS	AMB	POS	AMB	POS	NEG	POS	POS	POS	NEG	NEG	NEG	AMB	AMB
MSSA2115	NEG	POS	NEG	NEG	NEG	POS	POS	NEG	POS	AMB	POS	AMB	POS	NEG	POS	POS	POS	NEG	NEG	NEG	AMB	AMB
MSSA2195	NEG	POS	NEG	NEG	NEG	POS	POS	NEG	POS	AMB	POS	AMB	POS	NEG	POS	POS	POS	NEG	NEG	NEG	AMB	AMB
MSSA2207	NEG	POS	NEG	NEG	NEG	POS	POS	NEG	POS	AMB	POS	AMB	POS	AMB	POS	POS	POS	NEG	NEG	NEG	AMB	AMB
MSSA1758	NEG	POS	POS	POS	POS	POS	POS	NEG	POS	AMB	POS	AMB	POS	POS	NEG	POS	POS	NEG	NEG	NEG	NEG	NEG
MSSA1762	NEG	POS	POS	POS	POS	POS	POS	POS	POS	AMB	POS	AMB	POS	POS	POS	POS	POS	NEG	NEG	NEG	AMB	AMB
MSSA1769	NEG	POS	POS	POS	POS	POS	POS	AMB	POS	AMB	POS	AMB	POS	POS	NEG	POS	POS	NEG	NEG	NEG	AMB	AMB
MSSA1771	NEG	POS	POS	POS	POS	POS	POS	NEG	POS	AMB	POS	AMB	POS	POS	NEG	POS	POS	NEG	NEG	NEG	NEG	NEG
MSSA1789	NEG	POS	POS	POS	POS	POS	POS	NEG	POS	AMB	POS	AMB	POS	POS	NEG	POS	POS	NEG	NEG	NEG	NEG	NEG
MSSA1800	NEG	POS	POS	POS	POS	POS	POS	NEG	POS	AMB	POS	AMB	POS	POS	NEG	POS	POS	NEG	NEG	NEG	NEG	NEG
MSSA1833	NEG	POS	POS	POS	POS	POS	POS	AMB	POS	AMB	POS	AMB	POS	POS	NEG	POS	POS	NEG	NEG	NEG	NEG	NEG
MSSA1890	NEG	POS	POS	POS	POS	POS	POS	NEG	POS	AMB	POS	AMB	POS	POS	NEG	POS	POS	NEG	NEG	NEG	NEG	NEG
MSSA1903	NEG	POS	POS	POS	POS	POS	POS	AMB	POS	AMB	POS	AMB	POS	POS	NEG	POS	POS	NEG	NEG	NEG	NEG	NEG
MSSA1940	NEG	POS	POS	POS	POS	POS	POS	NEG	POS	AMB	POS	AMB	POS	POS	NEG	POS	POS	NEG	NEG	NEG	AMB	AMB
MSSA1962	NEG	POS	POS	POS	POS	POS	POS	NEG	POS	AMB	POS	AMB	POS	POS	NEG	POS	POS	NEG	NEG	NEG	NEG	NEG
MSSA1963	NEG	POS	POS	POS	POS	POS	POS	AMB	POS	AMB	POS	AMB	POS	POS	NEG	POS	POS	NEG	NEG	NEG	AMB	AMB
MSSA2016	NEG	POS	POS	POS	POS	POS	POS	NEG	POS	AMB	POS	AMB	POS	POS	NEG	POS	POS	NEG	NEG	NEG	NEG	NEG
MSSA2018	NEG	POS	POS	POS	POS	POS	POS	NEG	POS	AMB	POS	AMB	POS	POS	NEG	POS	POS	NEG	NEG	NEG	NEG	NEG
MSSA2019	NEG	POS	POS	POS	POS	POS	POS	NEG	POS	AMB	POS	AMB	POS	NEG	POS	POS	POS	NEG	NEG	NEG	NEG	NEG
MSSA2029	NEG	POS	POS	POS	POS	POS	POS	AMB	POS	AMB	POS	AMB	POS	POS	NEG	POS	POS	NEG	NEG	NEG	NEG	NEG
MSSA2045	NEG	POS	POS	POS	POS	POS	POS	NEG	POS	AMB	POS	AMB	POS	POS	NEG	POS	POS	NEG	NEG	NEG	NEG	NEG
MSSA2048	NEG	POS	POS	POS	POS	POS	POS	NEG	POS	AMB	POS	AMB	POS	POS	NEG	POS	POS	NEG	NEG	NEG	NEG	NEG
MSSA2049	NEG	POS	POS	POS	POS	POS	POS	NEG	POS	AMB	POS	AMB	POS	NEG	POS	POS	POS	NEG	NEG	NEG	NEG	NEG
MSSA2050	NEG	POS	POS	POS	POS	POS	POS	AMB	POS	AMB	POS	AMB	POS	NEG	POS	POS	POS	NEG	NEG	NEG	AMB	AMB
MSSA2099	NEG	POS	POS	POS	POS	POS	AMB	NEG	POS	AMB	POS	NEG	POS	POS	NEG	POS	POS	NEG	NEG	NEG	NEG	NEG
MSSA2109	NEG	POS	POS	POS	POS	POS	POS	NEG	POS	AMB	POS	AMB	POS	POS	NEG	POS	POS	NEG	NEG	NEG	NEG	NEG
MSSA2130	NEG	POS	POS	POS	POS	POS	POS	AMB	POS	AMB	POS	AMB	POS	POS	NEG	POS	POS	NEG	NEG	NEG	AMB	AMB
MSSA2133	NEG	POS	POS	POS	POS	POS	POS	NEG	POS	AMB	POS	AMB	POS	POS	NEG	POS	POS	NEG	NEG	NEG	POS	POS
MSSA2139	NEG	POS	POS	POS	POS	POS	POS	NEG	POS	AMB	POS	AMB	POS	POS	NEG	POS	POS	NEG	NEG	NEG	NEG	NEG
MSSA2141	NEG	POS	POS	POS	POS	POS	POS	NEG	POS	AMB	POS	AMB	POS	POS	NEG	POS	POS	NEG	NEG	NEG	AMB	AMB
MSSA2208	NEG	POS	POS	POS	POS	POS	POS	NEG	POS	AMB	POS	AMB	POS	NEG	POS	POS	POS	NEG	NEG	NEG	NEG	NEG
MSSA2209	NEG	POS	POS	POS	POS	POS	POS	NEG	POS	AMB	POS	AMB	POS	POS	NEG	POS	POS	NEG	NEG	NEG	NEG	NEG
MSSA1893	NEG	POS	NEG	NEG	NEG	POS	POS	NEG	POS	AMB	POS	AMB	POS	NEG	POS	POS	POS	NEG	NEG	NEG	NEG	NEG
MSSA2105	NEG	POS	NEG	NEG	NEG	POS	POS	NEG	POS	AMB	POS	AMB	POS	NEG	POS	POS	POS	NEG	NEG	NEG	NEG	NEG
MRSA3836	NEG	POS	NEG	AMB	NEG	POS	POS	POS	NEG	POS	AMB	POS	POS	NEG	POS	POS	POS	NEG	NEG	NEG	NEG	NEG
MRSA3840	NEG	POS	NEG	POS	NEG	POS	POS	POS	NEG	POS	AMB	POS	POS	NEG	POS	POS	POS	NEG	NEG	NEG	NEG	NEG
MRSA4188	NEG	POS	NEG	POS	NEG	POS	POS	POS	NEG	POS	POS	AMB	POS	AMB	POS	POS	POS	NEG	NEG	NEG	AMB	AMB
MSSA1814	NEG	POS	NEG	AMB	NEG	POS	POS	POS	NEG	POS	AMB	POS	POS	NEG	POS	POS	POS	NEG	NEG	NEG	NEG	NEG
MSSA1816	NEG	POS	NEG	NEG	NEG	POS	POS	NEG	NEG	POS	NEG	POS	POS	NEG	POS	POS	POS	NEG	NEG	NEG	NEG	NEG
MSSA1825	NEG	POS	NEG	POS	NEG	POS	POS	POS	NEG	POS	AMB	POS	POS	NEG	POS	POS	POS	NEG	NEG	NEG	NEG	NEG
MSSA1834	NEG	POS	NEG	NEG	NEG	POS	POS	NEG	NEG	POS	NEG	POS	POS	NEG	POS	POS	POS	NEG	NEG	NEG	NEG	NEG
MSSA1938	NEG	POS	NEG	AMB	NEG	POS	POS	AMB	NEG	POS	AMB	POS	POS	NEG	POS	POS	POS	NEG	NEG	NEG	NEG	NEG
MSSA1948	NEG	POS	NEG	AMB	NEG	POS	POS	AMB	NEG	POS	AMB	POS	POS	NEG	POS	POS	POS	NEG	NEG	NEG	NEG	NEG
MSSA1951	NEG	POS	NEG	NEG	NEG	POS	POS	NEG	NEG	POS	AMB	POS	POS	NEG	POS	POS	POS	NEG	NEG	NEG	NEG	NEG
MSSA2005	NEG	POS	NEG	AMB	NEG	POS	POS	POS	NEG	POS	AMB	POS	POS	NEG	POS	POS	POS	NEG	NEG	NEG	NEG	NEG
MSSA2051	NEG	POS	NEG	POS	NEG	POS	POS	POS	NEG	POS	AMB	POS	POS	NEG	POS	POS	POS	NEG	NEG	NEG	NEG	NEG
MSSA2056	NEG	POS	NEG	AMB	NEG	POS	POS	POS	NEG	POS	AMB	POS	POS	NEG	POS	POS	POS	NEG	NEG	NEG	NEG	NEG
MSSA2066	NEG	POS	NEG	POS	NEG	POS	POS	POS	NEG	POS	AMB	POS	POS	NEG	POS	POS	POS	NEG	NEG	NEG	NEG	NEG
MSSA2067	NEG	POS	NEG	AMB	NEG	POS	POS	AMB	NEG	POS	AMB	POS	POS	NEG	POS	POS	POS	NEG	NEG	NEG	NEG	NEG
MSSA2086	NEG	POS	NEG	AMB	NEG	POS	POS	AMB	NEG	POS	AMB	POS	POS	NEG	POS	POS	POS	NEG	NEG	NEG	NEG	NEG
MSSA2089	NEG	POS	NEG	AMB	NEG	POS	POS	AMB	NEG	POS	NEG	POS	POS	NEG	POS	POS	POS	NEG	NEG	NEG	NEG	NEG
MSSA2103	NEG	POS	NEG	AMB	NEG	POS	POS	AMB	NEG	POS	AMB	POS	POS	NEG	POS	POS	POS	NEG	NEG	NEG	NEG	NEG
MSSA2173	NEG	POS	NEG	NEG	NEG	POS	POS	AMB	NEG	POS	AMB	POS	POS	NEG	POS	POS	POS	NEG	NEG	NEG	NEG	NEG
MSSA2183	NEG	POS	AMB	POS	NEG	POS	POS	POS	NEG	POS	POS	AMB	POS	NEG	POS	POS	POS	NEG	NEG	NEG	NEG	NEG
MSSA2196	NEG	POS	NEG	AMB	NEG	POS	POS	POS	NEG	POS	POS	AMB	POS	NEG	POS	POS	POS	NEG	NEG	NEG	NEG	NEG
MSSA2219	NEG	POS	NEG	NEG	NEG	POS	POS	NEG	NEG	POS	POS	AMB	POS	NEG	POS	POS	POS	NEG	NEG	NEG	AMB	AMB
MSSA1753	NEG	POS	NEG	AMB	NEG	POS	POS	NEG	POS	AMB	AMB	POS	POS	NEG	POS	POS	POS	NEG	NEG	POS	NEG	NEG
MSSA1773	NEG	POS	NEG	AMB	NEG	POS	POS	NEG	POS	AMB	AMB	POS	POS	NEG	POS	POS	POS	NEG	NEG	POS	NEG	NEG
MSSA1811	NEG	POS	NEG	POS	NEG	POS	POS	NEG	POS	AMB	AMB	POS	POS	NEG	POS	POS	POS	NEG	NEG	POS	NEG	NEG
MSSA1897	NEG	POS	NEG	POS	NEG	POS	POS	NEG	POS	AMB	AMB	POS	POS	NEG	POS	POS	POS	NEG	NEG	POS	NEG	NEG
MSSA1931	NEG	POS	NEG	POS	NEG	POS	POS	NEG	POS	AMB	AMB	POS	POS	NEG	POS	POS	POS	NEG	NEG	POS	NEG	NEG
MSSA2114	NEG	POS	NEG	POS	NEG	POS	POS	NEG	POS	AMB	AMB	POS	POS	NEG	POS	POS	POS	NEG	NEG	POS	NEG	NEG
MSSA1745	POS	POS	POS	NEG	POS	POS	POS	POS	AMB	POS	POS	AMB	POS	POS	POS	POS	POS	NEG	NEG	NEG	AMB	AMB
MSSA1747	POS	POS	POS	NEG	POS	POS	POS	POS	NEG	POS	AMB	POS	POS	POS	NEG	POS	POS	NEG	NEG	NEG	NEG	NEG
MSSA1749	POS	POS	POS	NEG	POS	POS	POS	POS	NEG	POS	AMB	POS	POS	POS	NEG	POS	POS	NEG	NEG	NEG	NEG	NEG
MSSA1768	POS	POS	POS	NEG	POS	POS	POS	POS	NEG	POS	AMB	POS	POS	POS	NEG	POS	POS	NEG	NEG	NEG	NEG	NEG
MSSA1774	POS	POS	POS	NEG	POS	POS	POS	POS	NEG	POS	AMB	POS	POS	POS	NEG	POS	POS	NEG	NEG	NEG	NEG	NEG
MSSA1775	POS	POS	POS	NEG	POS	POS	POS	POS	NEG	POS	AMB	POS	POS	POS	NEG	POS	POS	NEG	NEG	NEG	AMB	AMB
MSSA1776	POS	POS	POS	NEG	POS	POS	POS	POS	NEG	POS	POS	AMB	POS	POS	NEG	POS	POS	NEG	NEG	NEG	AMB	AMB
MSSA1779	POS	POS	POS	NEG	POS	POS	POS	POS	NEG	POS	NEG	POS	POS	POS	NEG	POS	POS	NEG	NEG	NEG	NEG	NEG
MSSA1780	POS	POS	POS	NEG	POS	POS	POS	POS	NEG	POS	AMB	POS	POS	POS	NEG	POS	POS	NEG	NEG	NEG	NEG	NEG

	HYALURONATE LYASE								IMMUNODOMI- NANT ANTIGEN B		DEFENSIN RESISTANCE		TRANSFERRIN BINDING PROTEIN			PUTATIVE TRANSPORTER				MISCELLANEOUS GENES		
Strain	hysA1 (MRSA252)	hysA1 (MRSA252+RF122 and/or hysA2 (cons)	hysA1 (MRSA252+RF122) and/or hysA2 (COL+USA300)	hysA2 (Other than MRSA252)	hysA2 (COL+ USA300+ NCTC8325)	hysA2 (Other than COL+ USA300+ NCTC8325) (probe 1)	hysA2 (Other than COL+ USA300+ NCTC8325) (probe 2)	hysA2 (MRSA252)	isaB	isaB (MRSA252)	mprF (COL+MW2)	mprF (Mu50+252)	isdA	isdA (MRSA252)	isdA (Other than MRSA252)	lmrP (Other than RF122) (probe 1)	lmrP (Other than RF122) (probe 2)	lmrP (RF122) (probe 1)	lmrP (RF122) (probe 2)	Q2FXC0	Q2YUB3	Q7A4X2
MSSA1781	POS	POS	POS	NEG	POS	POS	POS	POS	NEG	POS	AMB	POS	POS	POS	NEG	POS	POS	NEG	NEG	NEG	AMB	AMB
MSSA1786	POS	POS	POS	NEG	POS	POS	POS	POS	NEG	POS	AMB	POS	POS	POS	NEG	POS	POS	NEG	NEG	NEG	AMB	AMB
MSSA1793	POS	POS	POS	NEG	POS	POS	POS	POS	NEG	POS	NEG	POS	POS	POS	NEG	POS	POS	NEG	NEG	NEG	NEG	NEG
MSSA1803	POS	POS	POS	NEG	POS	POS	POS	POS	NEG	POS	AMB	POS	POS	POS	NEG	POS	POS	NEG	NEG	NEG	NEG	NEG
MSSA1836	POS	POS	POS	NEG	POS	POS	POS	POS	NEG	POS	AMB	POS	POS	POS	NEG	POS	POS	NEG	NEG	NEG	NEG	NEG
MSSA1839	POS	POS	POS	NEG	POS	POS	POS	POS	NEG	POS	AMB	POS	POS	POS	NEG	POS	POS	NEG	NEG	NEG	NEG	NEG
MSSA1900	POS	POS	POS	NEG	POS	POS	POS	POS	NEG	POS	AMB	POS	POS	POS	NEG	POS	POS	NEG	NEG	NEG	NEG	NEG
MSSA1910	POS	POS	POS	NEG	POS	POS	POS	POS	NEG	POS	AMB	POS	POS	POS	NEG	POS	POS	NEG	NEG	NEG	NEG	NEG
MSSA1918	POS	POS	POS	NEG	POS	POS	POS	POS	NEG	POS	AMB	POS	POS	POS	NEG	POS	POS	NEG	NEG	NEG	NEG	NEG
MSSA1921	POS	POS	POS	NEG	POS	POS	POS	POS	NEG	POS	AMB	POS	POS	POS	NEG	POS	POS	NEG	NEG	NEG	NEG	NEG
MSSA1923	POS	POS	POS	NEG	POS	POS	POS	POS	NEG	POS	AMB	POS	POS	POS	NEG	POS	POS	NEG	NEG	NEG	NEG	NEG
MSSA1981	POS	POS	POS	NEG	POS	POS	POS	POS	NEG	POS	AMB	POS	POS	POS	NEG	POS	POS	NEG	NEG	NEG	NEG	NEG
MSSA1998	POS	POS	POS	NEG	POS	POS	POS	POS	NEG	POS	AMB	POS	POS	POS	NEG	POS	POS	NEG	NEG	NEG	NEG	NEG
MSSA2000	POS	POS	POS	NEG	POS	POS	POS	POS	NEG	POS	AMB	POS	POS	POS	NEG	POS	POS	NEG	NEG	NEG	NEG	NEG
MSSA2007	POS	POS	POS	NEG	POS	POS	POS	POS	NEG	POS	AMB	POS	POS	POS	NEG	POS	POS	NEG	NEG	NEG	AMB	AMB
MSSA2012	POS	POS	POS	NEG	POS	POS	POS	POS	NEG	POS	AMB	POS	POS	POS	NEG	POS	POS	NEG	NEG	NEG	AMB	AMB
MSSA2015	POS	POS	POS	NEG	POS	POS	POS	POS	NEG	POS	NEG	POS	POS	POS	NEG	POS	POS	NEG	NEG	NEG	NEG	NEG
MSSA2021	POS	POS	POS	NEG	POS	POS	POS	POS	NEG	POS	NEG	POS	POS	POS	NEG	POS	POS	NEG	NEG	NEG	NEG	NEG
MSSA2027	POS	POS	POS	NEG	POS	POS	POS	POS	NEG	POS	AMB	POS	POS	POS	NEG	POS	POS	NEG	NEG	NEG	NEG	NEG
MSSA2063	POS	POS	POS	NEG	POS	POS	POS	POS	NEG	POS	AMB	POS	POS	POS	NEG	POS	POS	NEG	NEG	NEG	NEG	NEG
MSSA2064	POS	POS	POS	NEG	POS	POS	POS	POS	NEG	POS	AMB	POS	POS	POS	NEG	POS	POS	NEG	NEG	NEG	NEG	NEG
MSSA2065	POS	POS	POS	NEG	POS	POS	POS	POS	NEG	POS	AMB	POS	POS	POS	NEG	POS	POS	NEG	NEG	NEG	NEG	NEG
MSSA2075	POS	POS	POS	NEG	POS	POS	POS	POS	NEG	POS	AMB	POS	POS	POS	NEG	POS	POS	NEG	NEG	NEG	NEG	NEG
MSSA2093	POS	POS	POS	NEG	POS	POS	POS	POS	NEG	POS	AMB	POS	POS	POS	NEG	POS	POS	NEG	NEG	NEG	NEG	NEG
MSSA2102	POS	POS	POS	NEG	POS	POS	POS	POS	NEG	POS	AMB	POS	POS	POS	NEG	POS	POS	NEG	NEG	NEG	NEG	NEG
MSSA2138	POS	POS	POS	NEG	POS	POS	POS	POS	NEG	NEG	AMB	POS	POS	POS	NEG	POS	POS	NEG	NEG	NEG	NEG	NEG
MSSA2168	POS	POS	POS	NEG	POS	POS	POS	POS	NEG	POS	AMB	POS	POS	POS	NEG	POS	POS	NEG	NEG	NEG	NEG	NEG
MSSA2181	POS	POS	POS	NEG	POS	POS	POS	POS	NEG	POS	AMB	POS	POS	POS	NEG	POS	POS	NEG	NEG	NEG	NEG	NEG
MSSA2189	POS	POS	POS	NEG	POS	POS	POS	POS	NEG	POS	AMB	POS	POS	POS	NEG	POS	POS	NEG	NEG	NEG	AMB	AMB
MSSA2214	POS	POS	POS	NEG	POS	POS	POS	POS	NEG	POS	AMB	POS	POS	POS	NEG	POS	POS	NEG	NEG	NEG	NEG	NEG
MRSA3803	NEG	POS	NEG	POS	NEG	POS	NEG	NEG	NEG	POS	AMB	POS	POS	POS	POS	POS	POS	NEG	NEG	NEG	AMB	AMB
MSSA1750	NEG	POS	NEG	AMB	NEG	POS	NEG	NEG	AMB	POS	AMB	POS	POS	AMB	POS	POS	POS	AMB	NEG	NEG	POS	POS
MSSA1756	NEG	POS	NEG	AMB	NEG	POS	NEG	NEG	AMB	POS	AMB	POS	POS	POS	POS	POS	POS	NEG	NEG	NEG	AMB	AMB
MSSA1759	NEG	POS	NEG	NEG	NEG	POS	NEG	NEG	NEG	POS	NEG	POS	POS	POS	POS	POS	POS	NEG	NEG	NEG	AMB	AMB
MSSA1767	NEG	POS	NEG	AMB	NEG	POS	NEG	NEG	NEG	POS	NEG	POS	POS	POS	POS	POS	POS	NEG	NEG	NEG	AMB	AMB
MSSA1787	NEG	POS	NEG	AMB	NEG	POS	NEG	NEG	NEG	POS	NEG	POS	POS	POS	POS	POS	POS	NEG	NEG	NEG	NEG	NEG
MSSA1790	NEG	POS	NEG	POS	NEG	POS	NEG	NEG	NEG	POS	NEG	POS	POS	POS	POS	POS	POS	NEG	NEG	NEG	AMB	AMB
MSSA1794	NEG	POS	NEG	NEG	NEG	AMB	NEG	NEG	NEG	POS	NEG	NEG	POS	POS	NEG	POS	POS	NEG	NEG	NEG	NEG	NEG
MSSA1808	NEG	POS	NEG	POS	NEG	POS	NEG	NEG	NEG	POS	NEG	NEG	POS	POS	POS	POS	POS	NEG	NEG	NEG	NEG	NEG
MSSA1812	NEG	POS	NEG	AMB	NEG	POS	NEG	NEG	NEG	POS	NEG	NEG	POS	POS	POS	POS	POS	NEG	NEG	NEG	NEG	NEG
MSSA1819	NEG	POS	NEG	AMB	NEG	POS	NEG	NEG	NEG	POS	NEG	NEG	POS	POS	POS	POS	POS	NEG	NEG	NEG	NEG	NEG
MSSA1824	NEG	POS	NEG	NEG	NEG	POS	NEG	NEG	NEG	POS	NEG	NEG	POS	POS	POS	POS	POS	NEG	NEG	NEG	NEG	NEG
MSSA1827	NEG	POS	NEG	NEG	NEG	POS	NEG	NEG	NEG	POS	NEG	NEG	POS	POS	POS	POS	POS	NEG	NEG	NEG	NEG	NEG
MSSA1889	NEG	POS	NEG	NEG	NEG	POS	NEG	NEG	NEG	POS	NEG	NEG	POS	POS	NEG	POS	POS	NEG	NEG	NEG	NEG	NEG
MSSA1895	NEG	POS	NEG	NEG	NEG	POS	NEG	NEG	NEG	POS	NEG	NEG	POS	POS	NEG	POS	POS	NEG	NEG	NEG	NEG	NEG
MSSA1904	NEG	POS	NEG	NEG	NEG	AMB	NEG	NEG	NEG	POS	NEG	NEG	POS	POS	NEG	POS	POS	NEG	NEG	NEG	NEG	NEG
MSSA1905	NEG	POS	NEG	NEG	NEG	AMB	NEG	NEG	NEG	POS	NEG	NEG	POS	POS	NEG	POS	POS	NEG	NEG	NEG	NEG	NEG
MSSA1907	NEG	POS	NEG	NEG	NEG	NEG	NEG	NEG	NEG	POS	NEG	NEG	POS	POS	NEG	POS	POS	NEG	NEG	NEG	NEG	NEG
MSSA1919	NEG	POS	NEG	NEG	NEG	POS	NEG	NEG	NEG	POS	NEG	NEG	POS	POS	NEG	POS	POS	NEG	NEG	NEG	NEG	NEG
MSSA1920	NEG	POS	NEG	NEG	NEG	POS	NEG	NEG	NEG	POS	NEG	NEG	POS	POS	POS	POS	POS	NEG	NEG	NEG	NEG	NEG
MSSA1929	NEG	POS	NEG	NEG	NEG	POS	NEG	NEG	NEG	POS	NEG	NEG	POS	POS	POS	POS	POS	NEG	NEG	NEG	NEG	NEG
MSSA1930	NEG	POS	NEG	AMB	NEG	POS	NEG	NEG	NEG	POS	NEG	POS	POS	POS	POS	POS	POS	NEG	NEG	NEG	NEG	NEG
MSSA1934	NEG	POS	NEG	AMB	NEG	POS	NEG	NEG	NEG	POS	NEG	NEG	POS	POS	NEG	POS	POS	NEG	NEG	NEG	NEG	NEG
MSSA1946	NEG	POS	NEG	NEG	NEG	AMB	NEG	NEG	NEG	POS	NEG	NEG	POS	POS	NEG	POS	POS	NEG	NEG	NEG	NEG	NEG
MSSA1971	NEG	POS	NEG	AMB	NEG	POS	NEG	NEG	NEG	POS	NEG	POS	POS	POS	POS	POS	POS	NEG	NEG	NEG	NEG	NEG
MSSA1980	NEG	POS	NEG	NEG	NEG	AMB	NEG	NEG	NEG	POS	NEG	NEG	POS	POS	NEG	POS	POS	NEG	NEG	NEG	NEG	NEG
MSSA1982	NEG	POS	NEG	NEG	NEG	POS	NEG	NEG	NEG	POS	NEG	NEG	POS	POS	NEG	POS	POS	NEG	NEG	NEG	NEG	NEG
MSSA1996	NEG	POS	NEG	AMB	NEG	AMB	NEG	NEG	NEG	POS	NEG	NEG	POS	POS	NEG	POS	POS	NEG	NEG	NEG	NEG	NEG
MSSA2002	NEG	POS	NEG	AMB	NEG	POS	NEG	NEG	NEG	POS	NEG	NEG	POS	POS	NEG	POS	POS	NEG	NEG	NEG	NEG	NEG
MSSA2014	NEG	POS	NEG	NEG	NEG	POS	NEG	NEG	NEG	POS	NEG	NEG	POS	POS	POS	POS	POS	NEG	NEG	NEG	AMB	AMB
MSSA2053	NEG	POS	NEG	AMB	NEG	POS	NEG	NEG	NEG	POS	NEG	NEG	POS	POS	NEG	POS	POS	NEG	NEG	NEG	NEG	NEG
MSSA2054	NEG	POS	NEG	AMB	NEG	AMB	NEG	NEG	NEG	POS	NEG	NEG	POS	POS	NEG	POS	POS	NEG	NEG	NEG	NEG	NEG
MSSA2058	NEG	POS	NEG	NEG	NEG	NEG	NEG	NEG	NEG	POS	NEG	NEG	POS	POS	NEG	POS	POS	NEG	NEG	NEG	NEG	NEG
MSSA2068	NEG	POS	NEG	AMB	NEG	POS	NEG	NEG	NEG	POS	NEG	NEG	POS	POS	POS	POS	POS	NEG	NEG	NEG	NEG	NEG
MSSA2070	NEG	POS	NEG	NEG	NEG	POS	NEG	NEG	NEG	POS	NEG	NEG	POS	POS	POS	POS	POS	NEG	NEG	NEG	NEG	NEG
MSSA2072	NEG	POS	NEG	AMB	NEG	POS	NEG	NEG	NEG	POS	NEG	NEG	POS	POS	POS	POS	POS	NEG	NEG	NEG	NEG	NEG
MSSA2073	NEG	AMB	NEG	NEG	NEG	NEG	NEG	NEG	NEG	POS	NEG	NEG	POS	POS	NEG	POS	POS	NEG	NEG	NEG	NEG	NEG
MSSA2074	NEG	POS	NEG	NEG	NEG	POS	NEG	NEG	NEG	POS	AMB	POS	POS	POS	POS	POS	POS	NEG	NEG	NEG	NEG	NEG
MSSA2107	NEG	POS	NEG	NEG	NEG	POS	NEG	NEG	NEG	POS	NEG	POS	POS	POS	POS	POS	POS	NEG	NEG	NEG	NEG	NEG
MSSA2108	NEG	POS	NEG	NEG	NEG	AMB	NEG	NEG	NEG	POS	NEG	NEG	POS	POS	NEG	POS	POS	NEG	NEG	NEG	NEG	NEG
MSSA2129	NEG	POS	NEG	POS	NEG	POS	NEG	NEG	NEG	POS	NEG	NEG	POS	POS	POS	POS	POS	NEG	NEG	NEG	NEG	NEG
MSSA2135	NEG	POS	NEG	NEG	NEG	POS	NEG	NEG	NEG	POS	AMB	POS	POS	POS	POS	POS	POS	NEG	NEG	NEG	NEG	NEG
MSSA2137	NEG	POS	NEG	NEG	NEG	NEG	AMB	NEG	NEG	POS	NEG	NEG	POS	POS	POS	NEG	NEG	POS	POS	NEG	NEG	NEG
MSSA2140	NEG	POS	NEG	AMB	NEG	POS	NEG	NEG	NEG	POS	NEG	POS	POS	POS	NEG	POS	POS	NEG	NEG	NEG	NEG	NEG
MSSA2182	NEG	POS	NEG	NEG	NEG	AMB	NEG	NEG	NEG	POS	NEG	POS	POS	POS	NEG	POS	POS	NEG	NEG	NEG	NEG	NEG
MSSA2188	NEG	POS	NEG	NEG	NEG	POS	NEG	NEG	NEG	POS	NEG	POS	POS	POS	POS	POS	POS	NEG	NEG	NEG	NEG	NEG
MSSA1899	NEG	POS	NEG	POS	NEG	POS	POS	NEG	POS	AMB	POS	AMB	POS	NEG	POS	NEG	NEG	POS	POS	NEG	NEG	NEG
MSSA1915	NEG	POS	NEG	AMB	NEG	POS	POS	NEG	POS	NEG	POS	AMB	POS	NEG	POS	NEG	NEG	POS	POS	NEG	NEG	NEG
MSSA1949	NEG	POS	NEG	POS	NEG	POS	POS	NEG	POS	AMB	POS	AMB	POS	NEG	POS	NEG	NEG	POS	POS	NEG	NEG	NEG
MSSA2042	NEG	POS	NEG	POS	NEG	POS	POS	NEG	POS	AMB	POS	AMB	POS	NEG	POS	NEG	NEG	POS	POS	NEG	NEG	NEG
MSSA2077	NEG	POS	NEG	AMB	NEG	POS	POS	NEG	POS	NEG	POS	NEG	POS	NEG	POS	NEG	NEG	POS	POS	NEG	NEG	NEG
MSSA2095	NEG	POS	NEG	POS	NEG	POS	POS	NEG	POS	AMB	POS	AMB	POS	AMB	POS	NEG	NEG	POS	POS	NEG	AMB	AMB
MSSA1997	NEG	POS	NEG	POS	NEG	POS	POS	NEG	POS	AMB	POS	AMB	POS	NEG	POS	POS	POS	NEG	NEG	NEG	NEG	NEG
MSSA1810	NEG	POS	NEG	NEG	NEG	POS	POS	NEG	POS	AMB	POS	NEG	POS	NEG	POS	POS	POS	NEG	NEG	NEG	NEG	NEG
MSSA1832	NEG	POS	NEG	NEG	NEG	POS	POS	NEG	POS	AMB	POS	AMB	POS	NEG	POS	POS	POS	NEG	NEG	NEG	NEG	NEG
MSSA1891	NEG	POS	NEG	NEG	NEG	POS	POS	NEG	POS	AMB	POS	AMB	POS	NEG	POS	POS	POS	NEG	NEG	NEG	NEG	NEG
MSSA1892	NEG	POS	NEG	NEG	NEG	POS	POS	NEG	POS	AMB	POS	AMB	POS	NEG	POS	POS	POS	NEG	NEG	NEG	NEG	NEG
MSSA1932	NEG	POS	NEG	NEG	NEG	POS	POS	NEG	POS	AMB	POS	AMB	POS	NEG	POS	POS	POS	NEG	NEG	NEG	NEG	NEG
MSSA1942	NEG	POS	NEG	NEG	NEG	POS	POS	NEG	POS	AMB	POS	AMB	POS	NEG	POS	POS	POS	NEG	NEG	NEG	NEG	NEG
MSSA1957	NEG	POS	NEG	NEG	NEG	POS	POS	NEG	POS	AMB	POS	AMB	POS	NEG	POS	POS	POS	NEG	NEG	NEG	NEG	NEG
MSSA1748	POS	POS	POS	NEG	POS	POS	POS	NEG	POS	AMB	AMB	POS	POS	POS	NEG	POS	POS	NEG	NEG	POS	AMB	AMB
MSSA1754	POS	POS	POS	NEG	POS	POS	POS	NEG	POS	AMB	AMB	POS	POS	POS	NEG	POS	POS	NEG	NEG	POS	NEG	NEG
MSSA1770	POS	POS	POS	NEG	POS	POS	POS	NEG	POS	AMB	AMB	POS	POS	POS	NEG	POS	POS	NEG	NEG	POS	NEG	NEG
MSSA1788	POS	POS	POS	NEG	POS	POS	POS	NEG	POS	AMB	AMB	POS	POS	POS	NEG	POS	POS	NEG	NEG	POS	NEG	NEG
MSSA1804	POS	POS	POS	NEG	POS	POS	POS	NEG	POS	AMB	AMB	POS	POS	POS	NEG	POS	POS	NEG	NEG	POS	NEG	NEG
MSSA1818	POS	POS	POS	NEG	POS	POS	POS	NEG	POS	AMB	AMB	POS	POS	POS	NEG	POS	POS	NEG	NEG	POS	NEG	NEG
MSSA1914	POS	POS	POS	NEG	POS	POS	POS	NEG	POS	AMB	AMB	POS	POS	POS	POS	POS	POS	NEG	NEG	POS	AMB	AMB
MSSA1974	POS	POS	POS	NEG	POS	POS	POS	NEG	POS	AMB	AMB	POS	POS	POS	NEG	POS	POS	NEG	NEG	POS	NEG	NEG
MSSA2023	POS	POS	POS	NEG	POS	POS	POS	NEG	POS	AMB	AMB	POS	POS	POS	NEG	POS	POS	NEG	NEG	POS	NEG	NEG
MSSA2030	POS	POS	POS	NEG	POS	POS	POS	NEG	POS	AMB	AMB	POS	POS	POS	NEG	POS	POS	NEG	NEG	POS	NEG	NEG
MSSA2096	NEG	POS	POS	NEG	POS	POS	POS	NEG	POS	AMB	NEG	POS	POS	POS	NEG	POS	POS	NEG	NEG	AMB	NEG	NEG
MSSA2142	POS	POS	POS	NEG	POS	AMB	POS	NEG	POS	AMB	AMB	POS	POS	POS	NEG	POS	POS	NEG	NEG	POS	NEG	NEG
MSSA2203	POS	POS	POS	NEG	POS	POS	POS	NEG	POS	AMB	POS	AMB	POS	POS	NEG	POS	POS	NEG	NEG	POS	NEG	NEG
MSSA1901	NEG	POS	POS	NEG	POS	NEG	NEG	NEG	POS	AMB	POS	AMB	POS	NEG	POS	NEG	NEG	POS	POS	NEG	NEG	NEG
MSSA2013	NEG	POS	POS	NEG	POS	NEG	NEG	NEG	POS	AMB	POS	AMB	POS	NEG	POS	NEG	NEG	POS	POS	NEG	AMB	AMB
MSSA2031	NEG	POS	POS	AMB	POS	NEG	NEG	NEG	POS	AMB	POS	AMB	POS	AMB	POS	NEG	NEG	POS	POS	NEG	POS	POS
MSSA2052	NEG	POS	POS	NEG	POS	NEG	NEG	NEG	POS	AMB	POS	AMB	POS	NEG	POS	NEG	NEG	POS	POS	NEG	NEG	NEG
MSSA2057	NEG	POS	POS	NEG	POS	NEG	NEG	NEG	POS	AMB	POS	AMB	POS	NEG	POS	NEG	NEG	POS	POS	NEG	NEG	NEG
MSSA2081	POS	POS	POS	NEG	POS	POS	POS	NEG	POS	AMB	POS	AMB	POS	NEG	POS	NEG	NEG	POS	POS	NEG	NEG	NEG
MSSA1955	NEG	POS	NEG	NEG	NEG	POS	POS	NEG	POS	AMB	NEG	NEG	POS	NEG	POS	NEG	NEG	POS	POS	NEG	NEG	NEG
MSSA1941	POS	POS	POS	NEG	POS	AMB	NEG	AMB	NEG	POS	NEG	NEG	POS	POS	NEG	NEG	NEG	POS	POS	NEG	NEG	NEG
MRSA3838	NEG	POS	POS	NEG	POS	NEG	NEG	NEG	NEG	POS	NEG	NEG	POS	POS	NEG	POS	POS	NEG	NEG	NEG	NEG	NEG
MRSA3839	NEG	POS	POS	NEG	POS	NEG	NEG	NEG	NEG	POS	NEG	NEG	POS	POS	NEG	POS	POS	NEG	NEG	NEG	NEG	NEG
MSSA1801	NEG	POS	NEG	NEG	NEG	POS	POS	NEG	NEG	POS	NEG	NEG	POS	POS	NEG	POS	POS	NEG	NEG	NEG	NEG	NEG
MSSA2131	NEG	POS	POS	NEG	POS	NEG	NEG	NEG	NEG	POS	NEG	NEG	POS	POS	POS	POS	POS	NEG	NEG	NEG	AMB	AMB
MSSA1792	NEG	POS	NEG	POS	NEG	POS	POS	NEG	POS	AMB	POS	NEG	POS	NEG	POS	POS	POS	NEG	NEG	NEG	NEG	NEG
MSSA1796	POS	POS	NEG	NEG	NEG	POS	POS	NEG	POS	AMB	POS	AMB	POS	AMB	POS	NEG	NEG	POS	POS	NEG	AMB	AMB
MSSA1817	NEG	NEG	NEG	NEG	NEG	POS	POS	NEG	POS	NEG	POS	AMB	POS	NEG	POS	NEG	NEG	POS	POS	NEG	NEG	NEG
MSSA1911	NEG	POS	NEG	NEG	NEG	POS	POS	NEG	NEG	POS	POS	NEG	NEG	NEG	NEG	NEG	POS	NEG	NEG	NEG	NEG	NEG
MSSA1927	NEG	POS	NEG	NEG	NEG	POS	POS	NEG	POS	AMB	POS	AMB	POS	NEG	POS	POS	POS	NEG	NEG	POS	AMB	AMB
MSSA1928	NEG	POS	NEG	NEG	NEG	POS	POS	NEG	POS	AMB	POS	AMB	POS	NEG	POS	POS	POS	NEG	NEG	POS	NEG	NEG
MSSA1953	NEG	POS	NEG	NEG	NEG	POS	POS	NEG	POS	AMB	POS	AMB	POS	NEG	POS	POS	POS	NEG	NEG	POS	NEG	NEG
MSSA2062	NEG	POS	NEG	POS	NEG	POS	POS	NEG	POS	AMB	POS	AMB	POS	NEG	POS	POS	POS	NEG	NEG	NEG	NEG	NEG
MSSA2078	POS	POS	POS	AMB	POS	POS	POS	NEG	NEG	POS	POS	AMB	POS	POS	NEG	POS	POS	NEG	NEG	NEG	AMB	AMB
MSSA2136	NEG	POS	POS	NEG	POS	NEG	NEG	NEG	POS	AMB	AMB	POS	POS	NEG	POS	POS	POS	NEG	NEG	NEG	AMB	AMB
