# Supplementary material for: Microarray-Based Genotyping and Clinical Outcomes of Staphylococcus aureus Bloodstream Infection: An Exploratory Study
Source: PLoS One. 2013 Aug 14;8(8):e71259. doi: 10.1371/journal.pone.0071259 (PMC3743874; doi:10.1371/journal.pone.0071259)
Supplement: File S3 — Distribution of clonal complexes and spa types in 317 patients with SAB. (PDF) [file pone.0071259.s003.pdf]

Supplemental file S3

Distribution of clonal complexes and *spa* types in 317 patients with SAB

Clonal complex	total n=317	Number of different *spa* types	Most commen *spa* types (number) $
CC5	50	17	t003 (20), t002 (13), t045 and t062 (2)
CC45	46	29	t015 (14), t050, t230, t331 and t630 (2)
CC30	39	24	t012 (13), t021 (3), t153 (2)
CC15	28	14	t084 (11), t346 (3), t254 and t360 (2)
CC7	27	4	t091 (23), t1943 (2)
CC8	24	11	t008 (11), t024 (2), t068 and t6395 (2)
CC22	22	8	t005 (8), t608 (5), t032 (4)
CC101	13	5	t056 (9)
CC12	10	4	t160 (6), t156 (2)
CC1	8	4	t127 (5)
CC121	6	6	
CC25	6	6	
CC59	6	3	t216 (4)
CC9	6	2	t100 (4), t209 (2)
CC97	6	4	t359 (2), t3051 (2)
CC398	4	4	
CC20	2	2	

CC78, CC80, CC182, CC395 were found as single isolates. Ten isolates could not be assigned to a clonal complex, one isolate could not be *spa* typed.
$ Single spa types are not shown.
