# Supplementary material for: Microarray-Based Genotyping and Clinical Outcomes of Staphylococcus aureus Bloodstream Infection: An Exploratory Study
Source: PLoS One. 2013 Aug 14;8(8):e71259. doi: 10.1371/journal.pone.0071259 (PMC3743874; doi:10.1371/journal.pone.0071259)
Supplement: File S4 — Complete data of univariate analyses of pathogen factors (clonal complex, agr type and microarray-derived virulence and resistance genes) associated with investigated clinical endpoints. (PDF) [file pone.0071259.s004.pdf]

Supplemental File S4

Complete data of univariate analyses of pathogen factors (clonal complex, agr type and microarray-derived virulence/resistance genes) associated with investigated clinical endpoints)

Mortality day 30

p-values of Fisher's exact tests and frequencies in percent for all microarray-derived virulence/resistance genes:

	p-value	Percentage
Ribos.STAU	1.00	100
gapA	1.00	100
katA	1.00	100
CoA	0.56	99
proteinA	0.34	99
sbi	0.34	99
nuc	1.00	100
hp.fnbA.615	0.56	99
hp.vraS.612	1.00	100
hp.sarA.611	1.00	100
hp.eno.611	1.00	100
sarA.new	1.00	100
saeS	1.00	100
vraS	1.00	100
mecA	<0.01	10
delta.mecR	<0.01	10
ugpQ	<0.01	10
ccrA.1	0.56	1
ccrB.1.612	1.00	1
ccrB.1.613	1.00	0
plsSCC.COL	1.00	0
Q9XB68.dcs	<0.01	9
ccrA.2	<0.01	9
ccrB.2	<0.01	9
kdpA.SCC	0.01	7
kdpB.SCC	0.01	7
kdpC.SCC	0.01	7
kdpD.SCC	0.01	7
kdpE.SCC	0.01	7
mecI	<0.01	7
mecR	<0.01	7
xylR	<0.01	7
ccrA.3	0.34	1
ccrB.3	0.18	0
merA	1.00	1
merB	1.00	1
ccrAA.MRSAZH47.any	1.00	1
ccrC.85.2082	0.46	1
ccrA.4	1.00	0
ccrB.4	1.00	1
blaZ.any	<0.01	70
blaI	<0.01	71
blaR.any	<0.01	70
ermA	0.02	12
ermB	1.00	0
ermC	<0.01	6
linA	1.00	0
msrA	1.00	0
mefA.any	1.00	0
mpbBM.any	1.00	0
vatA	1.00	0
vatB	1.00	0
vga	1.00	0

vgaA	1.00	0
vgb	1.00	0
aacA.aphD	1.00	2
aadD	<0.01	7
aphA.3	1.00	0
sat	1.00	0
dfrA	0.46	1
far1	1.00	0
Q6GD50	1.00	1
mupR	1.00	1
tetK	0.40	3
tetM	0.09	3
tetEfflux	1.00	93
cat.any	1.00	0
cfr	1.00	0
fexA	1.00	0
fosB	0.14	60
fosB.plasmid	0.34	1
qacA	1.00	1
qacC.any	0.70	3
tst.any	0.36	11
entA.any	0.76	36
entB	1.00	9
entC	0.85	18
entD	0.04	9
entE	1.00	0
entG	1.00	57
entH	0.49	5
entI	1.00	57
entJ	0.04	9
entK.any	0.19	5
entL	1.00	13
entM	1.00	57
entN	1.00	57
entO	1.00	57
entQ.any	0.19	5
entR	0.04	9
entU	1.00	57
lukF	0.36	97
lukS.any	1.00	99
hlgA	0.78	93
lukF.PV.any	1.00	0
lukS.PV	1.00	0
lukM	1.00	0
lukD	0.46	59
lukE	0.88	59
lukX	1.00	99
lukY.any	1.00	99
hl	0.46	99
hla	0.67	97
hld	1.00	100
hlIII.all.any	1.00	93
hlb.611	0.65	63
hlb.612	1.00	73
hlb.613	0.23	61
un.truncated.hlb	0.44	9
sak.any	0.24	83
chp	0.23	63
hp.scn.611	1.00	93
etA	0.56	1
etB	1.00	0
etD	1.00	3
edinA	1.00	0
edinB	1.00	3
edinC	1.00	0
arcA.SCC	1.00	0
arcB.SCC	1.00	0

arcC.SCC	1.00	0
arcD.SCC	1.00	0
aur.any	1.00	100
splA	0.38	60
splB	0.37	60
splE	0.56	52
sspA.all	1.00	100
sspB.any	1.00	100
sspP.any	1.00	100
setC.MW0345	0.73	77
ssl1.any	0.34	99
ssl2.any	0.59	98
ssl3.any	0.42	71
ssl4.any	1.00	99
ssl5.any	1.00	99
ssl6.any	0.30	40
ssl7.any	0.13	96
ssl8.any	0.65	64
ssl9.any	0.36	98
ssl10.any	1.00	95
ssl11.any	0.01	52
setB3.any	1.00	93
setB2.any	0.73	78
setB1	1.00	77
capH1	1.00	0
capJ1	1.00	0
capK1	1.00	0
capH5	0.88	40
capJ5.any	0.88	40
capK5	0.88	40
capH8	0.88	60
capI8	0.88	60
capJ8	0.88	60
capK8.any	0.88	60
icaA	1.00	99
icaC	1.00	99
icaD	1.00	99
bap	1.00	0
bbp.any	0.65	89
clfA.any	1.00	100
clfB.any	1.00	99
cna	1.00	42
ebh.all	1.00	93
ebpS.any	1.00	99
eno	1.00	100
fib.any	0.56	99
fnbA.any	0.56	99
fnbB.any	0.35	82
map.any	0.47	96
sdrC.any	1.00	100
sdrD.any	0.67	87
vwb.any	1.00	100
sasG.any	0.03	50
isaB.any	1.00	100
mprF.any	0.82	88
isdA.any	1.00	100
ImrP.any	1.00	100
Q2YUB3	1.00	2
hsdS1.RF122	1.00	1
hsdS2any	0.87	73
hsdS3.any	0.67	53
hsdSx	0.47	80
Q2FXC0	0.56	17
Q7A4X2	1.00	2
hysA1.2.any	0.34	99
hysA2.any	0.27	96

p-values of Fisher's exact tests and frequencies in percent for the seven most frequent clonal complexes:

	p-value	Percentage
CC15	0.02	9
CC22	1.00	7
CC30	1.00	12
CC45	1.00	14
CC5	0.84	16
CC7	0.19	9
CC8	0.41	8

The p-value of the Chi-squared test for agr type is 0.16. Frequency distribution:

alive at day 30	agrI	agrII	agrIII	agrIV	Sum
No	25	23	10	0	58
Yes	137	70	38	7	252
Sum	162	93	48	7	310

Severe sepsis or septic shock

p-values of Fisher's exact tests and frequencies in percent for all microarray-derived virulence/resistance genes:

	p-value	Percentage
Ribos.STAU	1.00	100
gapA	1.00	100
katA	0.32	100
CoA	0.60	99
proteinA	0.54	99
sbi	0.54	99
nuc	1.00	100
hp.fnbA.615	0.60	99
hp.vraS.612	1.00	100
hp.sarA.611	1.00	100
hp.eno.611	1.00	100
sarA.new	1.00	100
saeS	1.00	100
vraS	1.00	100
mecA	0.07	10
delta.mecR	0.04	9
ugpQ	0.07	10
ccrA.1	1.00	1
ccrB.1.612	1.00	1
ccrB.1.613	1.00	0
plsSCC.COL	1.00	0
Q9XB68.dcs	0.02	9
ccrA.2	0.05	9
ccrB.2	0.05	9
kdpA.SCC	0.05	7
kdpB.SCC	0.05	7
kdpC.SCC	0.05	7
kdpD.SCC	0.05	7
kdpE.SCC	0.05	7
mecI	0.03	7
mecR	0.03	7
xylR	0.04	7
ccrA.3	0.54	1
ccrB.3	0.32	0
merA	1.00	1
merB	1.00	1
ccrAA.MRSAZH47.any	1.00	1
ccrC.85.2082	1.00	1
ccrA.4	1.00	0
ccrB.4	1.00	1

blaZ.any	0.90	71
blaI	0.69	71
blaR.any	0.90	71
ermA	0.07	12
ermB	1.00	0
ermC	0.07	6
linA	1.00	0
msrA	1.00	0
mefA.any	1.00	0
mpbBM.any	1.00	0
vatA	1.00	0
vatB	1.00	0
vga	1.00	0
vgaA	1.00	0
vgb	1.00	0
aacA.aphD	1.00	2
aadD	0.02	7
aphA.3	1.00	0
sat	1.00	0
dfrA	0.55	1
far1	1.00	0
Q6GD50	1.00	1
mupR	1.00	1
tetK	0.51	3
tetM	0.73	3
tetEfflux	0.49	93
cat.any	1.00	0
cfr	1.00	0
fexA	1.00	0
fosB	0.01	60
fosB.plasmid	0.54	1
qacA	0.55	1
qacC.any	0.51	3
tst.any	0.25	11
entA.any	1.00	35
entB	0.84	9
entC	0.43	17
entD	0.01	9
entE	1.00	0
entG	0.72	57
entH	0.57	5
entI	0.63	57
entJ	0.01	9
entK.any	1.00	5
entL	0.15	13
entM	0.63	57
entN	0.63	57
entO	0.55	57
entQ.any	1.00	5
entR	0.01	9
entU	0.63	57
lukF	1.00	97
lukS.any	0.10	99
hlgA	0.48	93
lukF.PV.any	0.32	0
lukS.PV	0.32	0
lukM	1.00	0
lukD	0.18	59
lukE	0.27	58
lukX	0.54	99
lukY.any	1.00	99
hl	1.00	99
hla	1.00	97
hld	1.00	100
hlIII.all.any	0.49	93
hlb.611	0.38	63
hlb.612	0.23	72

hlb.613	0.27	60
un.truncated.hlb	0.83	9
sak.any	0.43	83
chp	0.90	63
hp.scn.611	0.81	93
etA	0.60	1
etB	1.00	0
etD	0.72	3
edinA	1.00	0
edinB	0.72	3
edinC	1.00	0
arcA.SCC	1.00	0
arcB.SCC	1.00	0
arcC.SCC	1.00	0
arcD.SCC	1.00	0
aur.any	0.32	100
splA	0.18	60
splB	0.11	59
splE	0.90	52
sspA.all	0.32	100
sspB.any	0.32	100
sspP.any	0.32	100
setC.MW0345	0.57	77
ssl1.any	0.54	99
ssl2.any	1.00	98
ssl3.any	0.24	71
ssl4.any	1.00	99
ssl5.any	1.00	99
ssl6.any	0.71	40
ssl7.any	0.18	97
ssl8.any	0.17	64
ssl9.any	0.44	98
ssl10.any	0.78	95
ssl11.any	0.05	52
setB3.any	0.49	93
setB2.any	0.78	77
setB1	0.78	77
capH1	1.00	0
capJ1	1.00	0
capK1	1.00	0
capH5	1.00	40
capJ5.any	1.00	40
capK5	1.00	40
capH8	0.81	60
capI8	0.81	60
capJ8	0.81	60
capK8.any	0.81	60
icaA	0.24	99
icaC	0.10	99
icaD	0.10	99
bap	1.00	0
bbp.any	0.58	88
clfA.any	0.32	100
clfB.any	1.00	99
cna	0.33	43
ebh.all	0.49	93
ebpS.any	0.54	99
eno	1.00	100
fib.any	0.60	99
fnbA.any	0.60	99
fnbB.any	0.88	82
map.any	0.56	96
sdrC.any	1.00	100
sdrD.any	0.59	87
vwb.any	0.32	100
sasG.any	0.15	50
isaB.any	1.00	100

mprF.any	0.19	88
isdA.any	0.32	100
ImrP.any	1.00	100
Q2YUB3	0.66	2
hsdS1.RF122	1.00	1
hsdS2any	0.69	73
hsdS3.any	0.90	52
hsdSx	0.37	80
Q2FXC0	0.52	16
Q7A4X2	0.66	2
hysA1.2.any	1.00	99
hysA2.any	1.00	96

p-values of Fisher's exact tests and frequencies in percent for the seven most frequent clonal complexes:

	p-value	Percentage
CC15	0.05	9
CC22	0.64	7
CC30	0.46	12
CC45	0.40	15
CC5	0.51	16
CC7	0.29	9
CC8	0.65	8

The p-value of the Chi-squared test for agr type is 0.08. Frequency distribution:

Severe sepsis or septic shock	agrI	agrII	agrIII	agrIV	Sum
No	116	54	37	5	212
Yes	47	40	12	2	101
Sum	163	94	49	7	313

Disseminated infection

p-values of Fisher's exact tests and frequencies in percent for all microarray-derived virulence/resistance genes:

	p-value	Percentage
Ribos.STAU	1.00	100
gapA	1.00	100
katA	0.25	100
CoA	1.00	99
proteinA	0.44	99
sbi	0.44	99
nuc	1.00	100
hp.fnbA.615	0.58	99
hp.vraS.612	1.00	100
hp.sarA.611	1.00	100
hp.eno.611	1.00	100
sarA.new	1.00	100
saeS	1.00	100
vraS	1.00	100
mecA	1.00	10
delta.mecR	0.83	9
ugpQ	1.00	10
ccrA.1	1.00	1
ccrB.1.612	1.00	1
ccrB.1.613	1.00	0
plsSCC.COL	1.00	0
Q9XB68.dcs	0.82	9
ccrA.2	0.65	9
ccrB.2	0.65	9
kdpA.SCC	0.79	7
kdpB.SCC	0.79	7
kdpC.SCC	0.79	7

kdpD.SCC	0.79	7
kdpE.SCC	0.79	7
mecI	0.80	7
mecR	0.80	7
xylR	1.00	7
ccrA.3	1.00	1
ccrB.3	1.00	0
merA	1.00	1
merB	1.00	1
ccrAA.MRSAZH47.any	1.00	1
ccrC.85.2082	0.58	1
ccrA.4	1.00	0
ccrB.4	0.58	1
blaZ.any	0.20	71
blaI	0.15	71
blaR.any	0.20	71
ermA	0.56	12
ermB	1.00	0
ermC	0.58	6
linA	1.00	0
msrA	1.00	0
mefA.any	1.00	0
mpbBM.any	1.00	0
vatA	1.00	0
vatB	1.00	0
vga	1.00	0
vgaA	1.00	0
vgb	1.00	0
aacA.aphD	0.34	2
aadD	0.79	7
aphA.3	1.00	0
sat	1.00	0
dfrA	1.00	1
far1	1.00	0
Q6GD50	0.44	1
mupR	1.00	1
tetK	0.07	3
tetM	0.27	3
tetEfflux	0.81	93
cat.any	1.00	0
cfr	1.00	0
fexA	1.00	0
fosB	1.00	60
fosB.plasmid	1.00	1
qacA	1.00	1
qacC.any	0.46	3
tst.any	0.54	11
entA.any	0.18	35
entB	1.00	9
entC	0.73	17
entD	0.26	9
entE	1.00	0
entG	0.51	57
entH	1.00	5
entI	0.43	57
entJ	0.26	9
entK.any	1.00	5
entL	1.00	13
entM	0.43	57
entN	0.43	57
entO	0.43	57
entQ.any	1.00	5
entR	0.26	9
entU	0.43	57
lukF	1.00	97
lukS.any	0.06	99
hlgA	0.31	93

lukF.PV.any	0.25	0
lukS.PV	0.25	0
lukM	1.00	0
lukD	0.19	59
lukE	0.04	58
lukX	0.44	99
lukY.any	1.00	99
hl	0.15	99
hla	0.70	97
hld	1.00	100
hlIII.all.any	0.81	93
hlb.611	<0.01	63
hlb.612	0.02	72
hlb.613	0.03	60
un.truncated.hlb	0.25	9
sak.any	0.01	83
chp	0.03	63
hp.scn.611	0.61	93
etA	1.00	1
etB	1.00	0
etD	1.00	3
edinA	1.00	0
edinB	1.00	3
edinC	1.00	0
arcA.SCC	1.00	0
arcB.SCC	1.00	0
arcC.SCC	1.00	0
arcD.SCC	1.00	0
aur.any	0.25	100
splA	0.09	60
splB	0.11	59
splE	0.30	52
sspA.all	0.25	100
sspB.any	0.25	100
sspP.any	0.25	100
setC.MW0345	0.64	77
ssl1.any	0.44	99
ssl2.any	1.00	98
ssl3.any	1.00	71
ssl4.any	0.44	99
ssl5.any	0.15	99
ssl6.any	1.00	40
ssl7.any	0.48	97
ssl8.any	0.23	64
ssl9.any	0.69	98
ssl10.any	0.77	95
ssl11.any	0.90	52
setB3.any	0.81	93
setB2.any	0.64	77
setB1	0.64	77
capH1	1.00	0
capJ1	1.00	0
capK1	1.00	0
capH5	0.89	40
capJ5.any	0.89	40
capK5	0.89	40
capH8	0.79	60
capI8	0.69	60
capJ8	0.79	60
capK8.any	0.79	60
icaA	0.15	99
icaC	0.06	99
icaD	0.06	99
bap	1.00	0
bbp.any	0.31	88
clfA.any	0.25	100
clfB.any	1.00	99

cna	0.79	43
ebh.all	0.81	93
ebpS.any	0.44	99
eno	1.00	100
fib.any	0.05	99
fnbA.any	0.58	99
fnbB.any	0.50	82
map.any	0.74	96
sdrC.any	1.00	100
sdrD.any	0.85	87
vwb.any	0.25	100
sasG.any	0.80	50
isaB.any	1.00	100
mprF.any	1.00	88
isdA.any	0.25	100
ImrP.any	1.00	100
Q2YUB3	0.34	2
hsdS1.RF122	1.00	1
hsdS2any	0.56	73
hsdS3.any	0.52	52
hsdSx	0.87	80
Q2FXC0	0.02	16
Q7A4X2	0.34	2
hysA1.2.any	1.00	99
hysA2.any	0.10	96

p-values of Fisher's exact tests and frequencies in percent for the seven most frequent clonal complexes:

	p-value	Percentage
CC15	0.02	9
CC22	0.61	7
CC30	0.69	12
CC45	1.00	15
CC5	1.00	16
CC7	0.35	9
CC8	0.33	8

The p-value of the Chi-squared test for agr type is 0.62. Frequency distribution:

Disseminated infection	agrI	agrII	agrIII	agrIV	Sum
No	118	75	37	5	235
Yes	45	19	12	2	78
Sum	163	94	49	7	313

Endocarditis

p-values of Fisher's exact tests and frequencies in percent for all microarray-derived virulence/resistance genes:

	p-value	Percentage
Ribos.STAU	1.00	100
gapA	1.00	100
katA	0.11	100
CoA	0.38	99
proteinA	0.21	99
sbi	0.21	99
nuc	1.00	100
hp.fnbA.615	1.00	99
hp.vraS.612	1.00	100
hp.sarA.611	1.00	100
hp.eno.611	1.00	100
sarA.new	1.00	100
saeS	1.00	100
vraS	1.00	100

mecA	1.00	10
delta.mecR	1.00	9
ugpQ	1.00	10
ccrA.1	1.00	1
ccrB.1.612	0.30	1
ccrB.1.613	1.00	0
plsSCC.COL	1.00	0
Q9XB68.dcs	1.00	9
ccrA.2	1.00	9
ccrB.2	1.00	9
kdpA.SCC	1.00	7
kdpB.SCC	1.00	7
kdpC.SCC	1.00	7
kdpD.SCC	1.00	7
kdpE.SCC	1.00	7
mecI	1.00	7
mecR	1.00	7
xylR	1.00	7
ccrA.3	1.00	1
ccrB.3	1.00	0
merA	1.00	1
merB	1.00	1
ccrAA.MRSAZH47.any	1.00	1
ccrC.85.2082	1.00	1
ccrA.4	1.00	0
ccrB.4	1.00	1
blaZ.any	1.00	71
blaI	1.00	71
blaR.any	1.00	71
ermA	0.59	12
ermB	1.00	0
ermC	1.00	6
linA	1.00	0
msrA	1.00	0
mefA.any	1.00	0
mpbBM.any	1.00	0
vatA	1.00	0
vatB	1.00	0
vga	1.00	0
vgaA	1.00	0
vgb	1.00	0
aacA.aphD	1.00	2
aadD	1.00	7
aphA.3	1.00	0
sat	1.00	0
dfrA	1.00	1
far1	1.00	0
Q6GD50	1.00	1
mupR	1.00	1
tetK	0.61	3
tetM	0.30	3
tetEfflux	0.30	93
cat.any	1.00	0
cfr	1.00	0
fexA	1.00	0
fosB	0.36	60
fosB.plasmid	1.00	1
qacA	1.00	1
qacC.any	1.00	3
tst.any	0.78	11
entA.any	0.85	35
entB	0.54	9
entC	0.81	17
entD	0.11	9
entE	1.00	0
entG	0.21	57
entH	0.68	5

entI	0.15	57
entJ	0.11	9
entK.any	0.69	5
entL	0.28	13
entM	0.15	57
entN	0.15	57
entO	0.15	57
entQ.any	0.69	5
entR	0.11	9
entU	0.15	57
lukF	1.00	97
lukS.any	0.21	99
hlgA	1.00	93
lukF.PV.any	1.00	0
lukS.PV	1.00	0
lukM	1.00	0
lukD	0.28	59
lukE	0.10	58
lukX	0.21	99
lukY.any	0.38	99
hl	0.30	99
hla	1.00	97
hld	1.00	100
hlIII.all.any	0.30	93
hlb.611	0.07	63
hlb.612	0.16	72
hlb.613	0.58	60
un.truncated.hlb	1.00	9
sak.any	0.81	83
chp	0.06	63
hp.scn.611	0.71	93
etA	0.38	1
etB	1.00	0
etD	1.00	3
edinA	1.00	0
edinB	1.00	3
edinC	1.00	0
arcA.SCC	1.00	0
arcB.SCC	1.00	0
arcC.SCC	1.00	0
arcD.SCC	1.00	0
aur.any	0.11	100
splA	0.20	60
splB	0.15	59
splE	0.59	52
sspA.all	0.11	100
sspB.any	0.11	100
sspP.any	0.11	100
setC.MW0345	0.13	77
ssl1.any	1.00	99
ssl2.any	0.45	98
ssl3.any	0.84	71
ssl4.any	1.00	99
ssl5.any	0.30	99
ssl6.any	0.36	40
ssl7.any	0.11	97
ssl8.any	0.36	64
ssl9.any	1.00	98
ssl10.any	0.68	95
ssl11.any	0.48	52
setB3.any	0.30	93
setB2.any	1.00	77
setB1	1.00	77
capH1	1.00	0
capJ1	1.00	0
capK1	1.00	0
capH5	0.72	40

capJ5.any	0.72	40
capK5	0.72	40
capH8	0.47	60
capI8	0.47	60
capJ8	0.47	60
capK8.any	0.47	60
icaA	0.30	99
icaC	0.21	99
icaD	0.21	99
bap	1.00	0
bbp.any	0.27	88
clfA.any	0.11	100
clfB.any	1.00	99
cna	0.86	43
ebh.all	0.30	93
ebpS.any	0.21	99
eno	1.00	100
fib.any	0.38	99
fnbA.any	1.00	99
fnbB.any	0.65	82
map.any	0.64	96
sdrC.any	1.00	100
sdrD.any	0.79	87
vwb.any	0.11	100
sasG.any	0.86	50
isaB.any	1.00	100
mprF.any	0.40	88
isdA.any	0.11	100
ImrP.any	1.00	100
Q2YUB3	1.00	2
hsdS1.RF122	0.30	1
hsdS2any	0.84	73
hsdS3.any	0.47	52
hsdSx	0.50	80
Q2FXC0	0.33	16
Q7A4X2	1.00	2
hysA1.2.any	1.00	99
hysA2.any	1.00	96

p-values of Fisher's exact tests and frequencies in percent for the seven most frequent clonal complexes:

	p-value	Percentage
CC15	0.75	9
CC22	0.72	7
CC30	0.28	12
CC45	0.80	15
CC5	0.62	16
CC7	0.20	9
CC8	0.32	8

The p-value of the Chi-squared test for agr type is 0.64. Frequency distribution:

Endocarditis	agrI	agrII	agrIII	agrIV	Sum
No	142	86	45	6	279
Yes	21	8	4	1	34
Sum	163	94	49	7	313

Osteoarticular infection

p-values of Fisher's exact tests and frequencies in percent for all microarray-derived virulence/resistance genes:

	p-value	Percentage
Ribos.STAU	1.00	100
gapA	1.00	100
katA	1.00	100
CoA	1.00	99
proteinA	1.00	99
sbi	1.00	99
nuc	1.00	100
hp.fnbA.615	0.50	99
hp.vraS.612	1.00	100
hp.sarA.611	1.00	100
hp.eno.611	1.00	100
sarA.new	1.00	100
saeS	1.00	100
vraS	1.00	100
mecA	1.00	10
delta.mecR	0.80	9
ugpQ	1.00	10
ccrA.1	1.00	1
ccrB.1.612	1.00	1
ccrB.1.613	1.00	0
plsSCC.COL	1.00	0
Q9XB68.dcs	0.79	9
ccrA.2	0.79	9
ccrB.2	0.79	9
kdpA.SCC	0.76	7
kdpB.SCC	0.76	7
kdpC.SCC	0.76	7
kdpD.SCC	0.76	7
kdpE.SCC	0.76	7
mecI	0.76	7
mecR	0.76	7
xylR	0.77	7
ccrA.3	1.00	1
ccrB.3	1.00	0
merA	1.00	1
merB	1.00	1
ccrAA.MRSAZH47.any	1.00	1
ccrC.85.2082	1.00	1
ccrA.4	1.00	0
ccrB.4	1.00	1
blaZ.any	0.18	71
blaI	0.13	71
blaR.any	0.18	71
ermA	0.64	12
ermB	1.00	0
ermC	0.20	6
linA	1.00	0
msrA	1.00	0
mefA.any	1.00	0
mpbBM.any	1.00	0
vatA	1.00	0
vatB	1.00	0
vga	1.00	0
vgaA	1.00	0
vgb	1.00	0
aacA.aphD	1.00	2
aadD	1.00	7
aphA.3	1.00	0
sat	1.00	0
dfrA	1.00	1

far1	1.00	0
Q6GD50	1.00	1
mupR	1.00	1
tetK	0.37	3
tetM	0.66	3
tetEfflux	0.02	93
cat.any	1.00	0
cfr	1.00	0
fexA	1.00	0
fosB	0.43	60
fosB.plasmid	1.00	1
qacA	0.40	1
qacC.any	0.37	3
tst.any	0.81	11
entA.any	0.63	35
entB	1.00	9
entC	0.10	17
entD	0.28	9
entE	1.00	0
entG	0.02	57
entH	0.48	5
entI	0.03	57
entJ	0.28	9
entK.any	0.48	5
entL	0.06	13
entM	0.03	57
entN	0.03	57
entO	0.03	57
entQ.any	0.48	5
entR	0.28	9
entU	0.03	57
lukF	0.62	97
lukS.any	1.00	99
hlgA	1.00	93
lukF.PV.any	1.00	0
lukS.PV	1.00	0
lukM	1.00	0
lukD	0.03	59
lukE	0.06	58
lukX	1.00	99
lukY.any	1.00	99
hl	0.40	99
hla	0.64	97
hld	1.00	100
hlIII.all.any	0.02	93
hlb.611	0.27	63
hlb.612	0.23	72
hlb.613	0.08	60
un.truncated.hlb	0.28	9
sak.any	0.07	83
chp	0.11	63
hp.scn.611	0.55	93
etA	0.12	1
etB	0.16	0
etD	0.36	3
edinA	1.00	0
edinB	0.36	3
edinC	0.16	0
arcA.SCC	1.00	0
arcB.SCC	1.00	0
arcC.SCC	1.00	0
arcD.SCC	1.00	0
aur.any	1.00	100
splA	0.04	60
splB	0.04	59
splE	0.09	52
sspA.all	1.00	100

sspB.any	1.00	100
sspP.any	1.00	100
setC.MW0345	0.04	77
ssl1.any	0.29	99
ssl2.any	1.00	98
ssl3.any	0.01	71
ssl4.any	0.29	99
ssl5.any	0.40	99
ssl6.any	0.06	40
ssl7.any	0.08	97
ssl8.any	0.04	64
ssl9.any	1.00	98
ssl10.any	0.07	95
ssl11.any	0.17	52
setB3.any	0.02	93
setB2.any	0.01	77
setB1	0.01	77
capH1	1.00	0
capJ1	1.00	0
capK1	1.00	0
capH5	0.53	40
capJ5.any	0.53	40
capK5	0.53	40
capH8	0.53	60
capI8	0.53	60
capJ8	0.53	60
capK8.any	0.53	60
icaA	0.40	99
icaC	0.29	99
icaD	0.29	99
bap	1.00	0
bbp.any	0.81	88
clfA.any	1.00	100
clfB.any	1.00	99
cna	0.02	43
ebh.all	0.02	93
ebpS.any	1.00	99
eno	1.00	100
fib.any	0.50	99
fnbA.any	0.50	99
fnbB.any	1.00	82
map.any	1.00	96
sdrC.any	1.00	100
sdrD.any	0.82	87
vwb.any	1.00	100
sasG.any	0.64	50
isaB.any	1.00	100
mprF.any	0.06	88
isdA.any	1.00	100
ImrP.any	1.00	100
Q2YUB3	1.00	2
hsdS1.RF122	1.00	1
hsdS2any	<0.01	73
hsdS3.any	0.36	52
hsdSx	<0.01	80
Q2FXC0	0.84	16
Q7A4X2	1.00	2
hysA1.2.any	1.00	99
hysA2.any	<0.01	96

p-values of Fisher's exact tests and frequencies in percent for the seven most frequent clonal complexes:

	p-value	Percentage
CC15	0.28	9
CC22	0.01	7
CC30	1.00	12

CC45	0.13	15
CC5	0.83	16
CC7	0.28	9
CC8	0.78	8

The p-value of the Chi-squared test for agr type is 0.84. Frequency distribution:

Osteoarticular infection	agrI	agrII	agrIII	agrIV	Sum
No	134	81	42	6	263
Yes	29	13	7	1	50
Sum	163	94	49	7	313
